# Molecular design, synthesis and *in vitro* biological evaluation of thienopyrimidine–hydroxamic acids as chimeric kinase HDAC inhibitors: a challenging approach to combat cancer

**DOI:** 10.1080/14756366.2021.1933465

**Published:** 2021-06-30

**Authors:** Mona M. Abdel-Atty, Nahla A. Farag, Rabah A. T. Serya, Khaled A. M. Abouzid, Samar Mowafy

**Affiliations:** a Pharmaceutical Chemistry Department, Faculty of Pharmacy, Misr International University, Cairo, Egypt; b Pharmaceutical Chemistry Department, Faculty of Pharmacy, Ain Shams University, Cairo, Egypt; c Organic and Medicinal Chemistry Department, Faculty of Pharmacy, University of Sadat City, Sadat City, Egypt; d Department of Chemistry, University of Washington, Seattle, WA, USA

**Keywords:** Thieno[2,3-d]pyrimidine; hydroxamic acid derivatives; chimeric HDAC-kinase inhibitors; multitarget therapy lead; ADMET study

## Abstract

A series of thieno[2,3-*d*]pyrimidine-based hydroxamic acid hybrids was designed and synthesised as multitarget anti-cancer agents, through incorporating the pharmacophore of EGFR, VEGFR2 into the inhibitory functionality of HDAC6. Three compounds **(12c, 15b and 20b)** were promising hits, whereas **(12c)** exhibited potent VEGFR2 inhibition (IC_50_=185 nM), potent EGFR inhibition (IC_50_=1.14 µM), and mild HDAC6 inhibition (23% inhibition). Moreover, compound **(15c)** was the most potent dual inhibitor among all the synthesised compounds, as it exhibited potent EGFR and VEGFR2 inhibition (IC_50_=19 nM) and (IC_50_=5.58 µM), respectively. While compounds **(20d)** and **(7c)** displayed nanomolar selective kinase inhibition with EGFR IC_50_= 68 nM and VEGFR2 IC_50_= 191 nM, respectively. All of the synthesised compounds were screened *in vitro* for their cytotoxic effect on 60 human NCI tumour cell lines. Additionally, molecular docking studies and ADMET studies were carried out to gain further insight into their binding mode and predict the pharmacokinetic properties of all the synthesised inhibitors.

## 1. Introduction

**Figure 1.**
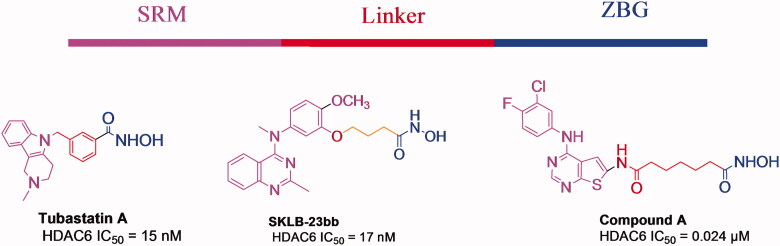
The structures of histone deacetylase 6 (HDAC6) inhibitors, colour codes show essential pharmacophore for HDAC inhibitors composed of zinc-binding group (blue), linker (red), and SRM (purple).

The development of multitarget drug therapy has become an important strategy for cancer treatment. Recently, the modulation of receptor tyrosine kinase (RTK) pathways by the inhibition of histone deacetylases (HDACs) has become a promising approach for cancer therapy^1^. HDACs are a family of numerous epigenetic enzymes that are important therapeutic targets for cancer^2^. HDACs have been classified into four distinct classes, in which class I (HDAC1–3 and 8), class II (HDAC4–7, 9 and 10), and class IV (HDAC11)^3^. HDAC6 is considered a therapeutically important target for cancer treatment due to its interaction with proteins involved in cell growth, migration, protein degradation, and apoptosis^4^. It is reported that HDAC inhibitors with large or rigid hydrophobic surface recognition moiety (SRM) and bulky aromatic or short linkers, are more efficient to achieve HDAC6 selectivity, which could achieve a closer approach of the hydroxamate group to Zn^2+^^5^. Numerous HDAC6 inhibitors have been developed having large SRM connected to the zinc-binding group (ZBG) with diverse linkers (Figure 1)^5–7^.

**Figure 2.**
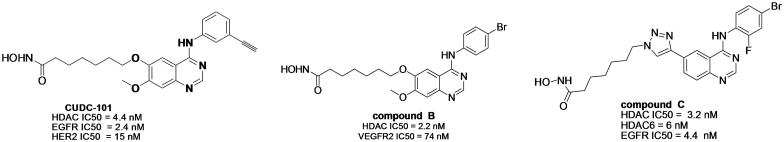
The structures of several reported dual inhibitors HDAC/EGFR or VEGFR2.

Despite the efficiency of HDAC inhibition as monotherapy in haematologic malignancies, it did not show significant competency against solid tumours. As a result, multitarget therapy is developed as a challenging approach for increasing the efficacy of HDAC inhibitors^8^. The combination of HDAC and TKIs is considered a promising approach to enhance therapeutic effect and repress resistance^1^. For example, a synergistic effect is observed by a combination of gefitinib (EGFR inhibitor) with vorinostat (HDAC inhibitor) by enhancing tumour growth inhibition and apoptosis compared with monotherapy of selective EGFR inhibitors^9^^,^^10^. Another example, combination therapy of pazopanib (VEGFR2 inhibitor) and abexinostat (HDAC inhibitor) showed that HDAC inhibition could improve response and holdback resistance to pazopanib in patients with solid tumour malignancies and renal cell carcinoma^11^. Thus, it is suggested that a single molecule that simultaneously inhibits HDAC and RTK activities targeting multiple biological molecules and multiple signal pathways can not only enhance drug efficacy but also can have additive or even synergistic antitumor effects and can overcome the problems of cancer resistance and relapse. Additionally, it can overcome unfavourable pharmacokinetic properties, drug–drug interactions, poor patient compliance, and high drug cost involved in multicomponent drug cocktails^1^^,^^12^. CUDC-101 is a first-in-class multi-targeted hybrid that was designed to simultaneously inhibit HDACs, EGFR, and HER2. It was found that it induces significantly synergistic tumour regression in breast, NSCLC, liver, colon, head, neck, and pancreatic cancers^1^. Series of hybrids of 4-anilinoquinaziline based hydroxamic acid derivatives were synthesised and discovered as dual VEGFR-2/HDAC inhibitors and EGFR/HDAC inhibitors (Figure 2)^13^^,^^14^.

**Figure 3.**
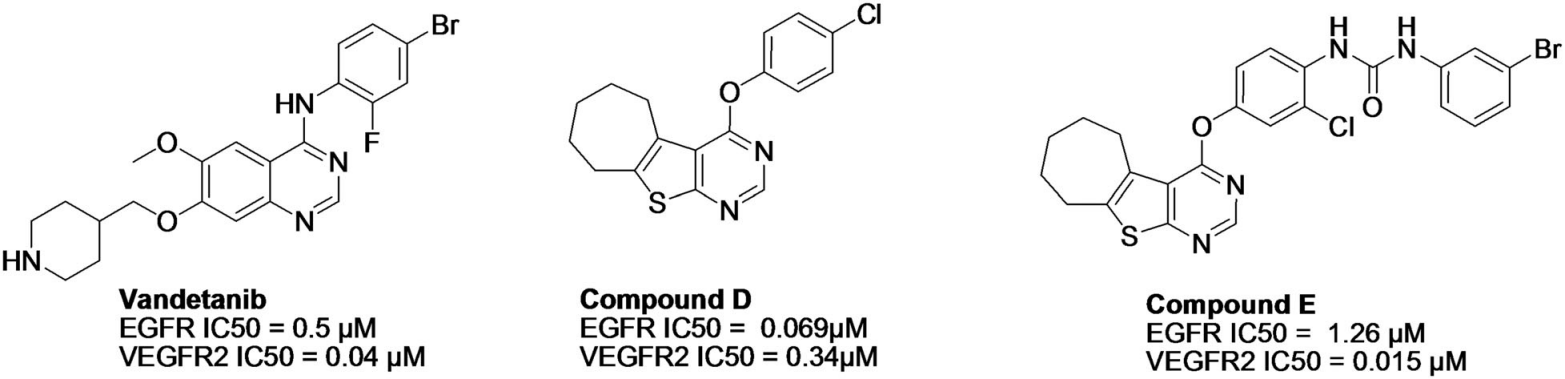
Examples of dual inhibitors acting on EGFR and VEGFR2.

Moreover, the development of dual-inhibitors targeting EGFR and VEGFR2 has recently become a promising strategy for the treatment of diverse types of cancer to overcome problematic drug resistance and low response to selective tyrosine kinase inhibitors (TKIs) and to produce a synergistic effect^15^. Inhibition of both EGFR and VEGFR2 produces a more significant effect on angiogenesis than selective VEGFR2 inhibitors, where EGFR-induced the production of angiogenic growth factors, which indirectly affects angiogenesis^16^^,^^17^. Several EGFR/VEGFR-2 dual inhibitors have been designed and synthesised (Figure 3)^15^^,^^16^.

In this study, six novel series of thieno[2,3-*d*]pyrimidine-based hydroxamic acid derivatives were rationally designed. The design is based on the strategy of multitarget drug therapy and the clinical advantage of combining HDAC, VEGFR2, or/and EGFR inhibition in one single drug. Investigating the structural requirements of EGFR, VEGFR2, and HDAC inhibitors led us to design several series of novel leads of multitarget or dual-target inhibitors by combining the pharmacophore of EGFR, VEGFR2 inhibitors with that of HDAC inhibitors.

## 2. Rationale of the design

Design strategy is based on the study of several reported dual inhibitors such as CUDC101 and compound B, which were reported as dual EGFR/HDAC and VEGFR2/HDAC inhibitors respectively; (bearing 4-anilinoquinaziline fragment) (Figure 2)^14^^,^^18^. Additionally, novel dual EGFR/VEGFR2 inhibitors such as compound D and compound E; (bearing thieno[2,3-*d*] pyrimidine fragment;) were recently reported (Figure 3)^15^.

Based on these reported dual inhibitors and by investigating the structural requirements for EGFR, VEGFR2, and HDAC inhibition, we designed six series of hybrid molecules **(7, 11, 12, 15, 19 and 20)** with potent multi acting EGFR/VEGFR/HDAC inhibition in an attempt to synergise inhibitory activity, decrease resistance and relapse caused by single-target therapy and also avoid multicomponent drug cocktails therapy.

**Figure 4.**
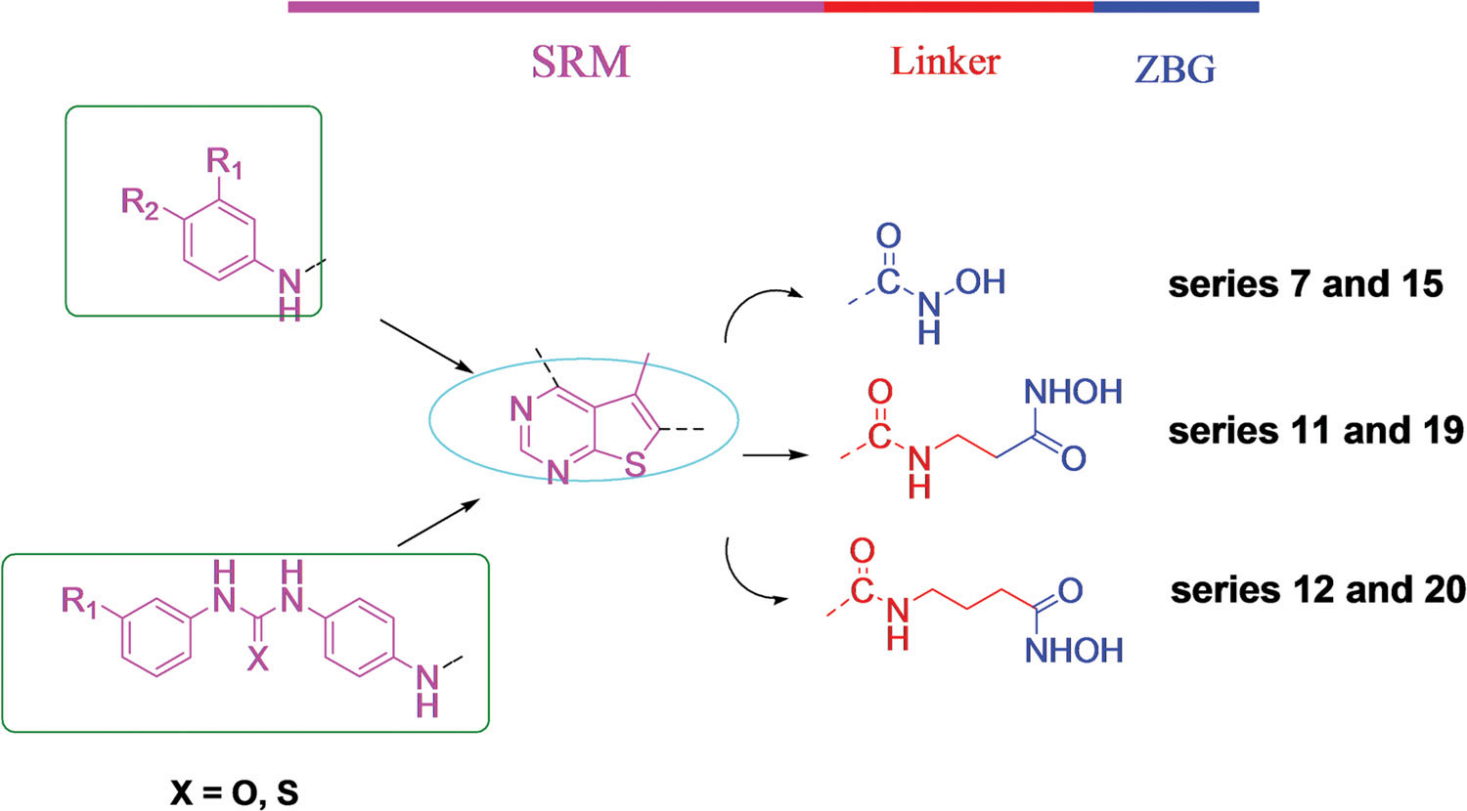
Design strategy of hybrid multitarget (EGFR, VEGFR, and HDAC inhibitors). Colour codes show essential pharmacophore for HDAC inhibitors composed of surface recognition moiety (purple), linker (red), and zinc-binding group (blue). The cyan oval shape represents the area binding to the hinge region of the ATP binding site of VEGFR2 and EGFR and the green square represents the area binding to the hydrophobic pocket of VEGFR2 and EGFR, while the rest of the structure represents the area fitting in the solvent-accessible area of VEGFR2 and EGFR.

The design aimed at replacing the quinazoline fragment in CUDC101 and compound B with thieno[2,3-*d*]pyrimidine fragment as in compound D and compound E, representing SRM of HDAC inhibitors and also showing suitable interactions in the hinge region of ATP-binding site of EGFR and VEGFR2 (Figure 4). This bioisostere replacement is also supported by recent reports of novel HDAC inhibitors bearing thieno[2,3-*d*]pyrimidine fragments representing the SRM that displayed excellent inhibitory activities on HDACs, such as compound A as shown in (Figure 1)^7^. Addition of aniline, 5-aminobenzimidazole, (4-phenylthioureido)phenylamine) and (4-phenylureido)phenylamine) fragments to 4-thieno[2,3-d]pyrimidine ring will extend the structure to the hydrophobic pocket of EGFR and VEGFR2 (Figure 4). Next, a hydroxamic acid functionality was directly attached to the thieno[2,3-*d*]pyrimidine fragment (SRM) or connected with a short linker through a connecting amide unit as the previously reported HDAC6 inhibitors (Figure 1)^19^^,^^20^, in which it served as a bridge to properly display HDAC6 inhibitory pharmacophore features and the solvent-accessible area of EGFR- and VEGFR2-binding sites (Figure 4).

## 3. Results and discussion

### 3.1. Chemistry

**Scheme 1.**
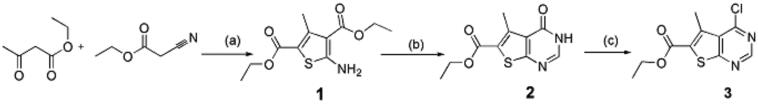
Synthesis of. Ethyl (4-chloro-5-methylthieno[2,3-d]pyrimidine)-6-carboxylate. Reagents and conditions: (a) Morpholine, S, EtOH, 70 °C, 4 h, 0 °C, 24 h; (b) Formamide, acetic acid, 40 h., 150 °C; (c) POCl_3,_ reflux, 3 h.

**Scheme 2.**
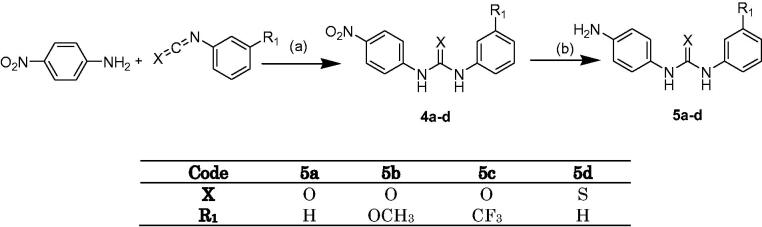
Synthesis of 1–(4-aminophenyl)-3-phenylurea and 1–(4-aminophenyl)-3-phenylthiourea derivatives. Reagents and conditions: (a) DCM, rt, 24 h. (b) H_2_, Pd/C, MeOH, rt, 4 h.

**Scheme 3.**
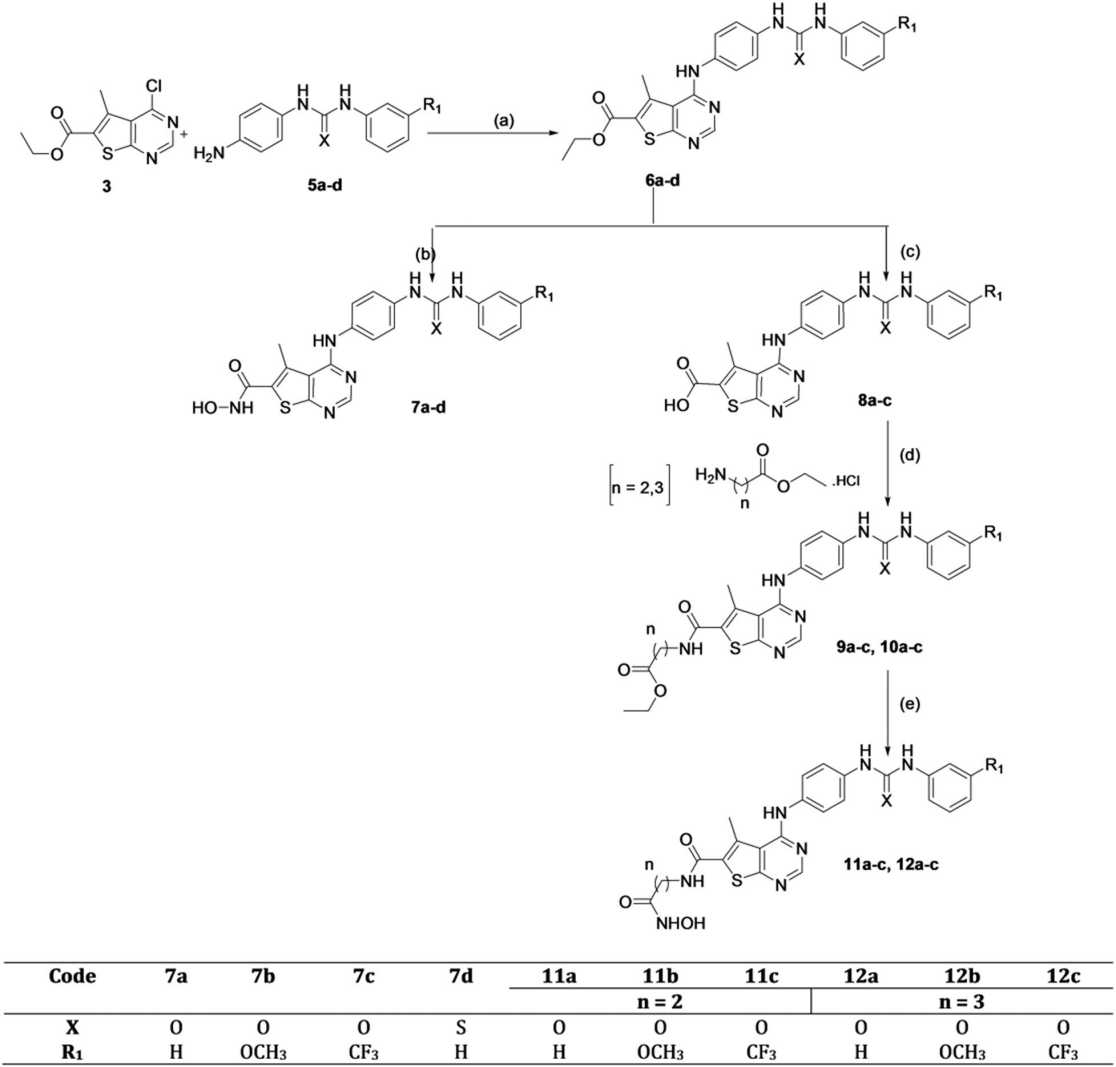
Synthesis of phenyl ureido phenyl amino thieno [2,3-d] pyrimidine hydroxamic acid derivatives. Reagents and conditions: (a) EtOH, TEA, reflux, 18–48 h; (b) NH_2_OH.HCl, MeOH,/Na, 0 °C; (c) Li(OH)_2_, THF, reflux, 7 h; (d) EDCI/HOBt, NMM, DMF, 0 °C, rt, 24 h; (e) NH_2_OH.HCl, EtOH/Na, 0 °C.

**Scheme 4.**
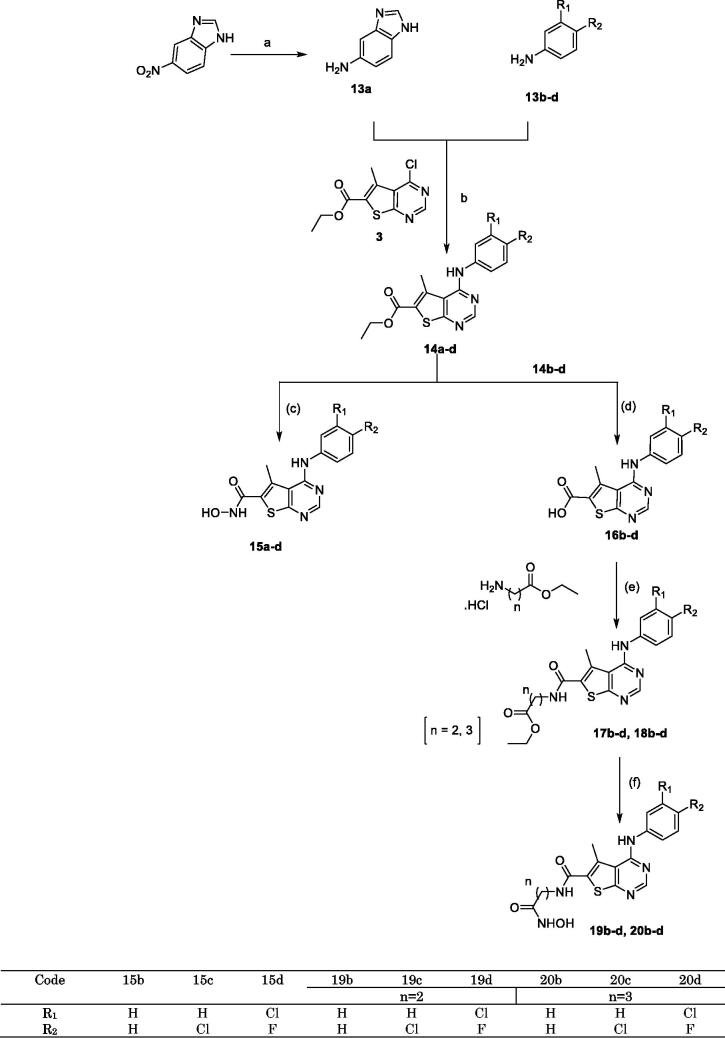
Synthesis of 5-amino benzimidazole thieno [2,3-d] pyrimidine hydroxamic acid derivative and 4-anilino thieno [2,3-d] pyrimidine hydroxamic acid derivatives. Reagents and conditions: (a) H_2_, Pd/C, MeOH, rt, 6 h; (b) EtOH, TEA, reflux, 18–48 h; (c) NH_2_OH.HCl, MeOH,/Na, 0 °C; (d) Li(OH)_2_, THF, reflux, 7 h; (e) EDCI/HOBt, NMM, DMF, 0 °C, rt, 24 h; (f) NH_2_OH.HCl, EtOH/Na, 0 °C.

The route adopted for the synthesis of key intermediates **(3)** and **(5a-d)** were prepared according to literature as illustrated in (Schemes 1 and 2), respectively. The synthetic route of the first three series of the target novel hydroxamic acid derivatives with different urea and thiourea moieties **(7a-d, 11a-c, 12a-c)** is depicted in (Scheme 3). Moreover, the synthesis of another three series of the novel target hydroxamic acid derivatives with different aniline moieties (**15a-d, 19 b-d, 20 b-d**) is depicted in (Scheme 4).

In (Scheme 1), intermediate **(1)** was synthesised by applying the Gewald reaction, in which ethyl cyanoacetate, ethyl acetoacetate, and sulphur were dissolved in absolute ethanol in the presence of morpholine^21^. The cyclisation of **(1)** into **(2)** was achieved through refluxing **(1)** in a mixture of formamide and acetic acid for 40 h. The structure was confirmed by its reported melting point. Compound **(3)** was synthesised through chlorination of **(2)** by refluxing it with phosphorus oxychloride for 3 h^22^, (Scheme 1).

The synthesis of the intermediates **(4a–d)** was carried out by reacting the appropriate isocyanate with p-nitroaniline in dry dichloromethane for 48 h at room temperature^23^ to yield compounds **(4a–d)**, which are then reduced to their corresponding amino derivatives **(5a-d)** using 10% Pd-C in methanol^24^, (Scheme 2).

In (Scheme 3), the synthesised key intermediates **(5a–d)** were then coupled with the previously prepared chloro derivative **(3)**^25^ to yield **(6a-d).** The first novel series of the target hydroxamic acid derivatives **(7a-d)** was obtained through the reaction of the ethyl ester group in compounds **(6a-d)** with freshly prepared hydroxylamine in absolute ethanol to give **(7a-d)**^26^^,^^27^, (Scheme 3). Compounds **(7a-d)** were confirmed by many spectral data, in which ^1^HNMR signals were consistent with protons of the targeted compounds **(7a-d).** ^1^HNMR signals revealed equally integrated signals between *δ* 8.23–9.82 ppm representing D_2_O exchangeable protons of the NH linker, the urea protons, and the NH of hydroxamic acid moiety in all target compounds **(7a-d).** Noticeably, ^1^HNMR signals revealed the absence of the triplet and the quartette peak around *δ* 1.31 − 1.36 ppm and *δ* 4.30–4.38 ppm, which confirms the absence of the ester group and the entrance of hydroxamic acid moiety.

Moreover, the synthesis of another two novel series of hydroxamic acid derivatives with urea and thiourea moieties **(11a-c** and **12a-c)** was depicted in (Scheme 3), through multistep reactions. The synthesis starts with the hydrolysis of the carboxylate ethyl ester of the previously prepared compounds **(6a-c)** (Scheme 3), through refluxing it with Li(OH)_2_ in THF and water to prepare the intermediate carboxylic acid derivatives **(8a-c)**^28^. Furthermore, the synthesis of novel two series of intermediate ethyl ester derivatives **(9a-c, 10a-c)** (Scheme 3) was achieved through coupling of the previously prepared carboxylic acid derivatives **(8a-c)** with the appropriate amino acid ester hydrochloride salt, utilising EDCI with HOBt in dry DMF^29^. This method offered a high yield of novel intermediate compounds **(9a-c and 10a-c)** confirmed by many spectral data. ^1^HNMR signals confirmed the prepared structures of the novel esters, in which the aliphatic protons were shown as quartette signals for synthesised novel esters, where it appeared around *δ* 4.05–4.25 ppm representing the two protons of OCH_2_-CH_3_ with *J* = 8 Hz. Another quartette signal around *δ* 3.26–3.78 ppm appeared in all compounds referring to two protons of CH_2_-NH with *J* = 8 Hz. Triplet signal appeared around *δ* 2.36–2.71 ppm for all compounds, representing the two protons of CH_2_-CO with *J* = 8 Hz. Moreover, another triplet signal appeared for all compounds around *δ* 1.17–1.34 ppm, representing three protons of OCH_2_-CH_3_ with *J* = 8 Hz. An additional multiplet signal appeared around *δ* 1.76–2.04 ppm, representing two protons of CH_2_-CH_2_-CH_2_ in all prepared ethyl ester derivatives of series **(10a-c)** (Scheme 3).

Finally, the ethyl ester group in the previously prepared novel compounds **(9a-c, 10a-c)** reacted with freshly prepared hydroxylamine in absolute ethanol to obtain target novel hydroxamic acid derivatives **(11a-c, 12a-c)** (Scheme 3). The two target novel series **(11a-c, 12a-c)** were confirmed by many spectral data, where ^1^HNMR spectrum revealed the disappearance of the quartette signal of the two protons of OCH_2_-CH_3_ around *δ* 4.05–4.25 and the triplet signal of the three protons OCH_2_-CH_3_ around *δ* 1.17–1.34 ppm, in which it confirms the absence of the ester group and the entrance of the hydroxamic acid moiety.

In (Scheme 4), the purchased compound (5-nitrobenzimidazole) was reduced to afford the corresponding amino derivative **(13a)** using 10% Pd-C in methanol. Prepared compound **(13a)** and purchased aniline derivatives **(13b-d)** were also coupled with the previously prepared chloro derivative **(3)** to give the corresponding thieno[2,3-*d*]pyrimidine derivatives bearing aniline moieties **(14a-d)** (Scheme 4). Finally, the route adopted for the synthesis of the three target novel series of hydroxamic acid derivatives bearing aniline moiety **(15a-d, 19 b-d and 20 b-d)** (Scheme 4) is the same route depicted in (Scheme 3) with the same reagents and conditions. The first novel series of the target hydroxamic acid derivatives **(15a-d)** was obtained through the reaction of the ethyl ester group in compounds **(14a-d)** with freshly prepared hydroxylamine in absolute ethanol to give **(15a-d)**^26^^,^^27^, (Scheme 4). Then, the hydrolysis of the carboxylate ethyl ester of the previously prepared compounds **(14b-d)** (Scheme 4), through refluxing it with Li(OH)_2_ in THF and water to prepare the intermediate carboxylic acid derivatives **(16b-d)**^28^. The previously prepared carboxylic acid derivatives **(16b-d)** are coupled with the appropriate amino acid ester hydrochloride salt, utilising EDCI with HOBt in dry DMF^29^, to obtain a high yield of novel intermediate compounds **(17b-d and 18b-d)**, (Scheme 4). Finally, the ethyl ester group in the previously prepared novel compounds **(17b-d, 18b-d)** reacted with freshly prepared hydroxylamine in absolute ethanol to obtain target novel hydroxamic acid derivatives **(19b-d, 20b-d)** confirmed by many spectral data, (Scheme 4).

### 3.2. Biological evaluation

**Figure 5.**
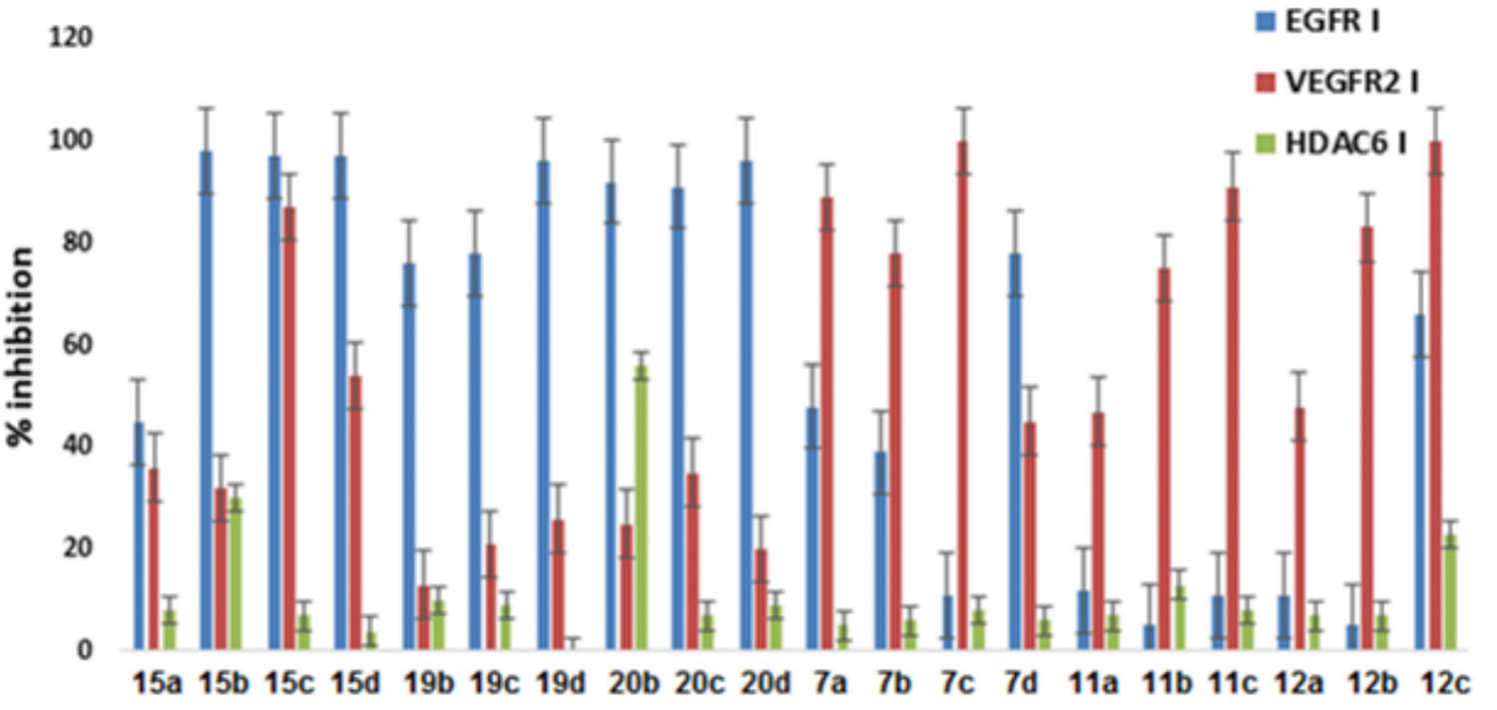
Mean % inhibition of the investigated compounds on EGFR, VEGFR-2 and HDAC6 at 10 µM concentration.

The target compounds were evaluated for their *in vitro* inhibitory activities against EGFR, VEGFR-2, and HDAC6. The EGFR and VEGFR-2 inhibitory assays were performed at Thermo Fischer Scientific, Madison, WI, USA (SelectScreenServices@thermofisher.com). The HDAC inhibitory assays were performed at BPS Bioscience, San Diego, CA, USA (www.bpsbioscience.com). Initially, all of the synthesised target compounds were evaluated for their percent inhibitory activity against EGFR, VEGFR-2, and HDACs at 10 µM single dose concentration, in which their mean percent inhibition is summarised in (Figure 5, Tables 1 and 2). Representative compounds were evaluated for their percent inhibitory activity against EGFR and VEGFR-2 at 6 different concentrations (0.1 nM, 1 nM, 10 nM, 100 nM, 1 µM, 10 µM) to subsequently calculate their IC_50_ values (Table 3). Additionally, all of the synthesised target compounds were submitted to the National Cancer Institute “NCI” Bethesda, Maryland, USA (www.dtp.nci.nih.gov), to evaluate theirs *in vitro* antiproliferative activities against NCI 60- cancer cell line panel.

**Table 1.** Mean % inhibition of the investigated compounds on EGFR, VEGFR-2 and HDAC6 at 10 µM concentration. 
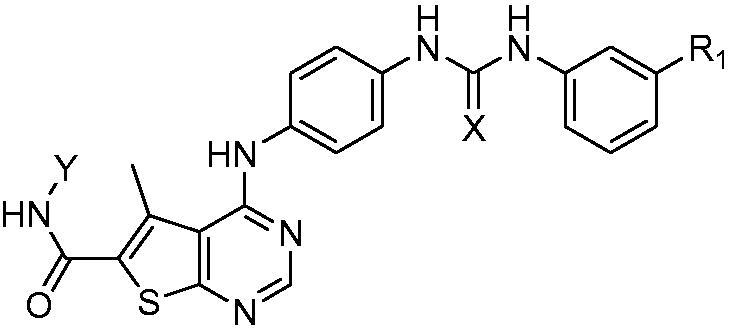

Code	R1	X	Y	EGFR	VEGFR-2	HDAC6
**7a**	H	O	OH	**48%**	**89%**	**5%**
**7b**	OCH_3_	O	OH	**39%**	**78%**	**6%**
**7c**	CF_3_	O	OH	**11%**	**100%**	**8%**
**7d**	H	S	OH	**78%**	**45%**	**6%**
**11a**	H	O		**12%**	**47%**	**7%**
**11b**	OCH_3_	O		**5%**	**75%**	**13%**
**11c**	CF_3_	O		**11%**	**91%**	**8%**
**12a**	H	O		**11%**	**48%**	**7%**
**12b**	OCH_3_	O		**5%**	**83%**	**7%**
**12c**	CF_3_	O		**66%**	**100%**	**23%**

Red-coloured data represent potent inhibition 70–100%, green-coloured data represent moderate inhibition 30–70%, blue-coloured data represent mild inhibition 20–30%, and the black-coloured data represent very weak inhibition <20%.

**Table 3.** The IC_50_ value of representative synthesised compounds against EGFR and VEGFR-2. 
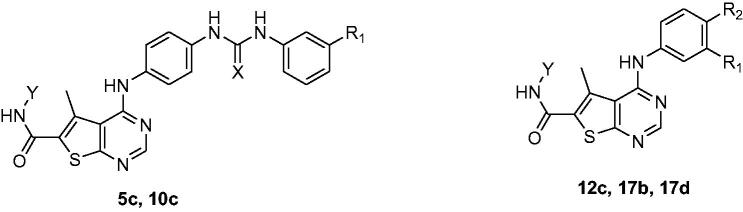

Compound					IC_50_ in enzyme assays		
Code	R1	R2	X	Y	EGFR		VEGFR-2
**7c**	CF_3_	_–_	O	OH	ND		191 nM
**12c**	CF_3_	_–_	O		1.14 µM		185 nM
**15c**	H	Cl	_–_	OH	19 nM		5.58 µM
**20b**	H	H	_–_		155 nM		ND
**20d**	Cl	F	_–_		68 nM		ND
**Gefitnib [**27**]**					1.08 µM		ND
**Vandetanib [**17**]**					0.5 µM		40 nM

ND: not determined.

#### 3.2.1. *In vitro* EGFR inhibitory assay

By closely investigating the structure activity relationship, thieno[2,3-d]pyrimidine derivatives with aniline moiety showed an excellent inhibitory profile compared to derivatives with urea and benzimidazole moieties (Tables 1 and 2). Moreover, thieno[2,3-d]pyrimidine derivatives with aniline moiety, mono-substitutions, or di-substitutions at 3- or 4- positions on its terminal phenyl ring exhibited excellent inhibitory profile, as previously reported^30^.

In general, EGFR inhibition increases by increasing the number of carbons in the linker chain between the hydroxamic acid moiety and the amide group to three carbons or by directly attaching the hydroxamic acid with the thienopyrimidine fragment. So that compounds **(20b and 20c)** showed 92 and 96% inhibition, compared to inhibition of 76 and 78% for the two carbons linker of **(19b and 19c),** respectively (Table 2).

**Table 2.** Mean % inhibition of the investigated compounds on EGFR, VEGFR-2, and HDAC6 at 10 µM concentration. 
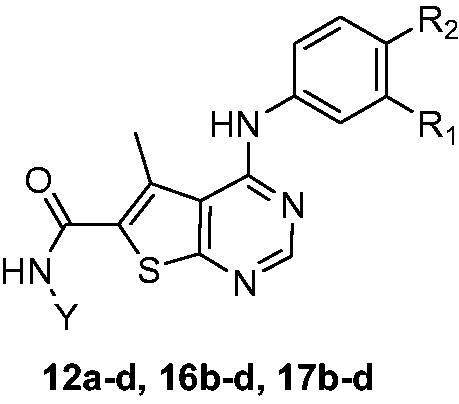

Code	R1	R2	Y	EGFR	VEGFR-2	HDAC6
**15a**			OH	**45%**	**36%**	**8%**
**15b**	H	H	OH	**98%**	**32%**	**30%**
**15c**	H	Cl	OH	**97%**	**87%**	**7%**
**15d**	Cl	F	OH	**97%**	**54%**	**4%**
**19b**	H	H		**76%**	**13%**	**10%**
**19c**	H	Cl		**78%**	**21%**	**9%**
**19d**	Cl	F		**96%**	**26%**	**0%**
**20b**	H	H		**92%**	**25%**	**56%**
**20c**	H	Cl		**91%**	**35%**	**7%**
**20d**	Cl	F		**96%**	**20%**	**9%**

Red-coloured data represent potent inhibition 70–100%, green-coloured data represent moderate inhibition 30–70%, blue-coloured data represent mild inhibition 20–30%, and the black-coloured data represent very weak inhibition <20%.

Remarkably, compound **(12c)** bearing urea moiety with 3-CF_3_ at its terminal phenyl ring showed an exceptional significant inhibitory activity towards EGFR kinase activity (66%), compared to other thieno[2,3-d]pyrimidine derivatives bearing urea moiety. Also, compound **(7d)** bearing thiourea demonstrated noticeable higher inhibitory activity (78% inhibition) than compound **(7a)** bearing urea moiety (48% inhibition).

Promising candidates, **(12c, 15c, 20b and 20d**) were chosen as representatives for evaluation of potential enzyme inhibitory activity (IC_50_) (Table 3). Compound **(15c)** demonstrated the most potent EGFR inhibition with IC_50_ value of 19 nM since its hydroxyl amine group is directly attached to the thieno[2,3-d]pyrimidine ring and also it has 4-Cl substitution on its terminal phenyl ring. Compound **(20d)** exhibited more potent inhibition than **(20b)** with IC_50_ value of 68 nM compared to 155 nM. Since both **(20d and 20b)** have a linker of 3 carbons, thus the (4-F, 3-Cl) substitution at the terminal phenyl ring of compound **(20d)** is suggested to be responsible for the increase of its inhibitory activity compared to **(20b),** with no substitution at its terminal phenyl ring.

Noticeably, compound **(12c)** bearing urea moiety with 3-CF_3_ at its terminal phenyl ring exhibited IC_50_ value of 1.14 µM, in which it showed the highest EGFR inhibition among all other synthesised derivatives bearing urea moiety.

#### 3.2.2. *In vitro* VEGFR-2 inhibitory assay

On the contrary of EGFR inhibitory assay, most of the thieno[2,3-d]pyrimidine derivatives bearing urea moiety (**7a-c, 11b,c, 12b,c)** have demonstrated potent inhibition ranging from (75-100%) for VEGFR-2 kinase activity at an initial screening of 10 μM concentration. Compounds with 3-CF_3_ substitution at its terminal phenyl ring **(7c, 11c and 12c)** demonstrated the most potent VEGFR-2 inhibition by 100, 91 and 100%, respectively (Table 1). Moreover, by observing the effect of the hydrocarbon linker on the VEGFR-2 inhibitory activity, it was found that the most potent inhibitory activities were observed with derivatives bearing urea moieties having hydroxylamine moiety directly attached to the thieno[2,3-d]pyrimidine ring, such as **(7a, 7b and 7c)** exhibited 89, 78 and 100% inhibition, respectively. Noticeably, compound **(7a)** bearing a urea moiety showed higher VEGFR-2 inhibition (89% inhibition) than compound **(7d)** bearing thiourea moiety (45% inhibition) (Table 1).

As for the aniline series, compound **(17c)** with no linear hydrocarbon linker between the hydroxamic acid moiety and the thieno[2,3-*d*]pyrimidine fragment and 4-chloro substitution on its terminal phenyl ring showed exceptional potent inhibition (87% inhibition) (Table 2).

Promising candidates, **(7c, 12c, and 15c)** were chosen as representatives for the evaluation of potential enzyme inhibitory activity (IC_50_) (Table 3). All of the evaluated target compounds exhibited potent VEGFR-2 inhibitory activity with IC_50_ values ranging from (185 nM to 5.58 µM). Compounds **(7c and 12c)** bearing urea moiety exhibited potent VEGFR-2 inhibition with IC_50_ values of 191 and 185 nM, respectively. These results suggest that -CF_3_ substitution at the terminal phenyl ring of compounds **(7c and 12c)** and the urea moiety is responsible for their high inhibitory activity, regardless of having 3 carbons in the linker chain **(12c)** or without having a linker **(7c).** Compound **(15c)** with 4-chloro aniline moiety exhibited significant inhibition to VEGFR-2 with a higher IC_50_ value of 5.58 µM than other derivatives bearing urea moiety.

#### 3.2.3. *In vitro* HDAC inhibitory assay

Initially, all synthesised compounds were screened against HDAC6 at 10 μM concentration. HDAC6 enzyme inhibition assay was selected since our newly synthesised hydroxamic acid compounds have a large capping group suitable for fitting in the large hydrophobic channel of HDAC6, which is unique to the HDAC6 receptor^26^. The newly designed compounds were synthesized either having short hydrocarbon linkers between the zinc-binding group (ZBG) and the connecting amide unit or having no linear hydrocarbon linker between the hydroxamic acid moiety and the thieno[2,3-*d*]pyrimidine fragment, as the previously reported potent HDAC6 inhibitors^19^^,^^20^.

Screening of the synthesised compounds against HDAC6 at 10 μM concentration is shown in (Tables 1 and 2). For derivatives bearing urea moiety, compound **(12c)** with three carbons in the linker region showed considerable HDAC6 inhibition by 23% (Table 1). For derivatives bearing aniline moiety, compound **(20b)** exhibited the most significant HDAC6 inhibition by 56% (Table 2). Compound **(20b);** bearing unsubstituted aniline moiety; is the smallest capping group among all synthesized compounds. The small capping group may allow it to fit in the HDAC6 hydrophobic channel. Additionally, compound **(20b)** had 3 carbons in the linker region compared to **(15b)** with no linear hydrocarbon linker and **(19b)** with 2 carbons in the linker region showing 30% and 10% inhibition, respectively (Table 2). Thus, having a small capping group of unsubstituted aniline and a longer carbon chain in the linker region between the hydroxamic acid moiety and the connecting amide is recommended for optimum HDAC6 inhibition.

In conclusion, potential novel multitarget hybrid lead inhibitors acting on EGFR, VEGFR-2 and HDAC6 are considered, such as compounds **(12c, 15 b and 20 b).** Compound **(12c)** exhibited potent VEGFR-2 inhibition (100%, IC_50_ = 185 nM), significant EGFR inhibition (66%, IC_50_ =1.14 µM) and mild HDAC6 inhibition (23%). Compound **(15 b)** exhibited potent EGFR inhibition (98%), moderate VEGFR-2 (32%) and moderate HDAC6 inhibition (30%). Compound **(20 b)** exhibited potent EGFR inhibition (92%, IC_50_ = 155 nM), moderate HDAC6 inhibition (56%) and mild VEGFR-2 inhibition (25%), (Figure 5).

Moreover, many of the synthesised novel compounds can be considered as promising leads of EGFR and VEGFR-2 dual inhibitors, (Table 2 and Figure 5). Where, compound **(15c)** is considered to be the most potent dual inhibitor among all the synthesised compounds, since it exhibits potent EGFR and VEGFR-2 inhibition (97%, IC_50_ = 19 nM) and (87%, IC_50_ =5.58 µM), respectively. Compound **(15d)** exhibited potent EGFR and moderate VEGFR-2 inhibition (97%) and (54%), respectively. Two selective potent inhibitors were also considered, such as **(7c and 20d).** Compound **(7c)** is considered as a potent novel VEGFR-2 inhibitor (100% inhibition, IC_50_ = 191 nM), while compound **(20d)** is considered as a potent novel EGFR inhibitor (96% inhibition, IC_50_ = 68 nM).

#### 3.2.4. *In vitro* antiproliferative activity

All of the synthesised structures were selected and submitted to NCI based on computer modelling techniques and the degree of diversity of structures for the evaluation of their antiproliferative activity. The 20 synthesised compounds were selected with their respective NCI codes [NCS D-820157- NCS D-820176] for the inhibition percent assay at an initial single dose of 10 μM on the full NCI 60 cell panel, where the mean graph of the percent growth for all compounds on the full NCI cell panel is evaluated, compared to the untreated control cells (Supplementary Material). *In vitro* cell line inhibition % of all synthesised compounds is represented in (Table 1 of Supplementary Material).

First, in series **(7a-d)** bearing urea moiety with no linear hydrocarbon linker between the hydroxamic acid moiety and the thieno[2,3-*d*]pyrimidine fragment, compound **(7c)** with urea moiety and 3-CF_3_ at the terminal phenyl ring showed the most significant anti-proliferative activity among all of the synthesised compounds, where it exhibited inhibitory activity against the central nervous system (CNS) cancer [SNB-75 cancer cell line (46% inhibition) and SF-539 cancer cell line (38.6% inhibition)] and colon cancer KM12 cancer cell line (33.7% inhibition). **(7c)** exhibited inhibition activity also against CNS cancer [U251 cancer cell line (26.9% inhibition) and SF-268 cancer cell line (10.8% inhibition)], renal cancer UO-31 (24.7% inhibition), leukaemia MOLT-4 (11.8% inhibition), NSCLC [NCI-H522 cancer cell line (11.7% inhibition) and NCI-H23 (11.1% inhibition)] and breast cancer MCF7 cancer cell line (11.6% inhibition). While in series **(15a-d)** bearing aniline moiety with no linear hydrocarbon linker between hydroxamic acid moiety and thieno[2,3-*d*]pyrimidine fragment, compound **(15c)** with 4-Cl aniline exhibited inhibitory activity mostly against renal cancer UO-31 cancer cell line (28.8% inhibition), NSCLC [NCI-H522 cancer cell line (25.4% inhibition) and EKVX cancer cell line (20.6% inhibition)] and CNS cancer SNB-75 cell line (20.4% inhibition).

In series **(12a-c)** bearing urea moiety with three carbons in the linker chain between the hydroxamic acid moiety and the amide group, compound **(12c)** with urea moiety and CF_3_ at the terminal phenyl ring showed moderate inhibitory activity mostly against CNS cancer [SNB-75 cancer cell line (29.2% inhibition) and SF-539 cell line (28.5% inhibition)]. In series **(20 b-d**) bearing aniline moiety with three carbons in linker chain between the hydroxamic acid moiety and the amide group, compound **(20d)** exhibited inhibitory activity mostly against renal cancer UO-31 cancer cell line (23.7% inhibition), breast cancer MDA-MB-468 (16.2% inhibition) and NSCLC NCI-H226 (11.3% inhibition).

In series **(11a-c)** and **(19 b-d)** with two carbons in the linker chain between the hydroxamic acid moiety and the amide group, most of the compounds showed lower inhibitory activities against most of the investigated cell lines.

Recent studies revealed that VEGFR-2 is a major contributor to the growth of CNS tumours and its interruption may effectively suppress tumour growth in CNS cancer^31^. It was also reported that VEGFR-2 is a promising platform for anti-angiogenesis cancer therapy for colon cancer cells^32^. Also, EGFR was reported to be overexpressed in different cancer cell lines, including NSCLC^33^, renal cancer^34^, and brain cancer^35^. Noticeably, it is observed that most of the compounds showed weak to moderate inhibition towards Renal UO-31 and CNS cancer SNB-75 cell lines (20–46% inhibition).

From these findings, it is suggested that the potent *in vitro* inhibition of compound **(7c)** towards VEGFR-2 (100% inhibition, IC_50_ = 191 nM) contributed to the significant inhibitory activity against CNS [SNB-75 cancer cell line (46% inhibition) and SF-539 cancer cell line (38.6% inhibition)] and colon KM12 cancer cell line (33.7% inhibition). It is also suggested that the potent *in vitro* inhibition of compound **(15c)** towards EGFR (97% inhibition, IC_50_ = 19 nM) and VEGFR2 (87% inhibition, IC_50_ = 5.58 µM) contributed to the significant inhibitory activity against renal, CNS and NSCLC cancer cell lines as discussed. Additionally, it is also observed that **(15c)** is the only compound that showed considerable inhibition towards NSCLC H522 (25.4% inhibition) and EKVX (20.6% inhibition) cancer cell lines, which is contributed to its potent EGFR inhibition with (IC_50_ = 19 nM).

It is obvious that despite most of our newly synthesised compounds exhibited potent EGFR and/or VEGFR-2 *in vitro* inhibitory activities (90–100% inhibition) but unexpectedly some of the compounds showed moderate *in vitro* inhibitory activity against NCI cell lines at 10 µM concentration. It is suggested that maybe the increased polarity of our synthesised hydroxamic acids, might contribute to poor cell membrane permeability towards NCI cancer cell lines. Also, VEGFR-2 inhibitors showed less sensitivity towards NCI cancer cell lines than human vascular endothelial cells, which contributed to their moderate antiproliferative activity towards NCI cancer cell lines^30^.

### 3.3. Molecular modelling studies

#### 3.3.1. Docking study

A molecular docking study using Discovery Studio 4.5 was performed using the C-DOCKER algorithm. A docking study is performed to analyse binding affinities, binding modes, and orientation of the newly synthesised target compounds into the active binding site of three different receptors such as EGFR, VEGFR-2, and HDAC6. The best docking pose is selected based on its binding mode in comparison to that of the reference ligand. The crystal structure of EGFR, VEGFR-2, and HDAC6 co-crystallised with their reference ligands were downloaded from protein data bank (www.rcsb.org) with PDB codes: 4HJO, 3VHE, and 5G0H, respectively. Re-docking the reference compounds into the X-ray crystal structure of the active site of its receptor was performed for validation.

##### 3.3.1.1. Docking study on EGFR

**Figure 6.**
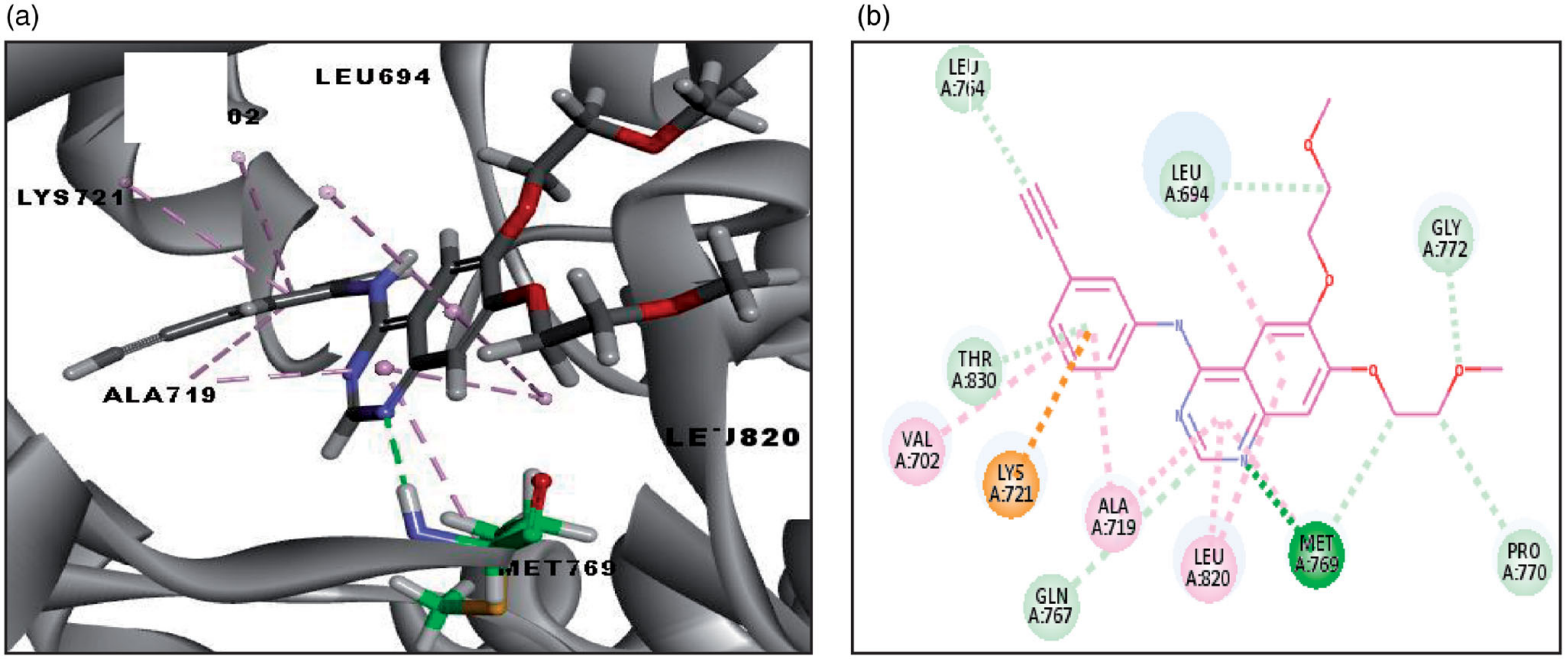
(a) 3D image of binding interaction of Erlotinib (-CDOCKER energy = 27.18) with EGFR (with PDB code: 4HJO) using Accelrys Discovery Studio 4.5. (b) 2D image of binding interaction of Erlotinib with EGFR. Hydrogen bond interaction is described as green dotted line, hydrophobic interactions are described as purple-dotted lines and electrostatic interactions are described as orange-dotted lines.

**Figure 7.**
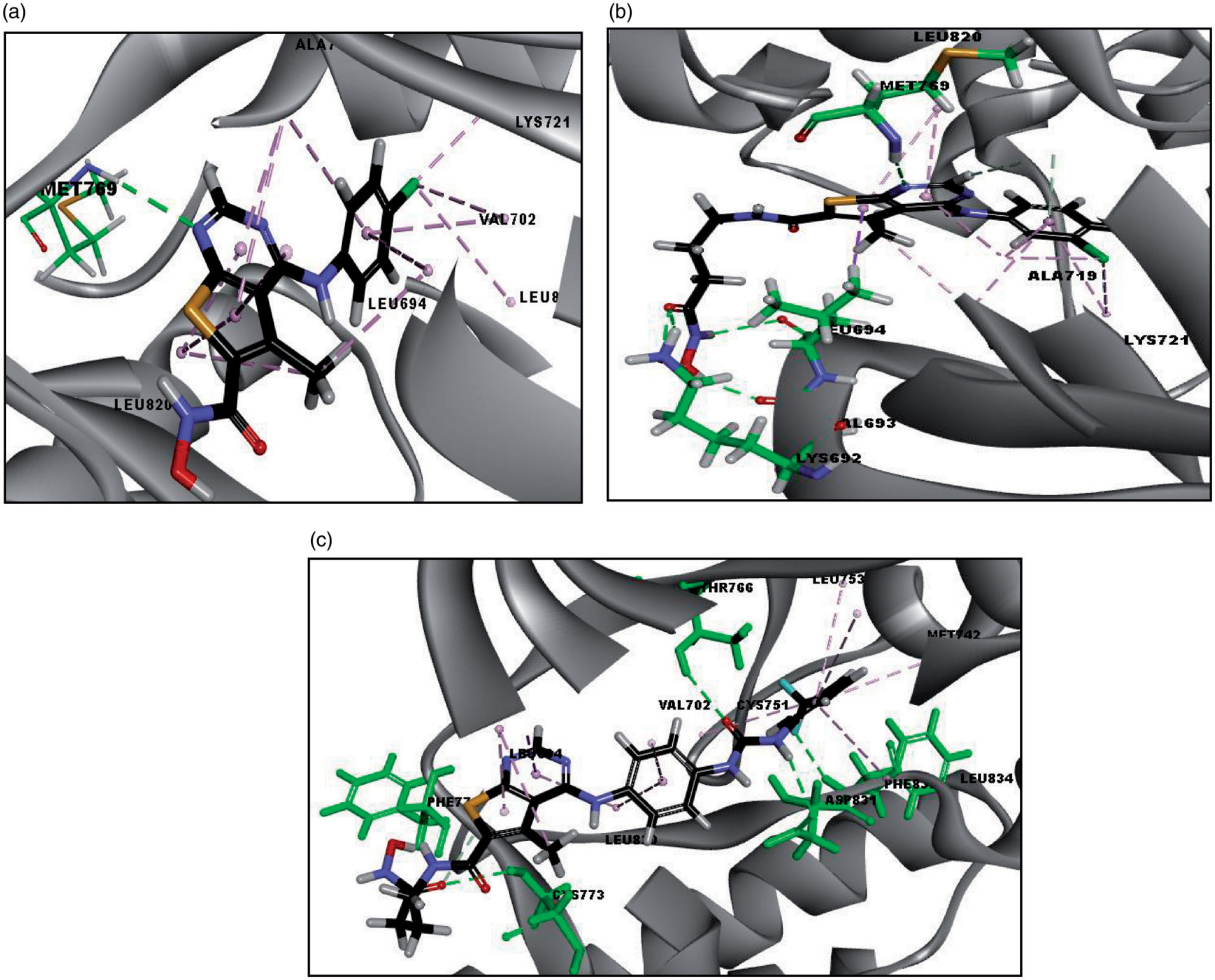
**(**a) 3D images of binding interaction of compound **(15c)** (-CDOCKER energy = 28.34) with EGFR (with PDB code: 4HJO), (b) 3D images of binding interaction of compounds **(20d)** (-CDOCKER energy = 42.52) with EGFR (c) 3D images of binding interaction of compound **(12c)** (-CDOCKER energy = 43.31) with EGFR. Hydrogen bond interaction is described as a green-dotted line, hydrophobic interactions are described as purple dotted lines and halogen bonds are described as cyan-dotted lines.

The first docking study was done on EGFR (PDB code 4HJO) co-crystallised with the reference ligand (erlotinib). The root mean square difference (RMSD) between the re-docked and the co-crystallised conformers of erlotinib is 0.5 Å, which signifies the validity of the C-DOCKER algorithm. Besides, re-docked erlotinib retrieved the reported binding mode into the active binding site of EGFR (-CDOCKER energy = 27.18 Kcal/mol) as depicted in (Figure 6)^36^. Where nitrogen of pyrimidine ring forms a hydrogen bond with NH of MET769, pyrimidine ring forms hydrophobic interactions with ALA719, MET769 and LEU820, phenyl ring forms hydrophobic interaction with LEU694 and LEU820 and the terminal phenyl ring forms hydrophobic interaction with VAL702, ALA719 and LYS721 (Figure 6). Docking of all synthesised compounds was done on EGFR (PDB code 4HJO), where the binding mode of representative thieno[2,3-*d*]pyrimidine derivatives were investigated to justify the potent or significant EGFR inhibition (high % inhibition or low IC_50_) exhibited by some of the synthesised compounds (Table 2 of Supplementary Material). In general, docking results revealed that thieno[2,3-*d*]pyrimidine derivatives with aniline moiety **(15c, 15d, 19d, 20 b-d)** showed higher binding affinities than erlotinib, fulfilled the reported binding mode and showed extra interactions with EGFR active site. Although thieno[2,3-*d*]pyrimidine derivatives with urea moiety showed higher binding affinities than erlotinib but it did not fulfil all the key interactions with the active site of EGFR. The binding mode and energies of three representatives docked compounds are presented in (Figure 7). Compound **(15c)** showed the most potent EGFR inhibitory activity (97%, IC_50_ = 19 nM) and revealed better binding affinity than erlotinib (-CDOCKER energy = 28.34 Kcal/mol). The binding mode of **(15c)** was consistent with erlotinib as shown in (Table 2 of Supplementary Material). Additionally, the methyl group of **(15c)** showed extra hydrophobic interactions with VAL702 and LEU820 and chlorine showed extra hydrophobic interactions with adjacent hydrophobic pockets including LEU764 and LEU834. Compound **(20d)** exhibited potent inhibitory activity towards EGFR (97%, IC_50_ = 68 nM) and revealed better binding affinity than erlotinib (-CDOCKER energy = 42.52 Kcal/mol). The binding mode was consistent with erlotinib as shown in (Table 2 of Supplementary Material), where the oxygen of the hydroxyl group showed 2 additional hydrogen bonds with LYS692 and VAL693, oxygen of hydroxamic carbonyl group and hydrogen of hydroxamic amine showed extra hydrogen bonds with LYS692 and LEU694, respectively. Moreover, the methyl group of **(20d)** showed additional hydrophobic interaction with VAL702, and chlorine showed additional hydrophobic interaction with ALA719 and LYS721. Compound **(12c)** bearing urea moiety exhibited significant EGFR inhibitory activity (66%, IC_50_ = 1.14 µM), but it showed lower inhibition than other compounds bearing aniline moiety. Compound **(12c)** revealed a missing hydrogen bond with the essential MET796 although having better binding affinity than erlotinib (-CDOCKER energy = 43.31 Kcal/mol). Compound **(12c)** showed five extra hydrogen bonds, where the oxygen of carbonyl urea showed hydrogen bond with THR766, oxygen of hydroxamic carbonyl group showed hydrogen bond with CYS773, oxygen of hydroxyl group showed hydrogen bond with PHE771, F of terminal phenyl ring showed hydrogen bond with PHE832 and hydrogen of urea amine showed hydrogen bond with ASP831. Additionally, the three fluorine atoms of CF_3_ showed three halogen interactions with CYS751 and one halogen interaction with ARG752. Pyrimidine ring of **(12c)** forms hydrophobic interactions with LEU694 and LEU820, thiophene ring forms hydrophobic interaction with LEU694, phenyl ring forms hydrophobic interaction with VAL702 and LEU820 and terminal phenyl ring shows four hydrophobic interactions with extra hydrophobic pocket including LEU753, LEU764, LEU834, and MET742. Moreover, a methyl group and C of CF_3_ of **(12c)** showed additional hydrophobic interactions with LEU694 and CYS751, respectively.

##### 3.3.1.2. Docking study on VEGFR-2

**Figure 8.**
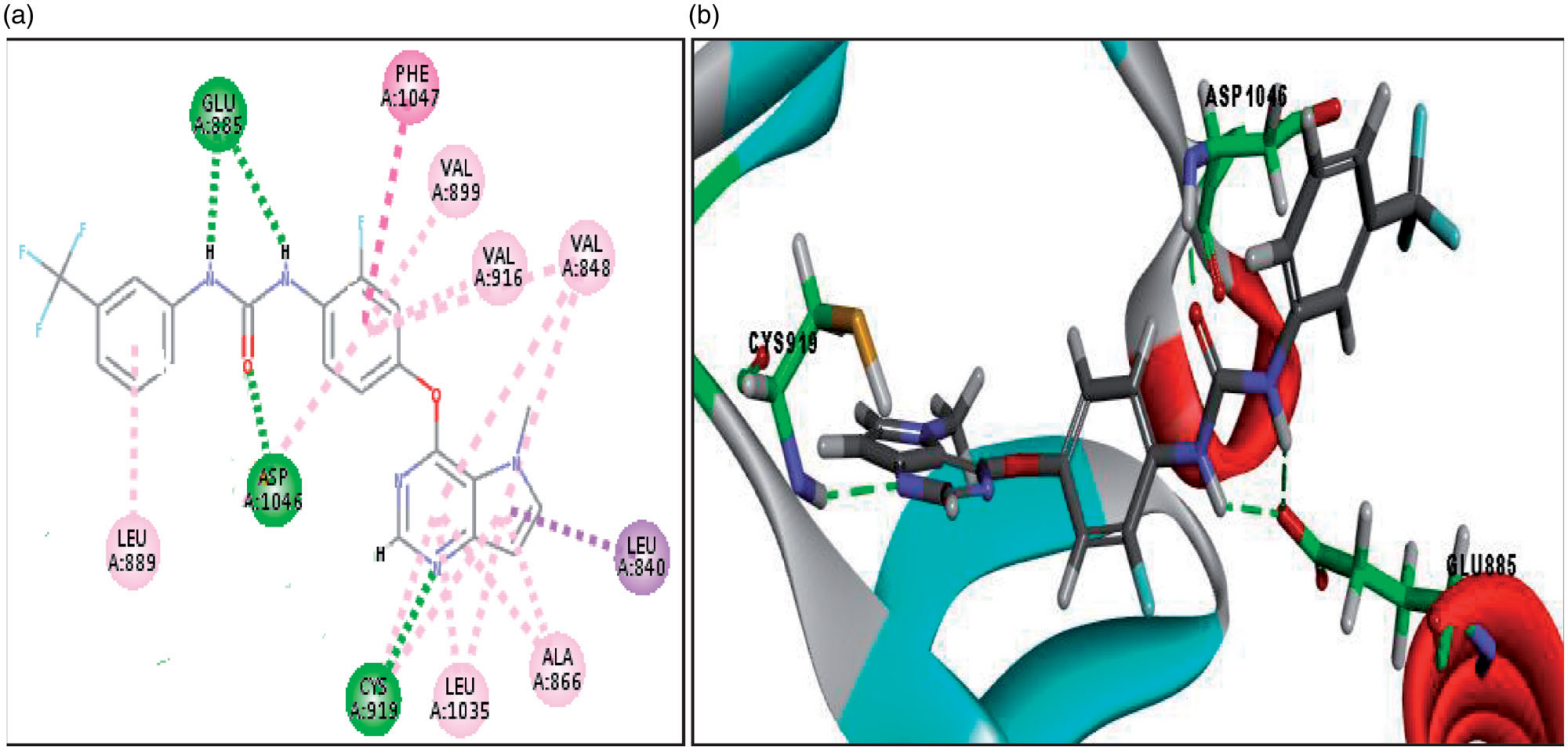
(a) 2D image of binding interaction of reference with VEGFR-2 (PDB code: 3VHE), (-CDOCKER energy = 42.91). (b) 3D image of binding interaction of reference with VEGFR-2 (with PDB code: 3VHE). Hydrogen bond interaction is described as a green-dotted line, hydrophobic interactions are described as purple dotted lines, Pi-Pi interaction is described as a pink-dotted line.

The second docking study was done on VEGFR-2 (PDB code: 3VHE) co-crystalised with the reference compound; (pyrrolo[3,2-*d*]pyrimidine derivative;). RMSD between the re-docked and the co-crystallised conformer of the reference is 0.5 Å, which proves the validity of the C-DOCKER algorithm. Noticeably, redocked pyrrolo[3,2-*d*]pyrimidine reference compound (-C-DOCKER energy = 42.91 Kcal/mol) retrieved the reported binding mode into the active site of VEGFR-2 (pdb code: 3VHE)^30^ as depicted in (Figure 8). Where nitrogen of pyrimidine ring showed hydrogen bond with CYS919 in the ATP-binding site (hinge region), the oxygen of urea carbonyl group formed hydrogen bond with ASP1046, while the two NH of urea moiety showed bifurcate hydrogen bonds with GLU885. Additionally, the pyrrole ring showed hydrophobic interactions with CYS919, LEU840, ALA866, VAL848 and LEU1035, the pyrimidine ring formed hydrophobic interactions with CYS919, ALA868, VAL848 and LEU1035. Furthermore, the phenyl ring showed hydrophobic interactions with VAL848, VAL899, LYS868, CYS1045 and pi-pi interaction with PHE1047. Finally, the terminal phenyl ring formed hydrophobic interaction with LEU889. Docking of all synthesised derivatives was done on VEGFR-2 (PDB code: 3VHE), where the binding mode of representative thieno[2,3-*d*]pyrimidine derivatives was explored to justify the potent or significant VEGFR-2 inhibition exhibited by some of the synthesised compounds (Table 3 of Supplementary Material).

**Figure 9.**
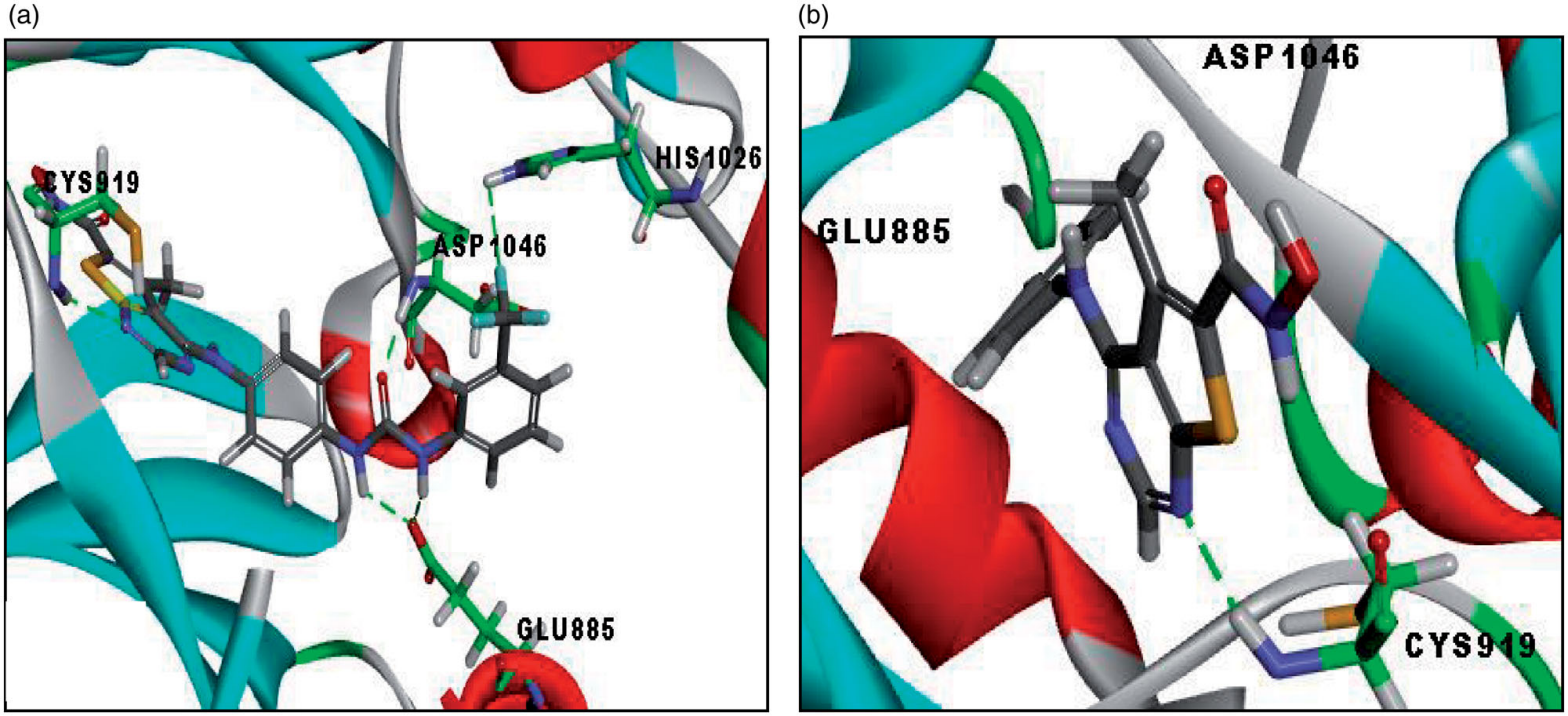
**(**a) 3D image of binding interactions of compound **(7c)** (-CDOCKER energy = 51.35) with VEGFR-2 (PDB code: 3VHE). (b) 3D image of binding interaction of compound **(15c)** (-CDOCKER energy =30.81) with VEGFR-2. Hydrogen bond interaction is described as green-dotted line.

In general, docking results revealed that most of the thieno[2,3-*d*]pyrimidine derivatives with urea moiety **(7c and 12c)** showed higher binding affinities than the reference compound and compounds with aniline moiety **(15c)** which is consistent with VEGFR-2 inhibition assay. In which, compounds bearing urea moiety, such as **(12c)** with (IC_50_ = 185 nM), and **(7c)** with (IC_50_ = 191 nM) exhibited potent VEGFR-2 inhibition compared to compound **(15c)** bearing aniline moiety with (IC_50_ = 5.58 µM). Where **(12c)** and **(7c)** retrieved all the essential hydrogen bond and hydrophobic interactions of the reference compound, while compound **(15c)** missed the essential hydrogen bond interactions with GLU885 and ASP1046 although its pyrimidine ring retrieved the essential hydrogen bond with CYS919 (Table 3 of Supplementary Material). The binding interactions and energies of two representatives docked compounds are presented in (Figure 9). Additionally, we found that compounds **(12c)** and **(7c)**; both bearing urea moiety with CF_3_ substituent and compound **(15c)** bearing 4-chloro aniline moiety showed higher binding affinities and made extra hydrophobic bonds than the reference compound. Where CF_3_ in compound **(12c)** made extra hydrophobic bonding with ILE892, LEU1019 and HIS1026, F of CF_3_ in compound **(7c)** made an extra halogen hydrogen bond with HIS1026 and 4-Cl of the terminal phenyl ring in compound **(15c)** forms additional hydrophobic interaction with LEU889, VAL899 and LYS868. According to previously reported SAR, this area is an allosteric hydrophobic binding pocket revealed for type II VEGFR inhibitors, in which the phenylalanine residue of the DFG loop flips out of its lipophilic pocket to increase the binding affinity of inhibitors to VEGFR receptors and increase residence time^37^^,^^38^.

##### 3.3.1.3. Docking study on HDAC6

**Figure 10.**
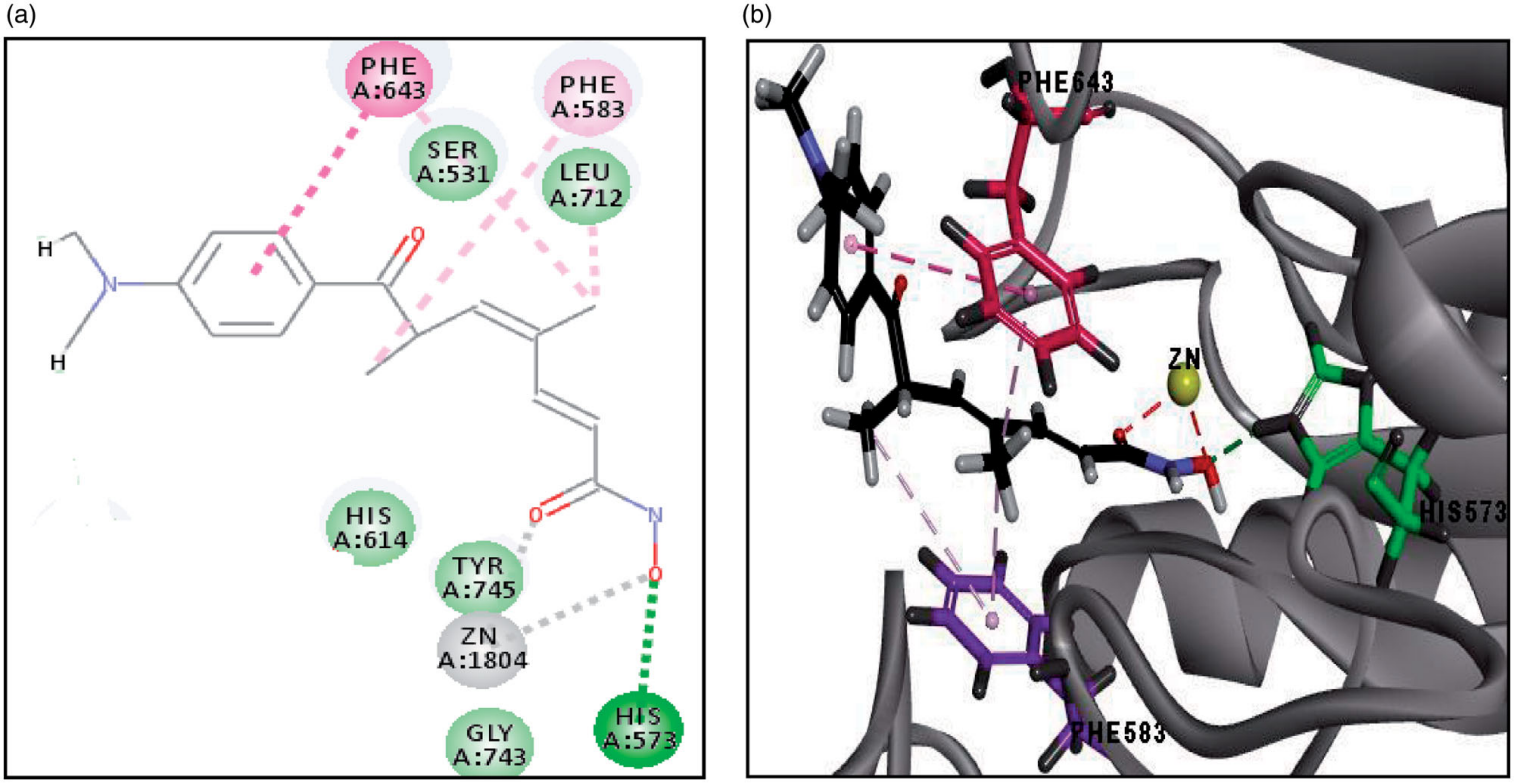
**(**a) The 2D image of binding interactions of Trichostatin A with HDAC6 (PDB code: 5G0H). (b) 3D image of binding interaction of redocked TSA with HDAC6 (-CDOCKER ENERGY= 25.54), where hydrogen bond interaction is described as green-dotted line, hydrophobic interactions are described as purple-dotted lines, pi-pi stacking is described as pink-dotted line and coordinate bond with zinc metal is described as grey-dotted line (2 D image) and red-dotted line (3 D image).

The final docking study was done on HDAC6 (PDB code: 5G0H) co-crystallised with the reference compound (Trichostatin A; TSA). RMSD is 0.44 Å, which signifies the validity of CDOCKER algorithm. Moreover, the reported binding mode^39^ of TSA was retrieved into the X-ray crystal structure of HDAC6 (-CDOCKER energy = 25.49) as shown in (Figure 10), where the oxygen of hydroxamic hydroxyl group forms a hydrogen bond with HIS573 and oxygen of both hydroxamic carbonyl and hydroxyl groups forms coordinate bonds with zinc metal in a bidentate fashion with distances 2.24 Å and 2.20 Å, respectively. Additionally, the phenyl ring of TSA shows pi-pi stacking with PHE643 and the aliphatic chain of the linker showed hydrophobic interaction with PHE583 and PHE643 (Figure 10). Docking of all synthesised compounds was performed on HDAC6 (PDB code: 5G0H), where the binding mode of representative thieno[2,3-*d*]pyrimidine derivatives were explored to justify the significant inhibition of compound **(20 b)** (56% inhibition) against HDAC6 among other derivatives that showed remarkably lower % inhibition against HDAC6 (Table 4 of Supplementary Material).

**Figure 11.**
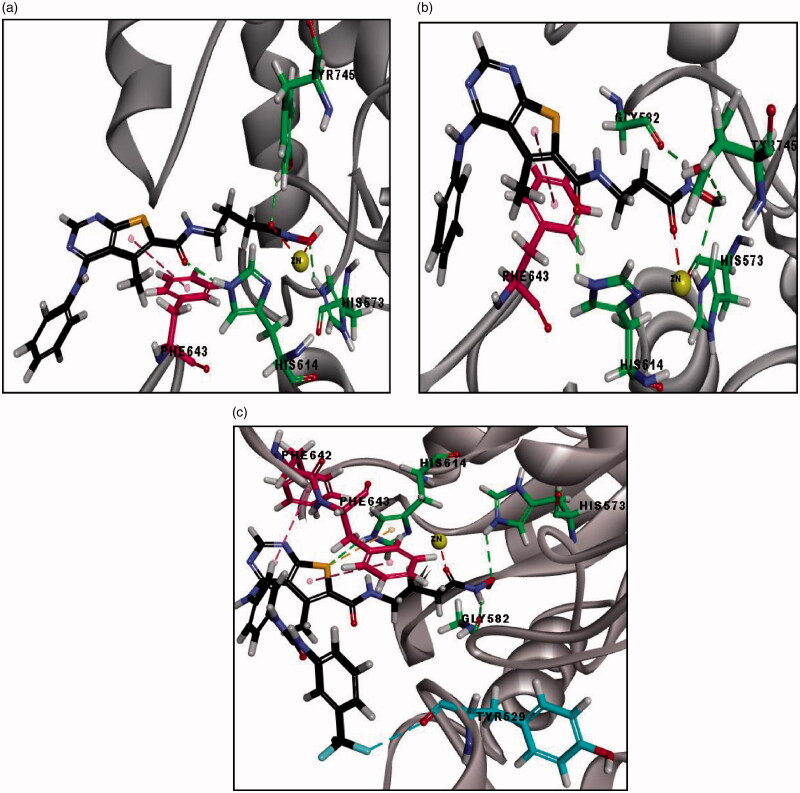
**(**a) 3D image of binding interactions of **(20 b)** (-CDOCKER ENERGY= 34.32) with HDAC6 (PDB code: 5G0H). (b) 3D image of binding interaction of **(19 b)** (-CDOCKER ENERGY= 37.68) with HDAC6. (c) 3D image of binding interaction of **(12c)** (-CDOCKER ENERGY= 37.58) with HDAC6. Hydrogen bond interaction is described as a green-dotted line, Pi-Pi interaction is described as a pink-dotted line, halogen interaction is described as cyan dotted lines and coordinate bond with zinc metal is described as a red dotted line.

The binding interactions and energies of three representatives docked compounds **(12c, 19 b and 20 b)** are presented in (Figure 11). Compound **(20 b)** with unsubstituted aniline moiety and three carbons in the linker region exhibited the most significant HDAC6 inhibition among all the synthesised compounds (56% inhibition) and it revealed higher binding affinity than TSA (-CDOCKER energy = 34.32). This significant inhibitory activity for **(20 b)** was justified by its consistent binding mode with the reported binding mode of TSA against HDAC6 (PDB: 5G0H), where the oxygen of hydroxamic carbonyl and hydroxyl groups forms coordinate bonds with zinc metal in a bidentate fashion with distances 2.24 Å and 2.36 Å, respectively. The oxygen of the hydroxamic hydroxyl group forms a hydrogen bond with HIS573 and oxygen of hydroxamic and amidic carbonyl group forms two additional hydrogen bonds with TYR745 and HIS614, respectively. Also, the thiophene ring of **(20 b)** shows pi-pi stacking with PHE643. While, compound **(19 b)** with unsubstituted aniline moiety and two carbons in the linker region exhibited insignificant inhibitory activity (10%), although it revealed higher binding affinity (-CDOCKER energy = 37.68) than **(20 b)** (Figure 11). By comparing the binding mode of compounds **(20 b)** and **(19 b),** it was found that the insignificant inhibitory activity of the compound **(19 b)** is certainly accounted for by having a shorter linker between the hydroxamate moiety and the connecting amide group (two carbons) than compound **(20 b)** (3 carbons) since they both bear the same hydrophobic cap group. The shorter two carbons linker of compound **(19 b)** contributes to its failure to form a coordinate bond with zinc metal in a bidentate fashion, where the oxygen of hydroxamic carbonyl group forms only one coordinate bond with zinc metal (Figure 11). Whereas compound **(12c)** with urea moiety exhibited lower HDAC6 inhibition (23%) than **(20 b)**, although it revealed higher binding affinity (-CDOCKER energy = 37.58) than **(20 b)** and TSA. The decrease in HDAC6 inhibition by compound **(12c)** is justified by its binding mode as described in (Figure 11), in which the oxygen of hydroxamic carbonyl group forms only one coordinate bond with zinc metal in a monodentate fashion, despite fulfilling all other key interactions with reported essential amino acids (Table 4 of Supplementary Material). Unexpectedly, compound **(15 b)** with unsubstituted aniline moiety, exhibited considerate HDAC6 inhibition (30%), although (i) it has no linear hydrocarbon linker between the thieno[2,3-*d*]pyrimidine fragment and its hydroxamate moiety. (ii) it has a low binding affinity to HDAC6 of (-CDOCKER energy = 15.78). (iii) it could not form a coordinate bond between the hydroxamate moiety and the zinc metal. (iv) it also missed the essential hydrogen bond with HIS573 as shown in (Table 4 of Supplementary Material). Finally, future directions towards optimisation of the identified lead inhibitor **(20 b)** involve: (i) hydrocarbon linker from five to seven carbons to enhance the potency of novel thieno[2,3-*d*]pyrimidine derivatives against HDACs, to be able to capture the zinc metal in bidentate fashion with shorter distances as TSA. (ii) Hydrophobic cap group of thieno[2,3-*d*]pyrimidine derivatives bearing aniline moiety instead of the larger thieno[2,3-*d*]pyrimidine derivatives with biphenyl urea or thiourea moiety, in which it has a better binding affinity and showed higher inhibition to HDAC6.

#### 3.3.2. *In silico* ADMET predictive study

A computer-aided ADMET study was performed using Accelrys Discovery Studio 4.5 software protocols to investigate pharmacokinetics properties of newly synthesised thieno[2,3-*d*] pyrimidine hydroxamic acid derivatives and their ester derivatives, such as absorption, distribution, metabolism, excretion and toxicity. This study was done to investigate the unexpected moderate *in vitro* antiproliferative activity of most of the synthesised hydroxamic acid compounds despite their potent *in vitro* EGFR and VEGFR-2 enzyme inhibition. Aqueous solubility, blood brain barrier (BBB) penetration, CYP2D6 binding, human intestinal absorption (HIA), toxicity and plasma protein binding descriptors are predicted. 2 D ADMET plot for thieno[2,3-*d*] pyrimidine hydroxamic acid derivatives is drawn between ADMET_PSA_2D versus ADMET_AlogP98 (Figure 1 of Supplementary Material). Two analogous 95% and 99% confidence ellipses are shown in the biplot corresponding to HIA and BBB models^40^. The results of the calculated representative parameters for thieno[2,3-*d*] pyrimidine hydroxamic acid derivatives are tabulated in (Table 5 of Supplementary Material). There is an inverse relationship between polar surface area (PSA) with percent HIA and thus cell membrane permeability^41^. ALogP is used to indicate the lipophilicity of the investigated compounds, thus calculating ALogP with PSA is used to bring information about hydrogen bond characteristics^42^. Optimum cell membrane permeability is predicted if the compound has (PSA < 140˚A2 and AlogP98 < 5)^42^. In this study, all hydroxamic acid compounds showed ALogP98 < 5, 14 compounds showed high accepted values of PSA: 140˚A2 < PSA > 100˚A2, indicating considerable cell membrane permeability but not highly permeable due to its high value of PSA (>100˚A2), which is inversely proportional with cell membrane permeability. Whereas, the rest of the compounds bearing urea moiety and with linker between the hydroxamic acid moiety and the amide connecting unit **(11a-c, 12a-c)** showed PSA > 140, which indicated poor cell membrane permeability and absorption.

By comparing the AlogP98 and the PSA of the synthesised thieno[2,3-*d*] pyrimidine hydroxamic acids and their esters (Table 6 of Supplementary Material), it was found that almost all of the prepared thieno[2,3-*d*]pyrimidine esters showed lower PSA (PSA < 140) and higher AlogP than most of the synthesised target thieno[2,3-*d*]pyrimidine hydroxamic acids. These results coincide with the unsatisfactory poor to moderate *in vitro* antiproliferative activities of the hydroxamic acid derivatives which could be attributed to their poor cell membrane permeability. Also, it was found that the synthesised hydroxamic acid compounds bearing aniline moiety were predicted to show better intestinal absorption and cell membrane permeability than compounds bearing urea moieties. While compounds **(7a-d)** bearing urea moiety with no linear hydrocarbon linker between the hydroxamic acid moiety and the thieno[2,3-*d*]pyrimidine fragment were predicted to have acceptable cell membrane permeability as an exception.

## 4. Conclusion

In the current study, series of hybrids of thieno[2,3-*d*]pyrimidine fragment bearing arylamino and diaryl urea moieties at 4-position linked to hydroxamic acid moiety were designed and synthesised as inhibitors for EGFR/VEGFR-2/HDAC6. Compounds **(12c, 15 b and 20 b)** may be considered as potential multitarget inhibitors against EGFR/VEGFR-2/HDAC6. Additionally, other synthesised novel hydroxamic acid compounds may be considered as EGFR/VEGFR-2 dual inhibitors, such as **(7a, 15c and 15d),** in which the hydroxamic acid moiety is directly attached to the thieno[2,3-*d*]pyrimidine fragment, contributing to maximum inhibitory dual activity. Compound **(15c)** could be the most potent dual EGFR/VEGFR-2 inhibitor in this study. Moreover, promising selective VEGFR-2 **(7c and 11c)** and EGFR **(20d)** inhibitors were identified. The results of the enzyme inhibitory assays were found to coincide with the molecular docking studies performed on the three enzymes EGFR, VEGFR-2 and HDAC6.

These results supported the rationale of the design strategy, where the thieno[2,3-*d*]pyrimidine fragment bearing urea moiety or aniline moiety may be considered as a promising capping group. Unexpectedly, it is observed that most of the compounds showed common inhibition (weak to moderate inhibition) towards Renal UO-31 and CNS cancer SNB-75 cell lines (20–29% inhibition) although they showed potent enzyme inhibitory profiles. Results of the ADMET study revealed poor cell membrane permeability, explaining their unsatisfactory *in vitro* antiproliferative activities. Future structural modifications will be studied by implementing specific structural modifications to optimise the hydrophobicity of the designed compounds for enhancing their cell membrane permeability.

## 5. Experimental protocols

### 5.1. Chemistry

Reagents and starting materials were obtained from Alfa-Aeser organics and Sigma Aldrich without further purification. Melting points were uncorrected and recorded using BUCHI B-540 apparatus. Thin layer chromatography (TLC) is performed on 0.255 mm silica gel plates and is purchased from Merck to monitor reactions under U.V. light (254 nm). Mass spectrum was recorded in Thermo Scientific GCMS model on Direct Inlet part to mass analyser ISQ at the Regional Centre for Mycology and Biotechnology, Al-Azhar University. ^1^H and ^13 ^C NMR spectra were recorded on a Bruker Avance III HD FT-high resolution- NMR 400 MHz and scaled as δ in ppm at the Centre for Drug Discovery Research and Development, Faculty of Pharmacy, Ain Shams University. Description of coupling patterns is as follows: s, singlet; br s, singlet broad; d, doublet; t, triplet; m, multiplet; and 1H, 2H, 3H, etc. The coupling constants are described as *J*, which was rounded off to one decimal place. IR spectra were done at the Micro analytical centre, Cairo University, and recorded on a 4100 Jasco spectrophotometer. Elemental analysis was performed at the Regional Centre for Mycology and Biotechnology, AL Azhar University.

#### 5.1.1. Diethyl (5-amino-3-methylthiophene)-2,4-dicarboxylate (1)

A solution of ethyl cyanoacetate (0.01 mol), ethyl acetoacetate (0.01 mol), sulphur (0.01 mol) and morpholine (0.01 mol) was stirred and heated (70 °C) in absolute ethanol (30 ml) for 4 h. The mixture was then left for 24 h at 0 °C. The formed solid was filtered, rinsed with ethanol (20 ml), dried and then crystallised from absolute ethanol to yield the titled compound as yellow crystals **(1)** (1.85 g, 72%), m.p. 107–109 °C (as reported)^21^^,^^43^.

#### 5.1.2. Ethyl (5-methyl-4-oxo-3,4-dihydrothieno[2,3-d]pyrimidine)-6-carboxylate (2)

A solution of **(1)** (2 g, 7.8 mmol) in formamide (16 ml) and acetic acid (0.5 ml) was heated under reflux at 150 °C for 40 h, cooled to 70 °C and then 90 ml water was added. The suspension was then cooled to room temperature with stirring. The formed solid was collected by filtration, washed with water (2 × 10 ml) and dried to give the titled compound as pale yellow solid **(2)** (1.8 g, 90%), m.p. 246–248 °C (as reported)^44^^,^^45^.

#### 5.1.3. Ethyl (4-chloro-5-methylthieno[2,3-d]pyrimidine)-6-carboxylate (3)

A mixture of compound **(2)** (3.5 g, 14.7 mmol; 1 equiv) and phosphorus oxychloride (29 ml, 278 mmol; 18.9 equiv.) was refluxed at 90 °C for 4 h. The mixture was then slowly poured on iced cold water with continuous stirring. The formed solid was filtered off immediately and dried thoroughly over anhydrous sodium sulphate to afford the titled compound **(3)** (3.46 g, 92%) as light brown crystals, m.p. 114–115 °C (as reported)^22^.

#### 5.1.4. General procedure for preparation of compounds (4a-d)

The appropriate isocyanate (7 mmol;1 equiv.) was added to a solution of *p*-nitroaniline (7 mmol;1 equiv.) in dry methylene chloride (20 ml) and stirred at room temperature for 24 h. The formed solid was filtered, stirred again with dry methylene chloride then collected by filtration and dried. Crystallisation from ethanol yielded the titled compounds **(4a-d)**^23^.

##### 5.1.4.1. 1–(4-nitrophenyl)-3-phenylurea (4a)

Yield 55% as yellowish white crystals, m.p. 226–228 °C (as reported)^46^.

##### 5.1.4.2. 1–(3-methoxyphenyl)-3–(4-nitrophenyl)urea (4 b)

Yield 50% as yellow crystals, m.p. 210–212 °C (as reported)^46^.

##### 5.1.4.3. 1–(4-nitrophenyl)-3–(3-(trifluoromethyl)phenyl)urea (4c)

Yield 69% as greenish yellow crystals, m.p. 258–260 °C (as reported)^37^.

##### 5.1.4.4. 1–(4-nitrophenyl)-3-phenylthiourea (4d)

Yield 59% as yellow crystals, m.p. 147–149 °C (as reported)^47^.

#### 5.1.5. General procedure for preparation of compounds (5a-d)

A mixture of the appropriate nitro phenyl urea or thiourea derivatives **(4a-d)** (4 mmol) and Pd-C (0.1 g, 10%) in methanol (100 ml) was stirred under H_2_ at 60 bars, at room temperature for 6 h. Then Pd-C was completely removed through filtration over celite. The filtrate was evaporated under vacuum and dried to afford titled compounds **(5a-d)** which were then recrystallized from its suitable solvent^24^.

##### 5.1.5.1. 1–(4-aminophenyl)-3-phenylurea (5a)

Yield 85%, crystallised from ethyl acetate/hexane as white crystals, m.p. 297–300 °C (as reported)^48^.

##### 5.1.5.2. 1–(3-methoxyphenyl)-3–(4-aminophenyl)urea (5 b)

Yield 70%, crystallised from ethanol as white crystals, m.p. 164–168 °C (as reported)^38^.

##### 5.1.5.3. 1–(4-aminophenyl)-3–(3-(trifluoromethyl)phenyl)urea (5c)

Yield 80%, crystalised from ethanol as greyish white crystals, m.p. 148–150 °C (as reported)^37^.

##### 5.1.5.4. 1–(4-aminophenyl)-3-phenylthiourea (5d)

Crystallised from ethanol as dark purple liquid, b.p. 410 °C (as reported)^30^.

#### 5.1.6. General procedure for preparation of 5-aminobenzimidazole (13a)

A mixture of 5-nitro benzimidazole (1 g, 30.65 mmol) and 10% Pd/C (0.1 g) in methanol (40 ml) was stirred under H_2_ at 60 bars, at room temperature for 6 h. The Pd/C was removed completely through filtration over celite, then the filtrate was evaporated under vacuum and dried to afford brown crystals of **(13a)** (0.84 g, 85%), m.p. 165–166 °C (as reported)^49^.

#### 5.1.7. General procedure for preparation of compounds (6a-d) and (14a-d)

To a solution of the ethyl (4-chloro-5-methylthieno[2,3-*d*]pyrimidine)-6-carboxylate **(3)** (0.256 g, 1 mmol; 1 equiv.) in ethanol (15 ml), the appropriate amino phenyl urea and thiourea derivatives **(5a-d)/**5 amino benzimidazole derivative **(13a)/**appropriate aniline derivatives **(13 b-d)** (1 mmol; 1 equiv.) and triethylamine TEA (0.3 ml, 2 mmol; 2equiv.) were added. The mixture was heated for 24–48 h under reflux. The formed solid was filtered, washed with hot ethanol, dried, and crystallised from appropriate solvent to afford the titled compounds **(6a-d)** and **(14a-d)**^25^.

##### 5.1.7.1. Ethyl 5-methyl-4–(4-(3-phenylureido)phenylamino)thieno[2,3-d]pyrimidine-6-carboxylate (6a)

Yield 88% as white crystals, crystalised from acetone, m.p. 319–322 °C (as reported)^50^.

##### 5.1.7.2. Ethyl 4–(4-(3–(3-methoxyphenyl)ureido)phenylamino)-5-methylthieno[2,3-d]pyrimidine-6-carboxylate (6 b)

Yield 84% as buff crystals, crystallised from acetone, m.p. 220–222 °C (as reported)^50^.

##### 5.1.7.3. Ethyl 4–(4-(3–(3-trifluromethyphenyl)ureido)phenylamino)-5-methylthieno[2,3-d]pyrimidine-6-carboxylate (6c)

Yield 60% as greyish white crystals, crystallised from ethanol, m.p. 305–307 °C. **^1^HNMR (400 MHz, DMSO-d6) δ:** 1.32–1.36 (*t*, 2H, CH_2_CH_3_, *J* = 8 Hz), 3.07 (*s*, 3H, CH_3_), 4.32–4.38 (*q*, 2H, CH_2_CH_3_, *J* = 8 Hz), 7.31–7.33 (d, 1H, aromatic H_6_″, *J* = 8 Hz), 7.49–7.51 (d, 2H, aromatic H_4_″, H_5_″, *J* = 8 Hz), 7.54–7.56 (d, 2H, aromatic H_2_′, H_6_′, *J* = 8 Hz), 7.58–7.60 (d, 2H, aromatic H_3_′, H_5_′, *J* = 8 Hz), 8.04 (*s*, 1H, aromatic H_2_″), 8.46 (*s*, 1H, pyrimidine H), 8.60, 8.88, 9.10 (*s* × 3, 3H, NH × 3, exchangeable by D_2_O).

##### 5.1.7.4. Ethyl 5-methyl-4–(4-(3-phenylthioureido)phenylamino)thieno[2,3-d]pyrimidine-6-carboxylate *(6d).*

Yield 71% as grey solid, crystallised from ethanol, m.p. 173–175 °C. **^1^HNMR (400 MHz, DMSO-d6) δ:** 1.31–1.35 (*t*, 2H, CH_2_CH_3_, *J* = 8 Hz), 3.02 (*s*, 3H, CH_3_), 4.30–4.36 (*q*, 2H, CH_2_CH_3_, *J* = 8 Hz), 5.31 (br *s*, 2H, NH ×2, exchangeable by D_2_O), 6.59–6.61 (d, 2H, aromatic H_2_′, H_6_′, *J* = 8 Hz), 7.20–7.22 (d, 2H, aromatic H_3_′, H_5_′, *J* = 8 Hz), 7.49–7.71 (*m*, 5H, aromatic H_2_″- H_6_″), 8.36 (*s*, 1H, pyrimidine H), 8.38 (*s*,1H, NH, exchangeable by D_2_O).

##### 5.1.7.5. Ethyl 4-(1H-benzo[d]imidazol-5-ylamino)-5-methylthieno[2,3-d]pyrimidine-6-carboxylate. (14a)

Yield 77% as faint brown crystals, crystallised from acetone, m.p 250–251 °C (as reported)^50^.

##### 5.1.7.6. Ethyl 5-methyl-4-(phenylamino)thieno[2,3-d]pyrimidine-6-carboxylate (14 b)

Yield 89% as white crystals, crystallised from ethanol, m.p 170–172 °C (as reported)^51^.

##### 5.1.7.7. Ethyl 4–(4-chlorophenylamino)-5-methylthieno[2,3-d]pyrimidine-6-carboxylate (14c)

Yield 82% as yellow crystals, crystallised from ethanol, m.p 174–176 °C (as reported)^52^.

##### 5.1.7.8. Ethyl 4–(3-chloro-4-fluorophenylamino)-5-methylthieno[2,3-d]pyrimidine-6-carboxylate (14d)

Yield 97% as faint yellow crystals, crystallised from ethanol, m.p 154–156 °C (as reported)^52^.

#### 5.1.8. General procedure for preparation of compounds (7a-d) and (15a-d)

A cooled solution of sodium (27 mmol; 10 equiv.) in absolute ethanol (20 ml) was added to a solution of hydroxylamine hydrochloride (54 mmol; 20 equiv.) in absolute ethanol cooled in an ice bath. The mixture was stirred for 15 min. The formed precipitate was filtered, and the free hydroxylamine solution was prepared. The freshly prepared hydroxylamine solution was placed in a rounded bottom flask and cooled in an ice bath. Compounds **(6a-d)** and **(14a-d)** (2.7 mmol; 1 equiv.) were added to the solution and the mixture was stirred overnight. The reaction is monitored by TLC till the complete disappearance of starting material using an appropriate eluting system [DCM: MeOH (9.5:0.5)]. The solvent was removed under vacuum to afford crude products, washed with water and crystalised from ethanol to afford target compounds **(7a-d)** and **(15a-d)**^26^^,^^27^.

##### 5.1.8.1. 1–(4-(6-(hydroxycarbamoyl)-5-methylthieno[2,3-d]pyrimidin-4-ylamino)phenyl)-3-phenylurea (7a)

Yield 80% as yellowish orange solid, m.*p* > 200 °C. **^1^HNMR (400 MHz, DMSO-d6) δ:** 3.16 (*s*, 3H, CH_3_), 6.93–6.96 (*t*, 1H, aromatic H_4_″, *J* = 8 Hz), 7.26–7.30 (*t*, 2H, aromatic H_2_′, H_6_′, *J* = 8 Hz), 7.56–7.62 (*m*, 6H, aromatic H_3_′, H_5_′, H_2_″, H_3_″, H_5_″, H_6_″), 8.23 (*s*, 1H, NH, exchangeable by D_2_O), 8.38 (*s*, 1H, pyrimidine H), 9.81, 9.95 (*s* × 2, 2H, NH × 2, exchangeable by D_2_O). **MS: *m*/*z* (%):** 435 (M^+^+1, 22%), 434 (M^+^, 40%), 130 (100%). **Anal. Calcd. For C_21_H_18_N_6_O_3_S:** C 58.05, H 4.18, N 19.35, S 7.38; Found: C 58.21, H 4.35, N 19.50, S 7.46.

##### 5.1.8.2. 1–(4-(6-(hydroxycarbamoyl)-5-methylthieno[2,3-d]pyrimidin-4-ylamino)phenyl)-3–(3-methoxyphenyl)urea (7 b)

Yield 75% as yellow solid, m.*p* > 200 °C. **^1^HNMR (400 MHz, DMSO-d6) δ:** 3.13 (*s*, 3H, CH_3_), 3.75 (*s*, 3H, OCH_3_), 6.52–6.54 (d, 1H, aromatic H_4_″, *J* = 8 Hz), 7.01–7.19 (*m*, 3H, aromatic H_2_′, H_6_′, H_5_″), 7.32 (*s*, 1H, aromatic H_2_″), 7.49–7.60 (*m*, 3H, aromatic H_3_′, H_5_′, H_6_′), 8.21 (*s*, 1H, pyrimidine H), 8.27, 8.36, 9.74, 9.79 (*s* × 4, 4H, NH × 4, exchangeable by D_2_O), 11.13 (*s*, OH, exchangeable by D_2_O). **^13 ^C NMR (DMSO-d6, 400 MHz) δ:** 14.92, 55.26, 104.21, 107.99, 112.50, 118.67, 121.01, 123.72, 133.72, 136.56, 144.23, 146.22, 149.27, 153.38, 156.91, 159.96, 162.90, 164.46, 168.44. **MS: m/z (%):** 466 (M^+^+2, 26%), 464 (M^+^, 30%), 132 (M^+^, 100%). **Anal. Calcd. For C_22_H_20_N_6_O_4_S:** C 56.89, H 4.34, N 18.09, S 6.90; Found: C 57.15, H 4.29, N 18.30, S 6.83. **FT-IR (**ú **max, cm^−1^)**: 3583 (NH stretch), 3441, 3417 (OH/NH broad), 3011 (CH aromatic), 2835–2769 (CH aliphatic), 1728 (C = O), 1635 (C = O amide), 1612 (NH bend).

##### 5.1.8.3. 1–(4-(6-(hydroxycarbamoyl)-5-methylthieno[2,3-d]pyrimidin-4-ylamino)phenyl)-3–(3-(trifluoromethyl)phenyl)urea (7c)

Yield 69% as yellow solid, m.p 285 °C. **^1^HNMR (400 MHz, DMSO-d6) δ:** 3.07 (*s*, 3H, CH_3_), 3.16 (*s*, 1H, OH, exchangeable by D_2_O), 7.29–7.31 (d, 1H, aromatic H_6_″, *J* = 8 Hz), 7.50–7.55 (*m*, 2H, aromatic H_4_″, H_5_″), 7.59–7.61 (d, 2H, aromatic H_2_′, H_6_′, *J* = 8 Hz), 7.66–7.68 (d, 2H, aromatic H_3_′, H_5_′, *J* = 8 Hz), 8.09 (*s*, 1H, aromatic H_2_″), 8.30 (*s*, 1H, pyrimidine H), 8.39, 8.47, 8.61, 9.59 (*s* × 4, 4H, NH × 4, exchangeable by D_2_O). **MS: *m*/*z* (%):** 502 (M^+^, 24%), 286 (94%), 211 (100%). **Anal. Calcd. For C_22_H_17_F_3_N_6_O_3_S:** C 52.59, H 3.41, N 16.73, S 6.38; Found: C 52.57, H 3.40, N 16.71, S 6.38.

##### 5.1.8.4. 1–(4-(6-(hydroxycarbamoyl)-5-methylthieno[2,3-d]pyrimidin-4-ylamino)phenyl)-3-phenylthiourea (7d)

Yield 65% as brown solid, m.p 300 °C. **^1^HNMR (400 MHz, DMSO-d6) δ:** 1.24 (*s*, 1H, OH, exchangeable by D_2_O), 3.04 (*s*, 3H, CH_3_), 4.94 (*s*, 1H, NH, exchangeable by D_2_O), 6.56–6.58 (d, 2H, aromatic H_2_′, H_6_′, *J* = 8 Hz), 7.22–7.24 (d, 2H, aromatic H_3_′, H_5_′, *J* = 8 Hz), 7.55–7.99 (*m*, 5H, aromatic H_2_″- H_6_″), 8.23 (*s*, 1H, pyrimidine H), 8.54 (s, 1H, NH, exchangeable by D_2_O). **MS: *m*/*z* (%):** 450 (M^+^, 28%), 448 (M^+^-2, 13%), 240 (100%). **Anal. Calcd. For C_21_H_18_N_6_O_2_S_2_:** C 55.98, H 4.03, N 18.65, S 14.23; Found: C 56.11, H 4.28, N 18.71, S 14.09. **FT-IR (**ú **max, cm^ − 1^)**: 3433 (OH/NH broad), 3016 (CH aromatic), 2958–2854 (CH aliphatic), 1732 (C = O), 1658 (C = O amide), 1612 (NH bend).

##### 5.1.8.5. 4-(1H-benzo[d]imidazol-4-ylamino)-N-hydroxy-5-methylthieno[2,3-d]pyrimidine-6-carboxamide (15a)

Yield 89% as orange brown solid, m.p 280 °C. **^1^HNMR (400 MHz, DMSO-d6) δ:** 2.94 (*s*, 3H, CH_3_), 5.42 (*s*, 1H, NH, exchangeable by D_2_O), 7.37–7.39 (d, 1H, aromatic H_5_′_,_ *J* = 8 Hz_)_ , 7.58–7.60 (d, 1H, aromatic H_4_′, *J* = 8 Hz), 7.98 (*s*, 1H, aromatic H_7_′), 8.21 (*s*, 1H, aromatic H_2_′), 8.41 (*s*, 1H, pyrimidine H), 8.53, 8.58 (*s*, 2H, NH × 2, exchangeable by D_2_O). **MS: *m*/*z* (%):** 340 (M^+^, 65%), 341 (M^+^+1, 11%), 102 (100%). **Anal. Calcd. For C_15_H_12_N_6_O_2_S:** C 52.93, H 3.55, N 24.69, S 9.42; Found: C 53.17, H 3.64, N 24.58, S 9.53. **FT-IR (**ú **max, ^c^m − 1)**: 3441 (OH broad), 3417, 3398 (2 × NH stretch), 3170–3113 (CH aromatic), 2958–2854 (CH aliphatic), 1662 (C=O), 1573 (NH bend).

##### 5.1.8.6. N-hydroxy-5-methyl-4-(phenylamino)thieno[2,3-d]pyrimidine-6-carboxamide (15b)

Yield 90% as greyish brown, m.p 200 °C. **^1^HNMR (400 MHz, DMSO-d6) δ:** 2.92 (*s*, 1H, OH, exchangeable by D_2_O), 3.08 (*s*, 3H, CH_3_), 7.08–7.14 (*m*, 1H, aromatic H_4_′), 7.34–7.38 (*t*, 2H, aromatic H_2_′, H_6_′, *J* = 8 Hz), 7.66–7.70 (*t*, 2H, aromatic H_3_′, H_5_′, *J* = 8 Hz), 8.31, 8.39 (*s* × 2, 2H, NH × 2, exchangeable by D_2_O), 8.43 (*s*, 1H, pyrimidine H). **MS: *m*/*z* (%):** 300 (M^+^, 29%), 301 (M^+^+1, 19%), 195 (100%). **Anal. Calcd. For C_14_H_12_N_4_O_2_S:** C 55.99, H 4.03, N 18.65, S 10.68; Found: C 56.16, H 4.29, N 18.78, S 10.81. **FT-IR (**ú **max, cm^ − 1^)**: 3452 (NH stretch), 3394 (OH/NH broad), 3010 (CH aromatic), 2978–2862 (CH aliphatic), 1600 (C=O), 1566 (NH bend).

##### 5.1.8.7. 4–(4-chlorophenylamino)-N-hydroxy-5-methylthieno[2,3-d]pyrimidine-6-carboxamide (15c)

Yield 95% as yellow solid, m.p 349 °C. **^1^HNMR (400 MHz, DMSO-d6) δ**: 3.04 (*s*, 3H, CH_3_), 7.40–7.42 (d, 2H, aromatic H_2_′, H_6_′, *J* = 8 Hz), 7.73–7.75 (d, 1H, aromatic H_3_′, H_5_′, *J* = 8 Hz), 8.37 (s, 1H, NH, exchangeable by D_2_O), 8.40 (s, 1H, pyrimidine H). **MS: *m*/*z* (%):** 335 (M^+^+1, 5%), 334 (M^+^, 11%), 274 (100%). **Anal. Calcd. For C_14_H_11_ClN_4_O_2_S:** C 50.23, H 3.31, N 16.74, S 9.58; Found: C 50.18, H 3.28, N 16.73, S 9.55.

##### 5.1.8.8. 4–(3-chloro-4-fluorophenylamino)-N-hydroxy-5-methylthieno[2,3-d]pyrimidine-6-carboxamide (15d)

Yield 91% as buff solid, m.p 320 °C. **^1^HNMR (400 MHz, DMSO-d6) δ:** 1.72 (*s*, 1H, OH, exchangeable by D_2_O), 3.07 (*s*, 3H, CH_3_), 7.39–7.44 (*t*, 1H, aromatic H_5_′, *J* = 8 Hz), 7.65–7.69 (*m*, 1H, aromatic H_2_′), 7.96–7.99 (dd, 1H, aromatic H_6_′, *J* = 4, 12 Hz) , 8.41 (*s*, NH, exchangeable by D_2_O), 8.42 (*s*, 1H, pyrimidine H), 8.50 (*s*, 1H, NH, exchangeable by D_2_O). **MS: *m*/*z* (%):** 354 (M^+^+2, 20%), 353 (M^+^+1, 10%), 352 (M^+^, 100%). **Anal. Calcd. For C_14_H_10_FClN_4_O_2_S:** C 47.67, H 2.86, N 15.88, S 9.09; Found: C 47.80, H 3.12, N 16.15, S 9.23. **FT-IR (**ú **max, cm^−1^)**: 3441 (OH/NH broad), 2985 (CH aromatic), 2916–2769 (CH aliphatic), 1612 (C = O), 1573 (NH bend).

#### 5.1.9. General procedure for preparation of compounds (8a-c, 16b-d)

An aqueous solution of lithium hydroxide (30 mmol) in water (50 ml) was added to a solution of compounds **(6a-c, 14b-d)** (10 mmol) in THF (30 ml) and ethanol (9 ml). The mixture is stirred for 7 h at 50–60 °C. Complete hydrolysis is monitored by TLC, followed by the addition of ethyl acetate (50 ml), 1 N HCl (10 ml) and water (20 ml). The aqueous layer was separated from the organic layer and washed with ethyl acetate (2 × 50 ml). Then, the combined organic solutions were dried over anhydrous sodium sulphate (20 g), filtered and removed under vacuum to afford titled compounds **(8a-c, 16 b-d)**^28^.

##### 5.1.9.1. 5-methyl-4–(4-(3-phenylureido)phenylamino)thieno[2,3-d]pyrimidine-6-carboxylic acid (8a)

Yield 90% as white crystals, m.p 281–283 °C (as reported)^50^.

##### 5.1.9.2. 4–(4-(3–(3-methoxyphenyl)ureido)phenylamino)-5-methylthieno[2,3-d]pyrimidine-6-carboxylic acid (8 b)

Yield 85% as white crystals, m.p 260–263 °C (as reported)^50^.

##### 5.1.9.3. 5-methyl-4–(4-(3–(3-(trifluoromethyl)phenyl)ureido)phenylamino)thieno[2,3-d]pyrimidine-6-carboxylic acid (8c)

Yield 74% as yellow crystals, m.p. 223–225 °C. **^1^HNMR (400 MHz, DMSO-d6) δ:** 3.06 (*s*, 3H, CH_3_), 7.29–7.31 (d, 1H, aromatic H_6_″, *J* = 8 Hz), 7.49–7.51 (d, 2H, aromatic H_4_″, H_5_″, *J* = 8 Hz), 7.54–7.56 (d, 2H, aromatic H_2_′, H_6_′, *J* = 8 Hz), 7.58–7.60 (d, 2H, aromatic H_3_′, H_5_′, *J* = 8 Hz), 8.03 (*s*, 1H, aromatic H_2_″), 8.46 (*s*, 1H, pyrimidine H), 8.63, 9.05, 9.31 (*s* × 3, 3H, NH × 3, exchangeable by D_2_O).

##### 5.1.9.4. 5-methyl-4-(phenylamino)thieno[2,3-d]pyrimidine-6-carboxylic acid (16b) [53]

Yield 99% as buff crystals, m.p. 178–180 °C.

##### 5.1.9.5. 4–(4-chlorophenylamino)-5-methylthieno[2,3-d]pyrimidine-6-carboxylic acid (16c)

Yield 89% as off white crystals, m.p 316–318 °C (as reported)^52^.

##### 5.1.9.6. 4–(3-chloro-4-fluorophenylamino)-5-methylthieno[2,3-d]pyrimidine-6-carboxylic acid (16d)

Yield 84% as white crystals, m.p 305–307 °C (as reported)^52^.

#### 5.1.10. General procedure for preparation of compounds (9a-c, 17b-d)

To a solution of beta-alanine ethyl ester hydrochloride (8.94 mmol), thieno[2,3-d]pyrimidine-6-carboxylic acid derivatives **(8a-c, 16b-d)** (8.94 mmol) and 1‐hydroxybenzotriazole (1.32 g, 9.18 mmol) in dry dimethylformamide (20 ml), N‐methyl morpholine (4.51 g, 44.65 mmol) was added at 0 °C and stirred for 30 min. Then EDCI.HCl (1.8 g, 13.12 mmol) was added and stirred for another 30 min maintaining the same temperature. The reaction mixture was stirred for 8 h at room temperature and then followed by the addition of water (20 ml) and ethyl acetate (2 × 20 ml) for extraction. Organic extracts were combined, washed with water (2 × 10 ml) and aqueous sodium bicarbonate solution (2 × 10 ml). Then organic solution was dried with anhydrous Na_2_SO_4_, and evaporated under vacuum to afford the titled compounds **(9a-c, 17 b-d)**^29^.

##### 5.1.10.1. Ethyl 3–(5-methyl-4–(4-(3-phenylureido)phenylamino)thieno[2,3-d]pyrimidine-6-carboxamido)propanoate (9a)

Yield 84% as white solid, m.p 229 °C. **^1^HNMR (400 MHz, DMSO-d6) δ:** 1.20–1.23 (*t*, 3H, OCH_2_CH_3_, *J* = 8 Hz), 2.59–2.62 (*t*, 2H, CH_2_CO, *J* = 8 Hz), 2.86 (*s*, 3H, CH_3_), 3.48–3.53 (*q*, 2H, CH_2_NH, *J* = 8 Hz) , 4.07–4.13 (*q*, 2H, OCH_2_CH_3_, *J* = 8 Hz), 6.96–7.00 (*t*, 1H, aromatic H_4_″, *J* = 8 Hz), 7.27–7.31 (*t*, 2H, aromatic H_2_′, H_6_′, *J* = 8 Hz), 7.45–7.48 (d, 4H, aromatic H_2_″, H_3_″, H_5_″, H_6_″, *J* = 12 Hz) , 7.52–7.54 (d, 2H, aromatic H_3_′_,_ H_5_′, *J* = 8 Hz), 8.41 (*s*, 1H, pyrimidine H), 8.44, 8.50, 8.66, 8.69 (*s* × 4, 4H, NH × 4,exchangeable by D_2_O). **MS: *m*/*z* (%):** 518 (M^+^, 36%), 515 (M^+^-3, 2%), 92 (100%). **Anal. Calcd. For C_26_H_26_N_6_O_4_S:** C 60.22, H 5.05, N 16.21, S 6.18; Found: C 60.08, H 5.17, N 16.48, S 6.09.

##### 5.1.10.2. Ethyl 3–(4-(4–(3-(3-methoxyphenyl)ureido)phenylamino)-5-methylthieno[2,3-d]pyrimidine-6-carboxamido)propanoate (9 b)

Yield 89% as buff solid, m.p 250 °C. **^1^HNMR (400 MHz, DMSO-d6) δ:** 1.20–1.23 (*t*, 3H, OCH_2_CH_3_, *J* = 8 Hz), 2.59–2.62 (*t*, 2H, CH_2_CO, *J* = 8 Hz), 2.86 (*s*, 3H, CH_3_), 3.48–3.53 (*q*, 2H, CH_2_NH, *J* = 8 Hz), 3.74 (*s*, 3H, OCH_3_), 4.07–4.13 (*q*, 2H, OCH_2_CH_3_, *J* = 8 Hz), 6.55–6.57 (d, 1H, aromatic H_4_″, *J* = 8 Hz), 6.93–6.95 (d, 1H, aromatic H_5_″, *J* = 8 Hz), 7.16–7.20 (*t*, 2H, aromatic H_2_″, H_6_″, *J* = 8 Hz) 7.45–7.47 (d, 2H, aromatic H_2_′, H_6_′, *J* = 8 Hz), 7.52–7.54 (d, 2H, aromatic H_3_′, H_5_′, *J* = 8 Hz), 8.41 (*s*, 1H, pyrimidine H), 8.44, 8.50, 8.71, 10.08 (*s* × 4, 4H, NH × 4, exchangeable by D_2_O). **MS: *m*/*z* (%):** 546 (M^+^, 66%), 548 (M^+^+2, 67%), 501 (100**%). Anal. Calcd. For C_27_H_28_N_6_O_5_S:** C 59.11, H 5.14, N 15.32, S 5.84; Found: C 59.37, H 5.31, N 15.49, S 6.02.

##### 5.1.10.3. Ethyl 3–(5-methyl-4–(4-(3(3(trifluoromethyl)phenyl)ureido)phenylamino)thieno[2,3-d]pyrimidine-6-carboxamido)propanoate (9c)

Yield 85% as grey solid, m.p 220 °C. **^1^HNMR (400 MHz, DMSO-d6) δ:** 1.17–1.21 (*t*, 3H, OCH_2_CH_3_, *J* = 8 Hz), 2.58–2.61 (t, 2H, CH_2_CO, *J* = 8 Hz), 3.01 (*s*, 3H, CH_3_), 3.71–3.78 (*q*, 2H, CH_2_NH, *J* = 8 Hz), 4.17–4.23 (*q*, 2H, OCH_2_CH_3_, *J* = 8 Hz), 7.29–7.31 (d, 1H, aromatic H_5_″, *J* = 8 Hz), 7.53–7.64 (*m*, 5H, aromatic H_2_′, H_3_′, H_5_′, H_6_′, H_4_″),8.01 (*s*, 1H, NH, exchangeable by D_2_O), 8.31–8.43 (*m*, 3H, aromatic H_2_″, H_6_″ and pyrimidine H), 8.86, 9.11 (*s* × 2, 2H, NH × 2,exchangeable by D_2_O). **MS: *m*/*z* (%):** 587 (M^+^+1, 17%), 586 (M^+^, 55%), 77 (100%). **Anal. Calcd. For C_27_H_25_F_3_N_6_O_4_S:** C 55.28, H 4.30, N 14.33, S 5.47; Found: C 55.22, H 4.27, N 14.30, S 5.45.

##### 5.1.10.4. Ethyl 3–(5-methyl-4-(phenylamino)thieno[2,3-d]pyrimidine-6-carboxamido)propanoate (17 b)

Yield 95% as light brown solid, m.p 206 °C . **^1^HNMR (400 MHz,DMSO) δ:** 1.19–1.23 (*t*, 3H, OCH_2_CH_3_, *J* = 8 Hz), 2.59–2.62 (t, 2H, CH_2_CO, *J* = 8 Hz), 2.86 (*s*, 3H, CH_3_), 3.48–3.53 (*q*, 2H, CH_2_NH, *J* = 8 Hz), 4.07–4.12 (*q*, 2H, OCH_2_CH_3_, *J* = 8 Hz), 7.14–7.17 (*t*, 1H, aromatic H_4_′, *J* = 8 Hz), 7.37–7.41 (*t*, 2H, aromatic H_3_′, H_5_′, *J* = 8 Hz), 7.64–7.66 (d, 2H, aromatic H_2_′, H_6_′, *J* = 8 Hz), 8.45 (s, 1H, pyrimidine H), 8.51 (*s*, 1H, NH, exchangeable by D_2_O). **MS: *m*/*z* (%):** 385 (M^+^+1, 7%), 384 (M^+^, 28%), 77 (100%). **Anal. Calcd. For C_19_H_20_N_4_O_3_S:** C 59.36, H 5.24, N 14.57, S 8.34; Found: C 59.48, H 5.37, N 14.49, S 8.47. **FT-IR (**ú **max, cm^−1^)**: 3452, 3224 (2 × NH stretch), 3047–3020 (CH aromatic), 2985–2904 (CH aliphatic), 1724 (C = O ester), 1651 (C = O amide), 1608 (NH bend).

##### 5.1.10.5. Ethyl 3–(4-(4-chlorophenylamino)-5-methylthieno[2,3-d]pyrimidine-6-carboxamidopropanoate (17c)

Yield 90% as buff solid, m.p 202 °C. **^1^HNMR (400 MHz, CDCl3) δ:** 1.29–1.34 (*t*, 3H, OCH_2_CH_3_, *J* = 8 Hz), 2.67–2.70 (t, 2H, CH_2_CO, *J* = 8 Hz), 3.02 (*s*, 3H, CH_3_), 3.69–3.77 (*m*, 2H, CH_2_NH), 4.19–4.24 (*q*, 2H, OCH_2_CH_3_, *J* = 8 Hz), 6.78 (*s*, 1H, NH, exchangeable by D_2_O), 7.38–7.40 (d, 2H, aromatic H_2_′, H_6_′, *J* = 8 Hz), 7.55 (*s*, 1H, NH, exchangeable by D_2_O), 7.63–7.65 (d, 1H, aromatic H_3_′, H_5_′, *J* = 8 Hz), 8.57 (*s*, 1H, pyrimidine H). **MS: *m*/*z* (%):** 420 (M^+^+2, 10%) & base peak at 418 (M^+^, 100%). **Anal. Calcd. For C_19_H_19_ClN_4_O_3_S:** C 54.48, H 4.57, N 13.37, S 7.65; Found: C 54.65, H 4.80, N 13.48, S 7.78.

##### 5.1.10.6. Ethyl 3–(4-(3-chloro-4-fluorophenylamino)-5-methylthieno[2,3-d]pyrimidine-6-carboxamido)propanoate (17d)

Yield 92% as buff solid, m.p 184 °C. **^1^HNMR (400 MHz, CDCl_3_) δ:** 1.30–1.34 (*t*, 3H, OCH_2_CH_3_, *J* = 8.8 Hz), 2.67–2.71 (*t*, 2H, CH_2_CO, *J* = 8 Hz), 3.04 (*s*, 3H, CH_3_), 3.73–3.77 (*q*, 2H, CH_2_NH, *J* = 8 Hz), 4.19–4.25 (*q*, 2H, OCH_2_CH_3_, *J* = 8.8 Hz), 6.73 ((*s*, 1H, NH, exchangeable by D_2_O), 7.19–7.23 (*t*, 1H, aromatic H_5_′, *J* = 8 Hz), 7.51 (*s*, 1H, aromatic H_2_′), 7.87–7.89 (d, 1H, aromatic H_6_′, *J* = 8 Hz), 8.60 (*s*, 1H, pyrimidine H). **MS: *m*/*z* (%):** 437 (M^+^+1, 18%), 436 (M^+^, 26%), 285 (100%). **Anal. Calcd. For C_19_H_18_ClFN_4_O_3_S:** C 52.23, H 4.15, N 12.82, S 7.34; Found: C 52.40, H 4.28, N 13.09, S 7.46. **FT-IR (**ú **max, cm^−1^)**: 3444, 3367 (2 × NH stretch), 3120 (CH aromatic), 2981–2858 (CH aliphatic), 1728 (C = O ester), 1639 (C = O amide), 1612 (NH bend).

#### 5.1.11. General procedure for preparation of compounds (10a-c, 18 b-d)

To a solution of ethyl 4-amino butyrate hydrochloride (8.94 mmol), prepared thieno[2,3-*d*]pyrimidine-6-carboxylic acid derivatives **(8a-c, 16 b-d)** (8.94 mmol) and 1‐hydroxybenzotriazole (1.32 g, 9.18 mmol) in dry dimethylformamide (20 ml), N‐methyl morpholine (4.51 g, 44.65 mmol) was added at 0 °C and stirred for 30 min. Then, EDCI.HCl (1.8 g, 13.12 mmol) was added and stirred for another 30 min maintaining the same temperature. The reaction mixture was stirred for 8 h at room temperature and then followed by the addition of water (20 ml) and ethyl acetate (2 × 20 ml) for extraction. Organic extracts were combined, washed with water (2 × 10 ml) and aqueous sodium bicarbonate solution (2 × 10 ml). Then the organic solution was dried with anhydrous Na_2_SO_4_, and evaporated under vacuum to afford the titled compounds **(10a-c, 18 b-d)**^29^.

##### 5.1.11.1. Ethyl 4–(5-methyl-4–(4-(3-phenylureido)phenylamino)thieno[2,3-d]pyrimidine-6-carboxamido)butanoate (10a)

Yield 84% as greyish white solid, m.p 219–221 °C. **^1^HNMR (400 MHz, DMSO-d6) δ:** 1.19–1.22 (*t*, 3H, OCH_2_CH_3_, *J* = 8 Hz), 1.77–1.84 (*m*, 2H, CH_2_CH_2_CH_2_), 2.36–2.40 (*t*, 2H, CH_2_CO, *J* = 8 Hz), 2.88 (*s*, 3H, CH_3_), 3.26–3.32 (*q*, 2H, CH_2_NH, *J* = 8 Hz), 4.05–4.10 (*q*, 2H, OCH_2_CH_3_, *J* = 8 Hz), 6.96–6.99 (t, 1H, aromatic H_4_″, *J* = 7.6 Hz), 7.27–7.31 (*t,* 2H, aromatic H_2_′, H_6_′, *J* = 8 Hz), 7.46–7.48 (d, 4H, aromatic H_2_″, H_3_″, H_5_″, H_6_″), 7.52–7.54 (d, 2H, aromatic H_3_′_,_ H_5_′, *J* = 8 Hz), 8.41 (*s*, 1H, pyrimidine H), 8.43, 8.46, 8.68, 8.71 (*s* × 4, 4H, NH × 4, exchangeable by D_2_O). **MS: *m*/*z* (%):** 532 (M^+^, 28%), 530 (M^+^-2, 14%), 452 (100%). **Anal. Calcd. For C_27_H_28_N_6_O_4_S:** C 60.89, H 5.30, N 15.78, S 6.02; Found: C 60.75, H 5.43, N 16.01, S 6.14.

##### 5.1.11.2. Ethyl 4–(4-(4–(3-(3-methoxyphenyl)ureido)phenylamino)-5-methylthieno[2,3-d]pyrimidine-6-carboxamido)butanoate (10b)

Yield 80% as yellow solid, m.p 193 °C. **^1^HNMR (400 MHz, DMSO-d6) δ:** 1.18–1.22 (*t*, 3H, OCH_2_CH_3_, *J* = 8 Hz), 1.77–1.84 (*m*, 2H, CH_2_CH_2_CH_2_), 2.36–2.40 (*t*, 2H, CH_2_CO, *J* = 8 Hz), 2.88 (*s*, 3H, CH_3_), 3.26–3.32 (*q*, 2H, CH_2_NH, *J* = 8 Hz), 3.74 (*s*, 3H, OCH_3_), 4.05–4.11 (*q*, 2H, OCH_2_CH_3_, *J* = 8 Hz), 6.54–7.56 (d, 1H, aromatic H_4_″, *J* = 8 Hz), 6.94–6.96 (d, 1H, aromatic H_5_″, *J* = 8 Hz), 7.16–7.21 (m, 2H, aromatic H_2_″, H_6_″), 7.46–7.49 (d, 2H, aromatic H_2_′, H_6_′, *J* = 12 Hz), 7.51–7.54 (d, 2H, aromatic H_3_′, H_5_′, *J* = 12 Hz), 8.41 (*s*, 1H, pyrimidine H), 8.43, 8.46, 8.89, 8.98 (*s* × 4, 4H, NH × 4, exchangeable by D_2_O). **MS: *m/z* (%):** 562 (M^+^, 41%), 530 (M^+^-2, 20%), 256 (100%). **Anal. Calcd. For C_28_H_30_N_6_O_5_S**: C 59.77, H 5.37, N 14.94, S 5.70; Found: C 59.64, H 5.48, N 15.12, S 5.78.

##### 5.1.11.3. Ethyl 4–(5-methyl-4–(4-(3–(3(trifluoromethyl)phenyl)ureido)phenylamino)thieno[2,3-d]pyrimidine-6- carboxamido)butanoate (10c)

Yield 68% as yellowish white solid, m.p 197–199 °C. **^1^HNMR (400 MHz, DMSO-d6) δ:** 1.18–1.22 (*t*, 3H, OCH_2_CH_3_, *J* = 8 Hz), 1.77–1.84 (*m*, 2H, CH_2_CH_2_CH_2_), 2.36–2.40 (*t*, 2H, CH_2_CO, *J* = 8 Hz), 2.88 (*s*, 3H, CH_3_), 3.26–3.32 (*q*, 2H, CH_2_NH, *J* = 8 Hz), 4.05–4.10 (*q*, 2H, OCH_2_CH_3_, *J* = 8 Hz), 7.31–7.32 (d, 1H, aromatic H_5_″, *J* = 8 Hz), 7.48–7.60 (*m*, 7H, aromatic H_2_′, H_3_′, H_5_′, H_6_′, H_2_″, H_4_″, H_6_″), 8.05 (*s*, 1H, NH, exchangeable by D_2_O), 8.42–8.44 (*m*, 2H, pyrimidine H & NH, exchangeable by D_2_O) , 8.88, 9.11 (*s* × 2, 2H, NH × 2,exchangeable by D_2_O). **MS: m/z (%):** 600 (M^+^, 28%), 601 (M^+^+1, 28%), 77 (100%). **Anal. Calcd. For C_28_H_27_F_3_N_6_O_4_S:** C 55.99, H 4.53, N 13.99, S 5.34; Found: C 56.18, H 4.76, N 14.21, S 5.28. **FT-IR (**ú **max, cm^ − 1^)**: 3375, 3356, 3271 (3 × NH stretch), 3155–3070 (CH aromatic), 2978–2873 (CH aliphatic), 1712 (C = O ester), 1643(C = O amide), 1612 (NH bend).

##### 5.1.11.4. Ethyl 4–(5-methyl-4-(phenylamino)thieno[2,3-d]pyrimidine-6-carboxamido)butanoate (18 b)

Yield 94% as off white solid, m.p 145–147 °C. **^1^HNMR (400 MHz,CDCl_3_) δ:** 1.27–1.31 (*t*, 3H, OCH_2_CH_3_, *J* = 8 Hz), 1.97–2.04 (*m*, 2H, CH_2_CH_2_CH_2_), 2.46–2.50 (*t*, 2H, CH_2_CO, *J* = 8 Hz), 3.05 (*s*, 3H, CH_3_), 3.50–3.55 (*q*, 2H, CH_2_NH, *J* = 8 Hz), 4.16–4.21 (*q*, 2H, OCH_2_CH_3_, *J* = 6.8 Hz), 6.38 (*s*, 1H, NH, exchangeable by D_2_O), 7.20–7.24 (*t*, 1H, aromatic H_4_′, *J* = 8 Hz), 7.42–7.46 (*t*, 2H, aromatic H_3_′, H_5_′, *J* = 8 Hz), 7.67–7.69 (d, 2H, aromatic H_2_′, H_6_′, *J* = 8 Hz), 8.59 (*s*, 1H, pyrimidine H). **MS: *m/z* (%):** 398 (M^+^, 15%), 399 (M^+^+1, 4%), 130 (100%). **Anal. Calcd. For C_20_H_22_N_4_O_3_S:** C 60.28, H 5.56, N 14.06, S 8.05; Found: C 60.41, H 5.75, N 14.32, S 8.19.

##### 5.1.11.5. Ethyl 4–(4-(4-chlorophenylamino)-5-methylthieno[2,3-d]pyrimidine-6-carboxamido)butanoate (18c)

Yield 96% as pale yellow solid, m.p 138 °C. **^1^HNMR (400 MHz, DMSO) δ:** 1.18–1.22 (*t*, 3H, OCH_2_CH_3_, *J* = 8 Hz), 1.76–1.84 (*m*, 2H, CH_2_CH_2_CH_2_), 2.36–2.40 (*t*, 2H, CH_2_CO, *J* = 8 Hz), 2.87 (*s*, 3H, CH_3_), 3.26–3.31 (*q*, 2H, CH_2_NH, *J* = 8 Hz), 4.05–4.10 (*q*, 2H, OCH_2_CH_3_, *J* = 8 Hz), 7.43–7.45 (d, 2H, aromatic H_2_′, H_6_′, *J* = 8 Hz), 7.69–7.71 (d, 1H, aromatic H_3_′, H_5_′, *J* = 8 Hz), 8.47 (br s, 1H, NH, exchangeable by D_2_O), 8.49 (*s*, 1H, pyrimidine H), 8.59 (*s*, 1H, NH, exchangeable by D_2_O). **MS: *m*/*z* (%):** 433 (M^+^+ 1, 36%), 434 (M^+^+2, 35%), 432 (M^+^, 100%). **Anal.Calcd. For C_20_H_21_ClN_4_O_3_S:** C 55.49, H 4.89, N 12.94, S 7.41; Found: C 55.38, H 5.12, N 12.87, S 7.48.

##### 5.1.11.6. Ethyl 4–(4-(3-chloro-4-fluorophenylamino)-5-methylthieno[2,3-d]pyrimidine-6-carboxamido)butanoate (18d)

Yield 90% as faint yellow solid, m.p 131–133 °C. **^1^HNMR (400 MHz, DMSO-d6) δ:** 1.18–1.21 (*t*, 3H, OCH_2_CH_3_, *J* = 8 Hz), 1.77–1.84 (*m*, 2H, CH_2_CH_2_CH_2_), 2.36–2.40 (*t*, 2H, CH_2_CO, *J* = 8 Hz), 2.87 (*s*, 3H, CH_3_), 3.26–3.32 (*q*, 2H, CH_2_NH, *J* = 8 Hz), 4.05–4.10 (*q*, 2H, OCH_2_CH_3_, *J* = 8 Hz), 7.43–7.47 (*t*, 1H, aromatic H_5_′, *J* = 8 Hz), 7.63–7.67 (*m*, 1H, aromatic H_2_′), 7.92–7.94 (dd, 1H, aromatic H_6_′, *J* = 2.8, 6.8 Hz) , 8.48 (*s*, 1H, NH, exchangeable by D_2_O), 8.50 (*s*, 1H, pyrimidine H), 8.60 (*s*, 1H, NH, exchangeable by D_2_O). **MS: *m/z* (%):** 450 (M^+^, 30%), 434 (M^+^+2, 35%), 110 (100%), 66 (85%). **Anal. Calcd. For C_20_H_20_ClFN_4_O_3_S**: C 53.27, H 4.47, N 12.43, S 7.11; Found: C 53.49, H 4.61, N 12.69, S 7.17.

#### 5.1.12. General procedure for preparation of compounds (11a-c, 19 b-d, 12a-c and 20b-d)

A cooled solution of sodium (27 mmol; 10 equiv.) in absolute ethanol (20 ml) was added to a solution of hydroxylamine hydrochloride (54 mmol; 20 equiv.) in absolute ethanol cooled in an ice bath. The mixture was stirred for 15 min. The formed precipitate was filtered, and the free hydroxylamine solution was prepared. The freshly prepared hydroxylamine solution was placed in a rounded bottom flask and cooled in an ice bath. Compounds **(9a-c, 17 b-d, 10a-c, 18 b-d)** (2.7 mmol; 1 equiv.) were added to the solution and stirred overnight. The reaction is monitored by TLC till the complete disappearance of starting compounds using an appropriate eluting system [DCM: MeOH (9.5:0.5)]. The solvent was removed under vacuum to afford crude products, washed with water, and crystallised from ethanol to yield target compounds **(11a-c, 19 b-d, 12a-c, 20b-d)**^26^^,^^27^.

##### 5.1.12.1. 1–(4-(6-((3-(hydroxyamino)-3-oxopropyl)carbamoyl)-5-methylthieno[2,3-d]pyrimidin-4-ylamino)phenyl)-3-phenylurea (11a)

Yield 80% as yellow solid, m.p 225 °C. **^1^HNMR (400 MHz, DMSO-d6) δ:** 2.36–2.39 (*t*, 2H, CH_2_CO, *J* = 8 Hz), 2.90 (*s*, 3H, CH_3_), 3.47–3.52 (*q*, 2H, CH_2_NH, *J* = 8 Hz) , 6.88–6.91 (*t*, 1H, aromatic H_4_″, *J* = 8 Hz), 7.20–7.28 (*m*, 4H, aromatic H_2_′,H_6_′, H_3_″, H_5_″), 7.23–7.25 (d, 2H, aromatic H_2_′, H_6_′, *J* = 8 Hz), 7.50–7.52 (d, 2H, aromatic H_3_′,H_5_′, *J* = 8 Hz), 7.58–7.60 (d, 2H, aromatic H_2_″_,_ H_6_″, *J* = 8 Hz), 8.14 (*s*, 1H, NH, exchangeable by D_2_O), 8.28 (*s*, 1H, pyrimidine H), 8.40, 9.09, 9.42, 10.89 (*s* × 4, 4H, NH × 4, exchangeable by D_2_O), 11.01 (*s*, 1H, OH, exchangeable by D_2_O). **MS: *m*/*z* (%):** 505 (M^+^, 28%),504 (M^+^-1, 36%), 306 (100%). **Anal. Calcd. For C_24_H_23_N_7_O_4_S:** C 57.02, H 4.59, N 19.39, S 6.34; Found: C 57.21, H 4.80, N 19.47, S 6.41.

##### 5.1.12.2. 1–(4-(6-((3-(hydroxyamino)-3-oxopropyl)carbamoyl)-5-methylthieno[2,3-d]pyrimidin-4-ylamino)phenyl)-3–(3-methoxyphenyl)urea (11 b)

Yield 82% as yellow solid, m.p 257–260 °C. **^1^HNMR (400 MHz, DMSO-d6) δ:** 2.36–2.39 (*t*, 2H, CH_2_CO, *J* = 8 Hz), 2.89 (*s*, 3H, CH_3_), 3.47–3.52 (*q*, 2H, CH_2_NH, *J* = 8 Hz), 3.72 (*s*, 3H, OCH_3_), 6.47–6.49 (*m*, 1H, aromatic H_4_″), 7.11–7.13 (d, 2H, aromatic H_5_″, H_6_″, *J* = 8 Hz), 7.17–7.19 (d, 2H, aromatic H_2_′, H_6_′, *J* = 8 Hz), 7.37 (*s*,1H, aromatic H_2_″), 7.49–7.52 (d, 2H, aromatic H_3_′, H_5_′, *J* = 8 Hz), 8.12 (s, 1H, NH, exchangeable by D_2_O) 8.26 (*s*, 1H, pyrimidine H), 9.09, 10.99, 11.12 (s × 3, 3H, NH × 3,exchangeable by D_2_O). **MS: *m/z* (%):** 535 (M^+^, 15%), 537 (M^+^+2, 8%), 93 (100%). **Anal. Calcd. For C_25_H_25_N_7_O_5_S:** C 56.06, H 4.70, N 18.31, S 5.99; Found: C 56.14, H 4.88, N 18.49, S 6.07.

##### 5.1.12.3. 1–(4-(6-((3-(hydroxyamino)-3-oxopropyl)carbamoyl)-5-methylthieno[2,3-d]pyrimidin-4-ylamino)phenyl)-3–(3-(trifluoromethyl)phenyl)urea (11c)

Yield 79% as brown solid, m.p 281 °C. **^1^HNMR (400 MHz, DMSO-d6) δ:** 2.28–2.32 (*t*, 2H, CH_2_CO, *J* = 8 Hz) , 2.90 (*s*, 3H, CH_3_), 2.60 (*s*, 1H, OH, exchangeable by D_2_O), 3.45–3.48 (*m*, 2H, CH_2_NH), 7.22–7.24 (d, 1H, aromatic H_2_′, *J* = 8 Hz), 7.31–7.33 (d, 1H, aromatic H_6_′, *J* = 8) , 7.45–7.49 (t, 1H, aromatic H_5_′, *J* = 8 Hz), 7.53–7.55 (d, 1H, aromatic H_5_″, *J* = 8 Hz) , 7.73–7.75 (d, 1H, aromatic H_3_′, *J* = 8 Hz) , 7.95 (*s*, 1H, aromatic H_4_″), 8.13 (s, 1H, aromatic H_6_″), 8.23 (*s*, 1H, aromatic H_2_″), 8.35 (*s*, 1H, NH, exchangeable by D_2_O′), 8.51 (*s*, 1H, pyrimidine H), 9.00, 11.2, 11.5 (*s* × 3, 3H, NH × 3, exchangeable by D_2_O). **MS: *m*/*z* (%):** 575 (M^+^+2, 22%), 573 (M^+^, 52%), 140 (100%) **Anal. Calcd. For C_25_H_22_F_3_N_7_O_4_S:** C 52.35, H 3.87, N 17.09, S 5.59; Found: C 52.48, H 3.95, N 17.31, S 5.48.

##### 5.1.12.4. N-(3-(hydroxyamino)-3-oxopropyl)-5-methyl-4-(phenylamino)thieno[2,3-d]pyrimidine-6-carboxamide (19 b)

Yield 70% as yellow solid, m.p 267–270 °C. **^1^HNMR (400 MHz,CDCl_3_) δ:** 2.17–2.21 (*t*, 2H, CH_2_CO, *J* = 8 Hz), 2.90 (*s*, 3H, CH_3_), 3.36–3.40 (*t*, 2H, CH_2_NH, *J* = 8 Hz), 7.12–7.16 (*t*, 1H, aromatic H_4_′, *J* = 8 Hz), 7.36–7.40 (*t*, 2H, aromatic H_3_′, H_5_′, *J* = 8 Hz), 7.64–7.66 (d, 2H, aromatic H_2_′, H_6_′, *J* = 8 Hz), 8.45 (*s*, 1H, pyrimidine H), 8.49, 8.53, 8.84 (*s* × 3, 3H, NH × 3, exchangeable by D_2_O), 10.30 (*s*, 1H, OH, exchangeable by D_2_O). **^13 ^C NMR (DMSO-d6, 400 MHz) δ:** 16.20, 37.29, 37.73, 123.37, 124.39, 128.94, 139.36, 142.88, 154.50, 157.14, 159.06, 166.31, 173.12, 176.50. **MS: *m/z* (%):** 373 (M^+^+2, 22%), 371 (M^+^, 41%), 309 (100%). **Anal. Calcd. For C_17_H_17_N_5_O_3_S:** C 54.97, H 4.61, N 18.86, S 8.63; Found: C 55.16, H 4.89, N 18.72, S 8.75.

##### 5.1.12.5. 4–(4-chlorophenylamino)-N-(3-(hydroxyamino)-3-oxopropyl)-5-methylthieno[2,3-d]pyrimidine-6-carboxamide (19c)

Yield 75% as yellowish orange solid, m.p 282 °C. **^1^HNMR (400 MHz, DMSO-d6) δ:** 1.04–1.08 (*t*, 2H, CH_2_CO, *J* = 8 Hz), 2.09 (*s*, 3H, CH_3_), 3.38–3.46 (*m*, 2H, CH_2_NH), 4.40 (*s*, 1H, NH, exchangeable by D_2_O), 7.41–7.43 (d, 2H, aromatic H_3_′, H_5_′, *J* = 8 Hz), 7.69–7.71 (d, 2H, aromatic H_2_′, H_6_′, *J* = 8 Hz), 8.46 (*s*, 1H, pyrimidine H), 8.67, 8.90 (*s* × 2, 2H, NH × 2, exchangeable by D_2_O). **MS: *m/z* (%):** 407 (M^+^+2, 66%), 406 (M^+^+1, 41%), 405 (M^+^, 19%), 352 (100%). **Anal. Calcd. For C_17_H_16_ClN_5_O_3_S:** C 50.31, H 3.97, N 17.26, S 7.90; Found: C 50.49, H 4.18, N 17.40, S 7.86.

##### 5.1.12.6. 4–(3-chloro-4-fluorophenylamino)-N-(3-(hydroxyamino)-3-oxopropyl)-5-methylthieno[2,3-d]pyrimidine-6-carboxamide (19d)

Yield 78% as yellow solid, m.p. 276 °C. **^1^HNMR (400 MHz, DMSO-d6) δ:** 2.09 (*s*, 1H, OH, exchangeable by D_2_ O), 2.18–2.22 (*t*, 2H, CH_2_CO, *J* = 8 Hz), 2.89 (*s*, 3H, CH_3_), 3.42– 3.47 (*m*, 2H, CH_2_NH), 7.39–7.44 (*t*, 1H, aromatic H_5_′, *J* = 8 Hz), 7.62–7.64 (*m*, 1H, aromatic H_2_′), 7.90–7.93 (dd, 1H, aromatic H_6_′, *J* = 4 Hz, 8 Hz) , 8.47–8.48 (*m*, 2H, pyrimidine H, NH exchangeable by D_2_O), 8.78 (s, 1H, NH, exchangeable by D_2_O). **MS: *m/z* (%):** 423 (M^+^, 49%), 420, (M^+^-2, 10%), 76 (100%). **Anal. Calcd. For C_17_H_15_ClFN_5_O_3_S:** C 48.17, H 3.57, N 16.52, S 7.57; Found: C48.40, H 3.71, N 16.80, S 7.63.

##### 5.1.12.7. 1–(4-(6-((4-(hydroxyamino)-4-oxobutyl)carbamoyl)-5-methylthieno[2,3-d]pyrimidin-4-ylamino)phenyl)-3-phenylurea (12a)

Yield 69% as yellow solid, m.p 219 °C. **^1^HNMR (400 MHz, DMSO-d6) δ:** 1.77–1.84 (*m*, 2H, CH_2_CH_2_CH_2_), 2.18–2.22 (*t*, 2H, CH_2_CO, *J* = 8 Hz), 2.88 (*s*, 3H, CH_3_), 3.26–3.35 (*m*, 2H, CH_2_NH), 6.90–6.94 (*t*, 1H, aromatic H_4_″, *J* = 8 Hz), 7.23–7.27 (*t*, 2H, aromatic H_3_″, H_5_″, *J* = 8 Hz), 7.40–7.42 (d, 2H, aromatic H_2_′, H_6_′, *J* = 8 Hz), 7.47–7.49 (d, 2H, aromatic H_3_′_,_ H_5_′, *J* = 8 Hz), 7.53–7.55 (d, 2H, aromatic H_2_″_,_ H_6_″, *J* = 8 Hz), 8.22, 8.28 (s × 2, 2H, NH × 2, exchangeable by D_2_O), 8.36 (s, 1H, pyrimidine H), 8.41, 9.01, 9.50 (*s* × 3, 3H, NH × 3, exchangeable by D_2_O), 9.98 (s, 1H, OH, exchangeable by D_2_O). **MS: *m*/*z* (%):** 519 (M^+^, 27%), 516 (M^+^-3, 20%), 432 (100%). **Anal. Calcd. For C_25_H_25_N_7_O_4_S:** C 57.79, H 4.85, N 18.87, S 6.17; Found: C 57.80, H 4.82, N 18.89, S 6.16

##### 5.1.12.8. 1–(4-(6-((4-(hydroxyamino)-4-oxobutyl)carbamoyl)-5-methylthieno[2,3-d]pyrimidin-4-ylamino)phenyl)-3–(3-methoxyphenyl)urea (12 b)

Yield 67% as orange solid, m.p 231–233 °C. **^1^HNMR (400 MHz, DMSO-d6) δ:** 1.71–1.80 (*m*, 2H, CH_2_CH_2_CH_2_), 2.19–2.22 (*t*, 2H, CH_2_CO, *J* = 8 Hz), 2.87 (*s*, 3H, CH_3_), 3.31–3.41 (*m*, 2H, CH_2_NH), 3.72 (*s*, 3H, OCH_3_), 6.46–7.48 (d, 1H, aromatic H_4_″, *J* = 8 Hz), 7.09–7.13 (*m*, 2H, aromatic H_5_″, H_6_″), 7.30–7.32 (d, 2H, aromatic H_2_′, H_6_′, *J* = 8 Hz), 7.33 (*s*, 1H, aromatic H_2_″), 7.44–7.46 (d, 2H, aromatic H_3_′, H_5_′, *J* = 8 Hz), 8.18 (*s*, 1H, NH, exchangeable by D_2_O), 8.30 (*s*, 1H, pyrimidine H), 8.52, 9.53, 10.76, 10.86 (*s* × 4, 3H, NH × 3,exchangeable by D_2_O). **MS: *m/z* (%):** 549 (M^+^, 28%), 547 (M^+^-2, 16%), 221 (100%). **Anal. Calcd. For C_26_H_27_N_7_O_5_S:** C 56.82, H 4.95, N 17.84, S 5.83; Found: C 57.04, H 5.19, N 17.71, S 5.96. **FT-IR (**ú **max, cm^ − 1^)**: 3441 (OH broad), 3317 (NH stretch), 2958 (CH aromatic), 2931–2835 (CH aliphatic), 1708 (C = O), 1643 (C = O amide), 1612 (NH bend).

##### 5.1.12.9. 1–(4-(6-((4-(hydroxyamino)-4-oxobutyl)carbamoyl)-5-methylthieno[2,3-d]pyrimidin-4-ylamino)phenyl)-3–(3-(trifluoromethyl)phenyl)urea (12c)

Yield 72% as yellow solid, m.p. 215–217 °C. **^1^HNMR (400 MHz, DMSO-d6) δ:** 1.75–1.84 (*m*, 2H, CH_2_CH_2_CH_2_), 2.09–2.24 (*m*, 2H, CH_2_CO), 2.88 (*s*, 3H, CH_3_), 3.38–3.45 (*m*, 2H, CH_2_NH), 7.22–7.25 (*t*, 1H, aromatic H_6_′, *J* = 8 Hz), 7.35–7.37 (d, 1H, aromatic H_2_′, *J* = 8 Hz), 7.45–7.47 (d, 1H, aromatic H_5_′, *J* = 8 Hz), 7.49–7.51 (d, 1H, aromatic H_3_′, *J* = 8 Hz), 7.54–7.56 (d, 1H, aromatic H_4_″), 7.67–7.73 (dd, 1H, aromatic H_6_″, *J* = 8 Hz, 16 Hz), 8.09–8.12 (d, 1H, aromatic H_5_″), 8.32 (*s*, 1H, aromatic H_2_″), 8.41 (*s*, 1H, pyrimidine H), 8.53, 9.44, 10.53, 10.85, 10.93, (s × 5, 5H, NH × 5,exchangeable by D_2_O), 11.39 (s, 1H, OH, exchangeable by D_2_O). **MS: *m/z* (%):** 587 (M^+^, 64%), 255 (100%), 205 (93%). **Anal. Calcd. For C_26_H_24_F_3_N_7_O_4_S:** C 53.15, H 4.12, N 16.69, S 5.46; Found: C 53.31, H 4.26, N 16.83, S 5.60.

##### 5.1.12.10. N-(4-(hydroxyamino)-4-oxobutyl)-5-methyl-4-(phenylamino)thieno[2,3-d]pyrimidine-6-carboxamide (20 b)

Yield 75% as yellow solid, m.p 205 °C. **^1^HNMR (400 MHz,CDCl_3_) δ:** 1.78 (*m*, 2H, CH_2_CH_2_CH_2_), 2.06 (*m*, 2H, CH_2_CO), 2.88 (*s*, 3H, CH_3_), 3.25–3.36 (*m*, 2H, CH_2_NH), 7.16–7.65 (*m*, 5H, aromatic H), 8.45–8.49 (*m*, 2H, pyrimidine H, NH), 8.77 (*s*, 1H, NH, exchangeable by D_2_O). **MS: *m/z* (%):** 385 (M^+^, 12%), 383 (M^+^-2, 5%), 308 (100%). **Anal. Calcd. For C_18_H_19_N_5_O_3_S:** C 56.09, H 4.97, N 18.17, S 8.32; Found: C 56.24, H 4.89, N 18.40, S 8.45. **FT-IR (**ú **max, cm^−1^)**: 3452, 3232 (2 × NH stretch), 3410 (OH/NH broad), 3059–2970 (CH aromatic), 2931–2823 (CH aliphatic), 1647 (C=O amide), 1608 (NH bend).

##### 5.1.12.11. 4–(4-chlorophenylamino)-N-(4-(hydroxyamino)-4-oxobutyl)-5-methylthieno[2,3-d]pyrimidine-6-carboxamide (20c)

Yield 78% as yellow solid, m.p 212 °C. **^1^HNMR (400 MHz, DMSO-d6) δ:** 1.65 (*s*, 1H, OH, exchangeable by D_2_O), 1.69–1.76 (*m*, 2H, CH_2_CH_2_CH_2_), 2.06–2.09 (*t*, 2H, CH_2_CO, *J* = 8 Hz), 2.88 (*s*, 3H, CH_3_), 3.23–3.27 (*q,* 2H, CH_2_NH, *J* = 8 Hz), 7.41–7.43 (d, 2H, aromatic H_2_′, H_6_′, *J* = 8 Hz), 7.69–7.71 (d, 1H, aromatic H_3_′, H_5_′, *J* = 8 Hz), 8.46 (*s*, 1H, pyrimidine H), 8.50, 8.64, 9.63 (*s* × 3, 3H, NH × 3,exchangeable by D_2_O). **^13 ^C NMR (DMSO-d6, 400 MHz) δ:** 16.25, 22.69, 25.65, 36.32, 122.16, 124.90, 128.77, 129.29, 147.56, 147.94, 131.70, 150.08, 154.32, 160.59, 162.43, 166.44. **MS: *m/z* (%):** 419 (M^+^, 23%), 421 (M^+^+1, 31%), 98 (100%). **Anal. Calcd. For C_18_H_18_ClN_5_O_3_S:** C 51.49, H 4.32, N 16.68, S 7.64; Found: C 51.63, H 4.58, N 16.79, S 7.58.

##### 5.1.12.12. 4–(3-chloro-4-fluorophenylamino)-N-(4-(hydroxyamino)-4-oxobutyl)-5-methylthieno[2,3-d]pyrimidine-6-carboxamide (20d)

Yield 80% as brown solid, m.p 244–246 °C. **^1^HNMR (400 MHz, DMSO-d6) δ:** 1.71–1.76 (*m*, 2H, CH_2_CH_2_CH_2_), 2.09–2.13 (*t*, 2H, CH_2_CO, *J* = 8 Hz), 2.87 (*s*, 3H, CH_3_), 3.22–3.28 (*q*, 2H, CH_2_NH, *J* = 8 Hz), 7.40–7.44 (*t*, 1H, aromatic H_5_′*, J* = 8 Hz), 7.63–7.65 (*m,* 1H, aromatic H_2_′), 7.91–7.93 (d, 1H, aromatic H_6_′, *J* = 8 Hz) , 8.47 (*s*, 1H, pyrimidine H), 9.43 (*s*, 1H, NH, exchangeable by D_2_O). **MS: *m/z* (%):** 439 (M^+^+2, 26%), 437 (M^+^, 100%), 436 (M^+^-1, 84%). **Anal. Calcd. For C_18_H_17_ClFN_5_O_3_S:** C 49.37, H 3.91, N 15.99, S 7.32; Found: C 49.60, H 4.04, N 16.12, S 7.30.

### 5.2. Biological evaluation

#### 5.2.1. *In vitro* EGFR and VEGFR-2 tyrosine kinase inhibitory activity

*In vitro* enzyme inhibition determination for the synthesised compounds was carried out in Thermo Fischer Scientific, Madison, WI, USA (SelectScreenServices@thermofisher.com). The EGFR and VEGFR-2 tyrosine kinase % inhibitory activity at a single-dose concentration of 10 µM was performed initially then representative compounds were evaluated at single-dose concentrations of (0.1 nM, 1 nM, 10 nM, 100 nM, 1 µM) using ZYLTE technology. A fluorescence-based, coupled-enzyme format is employed in the biochemical assay and depends on the sensitivity of proteolytic cleavage to non-phosphorylated and phosphorylated peptides. Then IC50 values for selected compounds were determined by GraphPad Prism at Misr international university.

#### 5.2.2. *In vitro* HDAC inhibitory activity

##### 5.2.2.1. Materials and methods

The HDAC inhibitory activity was carried out at BPS Bioscience (www.bpsbioscience.com). All of the synthesised compounds were tested at 10 μM for their inhibitory percent against HDAC6. HDAC6 (BPS#50006), HDAC substrate 3 (BPS#50037), HDAC Assay Buffer (BPS catalogue number 50031) and HDAC Assay Developer (BPS catalogue number 50030) was used in testing. TSN is also used as a reference compound purchased from Selleckcom.com (#S1045).

##### 5.2.2.2. HDAC6 inhibitory activity assay protocols

All compounds are dissolved in DMSO. Serial dilutions of the compounds were carried out with 10% DMSO in HDAC assay buffer and 5 µl of the dilution was mixed to a 50 µl reaction so that the final concentration of DMSO is 1% in all of the reactions. Duplicate enzymatic reactions were conducted at 37 °C in a 50 µl mixture containing HDAC assay buffer, 5 µg BSA, HDAC substrate 3, HDAC6 (catalogue# 50006), and a test compound (10 µM) for 30 to 60 min. 50 μl of 2 × HDAC Developer was added to each well after enzymatic reaction, and the plate was incubated at room temperature for another 15 min. Fluorescence intensity was measured using a Tecan Infinite M1000 microplate reader at an excitation of 360 nm and an emission of 460 nm

##### 5.2.2.3. Data analysis

Duplicates of HDAC activity assays were performed at each concentration. Analysis of the fluorescent intensity data was performed using the Graphpad Prism computer software., the % activity was calculated according to the equation: % activity = (F-F_b_)/(F_t_-F_b_), where F = the fluorescent intensity in each data set in the presence of the compound, F_t_ = the fluorescent intensity in each data set, defined as 100% activity in the absence of the compound, F_b_ = the fluorescent intensity in each data set, defined as 0% activity in the absence of HDAC.

#### 5.2.3. *In vitro* anticancer activity

All of the synthesised compounds (20) were submitted to NCI and selected for *in vitro* anticancer assay against a panel of 60 cancer cell lines. The methodology of assay and calculations is as reported [54,55].

## Supplementary Material

Supplemental Material

## References

[1] Cai X, Zhai H-X, Wang J, et al. Discovery of 7-(4-(3-ethynylphenylamino)-7-methoxyquinazolin-6-yloxy)-N-hydroxyheptanamide (CUDc-101) as a potent multi-acting HDAC, EGFR, and HER2 inhibitor for the treatment of cancer. J Med Chem 2010;53:2000–9. 20143778 10.1021/jm901453q

[2] Falkenberg KJ, Johnstone RW. Histone deacetylases and their inhibitors in cancer, neurological diseases and immune disorders. Nat Rev Drug Discov 2014;13:673–91. 25131830 10.1038/nrd4360

[3] Lu H, Chen Y-d, Yang B, You Q-d. Design, synthesis and biological evaluation of novel histone deacetylase inhibitors based on virtual screening. Acta Pharmaceutica Sinica B 2011;1:240–7.

[4] Depetter Y, Geurs S, De Vreese R, et al. Selective pharmacological inhibitors of HDAC6 reveal biochemical activity but functional tolerance in cancer models. Int J Cancer 2019;145:735–47. 30694564 10.1002/ijc.32169

[5] Yang F, Zhao N, Ge D, Chen Y. Next-generation of selective histone deacetylase inhibitors. RSC Advances 2019;9:19571–83. 10.1039/c9ra02985k PMC9065321 35519364

[6] Yang Z, Wang T, Wang F, et al. Discovery of Selective Histone Deacetylase 6 Inhibitors Using the Quinazoline as the Cap for the Treatment of Cancer. J Med Chem 2016;59:1455–70. 26443078 10.1021/acs.jmedchem.5b01342

[7] Yang W, Li L, Ji X, et al. Design, synthesis and biological evaluation of 4-anilinothieno[2,3-d]pyrimidine-based hydroxamic acid derivatives as novel histone deacetylase inhibitors. Bioorg Med Chem 2014;22:6146–55. 25261927 10.1016/j.bmc.2014.08.030

[8] Guha M. HDAC inhibitors still need a home run, despite recent approval. Nat Rev Drug Discov 2015;14:225–6. 25829268 10.1038/nrd4583

[9] Suraweera A, O'Byrne KJ, Richard DJ. Combination therapy with histone deacetylase inhibitors (HDACi) for the treatment of cancer: achieving the full therapeutic potential of HDACi. Front Oncol 2018;8:92. 29651407 10.3389/fonc.2018.00092 PMC5884928

[10] Witta SE, Dziadziuszko R, Yoshida K, et al. ErbB-3 expression is associated with E-cadherin and their coexpression restores response to gefitinib in non-small-cell lung cancer (NSCLC). Ann Oncol 2009;20:689–695. 19150934 10.1093/annonc/mdn703 PMC2722370

[11] Aggarwal R, Thomas S, Pawlowska N, et al. Inhibiting histone deacetylase as a means to reverse resistance to angiogenesis inhibitors: phase I study of abexinostat plus pazopanib in advanced solid tumor malignancies. J Clin Oncol 2017;35:1231–1239. 28221861 10.1200/JCO.2016.70.5350 PMC5791833

[12] Zang J, Liang X, Huang Y, et al. Discovery of novel pazopanib-based HDAC and VEGFR dual inhibitors targeting cancer epigenetics and angiogenesis simultaneously. J Med Chem 2018;61:5304–5322. 29787262 10.1021/acs.jmedchem.8b00384

[13] Ding C, Li D, Wang Y-W, et al. Discovery of ErbB/HDAC inhibitors by combining the core pharmacophores of HDAC inhibitor vorinostat and kinase inhibitors vandetanib, BMS-690514, neratinib, and TAK-285. Chinese Chem Lett 2017;28:1220–1227.

[14] Peng FW, Xuan J, Wu TT, et al. Design, synthesis and biological evaluation of N-phenylquinazolin-4-amine hybrids as dual inhibitors of VEGFR-2 and HDAC. Eur J Med Chem 2016;109:1–12. 26741358 10.1016/j.ejmech.2015.12.033

[15] Mghwary AES, Gedawy EM, Kamal AM, Abuel-Maaty SM. Novel thienopyrimidine derivatives as dual EGFR and VEGFR-2 inhibitors: design, synthesis, anticancer activity and effect on cell cycle profile. J Enzyme Inhib Med Chem 2019;34:838–852. 30919701 10.1080/14756366.2019.1593160 PMC6442109

[16] Wedge SR, Ogilvie DJ, Dukes M, et al. ZD6474 inhibits vascular endothelial growth factor signaling, angiogenesis, and tumor growth following oral administration. Cancer Res 2002;62:4645–4655. 12183421

[17] Morabito A, Piccirillo MC, Falasconi F, et al. Vandetanib (ZD6474), a dual inhibitor of vascular endothelial growth factor receptor (VEGFR) and epidermal growth factor receptor (EGFR) tyrosine kinases: current status and future directions. The Oncologist 2009;14:378–390. 19349511 10.1634/theoncologist.2008-0261

[18] Yang W, Li L, Wang Y, et al. Design, synthesis and biological evaluation of isoquinoline-based derivatives as novel histone deacetylase inhibitors. Bioorg Med Chem 2015;23:5881–5890. 26211462 10.1016/j.bmc.2015.06.071

[19] Butler KV, Kalin J, Brochier C, et al. Rational design and simple chemistry yield a superior, neuroprotective HDAC6 inhibitor, tubastatin A. J Am Chem Soc 2010;132:10842–10846. 20614936 10.1021/ja102758v PMC2916045

[20] Wang F, Zheng L, Yi Y, et al. SKLB-23bb, A HDAC6-Selective Inhibitor. Exhibits Superior and Broad-Spectrum Antitumor Activity via Additionally Targeting Microtubules, Molecular Cancer Therapeutics 2018;17:763–775. 29610282 10.1158/1535-7163.MCT-17-0332

[21] Dewal MB, Wani AS, Vidaillac C, et al. Thieno[2,3-d]pyrimidinedione derivatives as antibacterial agents. Eur J Med Chem 2012;51:145–153. 22405289 10.1016/j.ejmech.2012.02.035 PMC3340521

[22] Adel M, Serya RAT, Lasheen DS, Abouzid KAM. Identification of new pyrrolo[2,3-d]pyrimidines as potent VEGFR-2 tyrosine kinase inhibitors: Design, synthesis, biological evaluation and molecular modeling. Bioorg Chem 2018;81:612–629. 30248512 10.1016/j.bioorg.2018.09.001

[23] Dai Y, Hartandi K, Ji Z, et al. Discovery of N-(4-(3-amino-1H-indazol-4-yl)phenyl)-N'-(2-fluoro-5-methylphenyl)urea (ABT-869), a 3-aminoindazole-based orally active multitargeted receptor tyrosine kinase inhibitor. J Med Chem 2007;50:1584–1597. 17343372 10.1021/jm061280h

[24] Suzuki T KM, Sawada H, Imai E, et al. Design, synthesis, and biological activity of a novel series of human sirtuin-2-selective inhibitors. J Med Chem 2012;55:5760–5773. 22642300 10.1021/jm3002108

[25] Wu CH, Coumar MS, Chu CY, et al. Design and synthesis of tetrahydropyridothieno[2,3-d]pyrimidine scaffold based epidermal growth factor receptor (EGFR) kinase inhibitors: the role of side chain chirality and Michael acceptor group for maximal potency. J Med Chem 2010;53:7316–7326. 20961149 10.1021/jm100607r

[26] Kassab SE, Mowafy S, Alserw AM, et al. Structure-based design generated novel hydroxamic acid based preferential HDAC6 lead inhibitor with on-target cytotoxic activity against primary choroid plexus carcinoma. J Enzyme Inhib Med Chem 2019;34:1062–1077. 31072216 10.1080/14756366.2019.1613987 PMC6522981

[27] Zuo M, Zheng Y-W, Lu S-M, et al. Synthesis and biological evaluation of N-aryl salicylamides with a hydroxamic acid moiety at 5-position as novel HDAC-EGFR dual inhibitors. Bioorg Med Chem 2012;20:4405–4412. 22698782 10.1016/j.bmc.2012.05.034

[28] G. Hoelzemann, D. Dorsch, H. Greiner, C. Amendt, F. Zenke, Alkoxy-thienopyrimidines as tgf-beta receptor kinase modulators, in, Google Patents, 2010.

[29] Voynikov Y, Stavrakov G, Tencheva J, Peikov P. Novel Leucine Derived Amides of Theophylline-7-acetic Acid. Comptes Rendus de L'Académie Bulgare Des Sciences: sciences Mathématiques et Naturelles 2013;66:1399.

[30] Ghith A, Youssef KM, Ismail NSM, Abouzid KAM. Design, synthesis and molecular modeling study of certain VEGFR-2 inhibitors based on thienopyrimidne scaffold as cancer targeting agents. Bioorg Chem 2019;83:111–128. 30343204 10.1016/j.bioorg.2018.10.008

[31] Zhang Y, Chen Y, Zhang D, et al. Discovery of novel potent VEGFR-2 inhibitors exerting significant antiproliferative activity against cancer cell lines. J Med Chem 2018;61:140–157. 29189002 10.1021/acs.jmedchem.7b01091

[32] Liu Z, Qi L, Li Y, et al. VEGFR2 regulates endothelial differentiation of colon cancer cells. BMC Cancer 2017;17:593. 28854900 10.1186/s12885-017-3578-9 PMC5577671

[33] Kris MG, Natale RB, Herbst RS, et al. Efficacy of gefitinib, an inhibitor of the epidermal growth factor receptor tyrosine kinase, in symptomatic patients with non-small cell lung cancer: a randomized trial. JAMA 2003;290:2149–2158. 14570950 10.1001/jama.290.16.2149

[34] Badalian G, Derecskei K, Szendroi A, et al. EGFR and VEGFR2 protein expressions in bone metastases of clear cell renal cancer. Anticancer Res 2007;27:889–894. 17465216

[35] Barker FG, 2nd, Simmons ML, Chang SM, et al. EGFR overexpression and radiation response in glioblastoma multiforme. Int J Radiation Oncol Biol Phys 2001;51:410–418. 10.1016/s0360-3016(01)01609-1 11567815

[36] Park JH, Liu Y, Lemmon MA, Radhakrishnan R. Erlotinib binds both inactive and active conformations of the EGFR tyrosine kinase domain. Biochem J 2012;448:417–423. 23101586 10.1042/BJ20121513 PMC3507260

[37] Sobhy MK, Mowafy S, Lasheen DS, et al. 3D-QSAR pharmacophore modelling, virtual screening and docking studies for lead discovery of a novel scaffold for VEGFR 2 inhibitors: Design, synthesis and biological evaluation. Bioorg Chem 2019;89:102988. 31146197 10.1016/j.bioorg.2019.102988

[38] Aziz MA, Serya RA, Lasheen DS, et al. Discovery of potent VEGFR-2 inhibitors based on furopyrimidine and thienopyrimidne scaffolds as cancer targeting agents. Scientific Rep 2016;6:24460. 10.1038/srep24460 PMC4832243 27080011

[39] Miyake Y, Keusch JJ, Wang L, et al. Structural insights into HDAC6 tubulin deacetylation and its selective inhibition. Nature Chem Biol 2016;12:748–754. 27454931 10.1038/nchembio.2140

[40] Alsawalha M, Rao Bolla S, Kandakatla N, et al. Molecular docking and ADMET analysis of hydroxamic acids as HDAC2 inhibitors. Bioinformation 2019;15:380–387. 31312074 10.6026/97320630015380 PMC6614126

[41] Palm K, Stenberg P, Luthman K, Artursson P. Polar molecular surface properties predict the intestinal absorption of drugs in humans. Pharmaceut Res 1997;14:568–571. 10.1023/a:1012188625088 9165525

[42] Egan WJ, Merz KM, Jr., Baldwin JJ. Prediction of drug absorption using multivariate statistics. J Med Chem 2000;43:3867–3877. 11052792 10.1021/jm000292e

[43] Hafez HN, El-Gazzar ABA. Design and synthesis of 3-pyrazolyl-thiophene, thieno[2,3-d]pyrimidines as new bioactive and pharmacological activities. Bioorg Med Chem Lett 2008;18:5222–5227. 18783947 10.1016/j.bmcl.2008.08.071

[44] Grinev AN, Kaplina NV. Transformations of 5-methyl-6-carbethoxy-3,4-dihydrothieno-[2,3-d]pyrimidine for synthesis of 4-methoxy-, 4-alkylamino-, and other derivatives of thieno[2,3-d]pyrimidine. Chem Heterocyclic Comp 1985;21:767–770.

[45] J. Das, J. Hynes, K. Leftheris, S. Lin, S. T. Wrobleski, H. Wu, Phenyl-aniline substituted bicyclic compounds useful as kinase inhibitors, in: US7419978, 2005.

[46] Reddy LS, Chandran SK, George S, et al. Crystal structures of N-Aryl-N′-4-nitrophenyl ureas: molecular conformation and weak interactions direct the strong hydrogen bond synthon. Crystal Growth Design 2007;7:2675–2690.

[47] Roice M, Christensen SF, Meldal M. ULTRAMINE: a high-capacity polyethylene-imine-based polymer and its application as a scavenger resin. Chemistry 2004;10:4407–4415. 15378618 10.1002/chem.200400314

[48] Uno M, Ban HS, Nabeyama W, Nakamura H. de novo design and synthesis of N-benzylanilines as new candidates for VEGFR tyrosine kinase inhibitors. Org Biomol Chem 2008;6:979–981. 18327319 10.1039/b719959g

[49] Shi L, Wu TT, Wang Z, et al. Discovery of quinazolin-4-amines bearing benzimidazole fragments as dual inhibitors of c-Met and VEGFR-2. Bioorg Med Chem 2014;22:4735–4744. 25082515 10.1016/j.bmc.2014.07.008

[50] K. Abouzid, E. Zaglol El-Razaz, R. T Serya, Thienopyrimidine compounds as tyrosine kinase inhibitors and anticancer agents, in: Egyptian patent office 545/2017, app date: 28-3-2017.

[51] Muthu K, Bariwal J, Vyas V, et al. Novel Dual Use of Formamide-POCl 3 Mixture for the Efficient, One-Pot Synthesis of Condensed 2 H -Pyrimidin-4-amine Libraries Under Microwave Irradiation. Syn Commun 2013;43:719–727.

[52] Kotaiah Y, Harikrishna N, Nagaraju K, Venkata Rao C. Synthesis and antioxidant activity of 1,3,4-oxadiazole tagged thieno[2,3-d]pyrimidine derivatives. Eur J Med Chem 2012;58:340–345. 23149297 10.1016/j.ejmech.2012.10.007

[53] *Scifinder*^n^, version 2020, Chemical Abstracts Service; RN 1029777-26-1 (accessed 7/7/2020).

[54] Alley MC, Scudiero DA, Monks A, et al. Feasibility of drug screening with panels of human tumor cell lines using a microculture tetrazolium assay. Cancer Res 1988;48:589–601. 3335022

[55] Shoemaker RH. The NCI60 human tumour cell line anticancer drug screen. Nature Rev Cancer 2006;6:813–823. 16990858 10.1038/nrc1951

