# Supplementary material for: Molecular design, synthesis and in vitro biological evaluation of thienopyrimidine–hydroxamic acids as chimeric kinase HDAC inhibitors: a challenging approach to combat cancer
Source: J Enzyme Inhib Med Chem. 2021 Jun 30;36(1):1290–312. doi: 10.1080/14756366.2021.1933465 (PMC8253220; doi:10.1080/14756366.2021.1933465)
Supplement: Supplemental Material [file IENZ_A_1933465_SM0468.pdf]

<u>Supplementary data tables and figures</u>

<u>List of tables and figures of SM:</u>

Table 1 of SM. Sixty human tumor NCI cell line anticancer screening data at single dose assay (10 µM concentrations) as percent cell growth promotion for the 20 synthesized compounds.

Table 2 of SM. Docking results of Erlotinib and representative synthesized thieno[2,3-d]pyrimidine derivatives against EGFR (PDB code: 4HJO),showing –CDOCKER energy score , hydrogen bonds and hydrophobic interactions along with their *in vitro* inhibitory activity against EGFR.

Table 3 of SM. Docking results of pyrrolo[3,2-*d*]pyrimidine reference compound and representative synthesized thieno[2,3-d]pyrimidine derivatives against VEGFR2 (PDB code 3VHE), showing –CDOCKER energy score , hydrogen bonds and hydrophobic interactions along with their *in vitro* inhibitory activity against VEGFR2

Table 4 of SM. Docking results of TSA reference compound and representative synthesized thieno[2,3-d]pyrimidine derivatives against HDAC6(PDB code: 3G0H), showing –CDOCKER energy score, hydrogen bonds and hydrophobic/ Pi-Pi interactions along with their *in vitro* inhibitory activity against HDAC6.

Figure 1 of SM. ADMET plot for the newly designed compounds*.*

Table 5 of SM. The computational parameters of pharmacokinetics (ADME) predictions for the synthesized thieno[2,3-d]pyrimidine hydroxamic acid derivatives.

Table 6 of SM. The AlogP98 and PSA parameters of pharmacokinetics (ADME) predictions for the synthesized thieno[2,3-d]pyrimidine hydroxamic acid derivatives versus their synthesized thieno[2,3-d]pyrimidine ester derivatives.

Table 1 of SM. Sixty human tumor NCI cell line anticancer screening data at single dose assay (10 μM concentrations) as percent cell growth promotion for the 20 synthesized compounds.

Cell line	***Cell Growth Percent for the tested compounds***																			
	**7a**	**7b**	**7c**	**7d**	**11a**	**11b**	**11c**	**12a**	**12b**	**12c**	**15a**	**15b**	**15c**	**15d**	**19b**	**19c**	**19d**	**20b**	**20c**	**20d**
Leukemia																				
**CCRF-CEM**	97.4	101.2	101.6	ND	106.8	105.0	98.8	107.0	103.5	107.8	110.7	98.7	103.4	ND	101.1	104.7	105.2	102.7	108.0	107.9
**HL-60(TB)**	102.2	103.1	102.0	102.7	103.5	103.9	105.0	101.4	102.4	104.7	101.2	103.0	104.9	101.9	103.7	104.5	102.8	104.1	103.4	104.6
**K-562**	105.9	108.5	91.7	103.0	108.3	115.1	103.6	97.8	101.6	103.8	101	94.9	105.0	105.3	101.9	106.4	113.3	104.9	108.0	117.4
**MOLT-4**	103.9	112.4	88.2	111.3	110.5	107.2	105.7	102.6	104.7	102.4	104	103.5	102.3	110.0	107.4	108.6	115.3	109.4	108.7	109.8
**RPMI-8226**	105.4	110.6	93.2	ND	109.4	110.5	102.2	110.5	106.6	104.0	112	106.0	104.3	ND	103.3	105.4	110.0	102.2	103.6	108.7
**SR**	99.1	115.8	94.0	110.3	108.7	112.2	101.0	105.3	108.2	103.3	106	78.2	102.7	101.4	104.5	101.3	110.8	99.2	102.6	110.0
Non-Small Cell Lung Cancer																				
**A549/ATCC**	103.8	101.8	100.4	101.3	97.7	96.2	103.7	99.6	105.8	107.3	99.5	98.3	105.8	100.7	98.3	107.5	104.3	98.8	104.4	94.0
**EKVX**	88.9	92.8	95.7	93.6	94.6	90.7	90.7	93.2	92.1	100.0	98.4	92.7	79.4	88.0	87.3	98.4	99.4	89.6	90.5	94.4
**HOP-62**	102.6	101.6	93.7	100.8	102.3	103.2	101.7	100.0	100.4	103.3	104.2	103.6	92.8	99.3	100.4	105.8	104.5	100.8	102.5	105.6
**HOP-92**	117.3	108.4	119.3	114.8	106.7	119.1	115.4	121.7	112.3	130.0	118.2	99.8	101.5	120.5	102.5	124.7	117.7	110.5	111.8	118.7
**NCI-H226**	99.4	94.5	99.2	103.8	90.4	87.7	97.9	93.3	96.6	105.6	99.3	89.3	85.6	103.2	89.4	106.8	104.0	97.5	92.0	88.7
**NCI-H23**	101.1	98.8	88.9	96.5	96.1	96.6	100.0	89.2	97.5	104.6	92.2	90.5	91.8	95.7	95.7	102.7	100.6	99.6	94.9	98.9
**NCI-322M**	98.4	103.7	99.6	107.3	101.2	100.7	103.7	99.7	95.9	105.0	99.4	101.8	97.2	107.6	99.0	105.7	108.1	107.3	101.3	105.2
**NCI-H460**	100.3	100.7	101.7	102.0	101.8	101.9	99.4	102.6	99.1	101.9	102.7	101.6	98.9	101.5	100.8	100.4	100.4	99.4	101.9	101.8
**NCI-H522**	92.0	90.2	88.3	92.1	92.8	94.6	94.3	87.7	81.7	94.1	94.4	86.6	74.5	88.1	90.5	90.0	95.5	89.7	91.2	92.6
Colon Cancer																				
**COLO 205**	106.8	114.4	103.3	112.9	113.5	113.1	112.9	111.9	111.3	114.9	112.8	115.6	116.7	112.3	110.7	115.7	117.3	106.5	117.8	119.1
**HCC-2998**	99.3	103.6	112.6	99.8	98.0	103.8	111.5	102.1	108.5	113.6	100.9	99.9	115.4	102.4	97.7	105.3	104.7	104.7	102.4	104.1
**HCT-116**	93.8	102.7	97.9	105.2	102.9	101.6	107.9	99.5	99.2	107.2	103.9	102.9	100.9	106.8	105.6	102.1	107.3	104.9	108.2	105.9
**HCT-15**	99.1	99.0	97.7	95.3	95.7	99.0	101.2	100.2	99.6	99.9	101.1	91.3	101.7	96.0	95.0	99.7	101.5	100.2	99.0	99.6
**HT29**	94.7	103.6	97.2	102.7	104.0	104.4	109.8	104.7	95.3	104.0	103.9	100.5	101.8	102.5	104.9	102.2	104.1	102.3	108.2	107.1
**KM12**	94.8	102.7	66.3	102.1	103.2	99.5	100.1	99.6	101.3	102.1	101.1	102.0	100.9	102.3	101.9	102.0	103.8	101.4	101.7	101.5
**SW-620**	98.4	101.2	108.7	104.8	103.0	103.0	104.3	103.3	101.5	109.4	103.6	89.5	102.6	108.2	99.6	102.5	101.5	102.0	102.3	103.1
CNS Cancer																				
**SF-268**	91.7	93.3	89.2	98.1	96.8	94.3	93.3	92.5	95.1	98.7	93.9	91.8	94.8	96.5	97.0	97.6	96.4	97.1	97.2	95.2
**SF-295**	96.2	94.5	95.1	98.3	95.2	100.0	91.8	102.0	102.5	93.7	103.7	91.9	102.7	93.7	91.8	99.7	96.0	90.6	101.1	101.9
**SF-539**	96.0	97.2	61.4	100.3	90.3	98.0	78.9	96.5	98.2	71.5	102.5	92.8	104.0	98.5	93.6	99.0	98.0	96.9	95.3	100.8
**SNB-19**	100.2	100.1	90.8	96.4	97.5	98.1	93.6	97.1	97.9	96.8	98.9	93.0	94.8	91.8	99.8	103.7	98.9	97.5	99.1	98.1
**SNB-75**	75.5	92.7	54.0	87.9	79.7	83.7	78.8	78.1	90.6	70.7	81.0	74.2	79.5	99.2	81.4	91.7	97.5	95.6	79.1	90.4
**U251**	89.3	101.4	73.1	104.0	96.9	97.1	80.2	92.3	92.8	87.3	96.7	90.0	100.9	98.2	101.5	104.8	102.2	98.2	103.1	99.1
Melanoma																				
**LOX IMVI**	90.5	94.1	90.9	93.7	95.3	91.6	94.5	93.5	90.7	98.1	97.5	96.0	95.8	101.3	93.7	98.8	98.8	96.5	99.2	96.3
**MALME-3M**	89.5	101.9	105.0	99.4	103.6	107.0	112.1	104.5	95.2	116.7	109.7	101.1	102.3	116.1	102.5	104.5	112.6	111.9	112.5	115.8
**M14**	95.1	101.0	102.4	104.3	99.1	92.9	107.0	99.0	104.0	107.2	106.5	100.4	107.8	110.2	106.1	107.9	104.7	112.3	107.6	102.6
**MDA-MB-435**	102.0	101.5	100.6	101.3	102.2	100.8	103.1	101.8	100.9	101.5	101.5	98.5	102.4	101.0	102.9	102.9	101.8	103.3	103.8	101.6
**SK-MEL-2**	107.8	100.6	108.5	104.1	107.0	116.4	108.7	113.3	94.6	107.8	115.8	109.3	94.5	100.9	108.6	101.8	100.6	102.9	106.2	113.2
**SK-MEL-28**	104.3	111.1	108.2	105.0	105.1	105.3	109.7	109.4	104.0	109.2	107.8	100.9	110.6	109.6	107.9	112.1	111.5	115.5	112.3	111.4
**SK-MEL-5**	101.0	99.5	99.6	101.2	99.1	95.8	97.1	97.8	97.3	100.9	101.5	99.0	96.9	100.5	94.4	102.4	101.8	97.6	100.0	100.4
**UACC-257**	115.5	100.8	106.3	99.4	101.0	99.1	108.3	104.2	104.3	107.0	112.4	107.0	110.0	109.9	105.0	115.4	99.7	103.9	112.8	103
**UACC-62**	101.7	96.7	96.4	94.9	98.7	95.3	99.6	99.1	95.3	98.5	94.2	92.5	92.4	88.5	95.6	101.7	102.3	96.8	98.8	93.7
Ovarian Cancer																				
**IGROV1**	103.6	106.5	97.6	111.5	103.0	104.1	102.2	99.0	97.0	116.7	97.9	87.7	72.7	103.9	101.8	105.4	106.2	107.3	81.1	98.9
**OVCAR-3**	105.4	106.5	104.2	111.0	102.6	108.9	111.6	107.1	99.3	117.8	106.1	96.9	98.9	108.5	115.3	110.3	106.5	109.4	106.5	108.4
**OVCAR-4**	94.1	101.8	103.4	104.6	101.4	91.0	105.0	100.6	99.9	110.1	111.9	94.7	106.3	102.2	104.9	112.3	108.8	105.4	109.4	104.8
**OVCAR-5**	105.3	102.8	114.3	102.8	106.2	101.9	104.4	105.1	107.5	111.3	107.0	98.4	107.6	101.3	102.0	110.3	108.4	100.2	109.8	100.1
**OVCAR-8**	106.3	96.6	100.4	103.1	101.0	99.2	102.2	104.6	103.6	105.9	103.6	104.4	102.0	105.0	99.4	104.1	101.3	98.7	105.5	99.6
**NCI/ADR-RES**	102.1	97.6	97.6	99.5	99.0	102.4	103.1	103.7	101.6	99.2	102.6	96.1	102.1	99.6	97.2	105.4	101.4	100.6	100.5	104.1
**SK-OV-3**	122.7	128.5	113.0	120.7	123.7	123.7	115.3	121.6	114.7	113.9	124.5	125.8	93.4	114.4	122.0	120.6	129.2	125.1	112.9	122.6
Renal Cancer																				
**786-0**	95.9	100.6	108.4	99.1	98.2	**101.3**	115.6	102.3	105.9	106.6	102.1	98.6	108.7	94.4	102.5	104.9	105.3	113.5	83.0	104.6
**ACHN**	102.3	103.7	110.8	105.4	101.6	106.0	101.7	95.4	98.6	105.9	99.1	95.0	97.3	97.6	106.3	106.8	102.1	103.8	105.1	102.2
**CAKI-1**	91.6	99.4	93.5	96.5	94.5	95.6	99.3	92.8	97.1	100.6	92.9	77.6	86.4	90.4	96.2	90.4	96.6	96.9	93.0	92.1
**RXF 393**	**110.2**	104.4	106.9	103.7	107.3	104.1	103.8	100.1	110.4	108.5	101.2	97.2	106.7	100.3	94.1	109.2	102.0	98.6	109.5	102.5
**SN12C**	102.8	99.1	93.2	100.8	101.4	105.1	100.0	99.0	104.5	104.6	102.0	92.8	98.6	100.1	98.5	101.7	102.7	100.3	100.0	103.5
**TK-10**	108.5	103.9	112.7	106.3	**97.6**	109.9	140.4	111.3	107.7	117.7	113.8	123.2	154.5	118.5	108.2	115.9	107.2	111.2	152.2	124.1
**UO-31**	74.6	83.4	75.7	85.0	83.7	83.4	87.0	75.3	82.0	89.6	80.6	72.7	71.2	77.0	86.4	79.9	84.8	88.2	81.5	76.3
Prostate Cancer																				
**PC-3**	97.3	101.6	91.1	105.6	101.7	102.0	100.2	98.9	98.6	111.1	104.3	100.1	97.6	110.4	105.1	110.4	103.5	108	101.4	105.3
**DU-145**	100.2	103.1	104.5	106.9	110.8	102.0	105.3	108.5	98.5	106.6	109.5	100.1	108.8	101.9	108.0	114.2	111.2	111	116.8	109.9
Breast Cancer																				
**MCF7**	90.7	95.2	88.4	95.2	92.3	90.5	89.2	92.4	91.5	95.2	95.2	87.2	88.6	93.8	92.3	93.4	95.1	92.1	91.4	91.4
**MDA-MB-231/ATCC**	91.1	99.5	95.2	94.9	97.6	100.5	105.2	92.1	97.0	99.1	102.4	100.3	88.1	97.2	101.7	104.1	102.5	110.9	95.4	99.7
**HS 578T**	97.7	102.4	90.3	104.5	99.6	100.7	93.5	106.3	99.2	93.9	106.4	104.7	102.5	102.4	103.8	105.8	103.1	102.2	99.4	99.9
**BT-549**	87.6	99.5	115.1	106.3	104.9	96.1	137.1	105.0	105.5	118.8	113.5	112.4	116.4	114.0	109.1	103.5	113.9	128.8	116.5	117.9
**T-47D**	95.7	99.6	96.3	112.9	107.7	100.1	101.4	103.9	97.6	109.7	108.3	90.8	90.6	95.4	101.1	106.9	106.5	104.4	105.2	112.6
**MDA-MB-468**	111.9	98.8	101.1	103.9	101.3	83.0	97.1	100.0	97.0	105.6	101.3	80.8	102.9	85.6	92.2	119.7	102.7	98.0	110.9	83.8

O 5-10% growth inhibition, **O** 10-20% growth inhibition, **O** 20-30% growth inhibition, **O** 30-40% growth inhibition, **O** 40-50% growth inhibition

Table 2 of SM. Docking results of Erlotinib and representative synthesized thieno[2,3-d]pyrimidine derivatives against EGFR (PDB code: 4HJO),showing –CDOCKER energy score , hydrogen bonds and hydrophobic interactions along with their *in vitro* inhibitory activity against EGFR.

Compound name	% inhibition	IC50	-CDocker energy	Hydrogen bonds	Hydrophobic and Pi-Pi interactions
Erlotinib	--------	---------	27.18	NH of MET769 - N of pyrimidine ring	ALA719, MET769, LEU820 – pyrimidine ring<br>LEU694, LEU820 – phenyl ring<br>VAL702, ALA719, LYS721 – terminal phenyl
15c	97%	19 nM	28.34	NH of MET769 - N of pyrimdine ring	ALA719, LEU820 – pyrimidine ring<br>LEU694, ALA719, LEU820 – thiophene ring<br>VAL702, ALA719, LYS721 – terminal phenyl ring<br>VAL702, LEU820 – CH_3 of thiophene<br>LEU764, LEU834 – Cl of terminal phenyl ring
20d	96%	68 nM	42.52	NH of MET769 - N of pyrimdine ring<br>HZ2 of LYS692 – O of OH<br>HZ2 of LYS692 – O of C=O of hydroxamic acid<br>O of VAL693 – O of OH<br>O of LEU694 – H of NH of hydroxamic acid	ALA719, LEU820 – pyrimidine ring<br>LEU694, LEU820 – thiophene ring<br>VAL702, ALA719, LYS721 – terminal phenyl ring<br>VAL702- CH_3 of thiophene<br>ALA719, LYS721 – Cl of terminal phenyl
15d	97%	--------	25.8	NH of MET769 - N of pyrimdine ring	ALA719, MET769, LEU820 – pyrimidine ring<br>LEU694, LEU820 – thiophene ring<br>VAL702, ALA719, LYS721 – terminal phenyl ring<br>VAL702- CH_3 of thiophene<br>ALA719, LYS721 – Cl of terminal phenyl

19d	96%	-------	39.92	HZ3 of LYS 692 - O of C=O of hydroxamic acid<br>NH of MET769 – O of C=O<br>HZ2 of LYS704 – O of C=O of hydroxamic acid<br>HZ3 of LYS704 – O of OH<br>O of PRO770- NH of hydroxamic acid	VAL702, LEU820 } pyrimidine ring<br>LEU694, VAL702, LEU820 } thiophene ring<br>VAL702, ALA719, LYS721 } terminal phenyl ring<br>ALA719, MET769, LEU820 } CH_3 of thiophene<br>LEU764, LYS721 } Cl of terminal phenyl ring
20b	92%	---------	42.99	NH of MET769 - N of pyrimidine ring<br>HG1 of THR766 - N of pyrimidine ring<br>HZ2 of LYS704 – O of C=O of hydroxamic acid<br>HZ3 of LYS692 – O of C=O of hydroxamic acid	ALA719, LEU796, LEU820 } pyrimidine ring<br>LEU694, ALA719, LEU820 } thiophene ring<br>VAL702, LYS721, ALA719 } terminal phenyl ring<br>VAL702, LEU694, LEU820 } CH_3 of thiophene
20c	91%	--------	45.91	NH of MET769 - N of pyrimidine ring<br>O of MET769 - H of NH of hydroxamic acid<br>O of MET769 - H of OH<br>O of PRO770 - H of NH of hydroxamic acid	ALA719, MET769, LEU820 } pyrimidine ring<br>LEU694, ALA719, LEU820 } thiophene ring<br>VAL702, LYS721, ALA719, LEU834 } terminal phenyl ring<br>VAL702, LEU694 } CH_3 of thiophene<br>LEU694, LEU764, LYS721 } Cl of terminal phenyl ring

12c	66%	1.14 µM	43.31	HG1 of THR766 – O of urea C=O<br>NH of CYS 773– O of C=O of hydroxamic acid<br>O of PHE771 – H of OH<br>NH of PHE832 – F of terminal phenyl ring<br>OD1 of ASP831 – H of urea NH	LEU694, LEU820 } pyrimidine ring<br>LEU694 - thiophene ring<br>VAL702, LEU820 } phenyl ring<br>LEU753, LEU764, LEU834, MET742 } terminal phenyl ring<br>LEU694– CH_3 of thiophene<br>CYS751 – C of CF_3

Table 3 of SM. Docking results of pyrrolo[3,2-*d*]pyrimidine reference compound and representative synthesized thieno[2,3-d]pyrimidine derivatives against VEGFR2 (PDB code 3VHE), showing –CDOCKER energy score , hydrogen bonds and hydrophobic interactions along with their *in vitro* inhibitory activity against VEGFR2.

Compound name	% inhibition	IC_{50}	-CDOCKER energy	Hydrogen bonds	Hydrophobic and Pi-Pi interactions
Reference	-----------	----------	42.91	NH of CYS919- N of pyrimidine ring<br>NH of ASP1026- O of urea C=O<br>OE2 of GLU885-H of urea NH<br>OE2 of GLU885- H of urea NH	LEU840, LEU1035, CYS919, ALA866, VAL848 } pyrole ring<br>CYS919, ALA866, VAL848, LEU1035 } Pyrimidine ring<br>VAL899, VAL916, VAL848, PHE1047, LYS868, CYS1045 } phenyl ring<br>LEU889 – terminal phenyl ring
7c	100%	19 1 nM	51.35	NH of CYS919- N of pyrimidine ring<br>HE2 of HIS1026-F of CF3 of terminal phenyl ring<br>NH of ASP1026- O of urea C=O<br>OE2 of GLU885-H of urea NH<br>OE2 of GLU885- H of urea NH	LEU840, LEU1035, CYS919, ALA866, VAL848 } Thiophene ring<br>CYS919, ALA866, VAL848, LEU1035 } Pyrimidine ring<br>VAL899, VAL916, VAL848, PHE1047, LYS868, CYS1045 } phenyl ring<br>Leu840, VAL848, PHE1047 } CH_3 of thiophene<br>HIS1026- CF_3 of terminal phenyl ring<br>LEU889 – terminal phenyl ring

12c	100%	185 nM	55.97	NH of CYS919- N of pyrimidine ring<br>NH of ASP1026- O of urea C=O<br>OE2 of GLU885-H of urea NH<br>OE2 of GLU885- H of urea NH	LEU840, LEU1035, ALA866, VAL848 } Thiophene ring<br>CYS919, ALA866, VAL848, LEU1035 } Pyrimidine ring<br>VAL916, VAL848, PHE1047, LYS868, CYS1045 } phenyl ring<br>Leu840, VAL848, PHE1047 } CH_3 of thiophene<br>HIS1026, LEU1019, ILEU892 } CF_3 of terminal phenyl ring<br>LEU889 – terminal phenyl ring
11c	91%	----------	52.66	NH of CYS919- N of pyrimidine ring<br>NH of ASP1026- O of urea C=O<br>OE2 of GLU885-H of urea NH<br>OE2 of GLU885- H of urea NH<br>O of LYS920- H of OH	LEU840, LEU1035, ALA866, VAL848 } Thiophene ring<br>CYS919, ALA866, VAL848, LEU1035 } Pyrimidine ring<br>VAL899, VAL848, PHE1047, LYS868, CYS1045 } phenyl ring<br>LEU840, VAL848, PHE1047 } CH_3 of thiophene<br>ILEU888- CF_3 of terminal phenyl ring<br>LEU889 – terminal phenyl ring
15c	87%	5.58 µM	30.81	NH of CYS919- N of pyrimidine ring	LEU840, LEU1035, ALA866 } Thiophene ring<br>CYS919, ALA866, VAL848, LEU1035 } Pyrimidine ring<br>VAL899, VAL848, VAL916, PHE1047, CYS1045, LYS868 } Phenyl ring<br>LEU840, VAL848, PHE1047 } CH_3 of thiophene<br>LEU889, VAL899, LYS868 } Cl of terminal phenyl ring

Table 4 of SM. Docking results of TSA reference compound and representative synthesized thieno[2,3-d]pyrimidine derivatives against HDAC6(PDB code: 3G0H), showing –CDOCKER energy score, hydrogen bonds and hydrophobic/ Pi-Pi interactions along with their *in vitro* inhibitory activity against HDAC6.

Compound name	% inhibition	-CDocker energy	Hydrogen bonds	Hydrophobic and Pi-Pi interactions	Coordinate bond with zinc /distance
TSA	-------	25.54	HE2 of HIS573 – O of OH	PHE643 – phenyl ring<br>PHE583 – CH_3<br>PHE583<br>PHE643 } CH_3	Zn – O of C=O / 2.24<br>Zn – O of OH / 2.20
20b	56%	34.32	HE2 of HIS573 – O of OH<br>HH of TYR745 – O of hydroxamic C=O<br>HE2 of HIS614 – O of amidic C=O	PHE643 – thiophene ring	Zn – O of C=O / 2.24<br>Zn – O of OH / 2.36
15b	30%	15.78	O of GLY582 – H of OH<br>HE2 of HIS614 – N of pyrimidine	PHE643 – pyrimidine ring<br>PHE583<br>PHE643 } thiophene<br>PHE712<br>PHE583<br>PHE643 } CH_3	None
12c	23%	37.58	HE2 of HIS573 – O of OH<br>HE2 of HIS614 – S of thiophene ring<br>O of GLY582 – H of hydroxamic NH	PHE642 – pyrimidine ring<br>PHE643 – thiophene ring	Zn – O of C=O / 2.11
19b	10%	37.68	OH of TYR745 – H of OH<br>HE2 of HIS573 – O of OH<br>HE2 of HIS614 – O of amidic C=O<br>O of GLY582 – H of hydroxamic NH	PHE643 – thiophene ring	Zn – O of C=O / 2.13

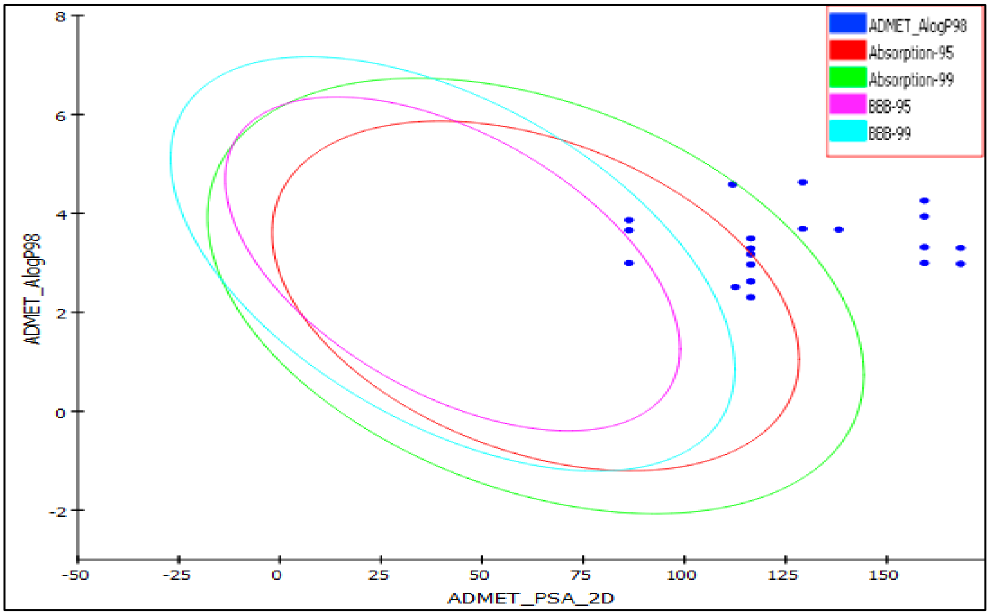

Figure 1 of SM. *ADMET plot for the newly designed compounds.*

Table 5 of SM. The computational parameters of pharmacokinetics (ADME) predictions for the synthesized thieno[2,3-d]pyrimidine hydroxamic acid derivatives.

Compound	ALOGP98	PSA_2D	Aqueous solubility level	BBB penetration level	Intestinal absorption level	Plasma protein binding	CYP2D6 binding
**7a**	3.68	129.17	2 (low)	4 (undefined)	2 (low)	True (highly bounded)	False (non-inhibitor)
**7b**	3.67	138.11	2 (low)	4 (undefined)	2 (low)	True (highly bounded)	False (non-inhibitor)
**7c**	4.62	129.17	1 (very low)	4 (undefined)	2 (low)	True (highly bounded)	False (non-inhibitor)
**7d**	4.58	111.87	1 (very low)	4 (undefined)	1 (moderate)	False (poorly bounded)	False (non-inhibitor)
**11a**	2.99	159.29	2 (low)	4 (undefined)	3 (very low)	True (highly bounded)	False (non-inhibitor)
**11b**	2.97	168.22	2 (low)	4 (undefined)	3 (very low)	False (poorly bounded)	False (non-inhibitor)
**11c**	3.93	159.29	1 (very low)	4 (undefined)	3 (very low)	True (highly bounded)	False (non-inhibitor)
**12a**	3.31	159.29	2 (low)	4 (undefined)	3 (very low)	False (poorly bounded)	False (non-inhibitor)
**12b**	3.30	168.22	2 (low)	4 (undefined)	3 (very low)	False (poorly bounded)	False (non-inhibitor)
**12c**	4.25	159.29	1 (very low)	4 (undefined)	3 (very low)	True (highly bounded)	False (non-inhibitor)
**15a**	2.50	112.57	2 (low)	4 (undefined)	0 (good)	False (poorly bounded)	False (non-inhibitor)
**15b**	2.99	86.25	2 (low)	3 (low)	0 (good)	True (highly bounded)	False (non-inhibitor)
**15c**	3.65	86.25	2 (low)	2 (moderate)	0 (good)	True (highly bounded)	False (non-inhibitor)
**15d**	3.86	86.25	2 (low)	2 (moderate)	0 (good)	True (highly bounded)	False (non-inhibitor)
**19b**	2.30	116.36	2 (low)	4 (undefined)	0 (good)	False (poorly bounded)	False (non-inhibitor)
**19c**	2.96	116.36	2 (low)	4 (undefined)	0 (good)	True (highly bounded)	False (non-inhibitor)
**19d**	3.17	116.36	2 (low)	4 (undefined)	0 (good)	True (highly bounded)	False (non-inhibitor)
**20b**	2.62	116.36	2 (low)	4 (undefined)	0 (good)	False (poorly bounded)	False (non-inhibitor)
**20c**	3.28	116.36	2 (low)	4 (undefined)	1 (moderate)	True (highly bounded)	False (non-inhibitor)
**20d**	3.49	116.36	2 (low)	4 (undefined)	1 (moderate)	True (highly bounded)	False (non-inhibitor)

;

Abbreviations: AlogP, the logarithm of the partition coefficient between n-octanol and water; PSA, polar surface area, CYP450 cytochrome P450, BBB blood brain barrier.

Table 6 of SM. The AlogP98 and PSA parameters of pharmacokinetics (ADME) predictions for the synthesized thieno[2,3-d]pyrimidine hydroxamic acid derivatives versus their synthesized thieno[2,3-d]pyrimidine ester derivatives.

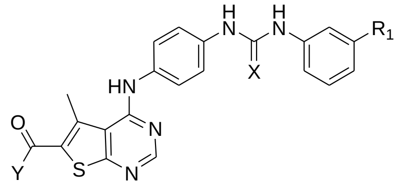

4a-d, 7a-c, 8a-c, 5a-d, 9a-c, 10a-c

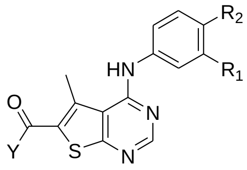

11a-d, 14b-d, 15b-d, 12a-d, 16b-d, 17b-d

Compound	Y	ALOGP98	PSA	Compound	Y	ALOGP98	PSA	X	R_1	R_2
**6a**	OC2H5	5.04	104.48	**7a**	NHOH	3.68	129.17	O	H	--
**6b**	OC2H5	5.02	113.41	**7b**	NHOH	3.67	138.11	O	OCH_3	--
**6c**	OC2H5	5.98	104.48	**7c**	NHOH	4.62	129.17	O	CF_3	--
**6d**	OC2H5	5.94	87.18	**7d**	NHOH	4.58	111.87	S	H	--
**9a**	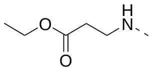	4.35	134.59	**11a**	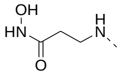	2.99	159.29	O	H	--
**9b**	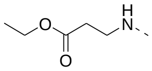	4.33	143.52	**11b**	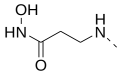	2.97	168.22	O	OCH_3	--
**9c**	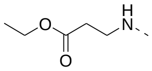	5.29	134.59	**11c**	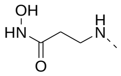	3.93	159.29	O	CF_3	--
**10a**	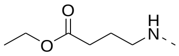	4.67	134.59	**12a**	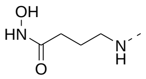	3.31	159.29	O	H	--
**10b**	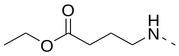	4.65	143.52	**12b**	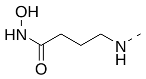	3.30	168.22	O	OCH_3	--
**10c**	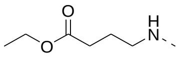	5.61	134.59	**12c**	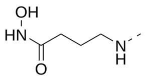	4.25	159.29	O	CF_3	--
**14a**	OC2H5	3.86	87.87	**15a**	NHOH	2.50	112.57	--	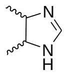	
**14b**	OC2H5	4.35	61.56	**15b**	NHOH	2.99	86.25	--	H	H
**14c**	OC2H5	5.01	61.56	**15c**	NHOH	3.65	86.25	--	H	Cl
**14d**	OC2H5	5.22	61.56	**15d**	NHOH	3.86	86.25	--	Cl	F
**17b**	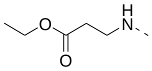	3.65	91.67	**19b**	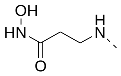	2.30	116.36	--	H	H
**17c**	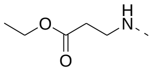	4.32	91.67	**19c**	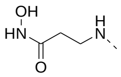	2.96	116.36	--	H	Cl
**17d**	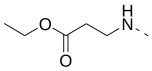	4.52	91.67	**19d**	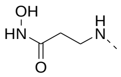	3.17	116.36	--	Cl	F
**18b**	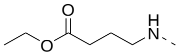	3.97	91.67	**20b**	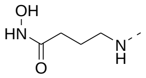	2.62	116.36	--	H	H
**18c**	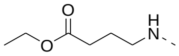	4.64	91.67	**20c**	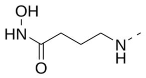	3.28	116.36	--	H	Cl
**18d**	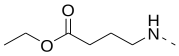	4.84	91.67	**20d**	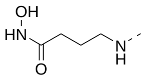	3.49	116.36	--	Cl	F

Abbreviations: AlogP, the logarithm of the partition coefficient between n-octanol and water; PSA, polar surface area,

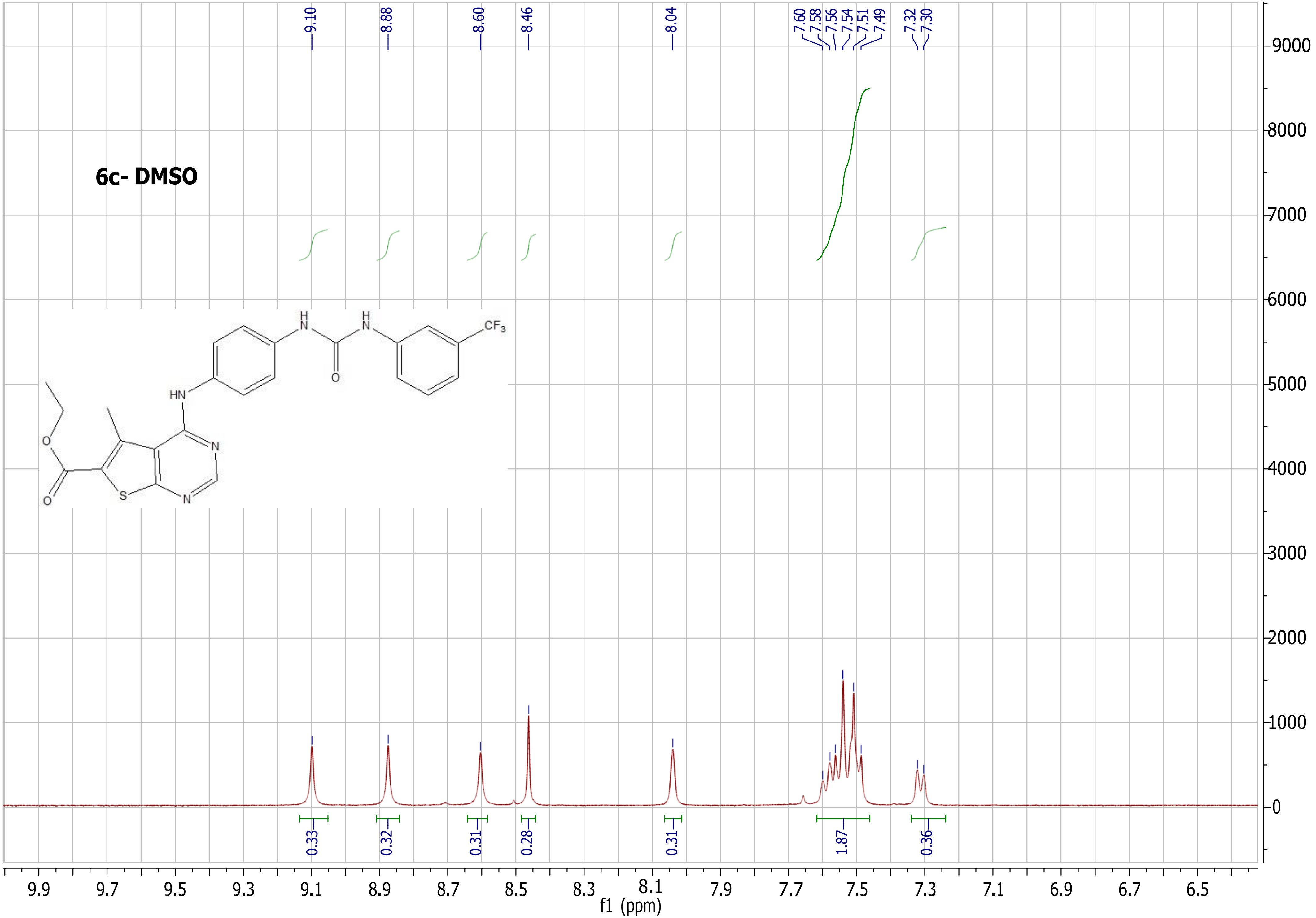
6c- DMSO
9.10
8.88
8.60
8.46
8.04
7.60
7.58
7.56
7.54
7.51
7.49
7.32
7.30
0.33
0.32
0.31
0.28
0.31
1.87
0.36
CF3
HN
O
S
N
f1 (ppm)

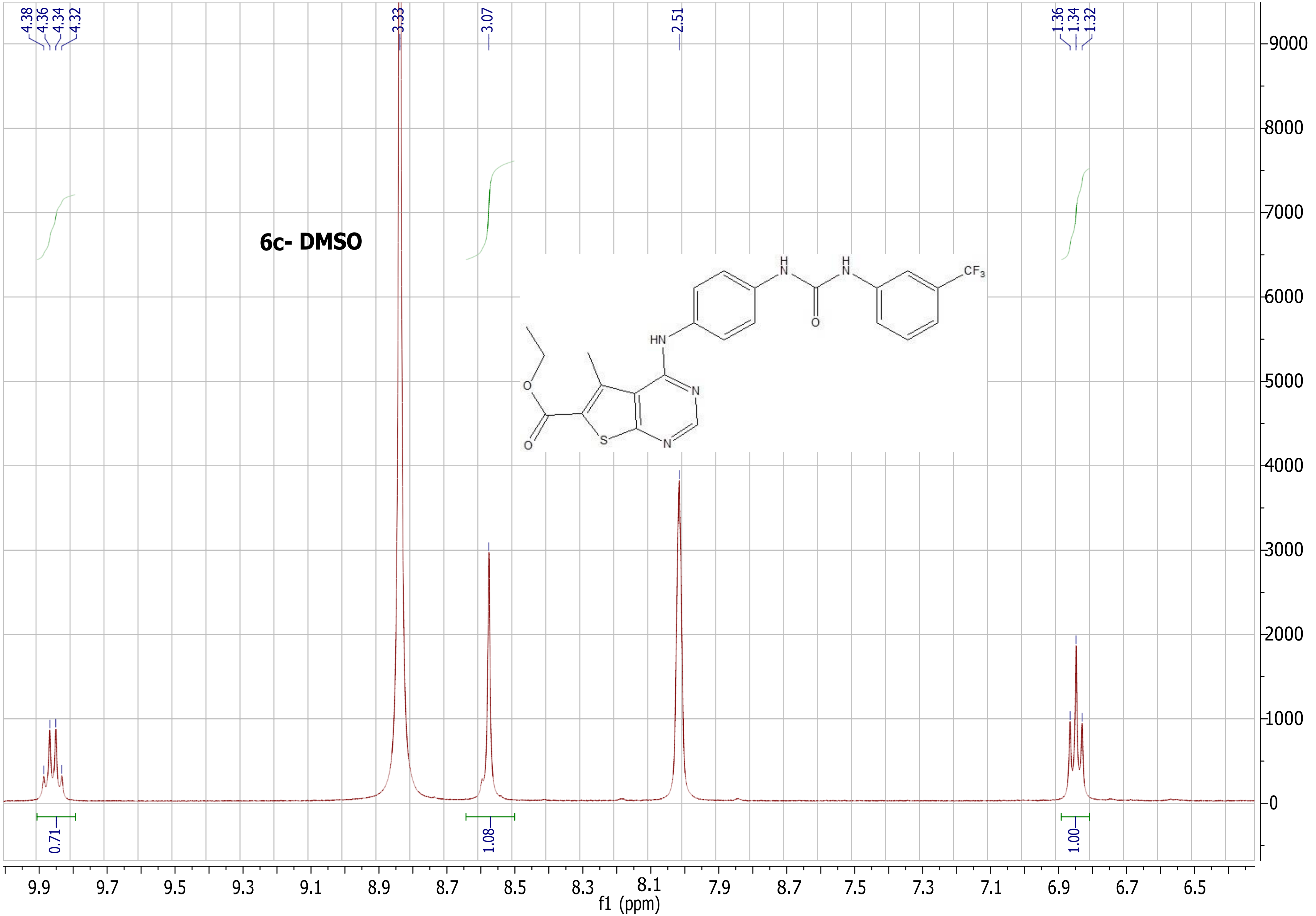

6c- DMSO
4.38
4.36
4.34
4.32
3.33
3.07
2.51
1.36
1.34
1.32
0.71
1.08
1.00
9.9
9.7
9.5
9.3
9.1
8.9
8.7
8.5
8.3
8.1
7.9
8.7
7.5
7.3
7.1
6.9
6.7
6.5
f1 (ppm)
9000
8000
7000
6000
5000
4000
3000
2000
1000
0
H
N
H
N
CF3
O
HN
O
N
O
S
N

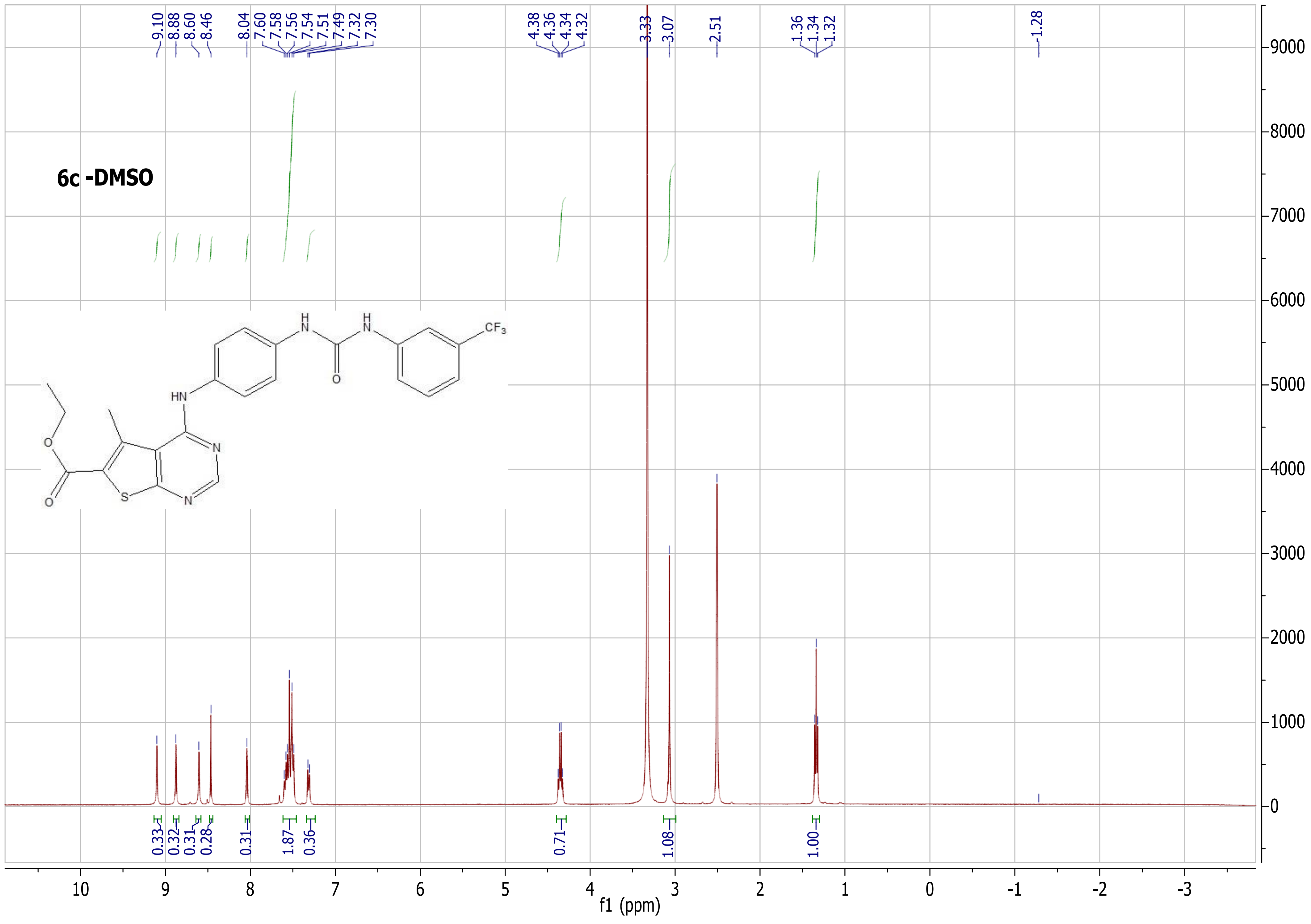
6c -DMSO
9.10
8.88
8.60
8.46
8.04
7.60
7.58
7.56
7.54
7.51
7.49
7.32
7.30
4.38
4.36
4.34
4.32
3.33
3.07
2.51
1.36
1.34
1.32
-1.28
0.33
0.32
0.31
0.28
0.31
1.87
0.36
0.71
1.08
1.00
HN
H
N
H
N
O
CF3
O
O
S
N
N
f1 (ppm)

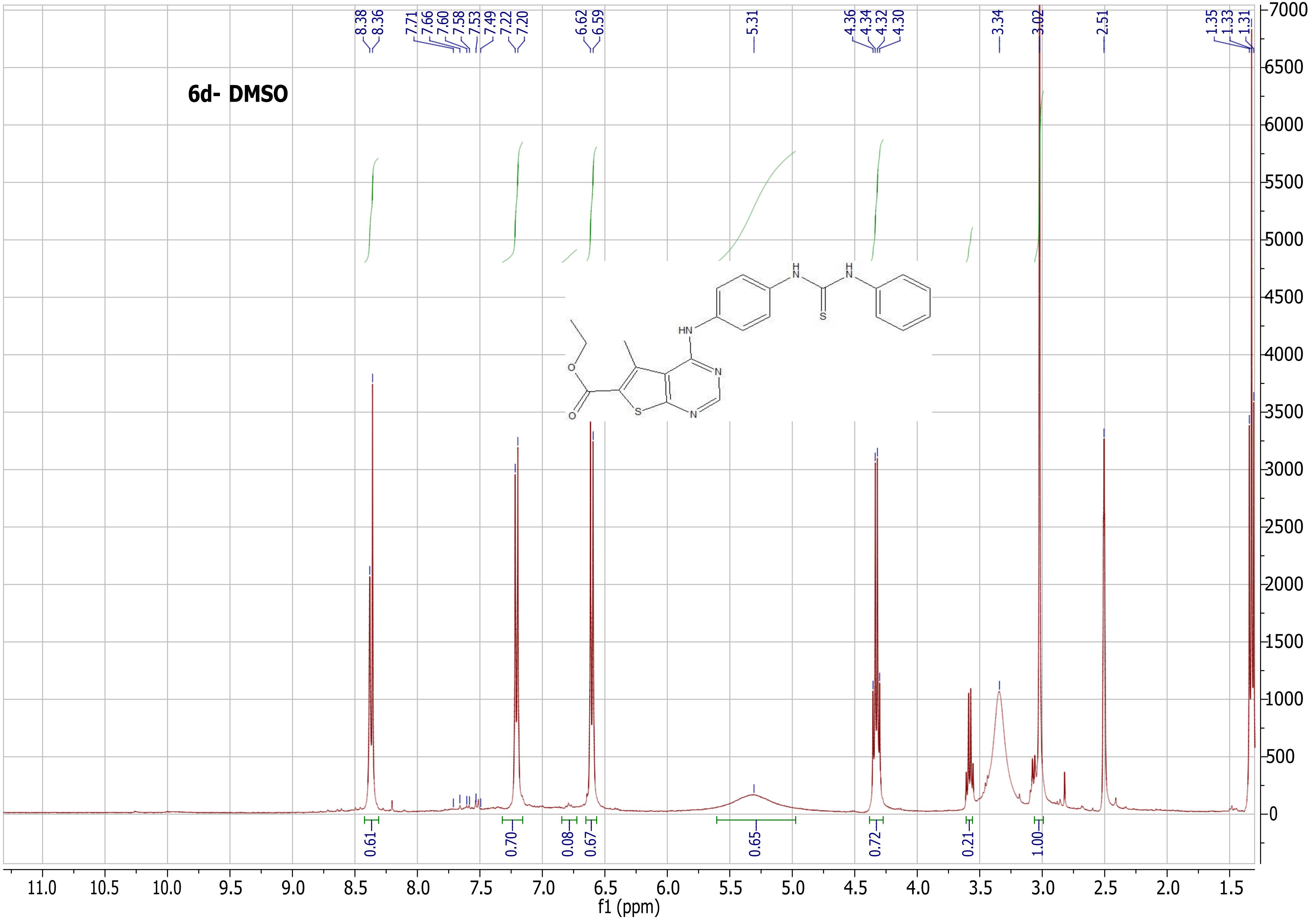
6d- DMSO
8.38
8.36
7.71
7.66
7.60
7.58
7.53
7.49
7.22
7.20
6.62
6.59
5.31
4.36
4.34
4.32
4.30
3.34
3.02
2.51
1.35
1.33
1.31
HN
H N
H N
S
N
O
O
S
N
0.61
0.70
0.08
0.67
0.65
0.72
0.21
1.00
f1 (ppm)

Compound 11a

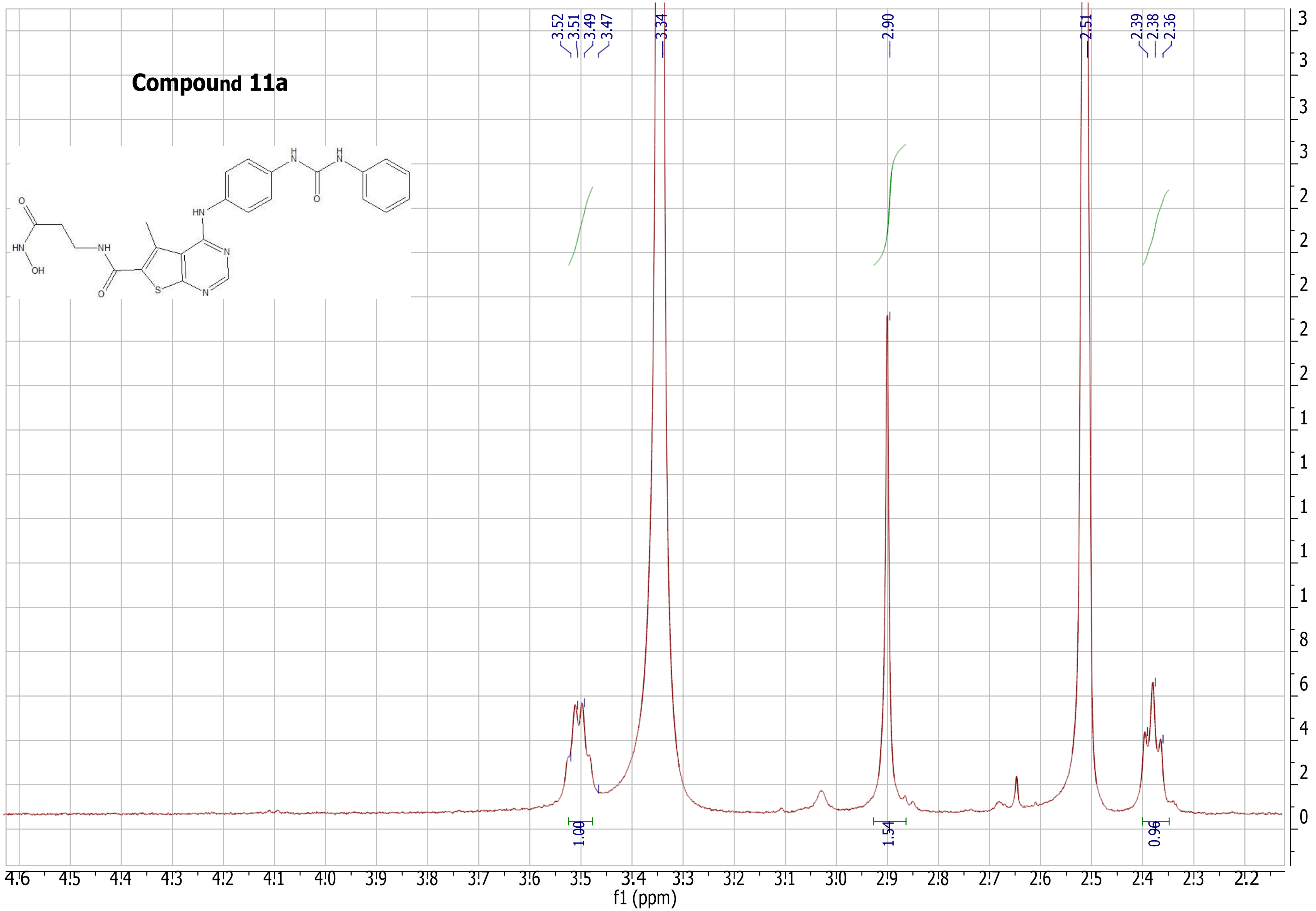

Compound 11a

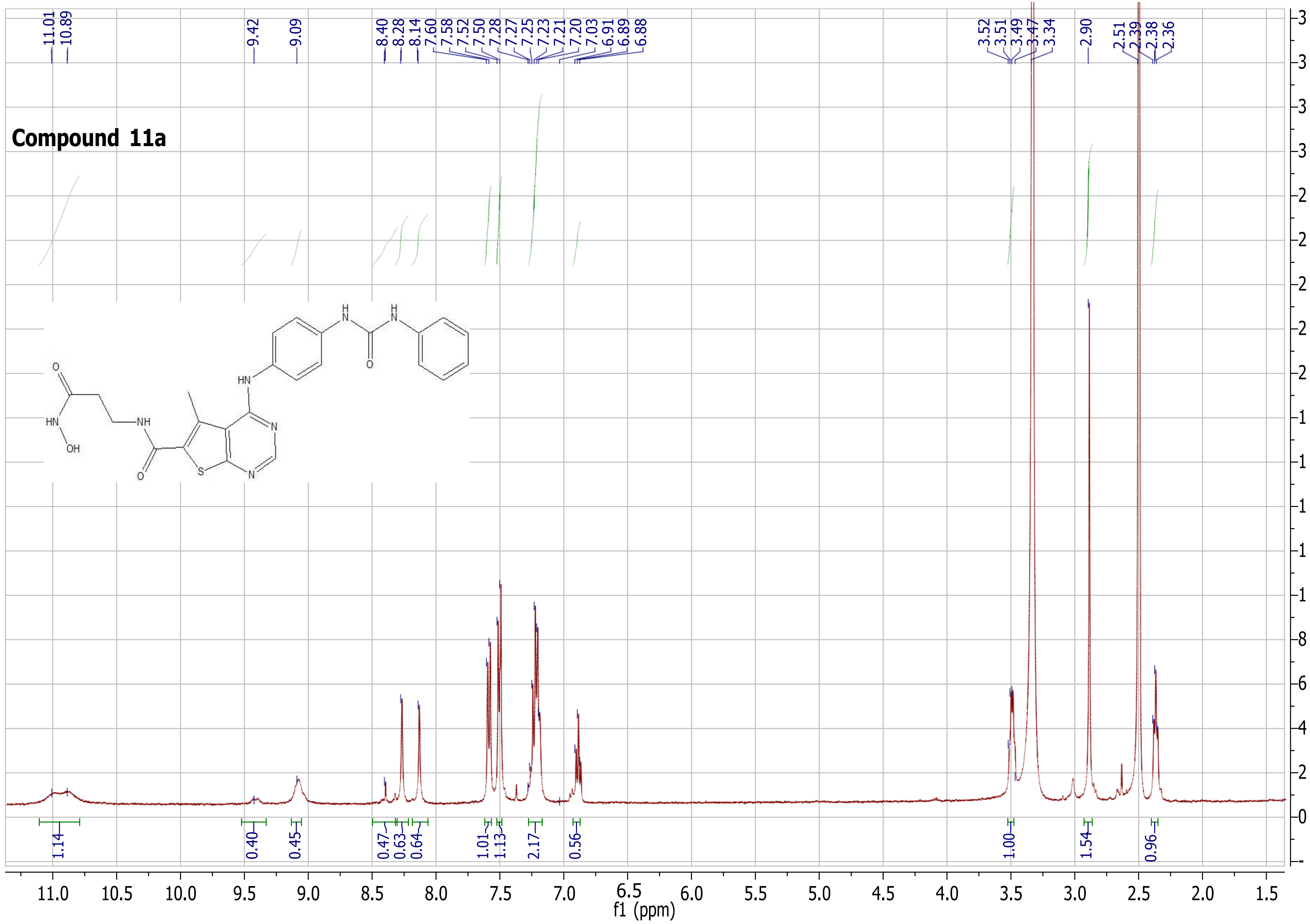

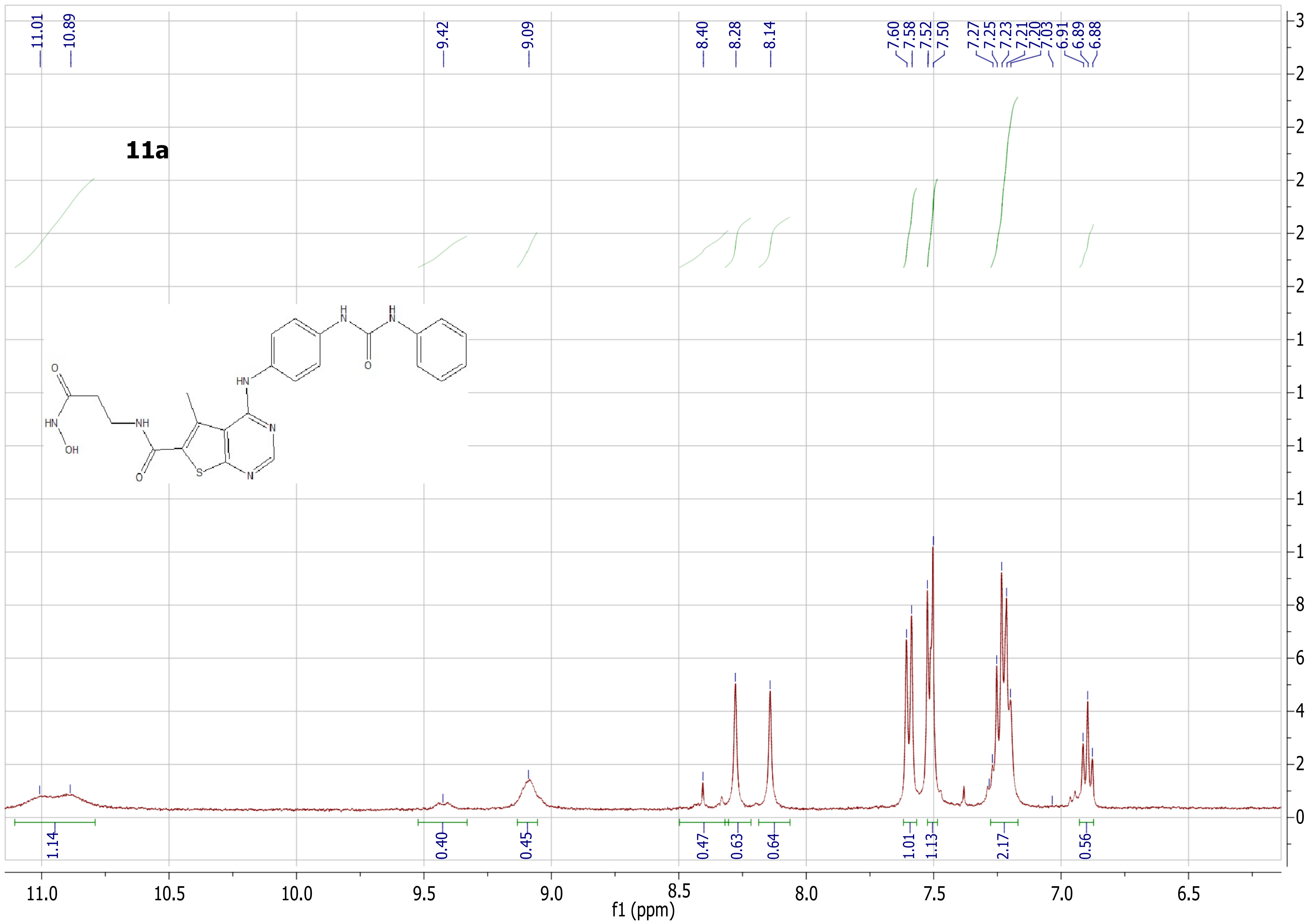
11a
11.01
10.89
9.42
9.09
8.40
8.28
8.14
7.60
7.58
7.52
7.50
7.27
7.25
7.23
7.21
7.20
7.03
6.91
6.89
6.88
1.14
0.40
0.45
0.47
0.63
0.64
1.01
1.13
2.17
0.56
f1 (ppm)

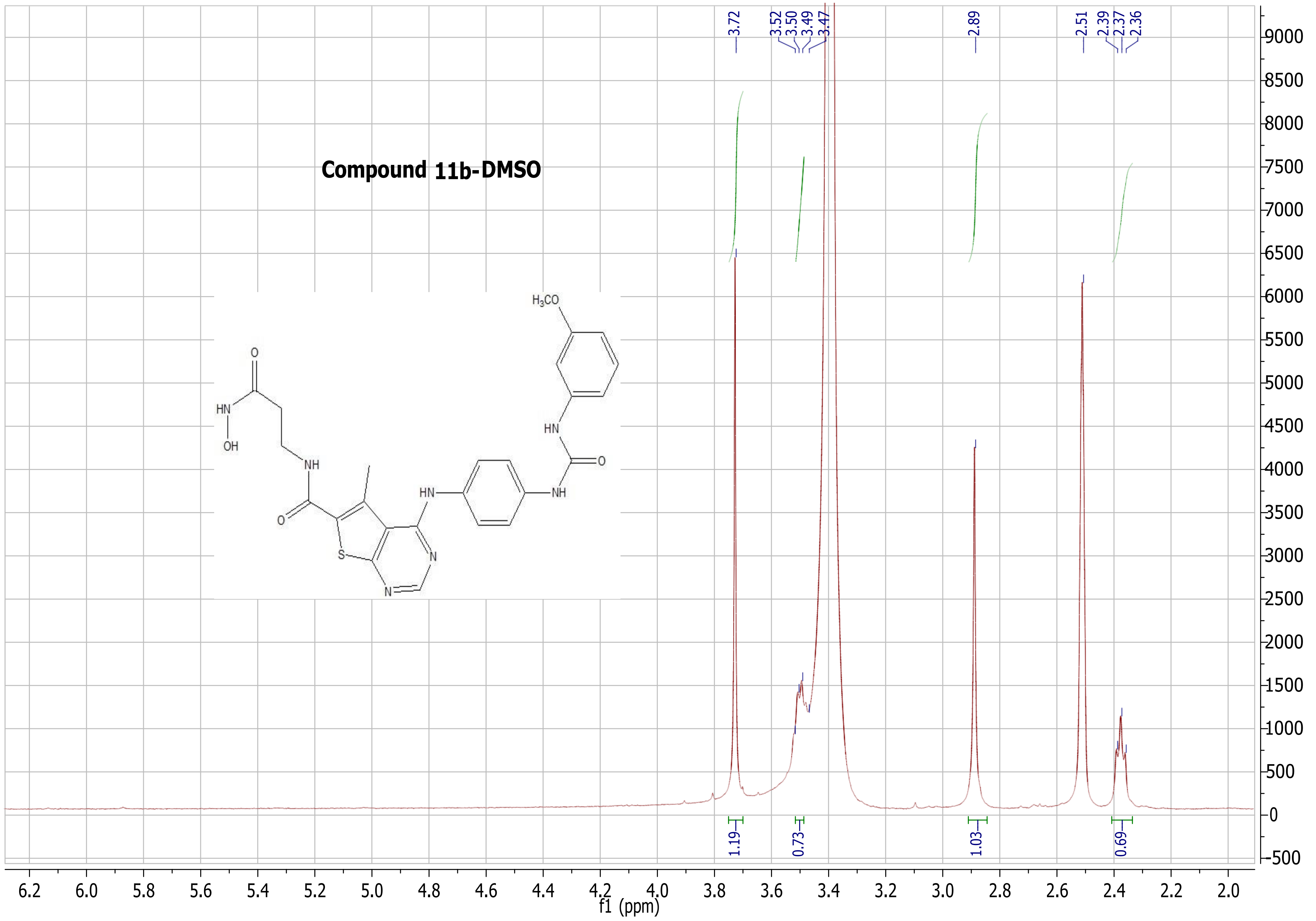

Compound 11b-DMSO
H3CO
HN
O
NH
OH
O
HN
NH
S
N
N
3.72
3.52
3.50
3.49
3.47
2.89
2.51
2.39
2.37
2.36
1.19
0.73
1.03
0.69
9000
8500
8000
7500
7000
6500
6000
5500
5000
4500
4000
3500
3000
2500
2000
1500
1000
500
0
-500
6.2
6.0
5.8
5.6
5.4
5.2
5.0
4.8
4.6
4.4
4.2
4.0
3.8
3.6
3.4
3.2
3.0
2.8
2.6
2.4
2.2
2.0
f1 (ppm)

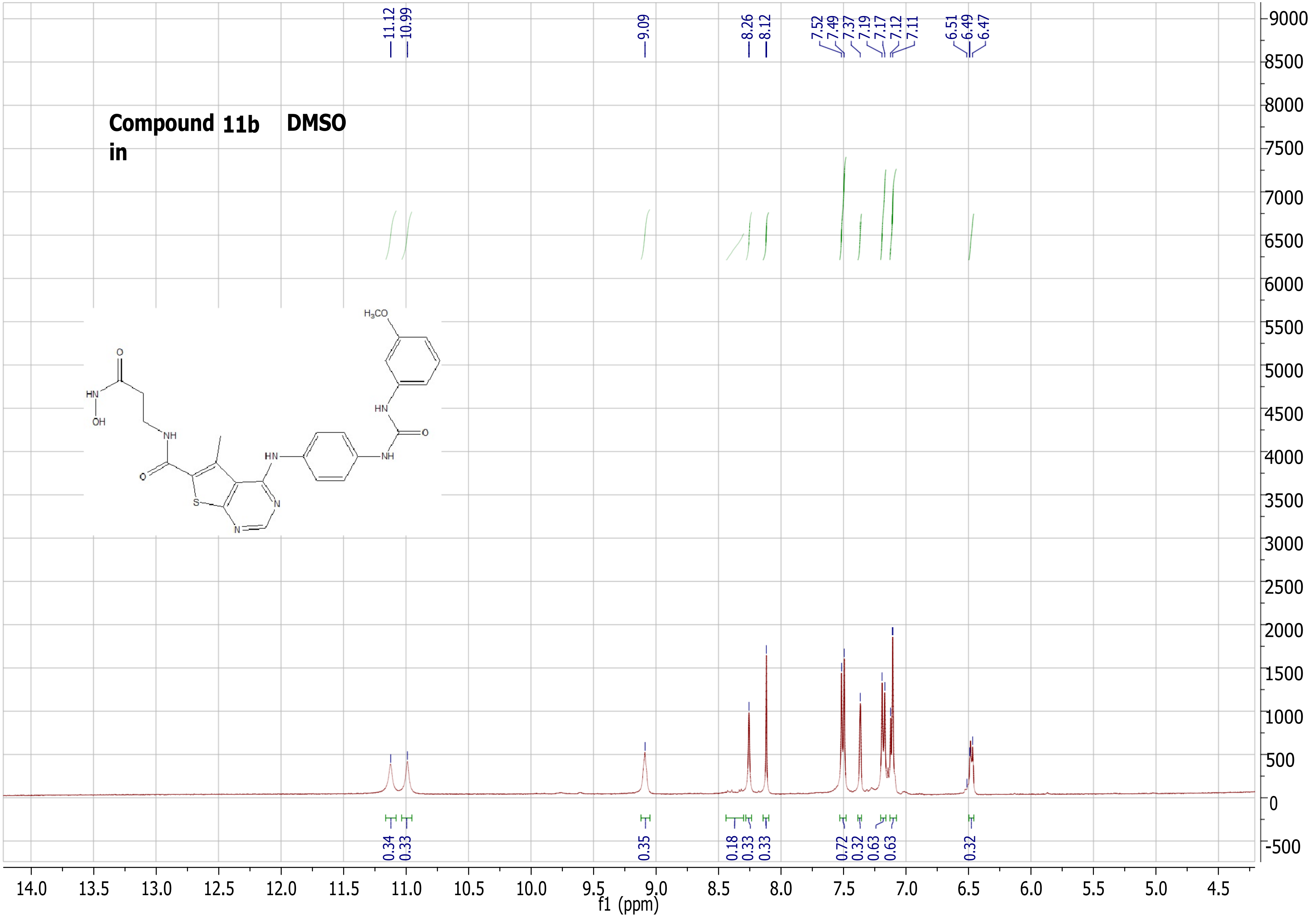

Compound 11b in DMSO
11.12
10.99
9.09
8.26
8.12
7.52
7.49
7.37
7.19
7.17
7.12
7.11
6.51
6.49
6.47
0.34
0.33
0.35
0.18
0.33
0.33
0.72
0.32
0.63
0.63
0.32
H3CO
HN
O
NH
HN
OH
NH
HN
S
N
N
9000
8500
8000
7500
7000
6500
6000
5500
5000
4500
4000
3500
3000
2500
2000
1500
1000
500
0
-500
14.0
13.5
13.0
12.5
12.0
11.5
11.0
10.5
10.0
9.5
9.0
8.5
8.0
7.5
7.0
6.5
6.0
5.5
5.0
4.5
f1 (ppm)

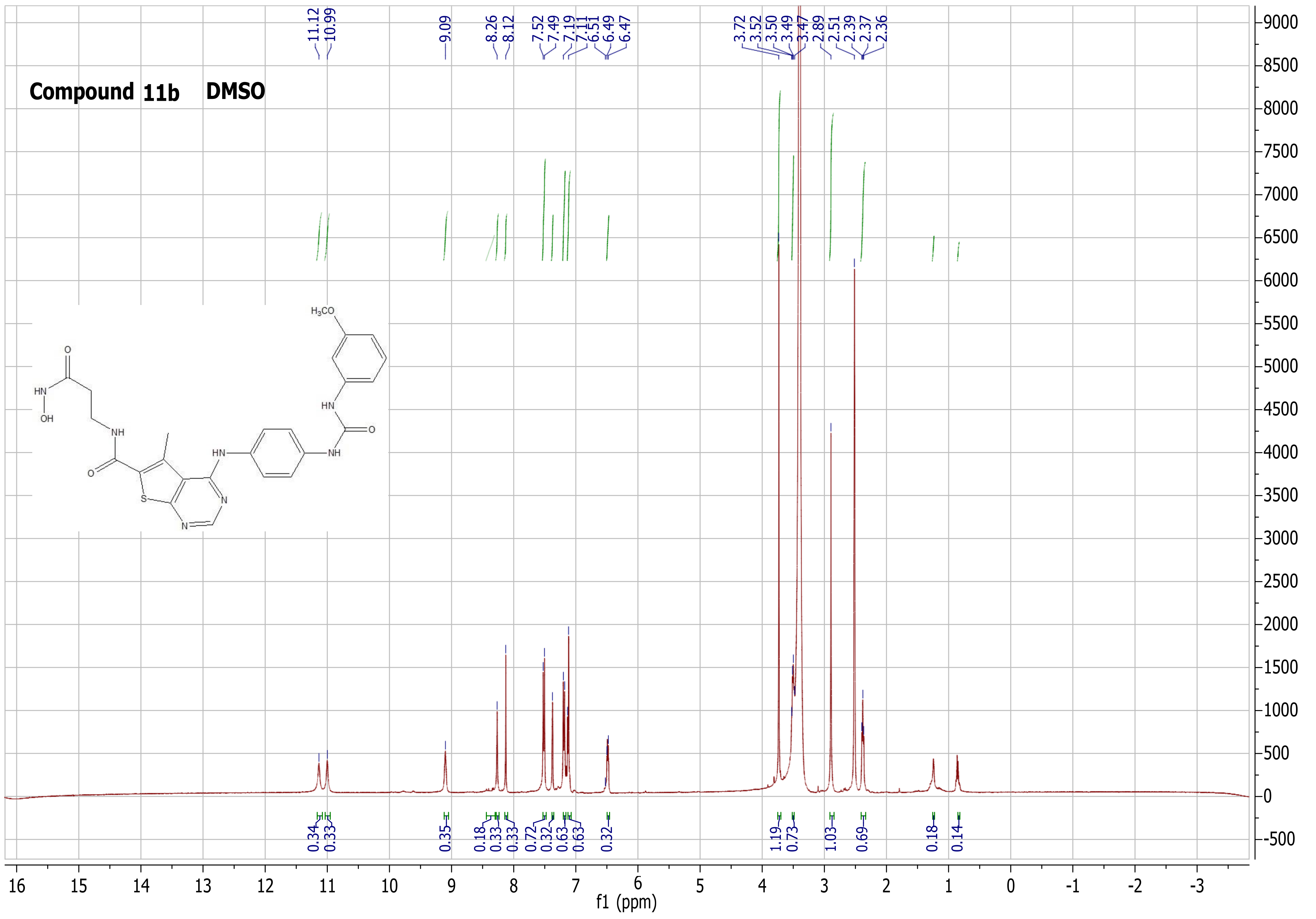
Compound 11b DMSO
11.12
10.99
9.09
8.26
8.12
7.52
7.49
7.19
7.11
6.51
6.49
6.47
3.72
3.52
3.50
3.49
3.47
2.89
2.51
2.39
2.37
2.36
0.34
0.33
0.35
0.18
0.33
0.33
0.72
0.32
0.63
0.63
0.32
1.19
0.73
1.03
0.69
0.18
0.14
f1 (ppm)

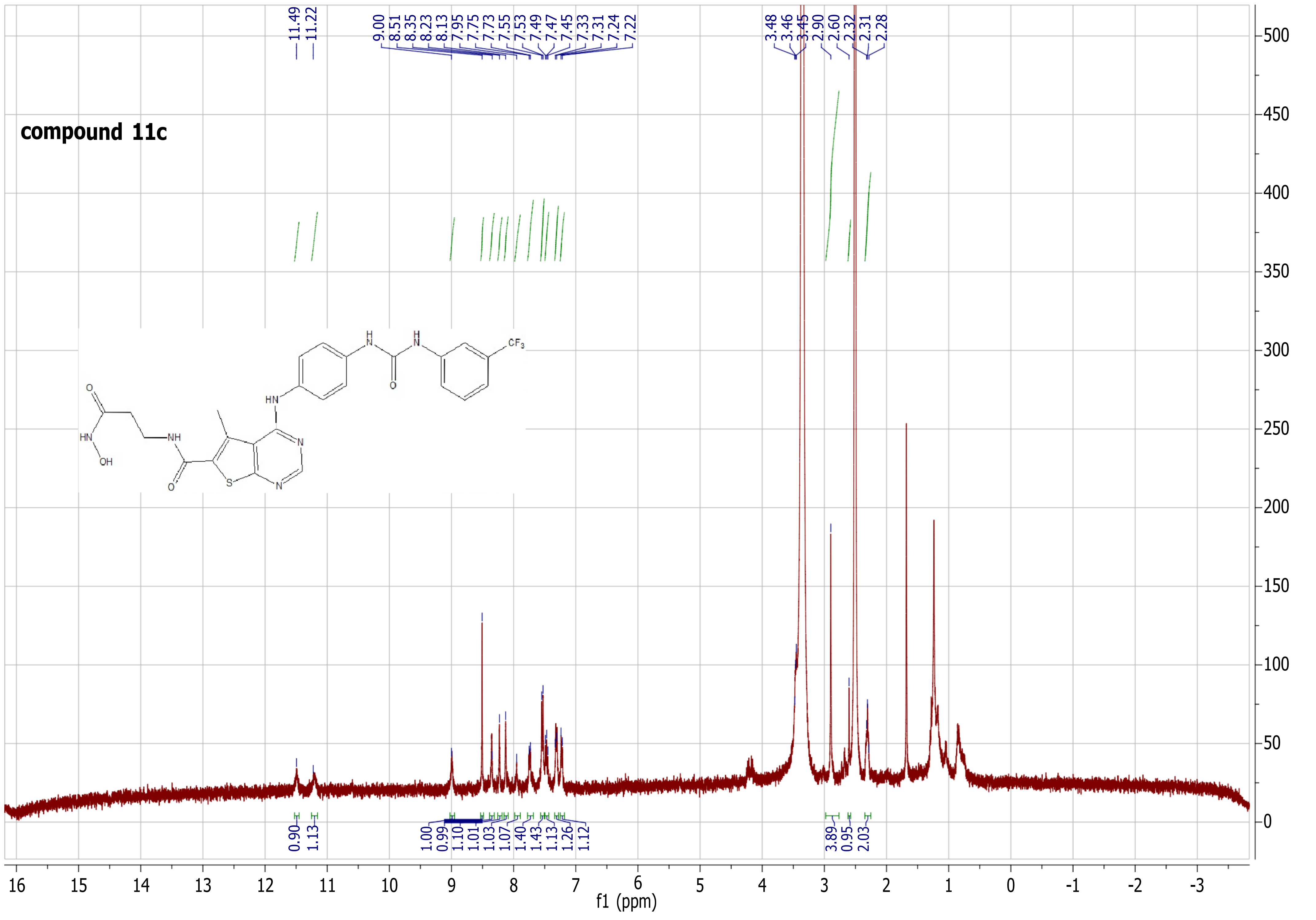
compound 11c
HN
OH
O
NH
O
S
N
N
HN
H
N
O
H
N
CF3
11.49
11.22
9.00
8.51
8.35
8.23
8.13
7.95
7.75
7.73
7.55
7.53
7.49
7.47
7.45
7.33
7.31
7.24
7.22
3.48
3.46
3.45
2.90
2.60
2.32
2.31
2.28
0.90
1.13
1.00
0.99
1.10
1.01
1.03
1.07
1.40
1.43
1.13
1.26
1.12
3.89
0.95
2.03
f1 (ppm)

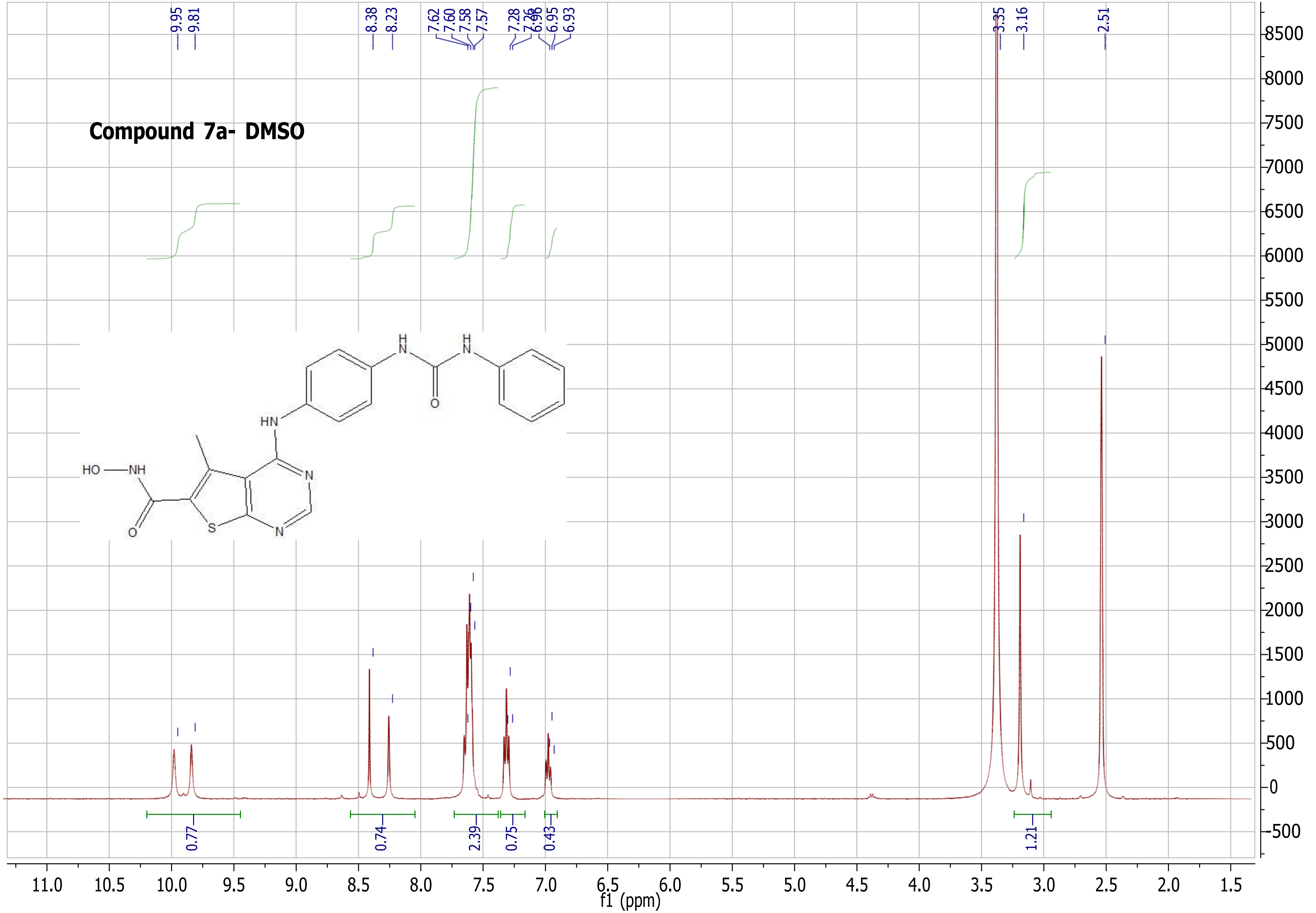
Compound 7a- DMSO
9.95
9.81
8.38
8.23
7.62
7.60
7.58
7.57
7.28
7.26
6.96
6.95
6.93
3.35
3.16
2.51
HO
NH
O
S
N
N
HN
H
N
H
N
O
0.77
0.74
2.39
0.75
0.43
1.21
f1 (ppm)

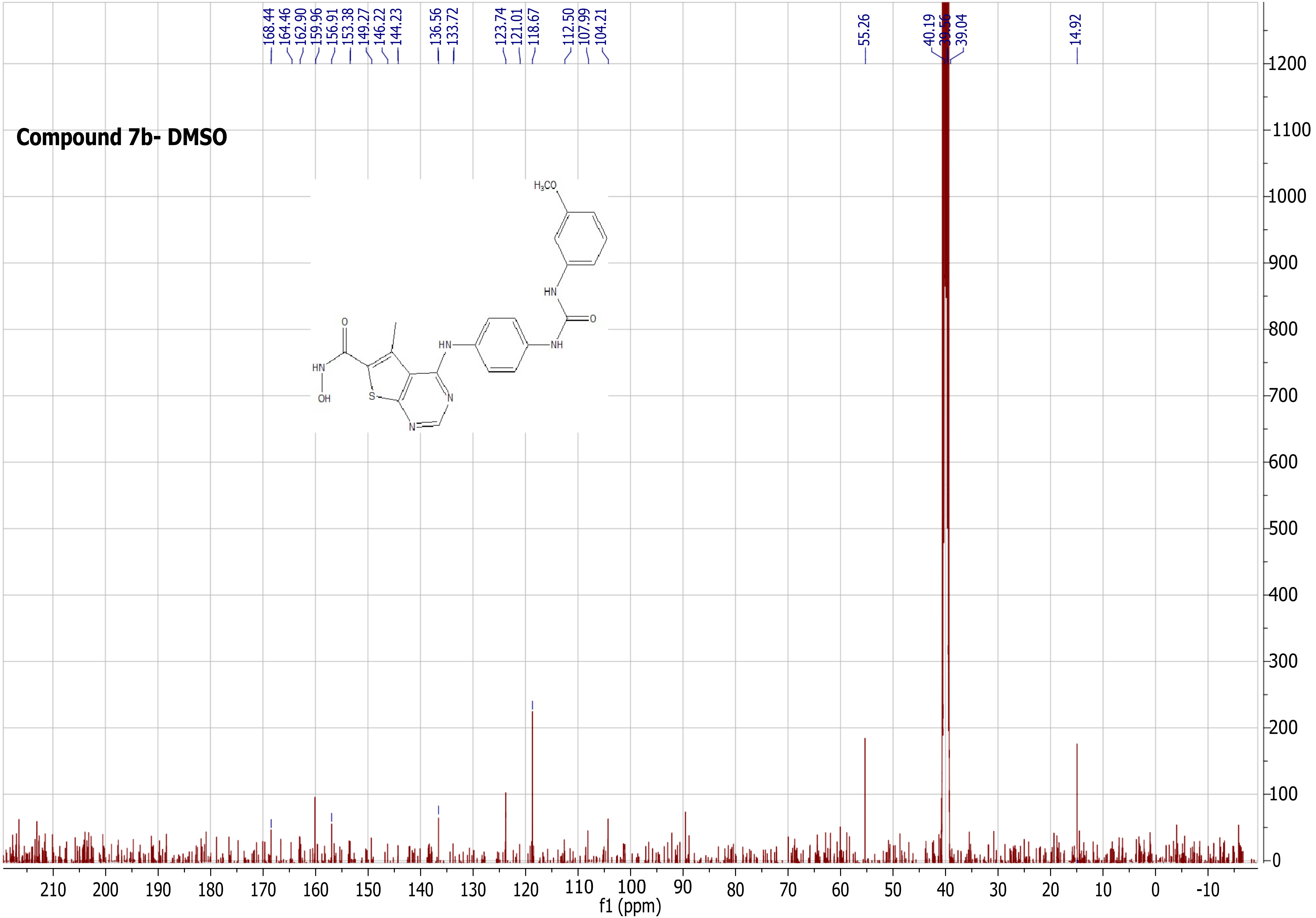

Compound 7b- DMSO
168.44
164.46
162.90
159.96
156.91
153.38
149.27
146.22
144.23
136.56
133.72
123.74
121.01
118.67
112.50
107.99
104.21
55.26
40.19
39.56
39.04
14.92
H3CO
HN
O
NH
HN
O
HN
OH
S
N
N
f1 (ppm)

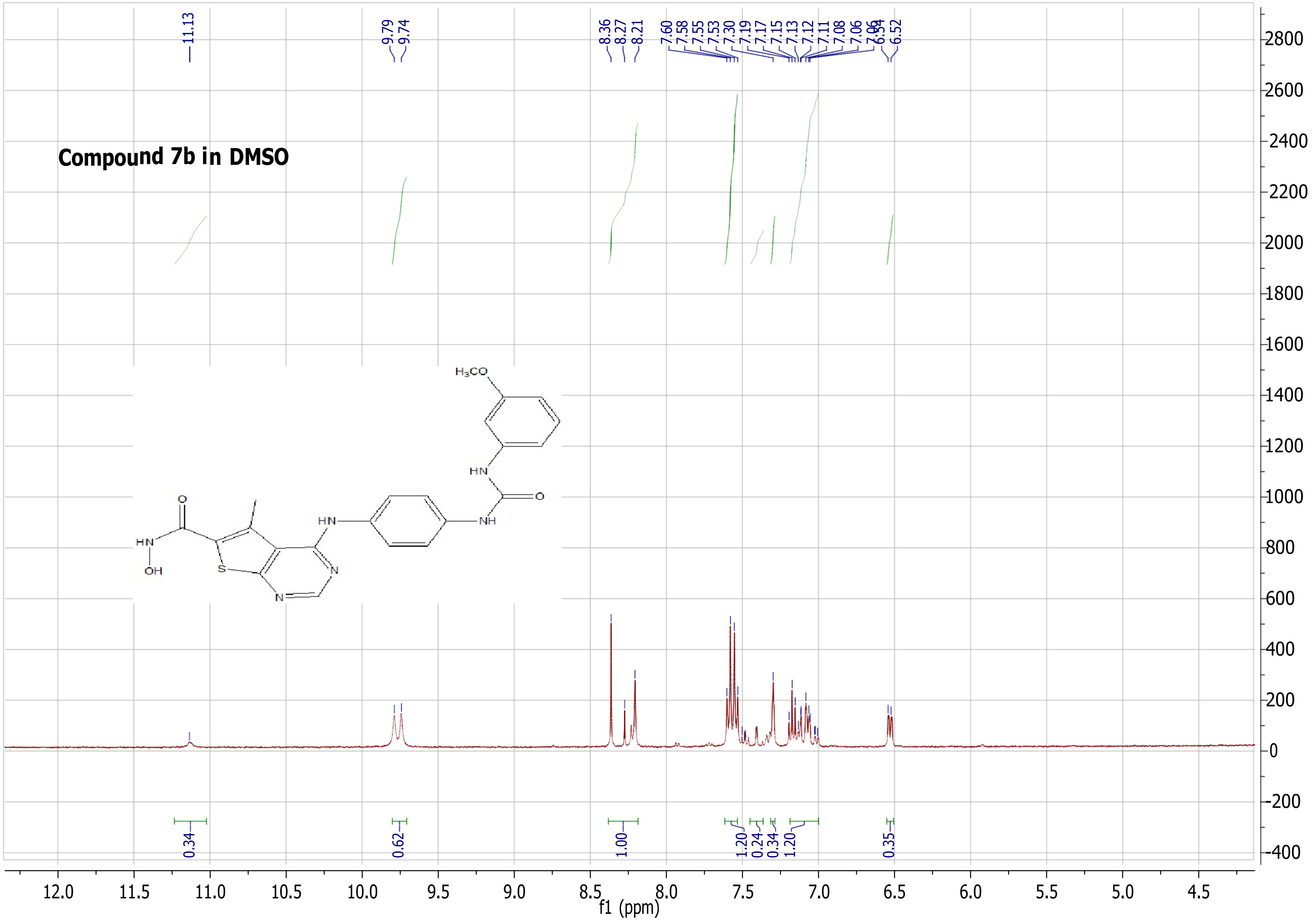

Compound 7b in DMSO
11.13
9.79
9.74
8.36
8.27
8.21
7.60
7.58
7.55
7.53
7.30
7.19
7.17
7.15
7.13
7.12
7.11
7.08
7.06
6.54
6.52
0.34
0.62
1.00
1.20
0.24
0.34
1.20
0.35
H3CO
HN
O
NH
HN
O
HN
OH
S
N
N
f1 (ppm)

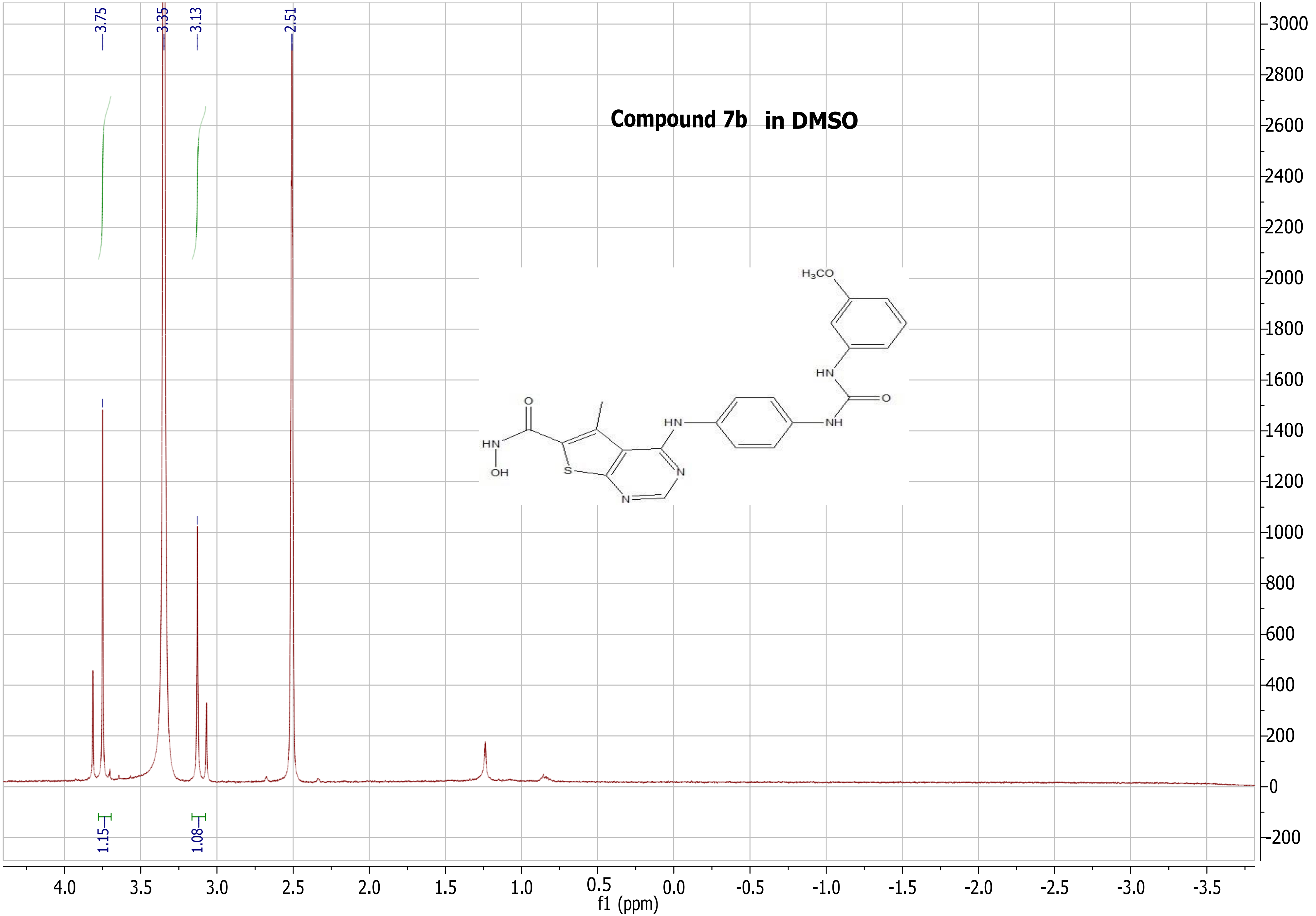

Compound 7b in DMSO
3.75
3.35
3.13
2.51
1.15
1.08
H3CO
HN
O
NH
HN
N
N
S
HN
OH
f1 (ppm)

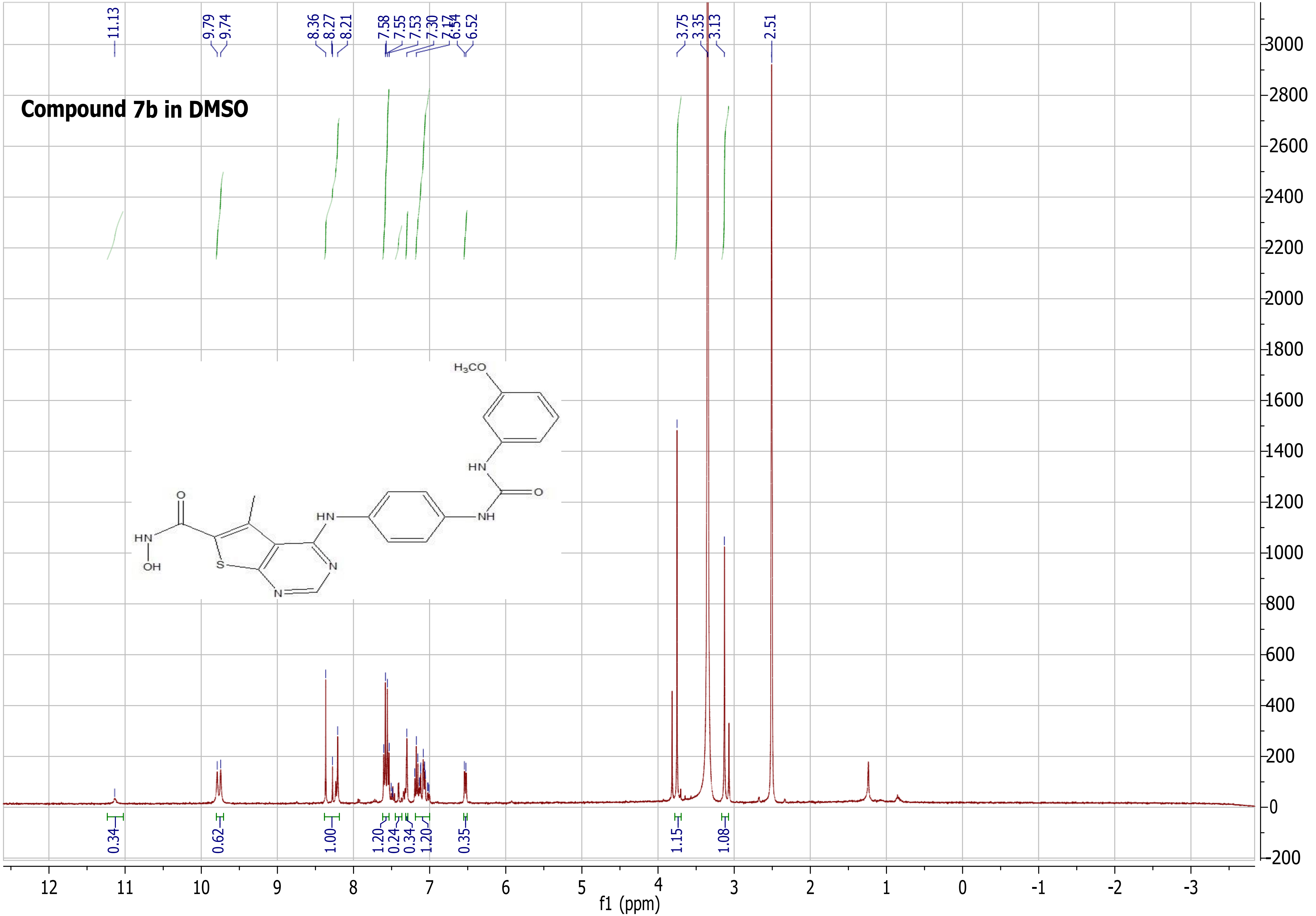
Compound 7b in DMSO
11.13
9.79
9.74
8.36
8.27
8.21
7.58
7.55
7.53
7.30
7.17
7.14
6.54
6.52
3.75
3.35
3.13
2.51
0.34
0.62
1.00
1.20
0.24
0.34
1.20
0.35
1.15
1.08
f1 (ppm)
H3CO
HN
O
NH
HN
N
N
S
HN
OH
O

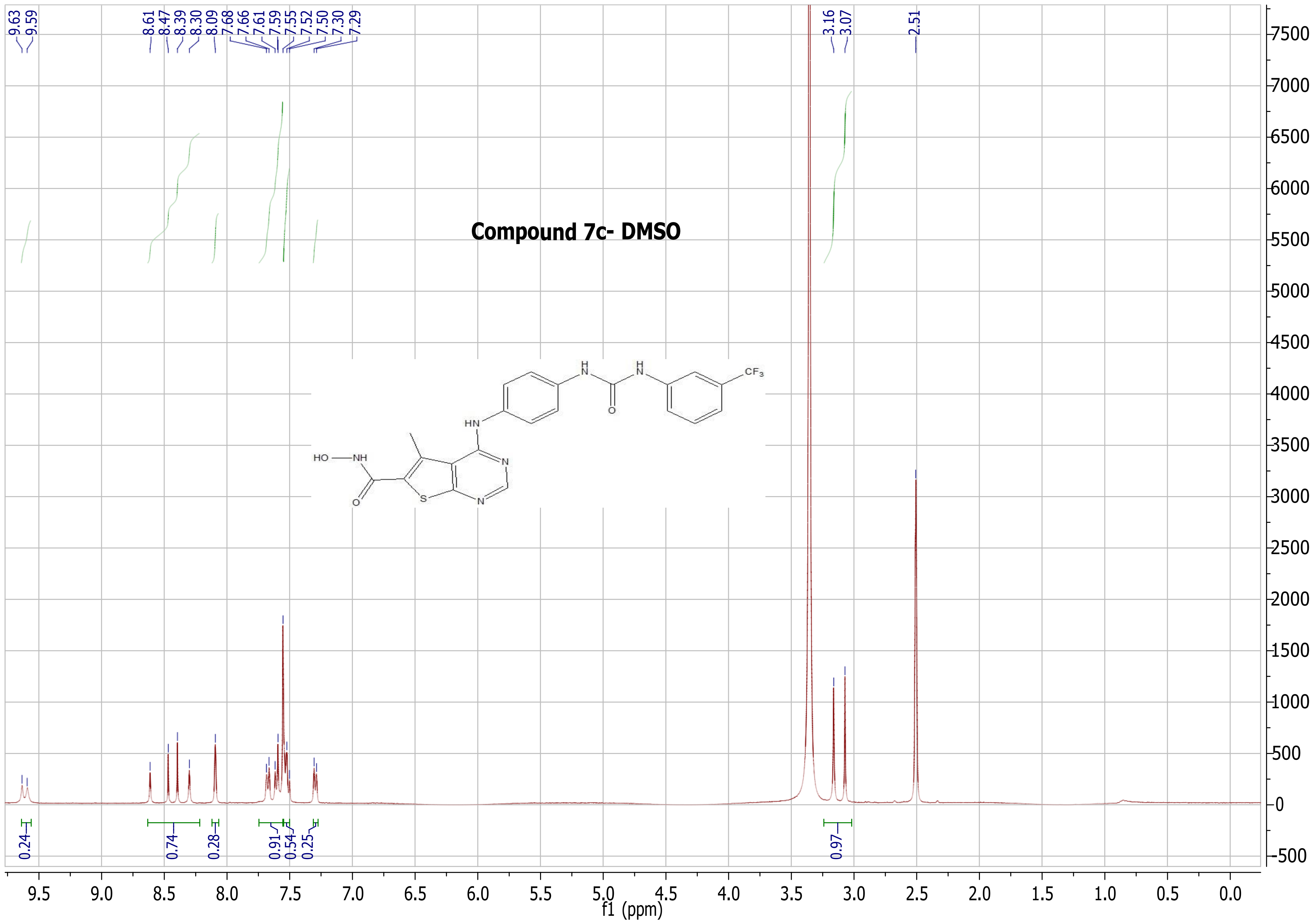

Compound 7c- DMSO
9.63
9.59
8.61
8.47
8.39
8.30
8.09
7.68
7.66
7.61
7.59
7.55
7.52
7.50
7.30
7.29
3.16
3.07
2.51
0.24
0.74
0.28
0.91
0.54
0.25
0.97
f1 (ppm)
HO
NH
HN
N
S
O
CF3

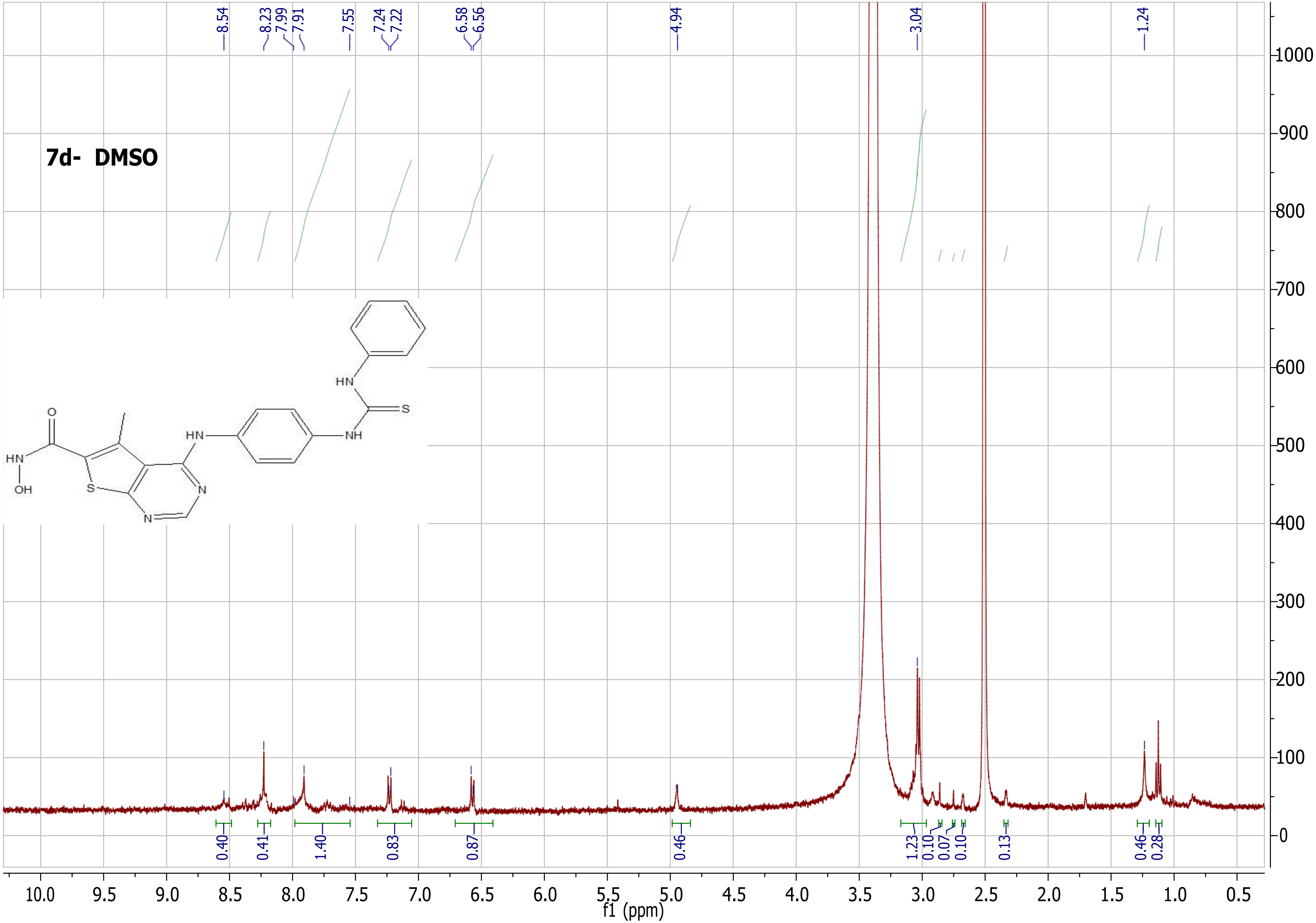

7d- DMSO
8.54
8.23
7.99
7.91
7.55
7.24
7.22
6.58
6.56
4.94
3.04
1.24
0.40
0.41
1.40
0.83
0.87
0.46
1.23
0.10
0.07
0.10
0.13
0.46
0.28
HN
OH
O
S
N
N
HN
NH
S
HN
10.0 9.5 9.0 8.5 8.0 7.5 7.0 6.5 6.0 5.5 5.0 4.5 4.0 3.5 3.0 2.5 2.0 1.5 1.0 0.5
f1 (ppm)
1000 900 800 700 600 500 400 300 200 100 0

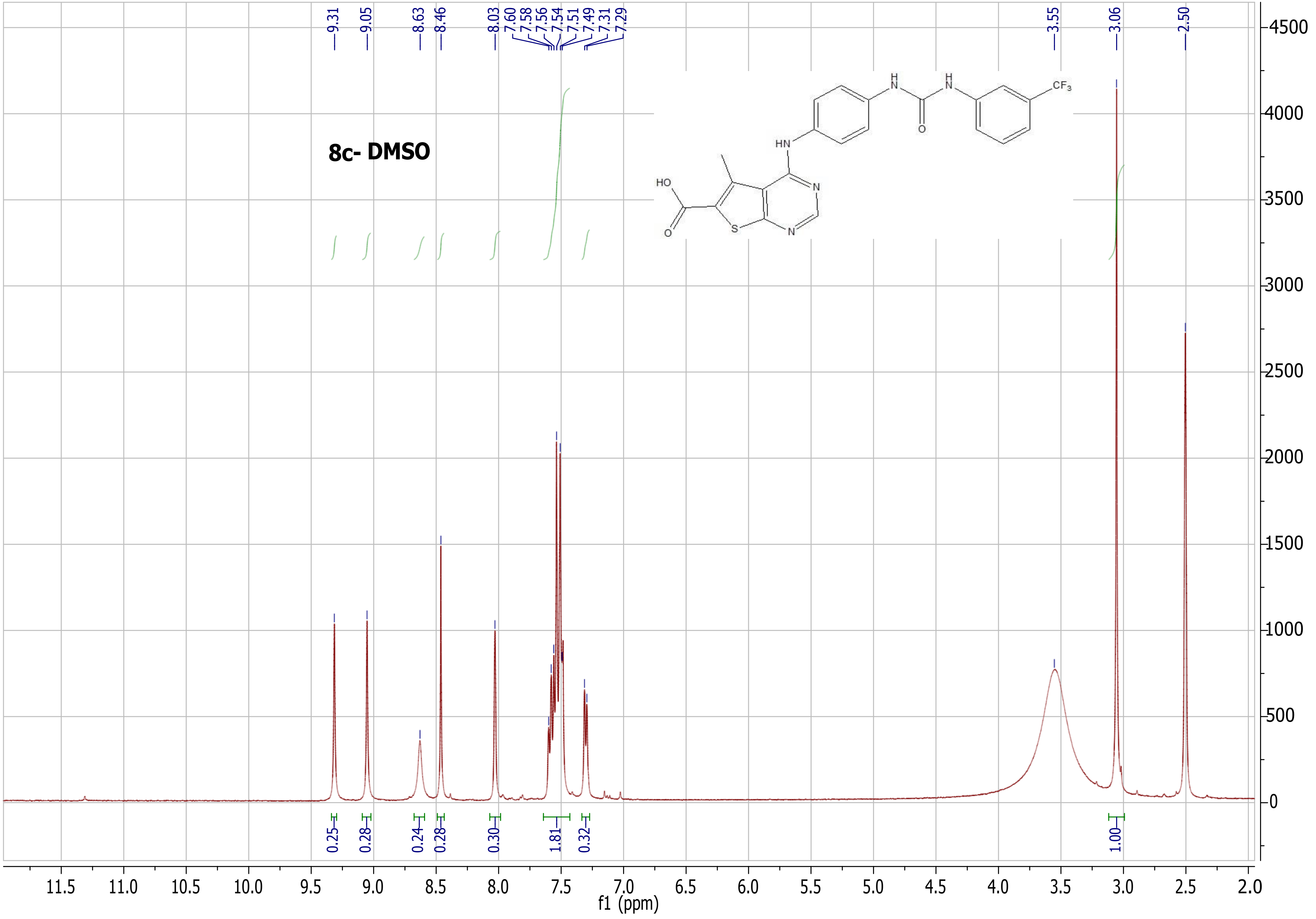

8c- DMSO
9.31
9.05
8.63
8.46
8.03
7.60
7.58
7.56
7.54
7.51
7.49
7.31
7.29
3.55
3.06
2.50
0.25
0.28
0.24
0.28
0.30
1.81
0.32
1.00
HO
O
S
N
N
HN
H
N
O
H
N
CF_3
f1 (ppm)

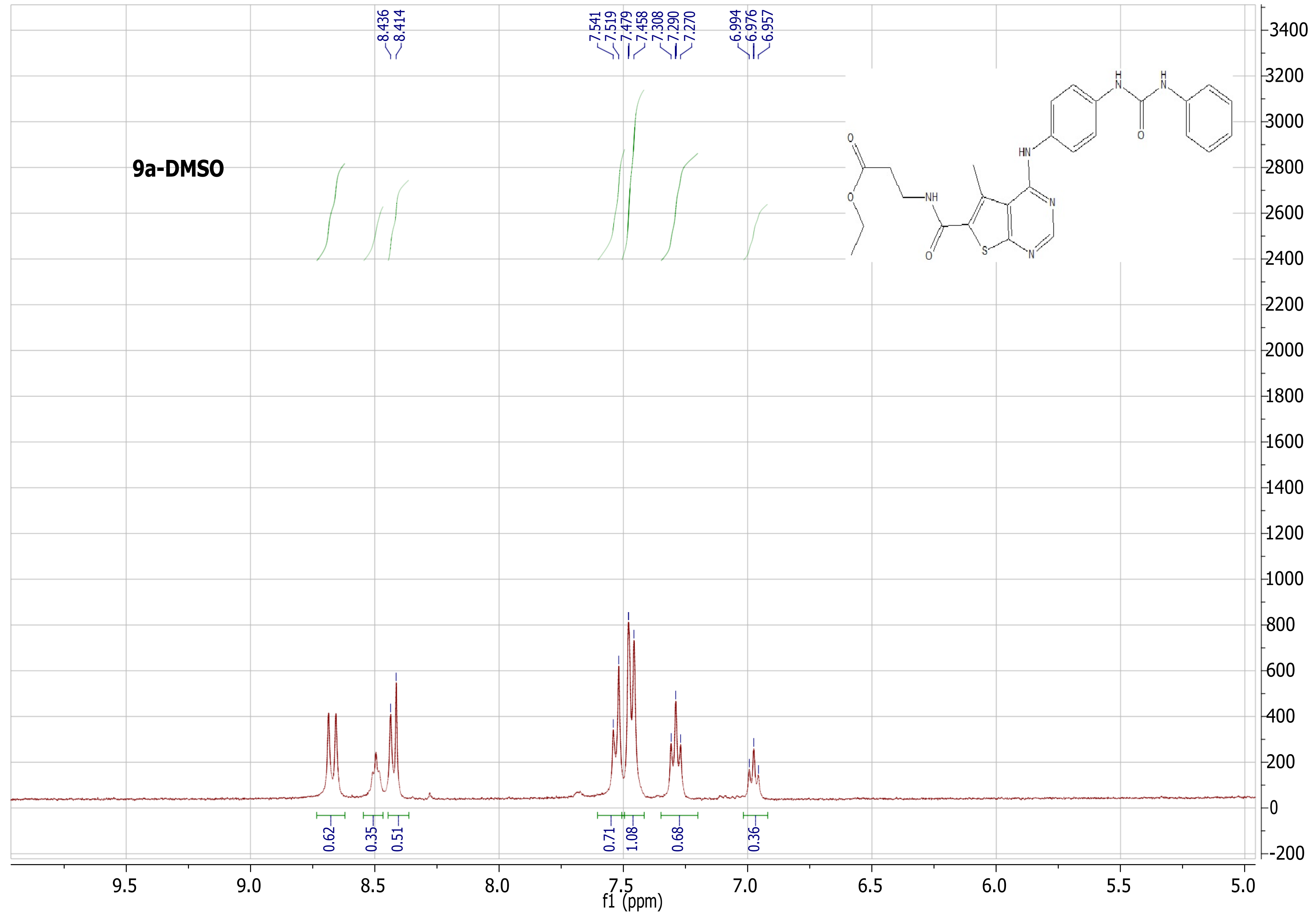

9a-DMSO
8.436
8.414
7.541
7.519
7.479
7.458
7.308
7.290
7.270
6.994
6.976
6.957
0.62
0.35
0.51
0.71
1.08
0.68
0.36
f1 (ppm)

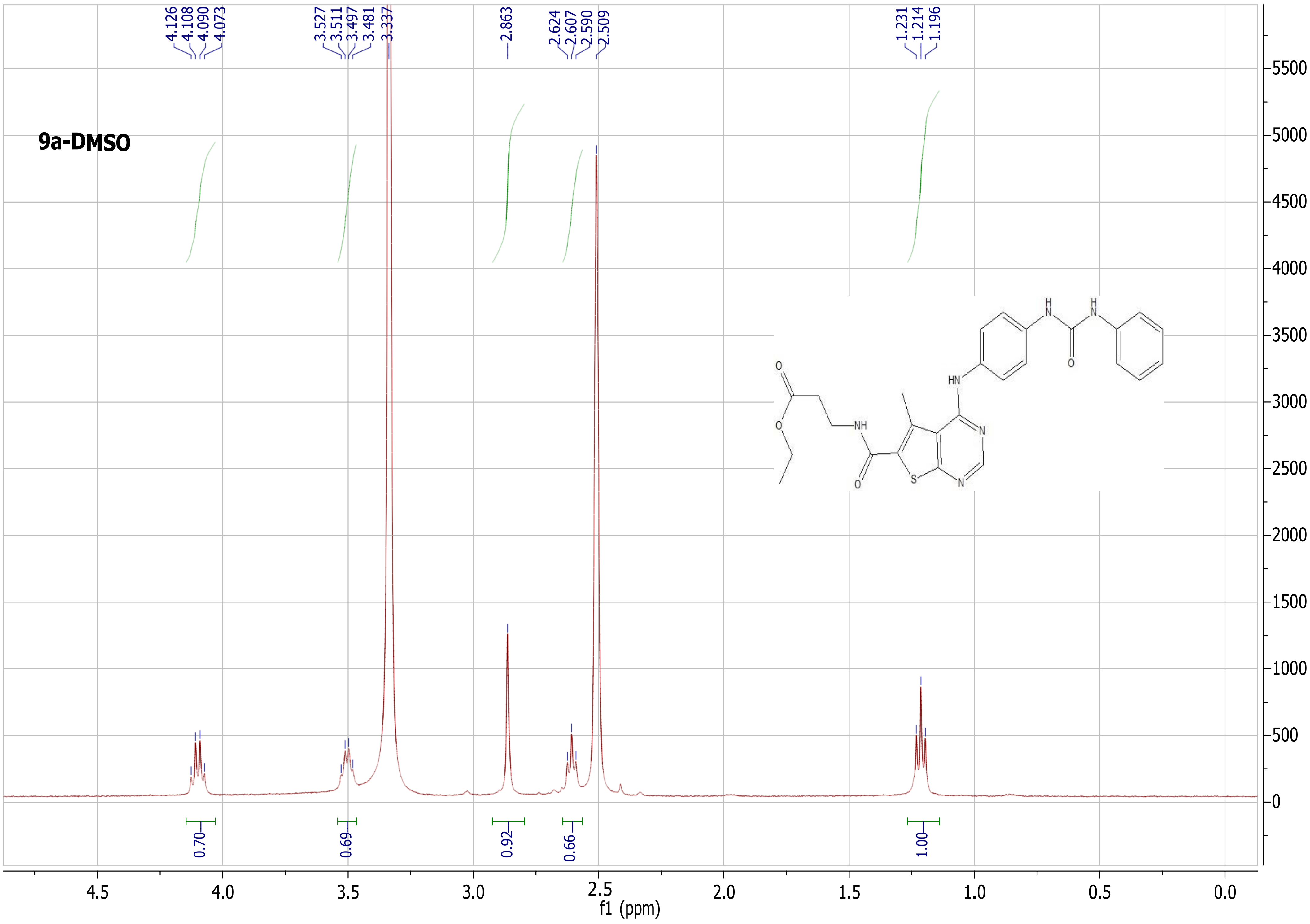

9a-DMSO
4.126
4.108
4.090
4.073
3.527
3.511
3.497
3.481
3.337
2.863
2.624
2.607
2.590
2.509
1.231
1.214
1.196
0.70
0.69
0.92
0.66
1.00
4.5
4.0
3.5
3.0
2.5
2.0
1.5
1.0
0.5
0.0
f1 (ppm)
5500
5000
4500
4000
3500
3000
2500
2000
1500
1000
500
0

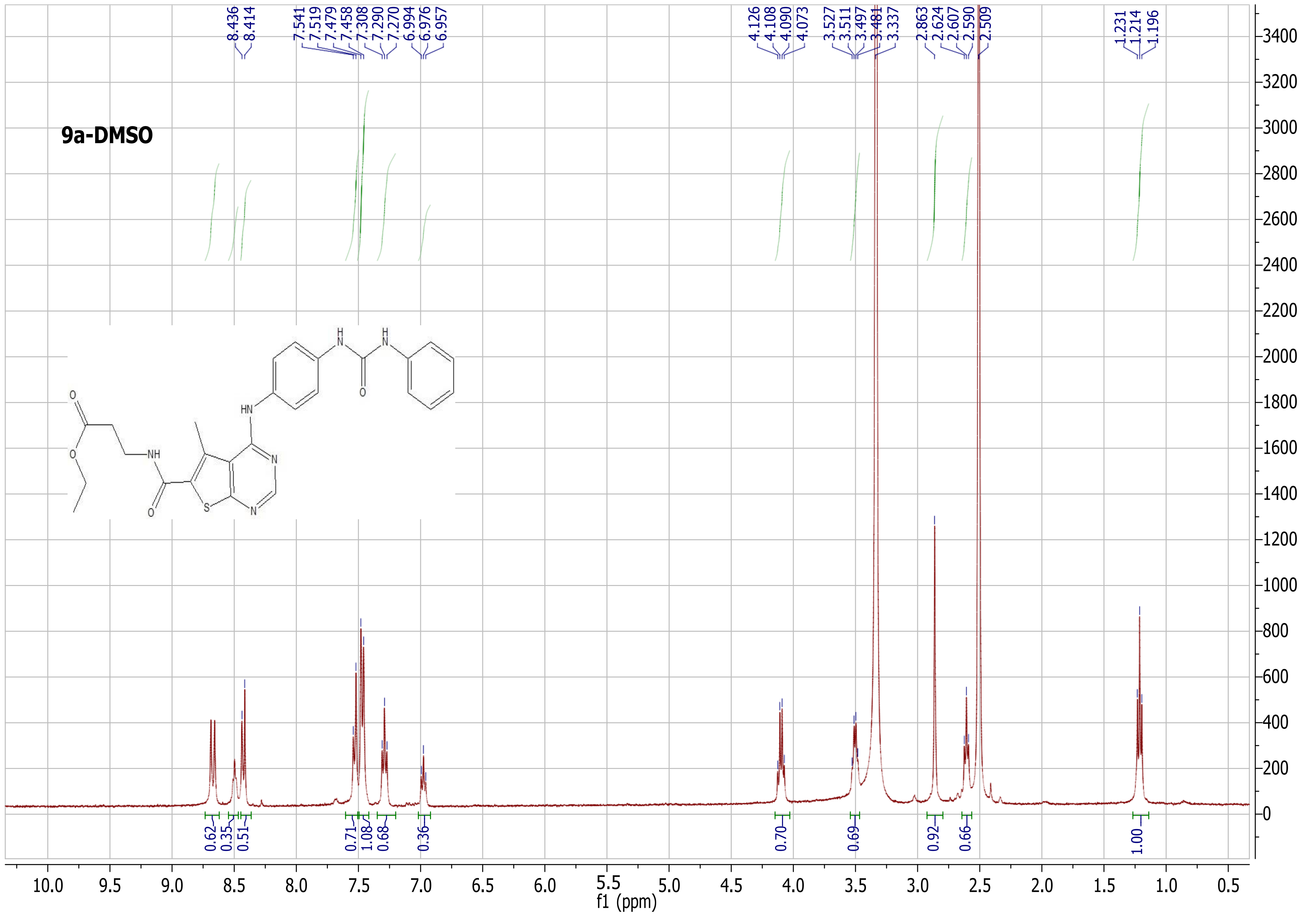

9a-DMSO
8.436
8.414
7.541
7.519
7.479
7.458
7.308
7.290
7.270
6.994
6.976
6.957
4.126
4.108
4.090
4.073
3.527
3.511
3.497
3.481
3.337
2.863
2.624
2.607
2.590
2.509
1.231
1.214
1.196
0.62
0.35
0.51
0.71
1.08
0.68
0.36
0.70
0.69
0.92
0.66
1.00
f1 (ppm)

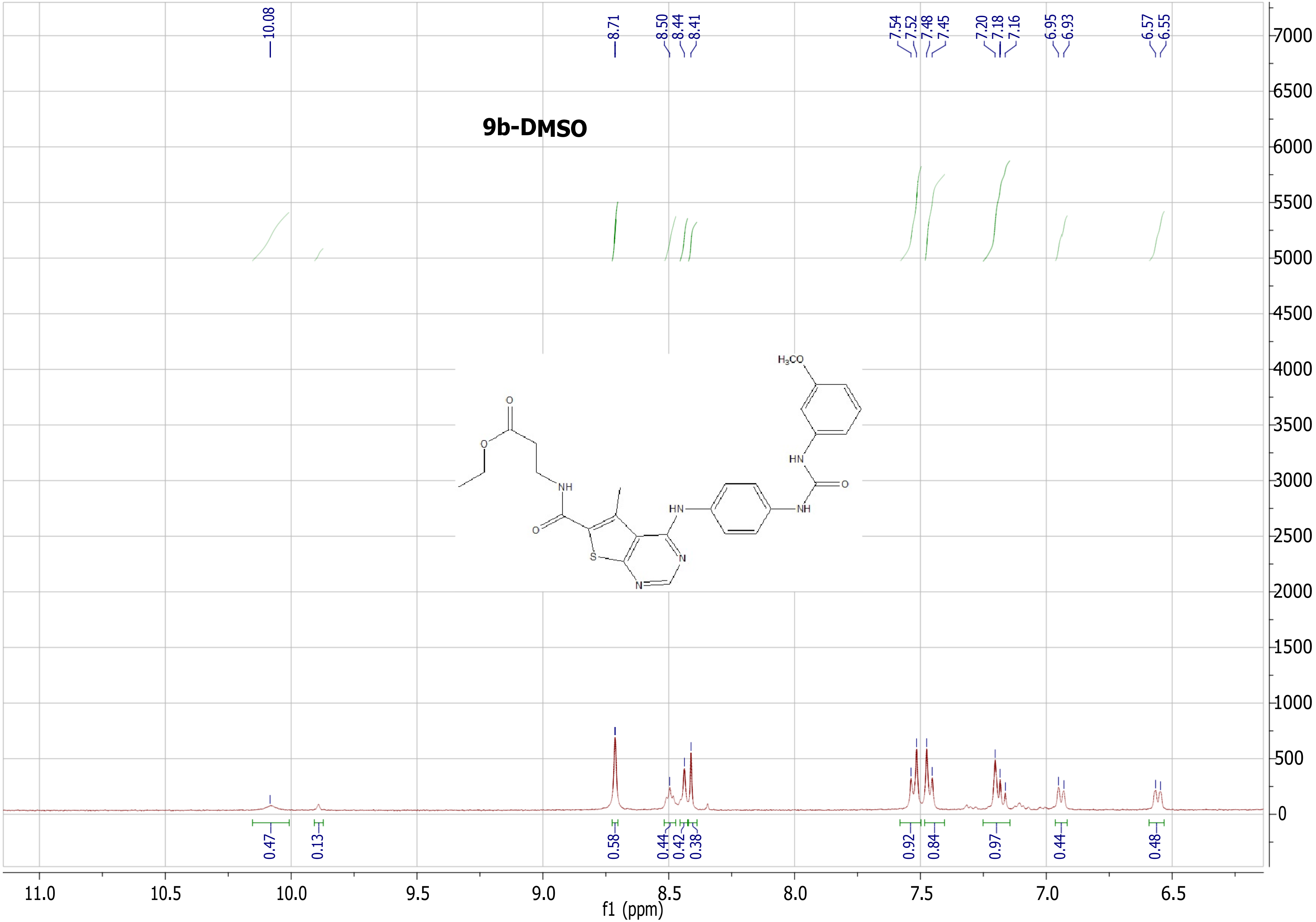

9b-DMSO
10.08
8.71
8.50
8.44
8.41
7.54
7.52
7.48
7.45
7.20
7.18
7.16
6.95
6.93
6.57
6.55
0.47
0.13
0.58
0.44
0.42
0.38
0.92
0.84
0.97
0.44
0.48
H3CO
HN
O
NH
HN
O
NH
O
O
S
N
N
7000
6500
6000
5500
5000
4500
4000
3500
3000
2500
2000
1500
1000
500
0
11.0
10.5
10.0
9.5
9.0
8.5
8.0
7.5
7.0
6.5
f1 (ppm)

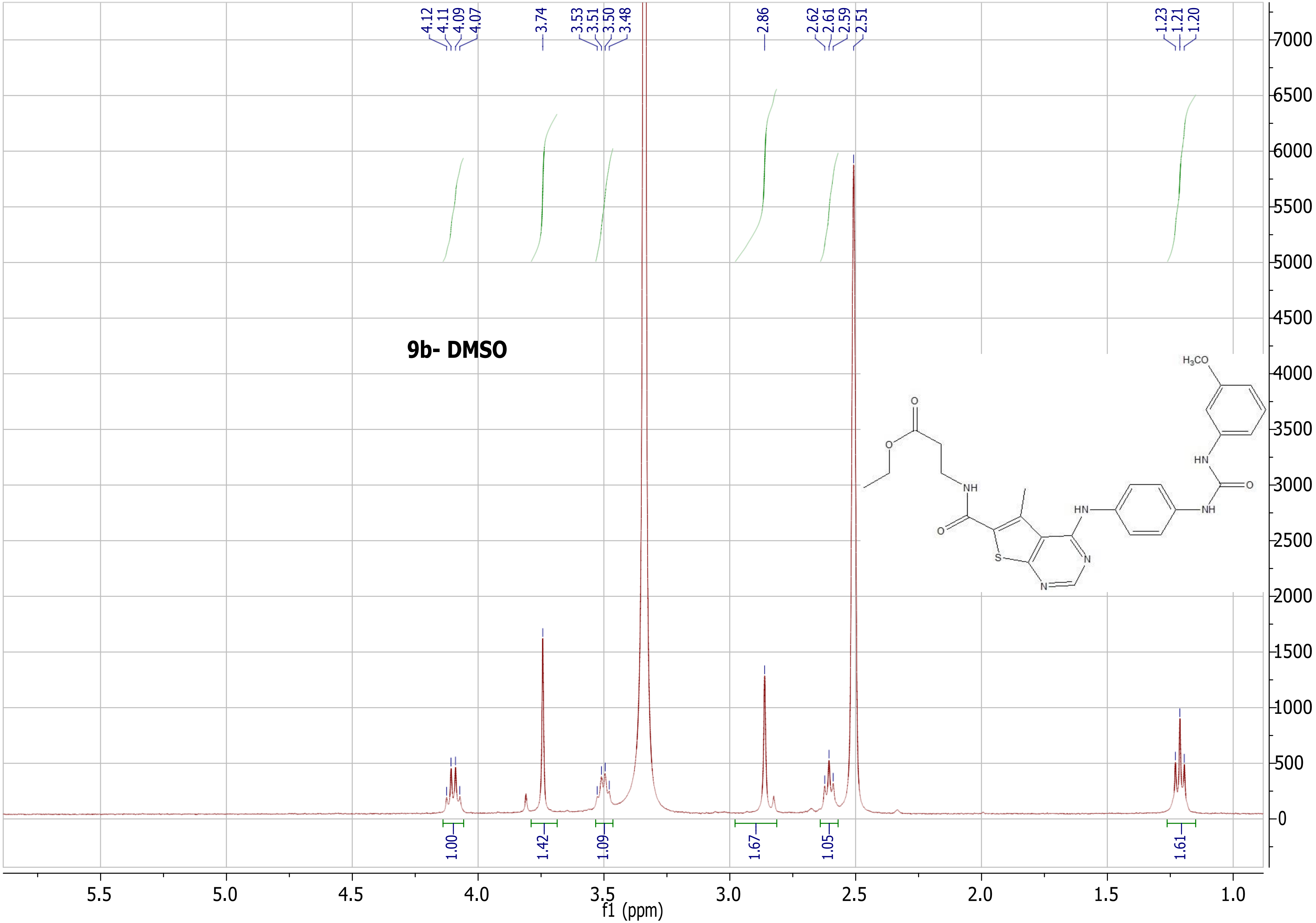
9b- DMSO
4.12
4.11
4.09
4.07
3.74
3.53
3.51
3.50
3.48
2.86
2.62
2.61
2.59
2.51
1.23
1.21
1.20
1.00
1.42
1.09
1.67
1.05
1.61
7000
6500
6000
5500
5000
4500
4000
3500
3000
2500
2000
1500
1000
500
0
5.5
5.0
4.5
4.0
3.5
3.0
2.5
2.0
1.5
1.0
f1 (ppm)
H3CO
O
HN
NH
O
HN
NH
O
S
N
N

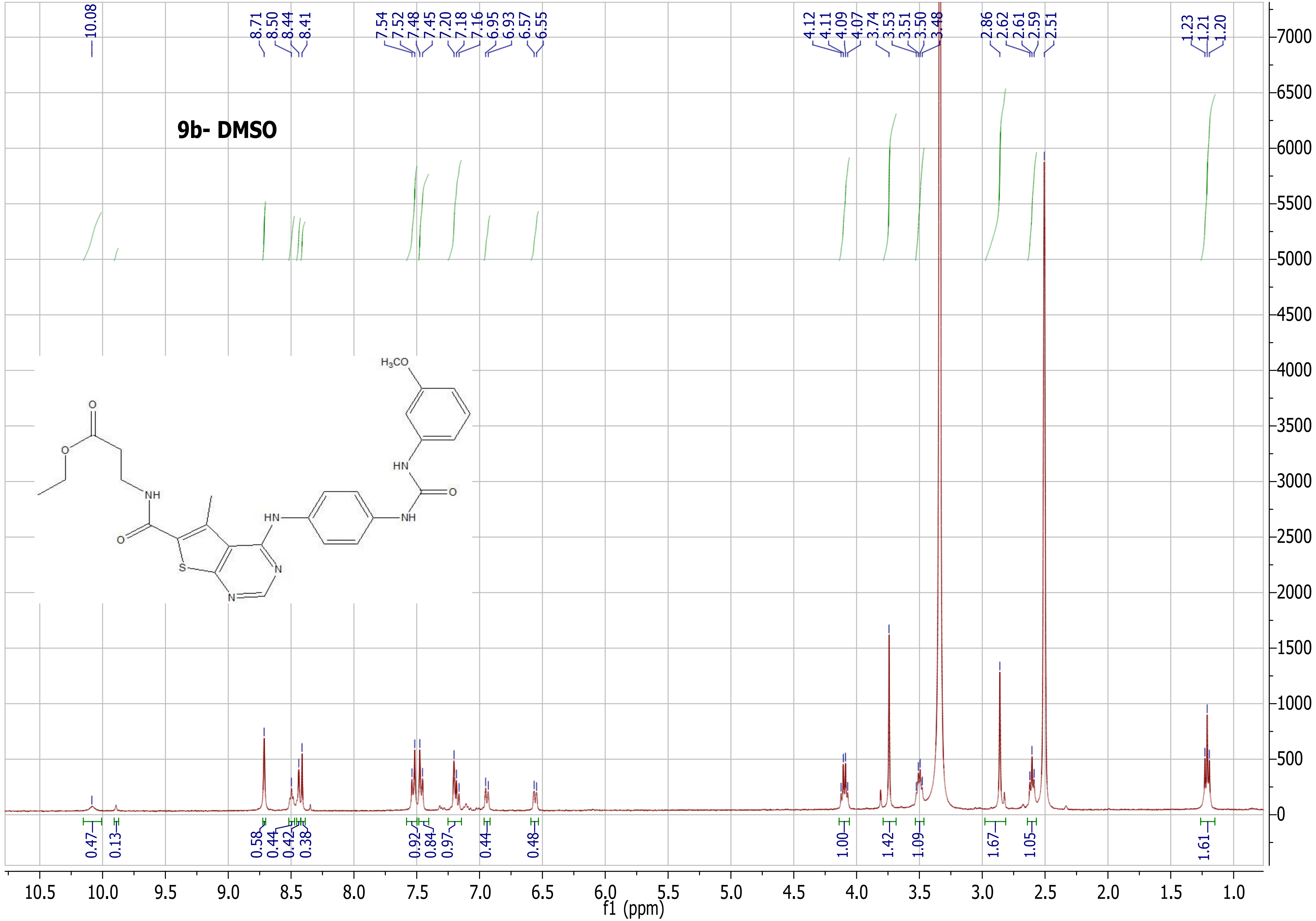

9b- DMSO
10.08
8.71
8.50
8.44
8.41
7.54
7.52
7.48
7.45
7.20
7.18
7.16
6.95
6.93
6.57
6.55
4.12
4.11
4.09
4.07
3.74
3.53
3.51
3.50
3.48
2.86
2.62
2.61
2.59
2.51
1.23
1.21
1.20
0.47
0.13
0.58
0.44
0.42
0.38
0.92
0.84
0.97
0.44
0.48
1.00
1.42
1.09
1.67
1.05
1.61
f1 (ppm)
H3CO
HN
NH
O
S
N

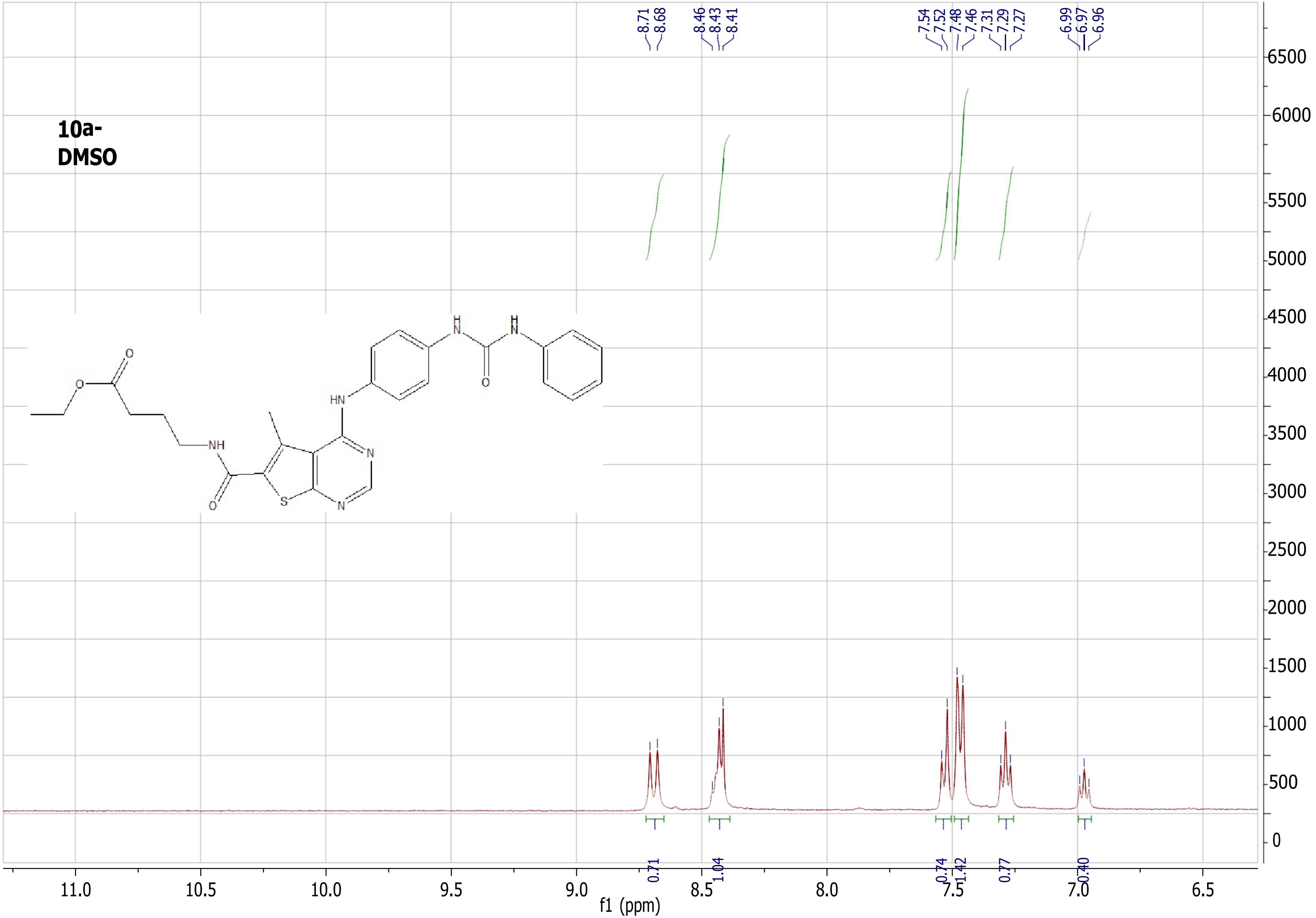

10a-
DMSO
8.71
8.68
8.46
8.43
8.41
7.54
7.52
7.48
7.46
7.31
7.29
7.27
6.99
6.97
6.96
0.71
1.04
0.74
1.42
0.77
0.40
f1 (ppm)
11.0
10.5
10.0
9.5
9.0
8.5
8.0
7.5
7.0
6.5
6500
6000
5500
5000
4500
4000
3500
3000
2500
2000
1500
1000
500
0
O
O
NH
O
S
N
N
HN
H
N
H
N
O

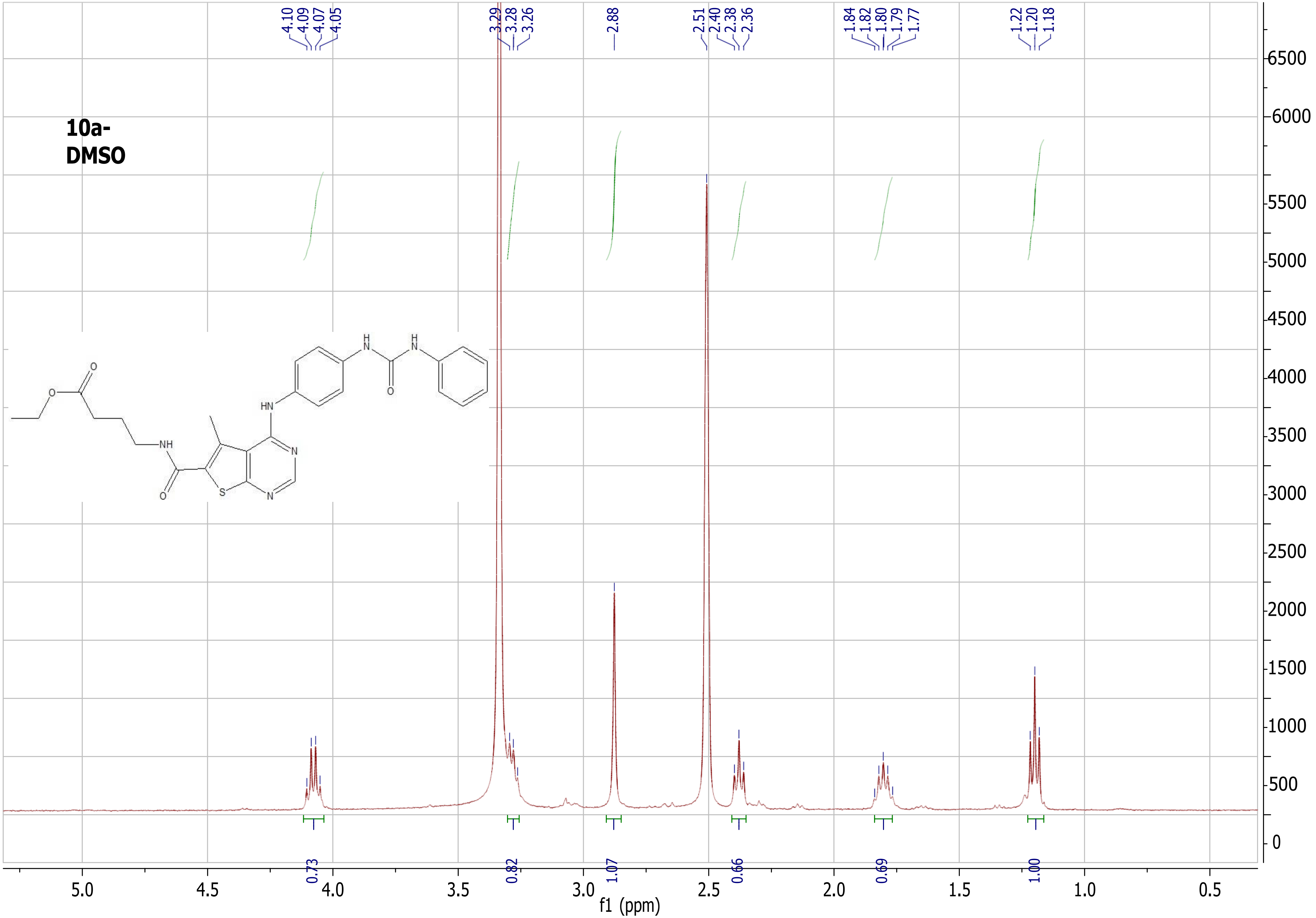
10a-
DMSO
4.10
4.09
4.07
4.05
3.29
3.28
3.26
2.88
2.51
2.40
2.38
2.36
1.84
1.82
1.80
1.79
1.77
1.22
1.20
1.18
0.73
0.82
1.07
0.66
0.69
1.00
f1 (ppm)
5.0
4.5
4.0
3.5
3.0
2.5
2.0
1.5
1.0
0.5
6500
6000
5500
5000
4500
4000
3500
3000
2500
2000
1500
1000
500
0
O
O
NH
O
S
N
N
HN
H
N
O
H
N

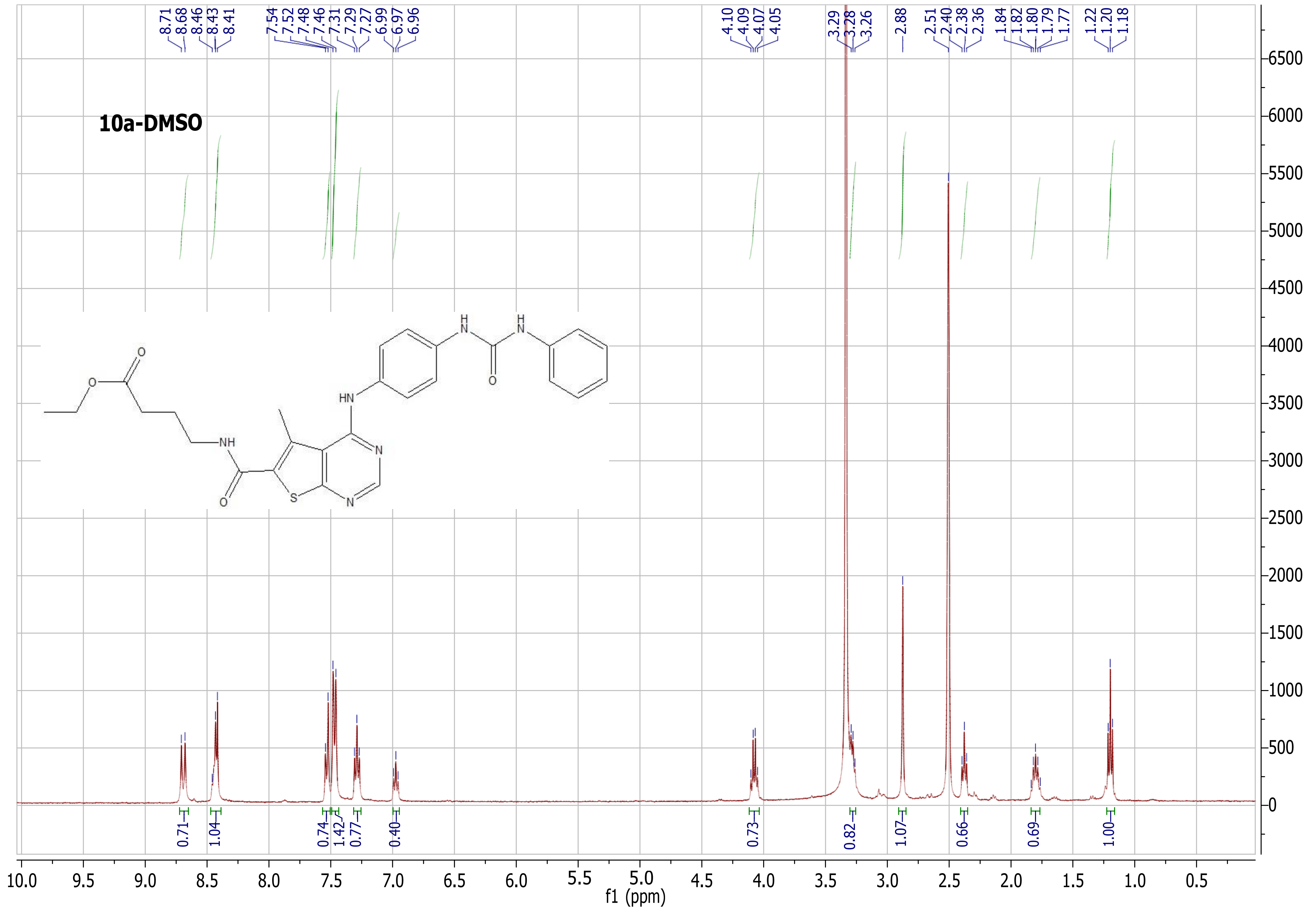
10a-DMSO
8.71
8.68
8.46
8.43
8.41
7.54
7.52
7.48
7.46
7.31
7.29
7.27
6.99
6.97
6.96
4.10
4.09
4.07
4.05
3.29
3.28
3.26
2.88
2.51
2.40
2.38
2.36
1.84
1.82
1.80
1.79
1.77
1.22
1.20
1.18
0.71
1.04
0.74
1.42
0.77
0.40
0.73
0.82
1.07
0.66
0.69
1.00
f1 (ppm)

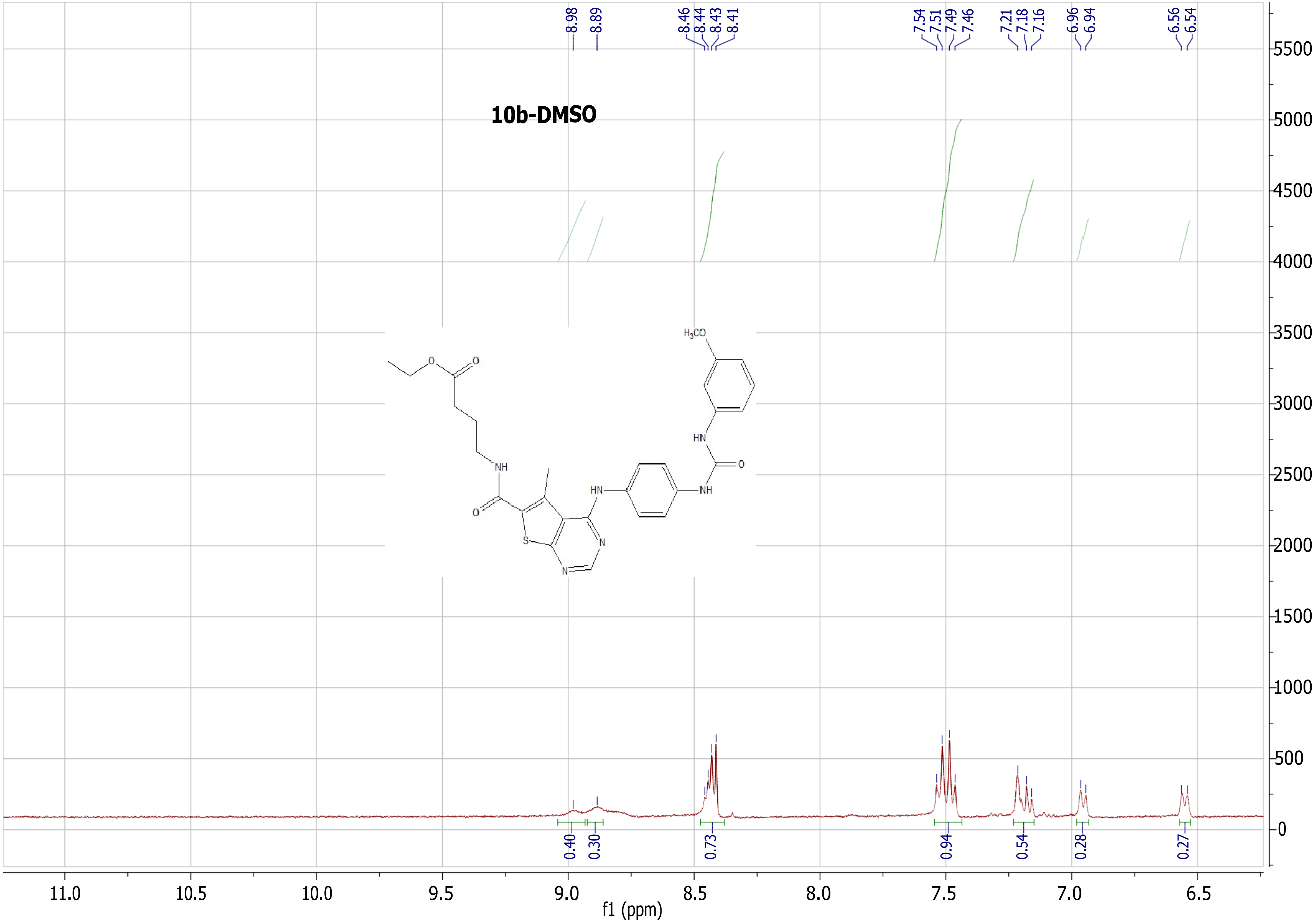

10b-DMSO
8.98
8.89
8.46
8.44
8.43
8.41
7.54
7.51
7.49
7.46
7.21
7.18
7.16
6.96
6.94
6.56
6.54
0.40
0.30
0.73
0.94
0.54
0.28
0.27
f1 (ppm)
H3CO
HN
O
NH
S
N

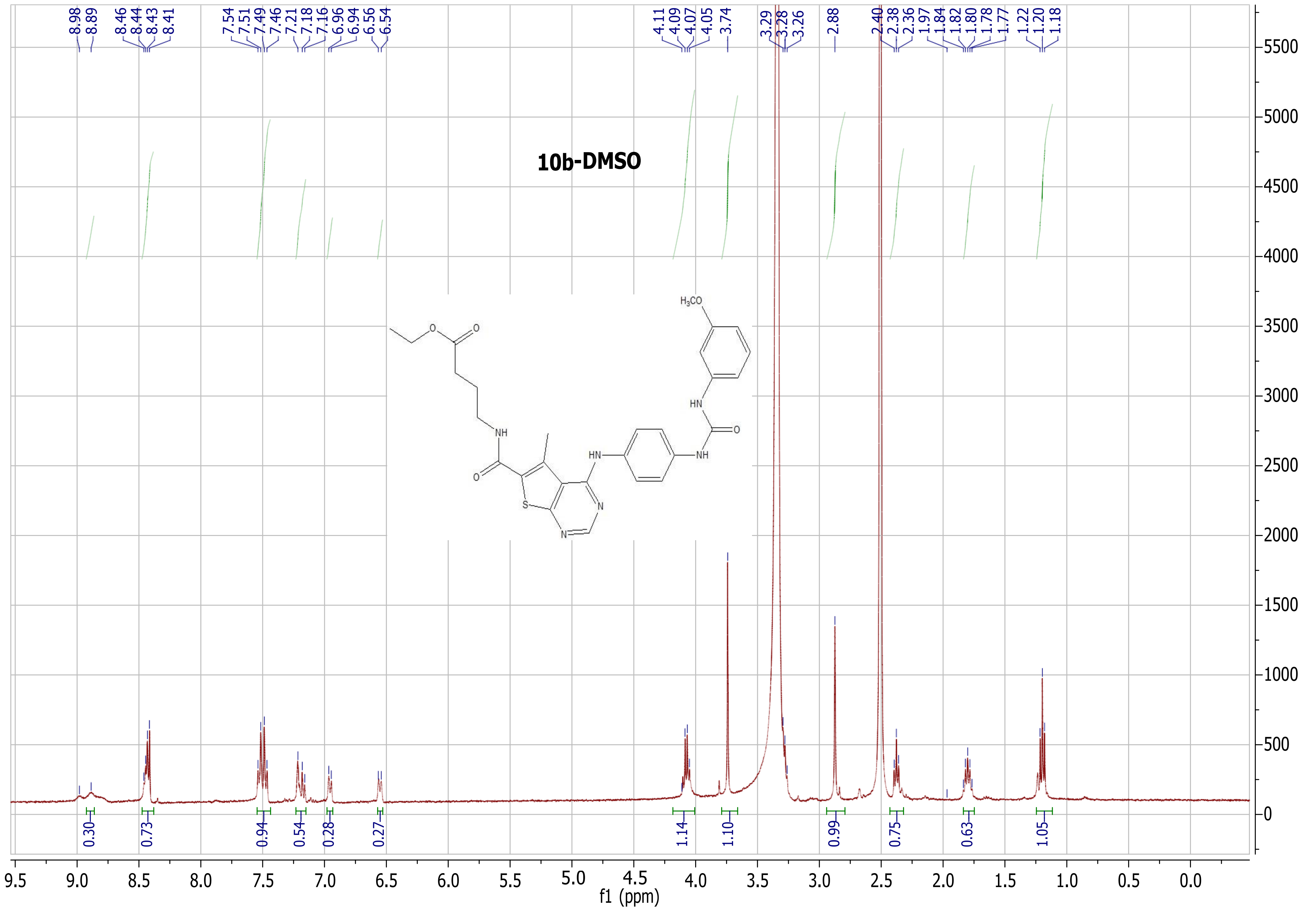

10b-DMSO
8.98
8.89
8.46
8.44
8.43
8.41
7.54
7.51
7.49
7.46
7.21
7.18
7.16
6.96
6.94
6.56
6.54
4.11
4.09
4.07
4.05
3.74
3.29
3.28
3.26
2.88
2.40
2.38
2.36
1.97
1.84
1.82
1.80
1.78
1.77
1.22
1.20
1.18
0.30
0.73
0.94
0.54
0.28
0.27
1.14
1.10
0.99
0.75
0.63
1.05
f1 (ppm)

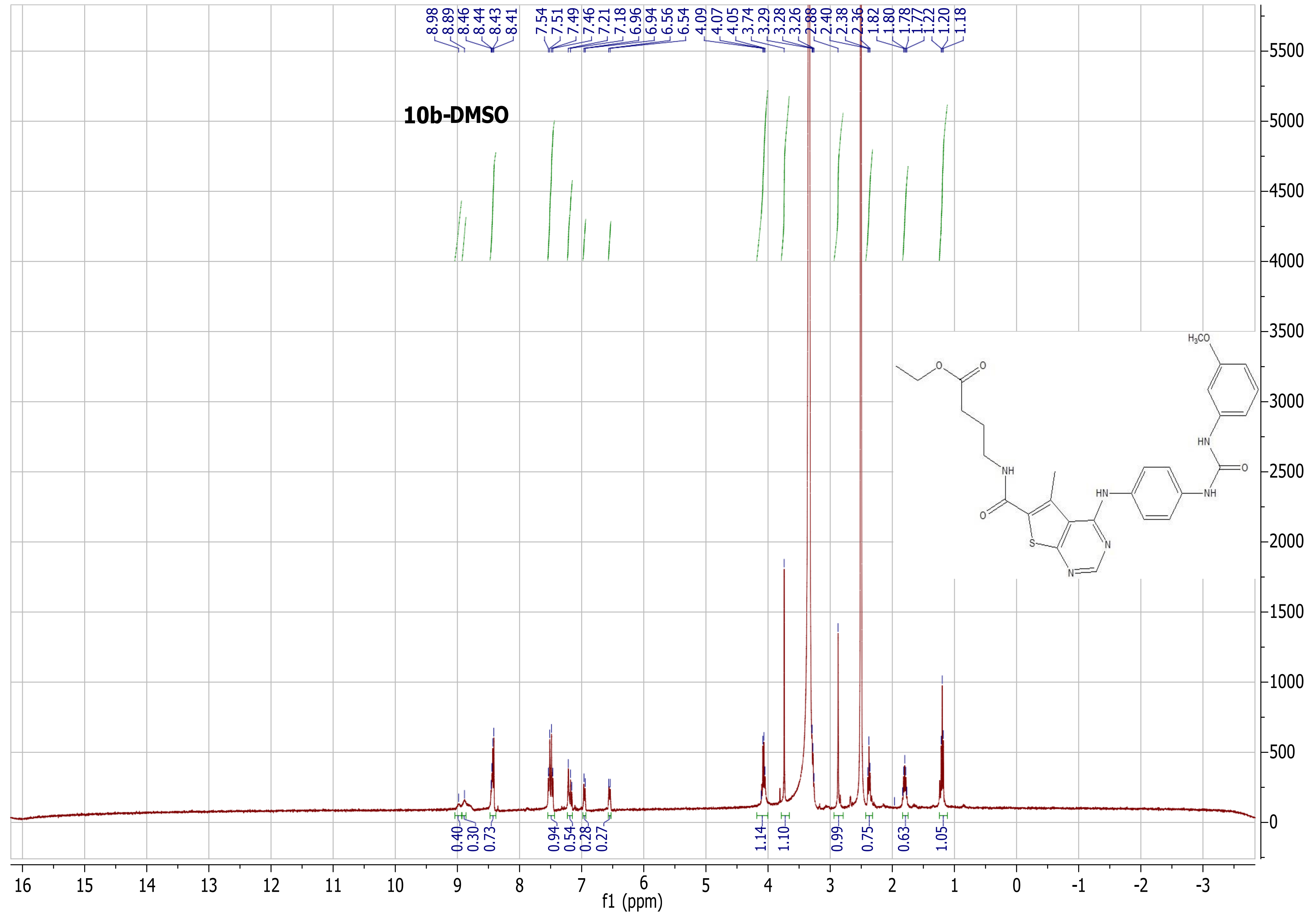
10b-DMSO
8.98
8.89
8.46
8.44
8.43
8.41
7.54
7.51
7.49
7.46
7.21
7.18
6.96
6.94
6.56
6.54
4.09
4.07
4.05
3.74
3.29
3.28
3.26
2.88
2.40
2.38
2.36
1.82
1.80
1.78
1.77
1.22
1.20
1.18
0.40
0.30
0.73
0.94
0.54
0.28
0.27
1.14
1.10
0.99
0.75
0.63
1.05
f1 (ppm)
H3CO
HN
NH
O
S
N

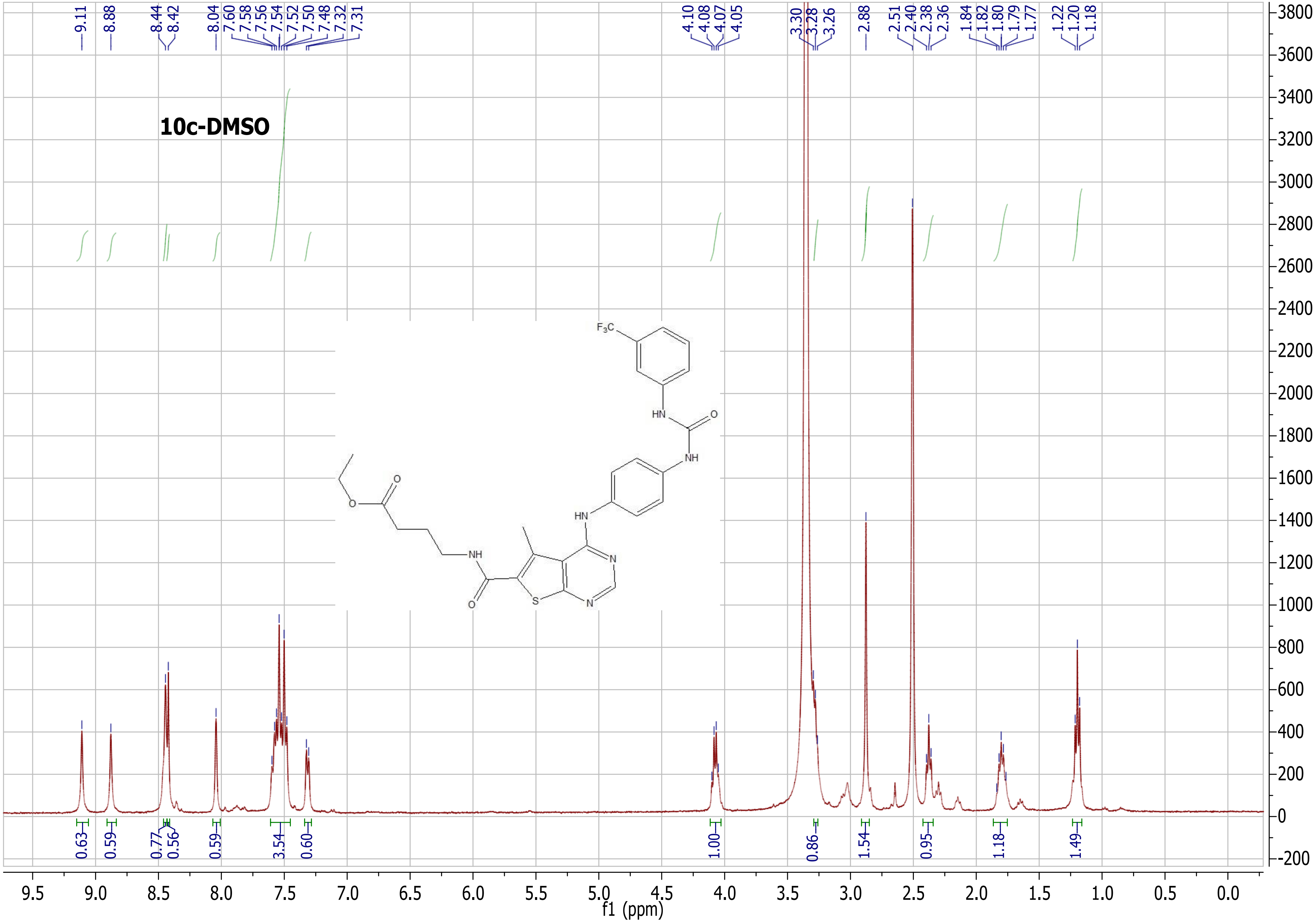
10c-DMSO
9.11
8.88
8.44
8.42
8.04
7.60
7.58
7.56
7.54
7.52
7.50
7.48
7.32
7.31
4.10
4.08
4.07
4.05
3.30
3.28
3.26
2.88
2.51
2.40
2.38
2.36
1.84
1.82
1.80
1.79
1.77
1.22
1.20
1.18
0.63
0.59
0.77
0.56
0.59
3.54
0.60
1.00
0.86
1.54
0.95
1.18
1.49
F3C
HN
O
NH
HN
N
N
S
O
NH
O
O
f1 (ppm)

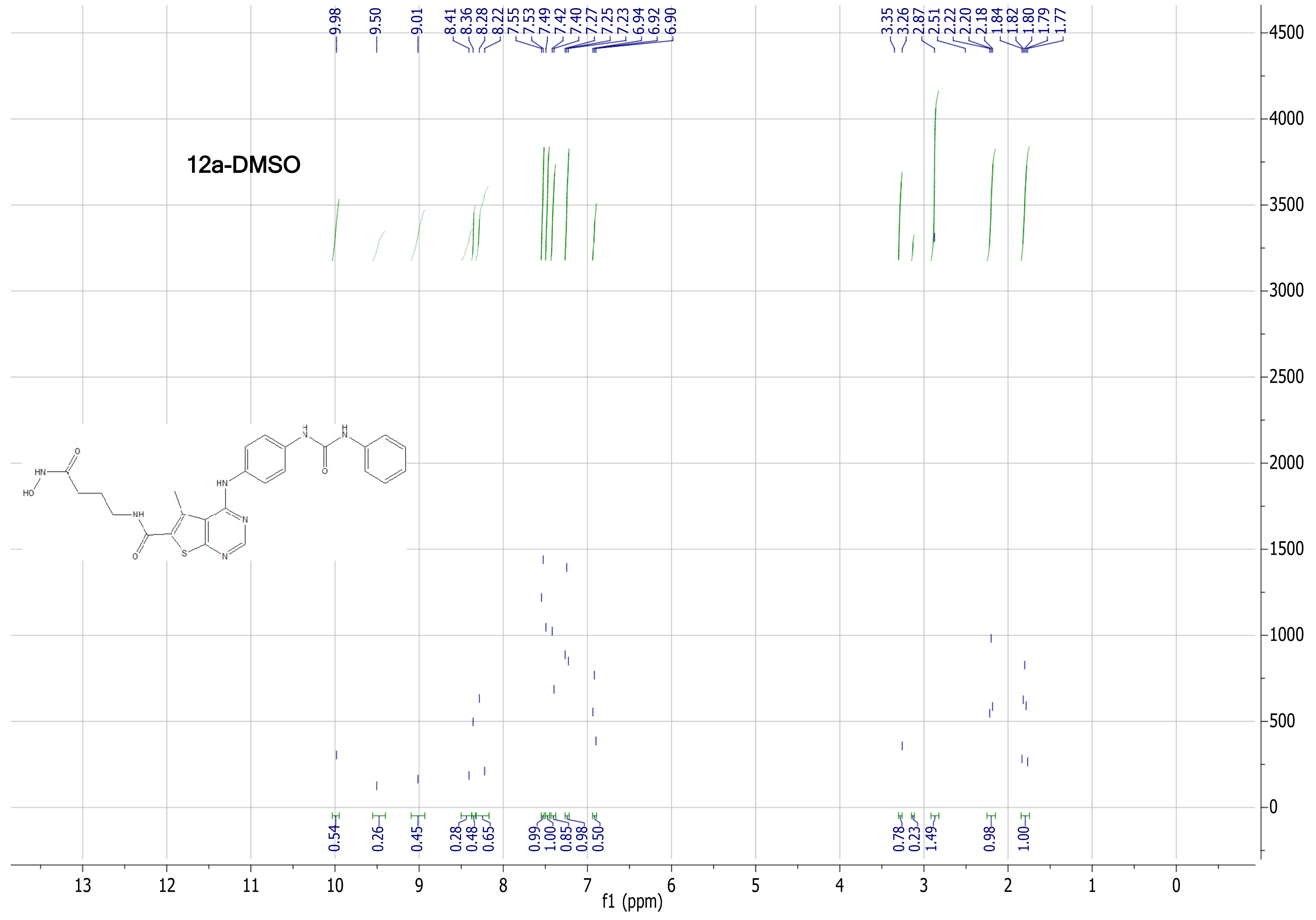
12a-DMSO
9.98
9.50
9.01
8.41
8.36
8.28
8.22
7.55
7.53
7.49
7.42
7.40
7.27
7.25
7.23
6.94
6.92
6.90
3.35
3.26
2.87
2.51
2.22
2.20
2.18
1.84
1.82
1.80
1.79
1.77
0.54
0.26
0.45
0.28
0.48
0.65
0.99
1.00
0.85
0.98
0.50
0.78
0.23
1.49
0.98
1.00
f1 (ppm)

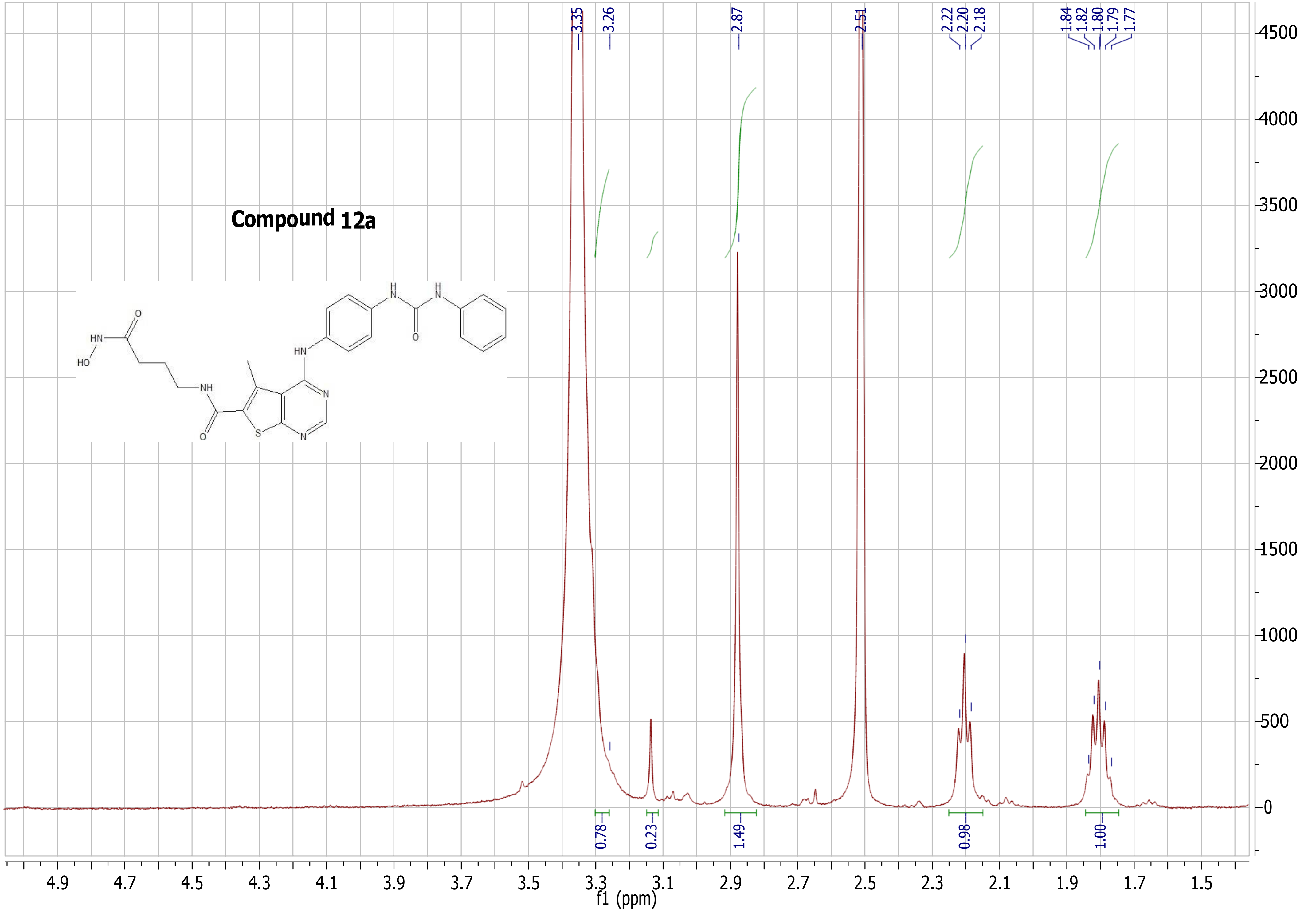

Compound 12a

Compound 12a

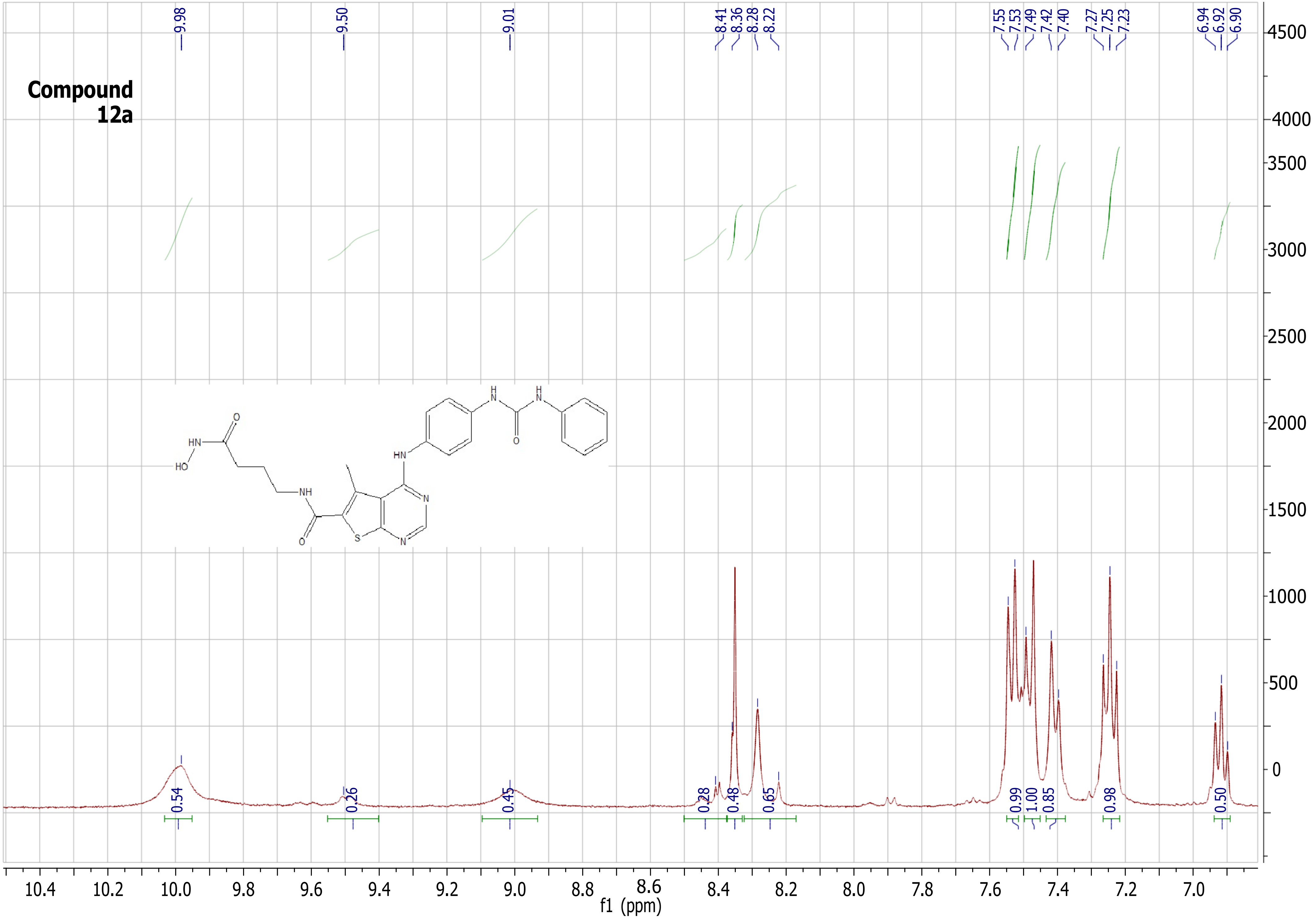

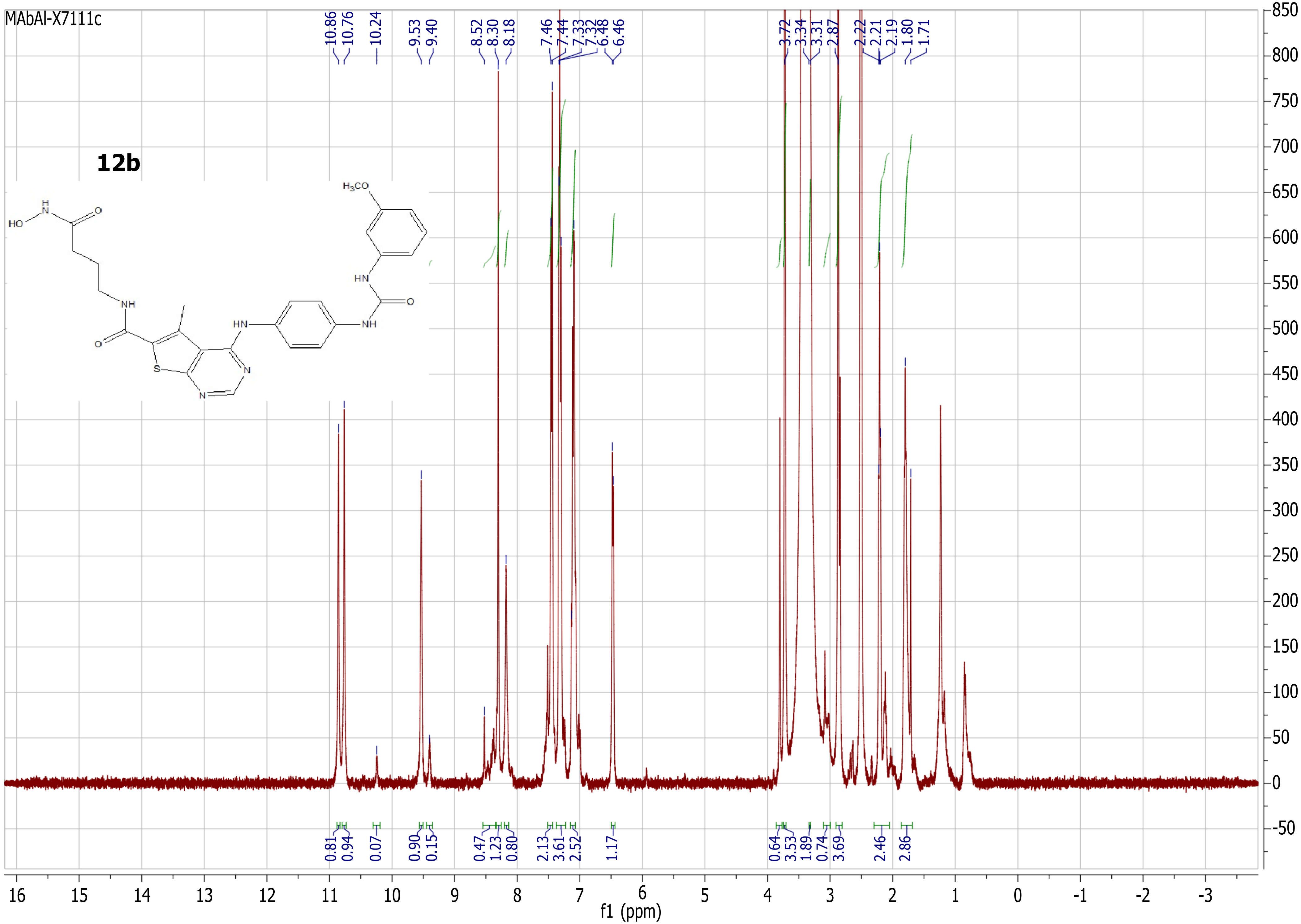
MAbAI-X7111c
12b
10.86
10.76
10.24
9.53
9.40
8.52
8.30
8.18
7.46
7.44
7.33
7.32
6.48
6.46
3.72
3.34
3.31
2.87
2.22
2.21
2.19
1.80
1.71
0.81
0.94
0.07
0.90
0.15
0.47
1.23
0.80
2.13
3.61
2.52
1.17
0.64
3.53
1.89
0.74
3.69
2.46
2.86
f1 (ppm)

compound 12c

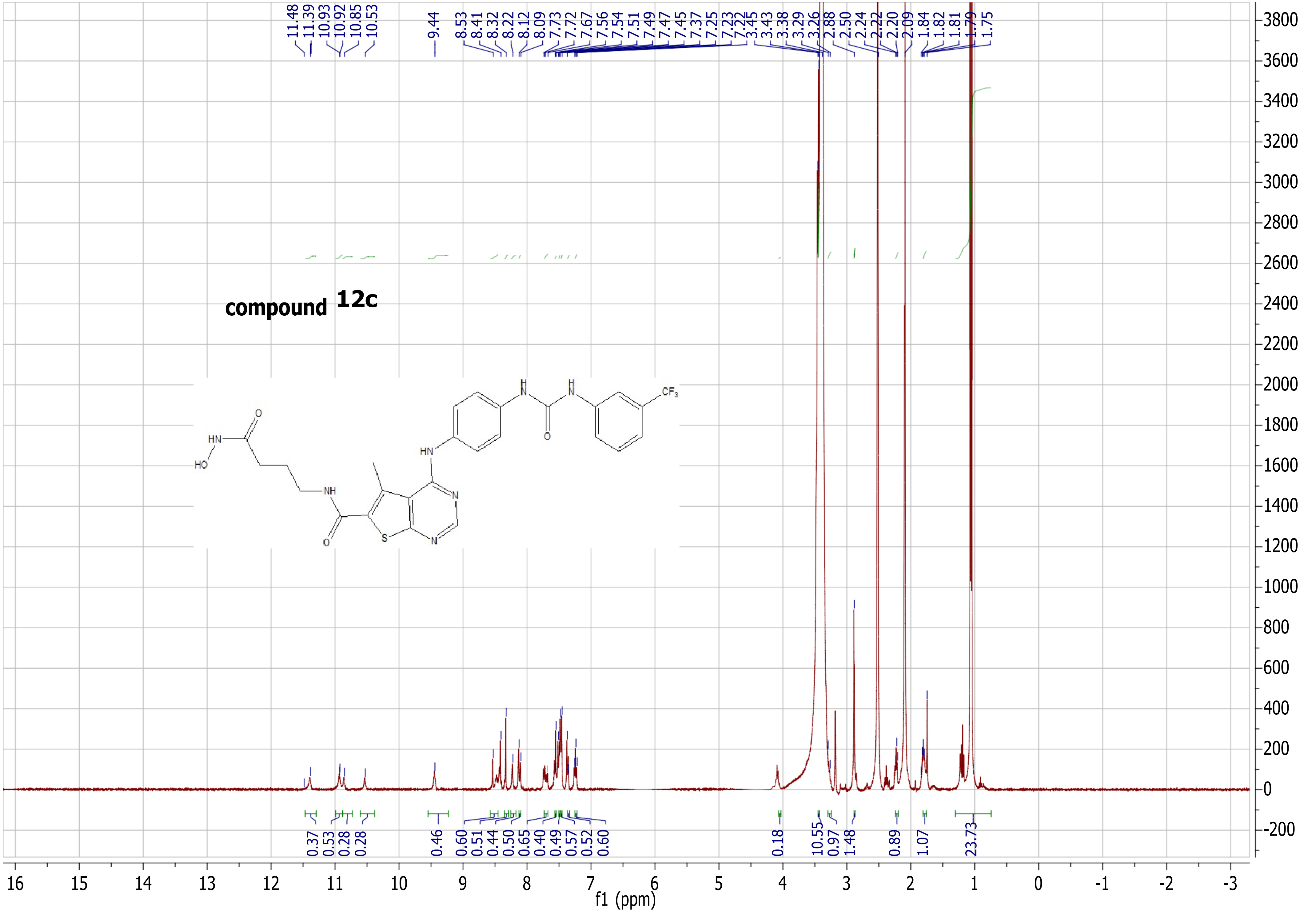

Compound 15a - DMSO

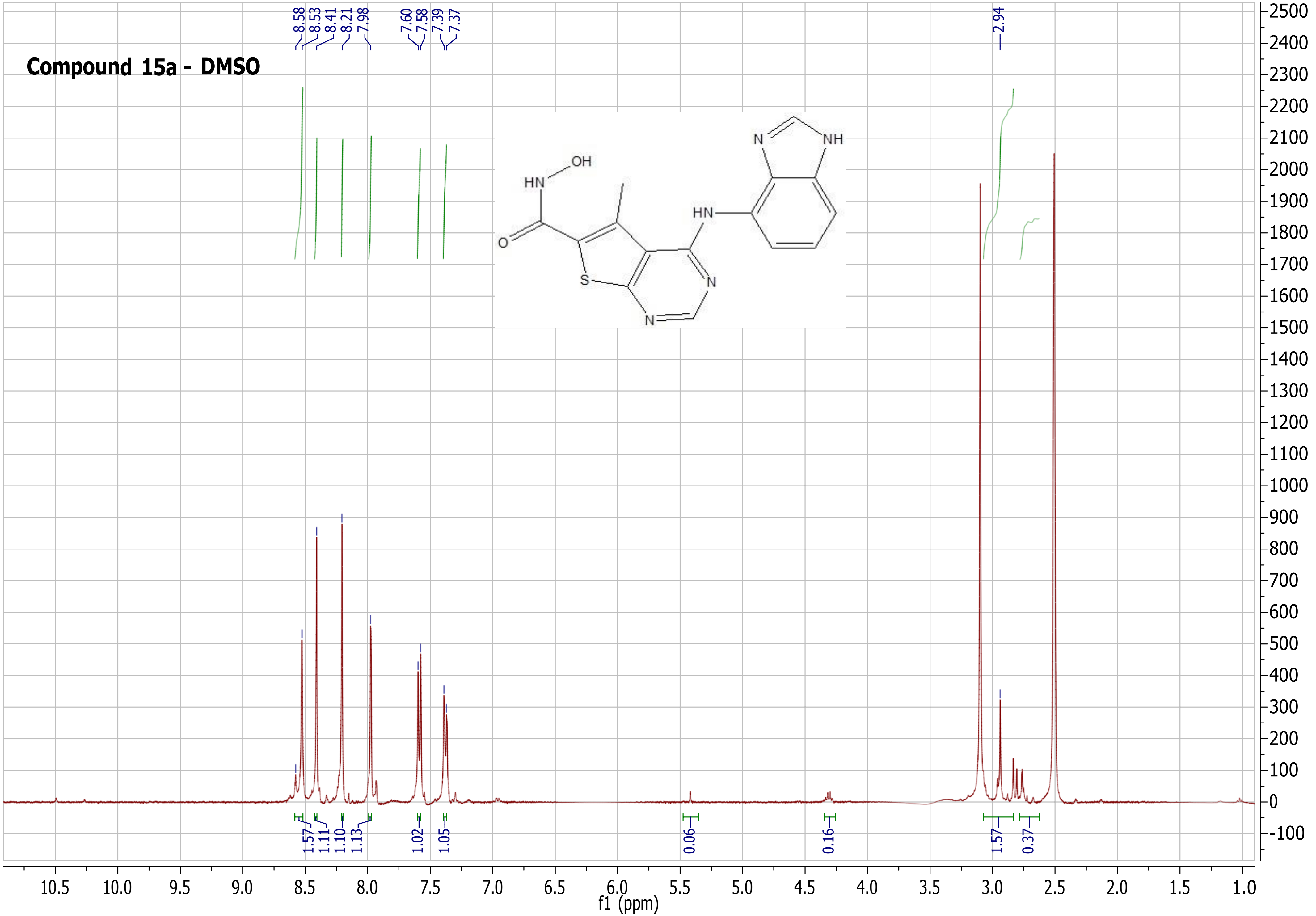

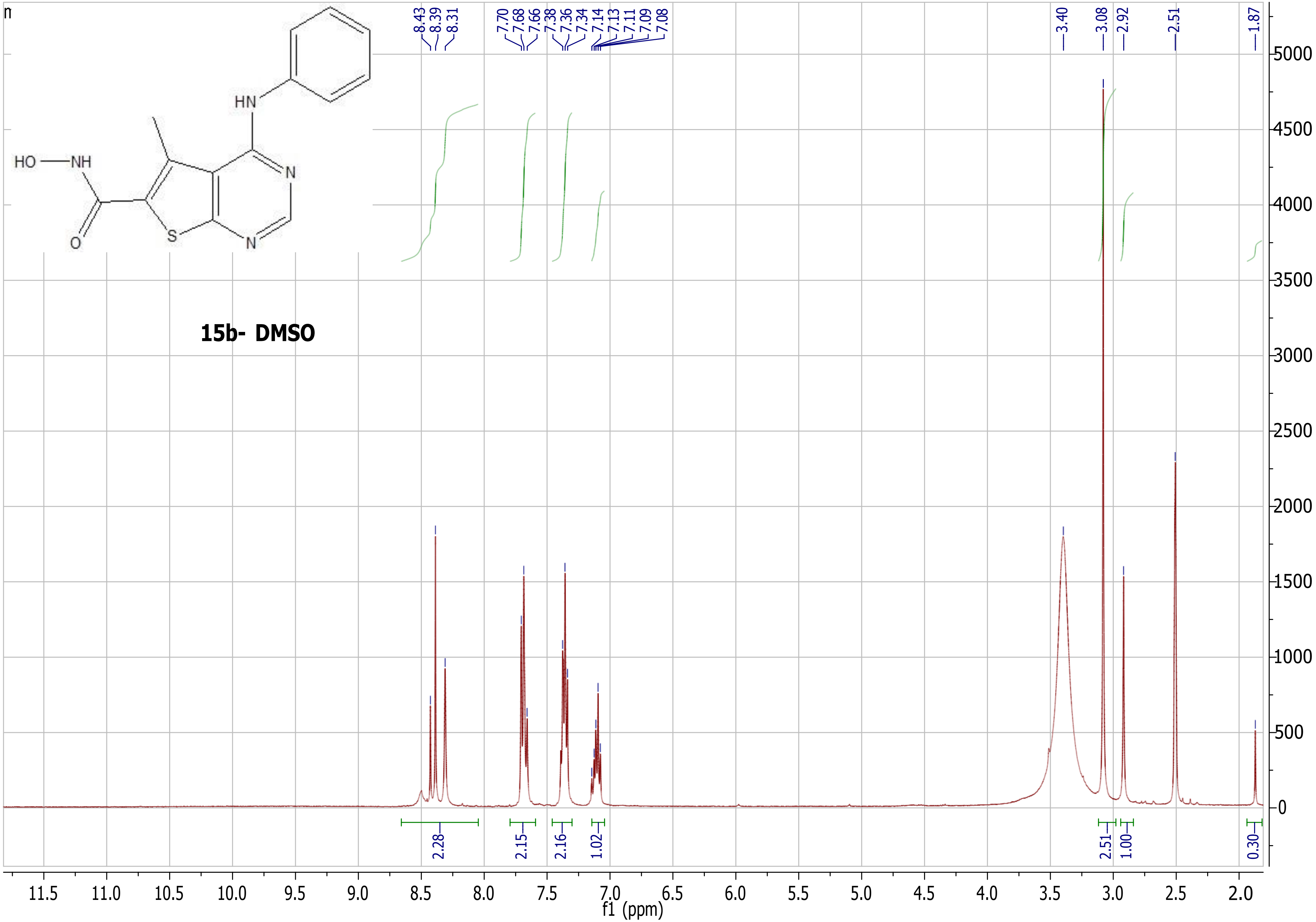

15b- DMSO
HO
NH
O
S
N
N
HN
8.43
8.39
8.31
7.70
7.68
7.66
7.38
7.36
7.34
7.14
7.13
7.11
7.09
7.08
3.40
3.08
2.92
2.51
1.87
2.28
2.15
2.16
1.02
2.51
1.00
0.30
f1 (ppm)

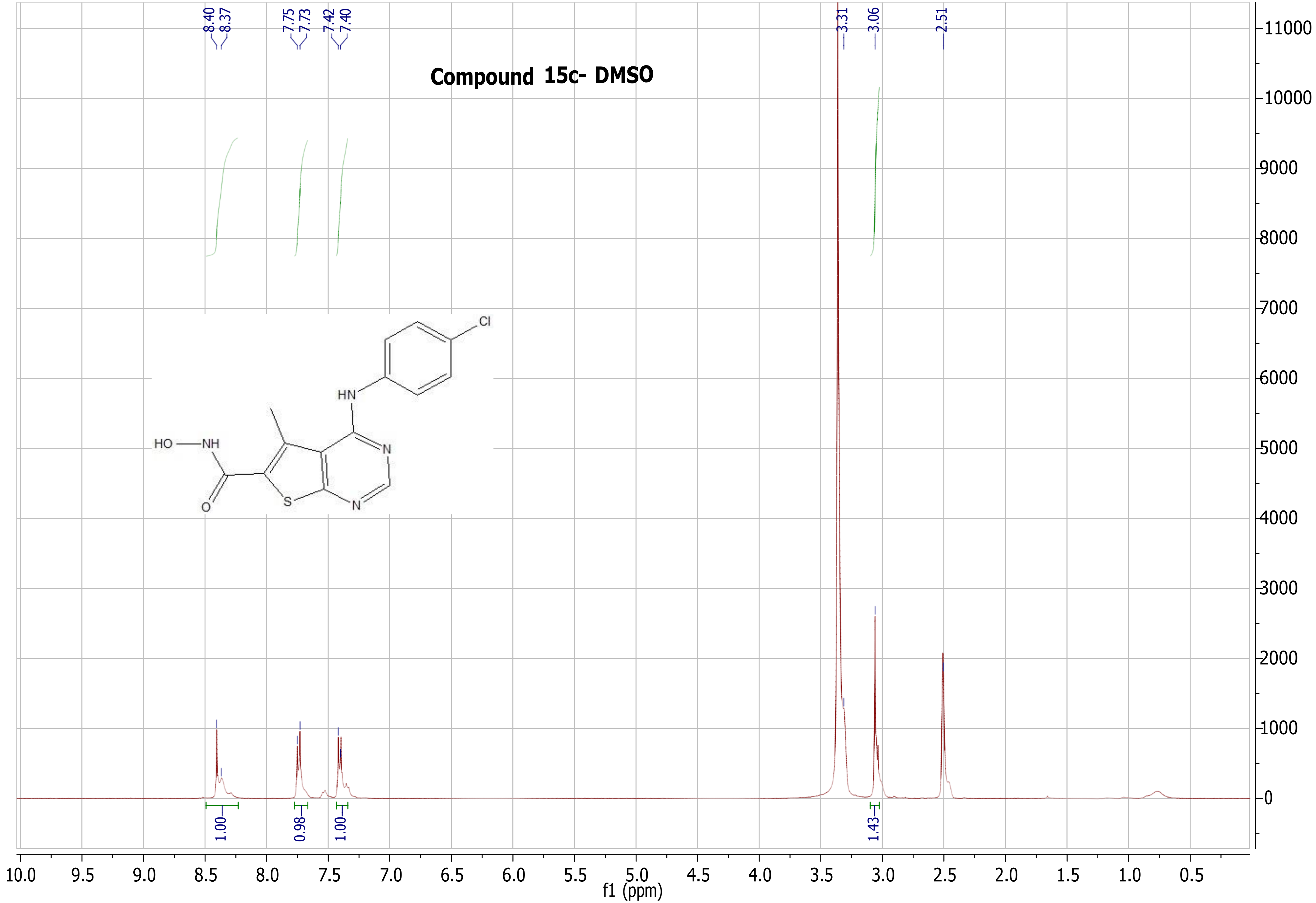

Compound 15c- DMSO
8.40
8.37
7.75
7.73
7.42
7.40
3.31
3.06
2.51
1.00
0.98
1.00
1.43
Cl
HN
HO
NH
N
S
N
O
f1 (ppm)

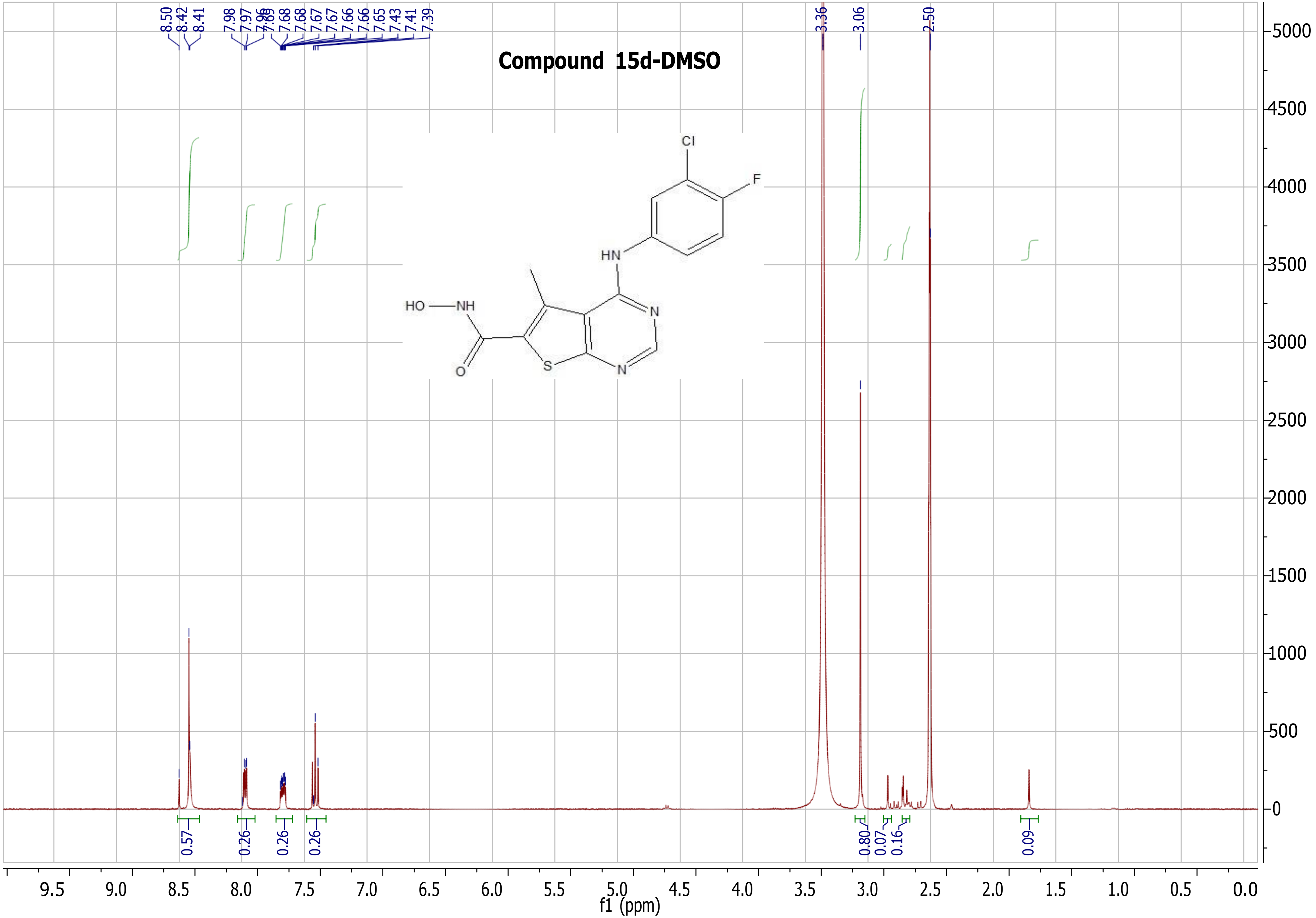

Compound 15d-DMSO
8.50
8.42
8.41
7.98
7.97
7.69
7.68
7.68
7.67
7.67
7.66
7.66
7.65
7.43
7.41
7.39
3.36
3.06
2.50
Cl
F
HN
N
N
S
O
HO
NH
0.57
0.26
0.26
0.26
0.80
0.07
0.16
0.09
f1 (ppm)

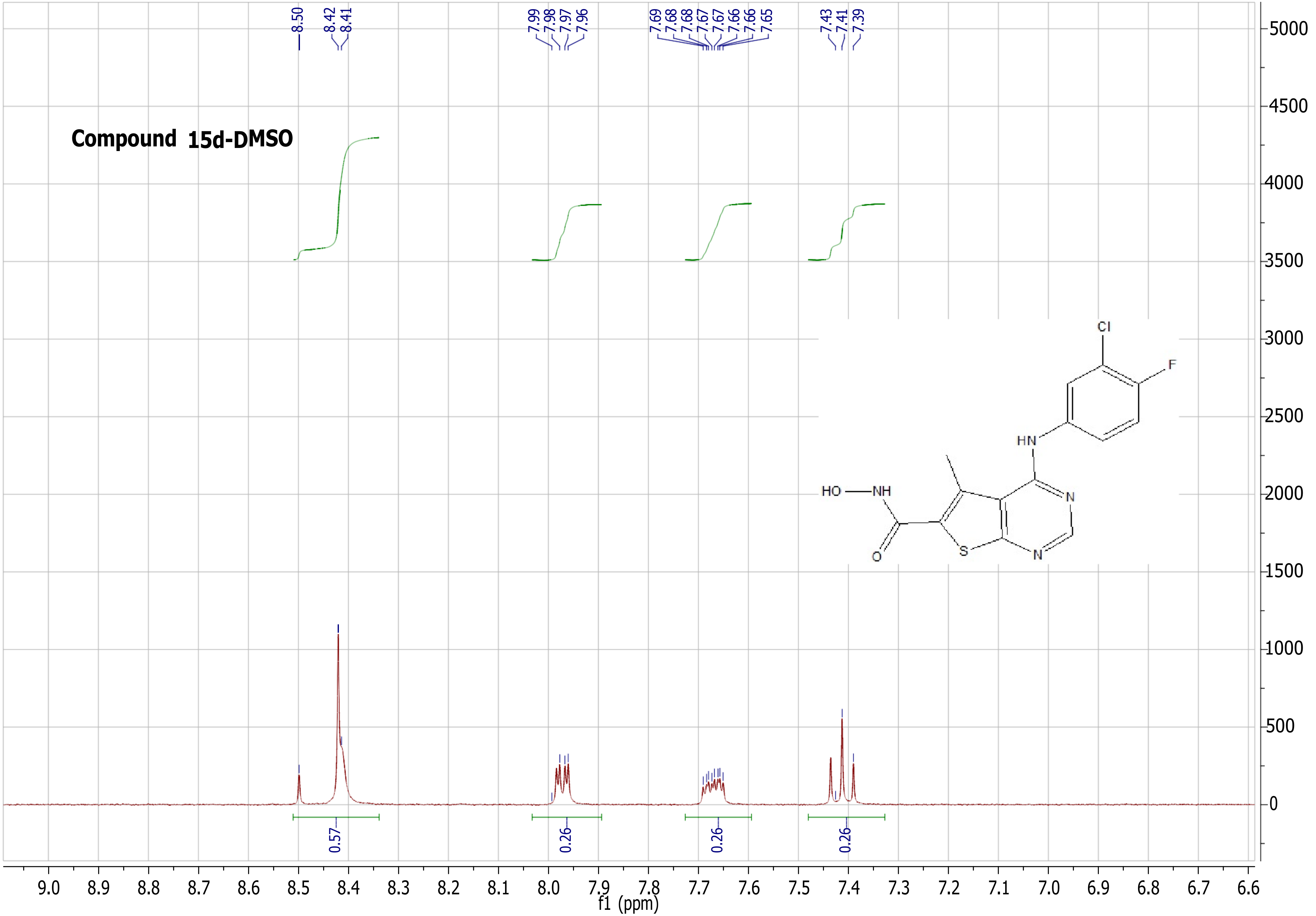
Compound 15d-DMSO
8.50
8.42
8.41
7.99
7.98
7.97
7.96
7.69
7.68
7.68
7.67
7.67
7.66
7.66
7.65
7.43
7.41
7.39
0.57
0.26
0.26
0.26
Cl
F
HN
HO
NH
N
N
S
O
f1 (ppm)

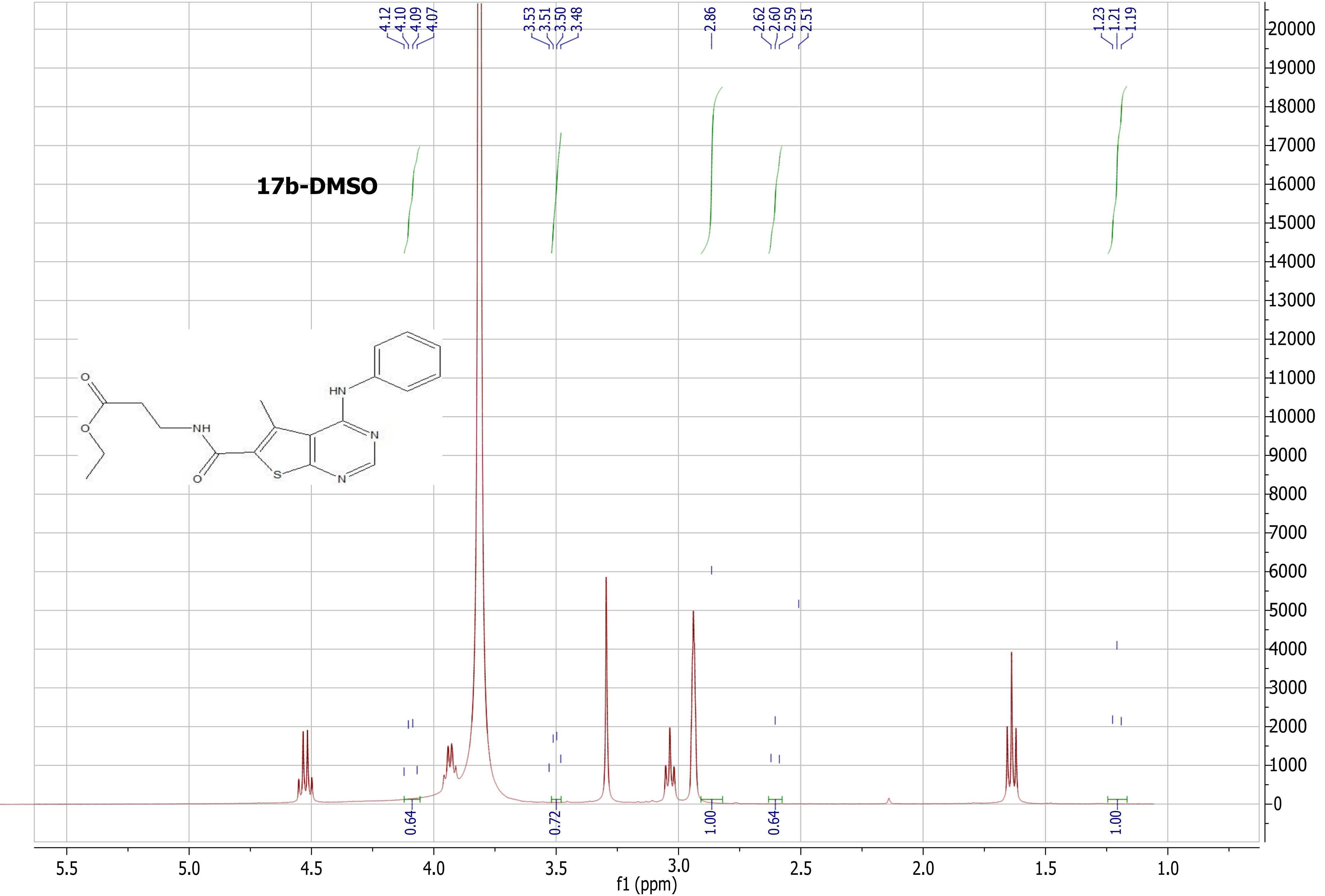
17b-DMSO
4.12
4.10
4.09
4.07
3.53
3.51
3.50
3.48
2.86
2.62
2.60
2.59
2.51
1.23
1.21
1.19
0.64
0.72
1.00
0.64
1.00
f1 (ppm)
HN
NH
N
N
S
O
O
O

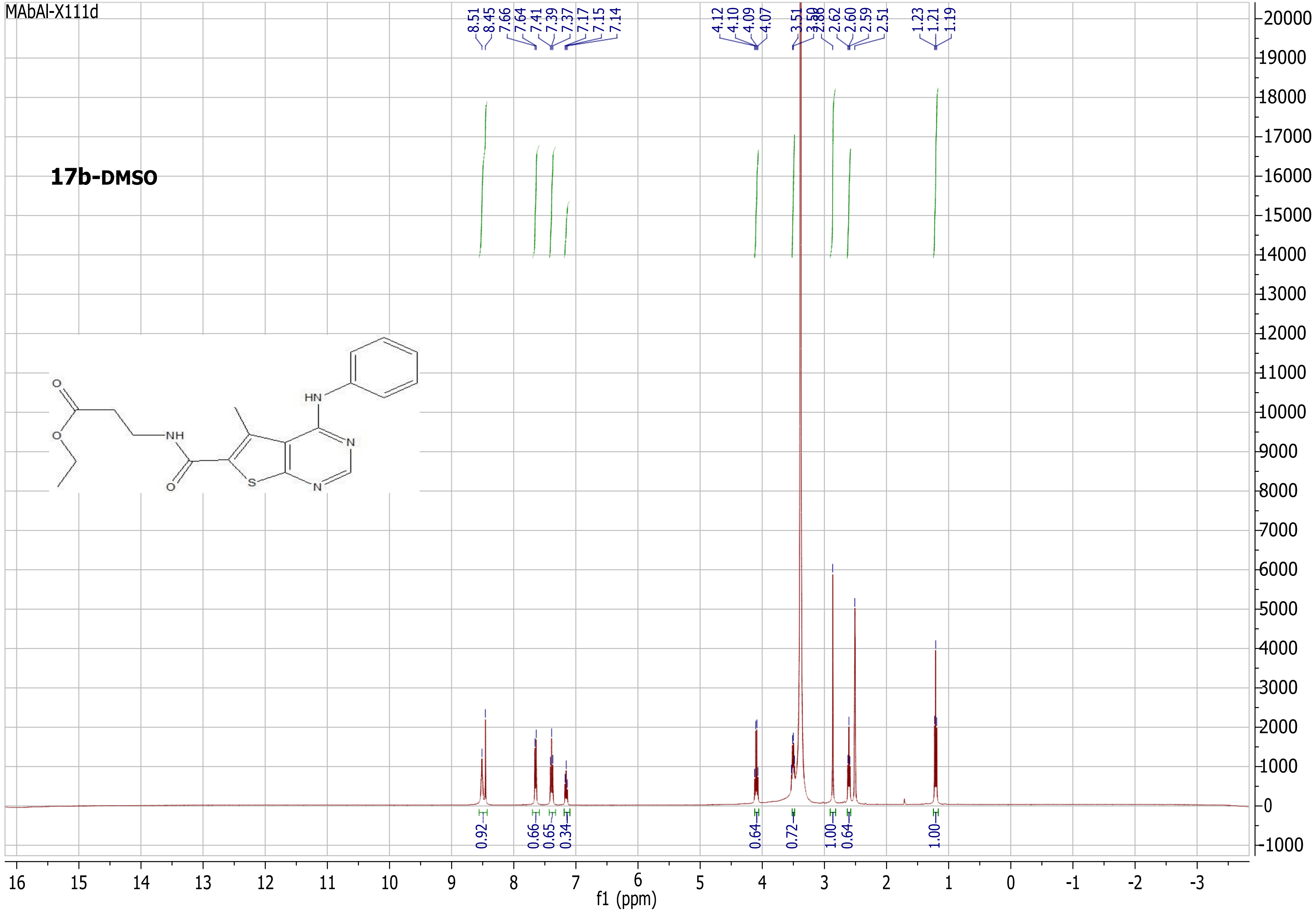

MAbAl-X111d
17b-DMSO
8.51
8.45
7.66
7.64
7.41
7.39
7.37
7.17
7.15
7.14
4.12
4.10
4.09
4.07
3.51
2.62
2.60
2.59
2.51
1.23
1.21
1.19
0.92
0.66
0.65
0.34
0.64
0.72
1.00
0.64
1.00
HN
NH
N
N
S
O
O
O
f1 (ppm)

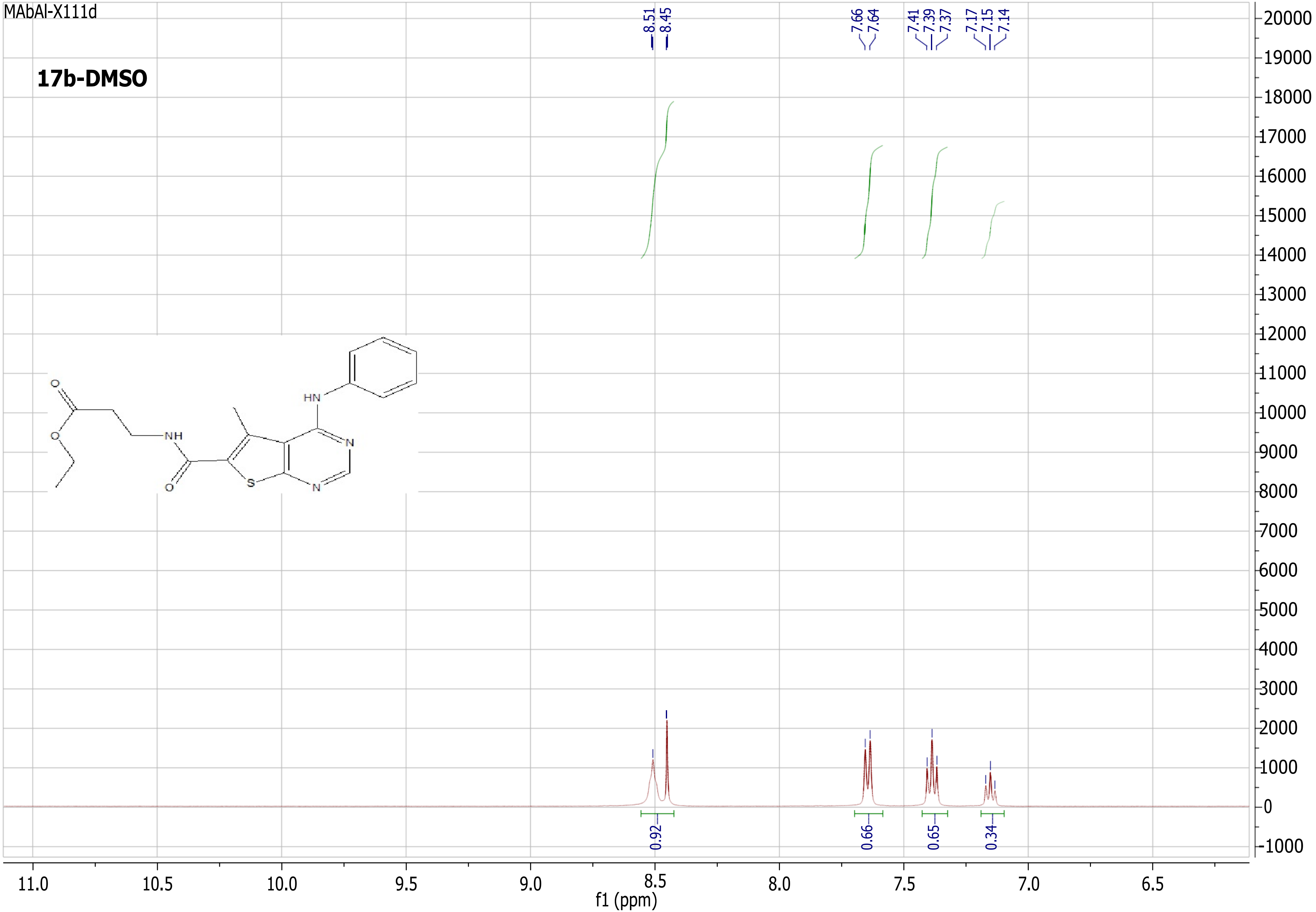

MAbAI-X111d
17b-DMSO
8.51
8.45
7.66
7.64
7.41
7.39
7.37
7.17
7.15
7.14
0.92
0.66
0.65
0.34
O
O
NH
O
S
HN
N
N
f1 (ppm)

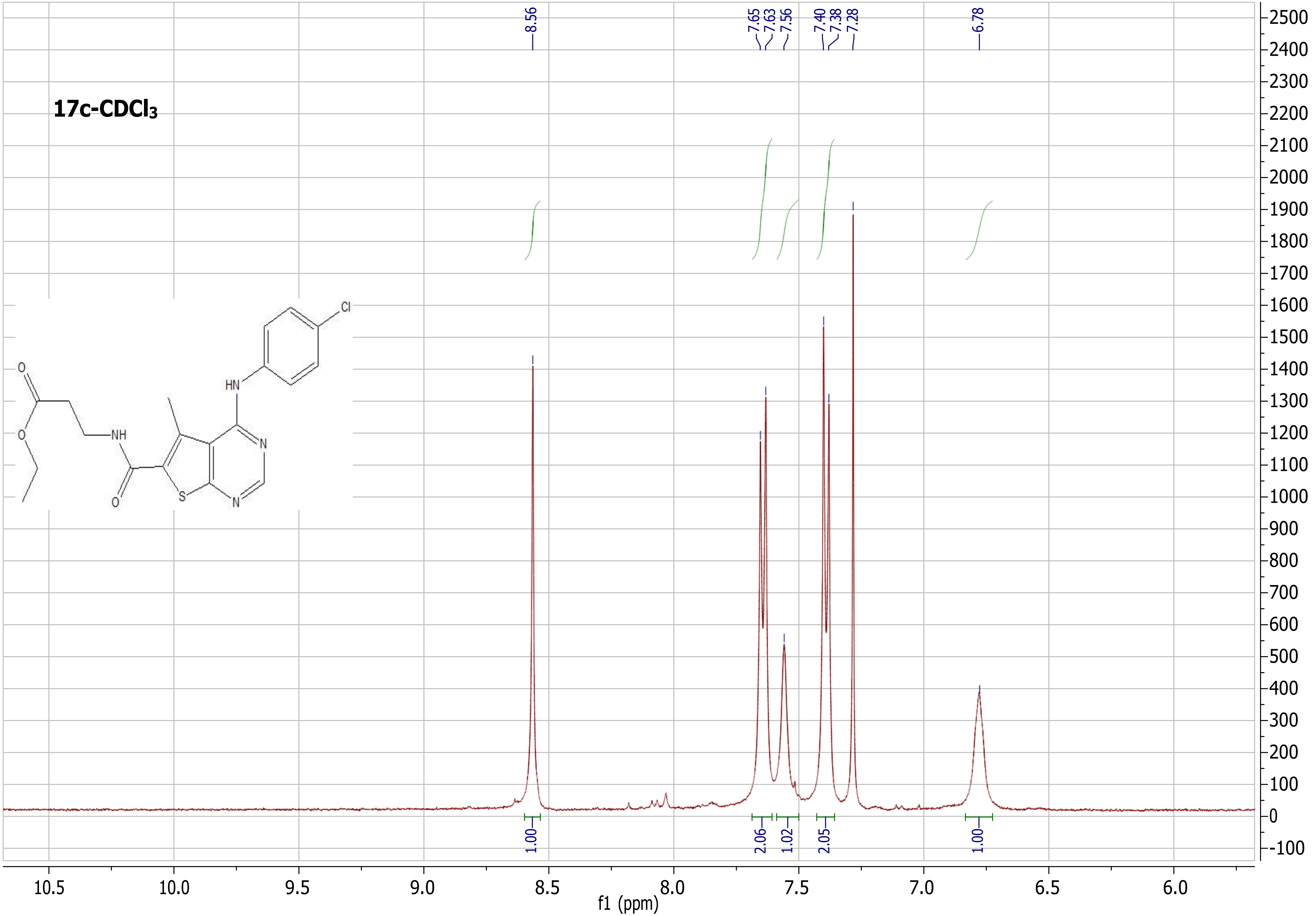

17c-CDCl3
8.56
7.65
7.63
7.56
7.40
7.38
7.28
6.78
1.00
2.06
1.02
2.05
1.00
f1 (ppm)
HN
Cl
NH
N
S
O

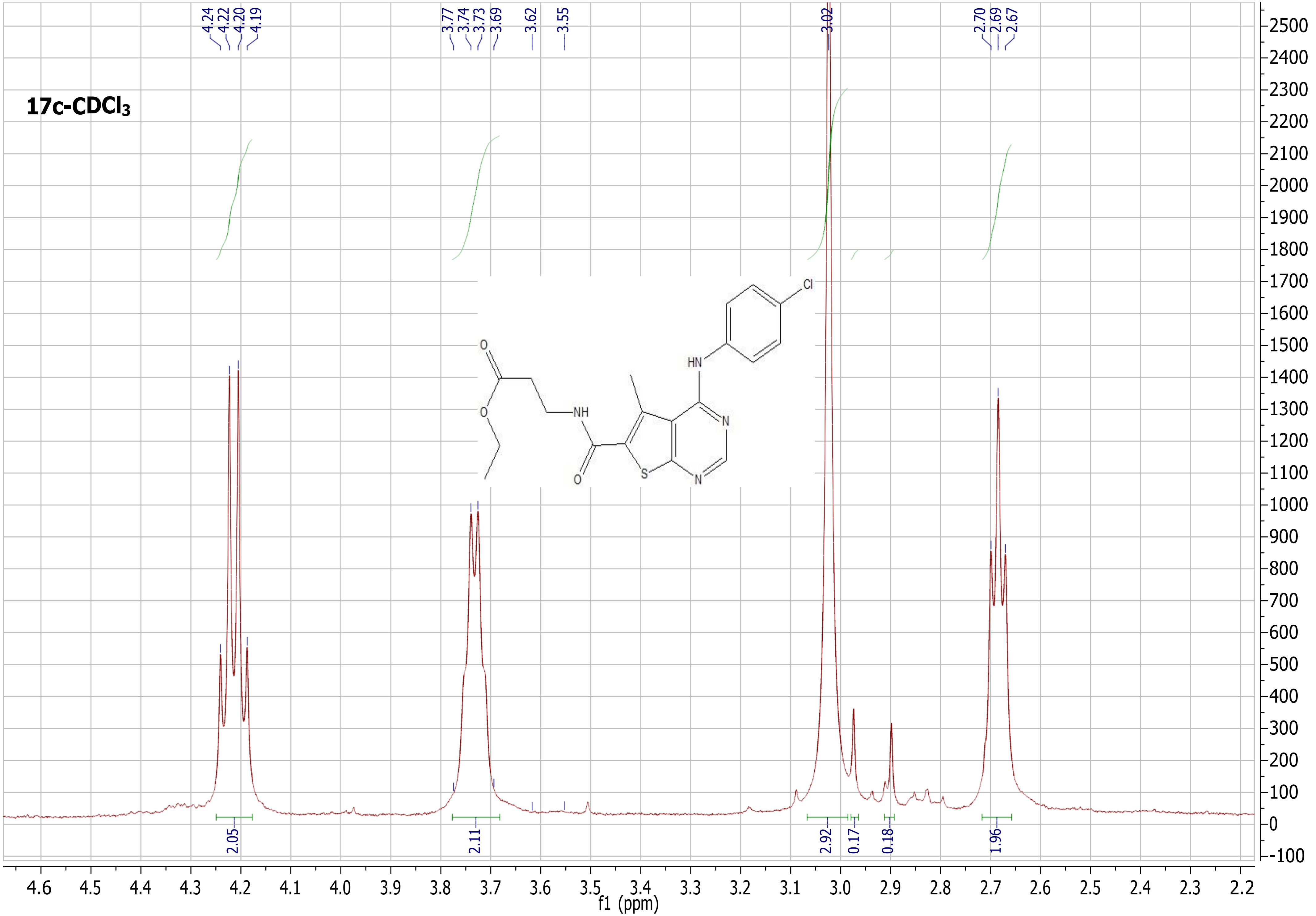

17c-CDCl3
4.24
4.22
4.20
4.19
3.77
3.74
3.73
3.69
3.62
3.55
3.02
2.70
2.69
2.67
2.05
2.11
2.92
0.17
0.18
1.96
f1 (ppm)
Cl
HN
N
N
S
O
NH
O
O

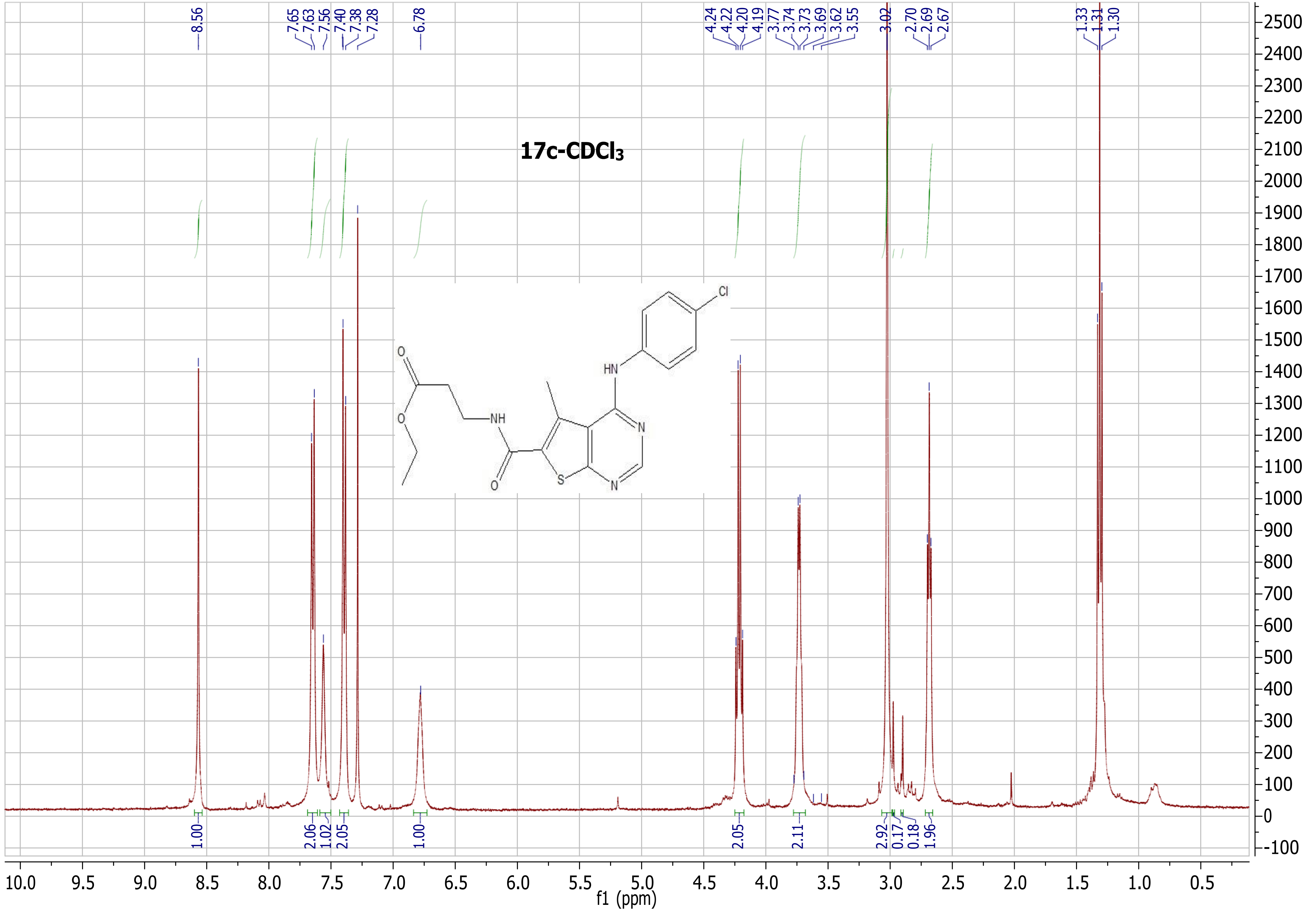
17c-CDCl3
8.56
7.65
7.63
7.56
7.40
7.38
7.28
6.78
4.24
4.22
4.20
4.19
3.77
3.74
3.73
3.69
3.62
3.55
3.02
2.70
2.69
2.67
1.33
1.31
1.30
1.00
2.06
1.02
2.05
1.00
2.05
2.11
2.92
0.17
0.18
1.96
f1 (ppm)
Cl
HN
O
O
NH
N
N
S
O

17d-CDCl$_3$

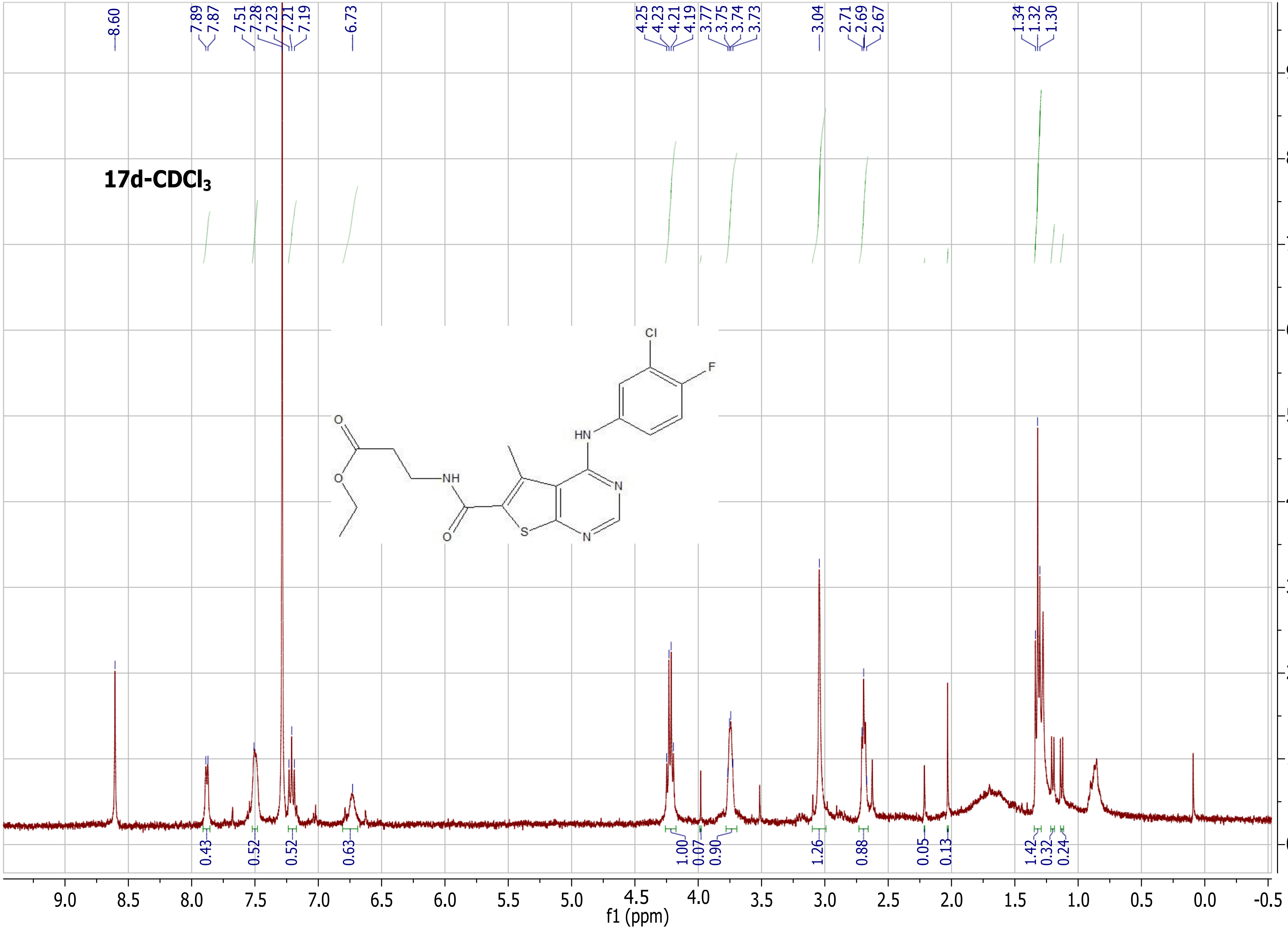

8.60
7.89
7.87
7.51
7.28
7.23
7.21
7.19
6.73
4.25
4.23
4.21
4.19
3.77
3.75
3.74
3.73
3.04
2.71
2.69
2.67
1.34
1.32
1.30
Cl
F
HN
O
O
NH
N
S
N
O
0.43
0.52
0.52
0.63
1.00
0.07
0.90
1.26
0.88
0.05
0.13
1.42
0.32
0.24
9.0
8.5
8.0
7.5
7.0
6.5
6.0
5.5
5.0
4.5
4.0
3.5
3.0
2.5
2.0
1.5
1.0
0.5
0.0
-0.5
f1 (ppm)
900
800
700
600
500
400
300
200
100
0

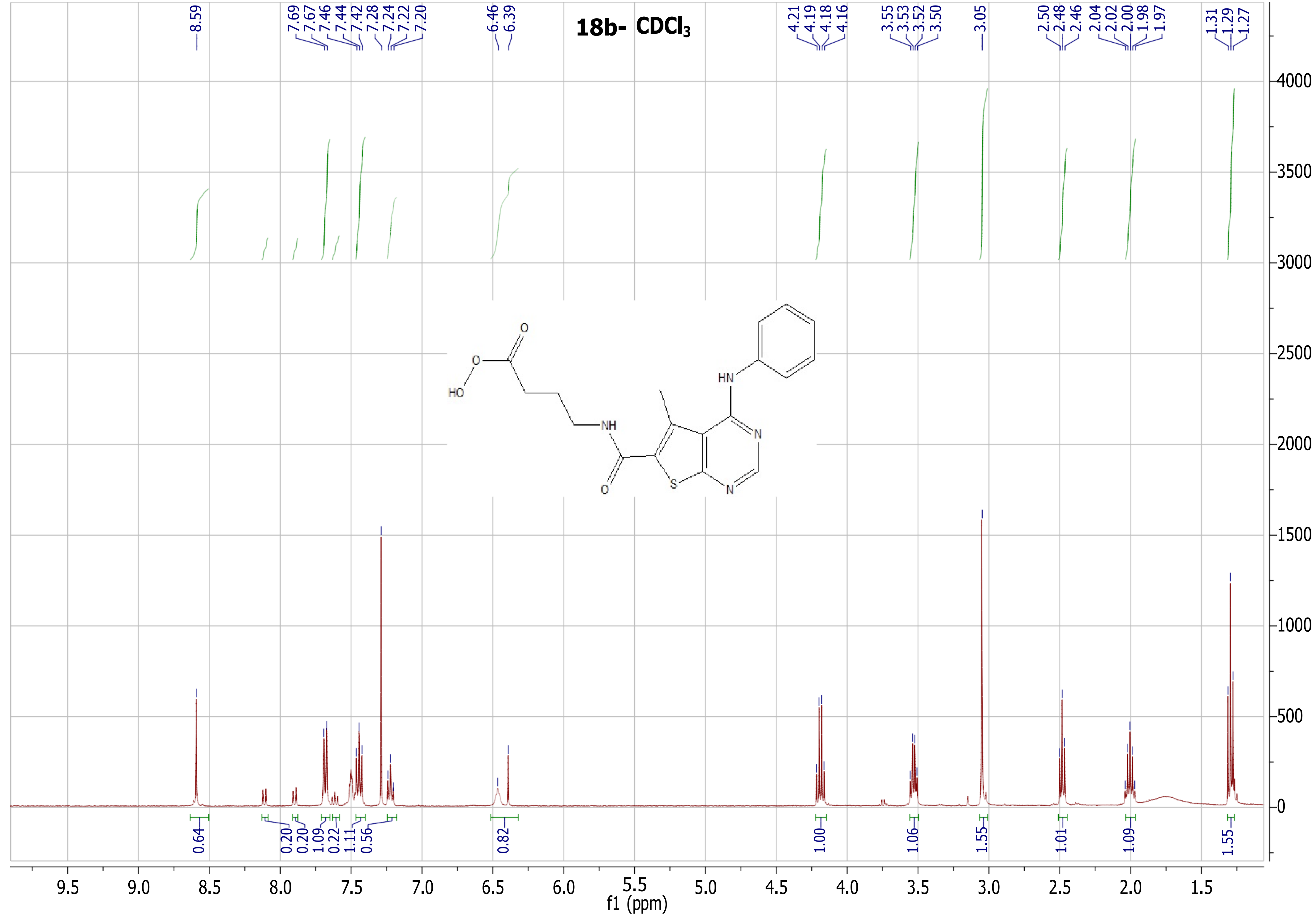

18b- CDCl3
8.59
7.69
7.67
7.46
7.44
7.42
7.28
7.24
7.22
7.20
6.46
6.39
4.21
4.19
4.18
4.16
3.55
3.53
3.52
3.50
3.05
2.50
2.48
2.46
2.04
2.02
2.00
1.98
1.97
1.31
1.29
1.27
0.64
0.20
0.20
1.09
0.22
1.11
0.56
0.82
1.00
1.06
1.55
1.01
1.09
1.55
f1 (ppm)

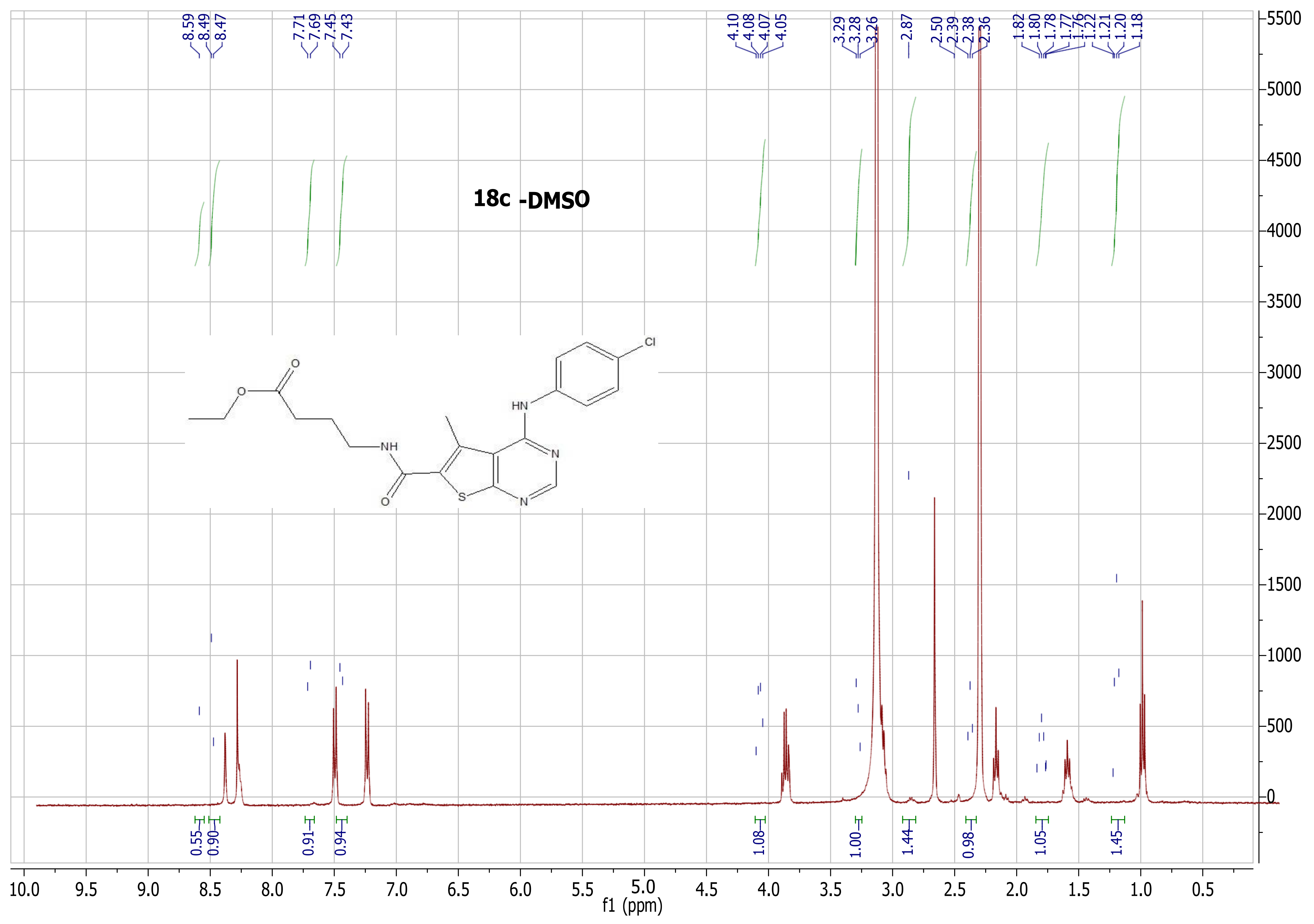

18c -DMSO
8.59
8.49
8.47
7.71
7.69
7.45
7.43
4.10
4.08
4.07
4.05
3.29
3.28
3.26
2.87
2.50
2.39
2.38
2.36
1.82
1.80
1.78
1.77
1.76
1.22
1.21
1.20
1.18
0.55
0.90
0.91
0.94
1.08
1.00
1.44
0.98
1.05
1.45
f1 (ppm)
HN
NH
N
S
O
Cl

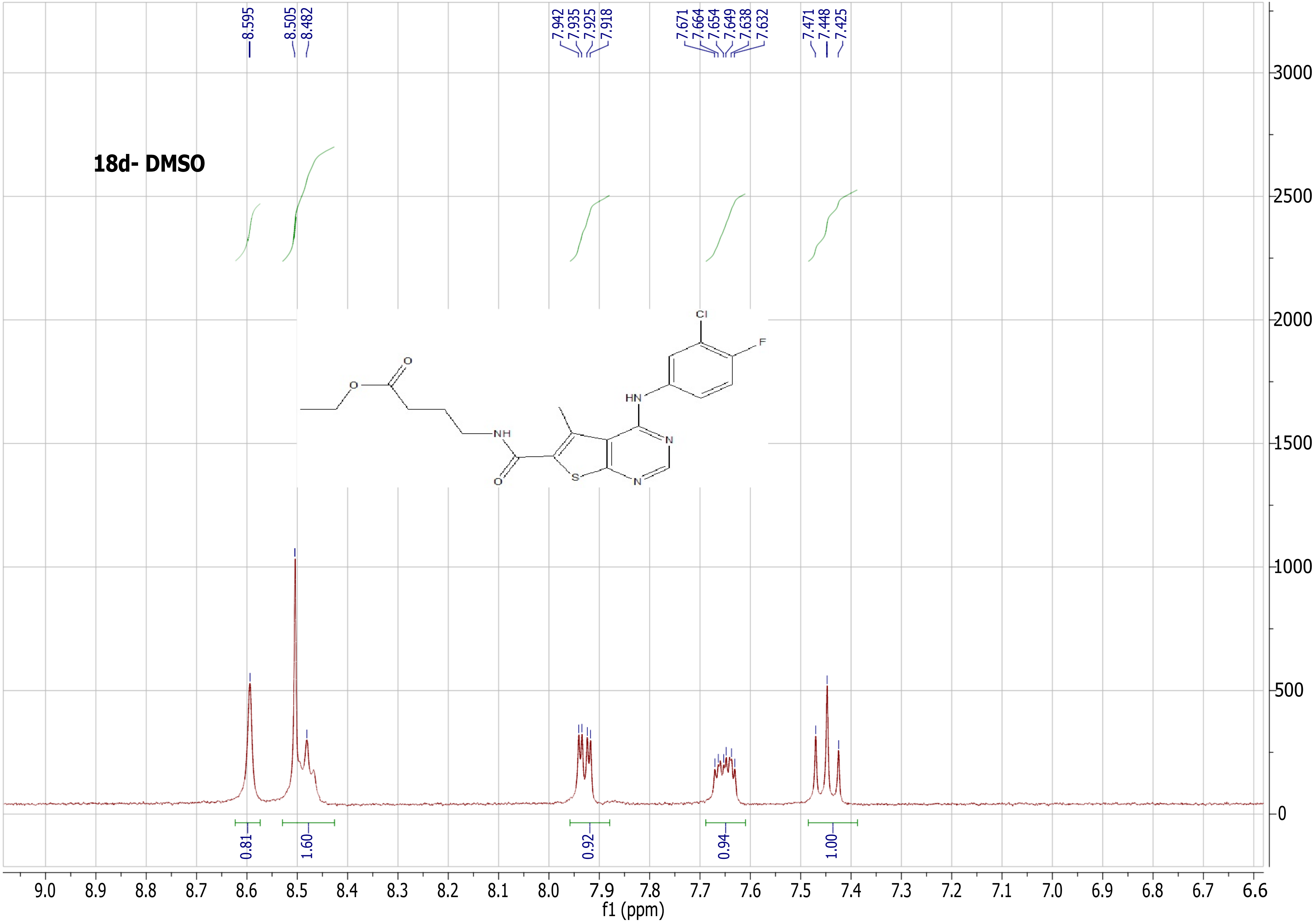
18d- DMSO
8.595
8.505
8.482
7.942
7.935
7.925
7.918
7.671
7.664
7.654
7.649
7.638
7.632
7.471
7.448
7.425
0.81
1.60
0.92
0.94
1.00
Cl
F
HN
N
N
S
O
O
O
NH
f1 (ppm)

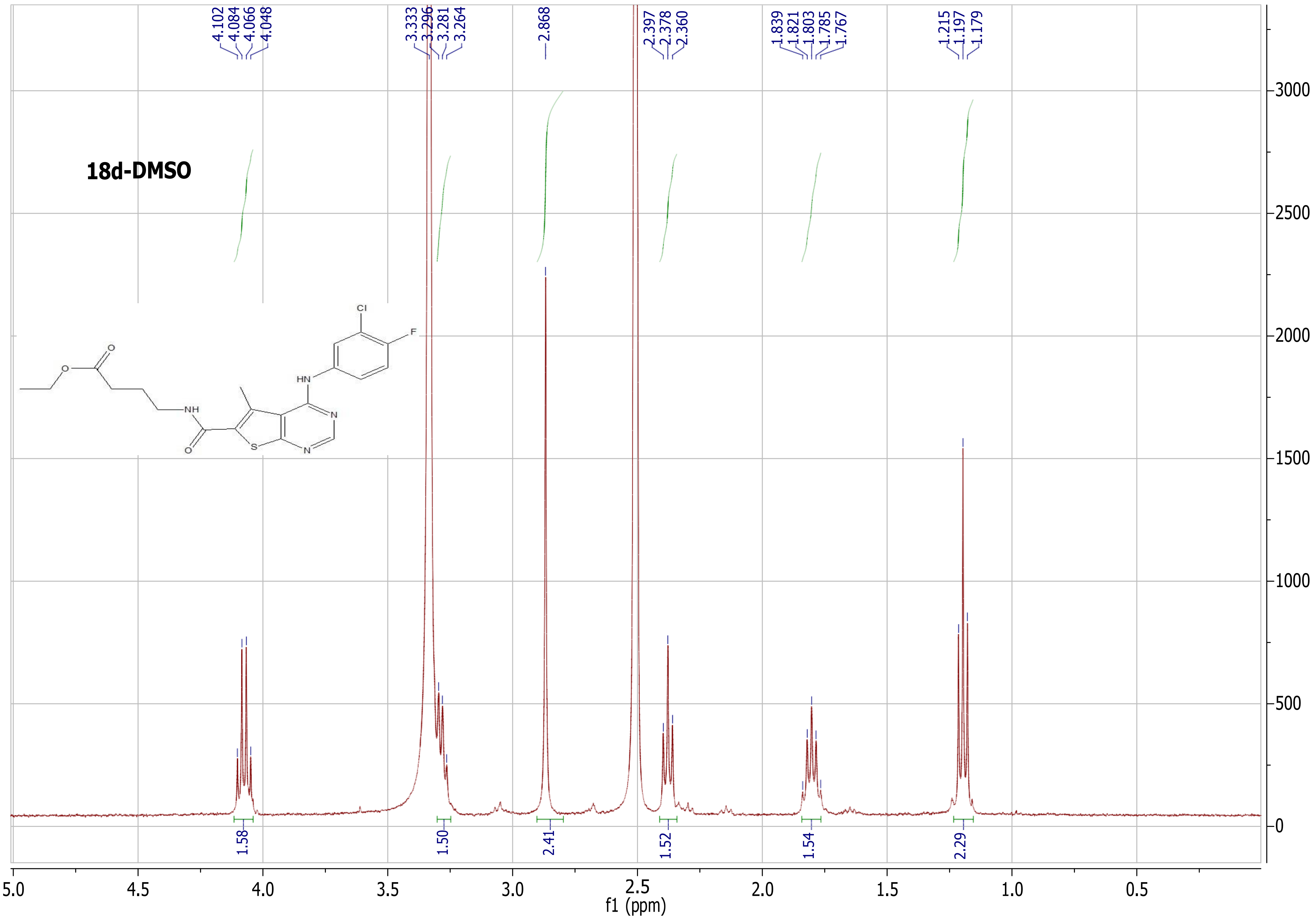

18d-DMSO
4.102
4.084
4.066
4.048
3.333
3.296
3.281
3.264
2.868
2.397
2.378
2.360
1.839
1.821
1.803
1.785
1.767
1.215
1.197
1.179
1.58
1.50
2.41
1.52
1.54
2.29
5.0
4.5
4.0
3.5
3.0
2.5
2.0
1.5
1.0
0.5
f1 (ppm)
3000
2500
2000
1500
1000
500
0

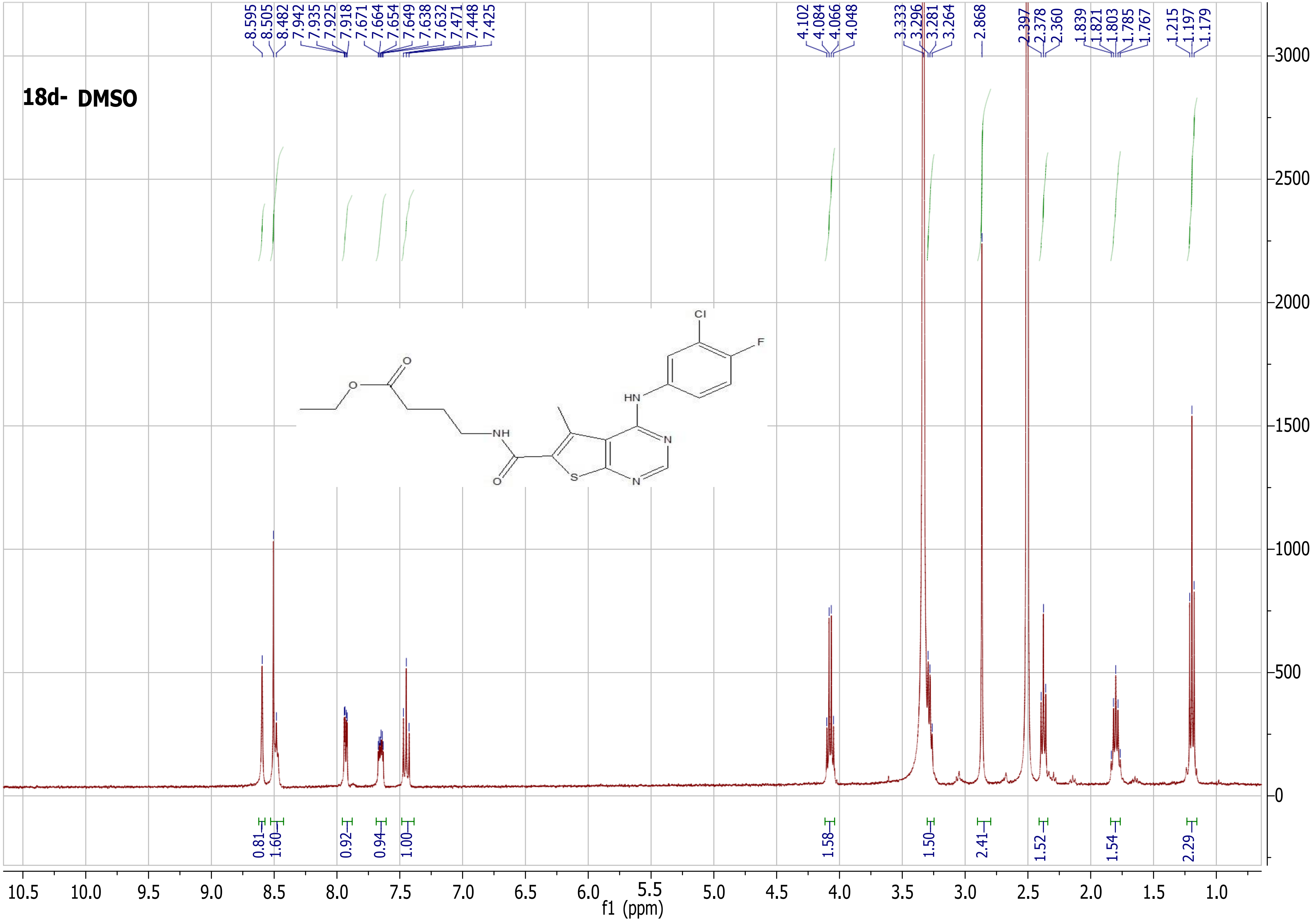

18d- DMSO
8.595
8.505
8.482
7.942
7.935
7.925
7.918
7.671
7.664
7.654
7.649
7.638
7.632
7.471
7.448
7.425
4.102
4.084
4.066
4.048
3.333
3.296
3.281
3.264
2.868
2.397
2.378
2.360
1.839
1.821
1.803
1.785
1.767
1.215
1.197
1.179
Cl
F
HN
N
N
S
NH
O
O
O
0.81
1.60
0.92
0.94
1.00
1.58
1.50
2.41
1.52
1.54
2.29
10.5
10.0
9.5
9.0
8.5
8.0
7.5
7.0
6.5
6.0
5.5
5.0
4.5
4.0
3.5
3.0
2.5
2.0
1.5
1.0
f1 (ppm)
3000
2500
2000
1500
1000
500
0

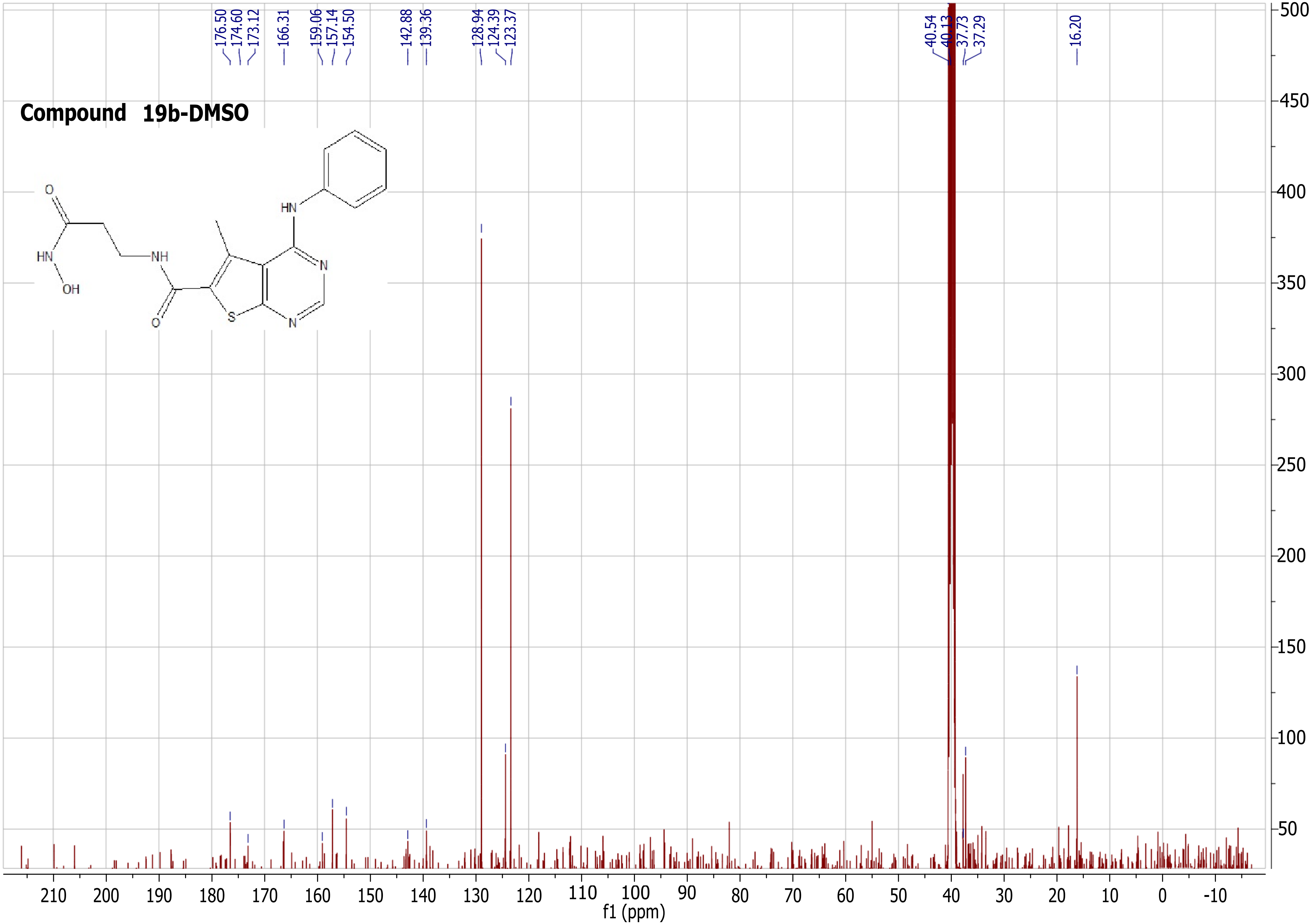
Compound 19b-DMSO
176.50
174.60
173.12
166.31
159.06
157.14
154.50
142.88
139.36
128.94
124.39
123.37
40.54
40.13
37.73
37.29
16.20
HN
NH
OH
O
N
S
f1 (ppm)

Compound 19b in DMSO

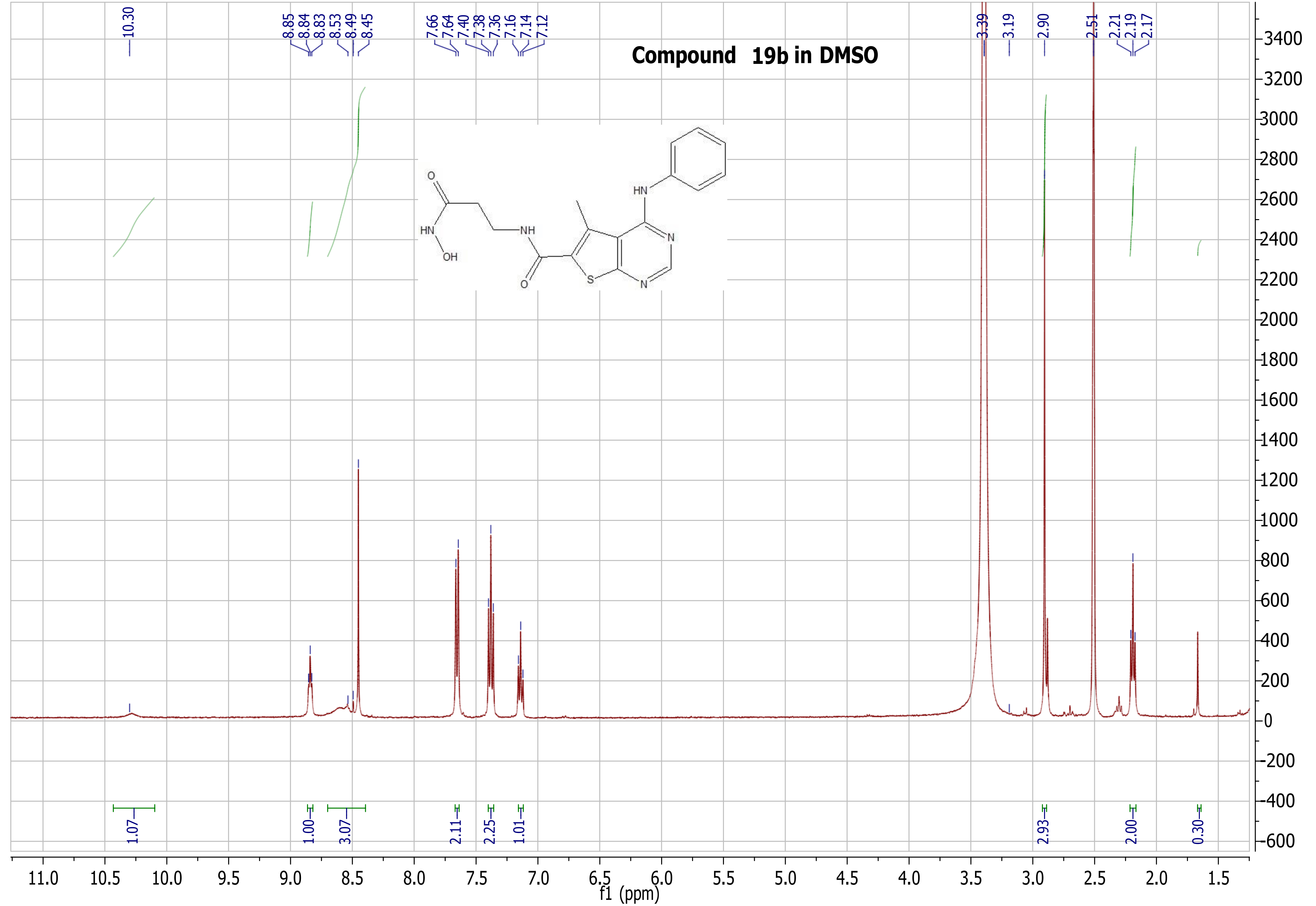

Compound 19b-d2O in DMSO

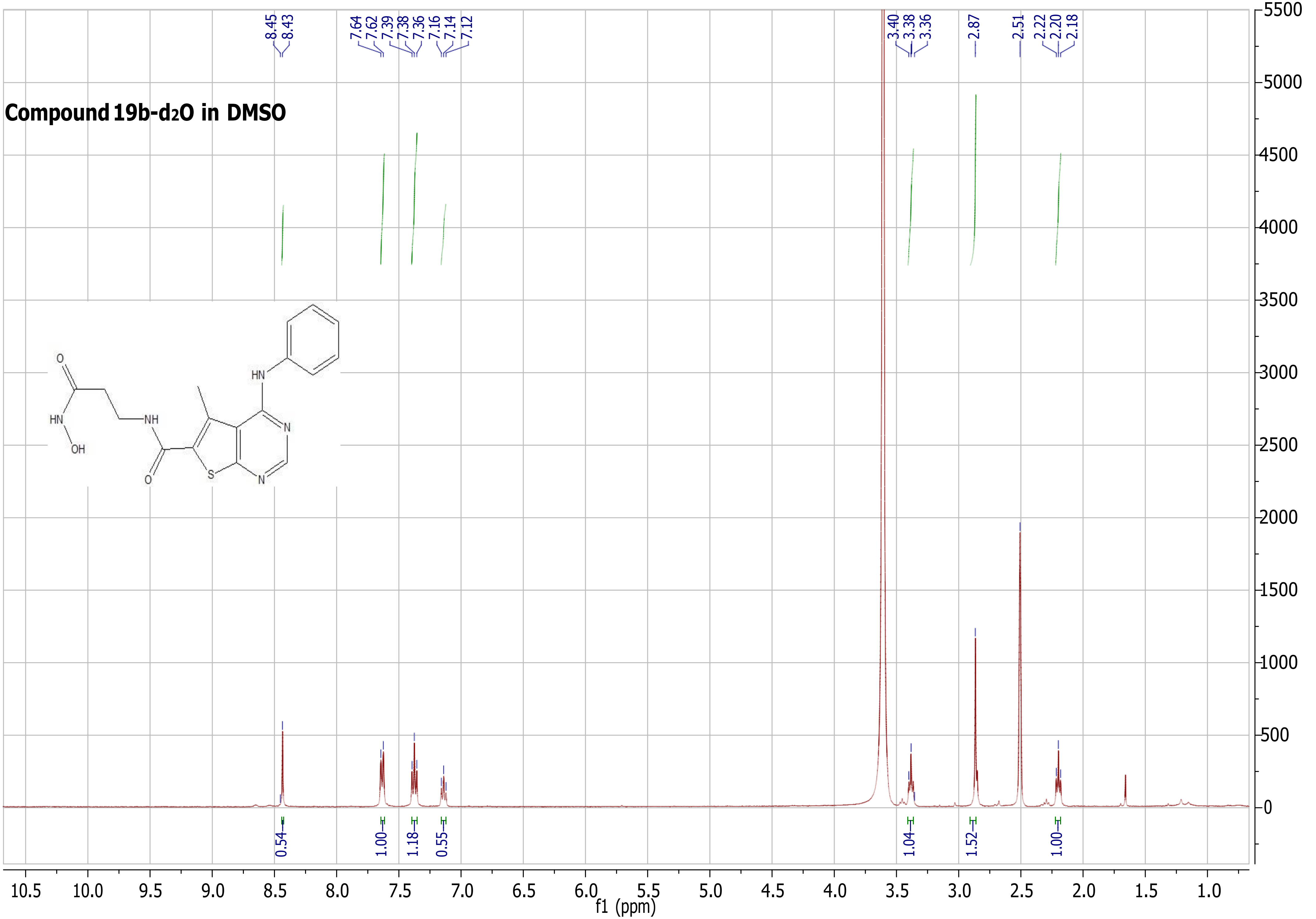

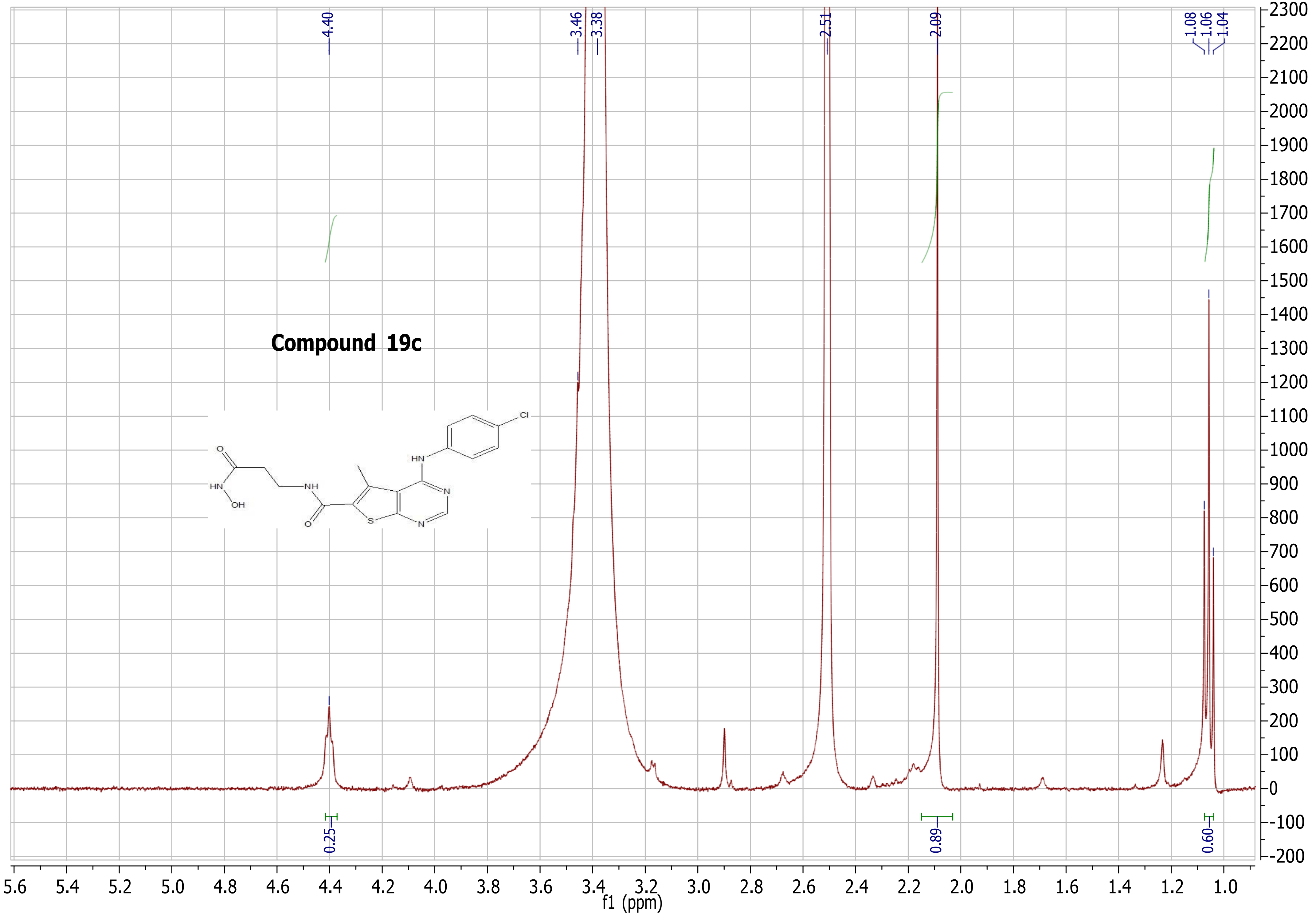
Compound 19c
4.40
3.46
3.38
2.51
2.09
1.08
1.06
1.04
0.25
0.89
0.60
HN
Cl
O
NH
HN
OH
N
S
N
O
f1 (ppm)

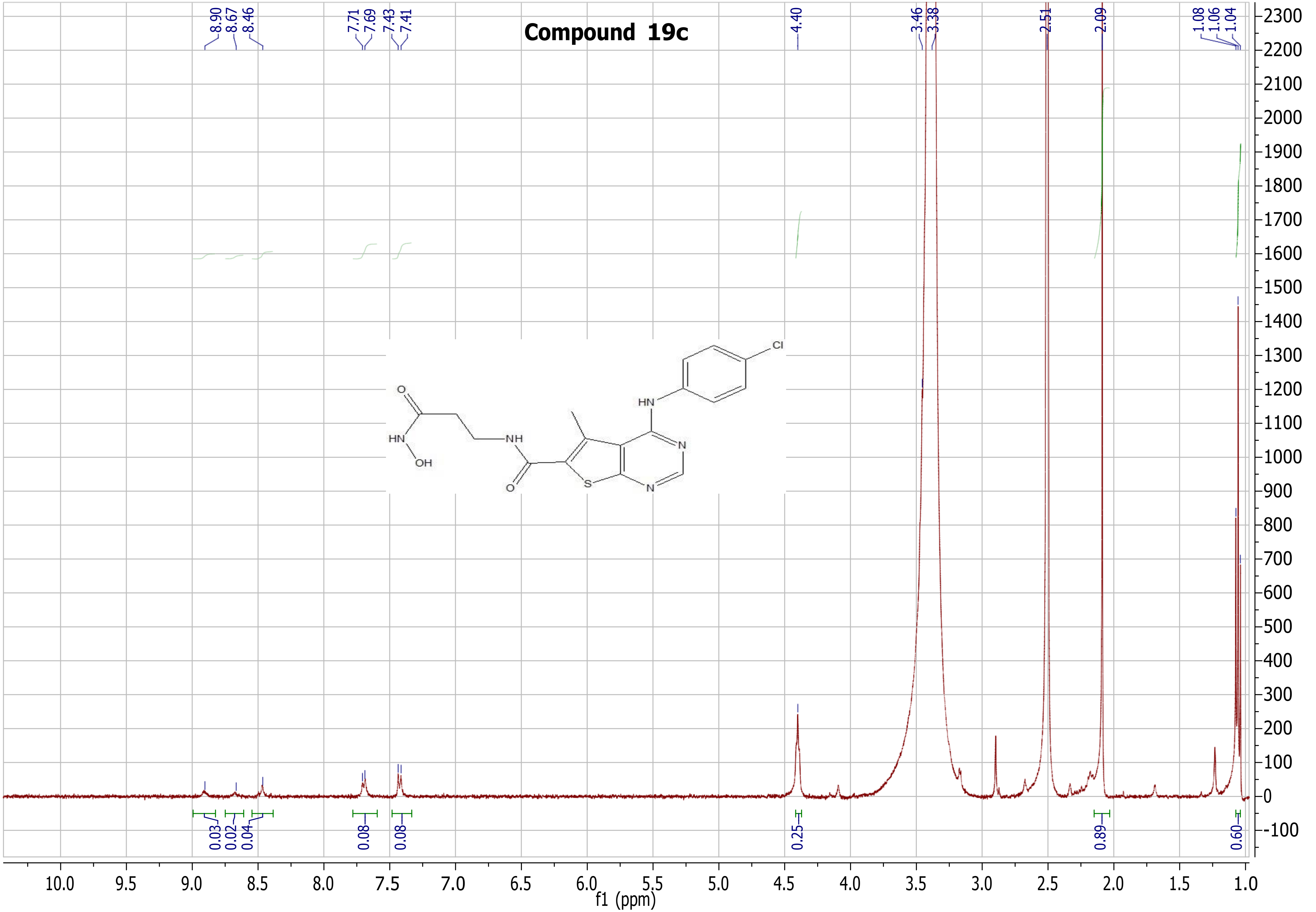

Compound 19c
8.90
8.67
8.46
7.71
7.69
7.43
7.41
4.40
3.46
3.38
2.51
2.09
1.08
1.06
1.04
0.03
0.02
0.04
0.08
0.08
0.25
0.89
0.60
O
HN
OH
NH
O
S
N
N
HN
Cl
f1 (ppm)

19d

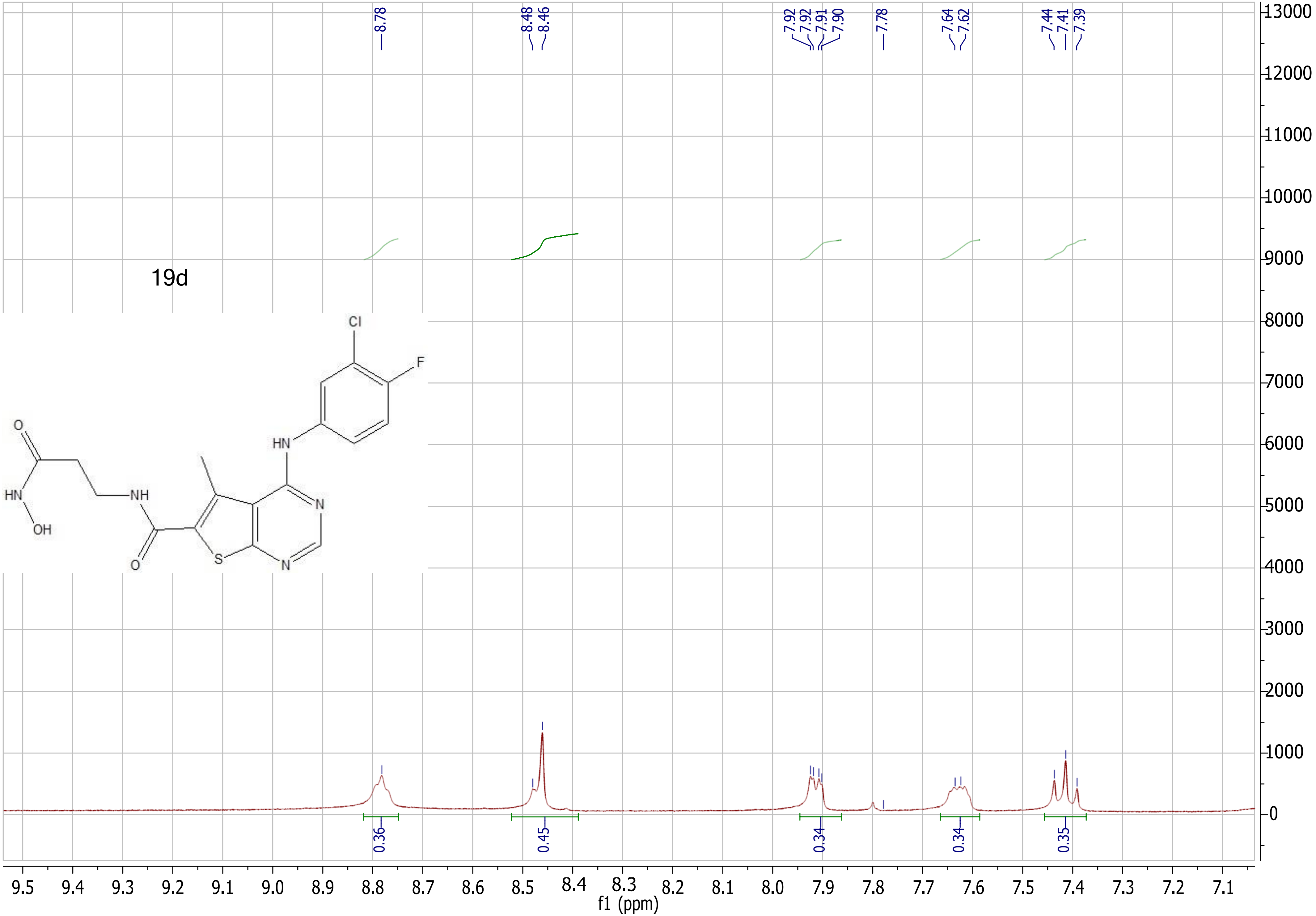
Cl
F
HN
O
HN
OH
NH
N
N
S
O
8.78
8.48
8.46
7.92
7.92
7.91
7.90
7.78
7.64
7.62
7.44
7.41
7.39
0.36
0.45
0.34
0.34
0.35
f1 (ppm)

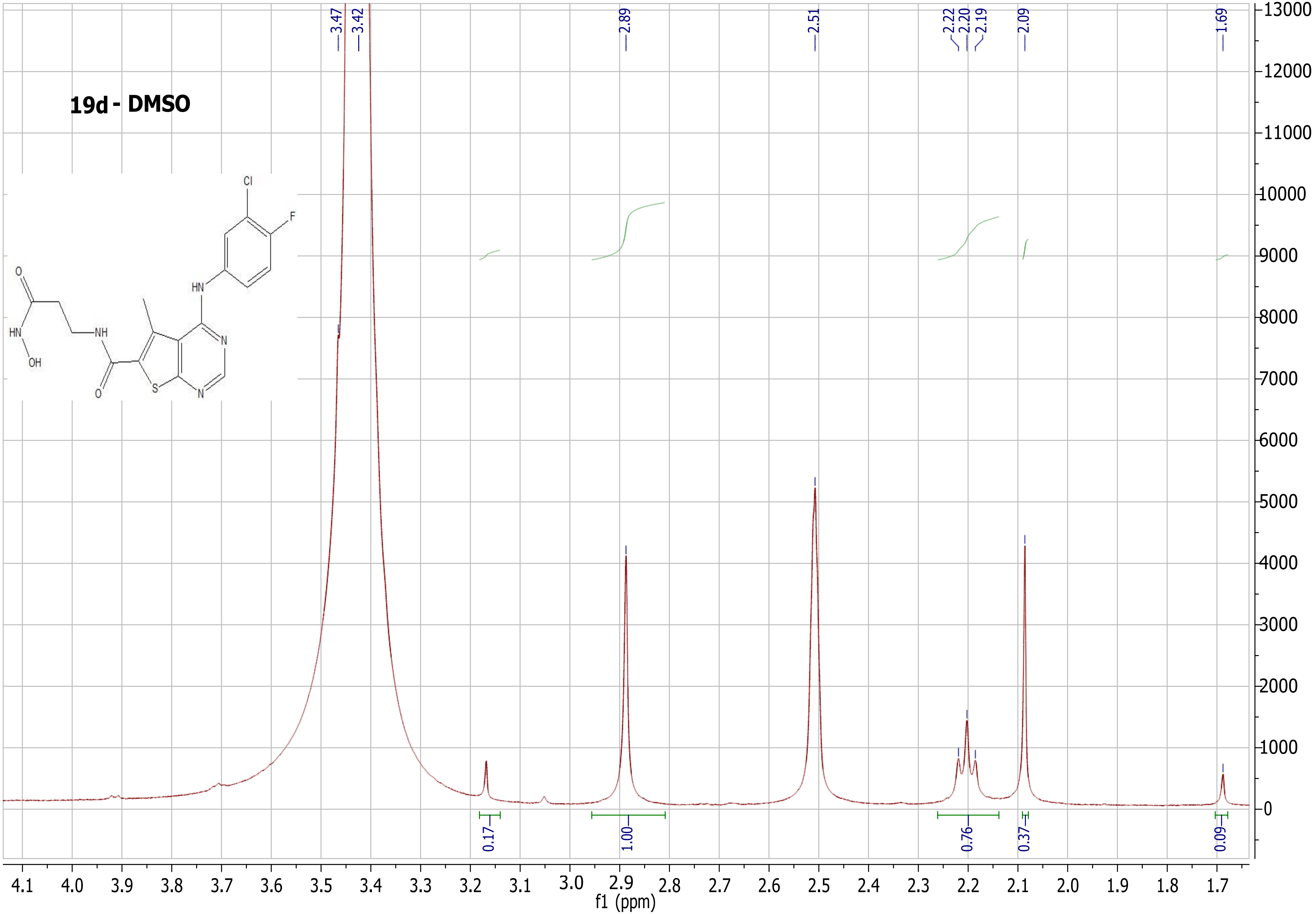

19d - DMSO
3.47
3.42
2.89
2.51
2.22
2.20
2.19
2.09
1.69
0.17
1.00
0.76
0.37
0.09
Cl
F
HN
O
HN
OH
NH
O
S
N
N
f1 (ppm)

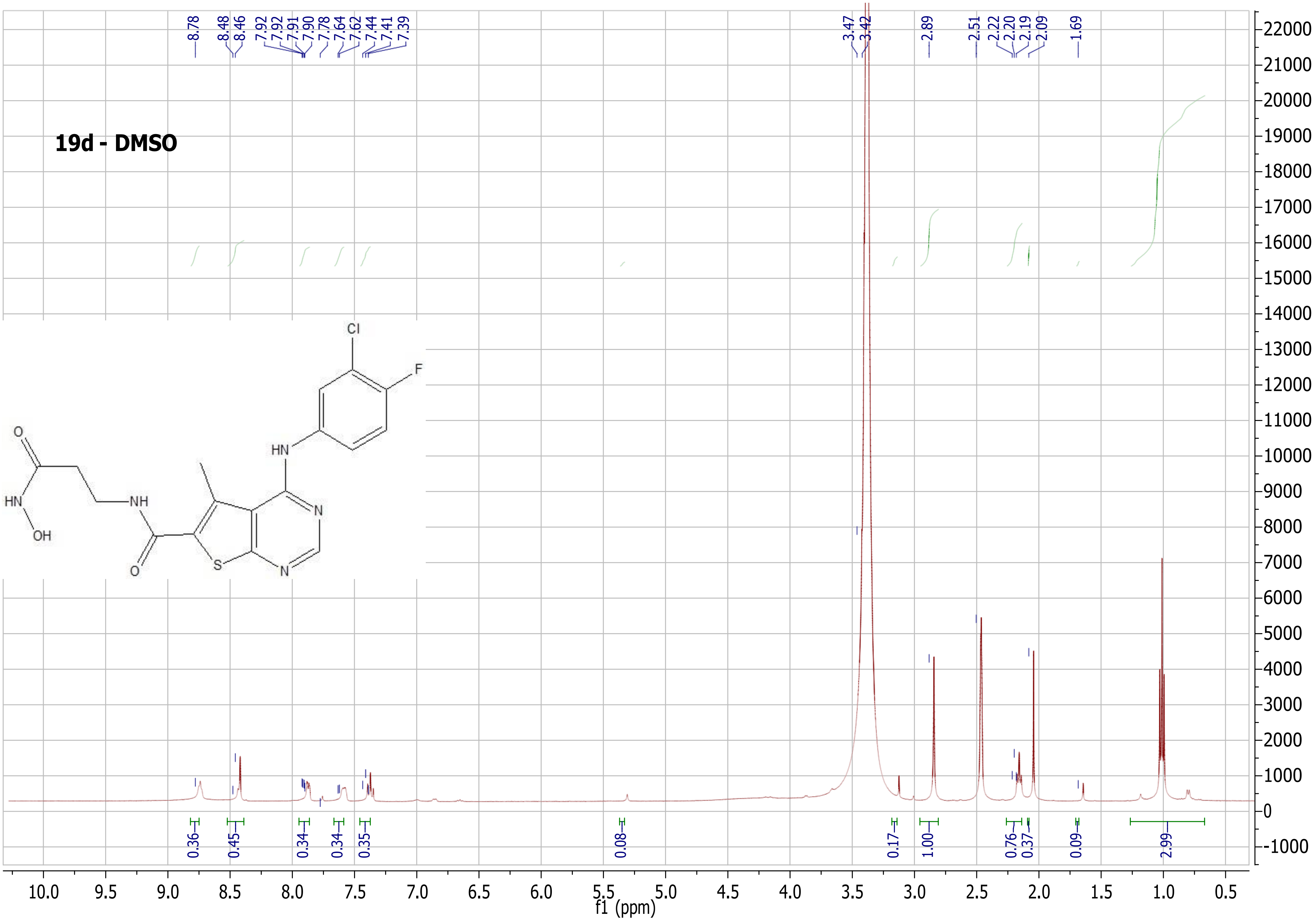

19d - DMSO
8.78
8.48
8.46
7.92
7.92
7.91
7.90
7.78
7.64
7.62
7.44
7.41
7.39
3.47
3.42
2.89
2.51
2.22
2.20
2.19
2.09
1.69
0.36
0.45
0.34
0.34
0.35
0.08
0.17
1.00
0.76
0.37
0.09
2.99
Cl
F
HN
O
HN
OH
NH
N
N
S
O
f1 (ppm)

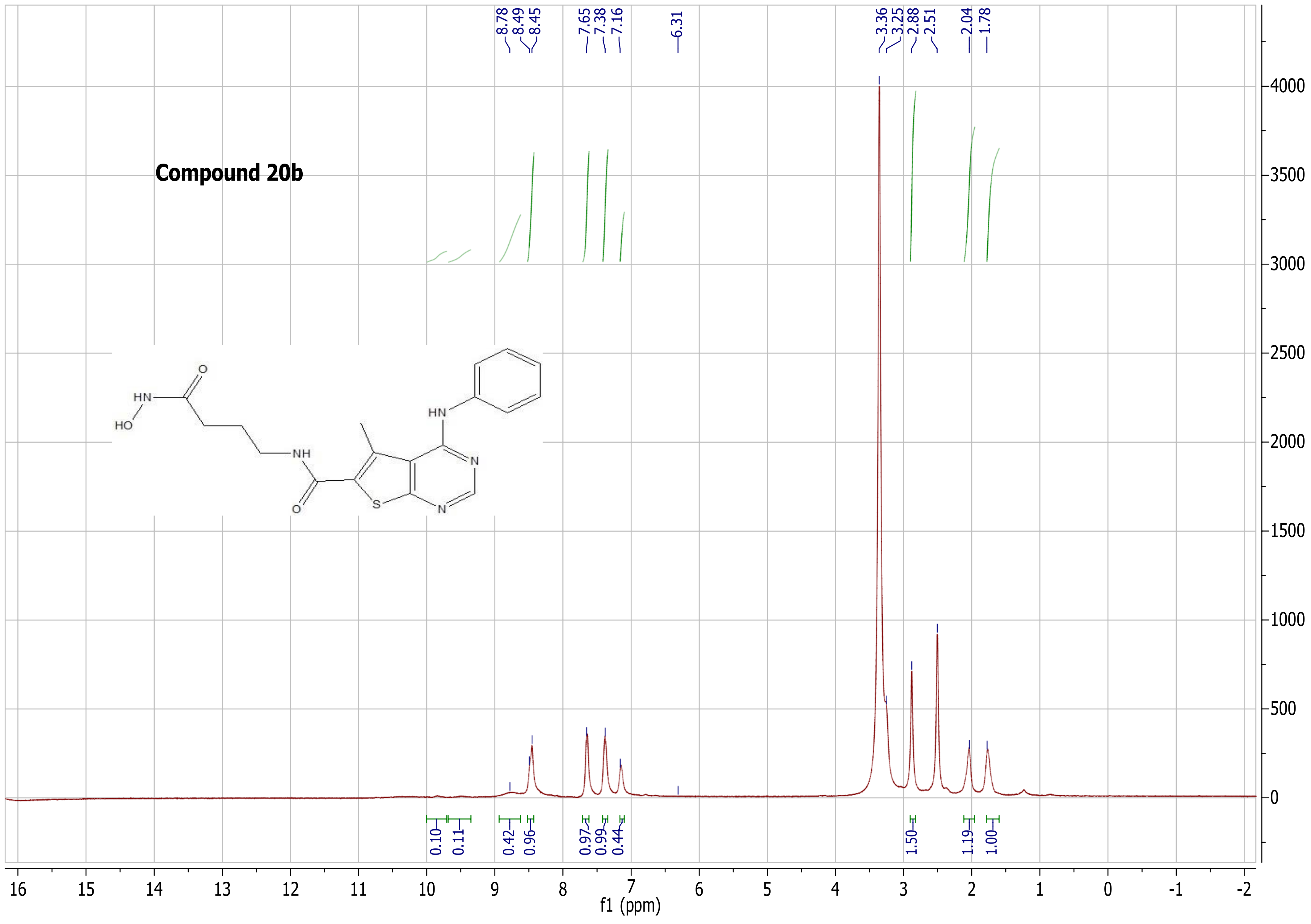
Compound 20b
HN
HO
O
NH
O
S
N
N
HN
8.78
8.49
8.45
7.65
7.38
7.16
6.31
3.36
3.25
2.88
2.51
2.04
1.78
0.10
0.11
0.42
0.96
0.97
0.99
0.44
1.50
1.19
1.00
f1 (ppm)

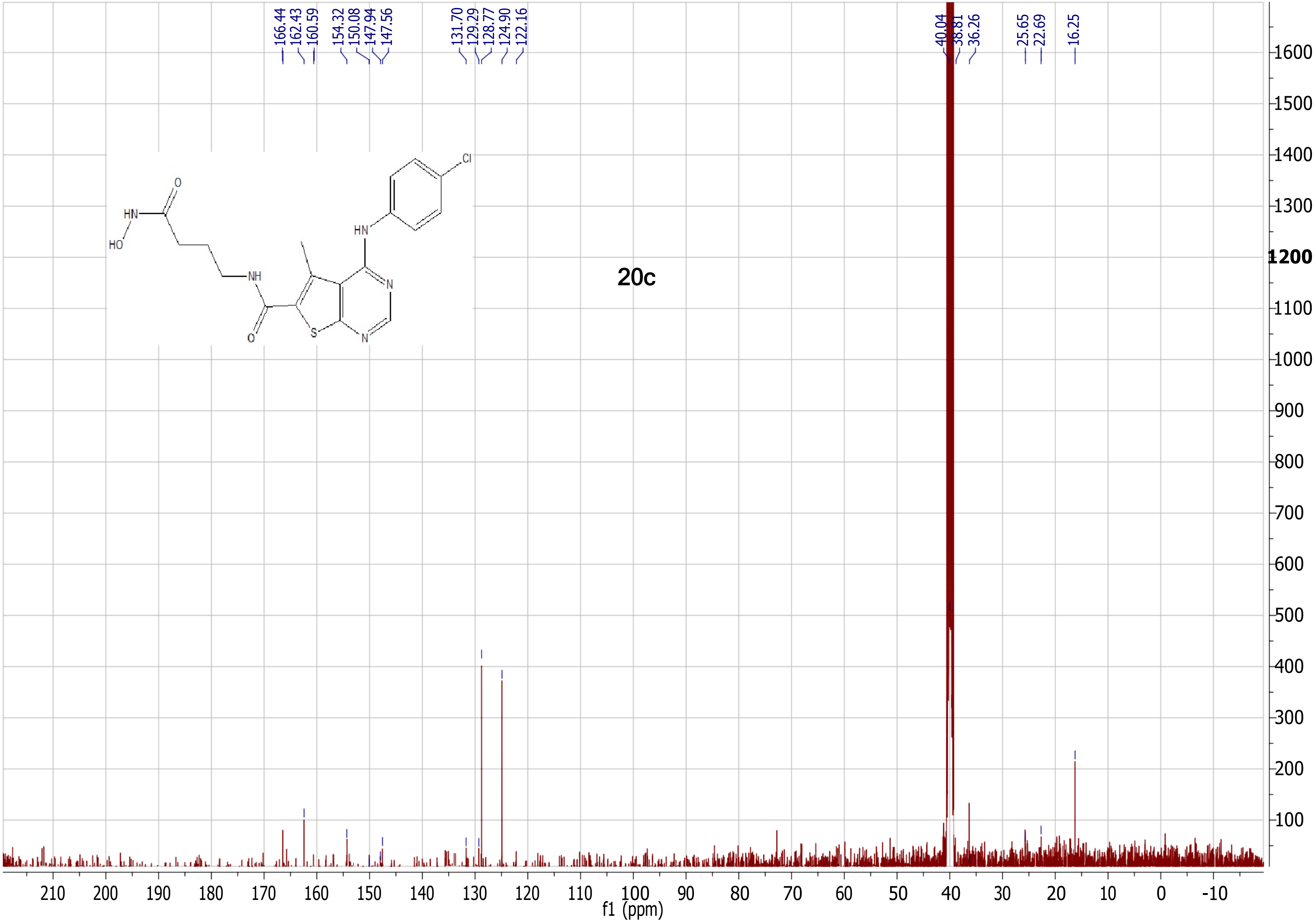
166.44
162.43
160.59
154.32
150.08
147.94
147.56
131.70
129.29
128.77
124.90
122.16
40.04
38.81
36.26
25.65
22.69
16.25
O
HN
HO
NH
O
S
N
N
HN
Cl
1600
1500
1400
1300
1200
1100
1000
900
800
700
600
500
400
300
200
100
210 200 190 180 170 160 150 140 130 120 110 100 90 80 70 60 50 40 30 20 10 0 -10
f1 (ppm)

20c

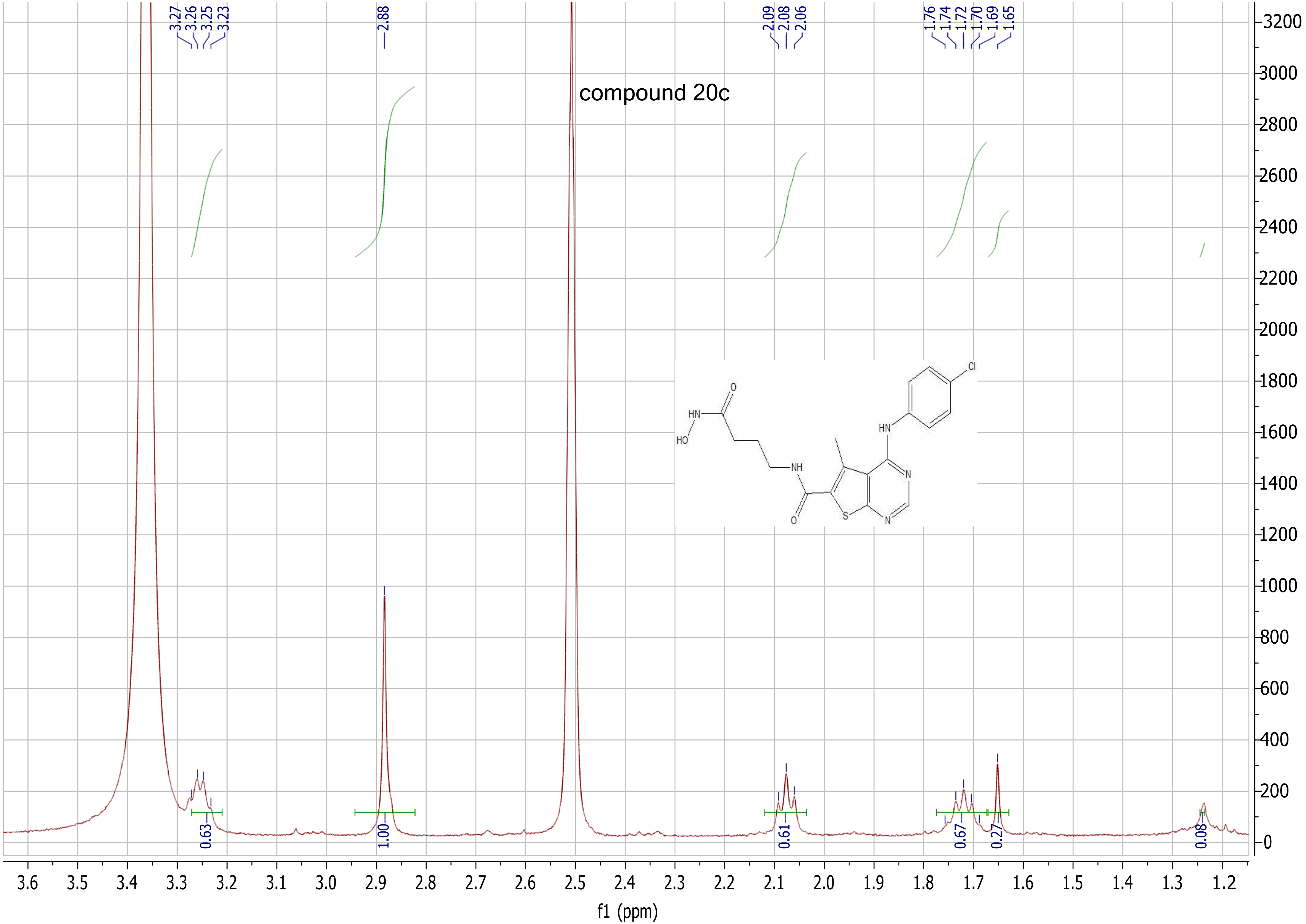

3.27
3.26
3.25
3.23
2.88
2.09
2.08
2.06
1.76
1.74
1.72
1.70
1.69
1.65
compound 20c
0.63
1.00
0.61
0.67
0.27
0.08
f1 (ppm)

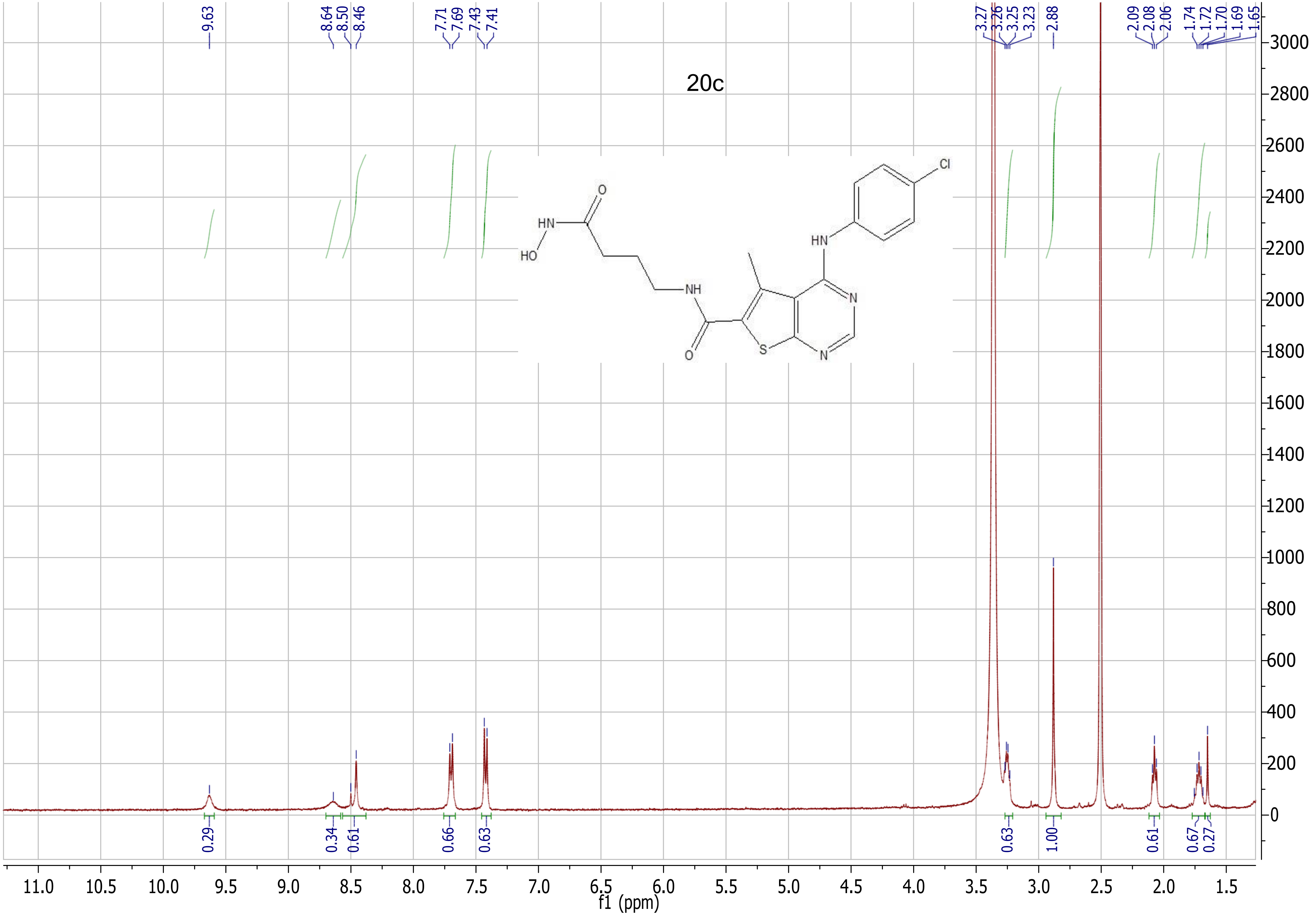

20c
9.63
8.64
8.50
8.46
7.71
7.69
7.43
7.41
3.27
3.26
3.25
3.23
2.88
2.09
2.08
2.06
1.74
1.72
1.70
1.69
1.65
0.29
0.34
0.61
0.66
0.63
0.63
1.00
0.61
0.67
0.27
HN
HO
O
NH
O
S
N
N
HN
Cl
f1 (ppm)
11.0 10.5 10.0 9.5 9.0 8.5 8.0 7.5 7.0 6.5 6.0 5.5 5.0 4.5 4.0 3.5 3.0 2.5 2.0 1.5
3000 2800 2600 2400 2200 2000 1800 1600 1400 1200 1000 800 600 400 200 0

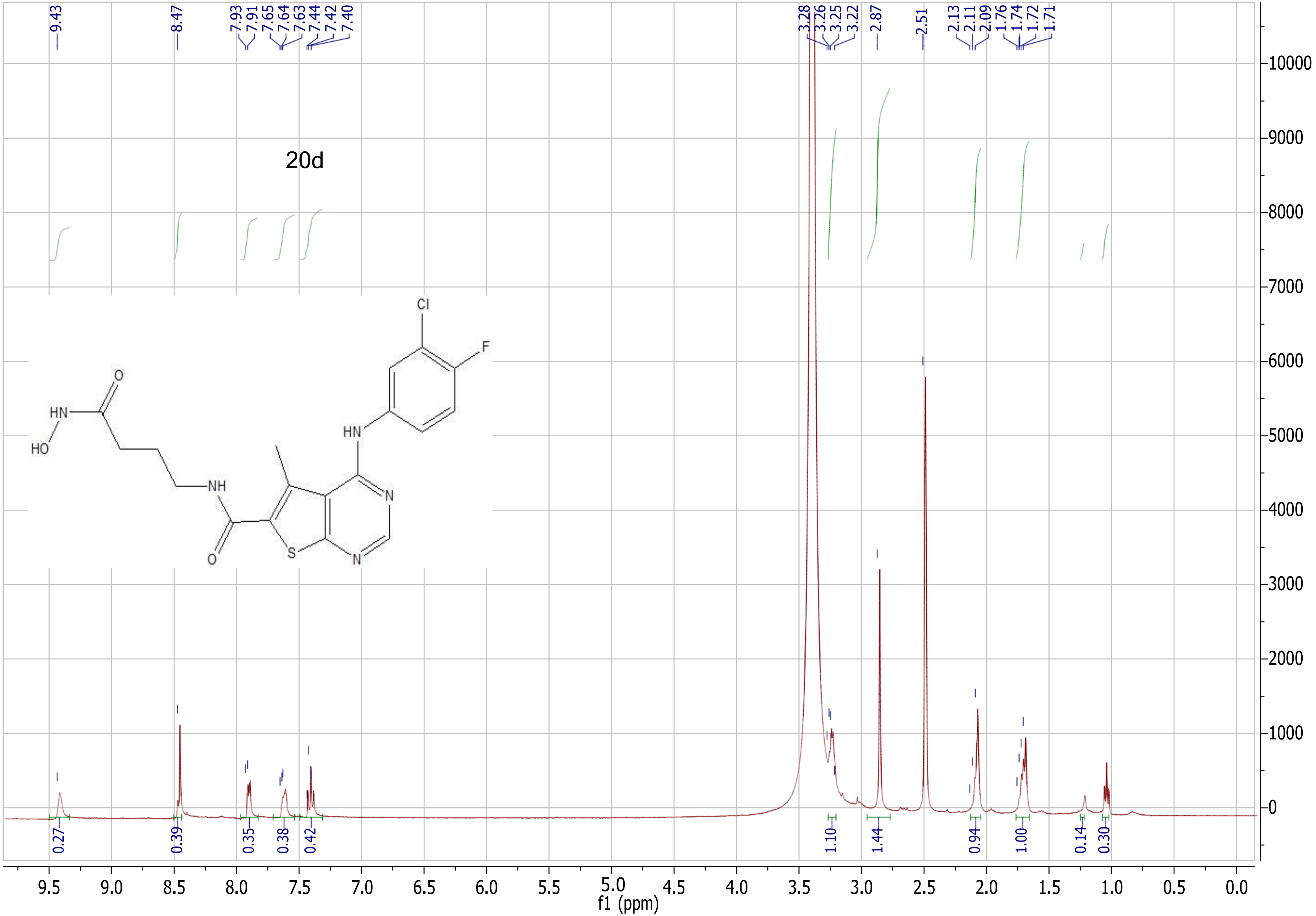

20d
9.43
8.47
7.93
7.91
7.65
7.64
7.63
7.44
7.42
7.40
3.28
3.26
3.25
3.22
2.87
2.51
2.13
2.11
2.09
1.76
1.74
1.72
1.71
0.27
0.39
0.35
0.38
0.42
1.10
1.44
0.94
1.00
0.14
0.30
f1 (ppm)
HN
HO
O
NH
HN
Cl
F
N
S

C:\Xcalibur\...\S\mona-abd-elatty-x5111d

THE OGY AND BIOTECHNOLOGY
AZH/

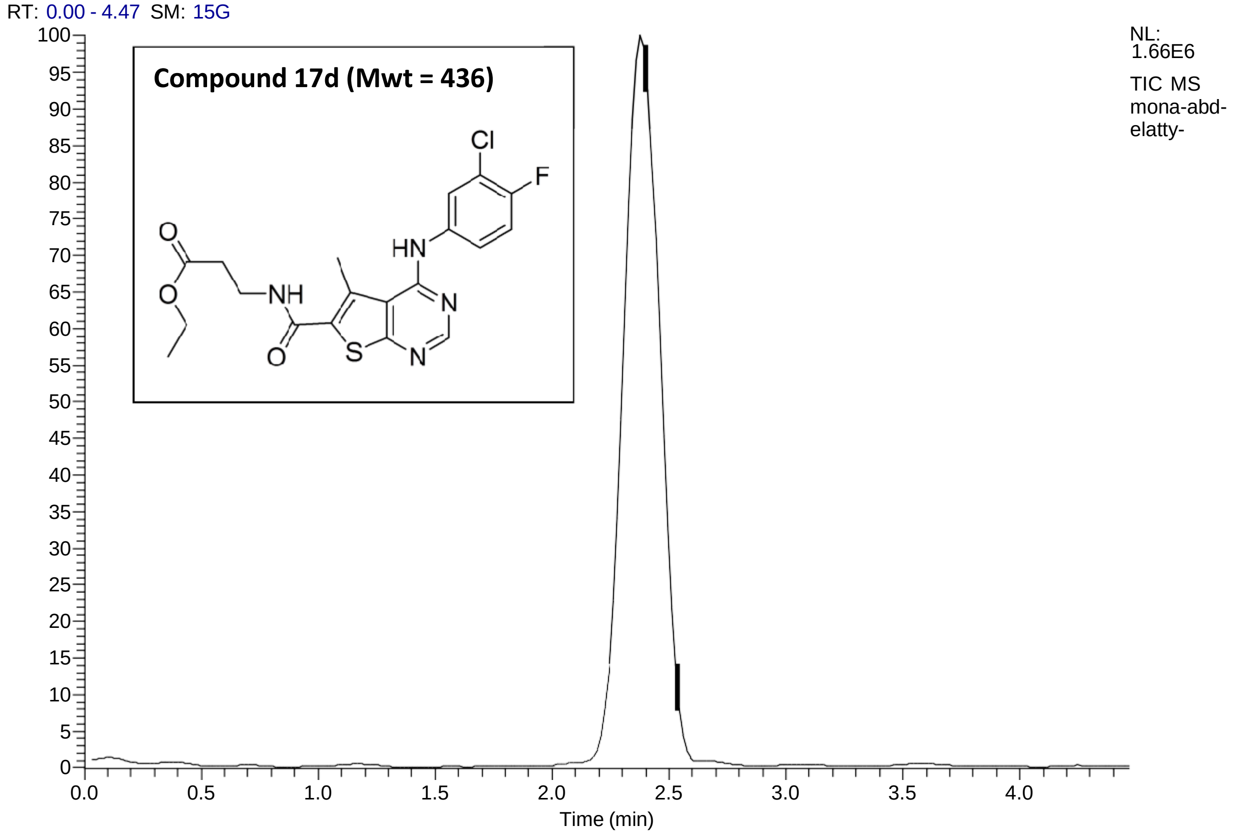

mona-abd-elatty-x5111d #130 RT: 2.19 AV: 1 SB: 2 1.89 , 1.87 NL: 5.07E2
T: {0,0} + c EI Full ms [40.00-1000.00]

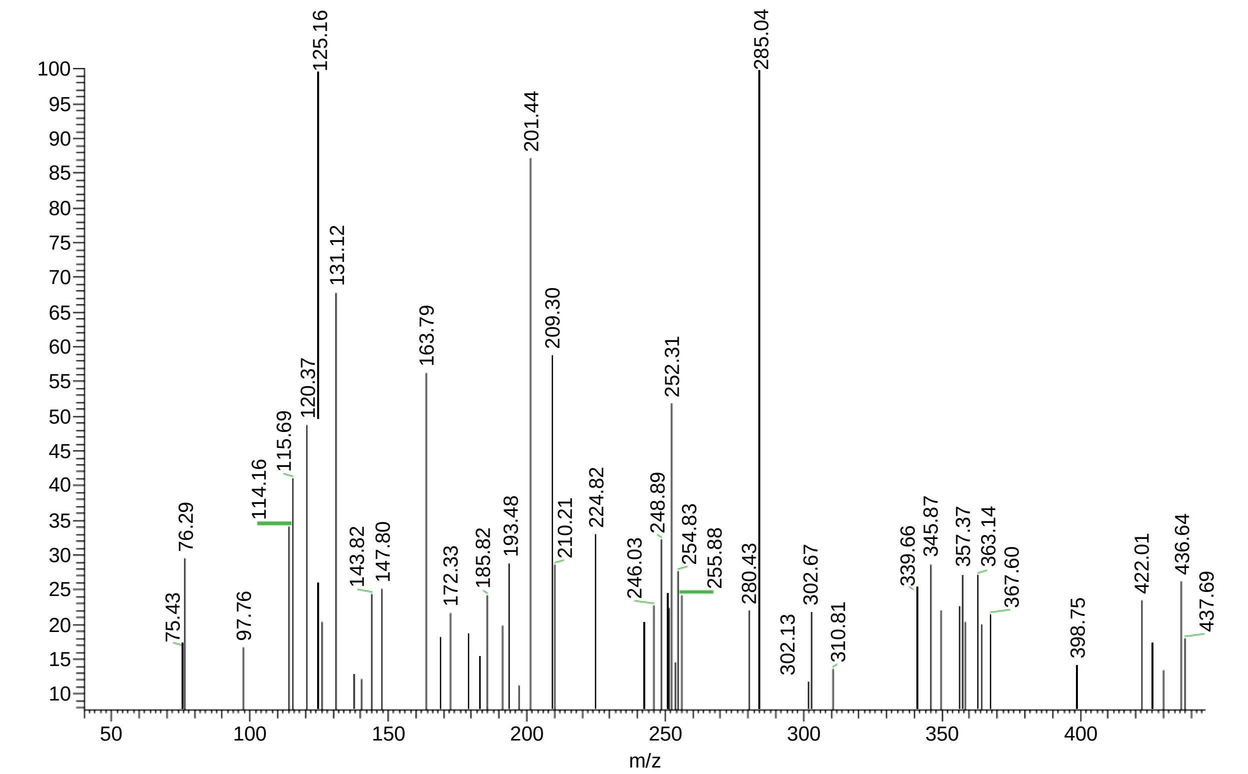

m/z	Intensity	Relative
75.43	84.4	16.66
76.29	149.2	29.46
97.76	84.3	16.64
112.77	37.8	7.46
114.16	172.8	34.11
115.69	207.3	40.93
120.37	246.7	48.70
123.29	127.7	25.22
125.16	504.9	99.68
126.10	102.5	20.24
131.12	342.6	67.65
137.52	65.2	12.87
140.50	61.2	12.08
143.82	123.7	24.43
147.80	127.3	25.14
163.79	284.8	56.23
168.74	92.0	18.16
172.33	109.5	21.61
178.77	94.3	18.61
183.07	78.1	15.41
185.82	122.8	24.24
191.14	99.7	19.69
193.48	145.3	28.69

197.05	56.7	11.19
201.44	441.4	87.15
206.80	39.5	7.79
209.30	297.7	58.78
210.21	144.4	28.51
224.82	166.7	32.90
242.72	99.6	19.66
246.03	115.2	22.74
248.89	162.9	32.17
249.41	120.3	23.74
251.54	113.1	22.32
252.31	262.5	51.83
253.93	73.1	14.42
254.83	140.4	27.72
255.88	122.7	24.22
280.43	111.3	21.98
285.04	506.5	100.00
302.13	59.1	11.66
302.67	110.0	21.72
310.81	68.4	13.50
339.66	125.6	24.80
345.87	144.9	28.61
349.54	110.8	21.87
356.73	114.0	22.51
357.37	137.2	27.09

358.43	103.1	20.35
363.14	136.9	27.03
364.33	100.8	19.90
367.60	108.7	21.45
398.75	71.6	14.14
422.01	118.4	23.37
425.26	84.5	16.69
427.07	93.3	18.43
430.16	67.3	13.29
436.64	132.9	26.24
437.69	90.5	17.87

mona-mohamed-abdelati-xiiic

THE AZH/ OGY AND BIOTECHNOLOGY

RT: 0.00 - 4.50 SM: 15G

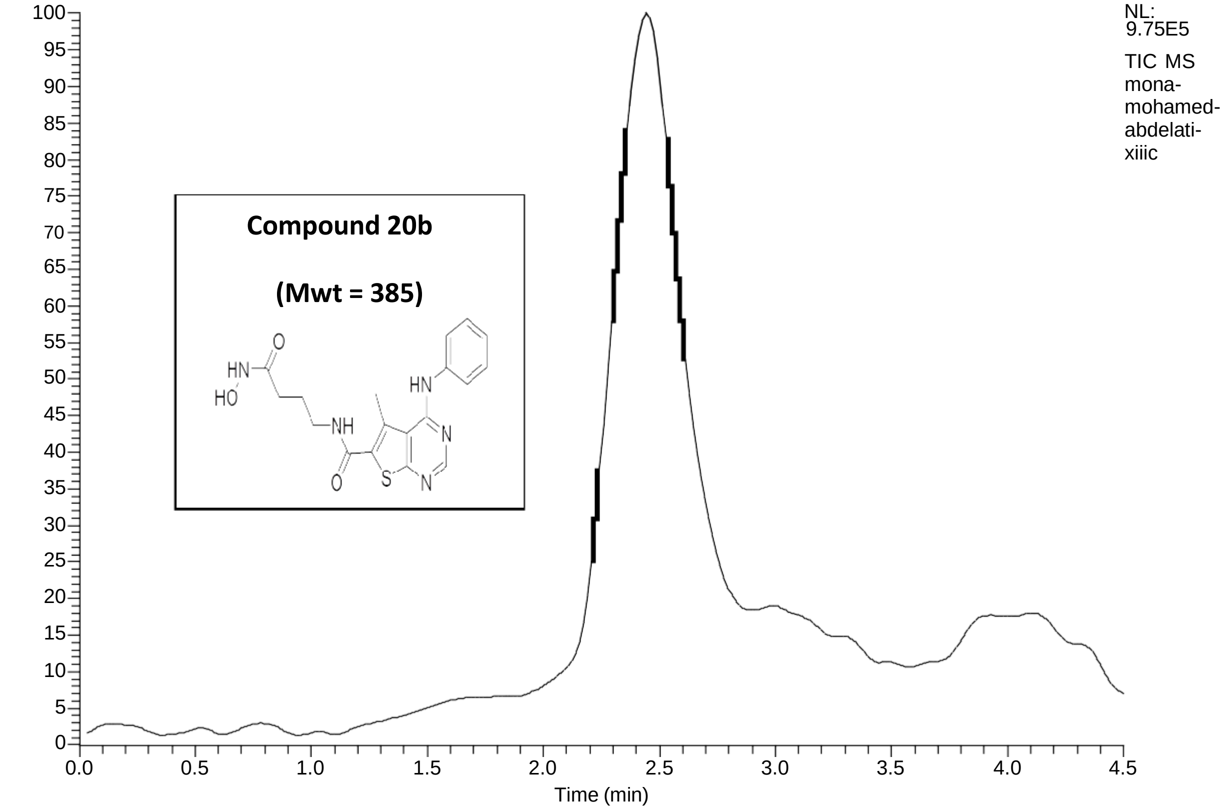

mona-mohamed-abdelati-xiiic #247 RT: 4.15 AV: 1 SB: 6 3.03 , 3.00-3.06 NL: 2.40E3
T: {0,0} + c EI Full ms [40.00-1000.00]

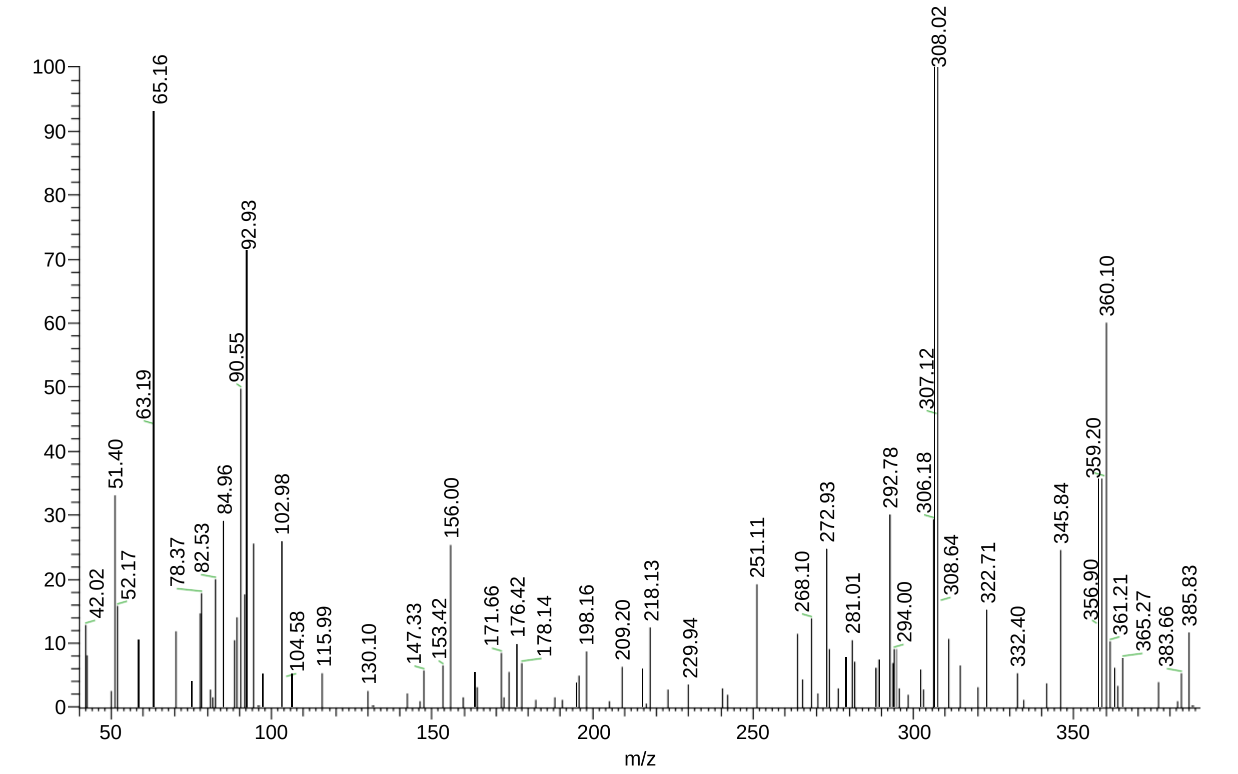

m/z	Intensity		Relative
42.02	307.6	12.83	
42.56	193.2	8.06	
50.17	57.2	2.39	
51.40	793.1	33.08	
52.17	375.8	15.68	
59.39	234.6	9.78	
63.19	1051.5		43.86
64.12	542.2	22.61	
65.16	2231.5		93.07
70.24	283.2	11.81	
74.95	95.3	3.98	
77.87	350.6	14.62	
78.37	426.6	17.79	
81.09	62.1	2.59	
81.96	33.6	1.40	
82.53	477.8	19.93	
84.96	697.6	29.10	
88.58	247.6	10.33	
89.18	336.0	14.01	
90.55	1191.7		49.70
91.73	423.0	17.64	
92.93	1715.6		71.55
94.52	613.4	25.58	

97.02	127.6	5.32
102.98	622.0	25.94
104.58	107.4	4.48
115.99	127.0	5.30
130.10	59.6	2.49
142.23	50.4	2.10
147.33	135.7	5.66
153.42	156.7	6.54
156.00	606.3	25.29
159.63	34.6	1.44
163.14	128.5	5.36
164.21	72.1	3.01
171.66	201.6	8.41
172.37	34.4	1.44
174.01	130.7	5.45
176.42	236.5	9.87
178.14	163.5	6.82
188.44	37.9	1.58
195.01	91.4	3.81
195.93	116.2	4.85
198.16	205.0	8.55
209.20	148.9	6.21
215.53	142.8	5.96
218.13	299.3	12.48
223.41	62.0	2.59

229.94	84.5	3.52
240.53	69.7	2.91
242.17	46.2	1.93
251.11	460.1	19.19
263.90	273.3	11.40
265.46	104.3	4.35
268.10	329.8	13.76
270.07	50.0	2.08
272.93	594.0	24.78
273.92	216.0	9.01
276.54	70.0	2.92
279.14	188.0	7.84
281.01	248.4	10.36
281.66	169.7	7.08
288.29	144.8	6.04
289.42	179.5	7.49
292.78	718.6	29.97
293.41	164.1	6.84
294.00	218.2	9.10
294.67	214.3	8.94
295.70	70.5	2.94
298.37	44.8	1.87
302.32	141.2	5.89
303.10	63.9	2.67
306.18	702.5	29.30

307.12	1091.0		45.50
308.02	2397.6		100.00
308.64	390.8	16.30	
309.37	177.7	7.41	
311.10	256.7	10.71	
314.74	155.9	6.50	
320.18	74.2	3.10	
322.71	366.0	15.26	
332.40	125.5	5.23	
341.45	89.2	3.72	
345.84	588.4	24.54	
356.90	307.2	12.81	
358.24	173.7	7.24	
359.20	857.5	35.77	
360.10	1438.6		60.00
361.21	247.1	10.31	
362.93	143.8	6.00	
363.58	77.0	3.21	
365.27	182.7	7.62	
376.54	93.7	3.91	
383.66	126.9	5.29	

385.83	276.5	11.53

C:\Xcalibur\...\S\mona-abd-elatii-X2111e

THE AZH OGY AND BIOTECHNOLOGY

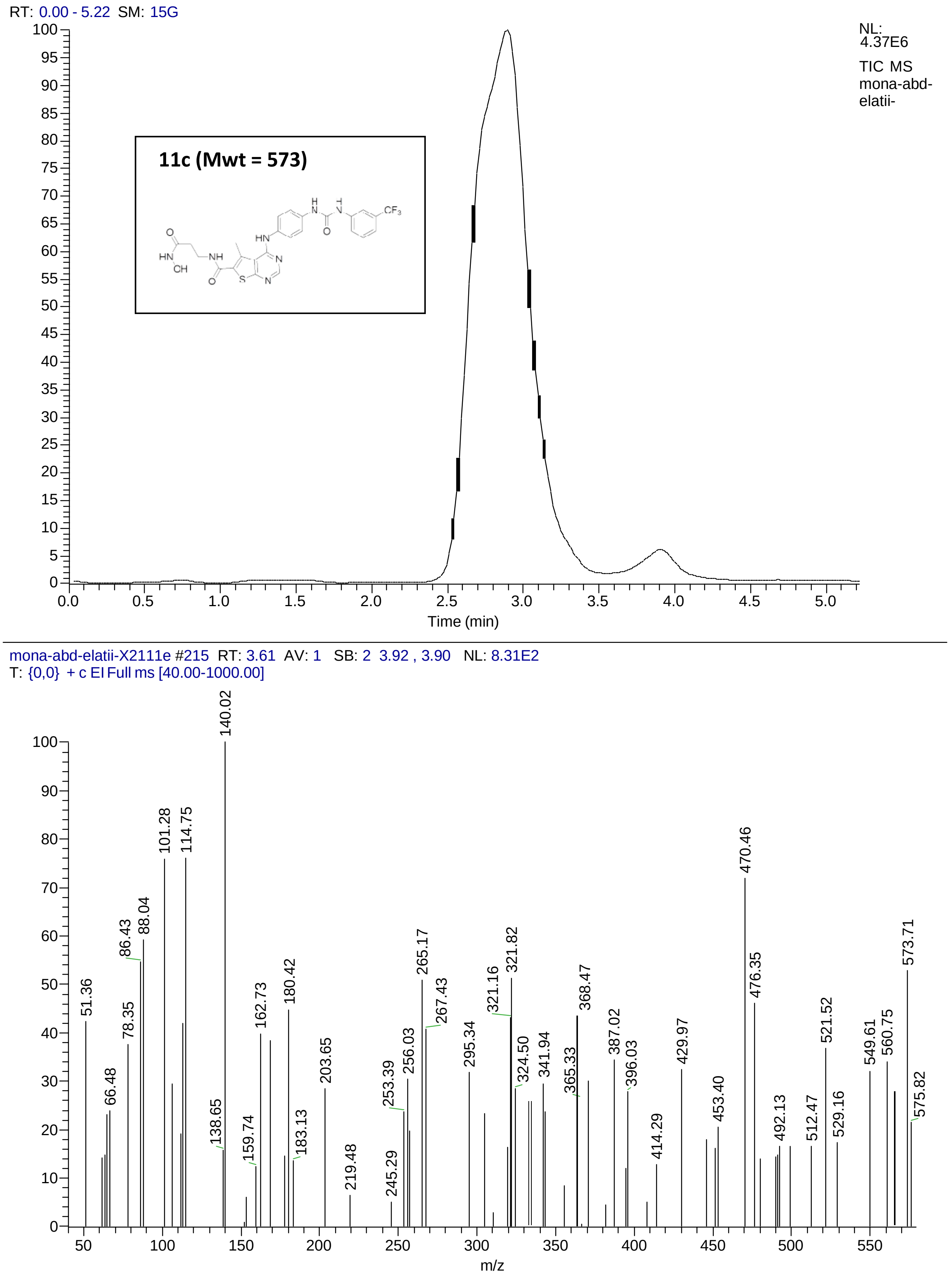

m/z	Intensity	Relative
51.36	351.8	42.35
61.50	117.6	14.15
63.40	122.5	14.75
64.49	191.9	23.09
66.48	199.3	23.99
78.35	311.6	37.50
86.43	454.1	54.65
88.04	492.7	59.30
101.28	631.2	75.97
106.14	245.5	29.55
111.50	158.5	19.07
113.25	348.0	41.89
114.75	632.8	76.16
138.65	130.9	15.76
140.02	830.9	100.00
153.37	50.4	6.07
159.74	103.1	12.40
162.73	330.6	39.79
168.31	319.3	38.43
177.54	121.4	14.61
180.42	371.3	44.69
183.13	112.3	13.51
203.65	236.9	28.51

219.48	53.3	6.42
245.29	42.2	5.08
253.39	196.8	23.68
256.03	253.8	30.54
257.18	163.3	19.65
265.17	422.8	50.88
267.43	338.9	40.79
295.34	264.1	31.79
305.00	194.5	23.41
310.17	23.3	2.80
319.39	136.1	16.38
321.16	358.5	43.15
321.82	426.0	51.27
324.50	236.4	28.45
332.68	206.1	24.81
337.34	129.3	15.56
341.94	244.9	29.48
343.45	196.7	23.67
355.32	70.8	8.52
365.33	220.4	26.52
368.47	362.6	43.65
370.94	249.3	30.01
382.09	36.7	4.42
387.02	286.5	34.48
394.73	100.0	12.03

396.03	231.1	27.81
408.12	42.0	5.05
414.29	106.9	12.87
429.97	270.0	32.49
446.26	149.7	18.02
451.60	134.1	16.14
453.40	170.5	20.52
470.46	598.1	71.98
476.35	383.4	46.15
480.31	115.9	13.94
490.25	118.8	14.30
491.42	123.3	14.84
492.13	137.9	16.59
499.27	136.9	16.48
512.47	138.1	16.62

521.52	305.6	36.78

mona-mohamed-abdelati-xu111e

THE AZH[illegible] [illegible]OGY AND BIOTECHNOLOGY

RT: 0.00 - 4.52 SM: 15G

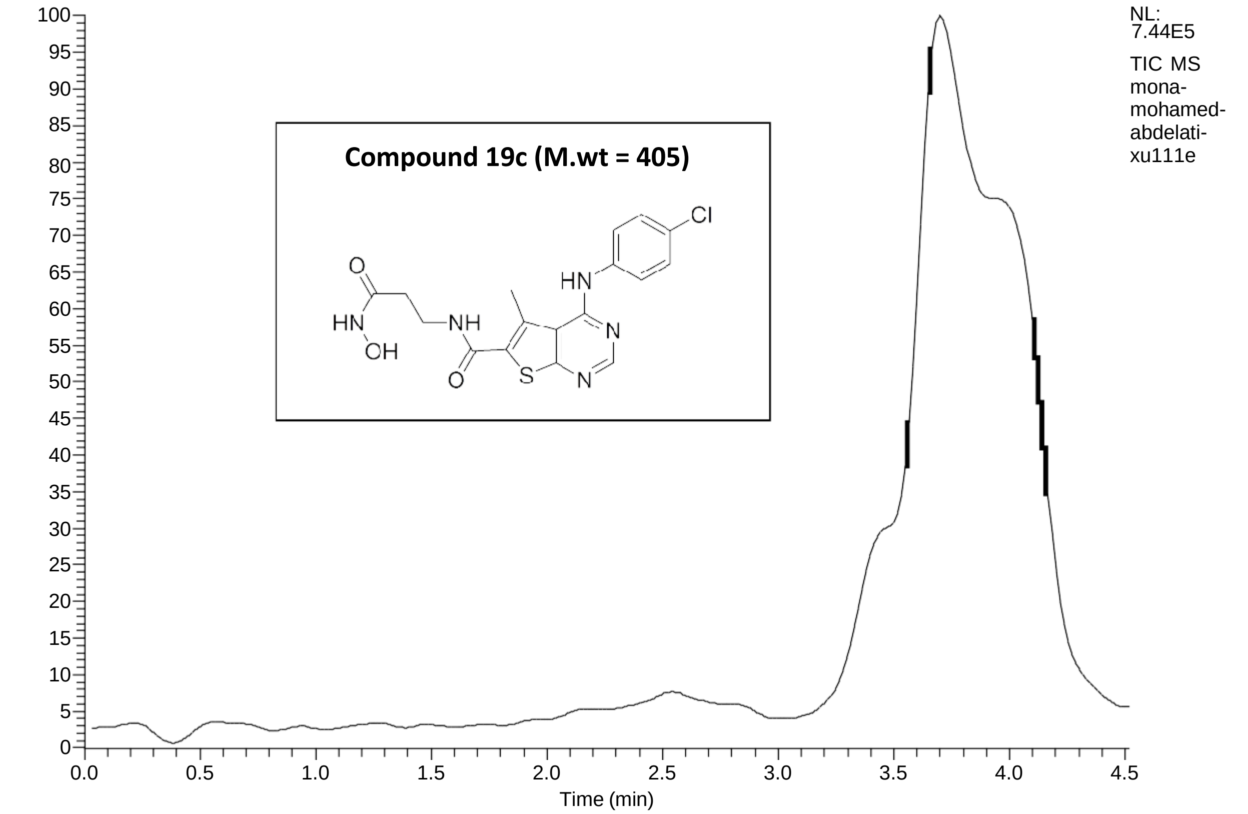

mona-mohamed-abdelati-xu111e #110 RT: 1.86 AV: 1 SB: 2 3.20 , 3.23 NL: 5.61E2
T: {0,0} + c EI Full ms [40.00-1000.00]

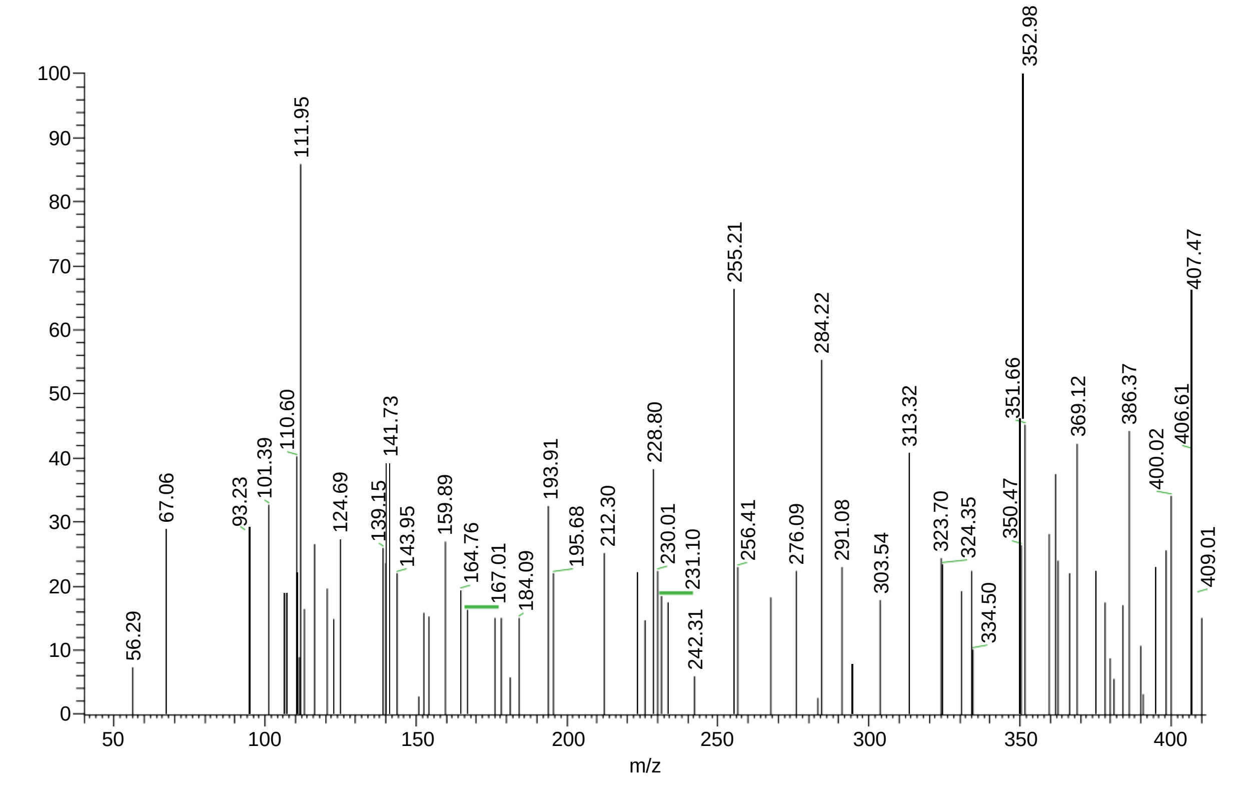

m/z	Intensity	Relative
56.29	40.3	7.18
67.06	162.0	28.88
93.23	160.1	28.55
101.39	183.3	32.68
106.09	106.0	18.90
107.23	106.7	19.02
110.60	225.5	40.19
111.24	49.1	8.75
111.95	481.2	85.79
113.22	91.8	16.37
114.53	119.2	21.25
116.30	148.4	26.46
120.65	109.9	19.59
122.70	82.4	14.69
124.69	153.3	27.34
139.15	145.5	25.93
140.09	131.3	23.41
141.73	220.1	39.24
143.41	105.2	18.75
143.95	122.5	21.84
150.81	15.3	2.72
152.61	88.1	15.71
154.21	84.9	15.14

159.89	150.5	26.84
164.76	108.5	19.35
167.01	90.5	16.14
176.10	84.1	15.00
178.41	83.9	14.95
181.38	31.7	5.66
184.09	84.5	15.07
193.91	181.6	32.37
195.68	123.3	21.99
212.30	140.9	25.12
223.28	123.9	22.08
226.03	82.1	14.64
228.80	214.7	38.27
230.01	124.7	22.22
231.10	103.5	18.45
233.37	97.7	17.42
242.31	32.6	5.81
255.21	372.5	66.41
256.41	129.1	23.01
267.34	101.7	18.14
276.09	125.2	22.32
283.16	13.7	2.45
284.22	310.2	55.31
291.08	129.1	23.01
292.68	39.6	7.06

303.54	100.1	17.85
313.32	229.3	40.87
323.70	136.0	24.25
324.35	130.9	23.34
330.71	107.2	19.11
333.94	124.9	22.27
334.50	55.7	9.94
350.47	147.5	26.29
351.66	253.0	45.12
352.98	560.9	100.00
359.56	157.3	28.05
361.95	210.3	37.49
362.83	133.9	23.88
366.22	122.9	21.92
369.12	236.7	42.19
375.15	124.7	22.22
378.26	97.2	17.33
379.73	48.3	8.60
381.26	30.8	5.49
384.19	95.5	17.02
386.37	247.3	44.09
389.99	60.0	10.70
390.92	17.4	3.10
395.21	128.7	22.94
398.28	142.9	25.48

400.02	191.3	34.11
405.00	104.8	18.68
406.05	46.5	8.28
406.61	231.5	41.26
407.47	371.7	66.27
409.01	104.8	18.68

409.99	83.9	14.95

mona-mohamed-abdelati-x5iiie

THE
AZH/

OGY AND BIOTECHNOLOGY

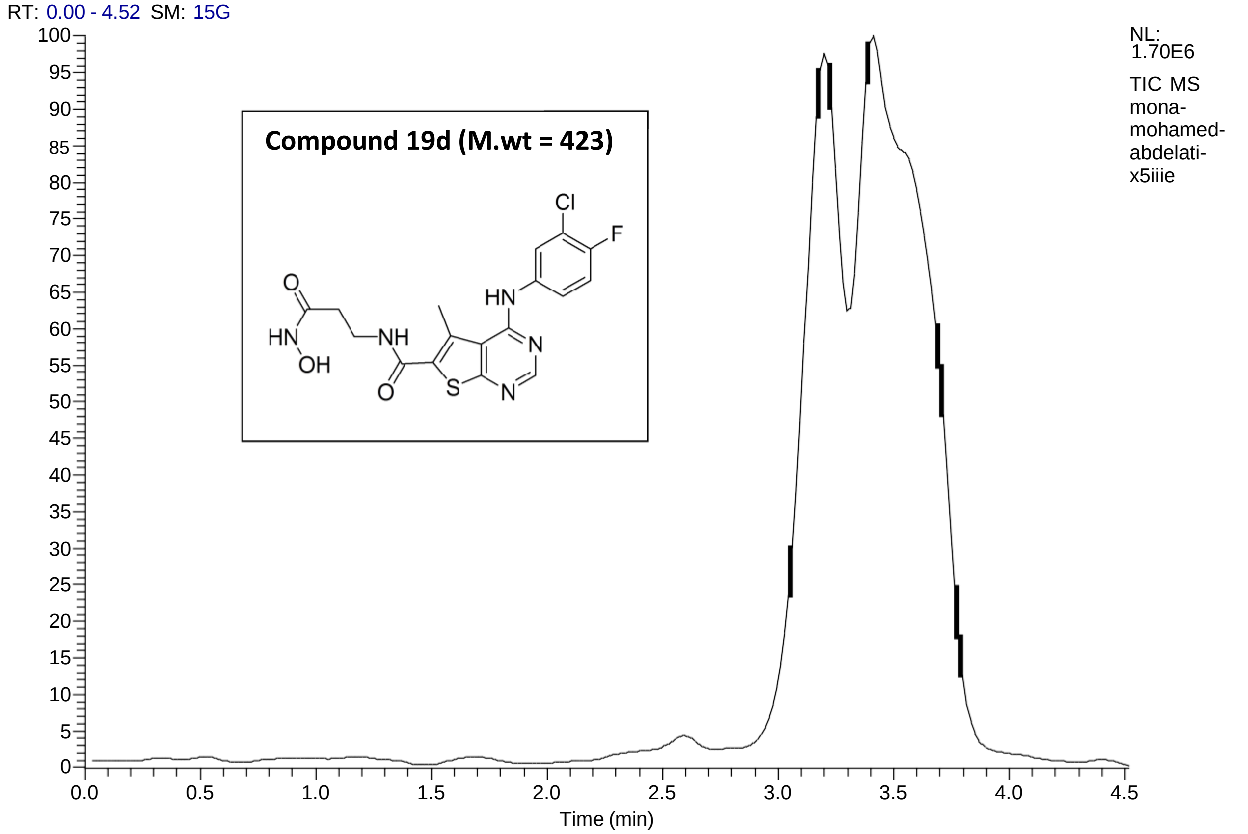

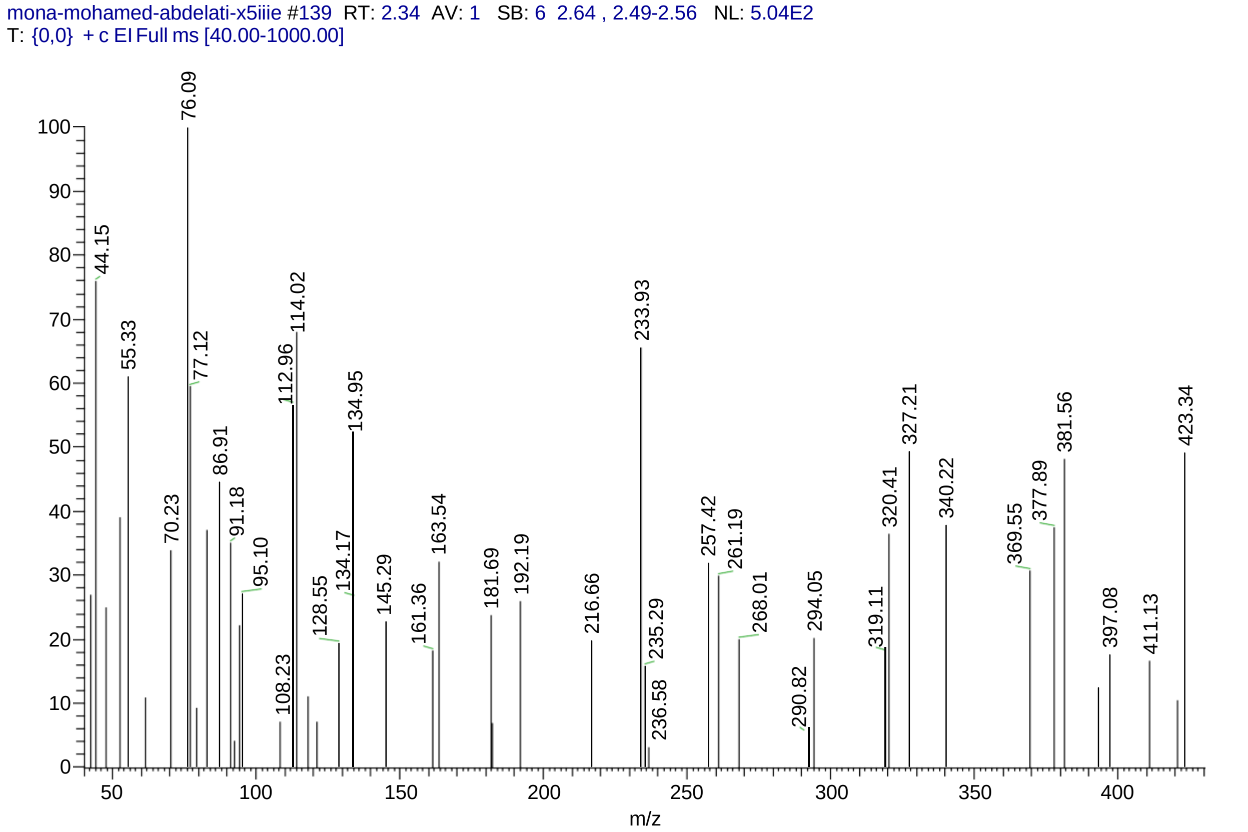

m/z	Intensity	Relative
42.23	135.7	26.92
44.15	382.5	75.85
47.80	125.3	24.85
52.62	196.5	38.97
55.33	307.8	61.04
61.56	54.8	10.88
70.23	170.7	33.85
76.09	504.3	100.00
77.12	300.0	59.50
79.03	46.7	9.27
82.55	186.8	37.04
86.91	224.3	44.47
91.18	176.6	35.01
92.26	20.2	4.00
94.09	111.5	22.11
95.10	137.0	27.17
108.23	35.8	7.09
112.96	285.2	56.55
114.02	342.5	67.92
118.23	55.4	10.98
121.34	35.8	7.10
128.55	97.7	19.38
134.17	133.6	26.49

134.95	265.1	52.57
145.29	114.1	22.63
161.36	91.9	18.22
163.54	161.6	32.05
181.69	119.7	23.75
182.23	34.2	6.78
192.19	130.5	25.87
216.66	99.2	19.67
233.93	330.8	65.60
235.29	79.4	15.75
236.58	15.3	3.03
257.42	160.1	31.75
261.19	150.9	29.93
268.01	100.1	19.86
290.82	27.7	5.49
294.05	101.8	20.19
319.11	90.9	18.02
320.41	183.8	36.45
327.21	248.7	49.32
340.22	191.1	37.90
369.55	154.8	30.70
377.89	189.1	37.49
381.56	242.3	48.06
393.42	63.0	12.49
397.08	89.0	17.65

411.13	84.0	16.66
420.82	52.1	10.32

423.34	247.7	49.11

mona-mohamed-abdelati-xu111c

THE
AZH/

OGY AND BIOTECHNOLOGY

RT: 1.67 - 3.18 SM: 15G

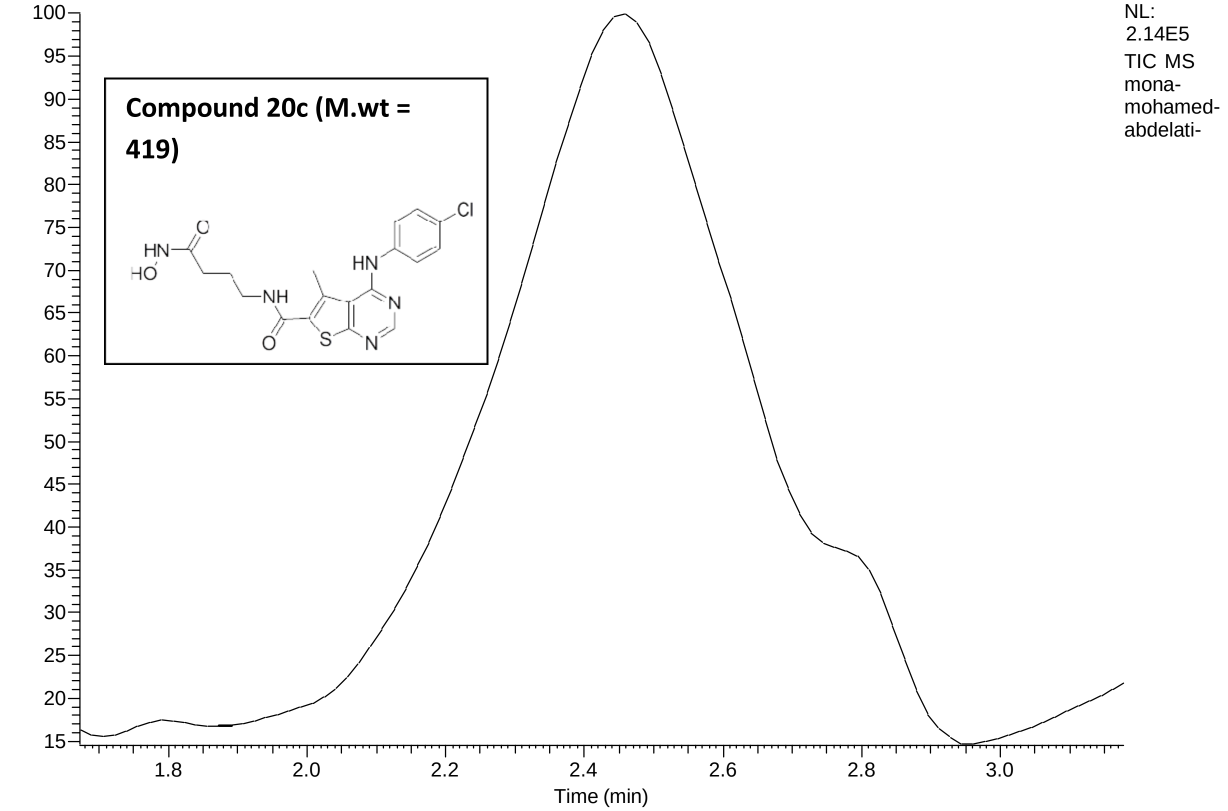

mona-mohamed-abdelati-xu111c #155 RT: 2.61 AV: 1 SB: 2 4.45 , 4.45 NL: 2.05E3
T: {0,0} + c EI Full ms [40.00-1000.00]

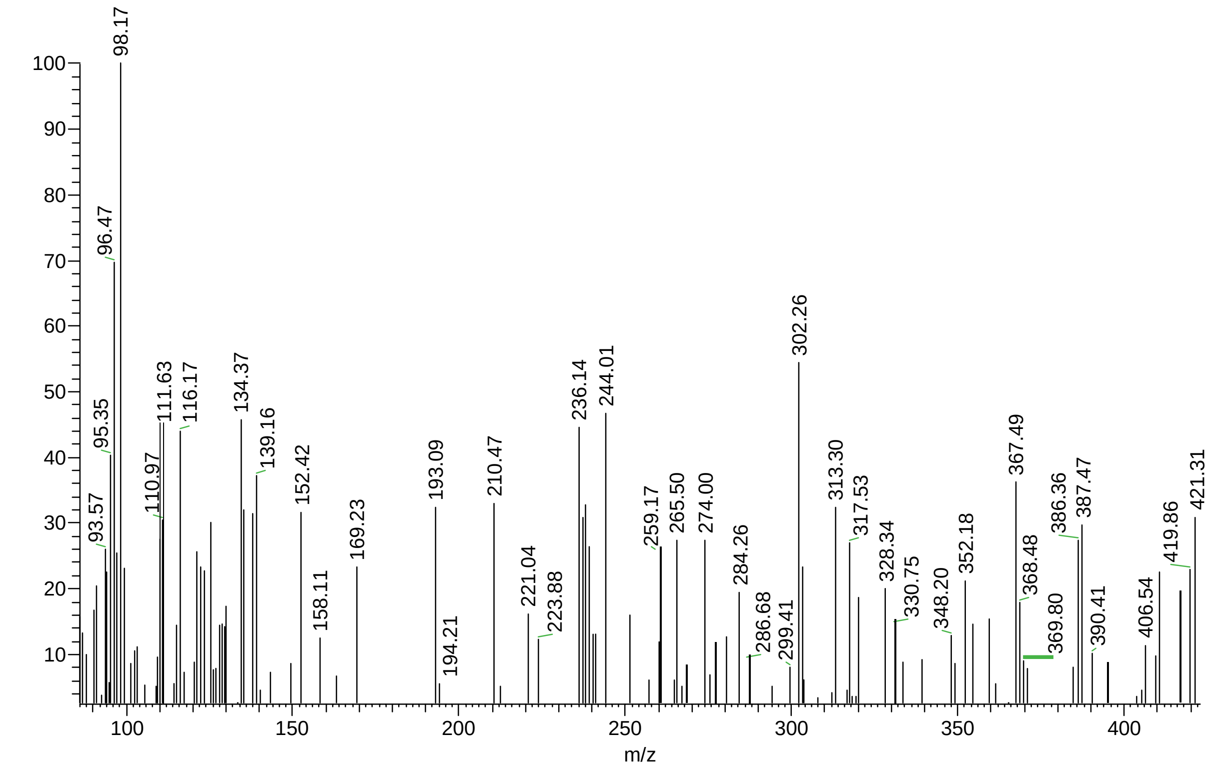

m/z	Intensity		Relative
86.19	322.5	15.73	
86.93	272.9	13.31	
88.09	206.0	10.05	
90.16	342.4	16.70	
91.17	417.8	20.38	
92.39	78.0	3.80	
93.57	534.4	26.06	
94.18	461.4	22.50	
94.74	116.7	5.69	
95.35	829.0	40.43	
96.47	1430.8		69.78
97.23	522.9	25.50	
98.17	2050.5		100.00
99.24	473.7	23.10	
101.22	175.2	8.54	
102.54	215.3	10.50	
103.29	229.1	11.17	
105.62	107.5	5.24	
108.84	107.3	5.23	
109.38	197.5	9.63	
110.22	566.8	27.64	
110.97	626.2	30.54	
111.63	931.7	45.44	

112.30	559.6	27.29
113.11	395.7	19.30
114.16	113.9	5.55
115.05	295.8	14.43
116.17	902.5	44.01
117.35	148.1	7.22
120.41	178.8	8.72
121.19	526.0	25.65
122.31	478.6	23.34
123.36	468.0	22.82
125.27	618.2	30.15
126.09	158.5	7.73
126.89	162.1	7.91
128.15	294.1	14.34
128.93	301.4	14.70
129.49	290.8	14.18
130.15	354.0	17.26
133.48	51.9	2.53
134.37	936.6	45.68
135.32	656.4	32.01
138.13	645.6	31.48
139.16	765.1	37.31
140.22	91.7	4.47
143.14	147.6	7.20
149.30	178.4	8.70

152.42	647.3	31.57
158.11	255.6	12.46
163.29	138.5	6.76
169.23	480.0	23.41
193.09	666.0	32.48
194.21	111.7	5.45
210.47	676.4	32.98
212.44	104.3	5.08
221.04	330.6	16.12
223.88	252.9	12.33
236.14	913.0	44.53
237.24	631.3	30.79
238.14	674.4	32.89
239.03	540.6	26.37
240.14	269.8	13.16
241.21	268.6	13.10
244.01	956.7	46.66
251.41	329.3	16.06
257.27	126.0	6.14
259.17	524.0	25.55
260.14	244.3	11.91
264.61	125.6	6.12
265.50	560.0	27.31
267.09	105.2	5.13
269.24	156.4	7.63

274.00	562.1	27.41	
275.35	142.0	6.92	
276.14	226.7	11.05	
280.47	259.8	12.67	
284.26	397.2	19.37	
286.68	187.1	9.12	
294.14	103.6	5.05	
299.41	164.3	8.01	
302.26	1118.5		54.54
303.19	478.5	23.34	
303.84	126.3	6.16	
307.87	69.5	3.39	
312.14	87.1	4.25	
313.30	664.1	32.39	
316.87	93.7	4.57	
317.53	553.7	27.00	
318.28	73.1	3.56	
319.33	71.7	3.50	
320.10	383.4	18.70	
328.34	412.6	20.12	
330.75	300.4	14.65	
333.53	178.8	8.72	
339.38	190.7	9.30	
348.20	264.1	12.88	
349.34	176.8	8.62	

352.18	435.3	21.23
354.42	298.1	14.54
359.35	314.9	15.36
361.20	113.5	5.53
365.20	54.8	2.67
367.49	742.0	36.18
368.48	367.7	17.93
369.80	184.5	9.00
370.74	159.1	7.76
384.61	164.4	8.02
386.36	561.3	27.37
387.47	609.4	29.72
390.41	207.7	10.13
395.34	163.5	7.97
403.86	73.1	3.56
405.35	94.8	4.62
406.54	233.9	11.40
409.47	198.7	9.69
410.74	462.4	22.55
416.62	385.2	18.78
419.86	469.4	22.89
421.31	633.8	30.91
529.16	144.5	17.39
549.61	265.6	31.96
560.75	283.3	34.10

565.85	223.2	26.86
573.71	440.1	52.97
575.82	178.7	21.50

C:\Xcalibur\data\S\mona-abd-elatii-Xiie

THE [illegible] OGY AND BIOTECHNOLOGY
AZH[illegible]

RT: 3.66 - 4.00 SM: 15G

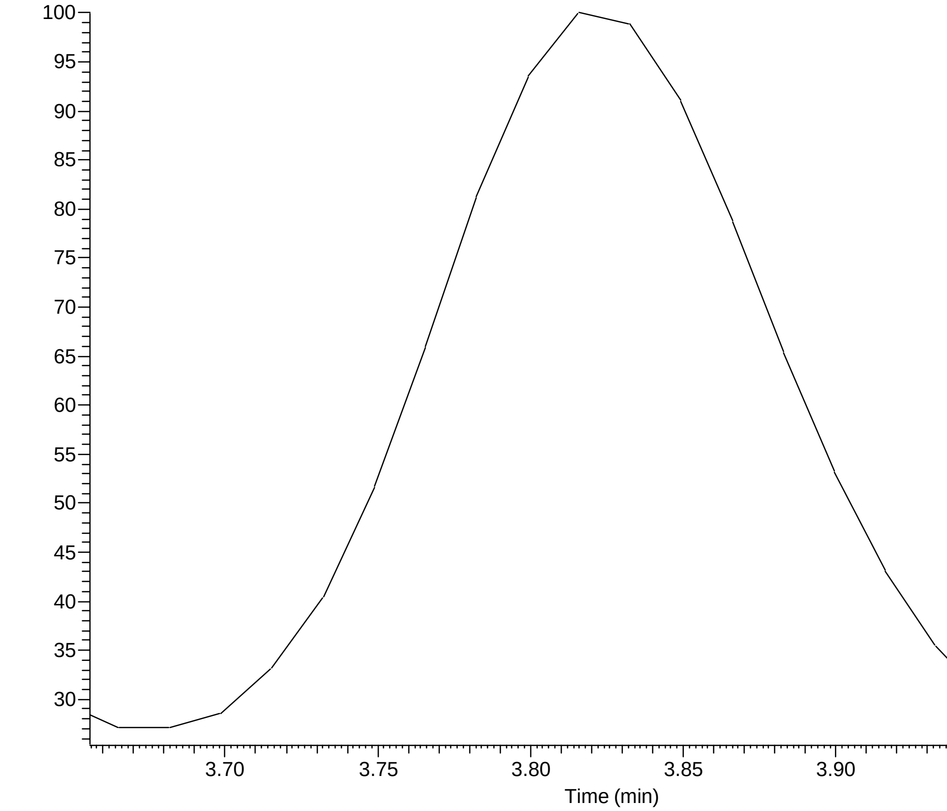

Compound 19b (M.wt = 371)

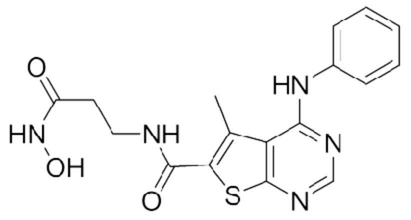

mona-abd-elatii-Xiie #191 RT: 3.21 AV: 1 SB: 2 4.45 , 4.45 NL: 4.35E2
T: {0,0} + c EI Full ms [40.00-1000.00]

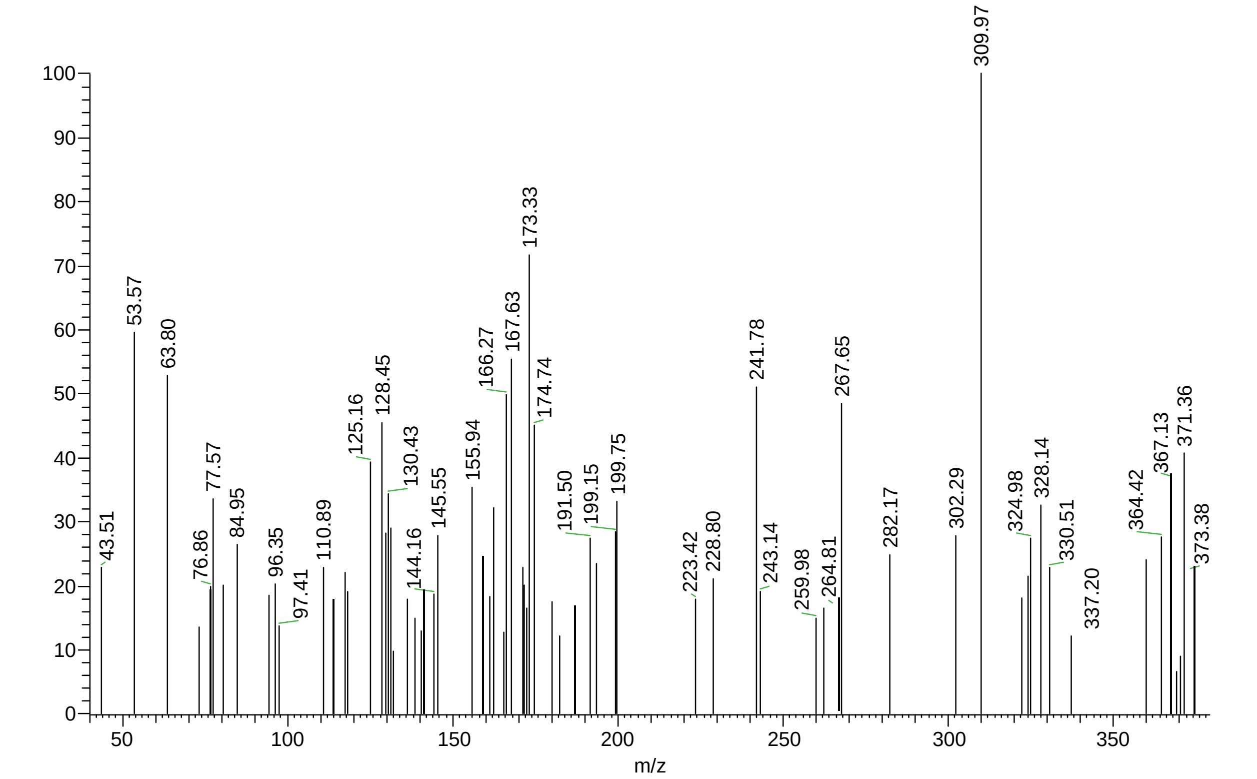

m/z	Intensity	Relative
43.51	99.7	22.92
53.57	259.8	59.71
63.80	230.3	52.91
73.41	58.9	13.54
76.86	87.2	20.04
77.57	146.3	33.61
79.23	81.7	18.78
80.62	87.6	20.13
84.95	115.2	26.47
94.37	80.7	18.54
96.35	88.1	20.25
97.41	59.6	13.69
110.89	99.6	22.89
113.22	74.4	17.10
117.35	96.0	22.06
118.15	83.2	19.12
125.16	171.2	39.34
128.45	198.0	45.50
129.62	123.3	28.34
130.43	149.6	34.37
131.09	126.7	29.11
132.14	43.1	9.90
136.42	78.1	17.95

138.75	64.9	14.92
140.37	56.9	13.08
140.96	81.2	18.66
144.16	81.9	18.81
145.55	121.5	27.91
155.94	153.7	35.32
157.29	103.7	23.84
161.28	79.5	18.26
162.23	140.4	32.26
165.55	55.6	12.78
166.27	217.2	49.91
167.63	241.5	55.48
171.12	100.0	22.98
171.65	88.0	20.22
172.21	72.1	16.57
173.33	311.8	71.66
174.74	196.1	45.07
180.06	76.0	17.46
182.24	52.9	12.16
186.57	70.3	16.15
191.50	119.3	27.42
193.69	102.0	23.44
199.15	124.3	28.55
199.75	144.8	33.27
223.42	78.0	17.92

228.80	91.9	21.11
241.78	222.3	51.07
243.14	83.2	19.12
259.98	65.6	15.07
262.14	72.3	16.61
264.81	74.0	17.00
267.65	210.9	48.47
282.17	108.3	24.88
302.29	121.1	27.82
309.97	435.2	100.00
322.27	79.1	18.17
324.13	93.6	21.51
324.98	119.5	27.45
328.14	142.0	32.63
330.51	99.5	22.86
337.20	53.1	12.19
359.86	104.9	24.11
364.42	120.1	27.60
367.13	160.1	36.80
369.30	28.9	6.65
370.09	39.2	9.01
371.36	177.5	40.78
373.38	96.9	22.27

mona-mohamed-abdelati-x2iv   18-Jun-19 3:32:15 PM
THE
AZH/   OGY AND BIOTECHNOLOGY

RT: 0.00 - 4.55 SM: 15G

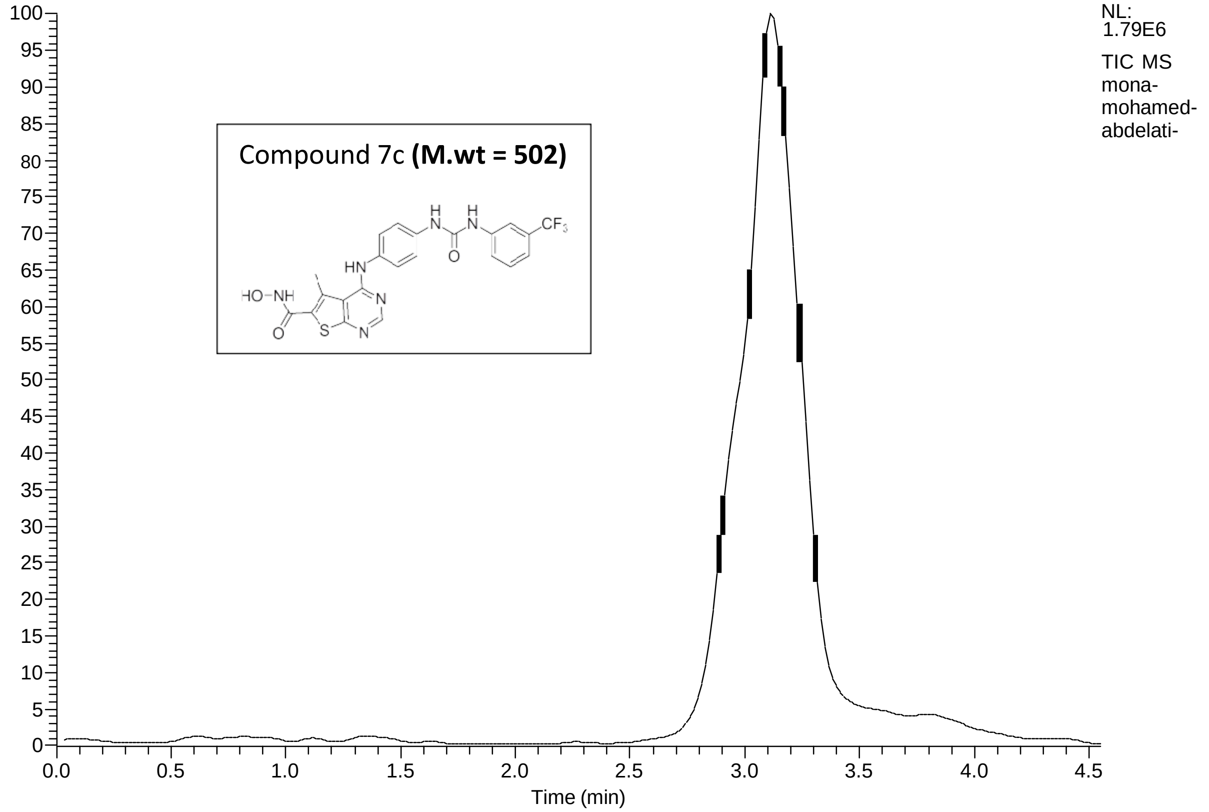

mona-mohamed-abdelati-x2iv #78 RT: 1.32 AV: 1 SB: 2 4.55 , 4.55 NL: 3.54E2
T: {0,0} + c EI Full ms [40.00-1000.00]

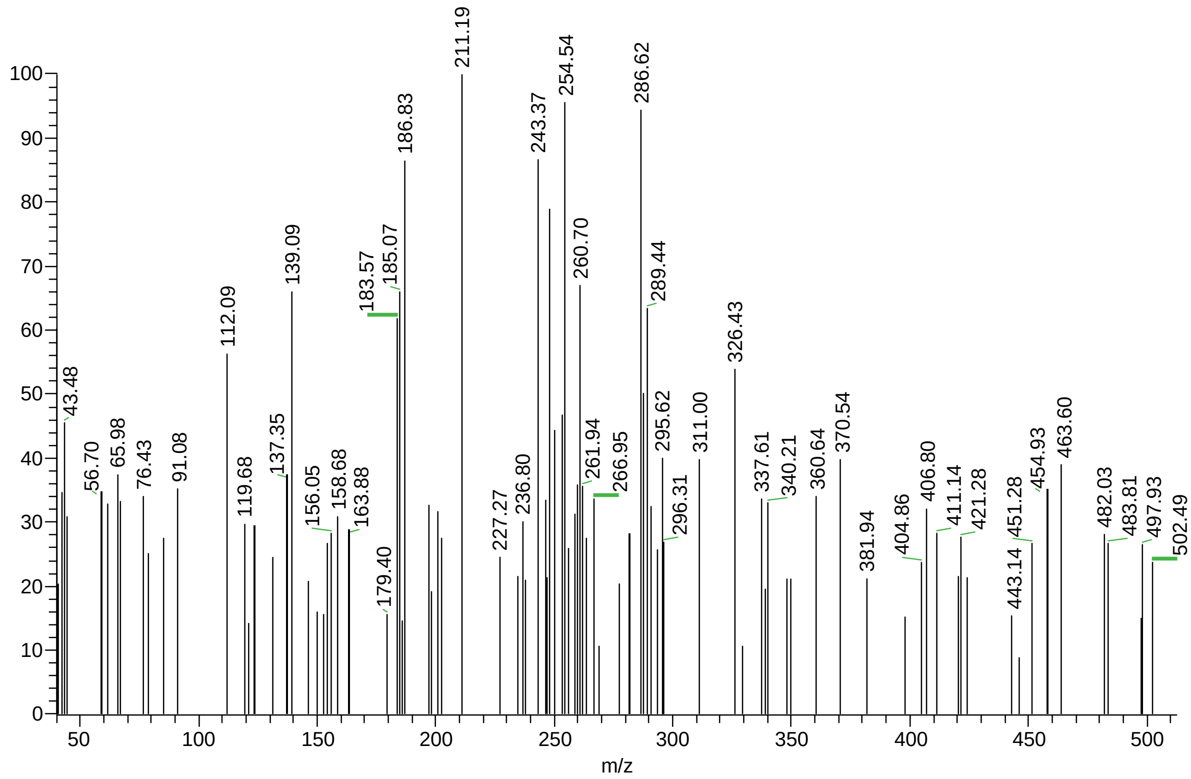

m/z	Intensity	Relative
40.10	212.9	60.22
40.74	71.6	20.25
42.36	122.4	34.62
43.48	161.3	45.63
44.80	108.9	30.81
56.70	120.5	34.09
61.57	115.9	32.77
65.98	132.1	37.37
67.10	117.6	33.26
76.43	120.3	34.01
78.81	88.5	25.04
85.43	97.3	27.53
91.08	124.4	35.18
112.09	199.1	56.30
119.68	104.8	29.64
121.04	50.4	14.25
125.08	101.6	28.73
131.45	86.8	24.55
137.35	129.6	36.65
139.09	233.2	65.95
146.10	73.1	20.66
150.26	56.3	15.91
152.94	54.9	15.54

154.33	94.1	26.62
156.05	99.9	28.24
158.68	109.1	30.84
163.88	99.3	28.09
179.40	55.1	15.57
183.57	218.3	61.73
185.07	232.9	65.87
185.95	51.7	14.63
186.83	305.8	86.50
197.37	115.1	32.54
198.10	68.0	19.23
200.64	112.0	31.67
202.56	97.1	27.45
211.19	353.6	100.00
227.27	86.4	24.43
234.62	76.3	21.57
236.80	106.1	30.02
238.07	74.1	20.97
243.37	306.2	86.61
246.15	118.0	33.37
246.69	75.2	21.27
248.17	278.5	78.77
249.99	156.8	44.34
253.28	165.2	46.72
254.54	337.7	95.51

256.26	91.3	25.83
258.81	110.5	31.26
259.74	126.8	35.86
260.70	236.8	66.97
261.94	125.6	35.52
263.49	97.3	27.53
266.95	119.1	33.67
269.07	37.3	10.56
277.67	71.6	20.25
279.51	97.1	27.45
286.62	333.4	94.31
287.47	177.1	50.08
289.44	224.0	63.35
290.63	114.8	32.47
293.47	90.5	25.60
295.62	141.5	40.01
296.31	94.9	26.85
311.00	140.9	39.86
326.43	190.1	53.77
329.39	37.2	10.52
337.61	118.7	33.56
339.16	69.2	19.57
340.21	117.1	33.11
347.95	74.5	21.08
350.01	74.4	21.04

360.64	120.7	34.13
370.54	140.9	39.86
381.94	74.8	21.15
398.14	53.5	15.12
404.86	84.0	23.76
406.80	113.6	32.13
411.14	99.9	28.24
420.21	76.3	21.57
421.28	97.9	27.68
424.20	75.7	21.42
443.14	54.5	15.42
446.33	30.9	8.75
451.28	94.3	26.66
454.93	121.9	34.46
463.60	138.0	39.03
482.03	99.5	28.13
483.81	94.5	26.73
497.28	52.9	14.97
497.93	93.6	26.47

RT: 2.44 - 2.73 SM: 15G

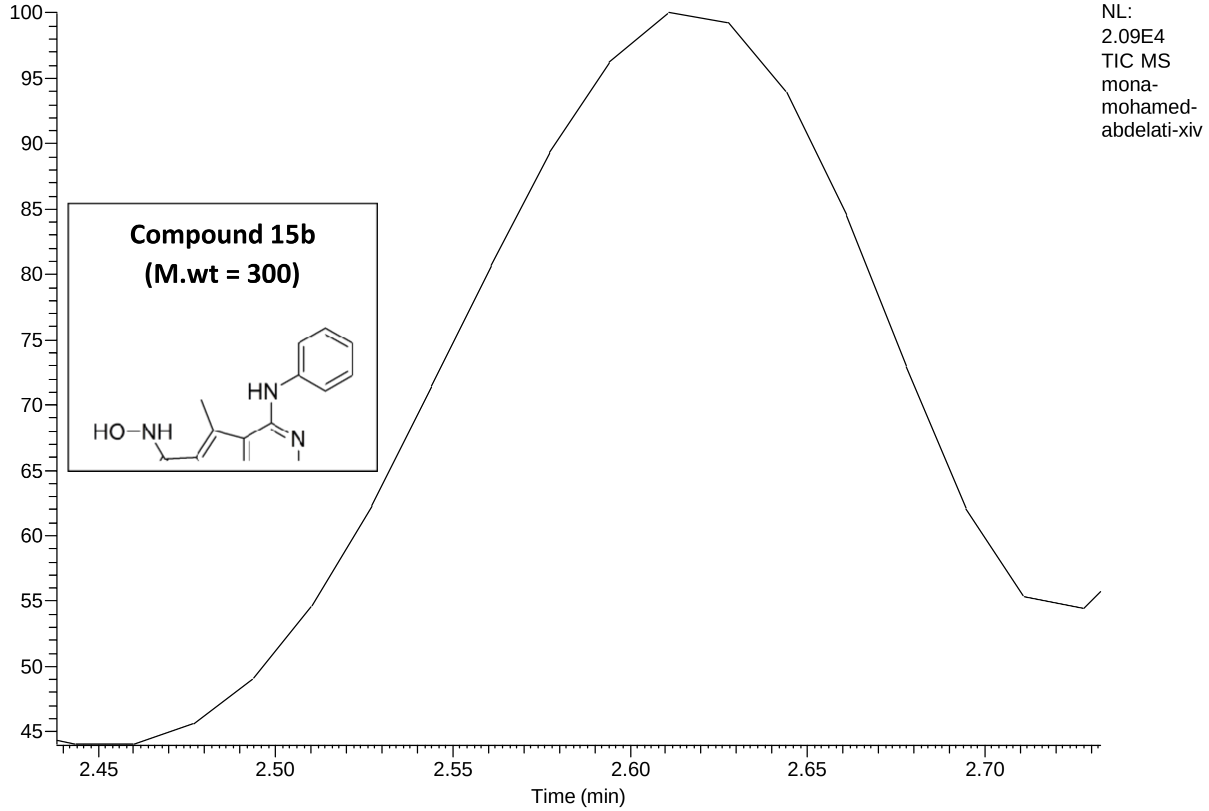

mona-mohamed-abdelati-xiv #166 RT: 2.79 AV: 1 SB: 2 4.45 , 4.45 NL: 5.24E2
T: {0,0} + c EI Full ms [40.00-1000.00]

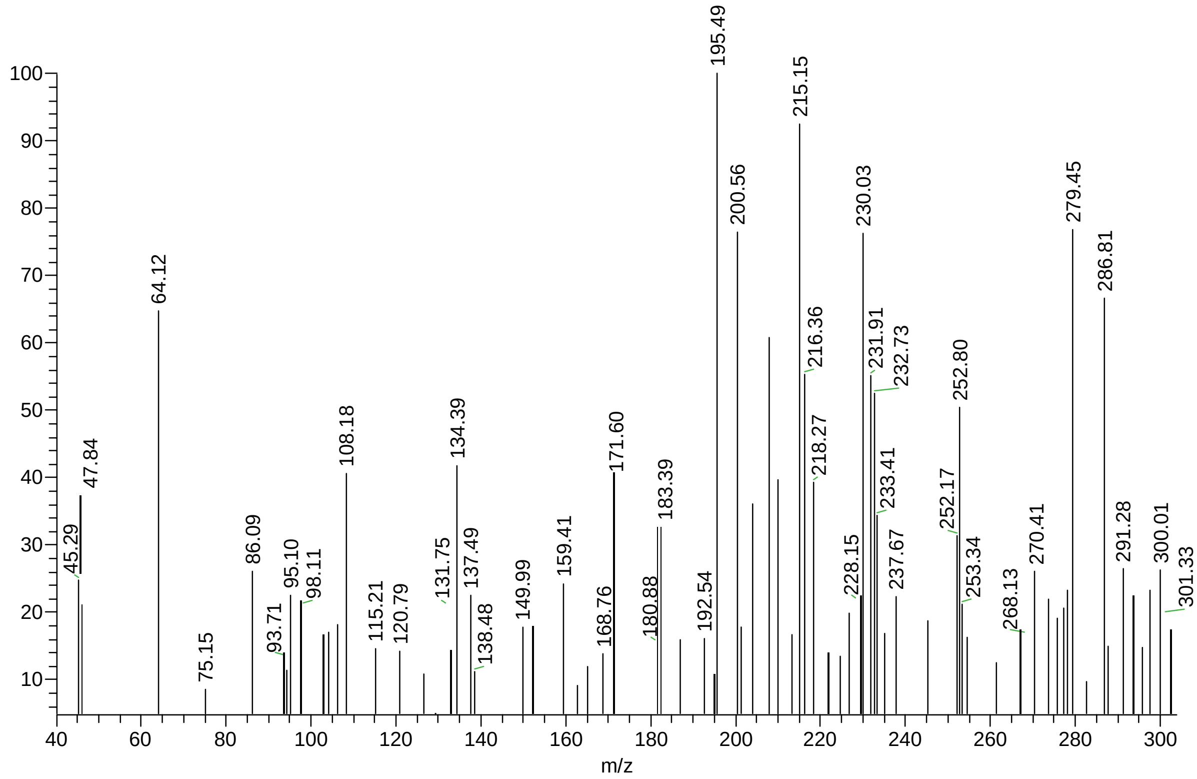

m/z	Intensity	Relative
45.29	129.9	24.80
45.84	91.1	17.39
46.45	107.5	20.52
47.84	196.5	37.54
64.12	339.0	64.76
75.15	44.8	8.56
86.09	137.1	26.18
93.71	69.3	13.24
94.22	60.1	11.48
95.10	118.4	22.61
98.11	110.3	21.06
101.37	48.8	9.32
102.02	83.5	15.94
104.22	89.5	17.09
106.09	95.2	18.18
108.18	212.4	40.57
115.21	76.9	14.69
120.79	74.5	14.23
126.46	56.5	10.80
129.37	25.6	4.89
131.75	110.3	21.06
132.60	71.2	13.60
134.39	218.7	41.76

137.49	118.1	22.56
138.48	58.3	11.13
149.99	93.7	17.90
151.28	90.7	17.32
159.41	126.4	24.14
162.82	47.6	9.09
165.01	62.4	11.92
168.76	72.7	13.88
171.60	213.9	40.85
180.88	81.5	15.56
181.68	58.8	11.23
183.39	171.2	32.70
187.04	83.7	15.99
192.54	84.0	16.04
195.49	523.6	100.00
198.83	52.8	10.08
200.56	400.2	76.45
201.35	93.7	17.90
203.94	188.8	36.06
207.93	318.1	60.76
209.89	207.7	39.67
213.27	87.2	16.65
215.15	484.9	92.62
216.36	289.7	55.33
218.27	206.1	39.37

221.88	69.2	13.22
224.76	70.1	13.39
226.80	103.9	19.84
228.15	114.1	21.80
230.03	399.4	76.29
231.91	289.2	55.23
232.73	275.3	52.58
233.41	180.0	34.38
235.01	88.7	16.93
237.67	117.2	22.38
245.27	98.3	18.77
252.17	164.8	31.47
252.80	264.6	50.55
253.34	111.2	21.24
254.54	85.6	16.35
261.41	65.6	12.53
267.34	69.6	13.29
268.13	87.2	16.65
270.41	136.9	26.15
273.55	115.2	22.00
275.70	100.7	19.23
277.34	107.9	20.60
278.27	122.4	23.38
279.45	402.1	76.80
282.66	50.5	9.65

286.81	349.6	66.77
287.74	78.5	15.00
291.28	138.4	26.43
294.41	113.9	21.75
295.94	77.9	14.87
297.47	122.4	23.38
300.01	137.9	26.33
301.33	103.1	19.68
302.54	87.5	16.70 m/z

mona-mohamed-abdelati-xuiv

18-Jun-19 1:39:01 PM
THE
AZH/

OGY AND BIOTECHNOLOGY

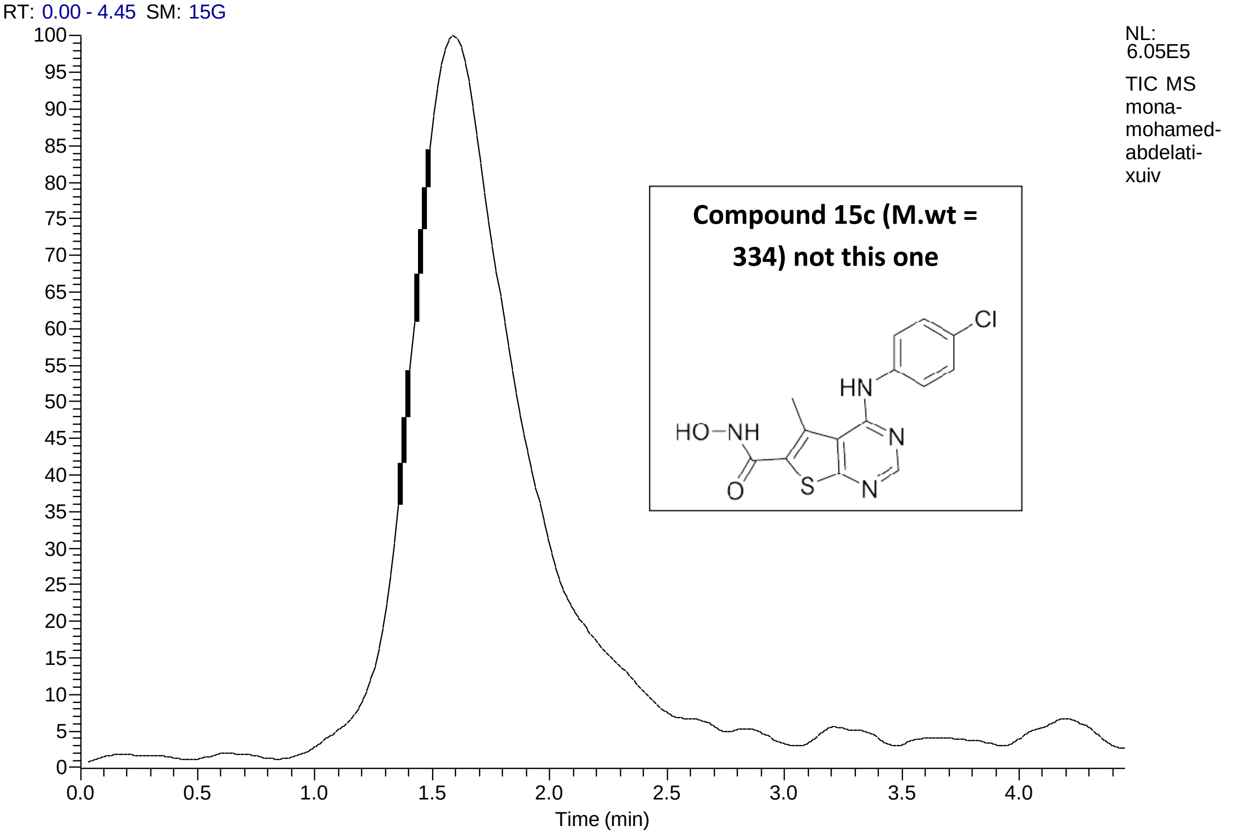

mona-mohamed-abdelati-xuiv #135 RT: 2.28 AV: 1 SB: 2 4.45 , 4.45 NL: 2.20E3
T: {0,0} + c EI Full ms [40.00-1000.00]

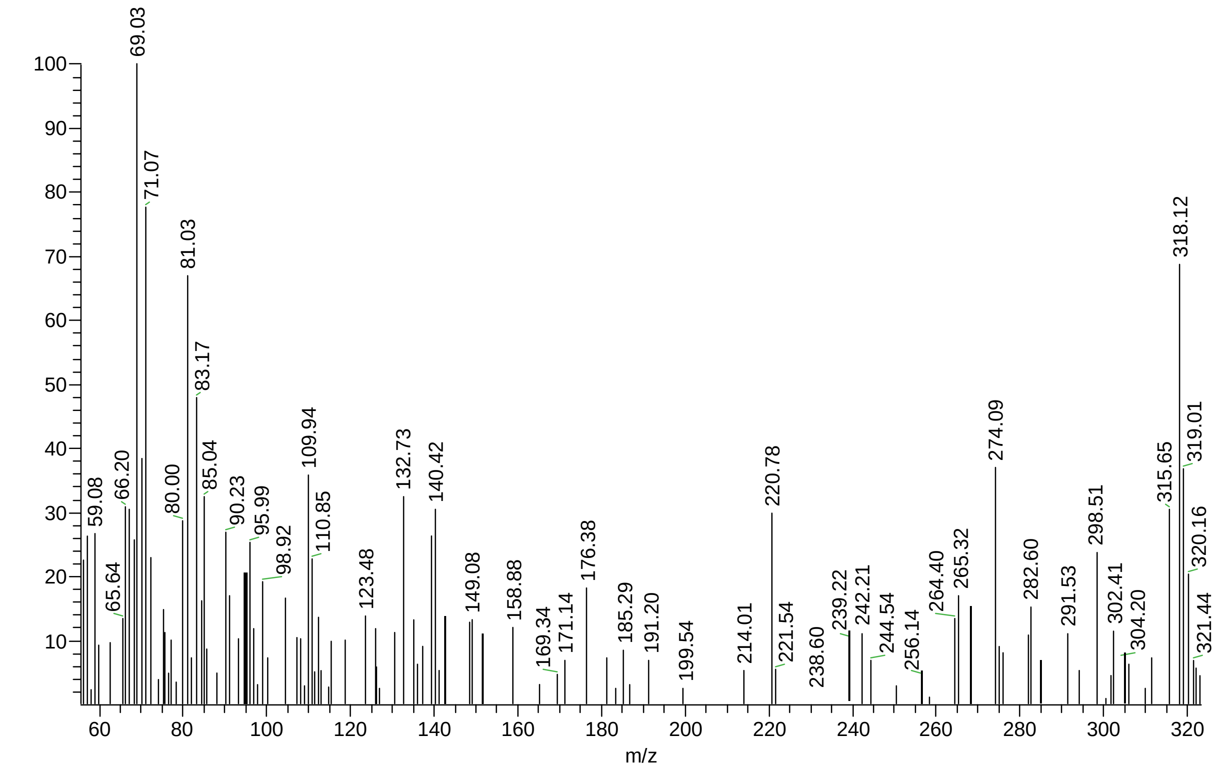

m/z	Intensity		Relative
56.18	498.9	22.68	
57.15	579.0	26.32	
58.14	51.5	2.34	
59.08	590.4	26.84	
59.95	203.9	9.27	
62.63	212.3	9.65	
65.64	298.4	13.56	
66.20	679.0	30.87	
67.10	672.0	30.55	
68.29	568.0	25.82	
69.03	2199.9		100.00
70.25	847.7	38.53	
71.07	1708.3		77.65
72.29	504.9	22.95	
74.08	87.9	3.99	
75.19	325.3	14.79	
75.75	248.4	11.29	
76.64	110.8	5.04	
77.27	223.6	10.16	
78.45	79.9	3.63	
80.00	632.6	28.76	
81.03	1473.6		66.99
82.16	161.3	7.33	

83.17	1053.4		47.88
84.31	356.1	16.19	
85.04	715.8	32.54	
85.62	189.9	8.63	
88.02	108.7	4.94	
90.23	595.0	27.05	
91.10	376.6	17.12	
93.16	226.5	10.30	
94.53	435.0	19.78	
95.18	414.2	18.83	
95.99	556.5	25.30	
96.99	261.3	11.88	
97.69	69.5	3.16	
98.92	422.8	19.22	
100.29	160.7	7.30	
104.52	365.2	16.60	
107.09	232.0	10.55	
108.26	224.7	10.21	
109.08	65.7	2.99	
109.94	790.6	35.94	
110.85	500.0	22.73	
111.50	114.7	5.21	
112.37	299.3	13.61	
113.09	117.3	5.33	
114.71	61.5	2.79	

115.29	217.1	9.87
118.84	223.3	10.15
123.48	305.3	13.88
124.70	114.1	5.19
125.41	56.1	2.55
126.15	260.6	11.85
127.08	58.4	2.65
130.47	247.5	11.25
132.73	715.6	32.53
135.15	294.0	13.36
136.08	140.4	6.38
137.10	201.5	9.16
139.44	580.1	26.37
140.42	670.6	30.48
141.34	118.9	5.41
142.31	286.1	13.01
148.46	281.6	12.80
149.08	290.4	13.20
152.16	140.0	6.36
153.62	226.1	10.28
158.88	267.2	12.15
165.08	67.9	3.08
169.34	105.5	4.79
171.14	151.2	6.87
176.38	400.6	18.21

181.22	162.9	7.41
183.27	55.7	2.53
185.29	185.3	8.42
186.75	71.3	3.24
191.20	150.7	6.85
199.54	55.6	2.53
214.01	117.7	5.35
220.78	657.6	29.89
221.54	119.7	5.44
238.60	34.3	1.56
239.22	225.9	10.27
242.21	245.3	11.15
244.54	152.8	6.95
250.41	64.5	2.93
256.14	98.0	4.45
264.40	297.6	13.53
265.32	375.4	17.07
266.21	321.0	14.59
274.09	814.7	37.04
275.21	202.1	9.19
276.06	178.3	8.10
281.92	240.5	10.93
282.60	335.8	15.27
284.00	135.5	6.16
291.53	245.3	11.15

294.15	116.0	5.27	
298.51	524.2	23.83	
301.73	101.2	4.60	
302.41	251.6	11.44	
304.20	161.5	7.34	
306.15	140.0	6.36	
309.87	55.2	2.51	
311.42	159.2	7.24	
315.65	669.7	30.44	
318.12	1513.0		68.78
319.01	810.5	36.84	
320.16	447.4	20.34	
321.44	152.9	6.95	
322.08	126.0	5.73	
323.09	98.9	4.50	

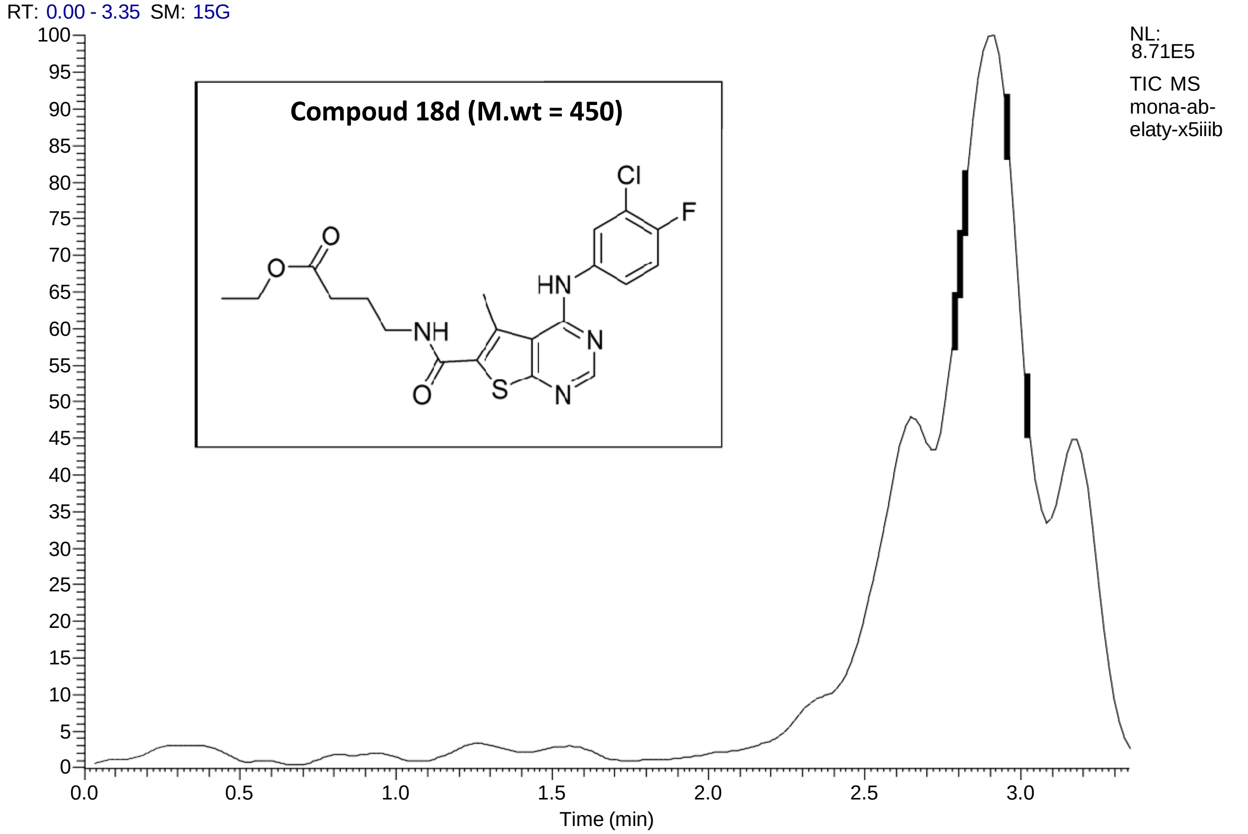
RT: 0.00 - 3.35 SM: 15G
NL: 8.71E5
TIC MS mona-ab-elaty-x5iiib
Compoud 18d (M.wt = 450)
Time (min)

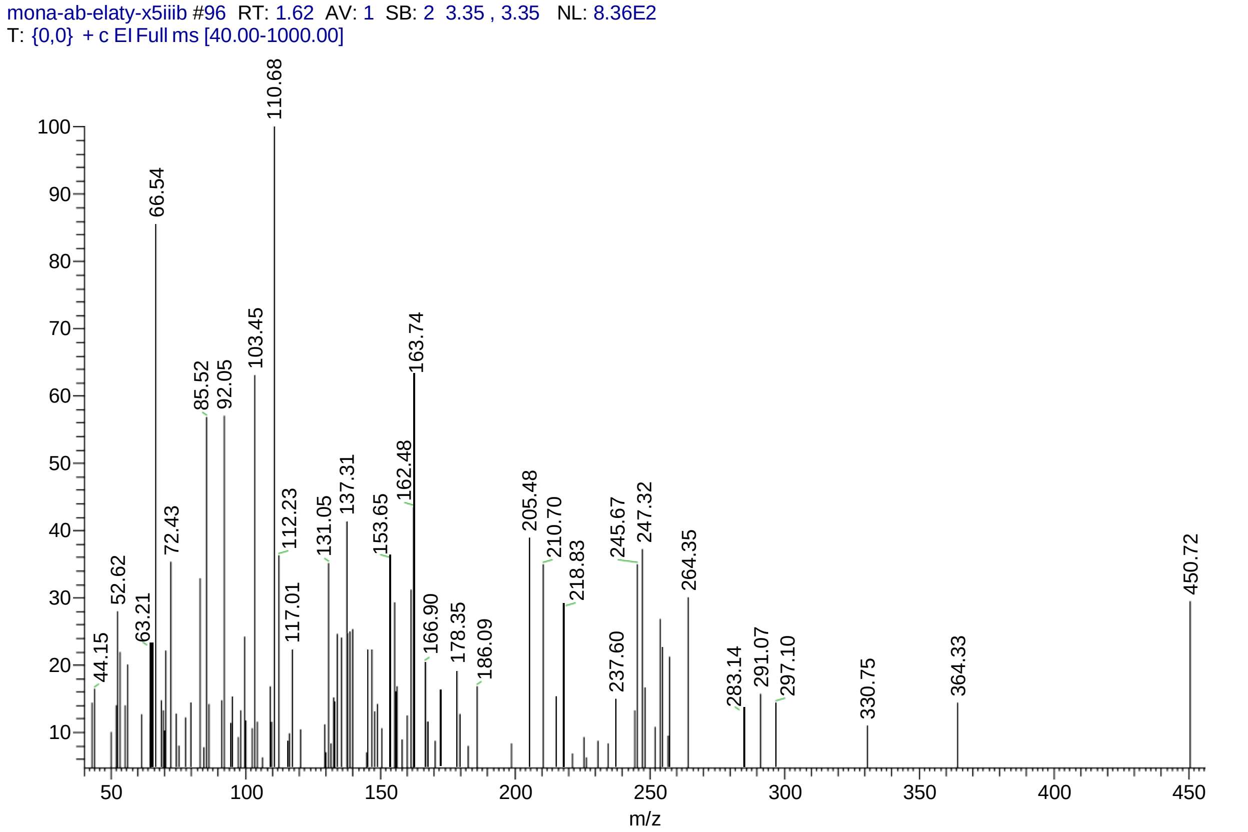
mona-ab-elaty-x5iiib #96 RT: 1.62 AV: 1 SB: 2 3.35 , 3.35 NL: 8.36E2
T: {0,0} + c EI Full ms [40.00-1000.00]
110.68
66.54
103.45
163.74
85.52
92.05
162.48
137.31
131.05
112.23
153.65
72.43
205.48
210.70
245.67
247.32
218.83
264.35
450.72
52.62
63.21
117.01
166.90
178.35
186.09
237.60
44.15
283.14
291.07
297.10
330.75
364.33
m/z

m/z	Intensity	Relative
42.97	120.8	14.45
44.15	137.9	16.49
50.22	83.9	10.03
51.89	116.9	13.99
52.62	234.3	28.02
53.29	183.6	21.96
55.12	116.5	13.94
56.39	167.3	20.01
61.68	105.2	12.58
62.44	139.2	16.65
63.21	189.3	22.64
64.60	94.1	11.26
66.54	715.2	85.54
68.68	122.7	14.67
69.29	110.7	13.24
69.82	84.8	10.14
70.49	185.5	22.18
72.43	295.3	35.32
74.09	106.4	12.73
74.98	66.0	7.89
77.35	101.6	12.15
79.56	120.5	14.42
83.01	274.1	32.79

84.62	64.4	7.70
85.52	475.4	56.86
86.62	117.7	14.08
91.08	122.7	14.67
92.05	477.6	57.12
94.42	94.0	11.24
94.95	127.3	15.23
97.26	77.1	9.22
98.22	110.1	13.17
99.65	202.8	24.25
100.29	97.3	11.64
102.42	88.7	10.60
103.45	527.7	63.12
104.16	95.7	11.45
106.07	52.7	6.30
109.10	140.7	16.82
109.61	96.0	11.48
110.68	836.1	100.00
112.23	303.6	36.31
115.49	72.8	8.71
116.15	82.8	9.90
117.01	187.1	22.37
120.27	87.1	10.41
129.39	93.2	11.15
130.08	58.8	7.03

131.05	294.1	35.18
131.80	70.3	8.40
132.51	125.7	15.04
133.18	121.2	14.50
134.04	204.9	24.51
135.75	200.9	24.03
137.31	345.8	41.37
137.90	207.3	24.80
138.94	209.1	25.00
140.01	211.3	25.28
144.75	58.8	7.03
145.64	186.7	22.32
147.01	187.1	22.37
147.64	109.6	13.11
148.62	117.9	14.10
150.51	88.4	10.57
152.41	85.2	10.19
153.65	298.0	35.64
155.17	244.7	29.26
155.68	134.1	16.04
156.36	140.8	16.84
158.13	73.7	8.82
160.08	104.1	12.45
161.46	260.9	31.21
162.48	363.8	43.52

163.74	530.1	63.40
164.63	117.1	14.00
166.90	170.1	20.35
167.75	95.6	11.43
170.49	72.7	8.69
171.09	117.9	14.10
172.89	130.3	15.58
178.35	160.3	19.17
179.31	106.3	12.71
182.61	66.3	7.93
186.09	140.4	16.79
198.88	70.0	8.37
205.48	324.6	38.83
210.70	291.4	34.86
215.40	127.9	15.29
217.51	102.0	12.20
218.83	238.3	28.50
221.34	56.1	6.71
225.74	76.9	9.20
226.40	51.9	6.20
231.00	72.1	8.63
234.40	69.6	8.32
237.60	125.2	14.97
244.65	111.2	13.30
245.67	291.8	34.91

247.32	310.9	37.19
248.14	138.9	16.62
251.92	89.6	10.72
254.01	223.9	26.77
254.68	189.3	22.64
256.67	79.2	9.47
257.36	177.1	21.18
264.35	251.7	30.11
283.14	108.5	12.98
291.07	130.9	15.66
297.10	119.7	14.32
330.75	91.5	10.94
364.33	120.4	14.40
450.72	245.9	29.41

C:\Xcalibur\...\S\mona-abd-elatty-x7111d 07-Dec-19 1:41:34 PM

R MYCOLOGY AND BIOTECHNOLOGY

RT: 3.22 - 4.11 SM: 15G

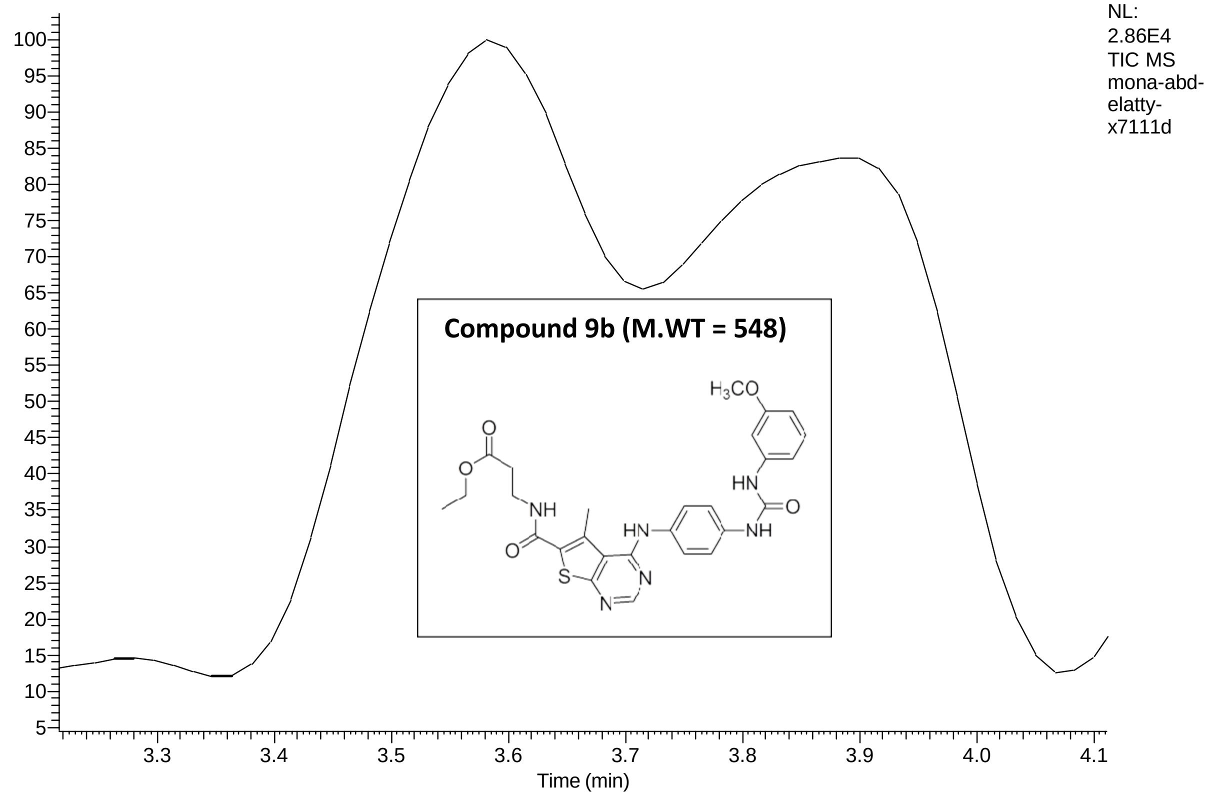

mona-abd-elatty-x7111d #213 RT: 3.58 AV: 1 SB: 2 4.45 , 4.45 NL: 3.21E2
T: {0,0} + c EI Full ms [40.00-1000.00]

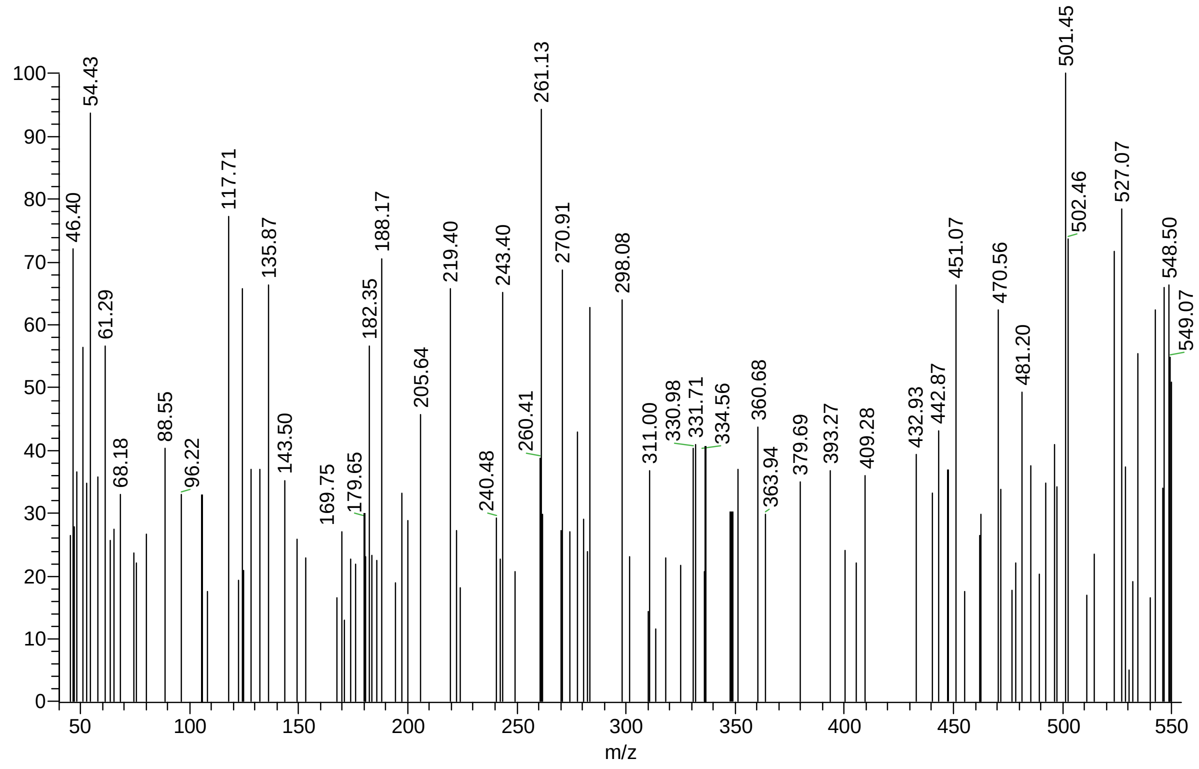

m/z	Intensity	Relative
40.30	138.1	43.09
45.38	85.1	26.54
46.40	231.3	72.17
47.29	89.6	27.95
48.50	117.6	36.69
51.36	181.1	56.49
52.89	111.3	34.73
54.43	300.8	93.84
58.04	114.5	35.73
61.29	181.3	56.57
63.69	82.5	25.75
65.62	88.4	27.58
68.18	106.1	33.11
74.28	76.0	23.71
75.56	71.1	22.17
80.02	85.5	26.66
88.55	129.3	40.35
96.22	106.0	33.07
105.88	103.1	32.15
108.06	56.5	17.64
117.71	247.6	77.25
122.28	62.1	19.38
124.13	210.8	65.77

124.84	67.3	21.01
128.18	118.8	37.06
131.89	118.4	36.94
135.87	212.8	66.39
143.50	112.8	35.19
149.35	83.2	25.96
153.42	73.2	22.84
167.48	52.9	16.51
169.75	86.9	27.12
170.98	41.9	13.06
173.82	73.1	22.80
175.88	70.1	21.88
179.65	93.7	29.24
180.79	74.1	23.13
182.35	181.6	56.66
183.74	74.7	23.29
185.83	72.4	22.59
188.17	226.1	70.55
194.21	60.9	19.01
197.27	106.7	33.28
199.94	92.8	28.95
205.64	146.7	45.76
219.40	210.5	65.68
222.27	87.5	27.29
224.21	58.0	18.09

240.48	93.9	29.28
242.55	72.7	22.67
243.40	209.2	65.27
249.41	66.4	20.72
260.41	124.5	38.85
261.13	302.4	94.34
261.88	96.0	29.95
270.26	87.3	27.25
270.91	220.7	68.84
274.06	87.1	27.16
277.62	137.6	42.93
280.74	93.5	29.16
282.47	76.9	24.00
283.65	201.2	62.77
298.08	204.9	63.94
301.85	74.1	23.13
310.40	46.0	14.35
311.00	118.3	36.90
313.38	37.3	11.65
318.32	73.7	23.00
324.82	69.6	21.71
330.98	129.3	40.35
331.71	131.1	40.89
333.02	49.3	15.39
334.56	128.0	39.93

335.60	66.7	20.80
345.87	94.3	29.41
347.27	71.7	22.38
347.89	92.7	28.91
351.35	118.4	36.94
360.68	140.5	43.84
363.94	95.6	29.83
379.69	112.3	35.02
393.27	118.0	36.81
400.44	77.5	24.17
405.72	70.7	22.05
409.28	115.7	36.11
432.93	126.0	39.31
440.49	106.4	33.19
442.87	138.3	43.14
447.79	115.9	36.15
451.07	212.7	66.35
455.12	56.1	17.51
461.86	84.8	26.46
462.53	95.7	29.87
470.56	200.0	62.40
471.53	108.7	33.90
476.84	56.9	17.76
478.32	70.7	22.05
481.20	158.1	49.33

485.32	120.5	37.60
489.39	64.9	20.26
492.19	111.7	34.86
495.99	131.5	41.01
497.24	110.0	34.32
501.45	320.5	100.00
502.46	236.4	73.75
510.98	54.1	16.89
514.67	75.3	23.50
523.85	230.1	71.80
527.07	251.3	78.41
528.94	119.6	37.31
530.68	16.3	5.07
531.92	61.6	19.22
534.51	177.5	55.37
539.98	52.9	16.51
542.14	200.1	62.44
545.59	109.1	34.03
546.39	211.6	66.01
548.50	212.8	66.39
549.07	175.9	54.87
550.05	162.9	50.83

C:\Xcalibur\...\S\mona-abd-elatty-x6111b 07-Dec-19 1:03:24 PM

R MYCOLOGY AND BIOTECHNOLOGY

RT: 0.00 - 5.50 SM: 15G

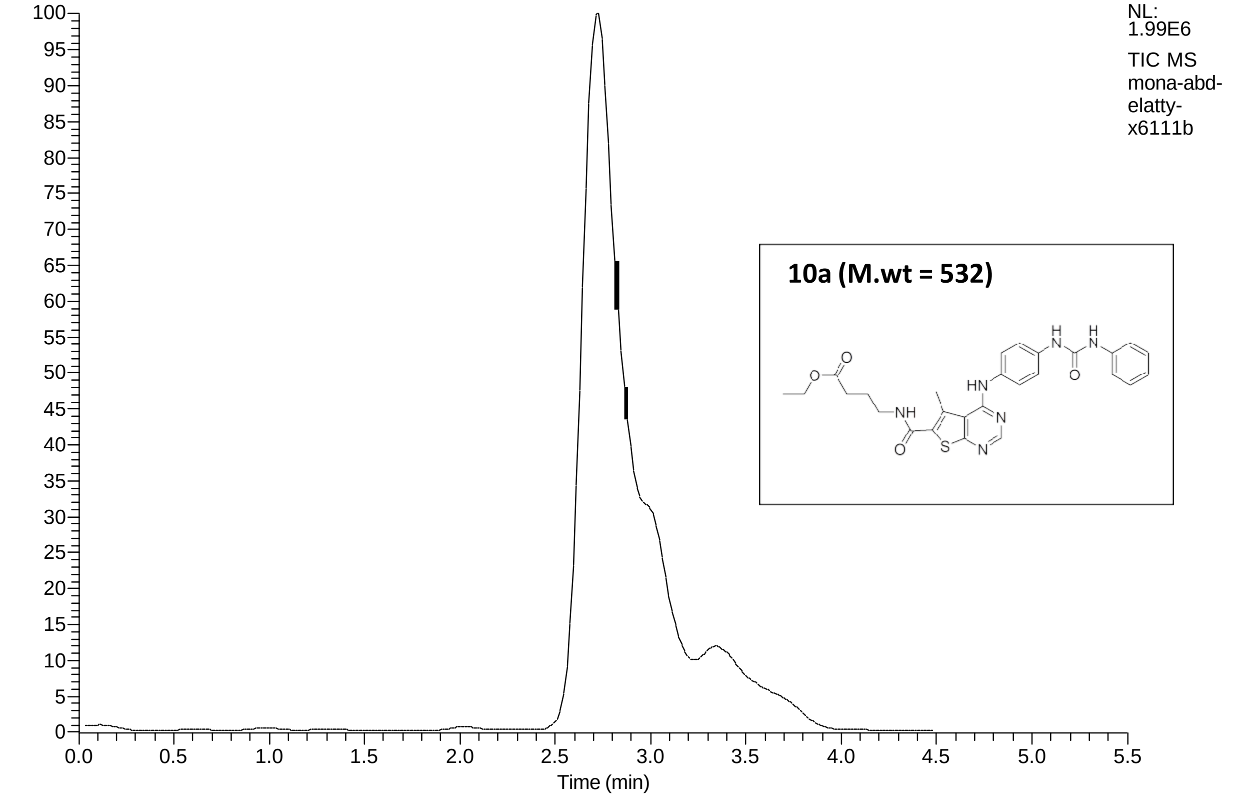

mona-abd-elatty-x6111b #236 RT: 3.97 AV: 1 SB: 2 4.45 , 4.45 NL: 3.42E2
T: {0,0} + c EI Full ms [40.00-1000.00]

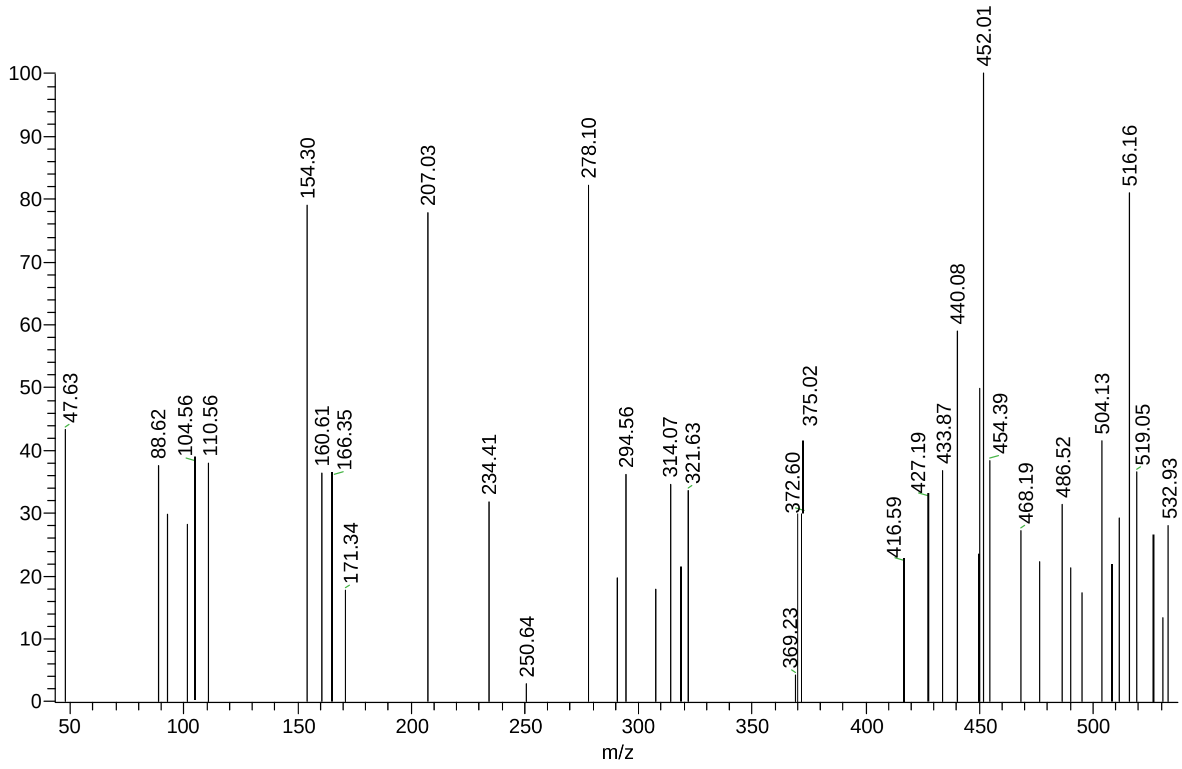

m/z	Intensity	Relative
47.63	148.5	43.45
88.62	128.8	37.68
92.93	101.9	29.80
101.55	96.8	28.32
104.56	129.6	37.91
110.56	130.1	38.07
154.30	270.4	79.10
160.61	124.8	36.51
166.35	122.4	35.80
171.34	60.4	17.67
207.03	266.0	77.81
234.41	109.1	31.90
250.64	10.1	2.96
278.10	281.0	82.22
290.80	67.2	19.66
294.56	123.6	36.15
307.67	61.5	17.98
314.07	118.5	34.67
318.67	70.8	20.71
321.63	114.7	33.54
369.23	14.5	4.25
372.60	102.9	30.11
375.02	146.0	42.71

416.59	75.3	22.04
427.19	111.2	32.53
433.87	125.6	36.74
440.08	201.7	59.01
449.34	80.1	23.44
450.41	170.3	49.80
452.01	341.8	100.00
454.39	131.1	38.34
468.19	93.3	27.30
476.68	76.3	22.31
486.52	107.3	31.40
490.05	73.2	21.41
495.12	59.3	17.36
504.13	142.3	41.61
509.18	72.5	21.22
511.66	99.9	29.21
516.16	277.0	81.05
519.05	124.9	36.54
527.08	88.4	25.86
530.52	45.9	13.42
532.93	95.9	28.04

C:\Xcalibur\...\S\mona-abd-elatty-x6111d 07-Dec-19 12:38:46 PM

R MYCOLOGY AND BIOTECHNOLOGY

RT: 1.49 - 2.25 SM: 15G

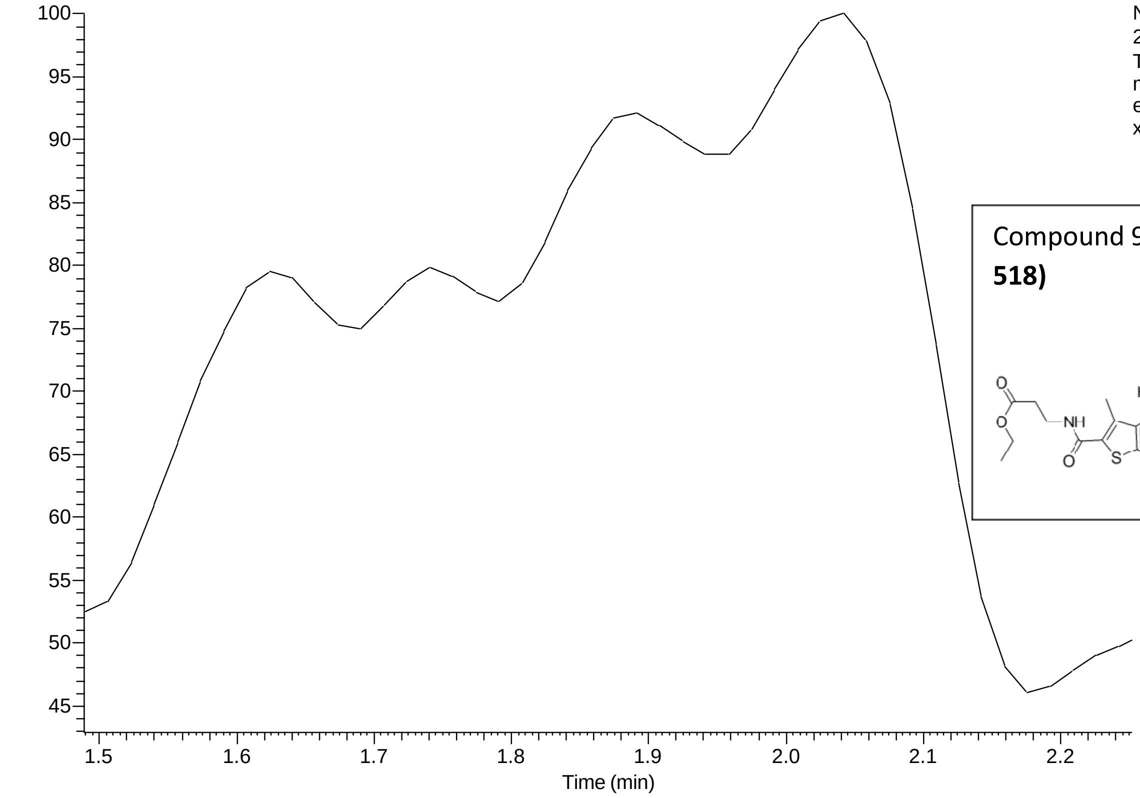

Compound 9a **(M.wt = 518)**

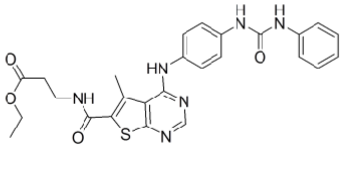

mona-abd-elatty-x6111d #95 RT: 1.61 AV: 1 SB: 2 4.45 , 4.45 NL: 2.79E2
T: {0,0} + c EI Full ms [40.00-1000.00]

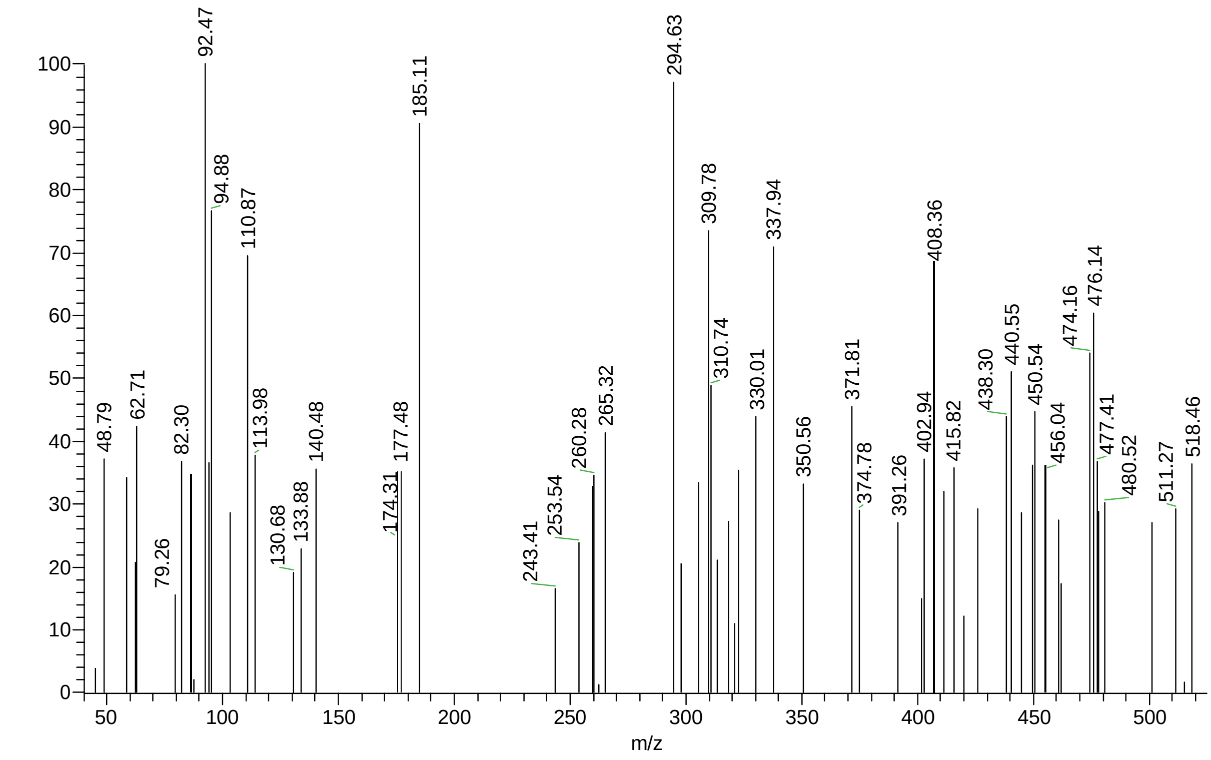

m/z	Intensity	Relative
44.84	10.5	3.77
48.79	104.0	37.23
58.36	95.6	34.22
62.15	58.0	20.76
62.71	118.4	42.39
79.26	43.5	15.56
82.30	102.5	36.71
86.75	94.8	33.94
87.85	5.7	2.05
92.47	279.3	100.00
94.10	102.3	36.61
94.88	214.1	76.66
103.08	80.3	28.74
110.87	194.1	69.50
113.98	105.5	37.76
130.68	53.3	19.09
133.88	64.3	23.01
140.48	99.6	35.66
174.31	69.1	24.73
177.48	100.4	35.94
185.11	252.8	90.50
243.41	46.5	16.66
253.54	66.5	23.82

259.70	91.9	32.89
260.28	96.7	34.61
262.38	3.7	1.34
265.32	115.6	41.38
294.63	271.2	97.09
297.96	57.5	20.57
305.66	93.5	33.46
309.78	205.3	73.51
310.74	136.4	48.83
313.33	58.8	21.05
318.06	76.3	27.30
320.84	30.5	10.93
322.54	99.1	35.47
330.01	122.9	44.01
337.94	198.1	70.93
350.56	92.7	33.17
371.81	126.9	45.44
374.78	81.3	29.12
391.26	75.5	27.02
401.52	41.9	14.99
402.94	104.0	37.23
406.81	81.1	29.02
408.36	192.0	68.74
411.20	89.7	32.12
415.82	100.3	35.89

419.96	33.9	12.12
425.94	81.6	29.21
438.30	122.9	44.01
440.55	142.5	51.03
444.87	79.9	28.59
449.73	101.2	36.23
450.54	125.1	44.77
456.04	99.1	35.47
460.85	76.5	27.40
461.87	48.4	17.33
474.16	150.8	53.99
476.14	168.5	60.33
477.41	102.9	36.85
477.93	80.8	28.93
480.52	84.5	30.26
501.39	75.6	27.06
511.27	81.7	29.26
515.30	4.7	1.67
518.46	101.7	36.42

C:\Xcalibur\data\S\mona-abdelatty-x2111c 05-Dec-19 2:12:37 PM

R MYCOLOGY AND BIOTECHNOLOGY

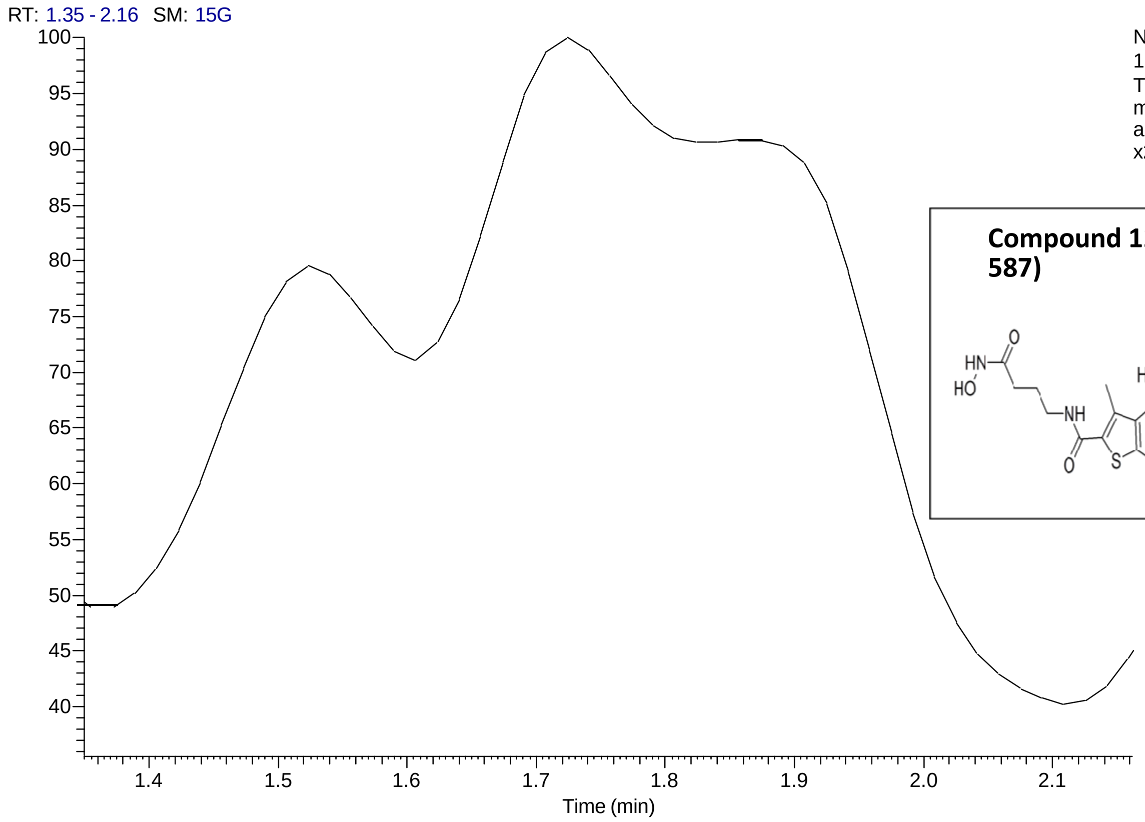

Compound 12c (Mwt = 587)

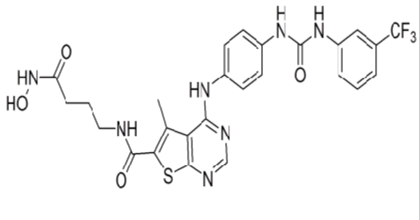

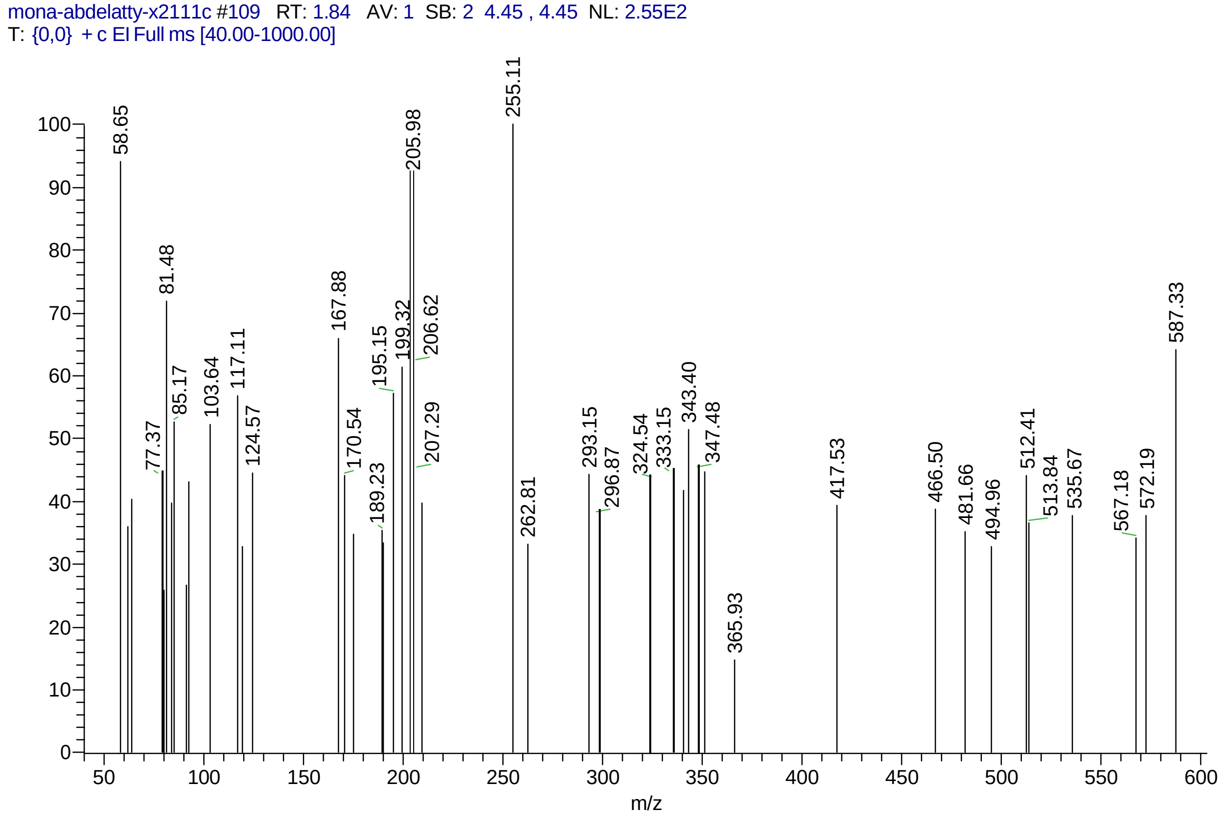

m/z	Intensity	Relative
58.65	239.5	94.08
62.35	91.7	36.04
63.78	102.7	40.34
77.37	112.3	44.11
80.51	66.0	25.93
81.48	182.9	71.87
84.21	101.3	39.81
85.17	134.1	52.70
91.40	68.1	26.77
92.91	109.9	43.16
103.64	133.2	52.33
117.11	144.7	56.84
119.74	83.5	32.79
124.57	113.2	44.47
167.88	167.9	65.95
170.54	112.3	44.11
174.95	88.8	34.89
189.23	90.1	35.41
190.40	84.9	33.37
195.15	145.6	57.20
199.32	156.3	61.39
205.98	236.1	92.77
206.62	158.4	62.23

207.29	114.9	45.15
209.73	101.1	39.71
255.11	254.5	100.00
262.81	84.5	33.21
293.15	112.8	44.32
296.87	96.7	37.98
324.54	110.7	43.48
333.15	113.2	44.47
340.58	106.4	41.80
343.40	130.9	51.44
347.48	115.1	45.21
351.02	113.7	44.68
365.93	37.9	14.88
417.53	100.0	39.29
466.50	98.7	38.76
481.66	89.5	35.15
494.96	83.7	32.90
512.41	112.4	44.16
513.84	92.9	36.51
535.67	96.4	37.87
567.18	87.2	34.26
572.19	96.3	37.82
587.33	163.6	64.27

C:\Xcalibur\...\S\mona-abd-elatty-x4111d 05-Dec-19 1:54:00 PM

R MYCOLOGY AND BIOTECHNOLOGY

RT: 0.84 - 1.30 SM: 15G

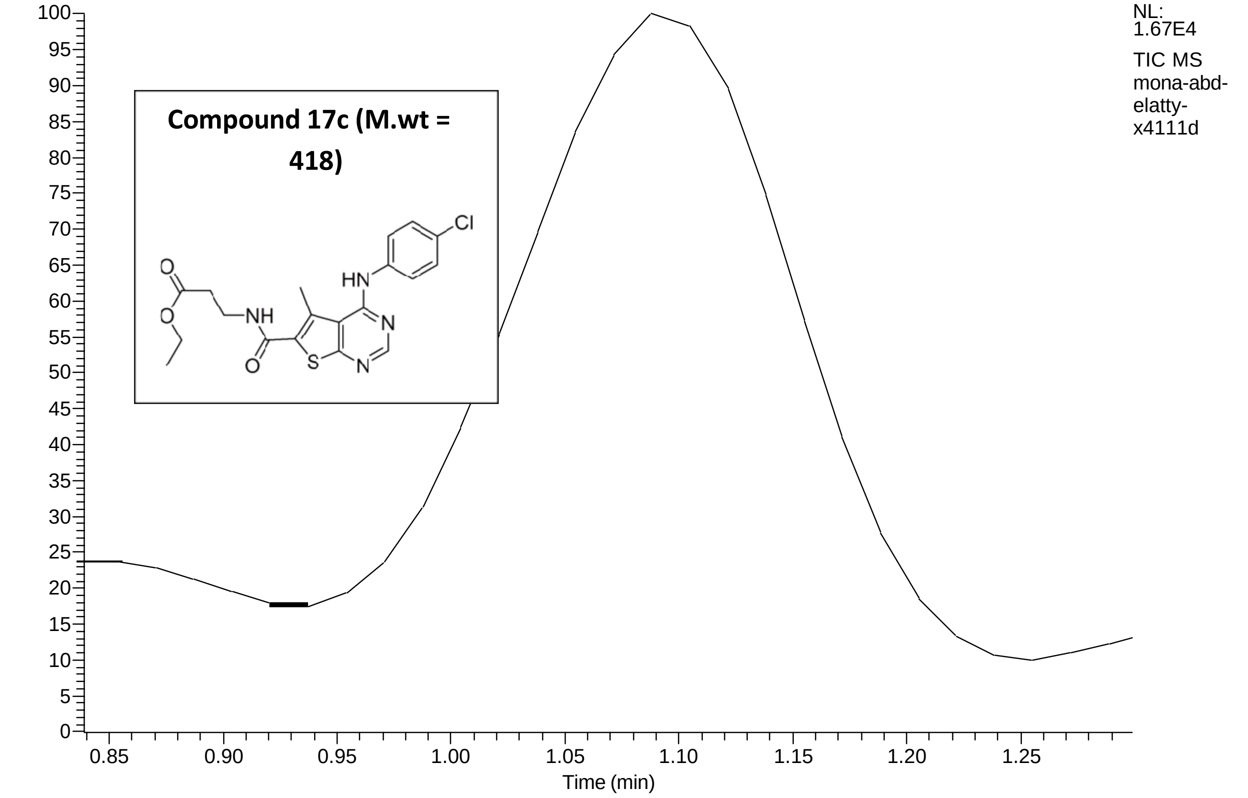

mona-abd-elatty-x4111d #63 RT: 1.07 AV: 1 SB: 2 4.45 , 4.45 NL: 4.02E2
T: {0,0} + c EI Full ms [40.00-1000.00]

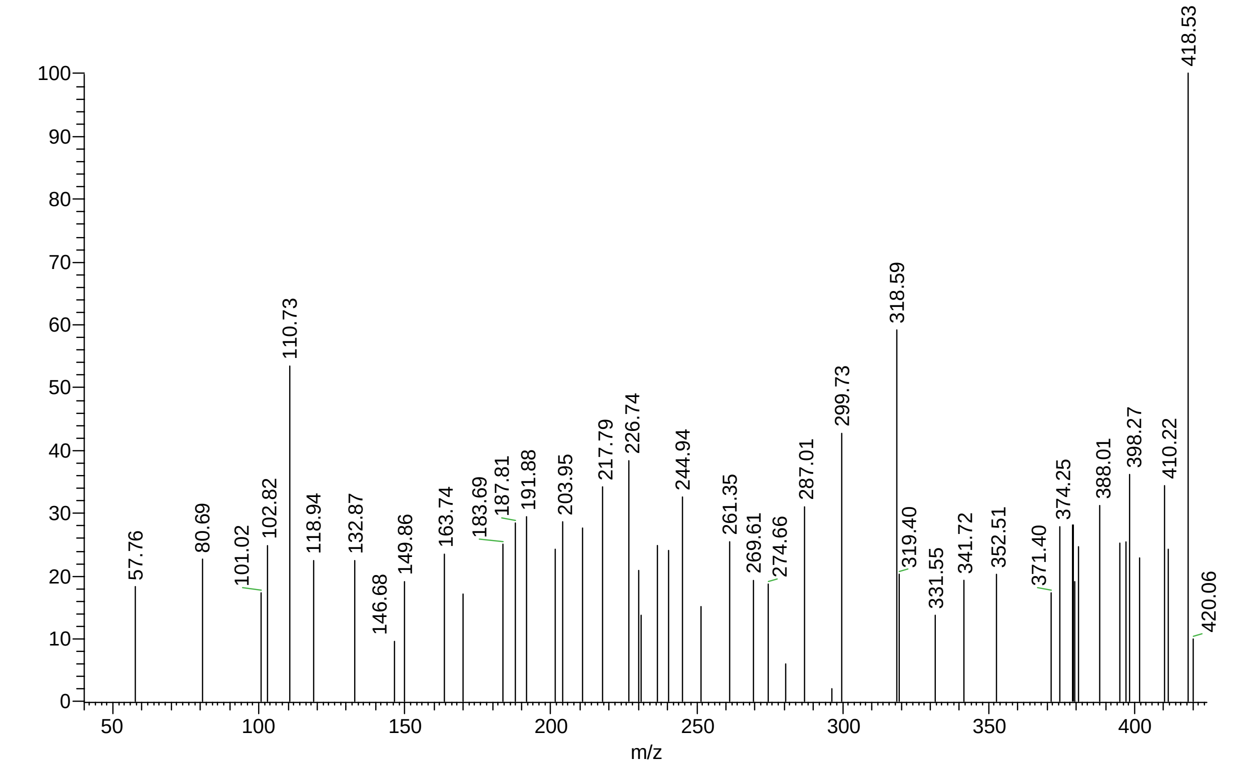

m/z	Intensity	Relative
57.76	74.1	18.44
80.69	91.5	22.75
101.02	69.5	17.27
102.82	99.9	24.83
110.73	214.7	53.38
118.94	90.3	22.45
132.87	90.8	22.58
146.68	38.8	9.65
149.86	76.7	19.06
163.74	94.3	23.44
169.78	68.8	17.11
183.69	100.9	25.10
187.81	114.7	28.51
191.88	118.8	29.54
201.63	97.7	24.30
203.95	115.1	28.61
210.91	111.3	27.69
217.79	138.0	34.32
226.74	154.5	38.43
230.05	84.3	20.95
230.83	55.6	13.83
236.36	100.1	24.90
240.45	97.1	24.14

244.94	131.1	32.59
251.67	60.9	15.15
261.35	102.3	25.43
269.61	77.9	19.36
274.66	75.1	18.67
280.47	24.7	6.13
287.01	124.7	31.00
296.19	8.3	2.06
299.73	172.0	42.77
318.59	238.5	59.32
319.40	82.1	20.42
331.55	55.5	13.79
341.72	77.7	19.33
352.51	82.0	20.39
371.40	69.7	17.34
374.25	111.7	27.79
377.10	92.7	23.04
378.53	110.4	27.45
379.53	77.1	19.16
380.80	99.1	24.64
388.01	125.6	31.23
394.97	101.3	25.20
397.06	102.3	25.43
398.27	145.6	36.21
401.59	91.9	22.84

410.22	138.7	34.48
411.73	97.6	24.27
418.53	402.1	100.00
420.06	40.3	10.01

C:\Xcalibur\...\S\mona-abd-elatty-x7111b 05-Dec-19 1:43:57 PM

R MYCOLOGY AND BIOTECHNOLOGY

RT: 0.00 - 5.50 SM: 15G

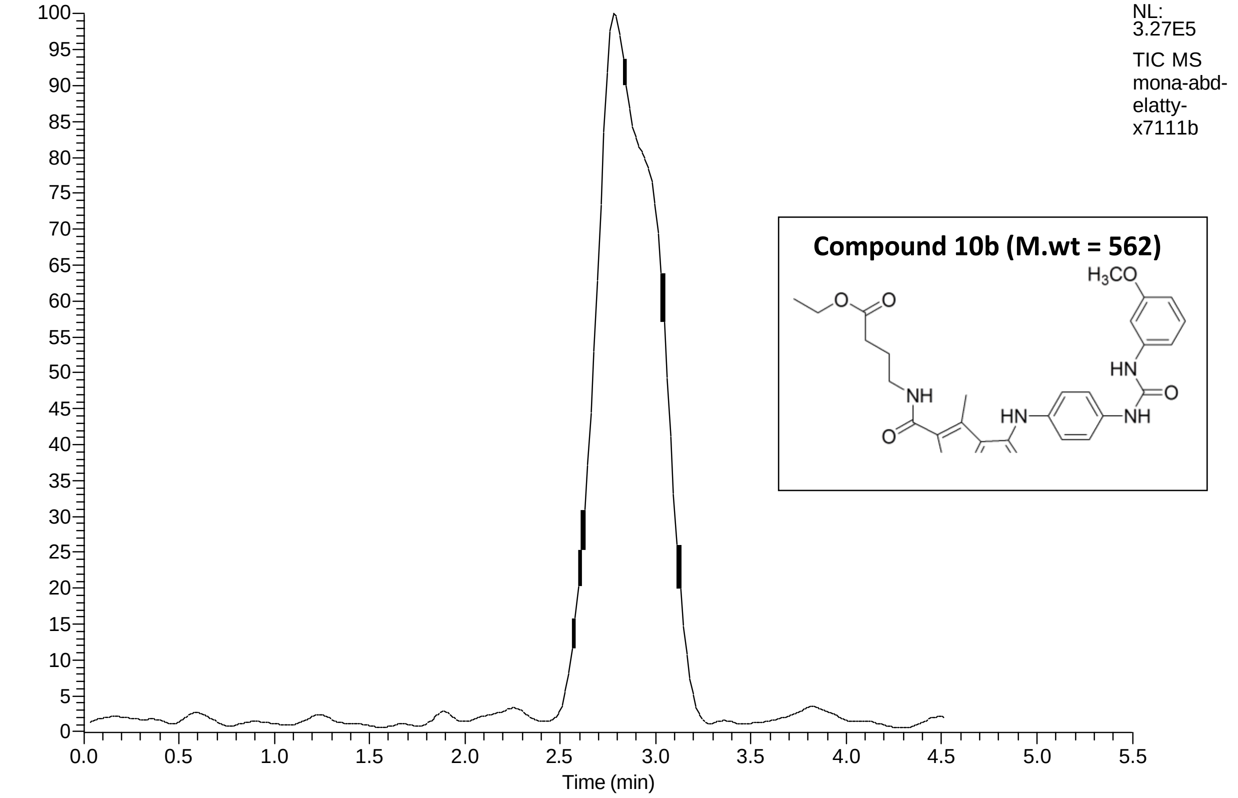

Compound 10b (M.wt = 562)

mona-abd-elatty-x7111b #226 RT: 3.80 AV: 1 SB: 2 4.45 , 4.45 NL: 3.12E2
T: {0,0} + c EI Full ms [40.00-1000.00]

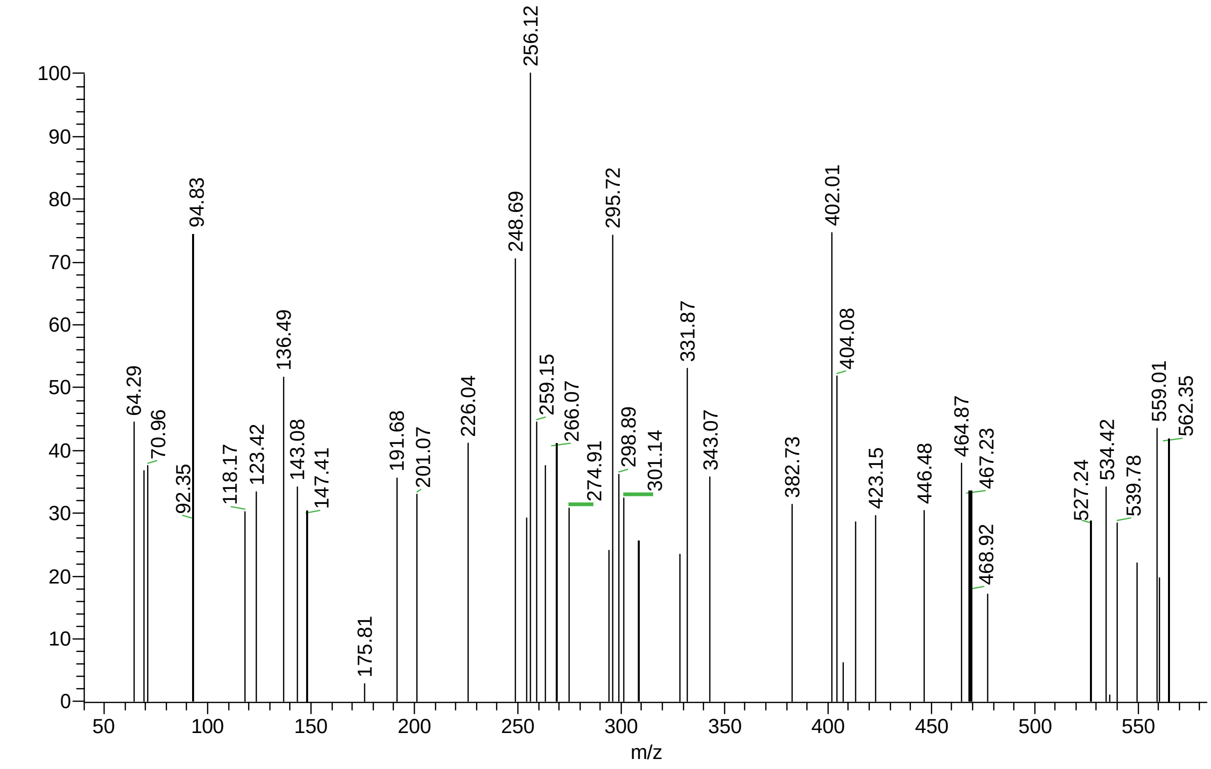

m/z	Intensity	Relative
64.29	139.1	44.63
69.51	114.8	36.84
70.96	117.2	37.61
92.35	89.7	28.80
94.83	232.3	74.54
118.17	94.1	30.21
123.42	104.4	33.50
136.49	161.2	51.73
143.08	106.9	34.32
147.41	92.1	29.57
175.81	9.1	2.91
191.68	110.9	35.60
201.07	103.1	33.08
226.04	128.3	41.16
248.69	219.7	70.52
254.28	91.1	29.23
256.12	311.6	100.00
259.15	138.7	44.50
263.49	116.9	37.53
266.07	126.0	40.44
274.91	96.4	30.94
294.20	75.2	24.13
295.72	231.6	74.33

298.89	112.9	36.24
301.14	100.9	32.39
306.40	77.9	24.99
328.45	73.5	23.58
331.87	165.5	53.10
343.07	111.5	35.77
382.73	98.3	31.54
402.01	233.1	74.80
404.08	161.5	51.82
407.48	19.3	6.20
413.49	89.1	28.58
423.15	92.3	29.61
446.48	95.2	30.55
464.87	118.5	38.04
467.23	102.5	32.91
468.26	98.3	31.54
468.92	54.7	17.54
476.98	53.3	17.12
527.24	87.3	28.03
534.42	106.4	34.15
539.78	88.7	28.46
549.39	69.1	22.17
559.01	135.6	43.52
560.29	61.5	19.73
562.35	128.1	41.12

RT: 1.54 - 1.78 SM: 15G

NL: 2.54E4 TIC MS mona-abd-elatty-x5111c

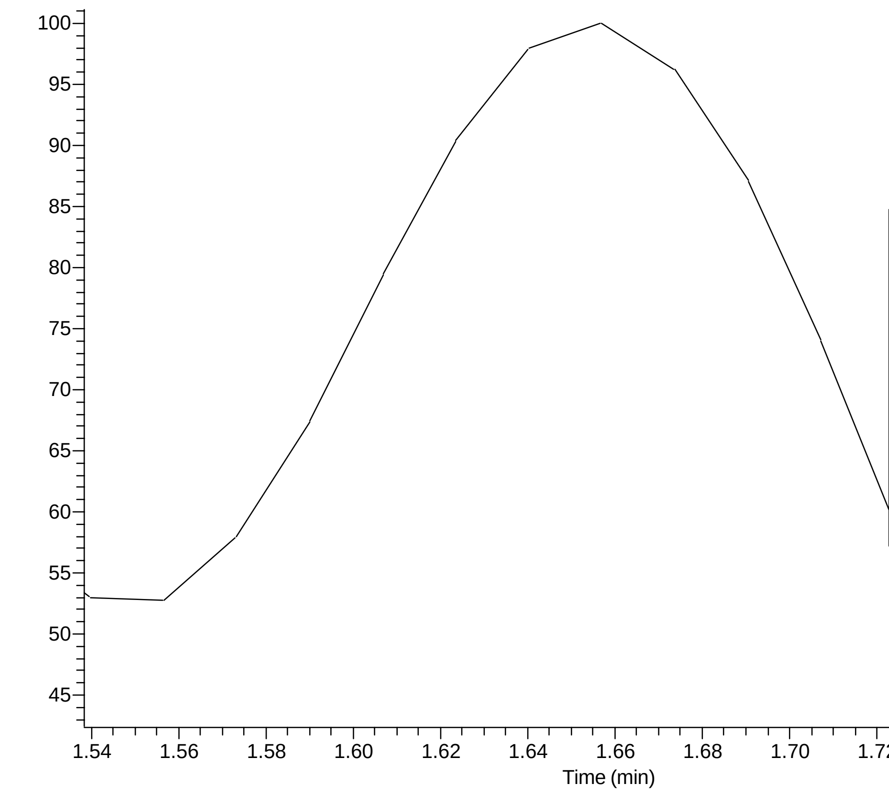

Compound 20d (M.wt = 437)

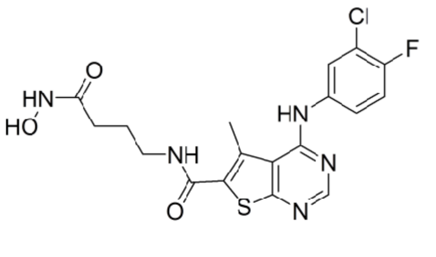

mona-abd-elatty-x5111c #97 RT: 1.64 AV: 1 SB: 2 4.45 , 4.45 NL: 5.81E2
T: {0,0} + c EI Full ms [40.00-1000.00]

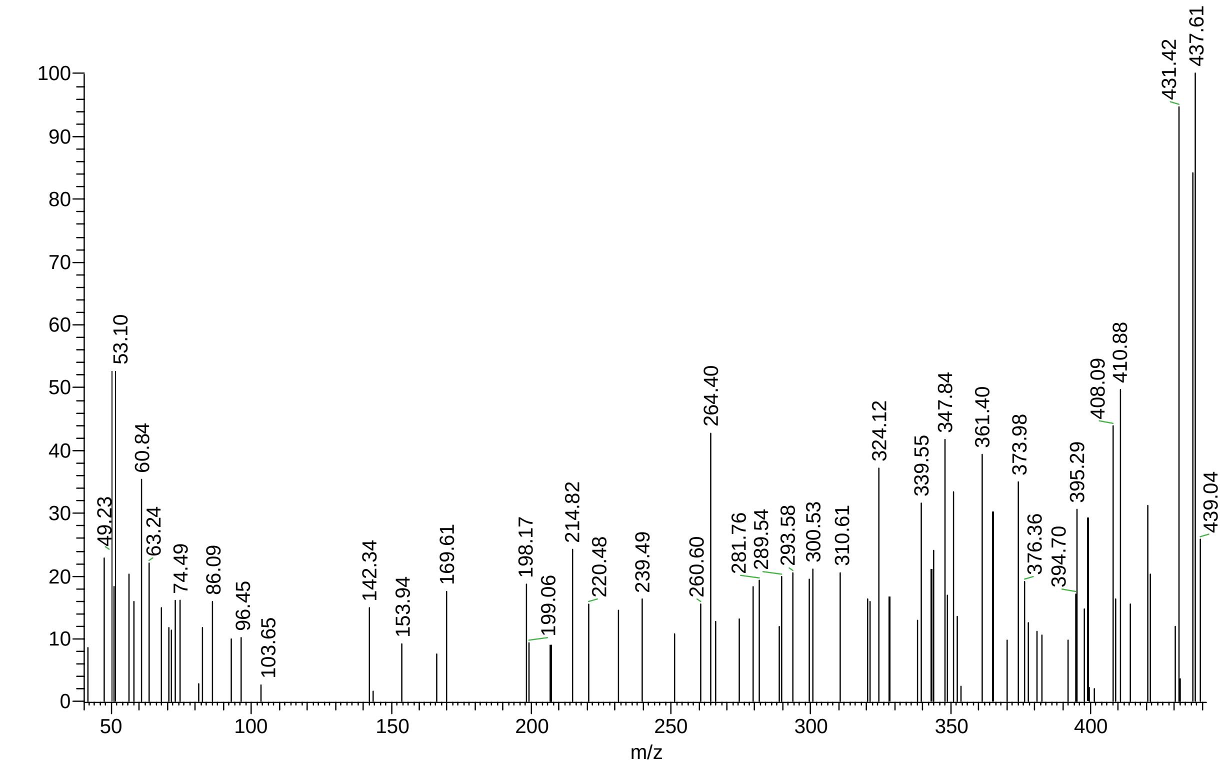

m/z	Intensity	Relative
41.48	50.3	8.66
47.16	133.6	23.01
49.23	138.4	23.83
51.05	106.9	18.42
53.10	305.3	52.58
56.29	118.7	20.44
58.04	92.7	15.96
60.84	205.2	35.34
63.24	128.7	22.16
67.71	87.3	15.04
70.61	69.1	11.89
71.24	66.3	11.41
72.56	93.3	16.07
74.49	93.3	16.07
81.11	17.1	2.94
82.60	68.9	11.87
86.09	92.4	15.91
92.62	57.9	9.97
96.45	59.3	10.22
103.65	15.3	2.64
142.34	87.1	14.99
143.63	10.0	1.72
153.94	53.7	9.25

166.35	44.1	7.60
169.61	102.1	17.59
198.17	108.8	18.74
199.06	54.5	9.39
206.68	52.5	9.05
207.35	52.8	9.09
214.82	140.7	24.23
220.48	90.5	15.59
231.44	84.7	14.58
239.49	94.5	16.28
251.41	63.2	10.88
260.60	90.0	15.50
264.40	248.1	42.73
265.96	74.0	12.74
274.34	76.8	13.23
279.26	107.1	18.44
281.76	112.0	19.29
288.76	70.3	12.10
289.54	115.9	19.95
293.58	119.3	20.55
299.25	112.9	19.45
300.53	123.2	21.22
310.61	119.3	20.55
320.22	94.9	16.35
321.07	93.1	16.03

324.12	215.9	37.18
327.58	93.2	16.05
338.08	75.3	12.97
339.55	184.3	31.73
342.77	20.0	3.44
343.28	118.4	20.39
343.87	139.6	24.04
347.84	242.1	41.70
348.70	98.7	16.99
350.94	193.9	33.39
352.47	78.9	13.59
353.80	14.5	2.50
361.40	228.9	39.43
364.65	73.3	12.63
366.92	171.6	29.55
369.98	56.9	9.80
373.98	203.1	34.97
376.36	111.3	19.17
377.61	72.7	12.51
380.67	64.5	11.11
382.53	62.1	10.70
392.13	56.8	9.78
394.70	100.0	17.22
395.29	178.3	30.70
396.25	165.1	28.43

397.25	90.1	15.52
397.76	85.7	14.76
399.45	12.8	2.20
401.31	12.7	2.18
408.09	255.1	43.93
408.88	95.3	16.42
410.88	288.1	49.62
414.07	90.4	15.57
420.56	181.1	31.18
421.20	118.0	20.32
430.26	70.1	12.08
431.42	550.0	94.72
432.19	21.1	3.63
436.61	489.2	84.25
437.61	580.6	100.00
439.04	150.7	25.95

C:\Xcalibur\...\S\mona-abd-elatty-x7111e 05-Dec-19 1:19:32 PM

R MYCOLOGY AND BIOTECHNOLOGY

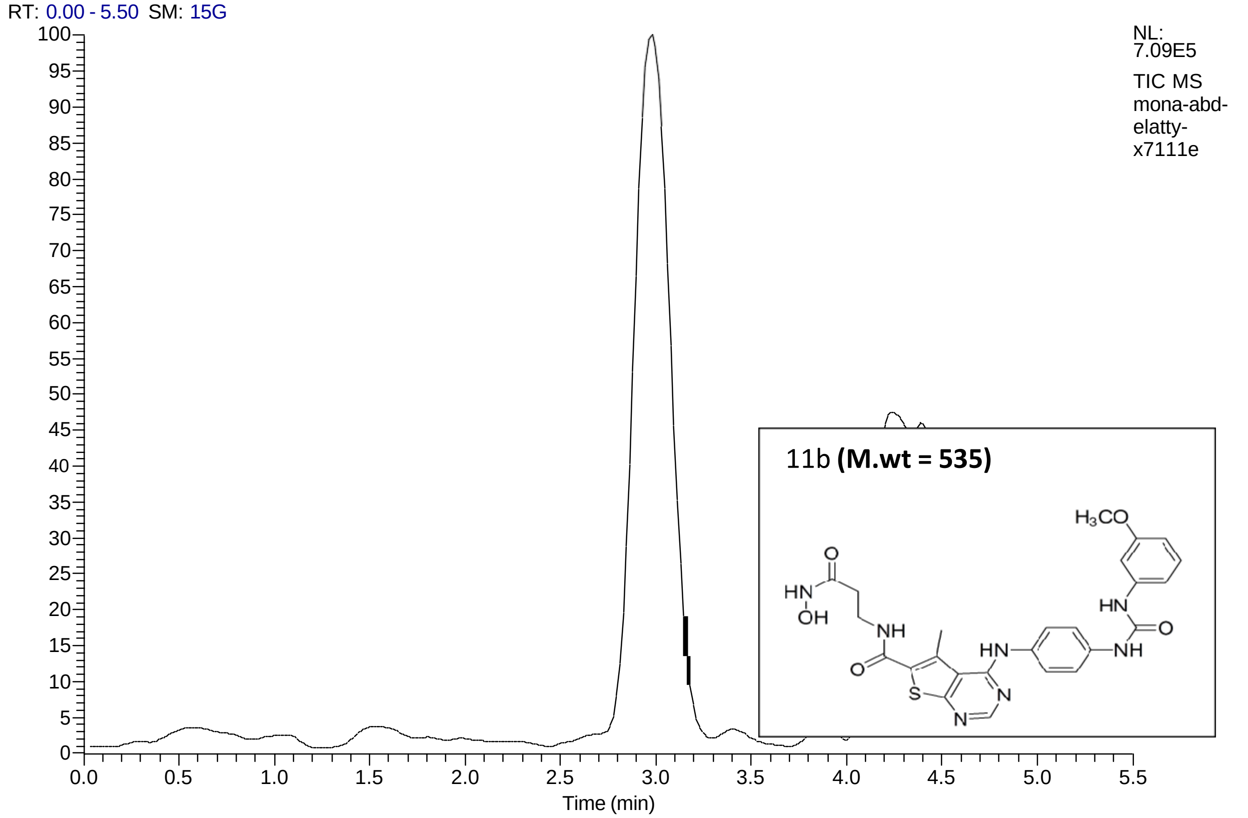

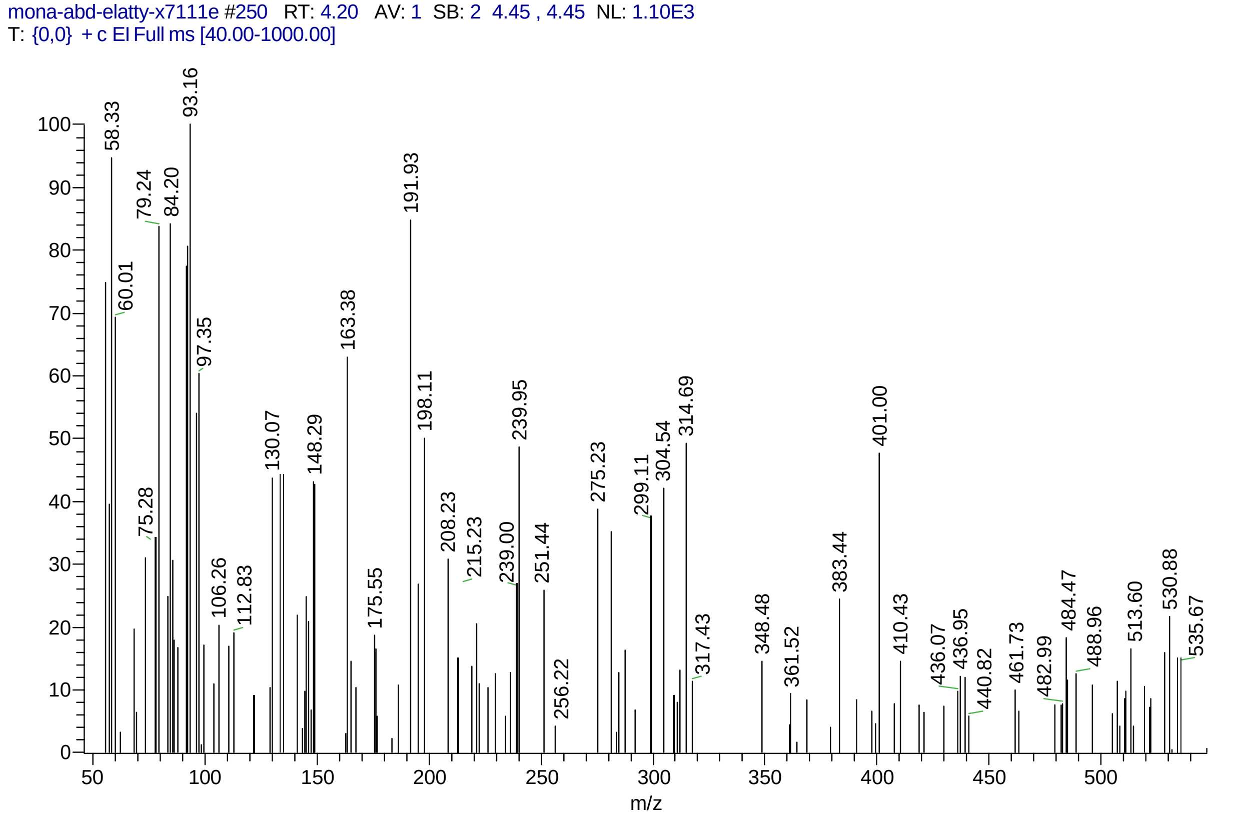

m/z	Intensity		Relative
55.18	822.2	74.81	
57.26	435.6	39.63	
58.33	1040.5		94.66
60.01	762.1	69.34	
62.24	36.8	3.35	
68.14	216.3	19.67	
69.16	71.7	6.53	
73.25	342.4	31.15	
75.28	370.0	33.66	
79.24	921.5	83.84	
83.11	272.6	24.81	
84.20	925.9	84.24	
85.43	336.0	30.57	
86.09	197.9	18.00	
87.87	184.8	16.81	
91.33	850.6	77.39	
92.27	886.5	80.65	
93.16	1099.1		100.00
96.23	595.3	54.16	
97.35	663.2	60.33	
97.99	14.7	1.33	
99.29	188.4	17.14	
103.95	120.0	10.92	
106.26	223.9	20.37	

110.33	187.5	17.05
112.83	210.5	19.15
122.50	91.7	8.35
128.81	114.9	10.46
130.07	480.1	43.68
132.28	478.8	43.56
133.18	26.4	2.40
136.15	318.4	28.97
141.22	241.6	21.98
143.40	41.5	3.77
144.32	107.9	9.81
145.21	273.6	24.89
146.08	230.8	21.00
147.19	74.4	6.77
148.29	473.4	43.07
149.02	470.8	42.83
162.85	33.2	3.02
163.38	691.6	62.92
165.09	161.2	14.67
167.16	114.9	10.46
175.55	205.9	18.73
176.15	181.7	16.53
176.70	64.3	5.85
183.37	26.1	2.38
186.13	119.6	10.88

191.93	932.5	84.84
195.15	296.2	26.95
198.11	551.4	50.17
208.23	340.1	30.94
214.15	157.2	14.30
215.23	295.0	26.84
218.88	150.9	13.73
221.22	226.1	20.57
222.35	120.9	11.00
226.15	114.0	10.37
229.82	137.7	12.53
234.04	63.5	5.77
236.31	140.7	12.80
239.00	289.3	26.32
239.95	534.9	48.67
251.44	284.2	25.86
256.22	46.4	4.22
275.23	426.0	38.76
281.32	387.0	35.21
283.55	35.1	3.19
284.53	140.4	12.77
287.49	180.5	16.42
291.87	74.8	6.80
299.11	405.7	36.91
304.54	462.6	42.09

307.88	91.9	8.36
311.05	87.7	7.98
311.69	144.7	13.16
314.69	540.9	49.21
317.43	124.9	11.37
348.48	159.6	14.52
360.70	49.5	4.50
361.52	102.7	9.34
364.13	19.5	1.77
368.86	91.9	8.36
379.47	44.0	4.00
383.44	269.8	24.55
390.94	93.1	8.47
397.44	72.3	6.57
399.19	51.6	4.69
401.00	523.4	47.62
407.93	86.3	7.85
410.43	159.5	14.51
418.72	83.6	7.61
421.29	70.4	6.40
429.66	81.1	7.38
436.07	107.2	9.75
436.95	133.3	12.13
439.20	131.1	11.92
440.82	64.7	5.88

461.73	110.8	10.08
463.50	74.1	6.74
479.31	84.0	7.64
482.42	83.2	7.57
482.99	86.4	7.86
484.47	202.8	18.45
485.06	127.9	11.63
488.96	139.3	12.68
495.92	118.3	10.76
505.05	69.5	6.32
507.39	125.6	11.43
510.46	93.9	8.54
511.01	108.8	9.90
513.60	182.5	16.61
514.59	47.9	4.35
516.94	107.9	9.81
517.52	79.6	7.24
521.72	79.7	7.25
522.52	94.8	8.62
528.66	175.3	15.95
530.88	238.3	21.68
534.25	120.5	10.97
534.98	151.7	13.80
535.67	158.3	14.40
537.85	86.9	7.91

C:\Xcalibur\data\S\mona-abd-elatty-x6iv

R MYCOLOGY AND BIOTECHNOLOGY

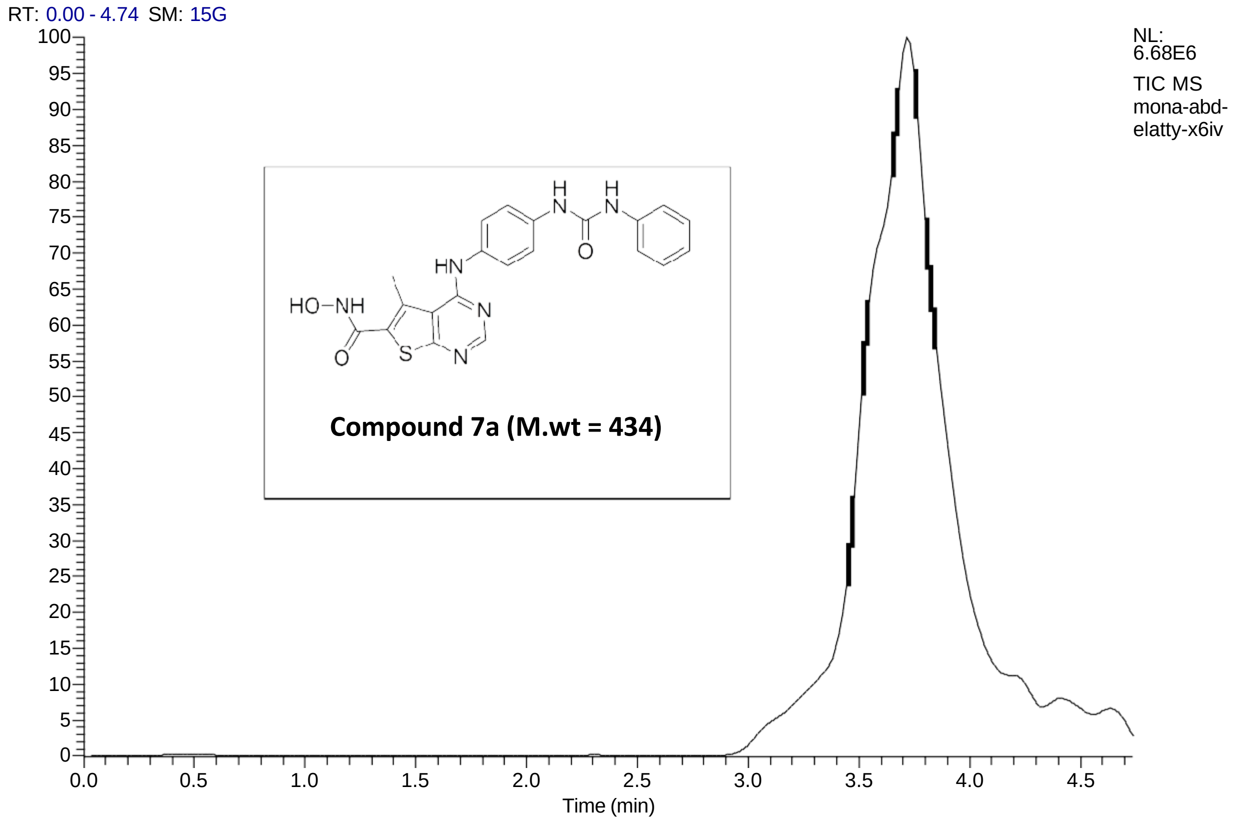

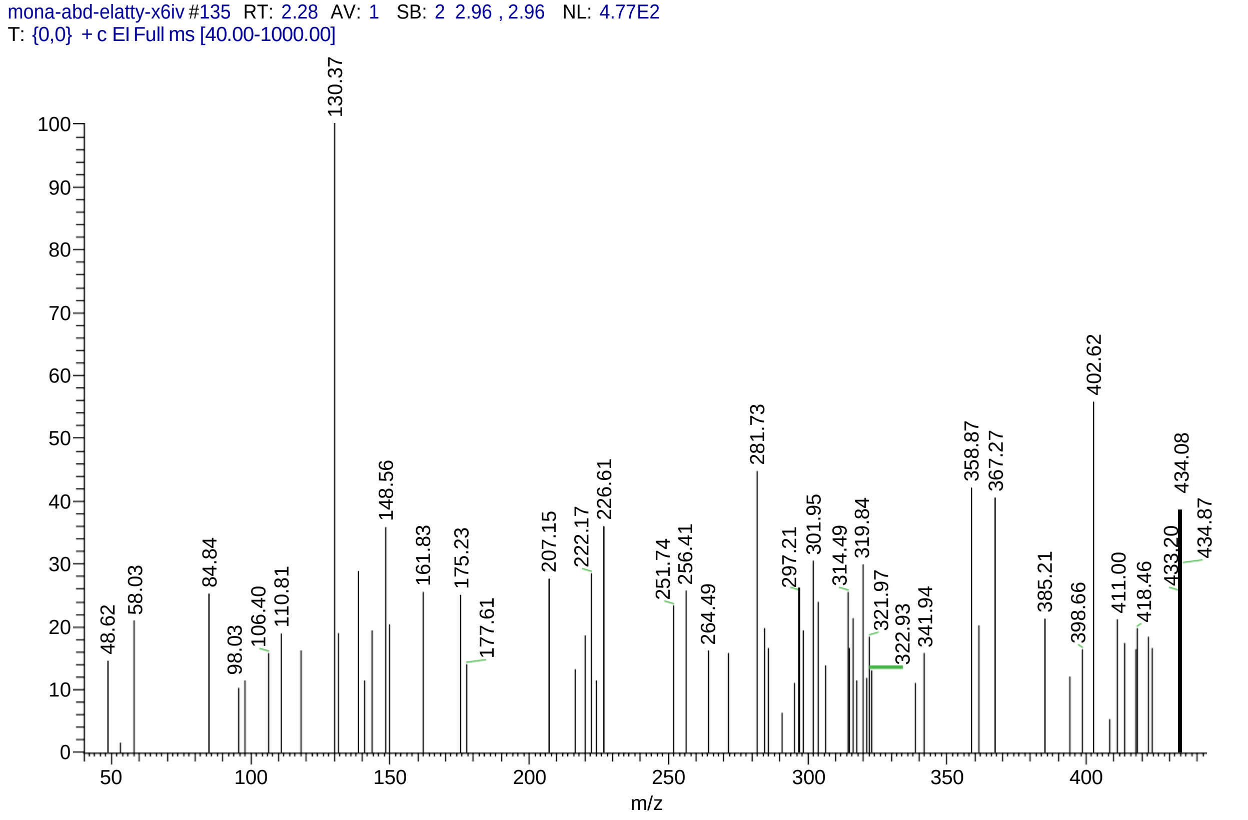

m/z	Intensity	Relative
48.62	69.6	14.59
53.01	6.8	1.42
58.03	99.9	20.93
84.84	120.4	25.23
95.71	48.9	10.25
98.03	54.7	11.46
106.40	74.9	15.70
110.81	90.3	18.92
118.08	76.8	16.09
130.37	477.2	100.00
131.54	90.1	18.89
138.45	138.0	28.92
141.08	54.1	11.34
143.55	92.0	19.28
148.56	170.7	35.76
149.81	97.5	20.42
161.83	121.5	25.45
175.23	119.3	25.01
177.61	66.9	14.03
207.15	131.9	27.63
216.45	62.9	13.19
220.01	88.8	18.61
222.17	135.6	28.42

224.35	54.5	11.43
226.61	172.0	36.04
251.74	111.1	23.27
256.41	122.7	25.71
264.49	77.1	16.15
271.46	75.2	15.76
281.73	213.9	44.82
284.34	94.0	19.70
286.08	79.1	16.57
290.61	30.1	6.31
295.34	52.1	10.92
297.21	121.9	25.54
298.61	92.3	19.34
301.95	145.6	30.51
303.94	114.1	23.92
306.40	65.7	13.77
314.49	121.6	25.48
315.15	78.7	16.49
316.41	101.9	21.35
317.61	54.5	11.43
319.84	142.1	29.78
321.33	55.9	11.71
321.97	87.3	18.30
322.93	62.0	12.99
338.83	52.3	10.95

341.94	75.5	15.81
358.87	201.6	42.25
361.42	96.3	20.17
367.27	193.2	40.49
385.21	101.7	21.32
394.06	57.3	12.01
398.66	78.0	16.35
402.62	266.1	55.77
408.44	25.3	5.31
411.00	101.1	21.18
413.73	82.7	17.32
417.86	78.0	16.35
418.46	94.1	19.73
422.26	87.6	18.36
423.59	78.8	16.51
433.20	121.3	25.43
434.08	192.9	40.43
434.87	142.4	29.84
435.53	106.3	22.27

C:\Xcalibur\...\S\mona-abd-elatty-x5111d

R MYCOLOGY AND BIOTECHNOLOGY

RT: 0.00 - 4.47 SM: 15G

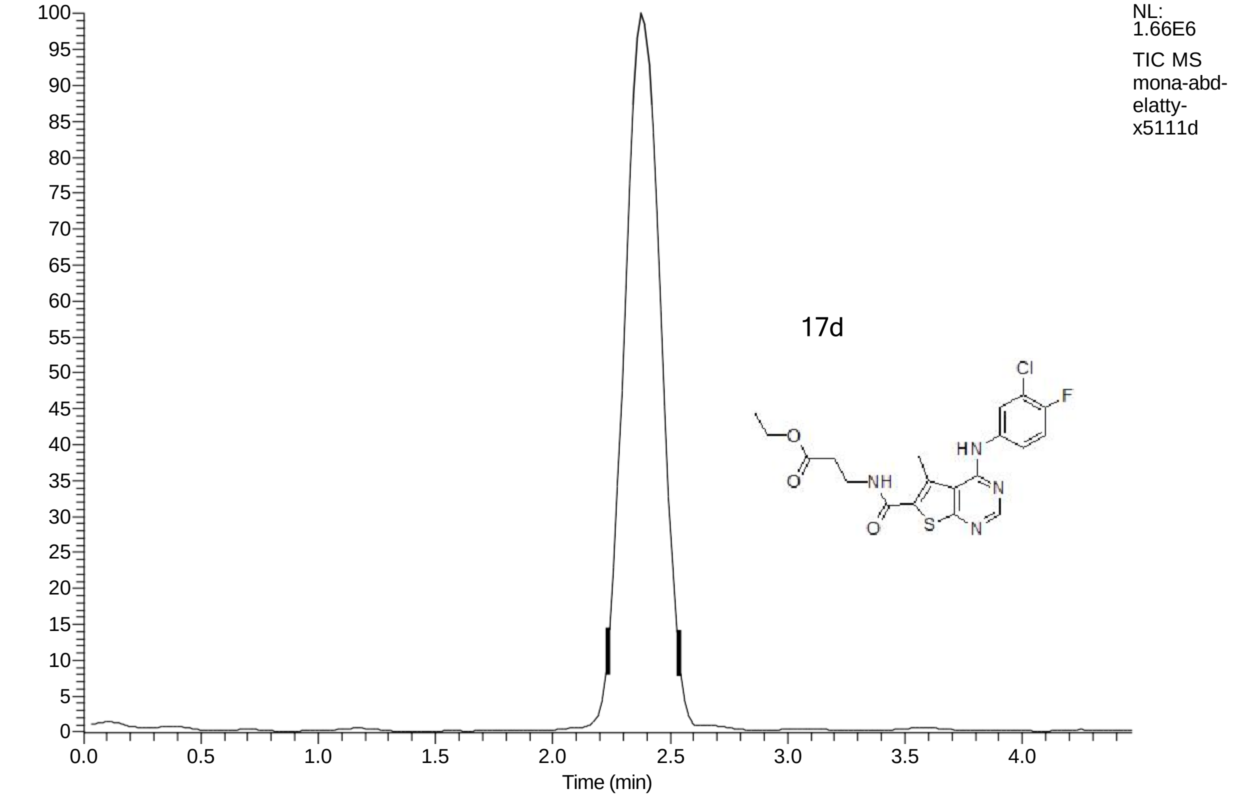

mona-abd-elatty-x5111d #144 RT: 2.43 AV: 1 SB: 2 4.45 , 4.45 NL: 1.06E5
T: {0,0} + c EI Full ms [40.00-1000.00]

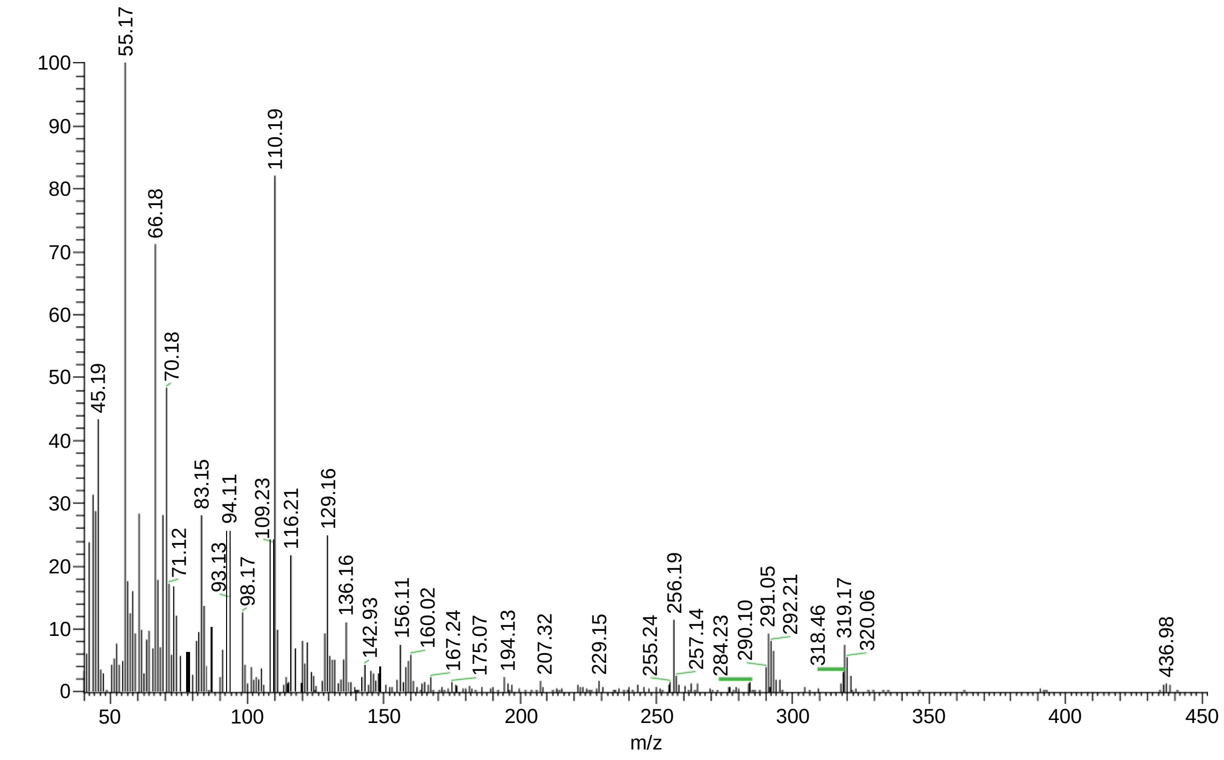

m/z	Intensity	Relative
40.29	3533.2	3.32
41.25	6344.0	5.96
42.20	25240.9	23.71
43.26	33240.3	31.23
44.23	30485.2	28.64
45.19	46109.0	43.32
46.22	3782.2	3.55
47.22	3101.9	2.91
50.19	4489.4	4.22
51.20	5667.4	5.32
52.18	8222.3	7.72
53.15	4635.7	4.35
54.21	5162.6	4.85
55.17	106447.8	100.00
56.12	18653.3	17.52
57.21	13134.6	12.34
58.16	17003.8	15.97
59.15	9906.8	9.31
60.17	30123.5	28.30
61.18	10556.1	9.92
62.26	3047.5	2.86
63.21	8766.6	8.24
64.18	10270.5	9.65

65.21	7354.3	6.91
66.18	75678.5	71.09
67.18	18830.1	17.69
68.32	7421.8	6.97
69.21	29949.0	28.13
70.18	51417.8	48.30
71.12	18183.6	17.08
72.17	6231.1	5.85
73.20	17744.7	16.67
74.20	12768.0	11.99
75.23	5946.4	5.59
76.13	5988.9	5.63
77.16	6101.2	5.73
78.17	12515.3	11.76
79.13	3602.3	3.38
80.05	2812.7	2.64
81.19	8569.7	8.05
82.17	10047.7	9.44
83.15	29816.2	28.01
84.17	14480.1	13.60
85.17	4374.6	4.11
86.27	2153.5	2.02
87.16	5164.5	4.85
88.15	10264.3	9.64
89.17	2053.1	1.93

90.15	2418.4	2.27
91.14	7138.7	6.71
92.11	2460.5	2.31
93.13	15666.0	14.72
94.11	27566.5	25.90
95.16	23386.3	21.97
96.17	8435.4	7.92
97.25	5378.7	5.05
98.17	13456.7	12.64
99.26	4447.6	4.18
100.16	1366.8	1.28
101.30	4075.7	3.83
102.24	1907.9	1.79
103.36	2398.0	2.25
104.22	1979.9	1.86
105.20	3950.1	3.71
107.19	7571.4	7.11
108.20	9315.7	8.75
109.23	25127.8	23.61
110.19	87412.1	82.12
111.14	10400.4	9.77
112.22	5909.1	5.55
114.12	2515.0	2.36
114.78	1310.0	1.23
115.34	1559.2	1.46

116.21	23095.7	21.70
117.27	7234.1	6.80
118.31	4613.6	4.33
119.11	1877.9	1.76
120.20	8597.7	8.08
121.18	4695.8	4.41
122.18	8444.0	7.93
123.26	3251.5	3.05
124.18	2724.6	2.56
127.53	1813.9	1.70
128.53	9867.8	9.27
129.16	26419.7	24.82
130.30	5981.7	5.62
131.18	5419.4	5.09
132.10	5409.2	5.08
133.47	1401.1	1.32
134.31	2011.6	1.89
135.15	5441.9	5.11
136.16	11618.7	10.91
137.07	1609.4	1.51
138.08	1657.2	1.56
142.23	2385.6	2.24
142.93	4491.4	4.22
145.17	3524.7	3.31
146.13	3150.6	2.96

147.18	1762.4	1.66
148.23	3004.9	2.82
149.12	4291.9	4.03
149.92	3569.5	3.35
154.91	1937.5	1.82
156.11	7823.1	7.35
157.15	1585.2	1.49
158.16	4167.5	3.92
159.19	5191.8	4.88
160.02	6135.6	5.76
160.83	1895.6	1.78
164.13	1318.2	1.24
165.10	1616.8	1.52
167.24	2396.9	2.25
175.07	1687.4	1.59
194.13	2400.1	2.25
195.55	1326.6	1.25
207.32	1746.4	1.64
229.15	1781.5	1.67
255.24	1490.4	1.40
256.19	12191.9	11.45
257.14	2658.8	2.50
265.14	1333.5	1.25
283.72	1321.5	1.24
284.23	1497.5	1.41

290.10	4061.7	3.82
291.05	9903.1	9.30
292.21	8468.5	7.96
293.19	6863.4	6.45
294.05	2062.0	1.94
295.21	2005.1	1.88
317.48	1473.2	1.38
318.46	3332.7	3.13
319.17	7917.7	7.44
320.06	5762.2	5.41
321.24	2586.5	2.43
436.98	1439.1	1.35

C:\Xcalibur\data\S\MONA-ABD-ELATTY-X7-IV

R MYCOLOGY AND BIOTECHNOLOGY

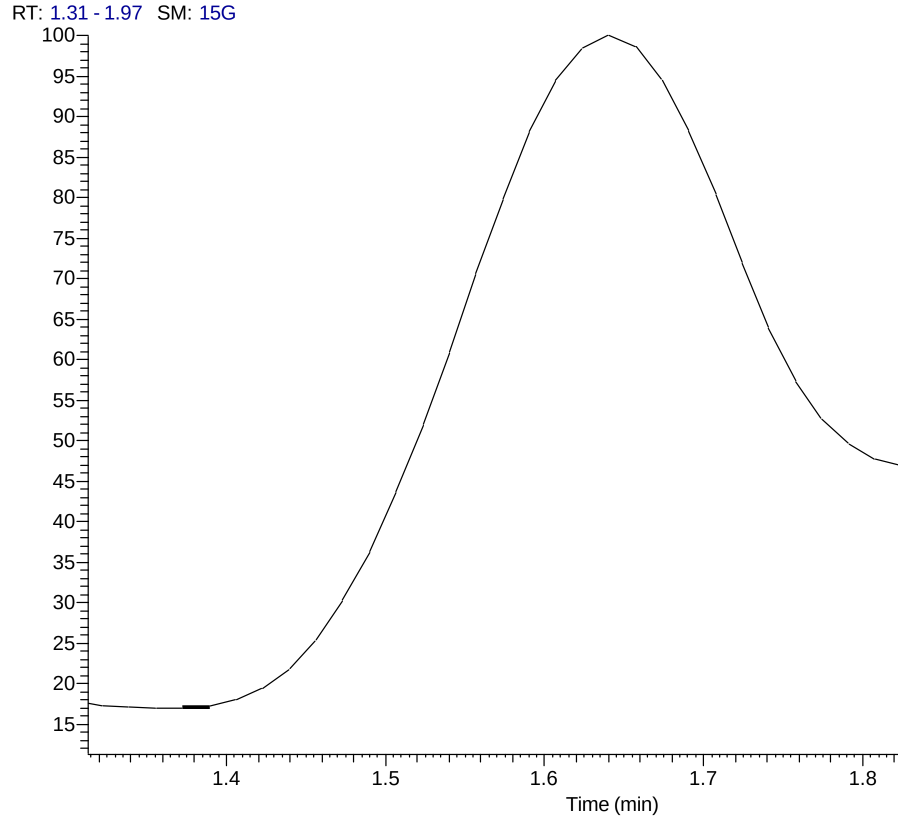

Compound 7b (M.wt = 464)

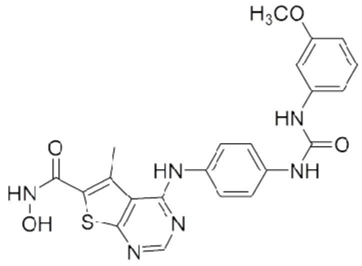

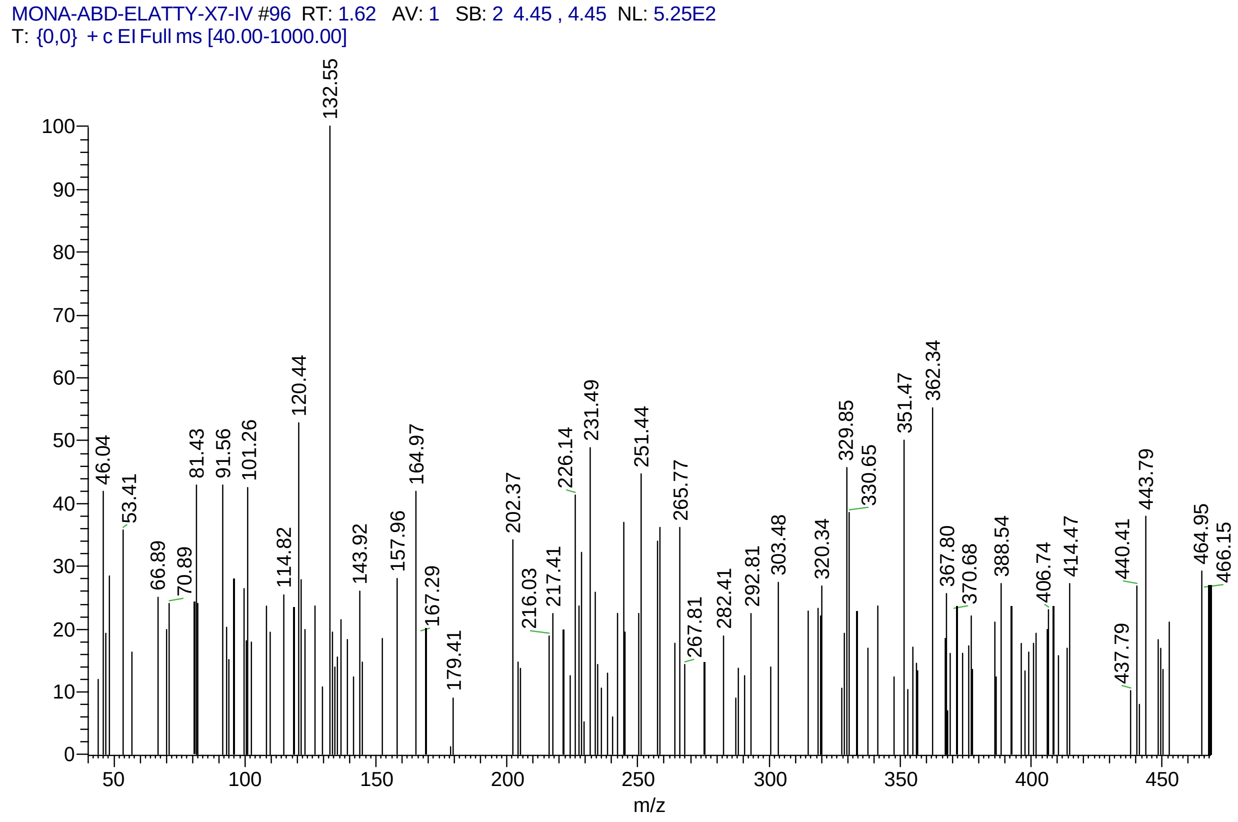

m/z	Intensity	Relative
44.15	63.3	12.07
46.04	220.0	41.92
46.66	102.0	19.44
48.37	149.9	28.56
53.41	188.1	35.85
56.82	85.9	16.36
66.89	131.7	25.10
70.10	104.3	19.87
70.89	126.0	24.01
81.43	225.3	42.94
82.10	126.5	24.11
84.77	123.5	23.53
91.56	225.7	43.01
93.15	107.1	20.40
93.88	80.1	15.27
94.82	143.1	27.26
99.57	139.2	26.52
100.51	95.2	18.14
101.26	223.7	42.63
102.28	94.5	18.01
108.09	124.3	23.68
109.63	102.1	19.46
114.82	133.6	25.46

117.37	121.6	23.17
118.50	119.5	22.76
119.04	116.3	22.15
120.44	277.4	52.87
121.30	146.1	27.85
122.81	105.1	20.02
126.56	124.3	23.68
129.47	57.2	10.90
132.55	524.8	100.00
133.41	102.4	19.51
134.08	73.6	14.02
135.21	81.6	15.55
136.51	112.9	21.52
139.22	96.5	18.39
141.45	65.1	12.40
143.92	136.5	26.02
144.60	77.7	14.81
152.17	97.2	18.52
157.96	146.9	28.00
164.97	219.7	41.87
167.29	101.6	19.36
178.40	6.9	1.32
179.41	47.2	8.99
202.37	179.7	34.25
204.39	77.5	14.76

204.90	72.0	13.72
216.03	99.3	18.93
217.41	118.3	22.54
223.02	100.7	19.18
224.27	66.1	12.60
226.14	217.3	41.41
227.35	124.1	23.65
228.57	168.8	32.16
229.52	27.7	5.28
231.49	256.5	48.88
233.64	136.0	25.91
234.48	76.0	14.48
235.96	55.3	10.54
238.21	68.0	12.96
240.34	31.3	5.97
241.96	117.7	22.43
244.34	193.9	36.94
245.04	102.3	19.49
250.28	118.1	22.51
251.44	234.7	44.72
257.18	178.3	33.97
258.50	190.0	36.20
264.26	93.3	17.78
265.77	190.4	36.28
267.81	75.6	14.41

275.53	73.1	13.92
282.41	99.7	19.00
287.40	47.7	9.10
288.06	72.7	13.85
290.60	65.6	12.50
292.81	118.5	22.59
300.43	73.5	14.00
303.48	144.7	27.57
314.81	120.4	22.94
318.61	122.7	23.37
319.74	116.0	22.10
320.34	141.3	26.93
327.66	55.9	10.65
328.47	101.5	19.33
329.85	240.4	45.81
330.65	202.8	38.64
333.00	116.3	22.15
337.79	89.1	16.97
341.64	124.0	23.63
347.73	65.5	12.47
351.47	263.2	50.15
352.66	54.8	10.44
354.86	90.3	17.20
356.01	76.9	14.66
356.53	70.3	13.39

362.34	290.1	55.28
367.01	97.9	18.65
367.80	135.3	25.79
368.32	37.1	7.06
368.87	85.1	16.21
370.68	120.3	22.92
373.76	84.9	16.18
376.36	91.6	17.45
377.28	116.4	22.18
377.79	71.3	13.59
386.07	110.7	21.09
386.73	64.8	12.35
388.54	142.9	27.24
392.61	120.7	22.99
396.33	93.5	17.81
397.79	70.8	13.49
399.06	85.9	16.36
400.73	93.6	17.84
401.80	101.1	19.26
406.00	104.5	19.92
406.74	120.8	23.02
408.74	120.4	22.94
410.46	82.5	15.73
413.65	88.8	16.92
414.47	143.2	27.29

437.79	54.1	10.32
440.41	141.2	26.91
441.13	42.7	8.13
443.79	199.1	37.93
448.48	96.4	18.37
449.53	88.8	16.92
450.12	71.6	13.64
452.60	110.9	21.14
464.95	153.5	29.24
466.15	138.3	26.35
466.86	132.3	25.20

C:\Xcalibur\...\S\mona-abd-elatty-x6111e

R MYCOLOGY AND BIOTECHNOLOGY

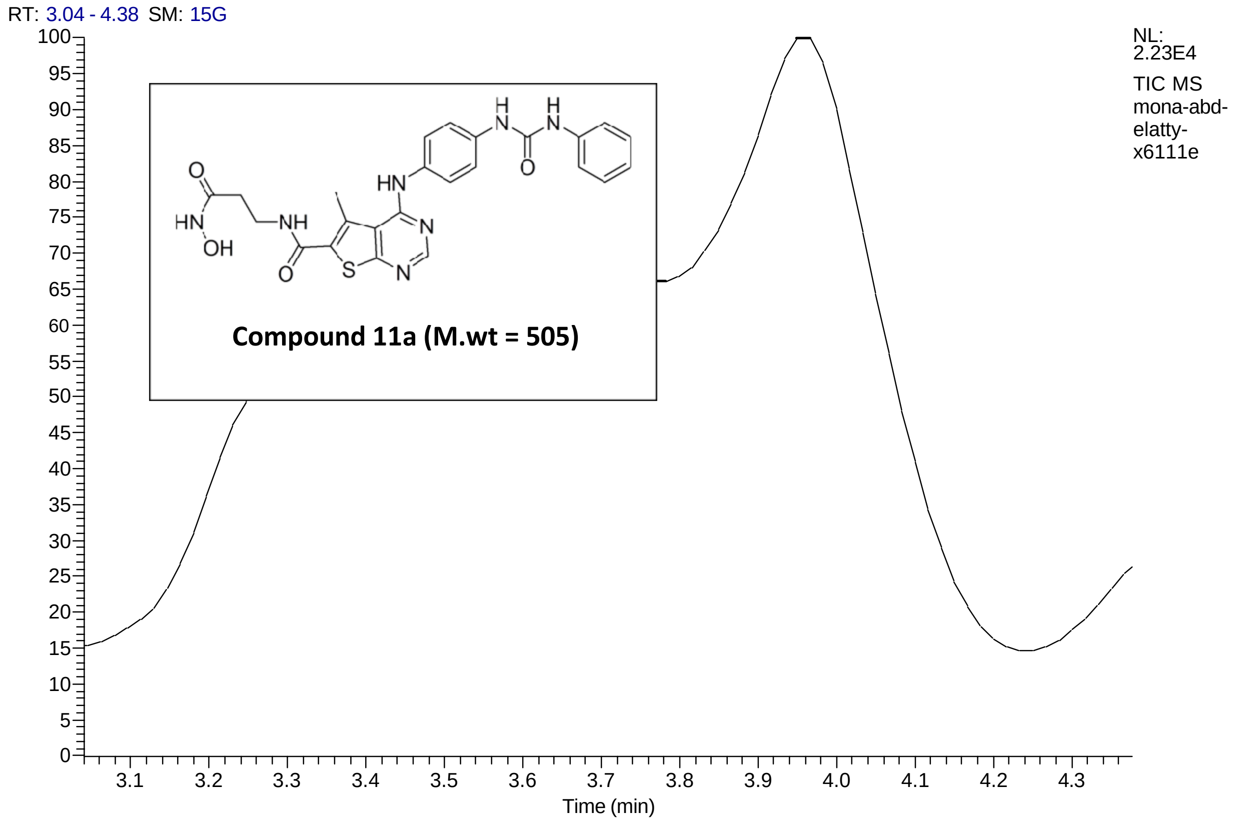

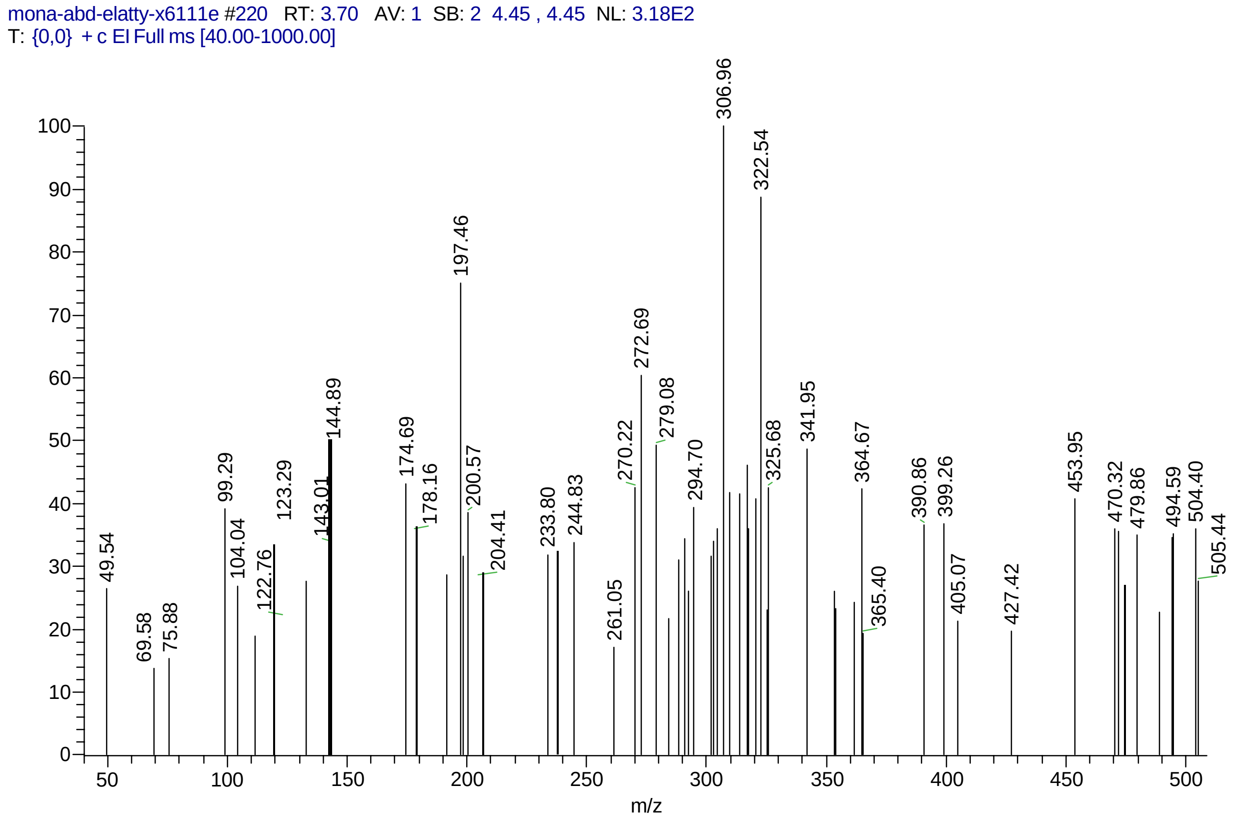

m/z	Intensity	Relative
49.54	84.1	26.46
69.58	44.1	13.88
75.88	49.2	15.47
99.29	124.7	39.20
104.04	85.5	26.88
111.38	60.3	18.95
122.76	69.6	21.89
123.29	115.2	36.23
132.95	87.9	27.63
142.36	92.1	28.97
143.01	106.7	33.54
144.89	160.1	50.36
174.69	137.2	43.14
178.16	113.2	35.60
191.68	91.2	28.68
197.46	238.7	75.05
198.21	100.8	31.70
200.57	122.7	38.57
204.41	89.9	28.26
233.80	101.3	31.87
236.36	88.4	27.80
237.01	100.9	31.74
244.83	107.7	33.88

261.05	54.8	17.23
270.22	135.3	42.56
272.69	192.3	60.46
279.08	156.8	49.31
284.08	68.8	21.64
288.14	98.7	31.03
290.96	109.3	34.38
292.37	83.1	26.12
294.70	124.9	39.29
301.67	100.8	31.70
302.93	108.3	34.05
304.21	114.8	36.10
306.96	318.0	100.00
309.55	132.7	41.72
313.69	132.0	41.51
316.84	146.4	46.04
317.54	114.5	36.02
320.42	129.5	40.71
322.54	282.2	88.76
325.07	73.5	23.10
325.68	135.6	42.64
341.95	154.9	48.72
353.14	82.7	26.00
353.67	74.4	23.40
361.77	77.5	24.36

364.67	134.9	42.43
365.40	61.5	19.33
390.86	116.3	36.56
399.26	117.1	36.81
405.07	67.6	21.26
427.42	62.9	19.79
453.95	129.6	40.75
470.32	114.7	36.06
471.94	113.1	35.56
473.52	83.7	26.33
476.01	91.6	28.81
477.66	95.2	29.94
479.86	111.6	35.09
488.90	72.5	22.81
494.06	110.1	34.63
494.59	111.7	35.14
504.40	114.8	36.10
505.44	88.3	27.76

C:\Xcalibur\data\S\mona-abd-elatty-x111b

R MYCOLOGY AND BIOTECHNOLOGY

RT: 0.00 - 4.55 SM: 15G

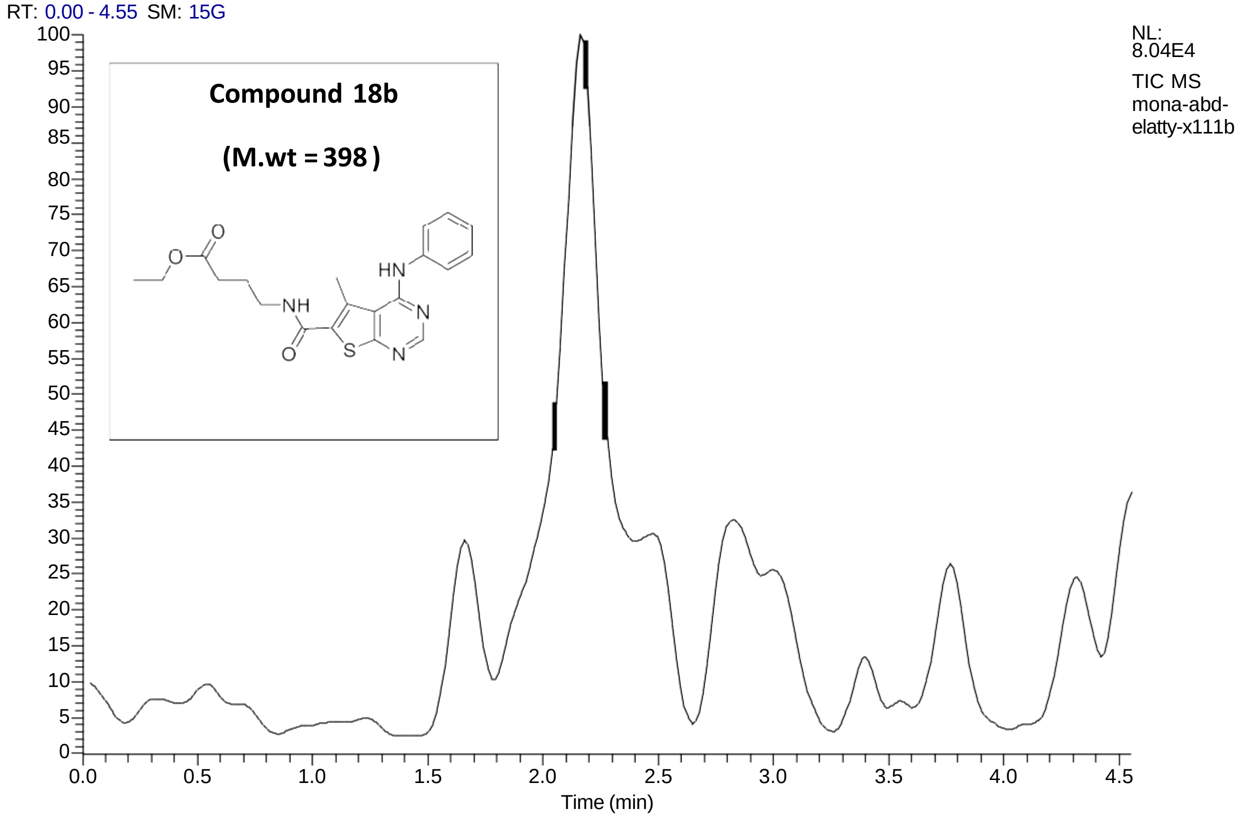

mona-abd-elatty-x111b #126 RT: 2.13 AV: 1 SB: 2 4.45 , 4.45 NL: 6.52E3
T: {0,0} + c EI Full ms [40.00-1000.00]

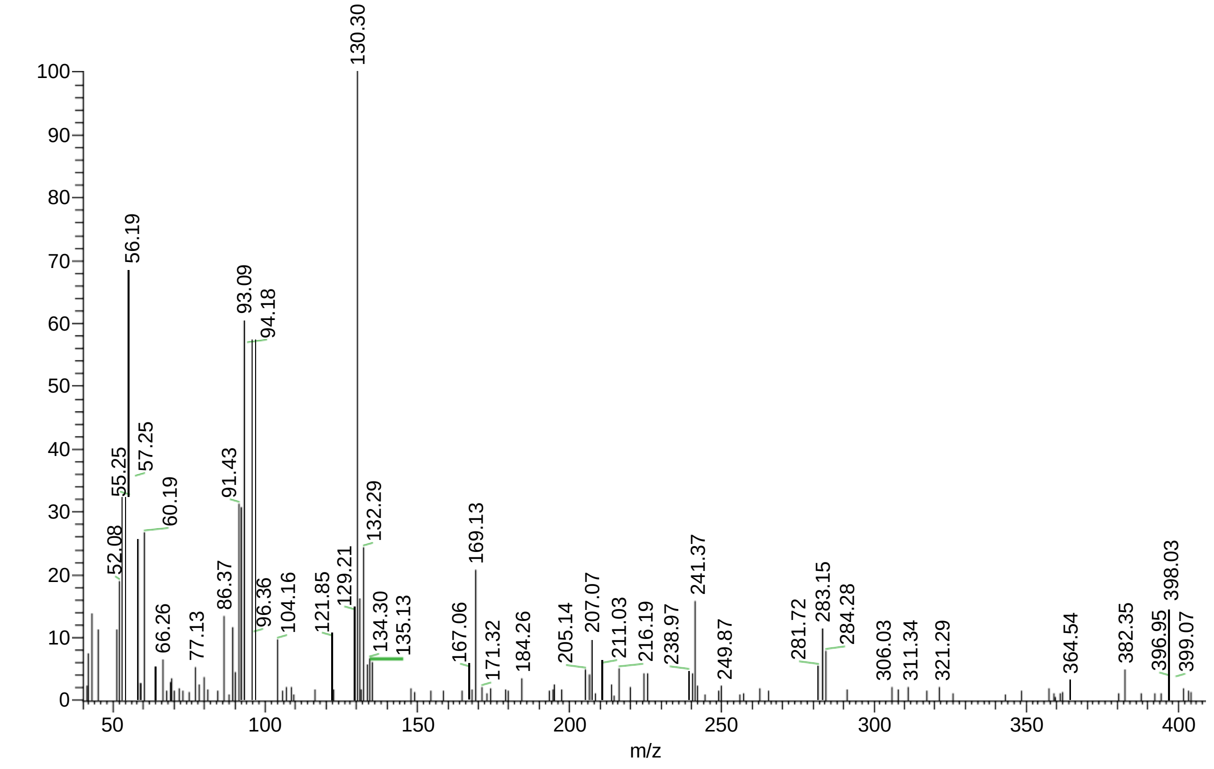

m/z	Intensity	Relative
41.44	152.0	2.33
42.01	483.8	7.42
43.12	903.3	13.84
45.08	728.0	11.16
51.30	732.4	11.22
52.08	1240.6	19.01
53.26	871.0	13.35
54.19	689.0	10.56
55.25	2116.4	32.44
56.19	4478.6	68.64
57.25	2307.0	35.36
58.25	1677.5	25.71
58.88	171.1	2.62
60.19	1736.8	26.62
65.29	306.1	4.69
66.26	418.2	6.41
67.63	96.7	1.48
68.85	187.1	2.87
69.38	220.5	3.38
69.96	95.2	1.46
71.76	117.6	1.80
72.86	100.7	1.54
74.85	84.0	1.29

77.13	347.2	5.32	
78.16	160.9	2.47	
79.76	233.5	3.58	
81.16	116.4	1.78	
84.37	95.2	1.46	
86.37	873.1	13.38	
89.35	755.7	11.58	
90.17	289.3	4.43	
91.43	2033.9		31.17
92.32	2002.3		30.69
93.09	3938.8		60.37
94.18	3693.1		56.60
95.11	429.8	6.59	
96.36	693.3	10.63	
97.04	277.6	4.25	
98.20	316.2	4.85	
99.19	115.2	1.77	
104.16	632.4	9.69	
105.76	97.6	1.50	
107.02	136.4	2.09	
108.78	141.3	2.17	
116.38	106.1	1.63	
121.85	659.6	10.11	
122.41	103.9	1.59	
128.42	421.4	6.46	

129.21	928.5	14.23	
130.30	6524.6		100.00
131.12	1056.9		16.20
131.64	115.5	1.77	
132.29	1590.8		24.38
133.74	375.3	5.75	
134.30	431.7	6.62	
135.13	397.3	6.09	
147.75	116.5	1.79	
149.31	89.9	1.38	
154.59	93.6	1.43	
158.42	92.1	1.41	
164.48	93.3	1.43	
167.06	331.0	5.07	
167.74	105.6	1.62	
169.13	1351.0		20.71
171.32	139.2	2.13	
174.10	116.7	1.79	
179.08	107.6	1.65	
180.01	94.1	1.44	
184.26	224.0	3.43	
193.15	97.2	1.49	
194.47	112.7	1.73	
194.99	157.6	2.42	
197.41	114.5	1.76	

205.14	313.7	4.81	
206.30	265.8	4.07	
207.07	624.1	9.57	
211.03	370.1	5.67	
213.88	161.2	2.47	
216.19	324.1	4.97	
219.75	142.4	2.18	
224.63	280.9	4.31	
225.85	274.5	4.21	
238.97	307.3	4.71	
240.31	282.0	4.32	
241.37	1031.7		15.81
242.02	142.9	2.19	
249.11	103.2	1.58	
249.87	151.5	2.32	
262.42	120.0	1.84	
265.22	95.3	1.46	
281.72	356.6	5.47	
283.15	746.4	11.44	
284.28	511.4	7.84	
291.07	112.7	1.73	
306.03	136.3	2.09	
308.00	104.5	1.60	
311.34	138.3	2.12	
317.20	90.8	1.39	

321.29	139.7	2.14
348.35	100.0	1.53
357.57	124.0	1.90
362.17	80.3	1.23
364.54	209.3	3.21
382.35	312.2	4.79
396.95	240.7	3.69
398.03	979.8	15.02
399.07	227.5	3.49

C:\Xcalibur\data\S\mona-abd-elatty-x8iv

R MYCOLOGY AND BIOTECHNOLOGY

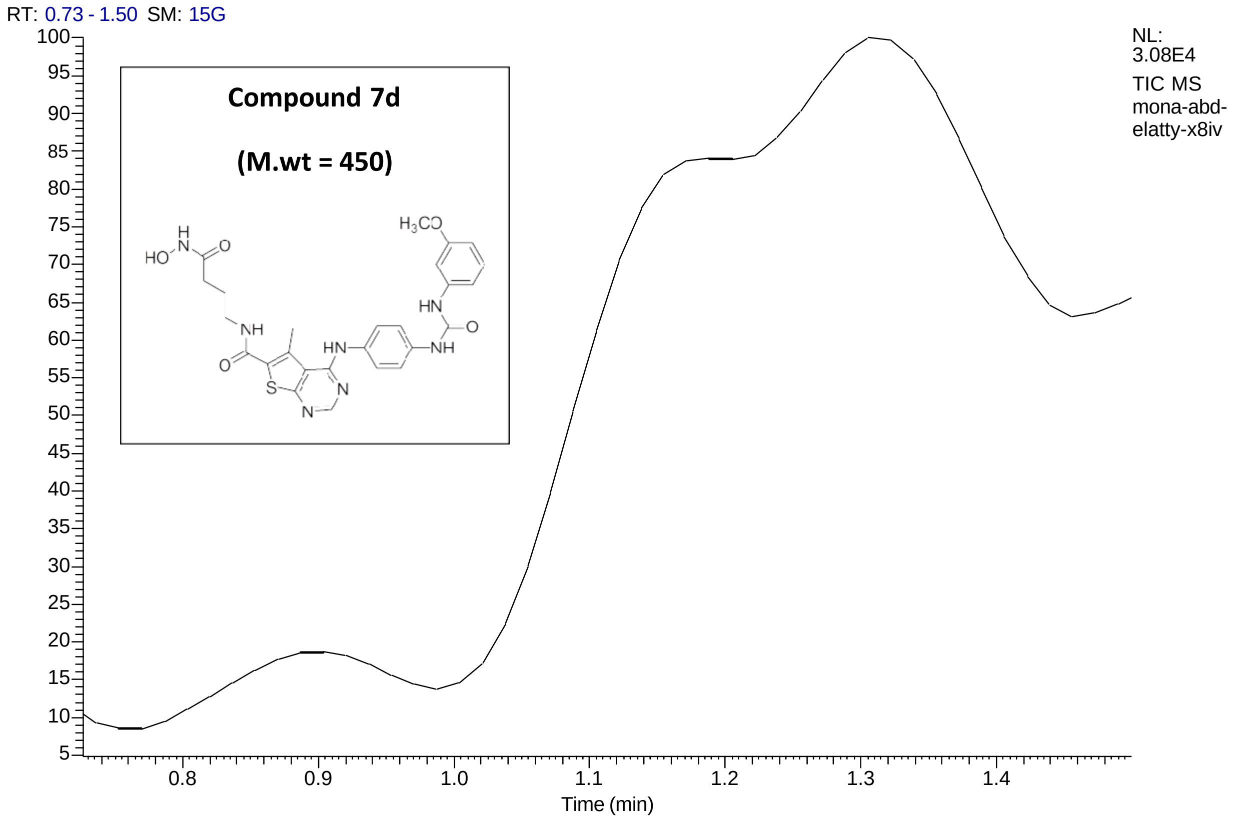

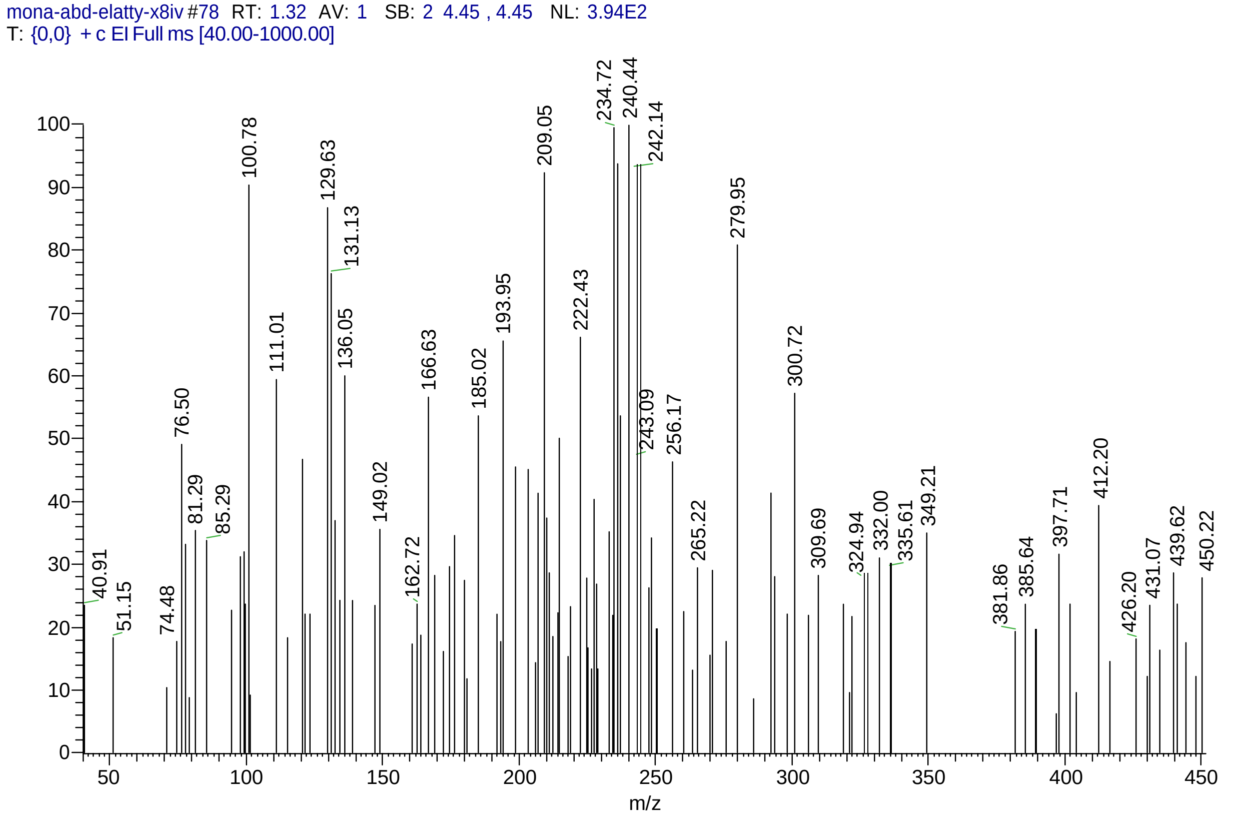

m/z	Intensity	Relative
40.91	92.8	23.53
51.15	72.1	18.29
70.62	41.5	10.51
74.48	70.3	17.82
76.50	193.9	49.15
77.88	131.3	33.30
79.01	35.2	8.92
81.29	139.9	35.46
85.29	133.3	33.81
94.42	89.7	22.75
97.72	123.6	31.34
99.15	126.0	31.95
99.77	93.5	23.70
100.78	356.5	90.40
101.41	36.7	9.30
111.01	234.7	59.50
115.16	72.4	18.36
120.75	184.7	46.82
121.60	87.1	22.08
123.41	86.9	22.04
129.63	342.5	86.85
131.13	300.6	76.23
132.68	145.6	36.92

134.36	95.7	24.27
136.05	237.1	60.11
139.03	95.7	24.27
147.29	92.8	23.53
149.02	140.7	35.67
160.74	68.8	17.44
162.72	93.7	23.77
163.86	74.1	18.80
166.63	223.5	56.66
169.10	111.9	28.36
172.41	63.9	16.19
174.55	116.7	29.58
176.33	136.4	34.58
180.08	108.5	27.52
180.68	46.8	11.87
185.02	211.5	53.62
191.68	87.2	22.11
193.40	70.0	17.75
193.95	258.4	65.52
198.47	179.9	45.61
203.09	178.1	45.17
205.92	57.1	14.47
206.99	162.9	41.31
209.05	364.2	92.36
210.12	147.7	37.46

210.81	112.9	28.63
212.29	72.8	18.46
214.00	87.9	22.28
214.85	197.9	50.17
217.65	60.7	15.38
218.94	91.9	23.29
222.43	261.0	66.19
224.68	109.6	27.79
225.37	66.0	16.73
226.54	52.9	13.42
227.22	159.6	40.47
228.28	106.3	26.94
228.87	53.1	13.46
233.02	138.8	35.19
234.08	86.3	21.87
234.72	392.2	99.46
236.09	369.7	93.75
237.11	211.3	53.58
240.44	394.4	100.00
242.14	366.6	92.97
243.09	185.7	47.09
244.22	114.1	28.94
247.64	104.0	26.37
248.62	134.9	34.21
252.78	75.2	19.07

256.17	182.8	46.35
260.16	89.1	22.58
263.59	52.1	13.22
265.22	116.5	29.55
269.81	61.3	15.55
270.95	114.8	29.11
275.67	70.1	17.78
279.95	318.8	80.83
285.99	34.1	8.65
292.47	163.5	41.45
293.58	110.4	27.99
298.27	87.5	22.18
300.72	225.6	57.20
305.94	86.7	21.97
309.69	111.6	28.30
318.61	93.3	23.66
321.00	37.9	9.60
322.12	85.5	21.67
324.40	64.3	16.29
324.94	109.9	27.86
327.99	33.6	8.52
329.11	96.0	24.34
332.00	122.8	31.14
335.61	116.4	29.51
349.21	138.3	35.06

381.86	76.5	19.41
385.64	93.7	23.77
386.84	74.4	18.86
397.00	24.3	6.15
397.71	124.9	31.68
401.67	93.9	23.80
404.26	38.3	9.70
412.20	155.6	39.45
416.46	57.6	14.60
426.20	71.3	18.09
430.00	48.1	12.20
431.07	92.5	23.46
434.60	64.9	16.46
439.62	113.3	28.74
440.93	93.2	23.63
444.18	69.6	17.65
448.06	48.4	12.27
450.22	110.1	27.92

C:\Xcalibur\...\S\mona-abd-elatty-x7111c

R MYCOLOGY AND BIOTECHNOLOGY

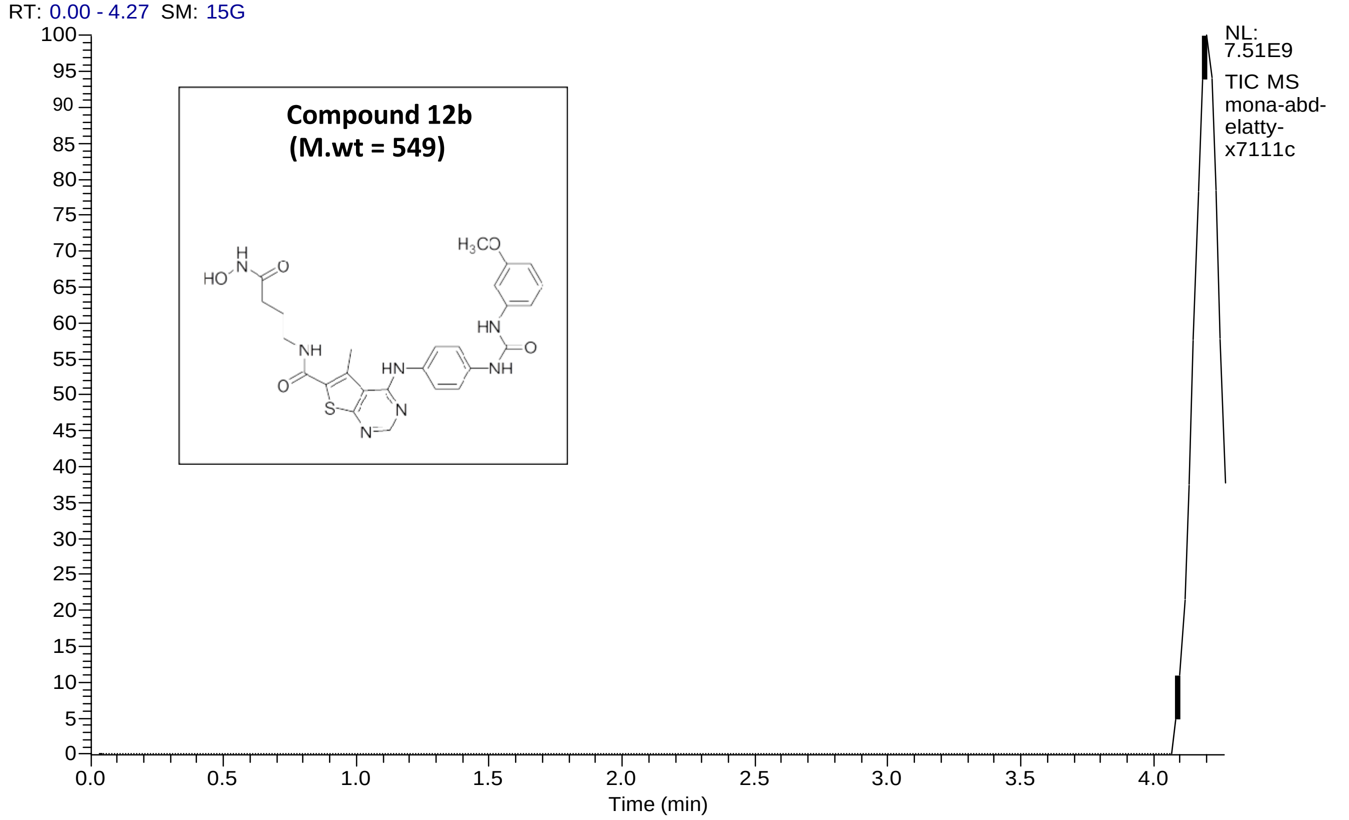

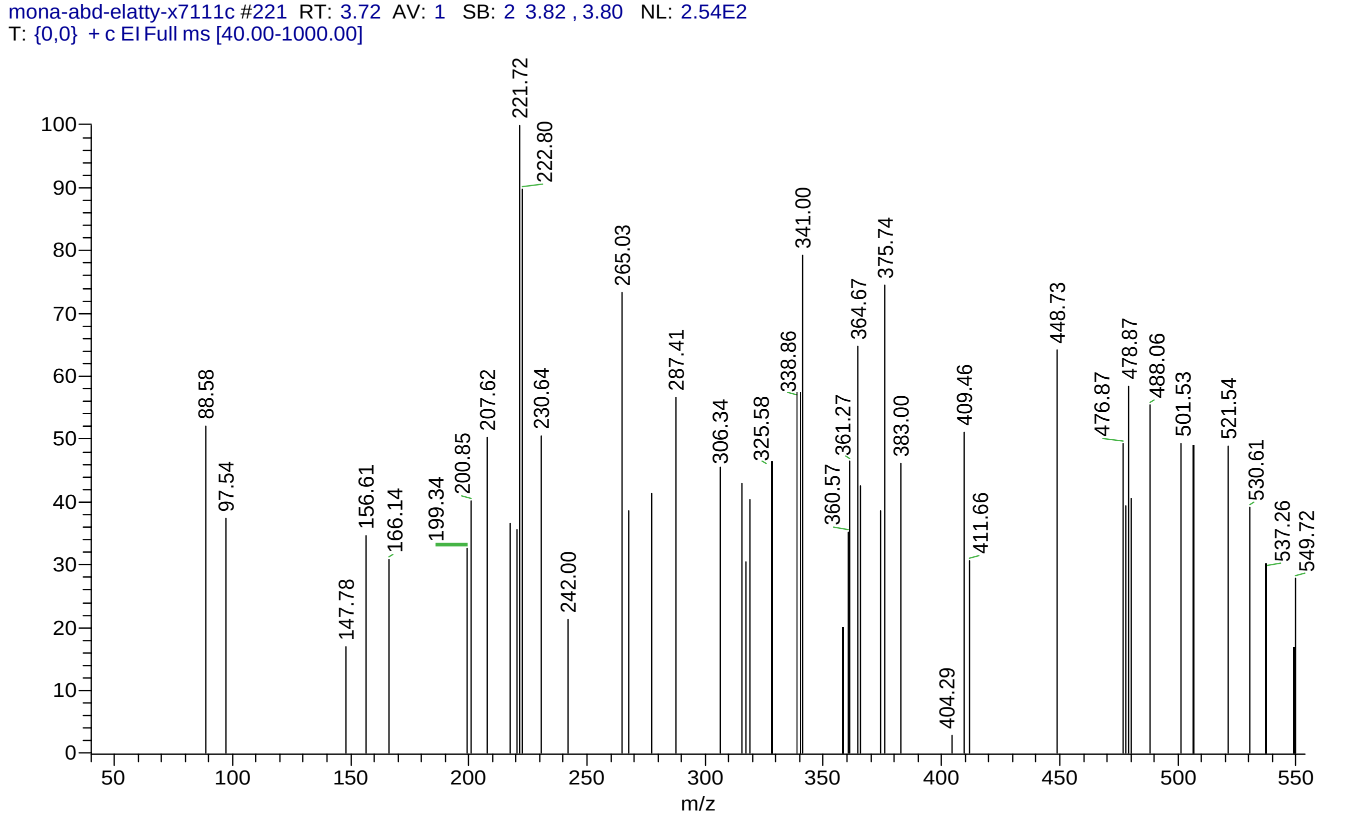

m/z	Intensity	Relative
88.58	132.1	52.08
97.54	94.8	37.36
147.78	43.2	17.03
156.61	87.7	34.58
166.14	78.5	30.95
199.34	82.8	32.63
200.85	101.9	40.15
207.62	127.7	50.34
217.46	92.7	36.52
220.55	90.1	35.52
221.72	253.7	100.00
222.80	227.9	89.81
230.64	128.0	50.45
242.00	54.1	21.33
265.03	186.1	73.36
267.70	98.1	38.68
277.22	105.2	41.46
287.41	143.6	56.59
306.34	115.6	45.56
315.40	109.2	43.04
317.07	77.5	30.53
318.82	102.5	40.41
325.58	116.3	45.82

338.86	143.7	56.65
341.00	200.9	79.19
341.67	88.0	34.68
360.57	89.2	35.16
361.27	118.0	46.51
364.67	164.1	64.69
365.63	108.1	42.62
367.87	49.3	19.42
374.11	98.0	38.62
375.74	189.1	74.51
383.00	117.2	46.19
404.29	7.4	2.92
409.46	129.9	51.18
411.66	77.9	30.69
448.73	162.7	64.11
476.87	125.1	49.29
478.12	100.1	39.46
478.87	148.3	58.43
480.32	102.9	40.57
488.06	140.9	55.54
501.53	125.3	49.40
506.21	122.7	48.34
521.54	124.1	48.92
530.61	99.2	39.10
537.26	74.8	29.48

547.52	41.2	16.24
549.72	70.9	27.96

C:\Xcalibur\data\S\mona-abd-elatty-xiiid

R MYCOLOGY AND BIOTECHNOLOGY

RT: 0.00 - 4.65 SM: 15G

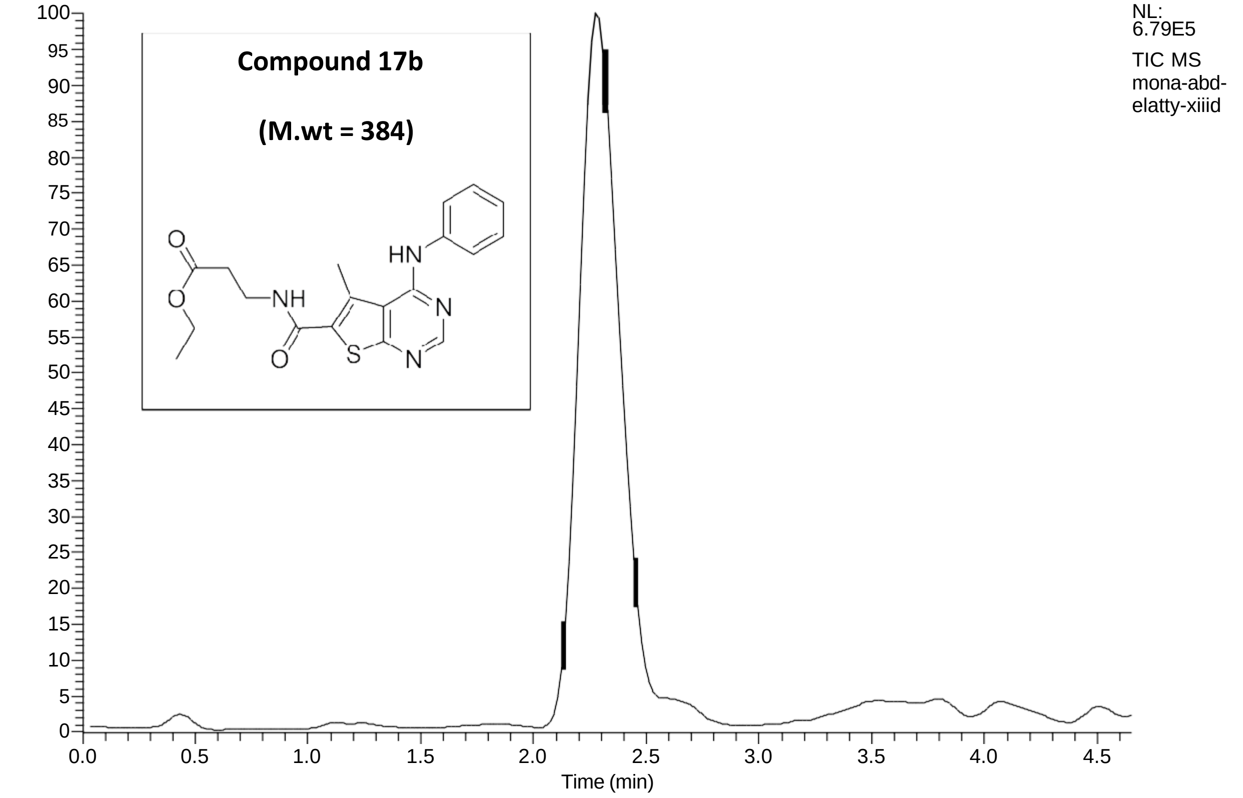

mona-abd-elatty-xiiid #133-138 RT: 2.24-2.33 AV: 6 SB: 2 4.45 , 4.45 NL: 6.07E4
T: {0,0} + c EI Full ms [40.00-1000.00]

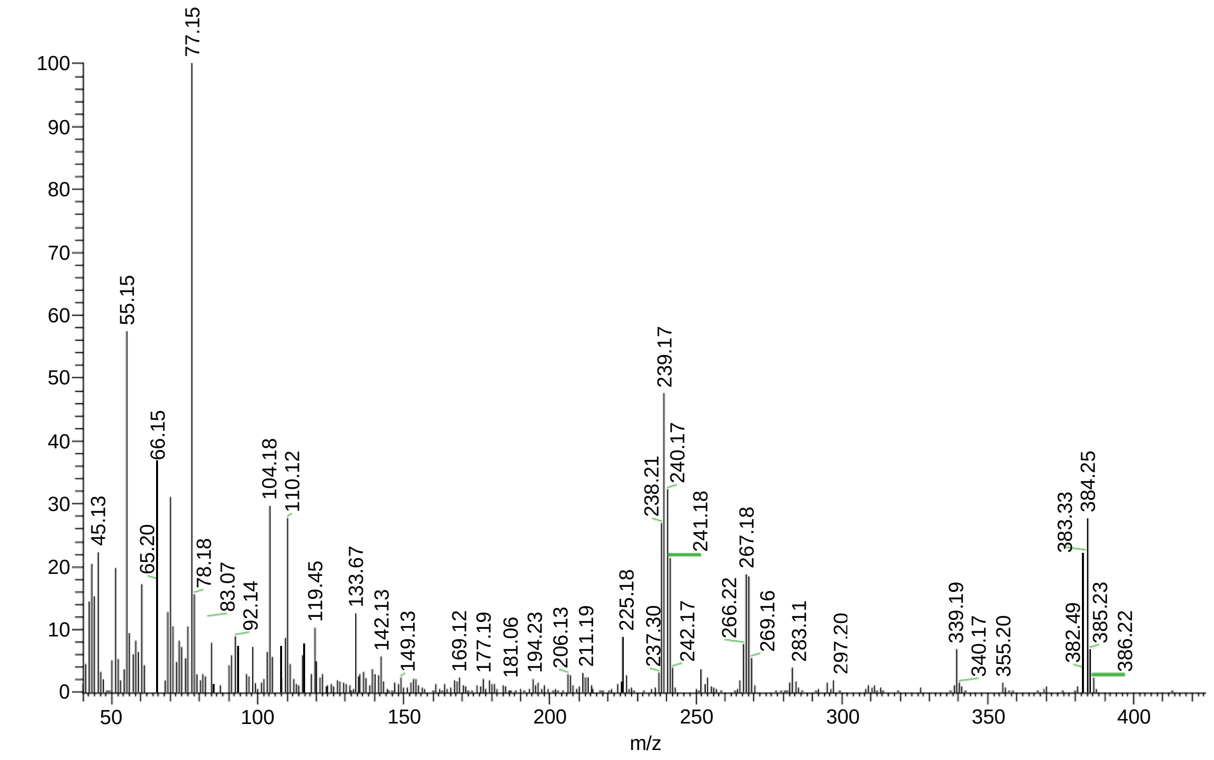

m/z	Intensity	Relative
40.34	829.2	1.37
41.23	2682.9	4.42
42.18	8695.4	14.33
43.15	12317.6	20.30
44.17	9178.8	15.13
45.13	13525.4	22.29
46.13	1928.9	3.18
47.19	1222.7	2.01
50.15	3053.6	5.03
51.17	11969.7	19.72
52.17	3216.7	5.30
53.14	1153.7	1.90
54.26	2225.7	3.67
55.15	34883.3	57.48
56.14	5695.0	9.38
57.20	3644.9	6.01
58.14	5022.4	8.28
59.16	3962.7	6.53
60.13	10421.1	17.17
61.17	2575.6	4.24
62.12	1174.4	1.94
63.15	2861.1	4.71
64.22	3989.2	6.57

65.20	10802.7	17.80
66.15	22494.1	37.07
67.17	5567.9	9.17
68.23	1154.8	1.90
69.14	7788.0	12.83
70.12	18854.6	31.07
71.14	6325.3	10.42
72.28	2928.1	4.83
73.16	5008.6	8.25
74.13	4405.1	7.26
75.22	3286.0	5.41
76.14	6298.0	10.38
77.15	60685.6	100.00
78.18	9501.7	15.66
79.18	1702.6	2.81
80.20	1139.9	1.88
81.24	1771.1	2.92
82.19	1541.3	2.54
83.07	7213.0	11.89
84.16	4799.1	7.91
85.12	2009.3	3.31
86.16	1127.1	1.86
88.22	3495.9	5.76
89.20	2143.2	3.53
90.15	2611.4	4.30

91.19	3579.5		5.90
92.14	5325.1		8.77
93.13	4760.4		7.84
94.10	4257.2		7.02
95.07	4056.5		6.68
96.01	1717.1		2.83
97.18	1524.6		2.51
98.18	4411.8		7.27
99.13	893.9	1.47	
101.14	950.3	1.57	
102.12	1213.4		2.00
103.21	3947.0		6.50
104.18	17992.5		29.65
105.13	3420.4		5.64
106.14	3993.6		6.58
107.14	2058.6		3.39
108.22	1349.5		2.22
109.21	5183.5		8.54
110.12	16825.8		27.73
111.09	2704.7		4.46
112.19	1276.2		2.10
115.15	3557.1		5.86
116.17	4326.0		7.13
117.22	1247.0		2.05
118.36	1776.5		2.93

119.45	6174.4		10.17
120.19	3002.6		4.95
121.16	1441.4		2.38
122.16	1783.4		2.94
125.13	821.4	1.35	
127.13	1180.1		1.94
128.14	1006.6		1.66
129.21	908.7	1.50	
130.27	798.7	1.32	
133.67	7595.3		12.52
134.38	1499.9		2.47
135.15	1716.2		2.83
136.18	2009.3		3.31
137.16	1403.8		2.31
139.13	2266.4		3.73
140.12	1708.4		2.82
141.16	1581.6		2.61
142.13	3399.9		5.60
143.03	997.9	1.64	
147.06	960.4	1.58	
148.14	807.7	1.33	
149.13	1428.2		2.35
152.25	847.0	1.40	
153.22	1215.2		2.00
154.15	1322.3		2.18

161.15	797.2	1.31	
164.11	733.2	1.21	
167.25	1106.0		1.82
168.31	973.5	1.60	
169.12	1370.2		2.26
177.19	1276.0		2.10
179.28	1163.8		1.92
180.12	777.4	1.28	
181.06	836.1	1.38	
194.23	1256.2		2.07
196.18	902.4	1.49	
206.13	1739.1		2.87
207.07	1603.8		2.64
211.19	1818.3		3.00
212.14	1443.2		2.38
213.07	1424.9		2.35
223.22	736.7	1.21	
224.41	968.8	1.60	
225.18	5379.0		8.86
226.09	1664.0		2.74
237.30	1914.1		3.15
238.21	16324.6		26.90
239.17	28784.3		47.43
240.17	19521.2		32.17
241.18	12910.5		21.27

242.17	2376.1		3.92
251.98	2170.1		3.58
253.26	795.1	1.31	
254.21	1381.0		2.28
265.33	1183.3		1.95
266.22	4595.8		7.57
267.18	11389.1		18.77
268.19	11125.7		18.33
269.16	3325.1		5.48
282.34	853.9	1.41	
283.11	2343.1		3.86
284.17	1014.3		1.67
295.14	944.2	1.56	
297.20	1123.0		1.85
339.19	4149.1		6.84
340.17	890.3	1.47	
355.20	850.7	1.40	
382.49	2230.0		3.67
383.33	13507.0		22.26
384.25	16834.2		27.74
385.23	4140.0		6.82
386.22	1326.7		2.19

C:\Xcalibur\...\S\mona-abd-elatty-x2111b

R MYCOLOGY AND BIOTECHNOLOGY

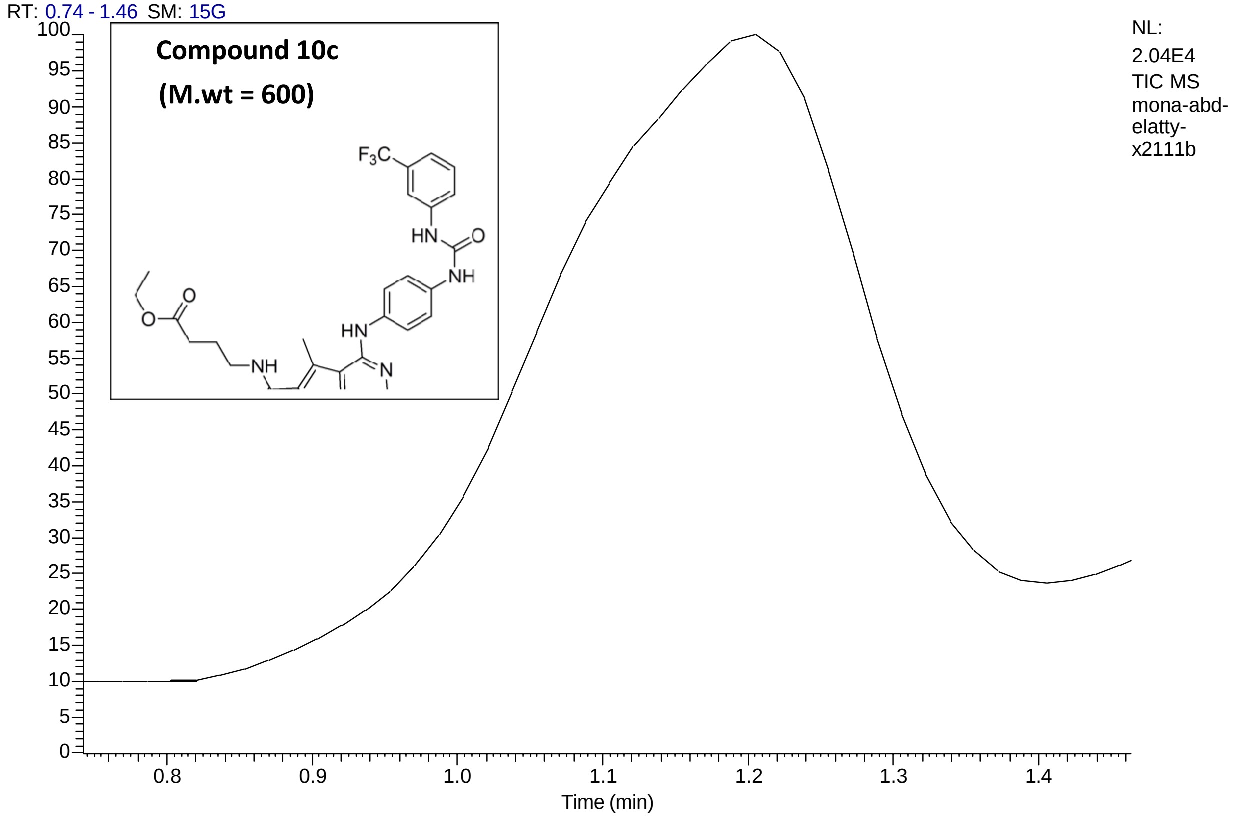

mona-abd-elatty-x2111b #69 RT: 1.17 AV: 1 SB: 2 4.45 , 4.45 NL: 4.39E2
T: {0,0} + c EI Full ms [40.00-1000.00]

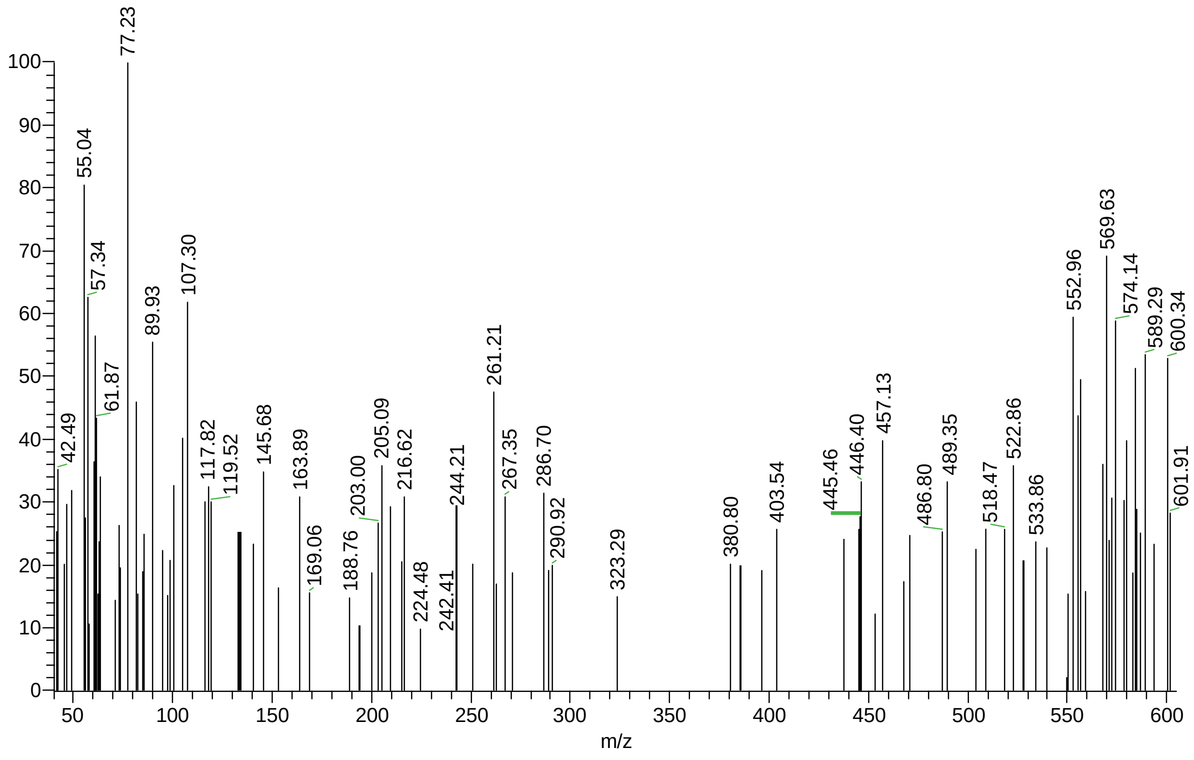

m/z	Intensity	Relative
41.42	111.3	25.36
42.49	154.8	35.26
45.38	88.5	20.16
46.69	130.1	29.64
49.23	139.6	31.79
55.04	352.9	80.38
55.69	120.3	27.39
57.34	274.9	62.62
57.89	46.8	10.66
60.10	160.0	36.44
60.88	248.3	56.54
61.87	190.7	43.43
62.49	67.5	15.37
63.02	103.7	23.63
63.73	149.7	34.10
70.95	63.2	14.39
72.83	115.7	26.36
73.61	86.1	19.62
77.23	439.0	100.00
81.52	201.7	45.95
82.37	67.3	15.34
84.68	83.6	19.04
85.24	108.9	24.81

89.93	243.6	55.48
94.65	98.4	22.41
97.61	66.9	15.24
98.50	91.3	20.80
100.62	143.6	32.71
105.01	176.3	40.15
107.30	271.6	61.86
115.89	132.1	30.09
117.82	142.8	32.52
119.52	132.1	30.09
132.42	107.9	24.57
133.82	89.3	20.35
140.88	102.5	23.35
145.68	152.5	34.74
152.88	72.3	16.46
163.89	135.5	30.85
169.06	68.4	15.58
188.76	65.3	14.88
197.59	42.7	9.72
199.94	82.3	18.74
203.00	116.8	26.60
205.09	157.1	35.77
209.75	128.5	29.27
215.01	90.4	20.59
216.62	135.3	30.82

224.48	43.1	9.81
242.41	37.1	8.44
244.21	129.2	29.43
251.14	88.8	20.22
261.21	208.4	47.46
262.80	74.3	16.91
267.35	135.6	30.88
271.03	82.4	18.77
286.70	138.4	31.52
288.96	84.4	19.22
290.92	87.2	19.86
323.29	65.7	14.97
380.80	88.8	20.22
386.46	84.0	19.13
396.20	84.3	19.19
403.54	112.8	25.69
437.75	105.5	24.02
444.87	112.7	25.66
445.46	121.3	27.63
446.40	145.6	33.16
453.52	53.2	12.12
457.13	174.7	39.78
467.66	76.3	17.37
470.63	108.1	24.63
486.80	111.2	25.33

489.35	146.0	33.25
503.82	98.8	22.50
509.20	112.5	25.63
518.47	112.8	25.69
522.86	157.2	35.80
526.06	87.9	20.01
533.86	104.0	23.69
539.51	100.0	22.78
549.52	9.3	2.13
550.40	67.3	15.34
552.96	260.8	59.40
555.39	192.0	43.73
556.72	217.1	49.44
558.85	69.2	15.76
568.13	158.1	36.02
569.63	304.0	69.24
570.74	104.8	23.87
572.13	134.9	30.73
574.14	258.1	58.79
578.33	132.7	30.22
580.07	174.3	39.69
582.88	82.5	18.80
584.17	225.1	51.26
584.87	126.7	28.85
586.46	110.3	25.11

589.29	234.8	53.48
593.58	102.7	23.38
600.34	232.5	52.96
601.91	124.4	28.33

C:\Xcalibur\...\S\mona-abd-elatty-x4iiib

R MYCOLOGY AND BIOTECHNOLOGY

RT: 0.00 - 4.47 SM: 15G

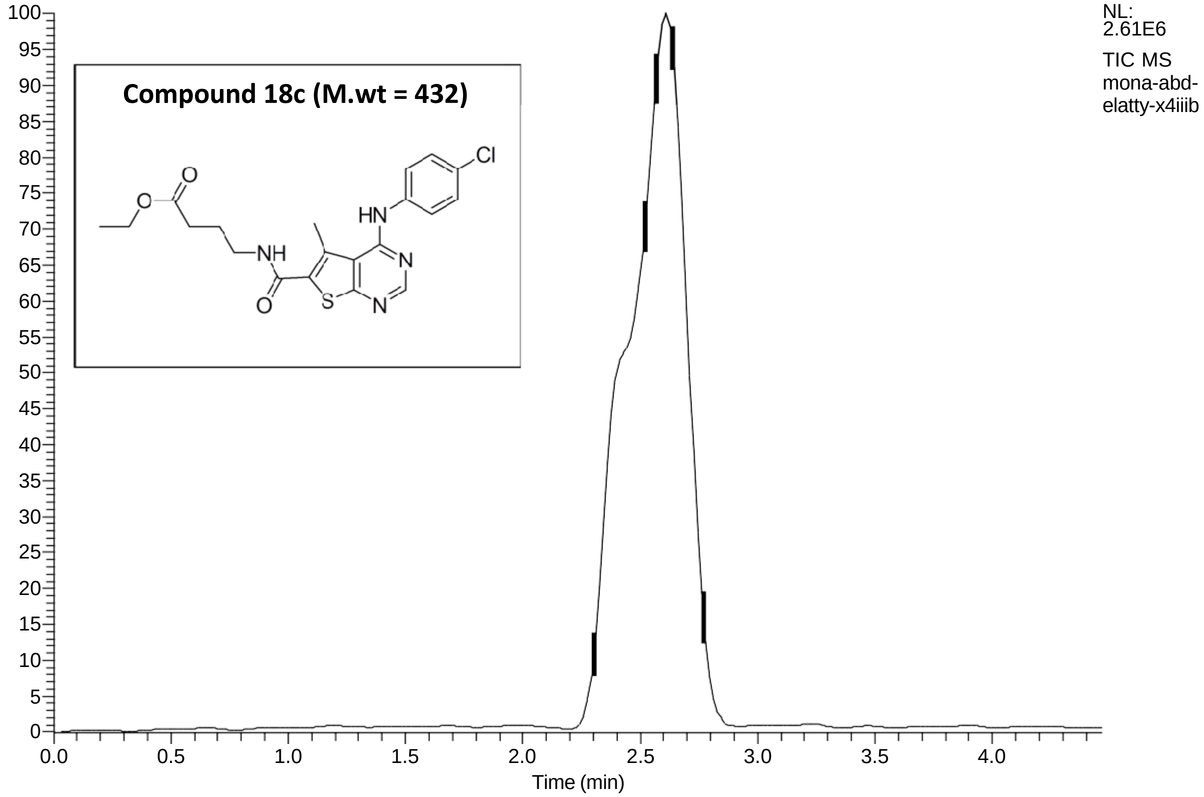

mona-abd-elatty-x4iiib #154 RT: 2.59 AV: 1 SB: 2 4.45 , 4.45 NL: 1.19E6
T: {0,0} + c EI Full ms [40.00-1000.00]

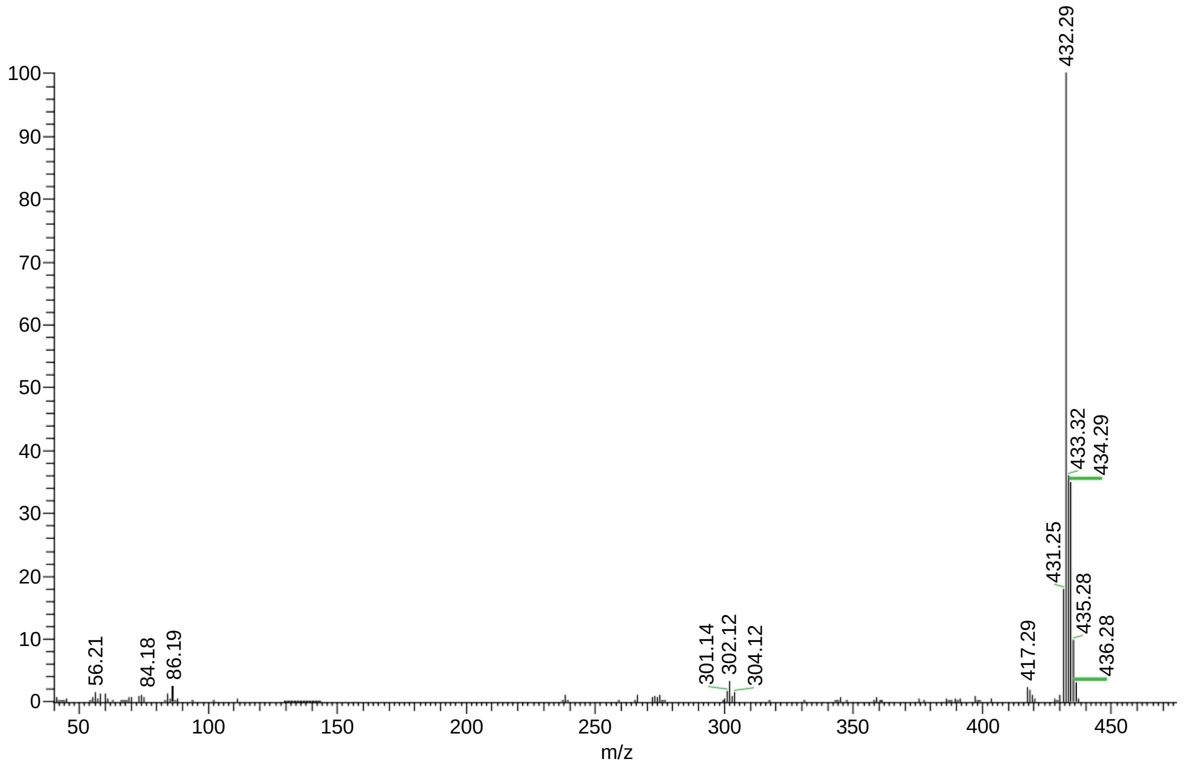

m/z	Intensity	Relative
56.21	17567.5	1.47
58.24	15839.5	1.33
60.12	15327.4	1.29
84.18	15725.5	1.32
86.19	28702.5	2.41
301.14	19819.5	1.66
302.12	39181.9	3.29
304.12	17877.9	1.50
417.29	26648.6	2.24
418.28	22061.5	1.85
431.25	214652.6	18.01
432.29	1191737.9	100.00
433.32	428043.0	35.92
434.29	416313.8	34.93
435.28	116577.5	9.78
436.28	37422.6	3.14

C:\Xcalibur\data\S\mona-abd-elatty-x3-iv

R MYCOLOGY AND BIOTECHNOLOGY

RT: 0.00 - 4.57 SM: 15G

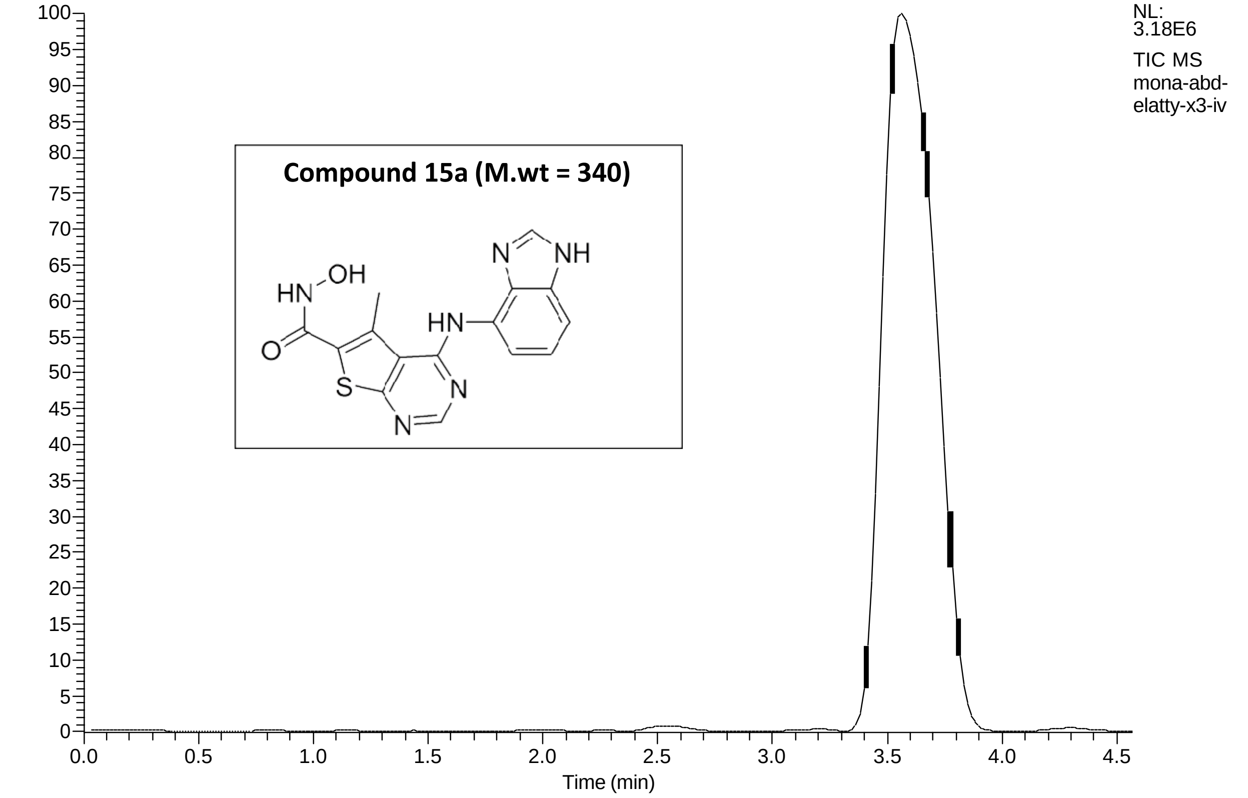

mona-abd-elatty-x3-iv #250 RT: 4.20 AV: 1 SB: 2 4.45 , 4.45 NL: 4.50E2
T: {0,0} + c EI Full ms [40.00-1000.00]

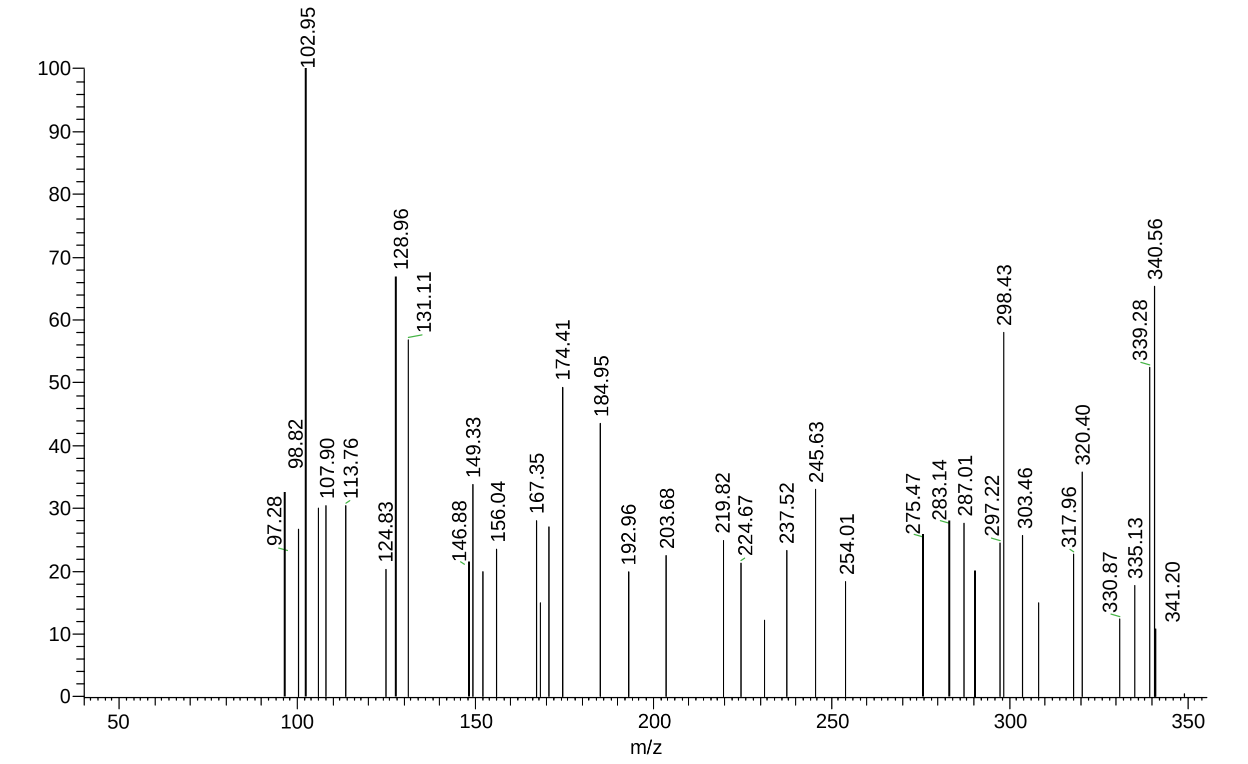

.

m/z	Intensity	Relative
97.28	102.8	22.86
98.82	158.1	35.16
100.45	119.9	26.65
102.01	154.0	34.24
102.95	449.7	100.00
106.09	135.2	30.06
107.90	137.2	30.51
113.76	136.5	30.36
124.83	91.6	20.37
127.16	89.3	19.86
128.96	301.2	66.97
131.11	255.6	56.83
146.88	92.9	20.66
149.33	152.5	33.92
152.03	89.3	19.86
156.04	105.9	23.54
167.35	126.7	28.16
168.15	66.9	14.88
170.54	122.0	27.13
174.41	221.9	49.33
184.95	195.7	43.52
192.96	89.6	19.92

203.68	101.3	22.53
219.82	112.3	24.96
224.67	96.1	21.38
231.32	54.7	12.16
237.52	105.1	23.36
245.63	148.8	33.09
254.01	82.1	18.26
275.47	112.7	25.05
283.14	122.4	27.22
287.01	124.5	27.69
297.22	110.1	24.49
298.43	261.0	58.05
303.46	115.9	25.76
308.27	67.7	15.06
317.96	101.7	22.62
320.40	161.2	35.84
330.87	55.6	12.36
335.13	80.3	17.85
339.28	235.7	52.42
340.56	293.8	65.34
341.20	48.3	10.73.

Thermo Fisher Scientific's SelectScreen™ Profiling Service: Single Point Results

SelectScreen Scientist: Joshua Campbell **Date:** 30-Aug-2019 **SSBK-Z'-LYTE (Madison, WI USA)**

Quality Assurance Review: Aaron Bergsma **Date:** 30-Aug-2019

**% Phosphorylation**	**Pass**
**Z' Determination**	**Pass**

Legend

- < 40% Inhibition
- 40% - 80% Inhibition
- ≥ 80% Inhibition

Project #	Compound Name	1X Test Compound Concentration (nM)	[ATP] Tested (µM)	Kinase Tested	% Inhibition Point 1	% Inhibition Point 2	% Inhibition mean	Difference Between Data Points |Point 1 - Point 2|	Development Reaction Interference	Test Compound Interference Coumarin	Test Compound Interference Fluorescein	Z'	Kinase Part# / Lot#
SSBK12643_56180	15c	10000	10	EGFR (ErbB1)	66	65	66	1	Pass	Pass	Pass	0.89	PV3872/1729964
SSBK12643_56180	11c	10000	10	EGFR (ErbB1)	10	11	11	1	Pass	Pass	Pass	0.89	PV3872/1729964
SSBK12643_56180	7c	10000	10	EGFR (ErbB1)	11	10	11	1	Pass	Pass	Pass	0.89	PV3872/1729964
SSBK12643_56180	20c	10000	10	KDR (VEGFR2)	33	36	35	4	Pass	Pass	Pass	0.63	PV3660/1825772
SSBK12643_56180	19c	10000	10	KDR (VEGFR2)	21	20	21	1	Pass	Pass	Pass	0.63	PV3660/1825772
SSBK12643_56180	15c	10000	10	KDR (VEGFR2)	86	89	87	3	Pass	Pass	Pass	0.63	PV3660/1825772
SSBK12643_56180	20d	10000	10	KDR (VEGFR2)	20	19	20	1	Pass	Pass	Pass	0.63	PV3660/1825772
SSBK12643_56180	19d	10000	10	KDR (VEGFR2)	27	25	26	1	Pass	Pass	Pass	0.63	PV3660/1825772
SSBK12643_56180	15d	10000	10	KDR (VEGFR2)	55	53	54	2	Pass	Pass	Pass	0.63	PV3660/1825772
SSBK12643_56180	20b	10000	10	KDR (VEGFR2)	25	25	25	0	Pass	Pass	Pass	0.72	PV3660/1825772
SSBK12643_56180	19b	10000	10	KDR (VEGFR2)	12	14	13	3	Pass	Pass	Pass	0.72	PV3660/1825772
SSBK12643_56180	15b	10000	10	KDR (VEGFR2)	32	32	32	0	Pass	Pass	Pass	0.63	PV3660/1825772

Thermo Fisher Scientific's SelectScreen™ Profiling Service: Single Point Results

SelectScreen Scientist: Joshua Campbell **Date:** 19-Jul-2019 **SSBK-Z'-LYTE (Madison, WI USA)**

Quality Assurance Review: Aaron Bergsma **Date:** 19-Jul-2019

**% Phosphorylation**	**Pass**
**Z' Determination**	**Pass**

Legend

- < 40% Inhibition
- 40% - 80% Inhibition
- ≥ 80% Inhibition

Project #	Compound Name	1X Test Compound Concentration (nM)	[ATP] Tested (µM)	Kinase Tested	% Inhibition Point 1	% Inhibition Point 2	% Inhibition mean	Difference Between Data Points |Point 1 - Point 2|	Development Reaction Interference	Test Compound Interference Coumarin	Test Compound Interference Fluorescein	Z'	Kinase Part# / Lot#
SSBK12643_55830	12c	10000	10	KDR (VEGFR2)	102	105	103	3	Pass	Pass	Pass	0.75	PV3660/1825772
SSBK12643_55830	11c	10000	10	KDR (VEGFR2)	95	87	91	8	Pass	Pass	Pass	0.75	PV3660/1825772
SSBK12643_55830	7c	10000	10	KDR (VEGFR2)	105	103	104	2	Pass	Pass	Pass	0.75	PV3660/1825772
SSBK12643_55830	20c	10000	10	EGFR (ErbB1)	92	90	91	2	Pass	Pass	Pass	0.84	PV3872/1729964
SSBK12643_55830	19c	10000	10	EGFR (ErbB1)	78	78	78	0	Pass	Pass	Pass	0.84	PV3872/1729964
SSBK12643_55830	15c	10000	10	EGFR (ErbB1)	97	97	97	0	Pass	Pass	Pass	0.84	PV3872/1729964
SSBK12643_55830	20d	10000	10	EGFR (ErbB1)	96	96	96	0	Pass	Pass	Pass	0.84	PV3872/1729964
SSBK12643_55830	19d	10000	10	EGFR (ErbB1)	96	97	96	1	Pass	Pass	Pass	0.84	PV3872/1729964
SSBK12643_55830	15d	10000	10	EGFR (ErbB1)	97	97	97	1	Pass	Pass	Pass	0.84	PV3872/1729964
SSBK12643_55830	20b	10000	10	EGFR (ErbB1)	93	91	92	2	Pass	Pass	Pass	0.84	PV3872/1729964
SSBK12643_55830	19b	10000	10	EGFR (ErbB1)	76	76	76	0	Pass	Pass	Pass	0.84	PV3872/1729964
SSBK12643_55830	15b	10000	10	EGFR (ErbB1)	97	98	98	0	Pass	Pass	Pass	0.84	PV3872/1729964

Thermo Fisher Scientific's SelectScreen™ Profiling Service: Single Point Results

SelectScreen Scientist: Joshua Campbell **Date:** 20-Oct-2019 **SSBK-Z'-LYTE (Madison, WI USA)**

Quality Assurance Review: Aaron Bergsma **Date:** 20-Oct-2019

**% Phosphorylation**	**Pass**
**Z' Determination**	**Pass**

Legend

- < 40% Inhibition
- 40% - 80% Inhibition
- ≥ 80% Inhibition

Project #	Compound Name	1X Test Compound Concentration (nM)	[ATP] Tested (µM)	Kinase Tested	% Inhibition Point 1	% Inhibition Point 2	% Inhibition mean	Difference Between Data Points |Point 1 - Point 2|	Development Reaction Interference	Test Compound Interference Coumarin	Test Compound Interference Fluorescein	Z'	Kinase Part# / Lot#
SSBK12643_56562	15a	10000	10	EGFR (ErbB1)	47	44	45	3	Pass	Pass	Pass	0.87	PV3872/1729964
SSBK12643_56562	15a	10000	10	KDR (VEGFR2)	36	35	36	1	Pass	Pass	Pass	0.55	PV3660/1825772
SSBK12643_56562	12a	10000	10	EGFR (ErbB1)	9	12	11	3	Pass	Pass	Pass	0.87	PV3872/1729964
SSBK12643_56562	12a	10000	10	KDR (VEGFR2)	47	50	48	3	Pass	Pass	Pass	0.55	PV3660/1825772
SSBK12643_56562	11a	10000	10	EGFR (ErbB1)	13	11	12	2	Pass	Pass	Pass	0.87	PV3872/1729964
SSBK12643_56562	11a	10000	10	KDR (VEGFR2)	48	46	47	2	Pass	Pass	Pass	0.55	PV3660/1825772
SSBK12643_56562	7a	10000	10	EGFR (ErbB1)	50	47	48	3	Pass	Pass	Pass	0.87	PV3872/1729964
SSBK12643_56562	7a	10000	10	KDR (VEGFR2)	86	92	89	6	Pass	Pass	Pass	0.55	PV3660/1825772
SSBK12643_56562	12b	10000	10	EGFR (ErbB1)	3	7	5	4	Pass	Pass	Pass	0.87	PV3872/1729964
SSBK12643_56562	12b	10000	10	KDR (VEGFR2)	85	82	83	3	Pass	Pass	Pass	0.55	PV3660/1825772
SSBK12643_56562	11b	10000	10	EGFR (ErbB1)	5	4	5	1	Pass	Pass	Pass	0.87	PV3872/1729964
SSBK12643_56562	11b	10000	10	KDR (VEGFR2)	76	74	75	2	Pass	Pass	Pass	0.55	PV3660/1825772
SSBK12643_56562	7b	10000	10	EGFR (ErbB1)	39	39	39	1	Pass	Pass	Pass	0.87	PV3872/1729964
SSBK12643_56562	7b	10000	10	KDR (VEGFR2)	75	81	78	6	Pass	Pass	Pass	0.55	PV3660/1825772
SSBK12643_56562	7d	10000	10	EGFR (ErbB1)	79	78	78	1	Pass	Pass	Pass	0.87	PV3872/1729964
SSBK12643_56562	7d	10000	10	KDR (VEGFR2)	44	46	45	2	Pass	Pass	Pass	0.55	PV3660/1825772

Thermo Fisher Scientific's SelectScreen™ Profiling Service: Single Point Results

SelectScreen Scientist: Joshua Campbell **Date:** 13-Dec-2019 **SSBK-Z'-LYTE (Madison, WI USA)**

Quality Assurance Review: Joseph Vance **Date:** 13-Dec-2019

**% Phosphorylation**	**Pass**
**Z' Determination**	**Pass**

Legend

- < 40% Inhibition
- 40% - 80% Inhibition
- ≥ 80% Inhibition

Project #	Compound Name	1X Test Compound Concentration (nM)	[ATP] Tested (µM)	Kinase Tested	% Inhibition Point 1	% Inhibition Point 2	% Inhibition mean	Difference Between Data Points |Point 1 - Point 2|	Development Reaction Interference	Test Compound Interference Coumarin	Test Compound Interference Fluorescein	Z'	Kinase Part# / Lot#
SSBK12643_57022	12c	1000	10	KDR (VEGFR2)	91	90	90	0	Pass	Pass	Pass	0.66	PV3660/1825772
SSBK12643_57022	12c	100	10	KDR (VEGFR2)	34	38	36	4	Pass	Pass	Pass	0.66	PV3660/1825772
SSBK12643_57022	12c	10	10	KDR (VEGFR2)	12	-2	5	15	Pass	Pass	Pass	0.66	PV3660/1825772
SSBK12643_57022	12c	1	10	KDR (VEGFR2)	14	6	10	8	Pass	Pass	Pass	0.66	PV3660/1825772
SSBK12643_57022	12c	0.1	10	KDR (VEGFR2)	11	-1	5	12	Pass	Pass	Pass	0.66	PV3660/1825772
SSBK12643_57022	7c	1000	10	KDR (VEGFR2)	94	86	90	8	Pass	Pass	Pass	0.66	PV3660/1825772
SSBK12643_57022	7c	100	10	KDR (VEGFR2)	38	33	36	4	Pass	Pass	Pass	0.66	PV3660/1825772
SSBK12643_57022	7c	10	10	KDR (VEGFR2)	3	0	2	3	Pass	Pass	Pass	0.66	PV3660/1825772

Project #	Compound Name	1X Test Compound Concentration (nM)	[ATP] Tested (µM)	Kinase Tested	% Inhibition Point 1	% Inhibition Point 2	% Inhibition mean	Difference Between Data Points |Point 1 - Point 2|	Development Reaction Interference	Test Compound Interference Coumarin	Test Compound Interference Fluorescein	Z'	Kinase Part# / Lot#
SSBK12643 57022	7c	1	10	KDR (VEGFR2)	7	5	6	2	Pass	Pass	Pass	0.66	PV3660/1825772
SSBK12643 57022	7c	0.1	10	KDR (VEGFR2)	7	4	6	2	Pass	Pass	Pass	0.66	PV3660/1825772
SSBK12643 57022	15c	1000	10	EGFR (ErbB1)	95	94	95	0	Pass	Pass	Pass	0.78	PV3872/2061314
SSBK12643 57022	15c	100	10	EGFR (ErbB1)	81	80	81	1	Pass	Pass	Pass	0.78	PV3872/2061314
SSBK12643 57022	15c	10	10	EGFR (ErbB1)	34	35	34	1	Pass	Pass	Pass	0.78	PV3872/2061314
SSBK12643 57022	15c	1	10	EGFR (ErbB1)	4	6	5	2	Pass	Pass	Pass	0.78	PV3872/2061314
SSBK12643 57022	15c	0.1	10	EGFR (ErbB1)	1	-1	0	2	Pass	Pass	Pass	0.78	PV3872/2061314
SSBK12643 57022	20d	1000	10	EGFR (ErbB1)	92	93	93	1	Pass	Pass	Pass	0.78	PV3872/2061314
SSBK12643 57022	20d	100	10	EGFR (ErbB1)	58	58	58	0	Pass	Pass	Pass	0.78	PV3872/2061314
SSBK12643 57022	20d	10	10	EGFR (ErbB1)	15	13	14	2	Pass	Pass	Pass	0.78	PV3872/2061314
SSBK12643 57022	20d	1	10	EGFR (ErbB1)	5	5	5	0	Pass	Pass	Pass	0.78	PV3872/2061314
SSBK12643 57022	20d	0.1	10	EGFR (ErbB1)	1	0	1	1	Pass	Pass	Pass	0.78	PV3872/2061314
SSBK12643 57022	20b	1000	10	EGFR (ErbB1)	85	85	85	0	Pass	Pass	Pass	0.78	PV3872/2061314
SSBK12643 57022	20b	100	10	EGFR (ErbB1)	34	36	35	2	Pass	Pass	Pass	0.78	PV3872/2061314
SSBK12643 57022	20b	10	10	EGFR (ErbB1)	9	6	7	3	Pass	Pass	Pass	0.78	PV3872/2061314
SSBK12643 57022	20b	1	10	EGFR (ErbB1)	3	0	1	3	Pass	Pass	Pass	0.78	PV3872/2061314
SSBK12643 57022	20b	0.1	10	EGFR (ErbB1)	3	0	2	3	Pass	Pass	Pass	0.78	PV3872/2061314

Thermo Fisher Scientific's SelectScreen™ Profiling Service: Single Point Results

SelectScreen Scientist: Joshua Campbell **Date:** 09-Jan-2020

SSBK-Z'-LYTE (Madison, WI USA)

Quality Assurance Review: Joseph Vance **Date:** 09-Jan-2020

**% Phosphorylation**	**Pass**
**Z' Determination**	**Pass**

Legend

- < 40% Inhibition
- 40% - 80% Inhibition
- ≥ 80% Inhibition

Project #	Compound Name	1X Test Compound Concentration (nM)	[ATP] Tested (µM)	Kinase Tested	% Inhibition Point 1	% Inhibition Point 2	% Inhibition mean	Difference Between Data Points |Point 1 - Point 2|	Development Reaction Interference	Test Compound Interference Coumarin	Test Compound Interference Fluorescein	Z'	Kinase Part# / Lot#
SSBK12643 57145	12c	1000	10	EGFR (ErbB1)	36	32	34	4	Pass	Pass	Pass	0.76	PV3872/2061314
SSBK12643 57145	12c	100	10	EGFR (ErbB1)	9	5	7	4	Pass	Pass	Pass	0.76	PV3872/2061314
SSBK12643 57145	12c	10	10	EGFR (ErbB1)	4	4	4	0	Pass	Pass	Pass	0.76	PV3872/2061314
SSBK12643 57145	12c	1	10	EGFR (ErbB1)	6	2	4	4	Pass	Pass	Pass	0.76	PV3872/2061314
SSBK12643 57145	12c	0.1	10	EGFR (ErbB1)	4	8	6	4	Pass	Pass	Pass	0.76	PV3872/2061314
SSBK12643 57145	15c	1000	10	KDR (VEGFR2)	40	38	39	2	Pass	Pass	Pass	0.64	PV3660/1825772
SSBK12643 57145	15c	100	10	KDR (VEGFR2)	19	11	15	8	Pass	Pass	Pass	0.64	PV3660/1825772
SSBK12643 57145	15c	10	10	KDR (VEGFR2)	3	10	7	7	Pass	Pass	Pass	0.64	PV3660/1825772
SSBK12643 57145	15c	1	10	KDR (VEGFR2)	3	5	4	2	Pass	Pass	Pass	0.64	PV3660/1825772
SSBK12643 57145	15c	0.1	10	KDR (VEGFR2)	6	7	7	1	Pass	Pass	Pass	0.64	PV3660/1825772

6042 Cornerstone Court West, Suite B
San Diego, CA 92121
Tel: 1.858.829.3082
Fax: 1.858.481.8694
Email: info@bpsbioscience.com

Assay Report

Histone Deacetylase (HDAC) Inhibitor Assays

Enzymatic Study of Twenty Compounds from Misr International University

6042 Cornerstone Court West, Suite B
San Diego, CA 92121
Tel: 1.858.829.3082
Fax: 1.858.481.8694
Email: info@bpsbioscience.com

MIU_HDAC_191024

HDAC Inhibitor Assays

Study Sponsor:	Misr International University
Attention:	Mona Abdelatty
Address:	Misr International University<br>Km 28 Cairo Ismailia Rd<br>Ahmed Orab District<br>Cairo-Egypt 11431
Study Director:	Henry Zhu, Ph.D.
Testing Facility:	BPS Bioscience Inc.<br>6042 Cornerstone Court West, Ste. B<br>San Diego, CA 92121<br>USA
Study Period:	
Report Version:	1
Report Date:	October 24, 2019

6042 Cornerstone Court West, Suite B
San Diego, CA 92121
Tel: 1.858.829.3082
Fax: 1.858.481.8694
Email: info@bpsbioscience.com

Study Director

Victoria Castillo
Research Associate I

10-24-19

Date

Henry Zhu, Ph.D.
President

10-24-19

Date

6042 Cornerstone Court West, Suite B
San Diego, CA 92121
Tel: 1.858.829.3082
Fax: 1.858.481.8694
Email: info@bpsbioscience.com

CONTENTS

HISTONE DEACETYLASE (HDAC) INHIBITOR ASSAYS **1**

ENZYMATIC STUDY OF TWENTY COMPOUNDS FROM MISR INTERNATIONAL UNIVERSITY **1**

HDAC INHIBITOR ASSAYS **2**

STUDY DIRECTOR **3**

1. PURPOSE OF THE STUDY **5**

2. MATERIALS AND METHODS **6**

2.1 MATERIALS 6
2.2 COMPOUNDS 6
2.3 EXPERIMENTAL CONDITIONS 7
2.3.1 Enzymes and Substrates ***7***
2.3.2 Assay Conditions ***7***
2.3.3 Data Analysis ***8***

3. ASSAY RESULTS **9**

3.1. SUMMARY OF THE INHIBITORY EFFECTS OF THE COMPOUNDS ON HDAC ACTIVITIES 9
3.2. RESULTS OF THE EFFECTS OF THE COMPOUNDS ON INDIVIDUAL HDAC ACTIVITY 10
3.2.1. HDAC6 .... ***10***
3.2.1.1. Twenty Compounds ***10***
Table 3.2.1.1. Data for the Effect of Twenty Compounds on HDAC6 Activity 10

4. QUALITY ASSURANCE STATEMENT **12**

6042 Cornerstone Court West, Suite B
San Diego, CA 92121
Tel: 1.858.829.3082
Fax: 1.858.481.8694
Email: info@bpsbioscience.com

1. Purpose of the Study

The purpose of the study is to determine the effects of twenty compounds from Misr International University on the enzymatic activities of recombinant HDAC6 using an *in vitro* enzymatic assay.

6042 Cornerstone Court West, Suite B
San Diego, CA 92121
Tel: 1.858.829.3082
Fax: 1.858.481.8694
Email: info@bpsbioscience.com

2. Materials and Methods

2.1 Materials

TSA is purchased from Selleckchem (Houston, TX, Catalog number S1045)
HDAC Assay Buffer (BPS catalog number 50031)
HDAC Assay Developer (BPS catalog number 50030)
HDAC Substrate 3 (BPS catalog number 50037)

2.2 Compounds

The test compounds are supplied by Misr International University.

Compound I.D.	Compound Supplied	Stock Concentration	Dissolving Solvent	Test Range (µM)	Intermediate Dilution
20b	Liquid	10 mM	DMSO	10	10 % DMSO in HDAC Assay Buffer
19b	Liquid	10 mM	DMSO	10	10 % DMSO in HDAC Assay Buffer
15b	Liquid	10 mM	DMSO	10	10 % DMSO in HDAC Assay Buffer
12c	Liquid	10 mM	DMSO	10	10 % DMSO in HDAC Assay Buffer
11c	Liquid	10 mM	DMSO	10	10 % DMSO in HDAC Assay Buffer
7c	Liquid	10 mM	DMSO	10	10 % DMSO in HDAC Assay Buffer
15a	Liquid	10 mM	DMSO	10	10 % DMSO in HDAC Assay Buffer
20c	Liquid	10 mM	DMSO	10	10 % DMSO in HDAC Assay Buffer
19c	Liquid	10 mM	DMSO	10	10 % DMSO in HDAC Assay Buffer
15c	Liquid	10 mM	DMSO	10	10 % DMSO in HDAC Assay Buffer
20d	Liquid	10 mM	DMSO	10	10 % DMSO in HDAC Assay Buffer
19d	Liquid	10 mM	DMSO	10	10 % DMSO in HDAC Assay Buffer
15d	Liquid	10 mM	DMSO	10	10 % DMSO in HDAC Assay Buffer
12a	Liquid	10 mM	DMSO	10	10 % DMSO in HDAC Assay Buffer

6042 Cornerstone Court West, Suite B
San Diego, CA 92121
Tel: 1.858.829.3082
Fax: 1.858.481.8694
Email: info@bpsbioscience.com

11a	Liquid	10 mM	DMSO	10	10 % DMSO in HDAC Assay Buffer
7a	Liquid	10 mM	DMSO	10	10 % DMSO in HDAC Assay Buffer
12b	Liquid	10 mM	DMSO	10	10 % DMSO in HDAC Assay Buffer
11b	Liquid	10 mM	DMSO	10	10 % DMSO in HDAC Assay Buffer
7b	Liquid	10 mM	DMSO	10	10 % DMSO in HDAC Assay Buffer
7d	Liquid	10 mM	DMSO	10	10 % DMSO in HDAC Assay Buffer
TSA*	Solid	10 mM	DMSO	0.0005, 0.005, 0.05	10 % DMSO in HDAC Assay Buffer

*Reference Compound.

2.3 Experimental Conditions

2.3.1 Enzymes and Substrates

Assay	Catalog #	Enzyme Lot #	Enzyme Used (ng) / Reaction	Substrate
HDAC6	50006	190529-G2	40	10 µM HDAC Substrate 3

2.3.2 Assay Conditions

All of the compounds are dissolved in DMSO. A series of dilutions of the compounds were prepared with 10% DMSO in HDAC assay buffer and 5 µl of the dilution was added to a 50 µl reaction so that the final concentration of DMSO is 1% in all of reactions.

The enzymatic reactions for the HDAC enzymes were conducted in duplicate at 37ºC for 30 minutes in a 50 µl mixture containing HDAC assay buffer, 5 µg BSA, an HDAC substrate (see 2.3.1), a HDAC enzyme (see 2.3.1) and a test compound (see 2.2).

After enzymatic reactions, 50 µl of 2 x HDAC Developer was added to each well for the HDAC enzymes and the plate was incubated at room temperature for an additional 15 minutes.

6042 Cornerstone Court West, Suite B
San Diego, CA 92121
Tel: 1.858.829.3082
Fax: 1.858.481.8694
Email: info@bpsbioscience.com

Fluorescence intensity was measured at an excitation of 360 nm and an emission of 460 nm using a Tecan Infinite M1000 microplate reader.

2.3.3 Data Analysis

HDAC activity assays were performed in duplicates at each concentration. The fluorescent intensity data were analyzed using the computer software, Graphpad Prism. In the absence of the compound, the fluorescent intensity (F_t) in each data set was defined as 100% activity. In the absence of HDAC, the fluorescent intensity (F_b) in each data set was defined as 0% activity. The percent activity in the presence of each compound was calculated according to the following equation: %activity = $(F-F_b)/(F_t-F_b)$, where F= the fluorescent intensity in the presence of the compound.

The values of percentage activity were plotted on a bar graph.

6042 Cornerstone Court West, Suite B
San Diego, CA 92121
Tel: 1.858.829.3082
Fax: 1.858.481.8694
Email: info@bpsbioscience.com

3. Assay Results

3.1. Summary of the Inhibitory Effects of the Compounds on HDAC Activities

The mean percent inhibition values of twenty compounds at different concentrations are summarized in Table 3.1.

Table 3.1 Inhibitory Effects of the Compounds on HDAC Activities

Compound I.D.	% Inhibition			
	**0.0005 μM**	**0.005 μM**	**0.05 μM**	**10 μM**
20b	-	-	-	56
19b	-	-	-	10
15b	-	-	-	30
12c	-	-	-	23
11c	-	-	-	8
7c	-	-	-	8
15a	-	-	-	7
20c	-	-	-	9
19c	-	-	-	7
15c	-	-	-	9
20d	-	-	-	0
19d	-	-	-	4
15d	-	-	-	7
12a	-	-	-	7
11a	-	-	-	5
7a	-	-	-	7
12b	-	-	-	13
11b	-	-	-	6
7b	-	-	-	8
7d	-	-	-	6
TSA	23	73	95	-

BPS Bioscience

6042 Cornerstone Court West, Suite B
San Diego, CA 92121
Tel: 1.858.829.3082
Fax: 1.858.481.8694
Email: info@bpsbioscience.com

3.2. Results of the Effects of the Compounds on Individual HDAC Activity

3.2.1. HDAC6

3.2.1.1. Twenty Compounds

Table 3.2.1.1. Data for the Effect of Twenty Compounds on HDAC6 Activity

Compound I.D.	HDAC6 Activity (Fluorescence count)		% Activity		% Inhibition
	Repeat 1	Repeat 2	Repeat 1	Repeat 2	
No Compound	43086	43211	100	100	0
20b	20383	20104	45	44	56
19b	39239	38977	90	90	10
15b	31062	30706	71	70	30
12c	34046	33527	78	77	23
11c	39315	40752	91	94	8
7c	39658	40013	92	92	8
15a	39905	40763	92	94	7
20c	39352	39496	91	91	9
19c	40313	40340	93	93	7
15c	39546	39320	91	91	9
20d	42596	43318	99	100	0
19d	41449	41579	96	96	4
15d	40267	40649	93	94	7
12a	39814	40409	92	93	7
11a	40865	41129	94	95	5
7a	39756	40644	92	94	7
12b	37984	37990	87	87	13
11b	40208	40927	93	95	6
7b	40317	39305	93	91	8
7d	40491	40611	94	94	6
TSA, 0.0005 µM	33793	33862	77	77	23
TSA, 0.005 µM	13050	12973	27	27	73
TSA, 0.05 µM	4156	3909	5	5	95
Background	2383	2339			

6042 Cornerstone Court West, Suite B
San Diego, CA 92121
Tel: 1.858.829.3082
Fax: 1.858.481.8694
Email: info@bpsbioscience.com

HDAC6 Activity

Substrate Conc.=10 μM Substrate 3 (50037)

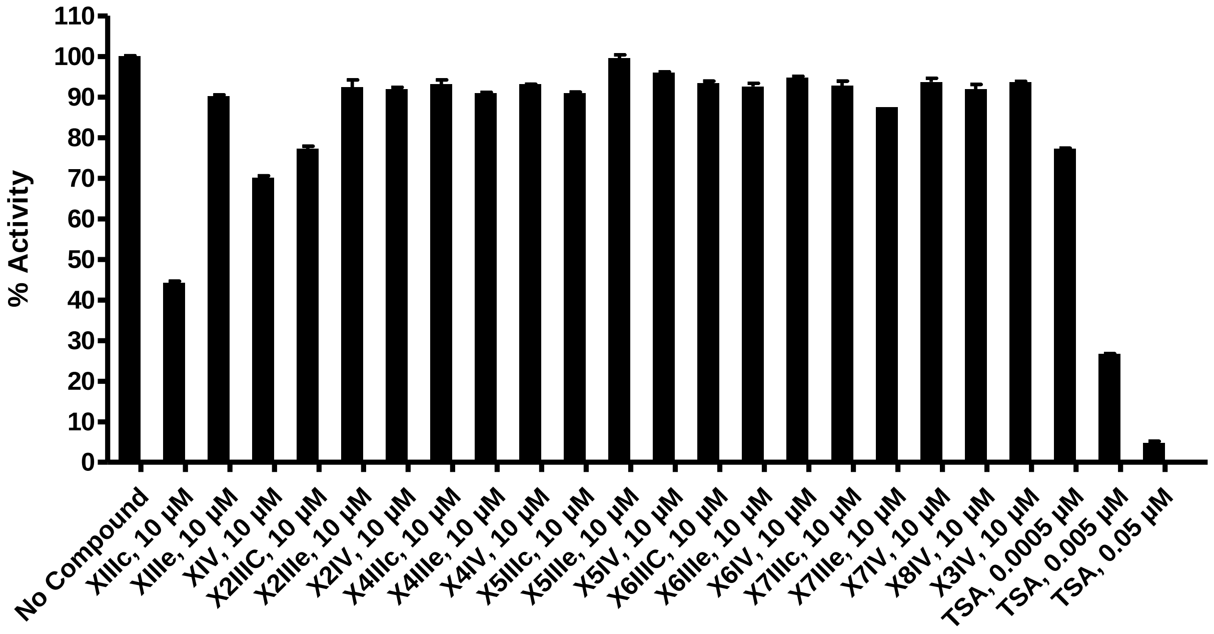

6042 Cornerstone Court West, Suite B
San Diego, CA 92121
Tel: 1.858.829.3082
Fax: 1.858.481.8694
Email: info@bpsbioscience.com

4. Quality Assurance Statement

I certify that the results presented in this report were generated using the materials and methods mentioned and that these results reflect the Raw Data.

Henry Zhu, Ph.D.
President

10-24-19

Date

6042 Cornerstone Court West, Suite B
San Diego, CA 92121
Tel: 1.858.829.3082
Fax: 1.858.481.8694
Email: info@bpsbioscience.com

Assay Report

Hela Nuclear Extract (HNU) Inhibitor Assays

Enzymatic Study of Twenty Compounds from Misr International University

6042 Cornerstone Court West, Suite B
San Diego, CA 92121
Tel: 1.858.829.3082
Fax: 1.858.481.8694
Email: info@bpsbioscience.com

MIU_HNU_191021

Hela Nuclear Extract Inhibitor Assays

Study Sponsor:	Misr International University
Attention:	Mona Abdelatty
Address:	Misr International University<br>Km 28 Cairo Ismailia Rd<br>Ahmed Orab District<br>Cairo-Egypt 11431
Study Director:	Henry Zhu, Ph.D.
Testing Facility:	BPS Bioscience Inc.<br>6042 Cornerstone Court West, Ste. B<br>San Diego, CA 92121<br>USA
Study Period:	
Report Version:	1
Report Date:	October 21, 2019

6042 Cornerstone Court West, Suite B
San Diego, CA 92121
Tel: 1.858.829.3082
Fax: 1.858.481.8694
Email: info@bpsbioscience.com

Study Director

Kevin Kurtz
Sr. Scientist II.

10-21-19

Date

Henry Zhu, Ph.D.
President

10-21-19

Date

6042 Cornerstone Court West, Suite B
San Diego, CA 92121
Tel: 1.858.829.3082
Fax: 1.858.481.8694
Email: info@bpsbioscience.com

CONTENTS

HISTONE DEACETYLASE (HDAC) INHIBITOR ASSAYS 1

ENZYMATIC STUDY OF TWENTY COMPOUNDS FROM MISR INTERNATIONAL UNIVERSITY 1

HELA NUCLEAR EXTRACT INHIBITOR ASSAYS 2

STUDY DIRECTOR 3

1. PURPOSE OF THE STUDY 5

2. MATERIALS AND METHODS 6

2.1 MATERIALS 6
2.2 COMPOUNDS 6
2.3 EXPERIMENTAL CONDITIONS 7
2.3.1 Enzymes and Substrates *7*
2.3.2 Assay Conditions *7*
2.3.3 Data Analysis *8*

3. ASSAY RESULTS 9

3.1. SUMMARY OF THE INHIBITORY EFFECTS OF THE COMPOUND ON INDIVIDUAL HELA NUCLEAR EXTRACT ACTIVITIES 9
3.2. RESULTS OF THE EFFECTS OF THE COMPOUNDS ON HELA NUCLEAR EXTRACT ACTIVITY 11
3.2.1. Hela Nuclear Extract *11*

4. QUALITY ASSURANCE STATEMENT 13

6042 Cornerstone Court West, Suite B
San Diego, CA 92121
Tel: 1.858.829.3082
Fax: 1.858.481.8694
Email: info@bpsbioscience.com

1. Purpose of the Study

The purpose of the study is to determine the effects of twenty compounds from Misr International University on the enzymatic activities of hela nuclear extracts using an in vitro enzymatic assay.

6042 Cornerstone Court West, Suite B
San Diego, CA 92121
Tel: 1.858.829.3082
Fax: 1.858.481.8694
Email: info@bpsbioscience.com

2. Materials and Methods

2.1 Materials

HDAC Assay Buffer (BPS catalog number 50031)
HDAC Assay Developer (BPS catalog number 50030)
HDAC Substrate 3 (BPS number 50037)
TSA is purchased from Selleck (Houston, TX, Catalog number S1045).
Nuclear Extraction Kit from abcam (Cambridge, MA, Cat # ab113474)

2.2 Compounds

The test compounds are supplied by Misr International University.

Compound I.D.	Compound Supplied	Stock Concentration	Dissolving Solvent	Test Range (μM)	Intermediate Dilution
20b	Solution	10mM	DMSO	10	10 % DMSO in HDAC Assay Buffer
19b	Solution	10mM	DMSO	10	10 % DMSO in HDAC Assay Buffer
15b	Solution	10mM	DMSO	10	10 % DMSO in HDAC Assay Buffer
12c	Solution	10mM	DMSO	10	10 % DMSO in HDAC Assay Buffer
11c	Solution	10mM	DMSO	10	10 % DMSO in HDAC Assay Buffer
7c	Solution	10mM	DMSO	10	10 % DMSO in HDAC Assay Buffer
15a	Solution	10mM	DMSO	10	10 % DMSO in HDAC Assay Buffer
20c	Solution	10mM	DMSO	10	10 % DMSO in HDAC Assay Buffer
19c	Solution	10mM	DMSO	10	10 % DMSO in HDAC Assay Buffer
15c	Solution	10mM	DMSO	10	10 % DMSO in HDAC Assay Buffer
20d	Solution	10mM	DMSO	10	10 % DMSO in HDAC Assay Buffer
19d	Solution	10mM	DMSO	10	10 % DMSO in HDAC Assay Buffer
15d	Solution	10mM	DMSO	10	10 % DMSO in HDAC Assay Buffer

6042 Cornerstone Court West, Suite B
San Diego, CA 92121
Tel: 1.858.829.3082
Fax: 1.858.481.8694
Email: info@bpsbioscience.com

12a	Solution	10mM	DMSO	10	10 % DMSO in HDAC Assay Buffer
11a	Solution	10mM	DMSO	10	10 % DMSO in HDAC Assay Buffer
7a	Solution	10mM	DMSO	10	10 % DMSO in HDAC Assay Buffer
12b	Solution	10mM	DMSO	10	10 % DMSO in HDAC Assay Buffer
11b	Solution	10mM	DMSO	10	10 % DMSO in HDAC Assay Buffer
7b	Solution	10mM	DMSO	10	10 % DMSO in HDAC Assay Buffer
7d	Solution	10mM	DMSO	10	10 % DMSO in HDAC Assay Buffer
TSA*	Solid	10mM	DMSO	0.03, 0.003, 0.0003	10 % DMSO in HDAC Assay Buffer

*Reference Compound

2.3 Experimental Conditions

2.3.1 Enzymes and Substrates

Assay	Catalog #	Substrate
Hela Nuclear Exctract	50053	10 µM HDAC Substrate 3

2.3.2 Assay Conditions

For the hela nuclear extracts, hela cells were grown to 90 % confluency. Hela nuclear extracts were prepared from the Nuclear Extraction Kit from abcam (Catalog # ab113474). The final total protein concentration was calculated to be 2.4 µg/µl. From this, the hela nuclear extracts was diluted 20-fold in HDAC assay buffer and 5 µl of the dilution was added to a 50 µl reaction.

The compounds are dissolved in DMSO. A series of dilutions of the compounds were prepared with 10% DMSO in HDAC assay buffer and 5 µl of the dilution was added to a 50 µl reaction so that the final concentration of DMSO is 1 % in all of reactions.

6042 Cornerstone Court West, Suite B
San Diego, CA 92121
Tel: 1.858.829.3082
Fax: 1.858.481.8694
Email: info@bpsbioscience.com

The enzymatic reactions for the hela nuclear extract were conducted in duplicate at 37 ºC for 60 minutes in a 50 µl mixture containing HDAC assay buffer, 5 µg BSA, an HDAC substrate (see 2.3.1), hela nuclear extract, and a test compound (see 2.2).

After enzymatic reactions, 50µl of 2 x HDAC Developer was added to each well for the hela nuclear extract and the plate was incubated at room temperature for an additional 15 minutes.

Fluorescence intensity was measured at an excitation of 360 nm and an emission of 460 nm using a Tecan Infinite M1000 microplate reader.

2.3.3 Data Analysis

Hela nuclear extract activity assays were performed in duplicates at each concentration. The fluorescent intensity data were analyzed using the computer software, Graphpad Prism. In the absence of the compound, the fluorescent intensity (F_t) in each data set was defined as 100% activity. In the absence of hela nuclear extract, the fluorescent intensity (F_b) in each data set was defined as 0% activity. The percent activity in the presence of each compound was calculated according to the following equation: %activity = $(F-F_b)/(F_t-F_b)$, where F= the fluorescent intensity in the presence of the compound.

The values of percentage activity were plotted on a bar graph.

6042 Cornerstone Court West, Suite B
San Diego, CA 92121
Tel: 1.858.829.3082
Fax: 1.858.481.8694
Email: info@bpsbioscience.com

3. Assay Results

3.1. Summary of the Inhibitory Effects of the Compound on Individual Hela Nuclear Extract Activities

The percentage inhibition of the twenty compounds against hela nuclear extracts is summarized on Table 3.1.

Table 3.1 Inhibitory Effects of the Compounds on HDAC Activities

Inhibitors	% Inhibition
	**Hela Nuclear Extracts**
20b	21
19b	5
15b	9
12c	7
11c	5
7c	3
15a	3
20c	3
19c	7
15c	4
20d	0
19d	2
15d	4
12a	2

6042 Cornerstone Court West, Suite B
San Diego, CA 92121
Tel: 1.858.829.3082
Fax: 1.858.481.8694
Email: info@bpsbioscience.com

11a	0
7a	2
12b	3
11b	4
7b	2
7d	3
TSA, 0.0003 µM	7
TSA, 0.003 µM	46
TSA, 0.03 µM	88

6042 Cornerstone Court West, Suite B
San Diego, CA 92121
Tel: 1.858.829.3082
Fax: 1.858.481.8694
Email: info@bpsbioscience.com

3.2. Results of the Effects of the Compounds on Hela Nuclear Extract Activity

3.2.1. Hela Nuclear Extract

Table 3.2.1. Data for the Effect of the Compounds on Hela Nuclear Extract Activity

Compounds	Activity (Fluorescence count)		% Activity		% Inhibition
	Repeat1	Repeat2	Repeat1	Repeat2	
No Compound	2066	2126	98	102	0
20b	1684	1718	78	80	21
19b	1976	2018	94	96	5
15b	1943	1930	92	91	9
12c	1898	2016	89	96	7
11c	2021	1988	96	94	5
7c	2001	2076	95	99	3
15a	2024	2048	96	97	3
20c	2036	2052	97	98	3
19c	2023	1926	96	91	7
15c	1995	2063	95	98	4
20d	2097	2096	100	100	0
19d	2068	2057	98	98	2
15d	2028	2010	96	95	4
12a	2072	2046	99	97	2
11a	2084	2132	99	102	0
7a	2055	2052	98	98	2
12b	1986	2094	94	100	3
11b	1978	2074	94	99	4
7b	2023	2087	96	100	2
7d	2016	2051	96	98	3
TSA, 0.0003 μM	1952	1983	92	94	7
TSA, 0.003 μM	1251	1240	54	54	46
TSA, 0.03 μM	482	460	13	12	88
Background	249	235			

6042 Cornerstone Court West, Suite B
San Diego, CA 92121
Tel: 1.858.829.3082
Fax: 1.858.481.8694
Email: info@bpsbioscience.com

Hela Nuclear Extract Activity

Substrate Conc.=10 µM Substrate 3 (50037)

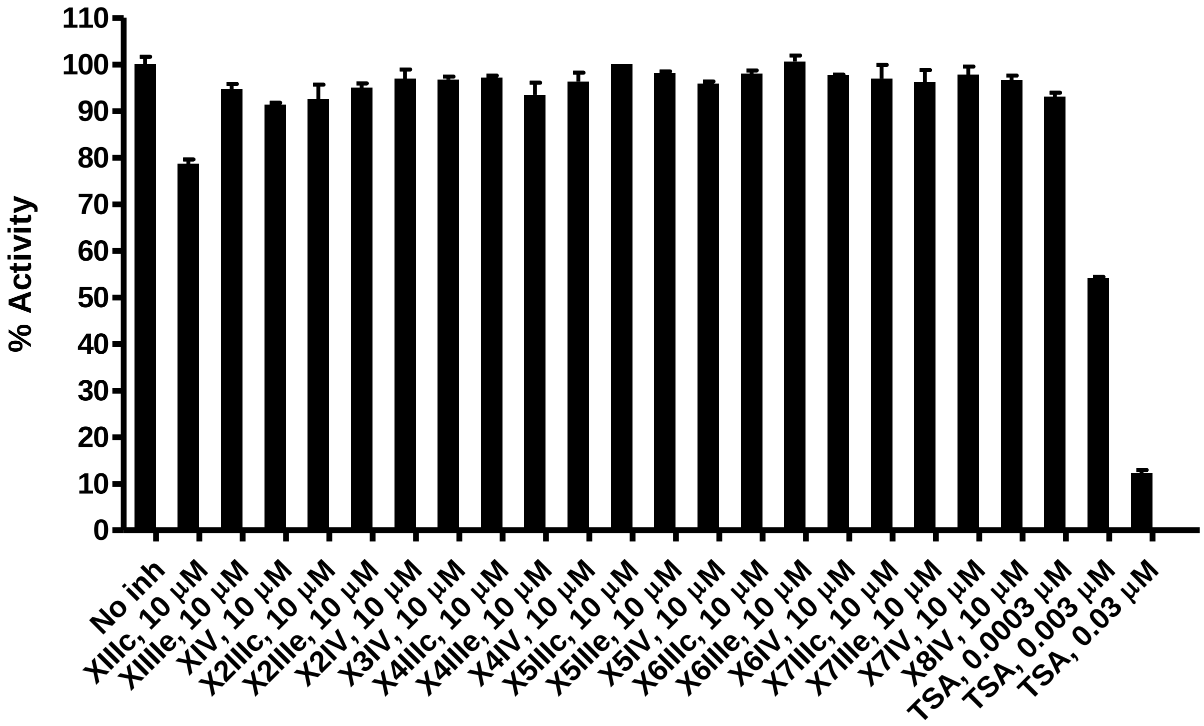

6042 Cornerstone Court West, Suite B
San Diego, CA 92121
Tel: 1.858.829.3082
Fax: 1.858.481.8694
Email: info@bpsbioscience.com

4. Quality Assurance Statement

I certify that the results presented in this report were generated using the materials and methods mentioned and that these results reflect the Raw Data.

Henry Zhu, Ph.D.
President

10-21-19

Date

Developmental Therapeutics Program

One Dose Mean Graph

NSC: D-820172 / 1 | **Conc** [illegible]

Experiment ID: 1910OS87 | compound 7b | **Report Date:** Nov 23, 2019

Panel/Cell Line	Growth Percent
Leukemia	
CCRF-CEM	101.22
HL-60(TB)	103.15
K-562	108.57
MOLT-4	112.45
RPMI-8226	110.62
SR	115.82
Non-Small Cell Lung Cancer	
A549/ATCC	101.81
EKVX	92.81
HOP-62	101.62
HOP-92	108.42
NCI-H226	94.51
NCI-H23	98.89
NCI-H322M	103.70
NCI-H460	100.73
NCI-H522	90.28
Colon Cancer	
COLO 205	114.44
HCC-2998	103.61
HCT-116	102.72
HCT-15	99.07
HT29	103.62
KM12	102.79
SW-620	101.24
CNS Cancer	
SF-268	93.33
SF-295	94.51
SF-539	97.26
SNB-19	100.14
SNB-75	92.76
U251	101.41
Melanoma	
LOX IMVI	94.14
MALME-3M	101.92
M14	101.01
MDA-MB-435	101.59
SK-MEL-2	100.68
SK-MEL-28	111.16
SK-MEL-5	99.50
UACC-257	100.87
UACC-62	96.74
Ovarian Cancer	
IGROV1	106.51
OVCAR-3	106.55
OVCAR-4	101.82
OVCAR-5	102.86
OVCAR-8	96.69
NCI/ADR-RES	97.67
SK-OV-3	128.59
Renal Cancer	
786-0	100.62
ACHN	103.71
CAKI-1	99.45
RXF 393	104.40
SN12C	99.12
TK-10	103.96
UO-31	83.47
Prostate Cancer	
PC-3	101.65
DU-145	103.17
Breast Cancer	
MCF7	95.27
MDA-MB-231/ATCC	99.59
HS 578T	102.45
BT-549	99.56
T-47D	99.67
MDA-MB-468	98.87
Mean	101.61
Delta	18.14
Range	45.12

Mean Growth Percent - Growth Percent

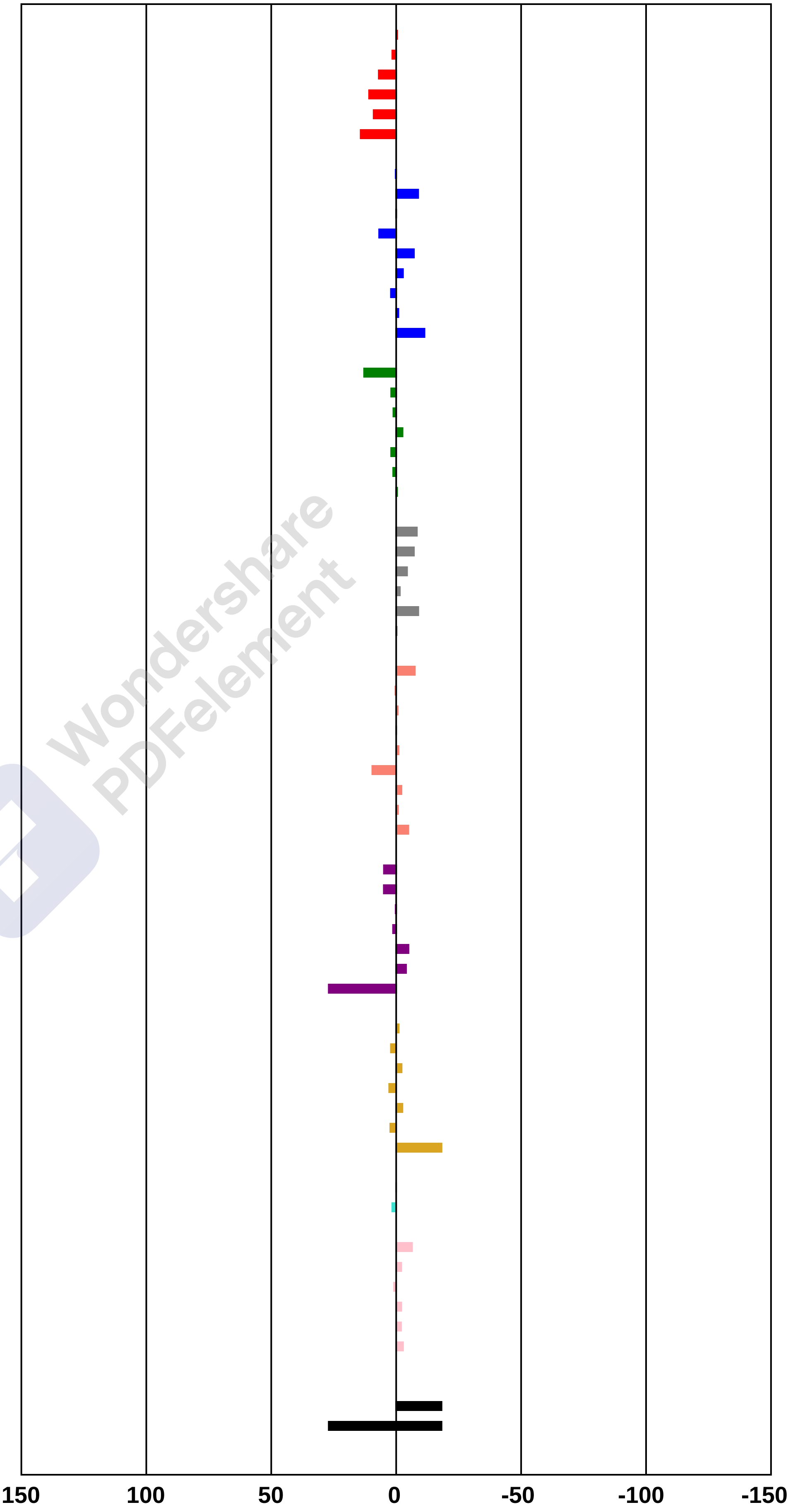

Remove Watermark Wondershare PDFelement
Wondershare PDFelement

Developmental Therapeutics Program	NSC: D-820157 / 1	Conc[illegible]
One Dose Mean Graph	Experiment ID: 1910OS87 [illegible]	[illegible]

Remove Watermark Wondershare PDFelement

Panel/Cell Line	Growth Percent
Leukemia	
CCRF-CEM	107.81
HL-60(TB)	104.71
K-562	103.86
MOLT-4	102.48
RPMI-8226	104.09
SR	103.37
Non-Small Cell Lung Cancer	
A549/ATCC	107.34
EKVX	100.09
HOP-62	103.30
HOP-92	130.03
NCI-H226	105.63
NCI-H23	104.64
NCI-H322M	105.06
NCI-H460	101.92
NCI-H522	94.19
Colon Cancer	
COLO 205	114.97
HCC-2998	113.65
HCT-116	107.24
HCT-15	99.96
HT29	104.09
KM12	102.13
SW-620	109.44
CNS Cancer	
SF-268	98.75
SF-295	93.78
SF-539	71.52
SNB-19	96.88
SNB-75	70.76
U251	87.39
Melanoma	
LOX IMVI	98.14
MALME-3M	116.65
M14	107.23
MDA-MB-435	101.59
SK-MEL-2	107.86
SK-MEL-28	109.22
SK-MEL-5	100.91
UACC-257	107.00
UACC-62	98.59
Ovarian Cancer	
IGROV1	116.77
OVCAR-3	117.86
OVCAR-4	110.14
OVCAR-5	111.33
OVCAR-8	105.93
NCI/ADR-RES	99.22
SK-OV-3	113.94
Renal Cancer	
786-0	106.68
ACHN	105.92
CAKI-1	100.67
RXF 393	108.58
SN12C	104.65
TK-10	117.79
UO-31	89.64
Prostate Cancer	
PC-3	111.12
DU-145	106.64
Breast Cancer	
MCF7	95.26
MDA-MB-231/ATCC	99.12
HS 578T	93.91
BT-549	118.81
T-47D	109.79
MDA-MB-468	105.66
Mean	104.16
Delta	33.40
Range	59.27

Mean Growth Percent - Growth Percent

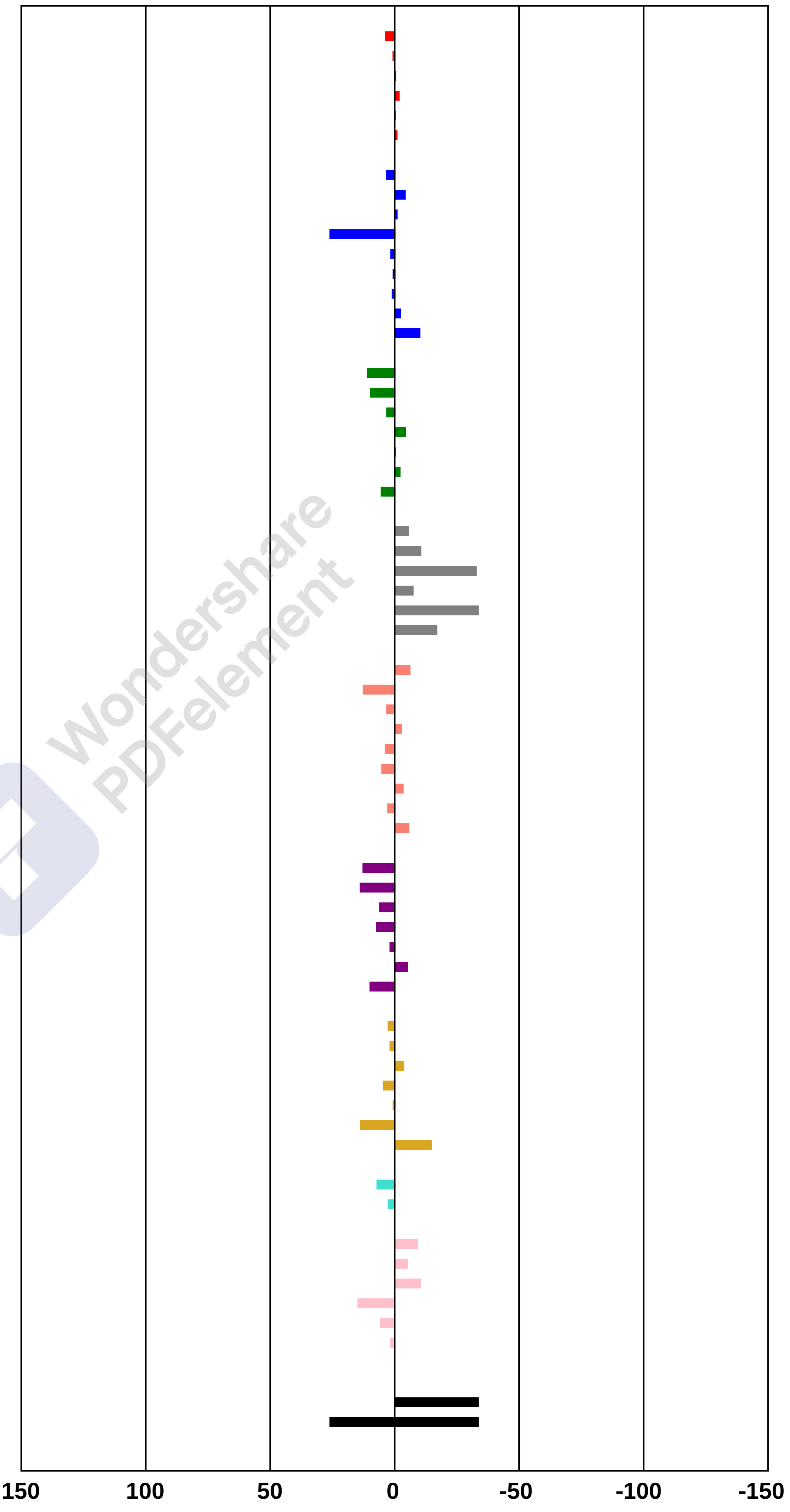

Wondershare PDFelement

Developmental Therapeutics Program

One Dose Mean Graph

NSC: D-820158 / 1 | **Conc**[illegible]

Experiment ID: 1910OS87 | Compound [illegible] | **Report Date:** [illegible]

Remove Watermark Wondershare PDFelement

Panel/Cell Line	Growth Percent
**Leukemia**	
CCRF-CEM	98.82
HL-60(TB)	105.04
K-562	103.67
MOLT-4	105.79
RPMI-8226	102.26
SR	101.07
**Non-Small Cell Lung Cancer**	
A549/ATCC	103.74
EKVX	90.76
HOP-62	101.70
HOP-92	115.40
NCI-H226	97.90
NCI-H23	100.01
NCI-H322M	103.74
NCI-H460	99.41
NCI-H522	94.36
**Colon Cancer**	
COLO 205	112.90
HCC-2998	111.56
HCT-116	107.91
HCT-15	101.22
HT29	109.85
KM12	100.15
SW-620	104.35
**CNS Cancer**	
SF-268	93.37
SF-295	91.82
SF-539	78.96
SNB-19	93.62
SNB-75	78.80
U251	80.22
**Melanoma**	
LOX IMVI	94.53
MALME-3M	112.11
M14	107.01
MDA-MB-435	103.18
SK-MEL-2	108.73
SK-MEL-28	109.72
SK-MEL-5	97.12
UACC-257	108.33
UACC-62	99.62
**Ovarian Cancer**	
IGROV1	102.29
OVCAR-3	111.60
OVCAR-4	105.02
OVCAR-5	104.48
OVCAR-8	102.23
NCI/ADR-RES	103.18
SK-OV-3	115.32
**Renal Cancer**	
786-0	115.64
ACHN	101.74
CAKI-1	99.31
RXF 393	103.88
SN12C	100.06
TK-10	140.42
UO-31	87.09
**Prostate Cancer**	
PC-3	100.24
DU-145	105.34
**Breast Cancer**	
MCF7	89.23
MDA-MB-231/ATCC	105.25
HS 578T	93.51
BT-549	137.18
T-47D	101.45
MDA-MB-468	97.16
Mean	102.55
Delta	23.75
Range	61.62

Mean Growth Percent - Growth Percent

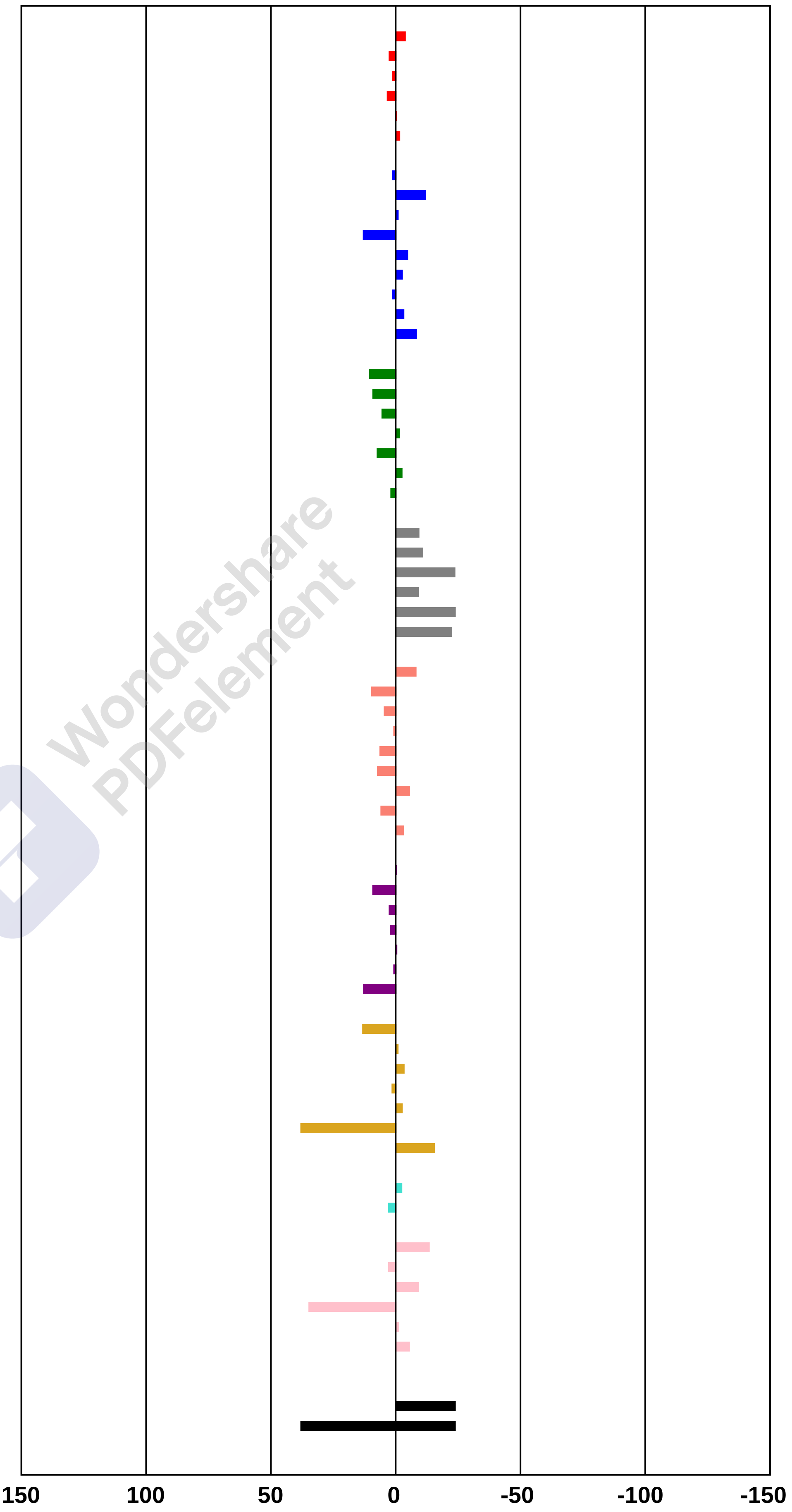

Wondershare PDFelement

Developmental Therapeutics Program

One Dose Mean Graph

NSC: D-820159 / 1 | **Conc** [illegible]
Experiment ID: 1910OS87 | Compound 7c | **Report Date:** Nov 25, 2019

Remove Watermark Wondershare PDFelement

Panel/Cell Line	Growth Percent
**Leukemia**	
CCRF-CEM	101.60
HL-60(TB)	102.08
K-562	91.72
MOLT-4	88.24
RPMI-8226	93.27
SR	94.07
**Non-Small Cell Lung Cancer**	
A549/ATCC	100.47
EKVX	95.77
HOP-62	93.71
HOP-92	119.39
NCI-H226	99.29
NCI-H23	88.92
NCI-H322M	99.69
NCI-H460	101.73
NCI-H522	88.38
**Colon Cancer**	
COLO 205	103.35
HCC-2998	112.66
HCT-116	97.94
HCT-15	97.77
HT29	97.28
KM12	66.34
SW-620	108.79
**CNS Cancer**	
SF-268	89.20
SF-295	95.16
SF-539	61.49
SNB-19	90.81
SNB-75	54.04
U251	73.13
**Melanoma**	
LOX IMVI	90.99
MALME-3M	105.06
M14	102.43
MDA-MB-435	100.67
SK-MEL-2	108.54
SK-MEL-28	108.23
SK-MEL-5	99.67
UACC-257	106.33
UACC-62	96.40
**Ovarian Cancer**	
IGROV1	97.68
OVCAR-3	104.27
OVCAR-4	103.45
OVCAR-5	114.33
OVCAR-8	100.41
NCI/ADR-RES	97.66
SK-OV-3	113.07
**Renal Cancer**	
786-0	108.43
ACHN	94.84
CAKI-1	90.57
RXF 393	106.61
SN12C	97.89
TK-10	116.24
UO-31	75.79
**Prostate Cancer**	
PC-3	91.19
DU-145	104.51
**Breast Cancer**	
MCF7	88.42
MDA-MB-231/ATCC	95.26
HS 578T	90.32
BT-549	115.17
T-47D	96.39
MDA-MB-468	109.16
Mean	97.22
Delta	43.18
Range	65.35

Mean Growth Percent - Growth Percent

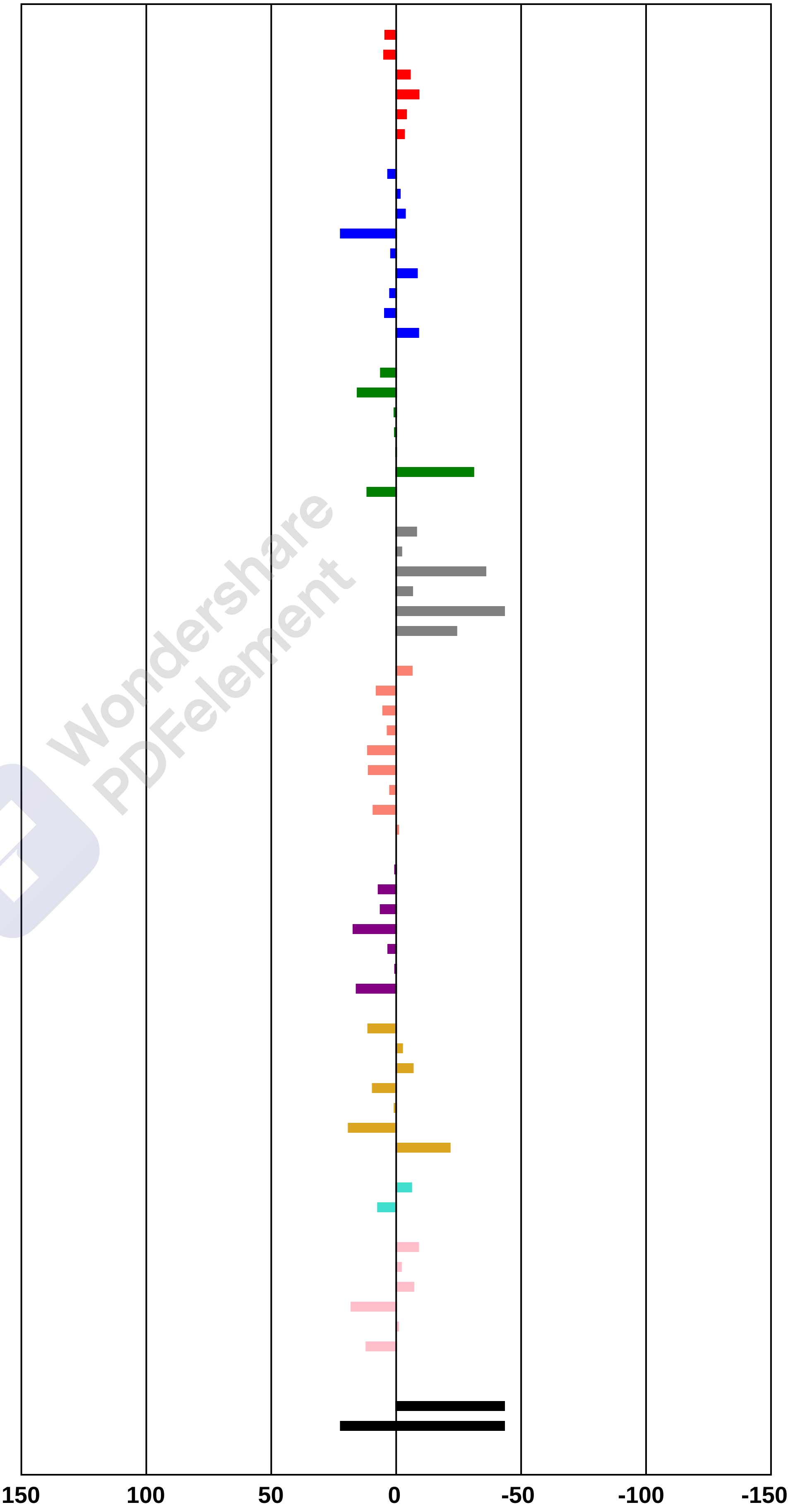

Wondershare PDFelement

Developmental Therapeutics Program

One Dose Mean Graph

NSC: D-820175 / 1	Conc[illegible]	
Experiment ID: 1910OS87	Compound 13a	Report Date: [illegible]

Remove Watermark Wondershare PDFelement

Panel/Cell Line	Growth Percent
Leukemia	
CCRF-CEM	110.72
HL-60(TB)	101.27
K-562	101.00
MOLT-4	104.07
RPMI-8226	112.17
SR	106.56
Non-Small Cell Lung Cancer	
A549/ATCC	99.51
EKVX	98.44
HOP-62	104.20
HOP-92	118.26
NCI-H226	99.31
NCI-H23	92.27
NCI-H322M	99.43
NCI-H460	102.72
NCI-H522	94.42
Colon Cancer	
COLO 205	112.81
HCC-2998	100.92
HCT-116	103.95
HCT-15	102.15
HT29	103.93
KM12	101.12
SW-620	103.66
CNS Cancer	
SF-268	93.90
SF-295	103.70
SF-539	102.52
SNB-19	98.99
SNB-75	81.08
U251	96.72
Melanoma	
LOX IMVI	97.58
MALME-3M	109.71
M14	106.58
MDA-MB-435	101.54
SK-MEL-2	115.88
SK-MEL-28	107.83
SK-MEL-5	101.54
UACC-257	112.47
UACC-62	94.26
Ovarian Cancer	
IGROV1	97.91
OVCAR-3	106.13
OVCAR-4	111.94
OVCAR-5	107.01
OVCAR-8	103.64
NCI/ADR-RES	102.67
SK-OV-3	124.53
Renal Cancer	
786-0	102.13
ACHN	99.11
CAKI-1	92.95
RXF 393	101.28
SN12C	102.01
TK-10	113.84
UO-31	80.64
Prostate Cancer	
PC-3	104.34
DU-145	109.53
Breast Cancer	
MCF7	95.25
MDA-MB-231/ATCC	102.49
HS 578T	106.47
BT-549	113.56
T-47D	108.35
MDA-MB-468	101.31
Mean	103.12
Delta	22.48
Range	43.89

Mean Growth Percent - Growth Percent

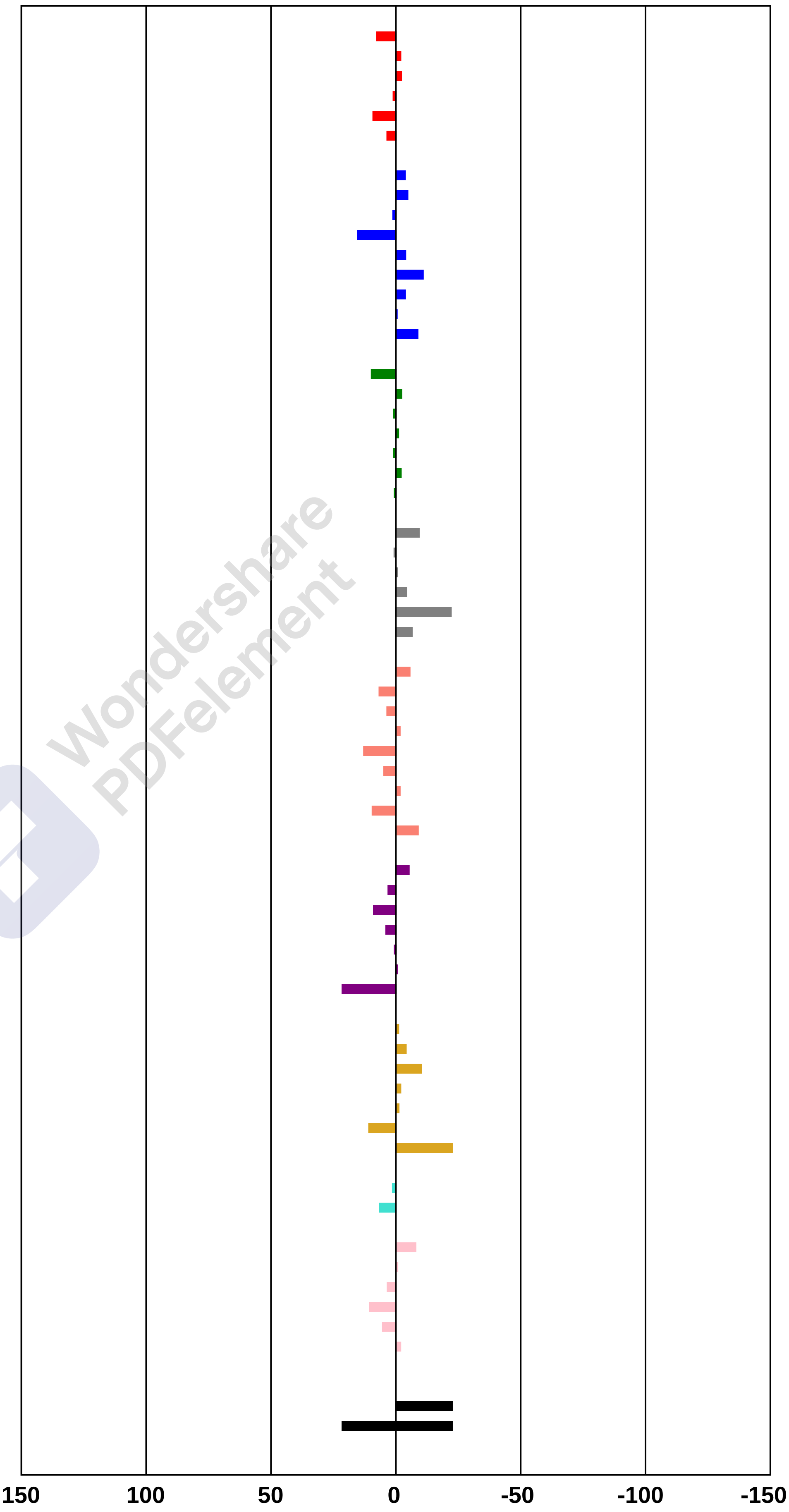

Wondershare PDFelement

Developmental Therapeutics Program

One Dose Mean Graph

NSC: D-820160 / 1 | **Conc** [illegible]

Experiment ID: 1910OS87 | Compound 20c | **Report Date:** [illegible]

Remove Watermark Wondershare PDFelement

Panel/Cell Line	Growth Percent
**Leukemia**	
CCRF-CEM	108.09
HL-60(TB)	103.41
K-562	108.00
MOLT-4	108.77
RPMI-8226	103.64
SR	102.62
**Non-Small Cell Lung Cancer**	
A549/ATCC	104.46
EKVX	90.52
HOP-62	102.52
HOP-92	111.83
NCI-H226	92.02
NCI-H23	94.98
NCI-H322M	101.35
NCI-H460	101.95
NCI-H522	91.22
**Colon Cancer**	
COLO 205	117.81
HCC-2998	102.48
HCT-116	108.21
HCT-15	99.06
HT29	108.21
KM12	101.78
SW-620	102.37
**CNS Cancer**	
SF-268	97.29
SF-295	101.19
SF-539	95.36
SNB-19	99.11
SNB-75	79.17
U251	103.12
**Melanoma**	
LOX IMVI	99.24
MALME-3M	112.53
M14	107.62
MDA-MB-435	103.83
SK-MEL-2	106.25
SK-MEL-28	112.39
SK-MEL-5	100.00
UACC-257	112.86
UACC-62	98.83
**Ovarian Cancer**	
IGROV1	81.14
OVCAR-3	106.57
OVCAR-4	109.40
OVCAR-5	109.86
OVCAR-8	105.50
NCI/ADR-RES	100.58
SK-OV-3	112.92
**Renal Cancer**	
786-0	83.06
ACHN	105.18
CAKI-1	93.08
RXF 393	109.55
SN12C	100.04
TK-10	152.23
UO-31	81.51
**Prostate Cancer**	
PC-3	101.49
DU-145	116.86
**Breast Cancer**	
MCF7	91.44
MDA-MB-231/ATCC	95.46
HS 578T	99.45
BT-549	116.50
T-47D	105.24
MDA-MB-468	110.91
Mean	103.09
Delta	23.92
Range	73.06

Mean Growth Percent - Growth Percent

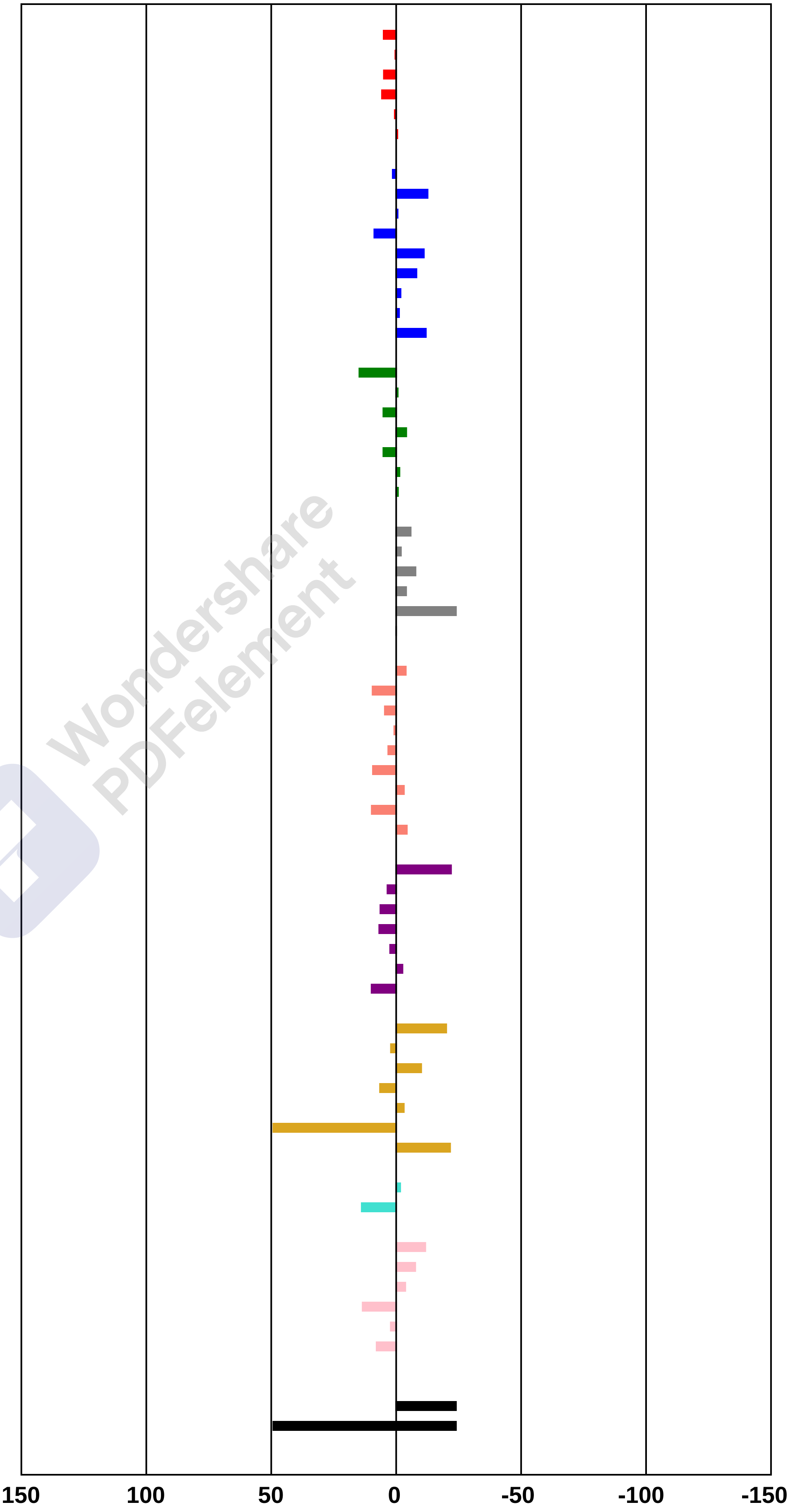

Wondershare PDFelement

Remove Watermark Wondershare PDFelement

Developmental Therapeutics Program
One Dose Mean Graph

NSC: D-820161 / 1 | **Conc** [illegible]

Experiment ID: 1910OS87 Compound 19c | **Report Date:** [illegible]

Panel/Cell Line	Growth Percent
Leukemia	
CCRF-CEM	104.72
HL-60(TB)	104.56
K-562	106.41
MOLT-4	108.67
RPMI-8226	105.48
SR	101.35
Non-Small Cell Lung Cancer	
A549/ATCC	107.54
EKVX	98.49
HOP-62	105.87
HOP-92	124.71
NCI-H226	106.83
NCI-H23	102.70
NCI-H322M	105.79
NCI-H460	100.45
NCI-H522	90.06
Colon Cancer	
COLO 205	115.76
HCC-2998	105.39
HCT-116	102.16
HCT-15	99.76
HT29	102.22
KM12	102.09
SW-620	102.53
CNS Cancer	
SF-268	97.68
SF-295	99.97
SF-539	99.00
SNB-19	103.73
SNB-75	91.75
U251	104.84
Melanoma	
LOX IMVI	98.82
MALME-3M	104.57
M14	107.96
MDA-MB-435	102.91
SK-MEL-2	101.81
SK-MEL-28	112.10
SK-MEL-5	102.44
UACC-257	115.40
UACC-62	101.73
Ovarian Cancer	
IGROV1	105.45
OVCAR-3	110.31
OVCAR-4	112.39
OVCAR-5	110.34
OVCAR-8	104.13
NCI/ADR-RES	105.48
SK-OV-3	120.62
Renal Cancer	
786-0	104.99
ACHN	106.82
CAKI-1	90.48
RXF 393	109.21
SN12C	101.77
TK-10	115.96
UO-31	79.99
Prostate Cancer	
PC-3	110.47
DU-145	114.20
Breast Cancer	
MCF7	93.40
MDA-MB-231/ATCC	104.18
HS 578T	105.84
BT-549	103.54
T-47D	106.95
MDA-MB-468	119.73
Mean	104.82
Delta	24.83
Range	44.72

Mean Growth Percent - Growth Percent

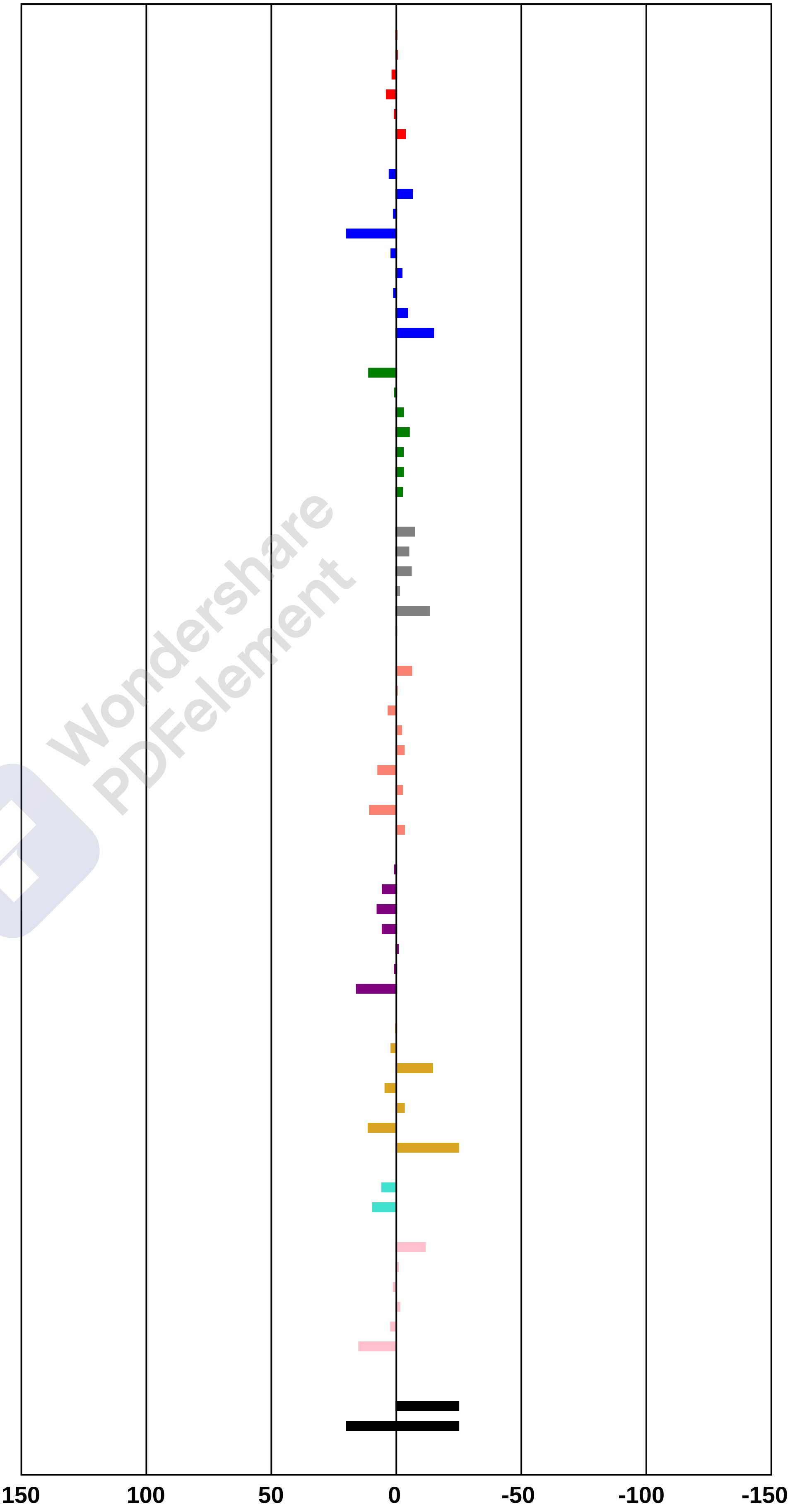

Wondershare PDFelement

Developmental Therapeutics Program

One Dose Mean Graph

NSC: D-820162 / 1 | **Conc**[illegible]

Experiment ID: 1910OS87 | Compound 15c | **Report Date:** [illegible]

Remove Watermark Wondershare PDFelement

Panel/Cell Line	Growth Percent
**Leukemia**	
CCRF-CEM	103.44
HL-60(TB)	104.93
K-562	105.00
MOLT-4	102.36
RPMI-8226	104.38
SR	102.79
**Non-Small Cell Lung Cancer**	
A549/ATCC	105.88
EKVX	79.43
HOP-62	92.81
HOP-92	101.51
NCI-H226	85.66
NCI-H23	91.83
NCI-H322M	97.24
NCI-H460	98.93
NCI-H522	74.57
**Colon Cancer**	
COLO 205	116.75
HCC-2998	115.42
HCT-116	100.92
HCT-15	101.72
HT29	101.84
KM12	100.92
SW-620	102.65
**CNS Cancer**	
SF-268	94.84
SF-295	102.79
SF-539	104.00
SNB-19	94.85
SNB-75	79.59
U251	100.96
**Melanoma**	
LOX IMVI	95.88
MALME-3M	102.31
M14	107.86
MDA-MB-435	102.42
SK-MEL-2	94.54
SK-MEL-28	110.64
SK-MEL-5	96.96
UACC-257	110.09
UACC-62	92.45
**Ovarian Cancer**	
IGROV1	72.73
OVCAR-3	98.94
OVCAR-4	106.34
OVCAR-5	107.61
OVCAR-8	102.02
NCI/ADR-RES	102.16
SK-OV-3	93.42
**Renal Cancer**	
786-0	108.78
ACHN	97.37
CAKI-1	86.45
RXF 393	106.75
SN12C	98.63
TK-10	154.58
UO-31	71.20
**Prostate Cancer**	
PC-3	97.64
DU-145	108.86
**Breast Cancer**	
MCF7	88.60
MDA-MB-231/ATCC	88.18
HS 578T	102.54
BT-549	116.44
T-47D	90.65
MDA-MB-468	102.91
Mean	99.73
Delta	28.53
Range	83.38

Mean Growth Percent - Growth Percent

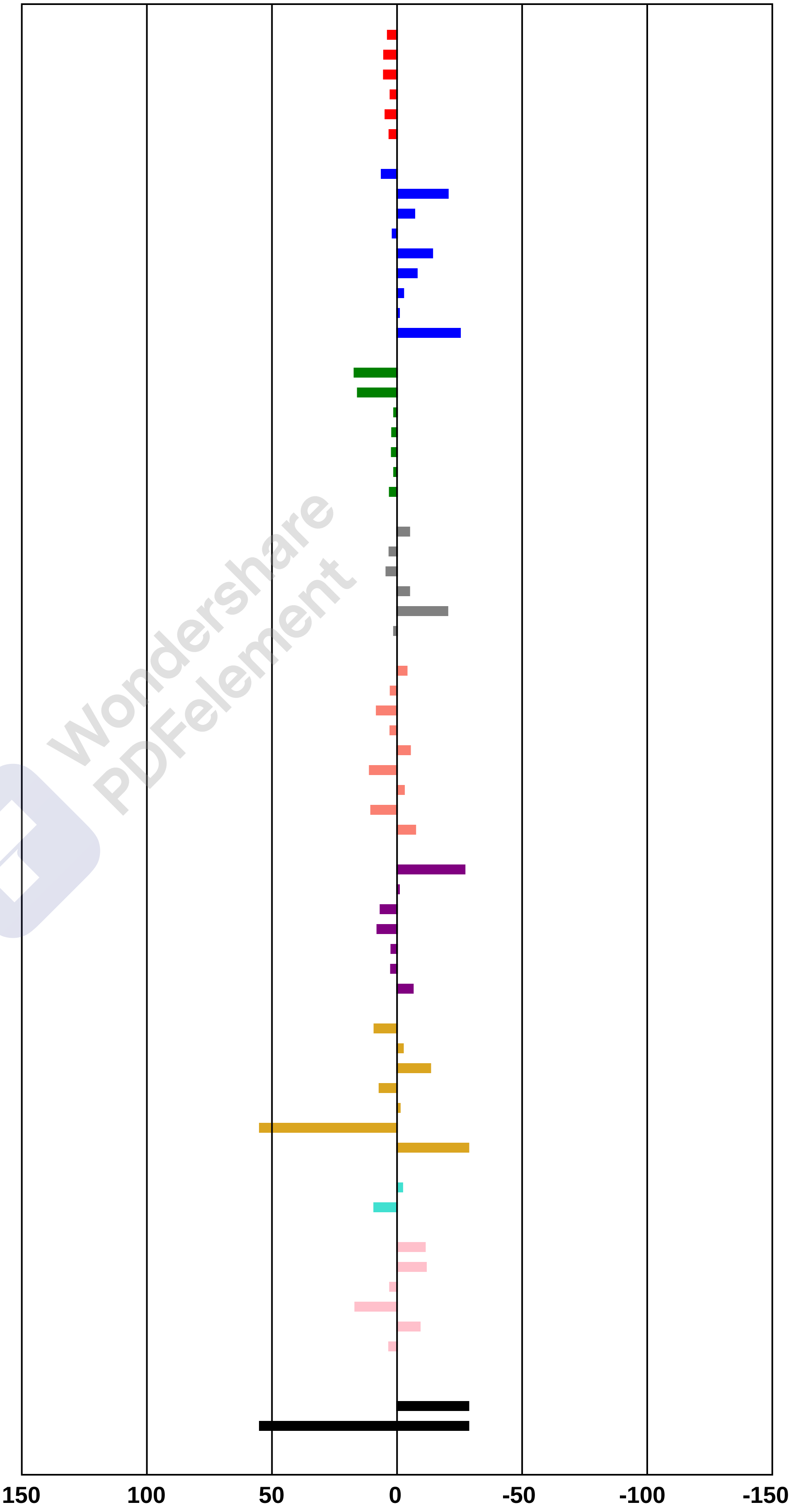

Wondershare PDFelement

Developmental Therapeutics Program

One Dose Mean Graph

NSC: D-820163 / 1 | **Conc** [illegible]

Experiment ID: 1910OS87 | Compound 20d | **Report Date:** Nov [illegible], 2019

Remove Watermark Wondershare PDFelement

Panel/Cell Line	Growth Percent
**Leukemia**	
CCRF-CEM	107.95
HL-60(TB)	104.62
K-562	117.47
MOLT-4	109.84
RPMI-8226	108.75
SR	110.02
**Non-Small Cell Lung Cancer**	
A549/ATCC	94.08
EKVX	94.41
HOP-62	105.66
HOP-92	118.75
NCI-H226	88.71
NCI-H23	98.94
NCI-H322M	105.25
NCI-H460	101.82
NCI-H522	92.67
**Colon Cancer**	
COLO 205	119.14
HCC-2998	104.19
HCT-116	105.97
HCT-15	99.67
HT29	107.13
KM12	101.54
SW-620	103.17
**CNS Cancer**	
SF-268	95.20
SF-295	101.90
SF-539	100.82
SNB-19	98.16
SNB-75	90.45
U251	99.18
**Melanoma**	
LOX IMVI	96.30
MALME-3M	115.88
M14	102.60
MDA-MB-435	101.67
SK-MEL-2	113.22
SK-MEL-28	111.42
SK-MEL-5	100.45
UACC-257	103.00
UACC-62	93.74
**Ovarian Cancer**	
IGROV1	98.94
OVCAR-3	108.42
OVCAR-4	104.88
OVCAR-5	100.15
OVCAR-8	99.61
NCI/ADR-RES	104.13
SK-OV-3	122.66
**Renal Cancer**	
786-0	104.67
ACHN	102.28
CAKI-1	92.19
RXF 393	102.57
SN12C	103.55
TK-10	124.14
UO-31	76.32
**Prostate Cancer**	
PC-3	105.36
DU-145	109.99
**Breast Cancer**	
MCF7	91.48
MDA-MB-231/ATCC	99.77
HS 578T	99.99
BT-549	117.96
T-47D	112.64
MDA-MB-468	83.88
Mean	103.21
Delta	26.89
Range	47.82

Mean Growth Percent - Growth Percent

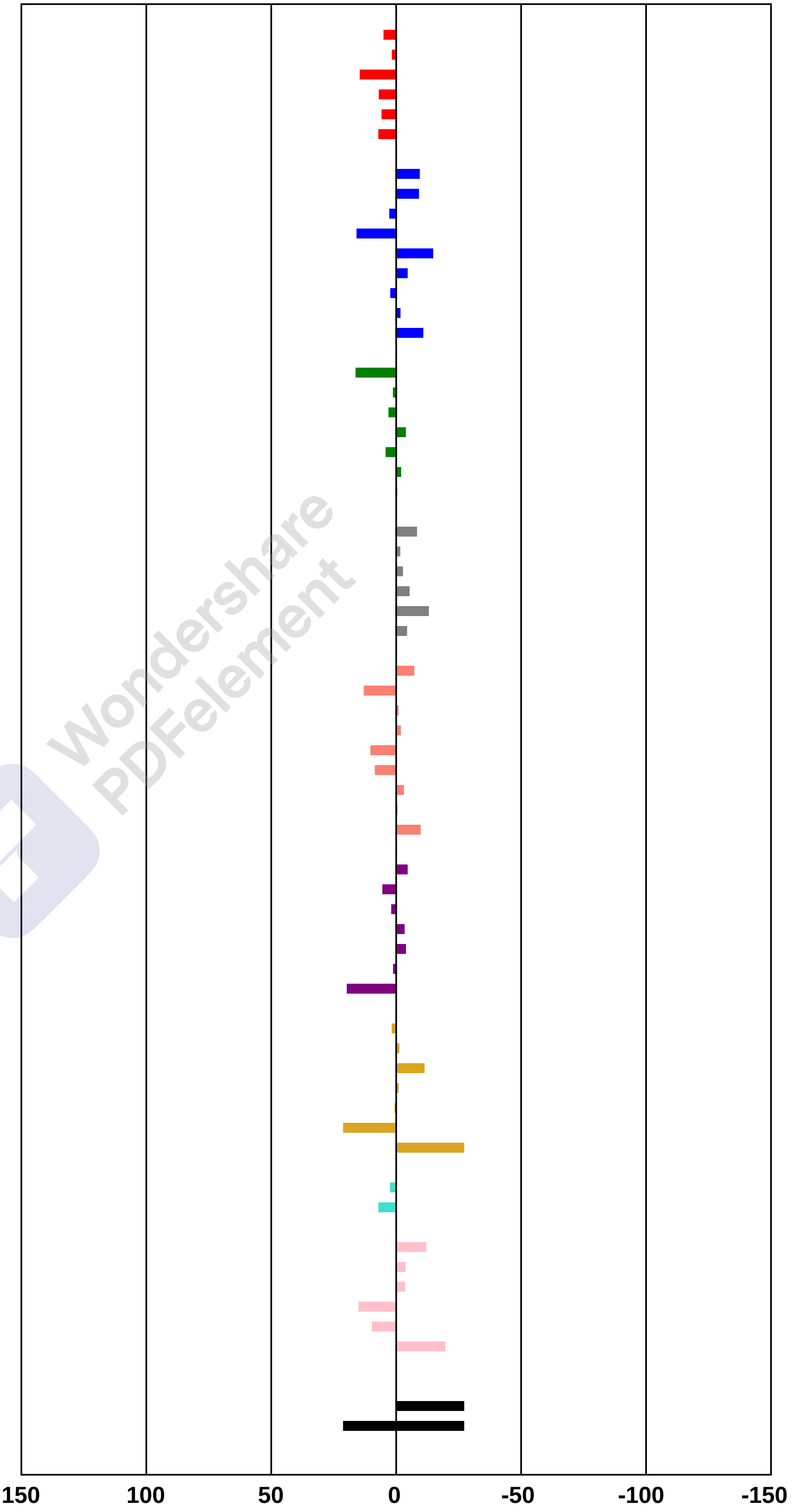

Wondershare PDFelement

Developmental Therapeutics Program

One Dose Mean Graph

NSC: D-820164 / 1 | **Conc** [illegible]

Experiment ID: 1910OS87 | Compound 19d | **Report Date:** Nov 23, 2019

Remove Watermark Wondershare PDFelement

Panel/Cell Line	Growth Percent
**Leukemia**	
CCRF-CEM	105.23
HL-60(TB)	102.80
K-562	113.30
MOLT-4	115.32
RPMI-8226	110.01
SR	110.82
**Non-Small Cell Lung Cancer**	
A549/ATCC	104.35
EKVX	99.43
HOP-62	104.52
HOP-92	117.77
NCI-H226	104.06
NCI-H23	100.60
NCI-H322M	108.11
NCI-H460	100.44
NCI-H522	95.56
**Colon Cancer**	
COLO 205	117.34
HCC-2998	104.72
HCT-116	107.30
HCT-15	101.58
HT29	104.10
KM12	103.84
SW-620	101.50
**CNS Cancer**	
SF-268	96.48
SF-295	96.07
SF-539	98.02
SNB-19	98.91
SNB-75	97.50
U251	102.27
**Melanoma**	
LOX IMVI	98.84
MALME-3M	112.63
M14	104.71
MDA-MB-435	101.82
SK-MEL-2	100.61
SK-MEL-28	111.54
SK-MEL-5	101.85
UACC-257	99.74
UACC-62	102.36
**Ovarian Cancer**	
IGROV1	106.28
OVCAR-3	106.50
OVCAR-4	108.82
OVCAR-5	108.42
OVCAR-8	101.35
NCI/ADR-RES	101.46
SK-OV-3	129.24
**Renal Cancer**	
786-0	105.34
ACHN	102.14
CAKI-1	96.62
RXF 393	102.06
SN12C	102.77
TK-10	107.20
UO-31	84.81
**Prostate Cancer**	
PC-3	103.52
DU-145	111.26
**Breast Cancer**	
MCF7	95.18
MDA-MB-231/ATCC	102.53
HS 578T	103.12
BT-549	113.96
T-47D	106.56
MDA-MB-468	102.77
Mean	104.37
Delta	19.56
Range	44.43

Mean Growth Percent - Growth Percent

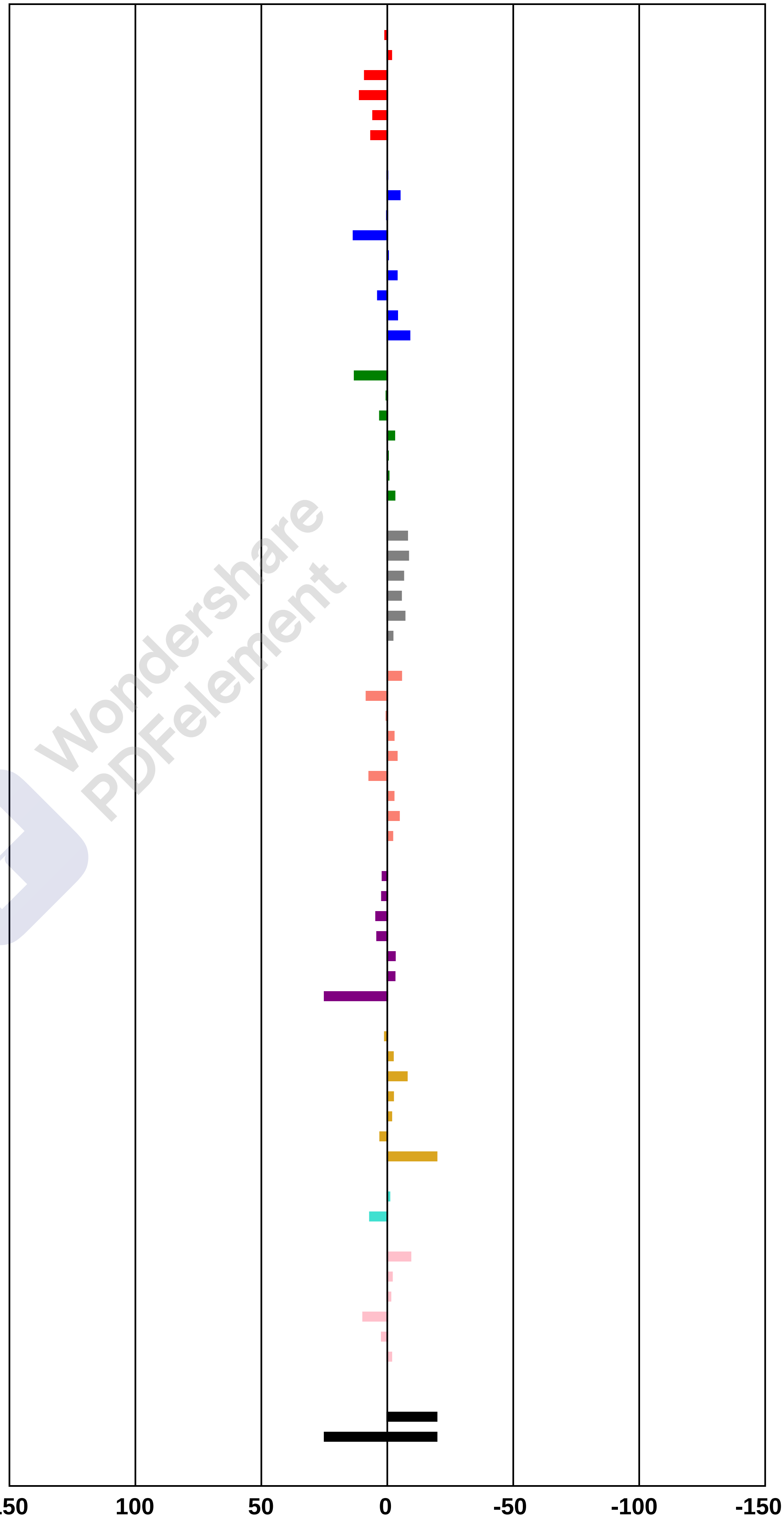

Wondershare PDFelement

Developmental Therapeutics Program

One Dose Mean Graph

NSC: D-820165 / 1 | Conc [illegible]

Experiment ID: 1910OS87 | Compound 15d | Report Date: [illegible]

Remove Watermark Wondershare PDFelement

Panel/Cell Line	Growth Percent
**Leukemia**	
HL-60(TB)	101.99
K-562	105.38
MOLT-4	110.03
SR	101.45
**Non-Small Cell Lung Cancer**	
A549/ATCC	100.72
EKVX	88.08
HOP-62	99.30
HOP-92	120.52
NCI-H226	103.24
NCI-H23	95.72
NCI-H322M	107.66
NCI-H460	101.54
NCI-H522	88.16
**Colon Cancer**	
COLO 205	112.34
HCC-2998	102.42
HCT-116	106.80
HCT-15	96.09
HT29	102.54
KM12	102.31
SW-620	108.22
**CNS Cancer**	
SF-268	96.50
SF-295	93.74
SF-539	98.57
SNB-19	91.80
SNB-75	99.20
U251	98.20
**Melanoma**	
LOX IMVI	101.32
MALME-3M	116.15
M14	110.24
MDA-MB-435	101.03
SK-MEL-2	100.90
SK-MEL-28	109.62
SK-MEL-5	100.59
UACC-257	109.91
UACC-62	88.53
**Ovarian Cancer**	
IGROV1	103.95
OVCAR-3	108.52
OVCAR-4	102.21
OVCAR-5	101.35
OVCAR-8	105.01
NCI/ADR-RES	99.61
SK-OV-3	114.45
**Renal Cancer**	
786-0	94.41
ACHN	97.62
CAKI-1	90.42
RXF 393	100.37
SN12C	100.14
TK-10	118.53
UO-31	77.07
**Prostate Cancer**	
PC-3	110.42
DU-145	101.96
**Breast Cancer**	
MCF7	93.88
MDA-MB-231/ATCC	97.25
HS 578T	102.42
BT-549	114.08
T-47D	95.46
MDA-MB-468	85.68
Mean	101.50
Delta	24.43
Range	43.45

Mean Growth Percent - Growth Percent

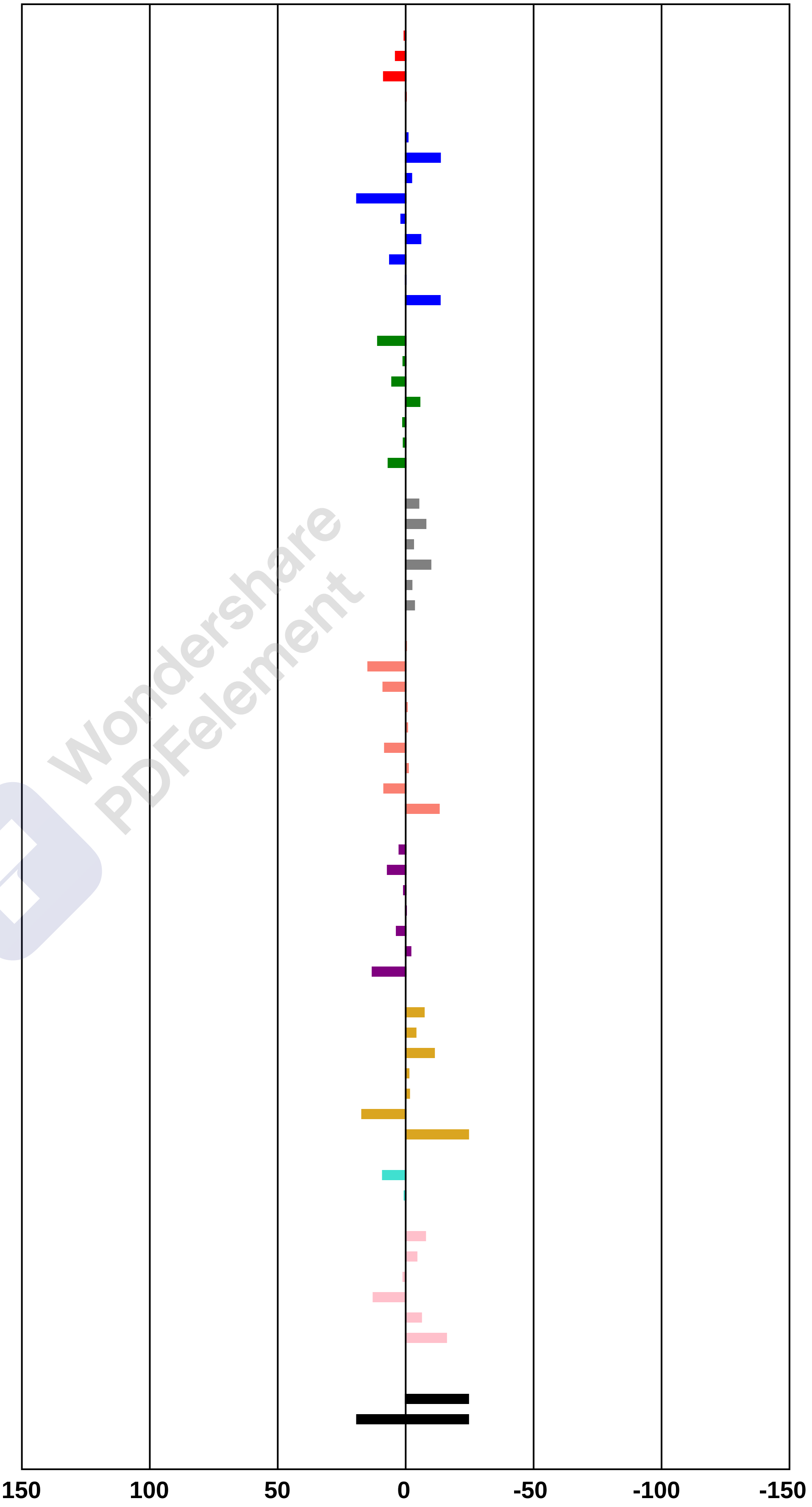

Wondershare PDFelement

Developmental Therapeutics Program

One Dose Mean Graph

NSC: D-820167 / 1 | **Conc:** [illegible]

Experiment ID: 1910OS87 | Compound 12a | **Report Date:** Nov 25, 2019

Panel/Cell Line	Growth Percent
**Leukemia**	
CCRF-CEM	107.09
HL-60(TB)	101.44
K-562	97.83
MOLT-4	102.68
RPMI-8226	110.53
SR	105.35
**Non-Small Cell Lung Cancer**	
A549/ATCC	99.69
EKVX	93.22
HOP-62	100.08
HOP-92	121.76
NCI-H226	93.35
NCI-H23	89.25
NCI-H322M	99.76
NCI-H460	102.61
NCI-H522	87.71
**Colon Cancer**	
COLO 205	111.95
HCC-2998	102.15
HCT-116	99.50
HCT-15	100.20
HT29	104.74
KM12	99.61
SW-620	103.39
**CNS Cancer**	
SF-268	92.51
SF-295	102.06
SF-539	96.54
SNB-19	97.12
SNB-75	78.17
U251	92.32
**Melanoma**	
LOX IMVI	93.57
MALME-3M	104.57
M14	99.09
MDA-MB-435	101.82
SK-MEL-2	113.39
SK-MEL-28	109.43
SK-MEL-5	97.81
UACC-257	104.23
UACC-62	99.19
**Ovarian Cancer**	
IGROV1	99.07
OVCAR-3	107.19
OVCAR-4	100.60
OVCAR-5	105.14
OVCAR-8	104.68
NCI/ADR-RES	103.79
SK-OV-3	121.69
**Renal Cancer**	
786-0	102.37
ACHN	95.45
CAKI-1	92.88
RXF 393	100.13
SN12C	99.09
TK-10	111.37
UO-31	75.33
**Prostate Cancer**	
PC-3	98.99
DU-145	108.51
**Breast Cancer**	
MCF7	92.41
MDA-MB-231/ATCC	92.16
HS 578T	106.34
BT-549	105.06
T-47D	103.98
MDA-MB-468	100.06
Mean	100.71
Delta	25.38
Range	46.43

Mean Growth Percent - Growth Percent

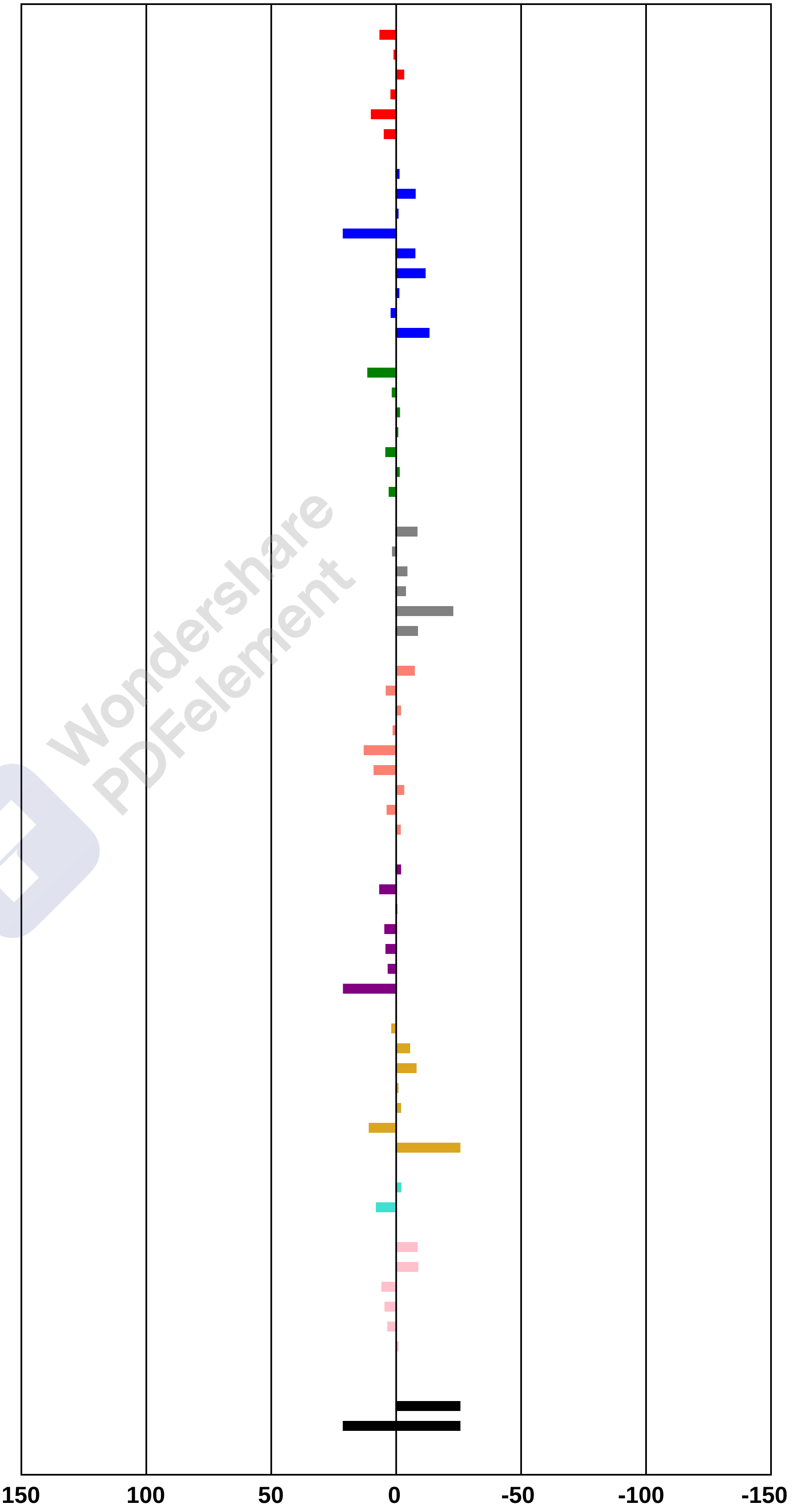

Remove Watermark
Wondershare PDFelement
Wondershare PDFelement

Developmental Therapeutics Program	NSC: D-820168 / 1	Conc[illegible]	[illegible]
One Dose Mean Graph	Experiment ID: 1910OS87	Compound 11a	Report Date: [illegible]

Remove Watermark Wondershare PDFelement

Panel/Cell Line	Growth Percent
**Leukemia**	
CCRF-CEM	106.84
HL-60(TB)	103.58
K-562	108.34
MOLT-4	110.52
RPMI-8226	109.40
SR	108.77
**Non-Small Cell Lung Cancer**	
A549/ATCC	97.77
EKVX	94.62
HOP-62	102.39
HOP-92	106.72
NCI-H226	90.42
NCI-H23	96.12
NCI-H322M	101.26
NCI-H460	101.81
NCI-H522	92.80
**Colon Cancer**	
COLO 205	113.57
HCC-2998	98.02
HCT-116	102.94
HCT-15	95.77
HT29	104.03
KM12	103.23
SW-620	103.02
**CNS Cancer**	
SF-268	96.85
SF-295	95.29
SF-539	90.31
SNB-19	97.59
SNB-75	79.72
U251	96.93
**Melanoma**	
LOX IMVI	95.38
MALME-3M	103.62
M14	99.16
MDA-MB-435	102.21
SK-MEL-2	107.07
SK-MEL-28	105.11
SK-MEL-5	99.18
UACC-257	101.08
UACC-62	98.79
**Ovarian Cancer**	
IGROV1	103.03
OVCAR-3	102.61
OVCAR-4	101.41
OVCAR-5	106.26
OVCAR-8	101.03
NCI/ADR-RES	99.07
SK-OV-3	123.79
**Renal Cancer**	
786-0	98.21
ACHN	101.68
CAKI-1	94.51
RXF 393	107.33
SN12C	101.42
TK-10	97.69
UO-31	83.77
**Prostate Cancer**	
PC-3	101.73
DU-145	110.81
**Breast Cancer**	
MCF7	92.30
MDA-MB-231/ATCC	97.67
HS 578T	99.62
BT-549	104.90
T-47D	107.73
MDA-MB-468	101.31
Mean	100.98
Delta	21.26
Range	44.07

Mean Growth Percent - Growth Percent

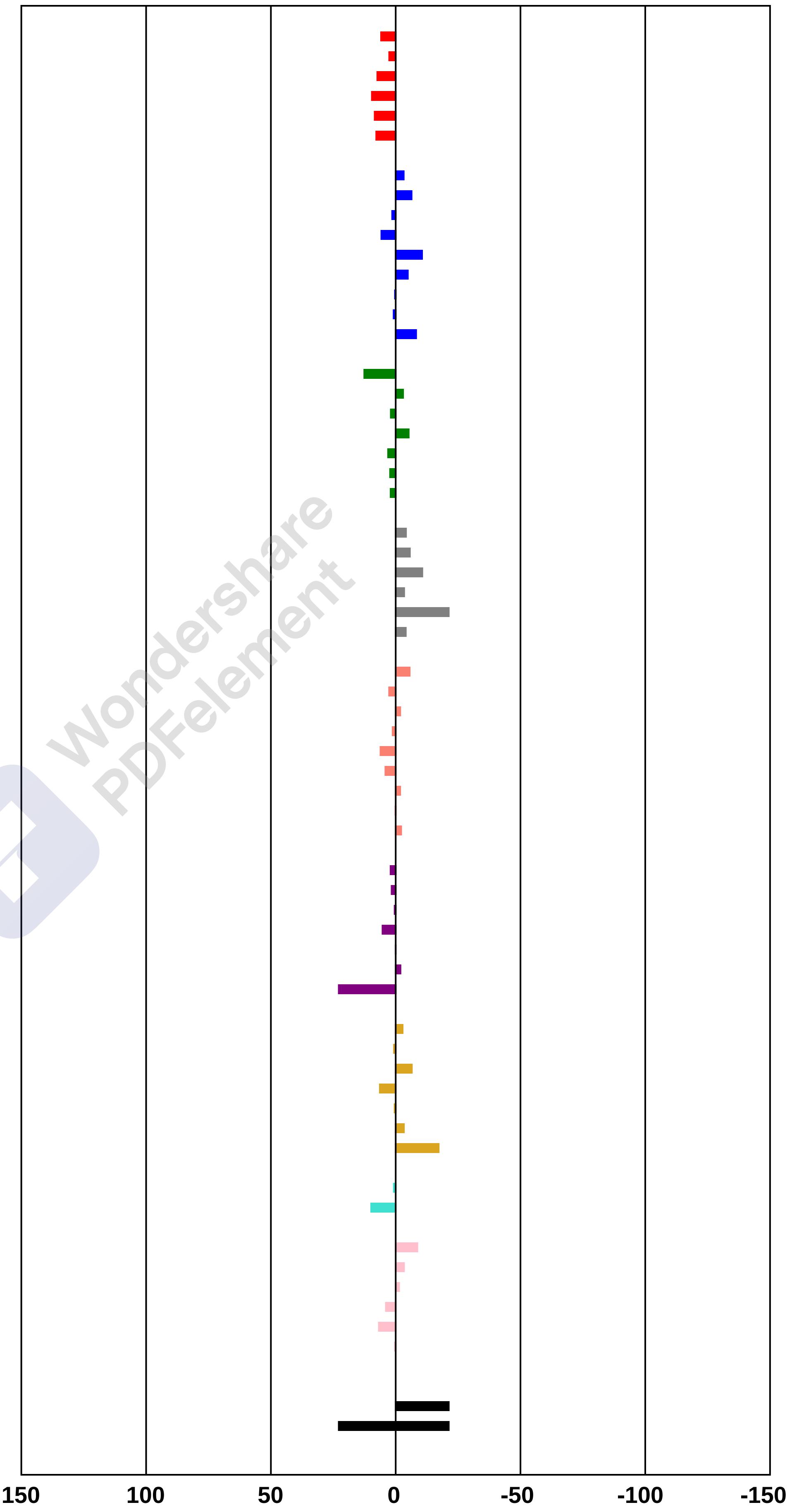

Wondershare PDFelement

CompoundCompound

Developmental Therapeutics Program

One Dose Mean Graph

NSC: D-820169 / 1 | **Conc** [illegible]

Experiment ID: 1910OS87 | Compound 7a | Report Date: [illegible]

Remove Watermark Wondershare PDFelement

Panel/Cell Line	Growth Percent
**Leukemia**	
CCRF-CEM	97.44
HL-60(TB)	102.25
K-562	105.92
MOLT-4	103.94
RPMI-8226	105.45
SR	99.16
**Non-Small Cell Lung Cancer**	
A549/ATCC	103.82
EKVX	88.95
HOP-62	102.62
HOP-92	117.36
NCI-H226	99.47
NCI-H23	101.19
NCI-H322M	98.47
NCI-H460	100.39
NCI-H522	92.04
**Colon Cancer**	
COLO 205	106.84
HCC-2998	99.38
HCT-116	93.86
HCT-15	99.16
HT29	94.78
KM12	94.85
SW-620	98.44
**CNS Cancer**	
SF-268	91.70
SF-295	96.23
SF-539	96.00
SNB-19	100.20
SNB-75	75.56
U251	89.34
**Melanoma**	
LOX IMVI	90.57
MALME-3M	89.51
M14	95.18
MDA-MB-435	102.09
SK-MEL-2	107.80
SK-MEL-28	104.39
SK-MEL-5	101.07
UACC-257	115.56
UACC-62	101.73
**Ovarian Cancer**	
IGROV1	103.69
OVCAR-3	105.48
OVCAR-4	94.11
OVCAR-5	105.35
OVCAR-8	106.33
NCI/ADR-RES	102.16
SK-OV-3	122.74
**Renal Cancer**	
786-0	95.93
ACHN	102.35
CAKI-1	91.67
RXF 393	110.20
SN12C	102.86
TK-10	108.59
UO-31	74.65
**Prostate Cancer**	
PC-3	97.34
DU-145	100.26
**Breast Cancer**	
MCF7	90.78
MDA-MB-231/ATCC	91.12
HS 578T	97.71
BT-549	87.60
T-47D	95.72
MDA-MB-468	111.92
Mean	99.34
Delta	24.69
Range	48.09

Mean Growth Percent - Growth Percent

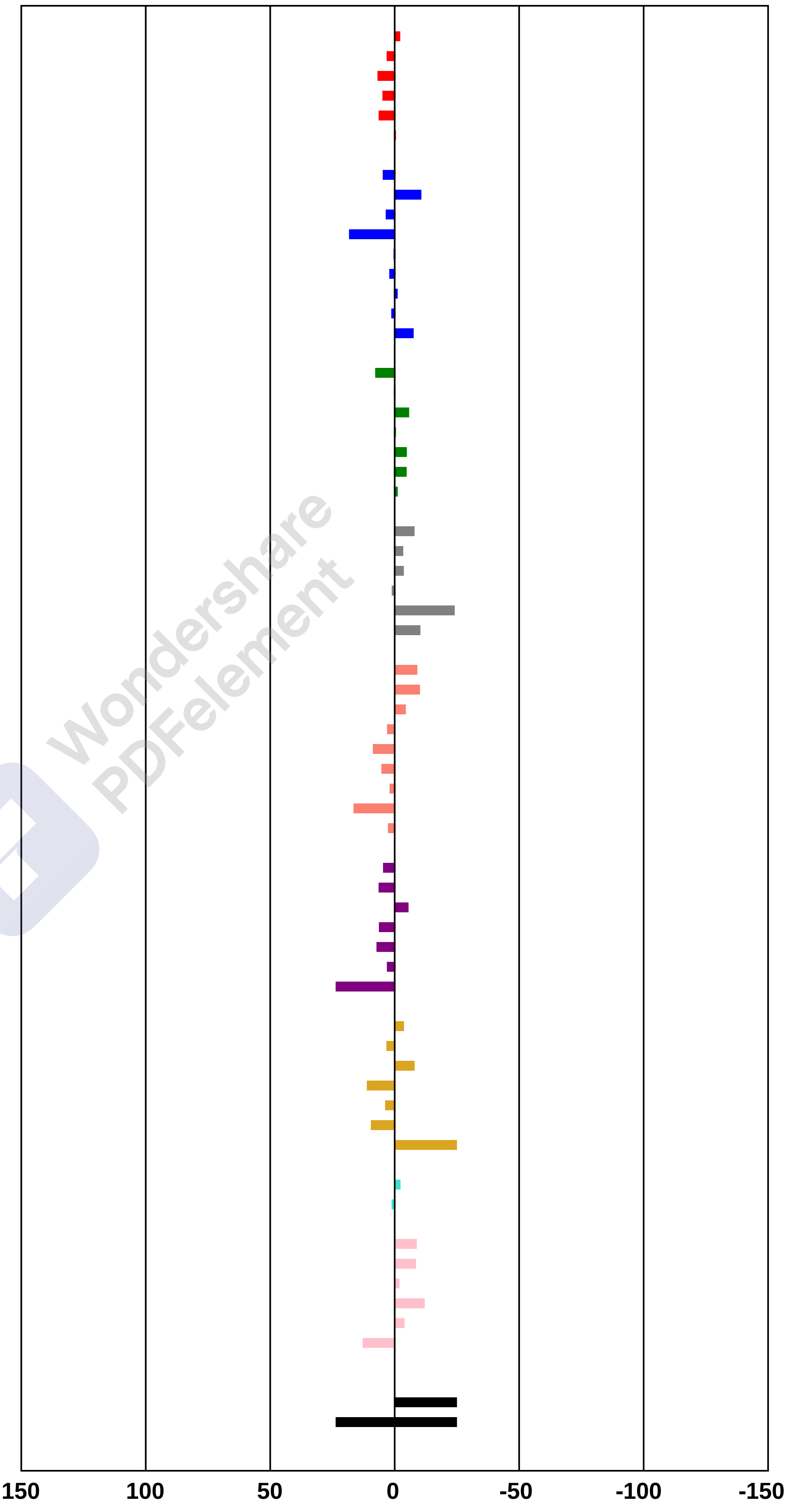

Wondershare PDFelement

Developmental Therapeutics Program
One Dose Mean Graph

NSC: D-820170 / 1 | **Conc** [illegible]
Experiment ID: 1910OS87 | Compound 12b | **Report Date:** [illegible]

Remove Watermark Wondershare PDFelement

Panel/Cell Line	Growth Percent
**Leukemia**	
CCRF-CEM	103.52
HL-60(TB)	102.40
K-562	101.69
MOLT-4	104.76
RPMI-8226	106.68
SR	108.25
**Non-Small Cell Lung Cancer**	
A549/ATCC	105.84
EKVX	92.11
HOP-62	100.49
HOP-92	112.35
NCI-H226	96.68
NCI-H23	97.53
NCI-H322M	95.94
NCI-H460	99.10
NCI-H522	81.76
**Colon Cancer**	
COLO 205	111.31
HCC-2998	108.55
HCT-116	99.29
HCT-15	99.68
HT29	95.30
KM12	101.38
SW-620	101.51
**CNS Cancer**	
SF-268	95.10
SF-295	102.51
SF-539	98.26
SNB-19	97.90
SNB-75	90.62
U251	92.88
**Melanoma**	
LOX IMVI	90.77
MALME-3M	95.25
M14	104.04
MDA-MB-435	100.90
SK-MEL-2	94.66
SK-MEL-28	104.05
SK-MEL-5	97.37
UACC-257	104.35
UACC-62	95.39
**Ovarian Cancer**	
IGROV1	97.08
OVCAR-3	99.38
OVCAR-4	99.93
OVCAR-5	107.59
OVCAR-8	103.67
NCI/ADR-RES	101.63
SK-OV-3	114.77
**Renal Cancer**	
786-0	105.90
ACHN	98.68
CAKI-1	97.14
RXF 393	110.44
SN12C	104.52
TK-10	107.72
UO-31	82.06
**Prostate Cancer**	
PC-3	98.65
DU-145	98.54
**Breast Cancer**	
MCF7	91.50
MDA-MB-231/ATCC	97.05
HS 578T	99.20
BT-549	105.57
T-47D	97.69
MDA-MB-468	97.02
Mean	100.10
Delta	18.34
Range	33.01

Mean Growth Percent - Growth Percent

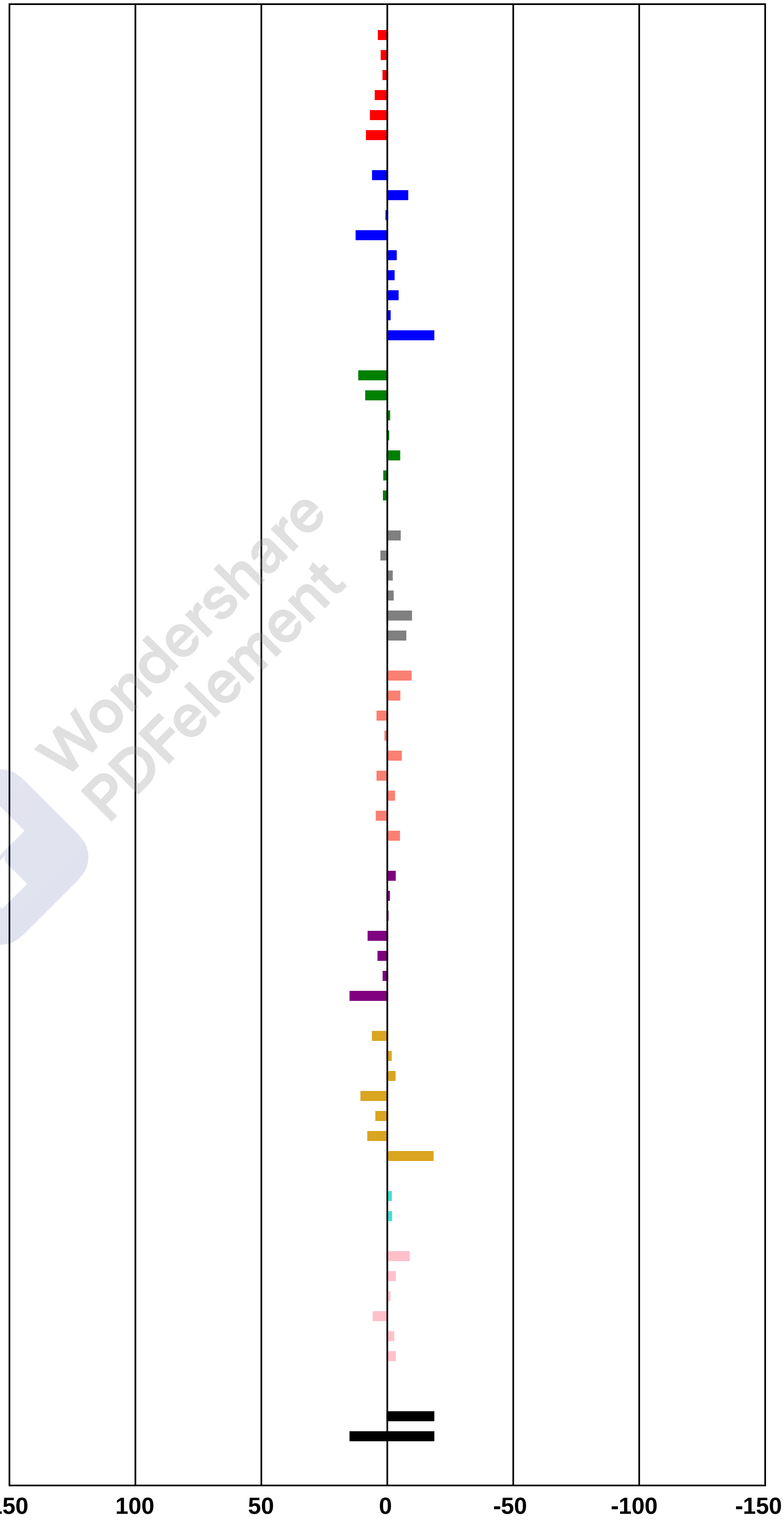

Wondershare PDFelement

Developmental Therapeutics Program

One Dose Mean Graph

NSC: D-820171 / 1 | **Conc** [illegible]

Experiment ID: 1910OS87 | Compound 11b | **Report Date:** [illegible]

Remove Watermark Wondershare PDFelement

Panel/Cell Line	Growth Percent
**Leukemia**	
CCRF-CEM	105.08
HL-60(TB)	103.99
K-562	115.17
MOLT-4	107.22
RPMI-8226	110.50
SR	112.27
**Non-Small Cell Lung Cancer**	
A549/ATCC	96.21
EKVX	90.77
HOP-62	103.23
HOP-92	119.18
NCI-H226	87.79
NCI-H23	96.67
NCI-H322M	100.74
NCI-H460	101.91
NCI-H522	94.61
**Colon Cancer**	
COLO 205	113.15
HCC-2998	103.81
HCT-116	101.62
HCT-15	99.06
HT29	104.43
KM12	99.57
SW-620	103.05
**CNS Cancer**	
SF-268	94.39
SF-295	100.08
SF-539	98.07
SNB-19	98.10
SNB-75	83.79
U251	97.12
**Melanoma**	
LOX IMVI	91.65
MALME-3M	107.03
M14	92.95
MDA-MB-435	100.85
SK-MEL-2	116.43
SK-MEL-28	105.30
SK-MEL-5	95.84
UACC-257	99.15
UACC-62	95.33
**Ovarian Cancer**	
IGROV1	104.14
OVCAR-3	108.90
OVCAR-4	91.01
OVCAR-5	101.91
OVCAR-8	99.26
NCI/ADR-RES	102.42
SK-OV-3	123.78
**Renal Cancer**	
786-0	101.34
ACHN	106.03
CAKI-1	95.62
RXF 393	104.10
SN12C	105.11
TK-10	109.96
UO-31	83.44
**Prostate Cancer**	
PC-3	102.03
DU-145	102.08
**Breast Cancer**	
MCF7	90.58
MDA-MB-231/ATCC	100.55
HS 578T	100.70
BT-549	96.14
T-47D	100.10
MDA-MB-468	83.04
Mean	100.99
Delta	17.95
Range	40.74

Mean Growth Percent - Growth Percent

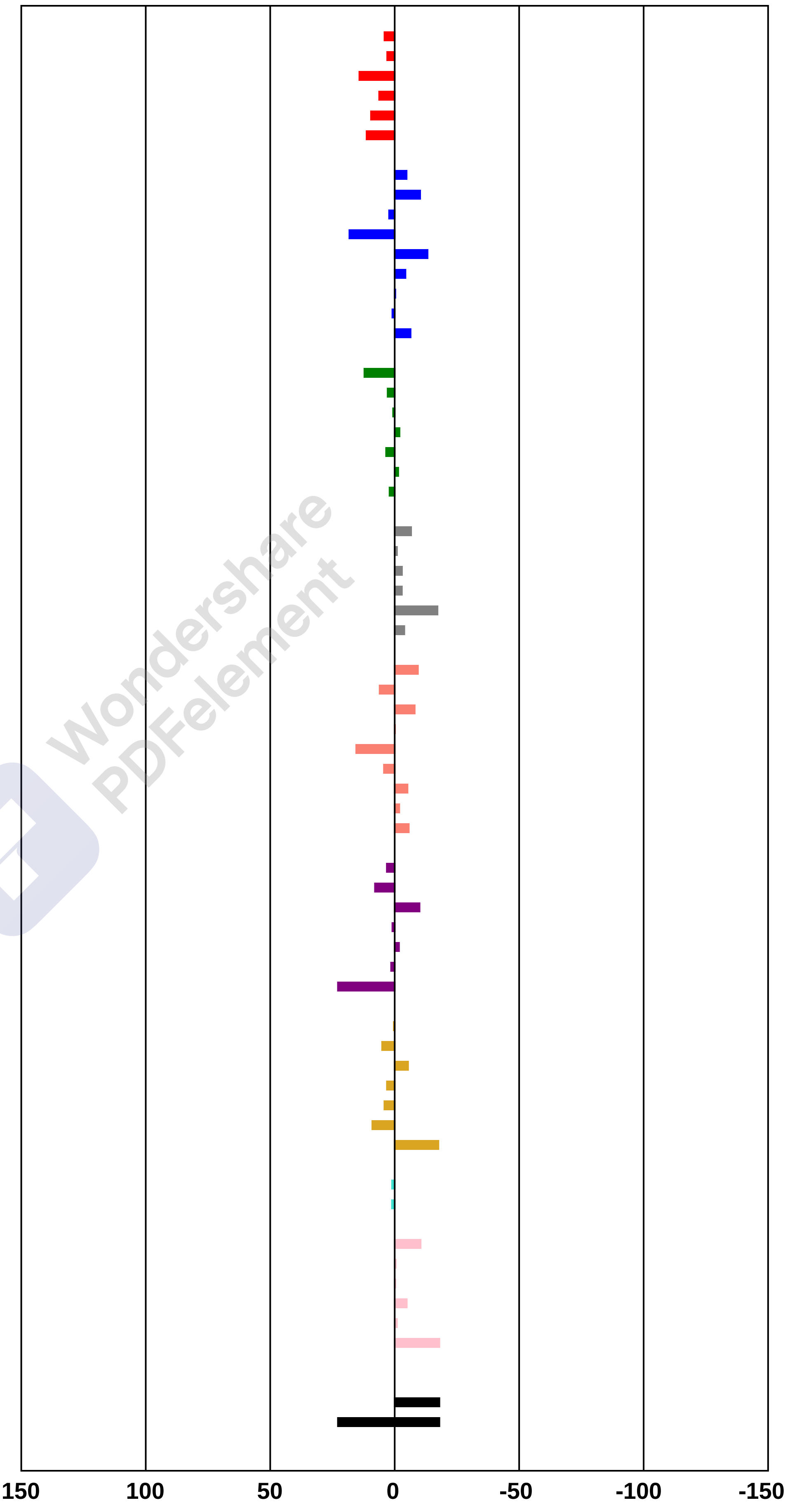

Wondershare PDFelement

Developmental Therapeutics Program

One Dose Mean Graph

NSC: D-820173 / 1 | **Conc** [illegible]

Experiment ID: 1910OS87 | Compound 7d | **Report Date:** [illegible]

Panel/Cell Line	Growth Percent
**Leukemia**	
HL-60(TB)	102.72
K-562	103.01
MOLT-4	111.35
SR	110.34
**Non-Small Cell Lung Cancer**	
A549/ATCC	101.33
EKVX	93.61
HOP-62	100.85
HOP-92	114.88
NCI-H226	103.88
NCI-H23	96.54
NCI-H322M	107.32
NCI-H460	102.05
NCI-H522	92.13
**Colon Cancer**	
COLO 205	112.97
HCC-2998	99.86
HCT-116	105.28
HCT-15	95.39
HT29	102.73
KM12	102.11
SW-620	104.86
**CNS Cancer**	
SF-268	98.17
SF-295	98.32
SF-539	100.31
SNB-19	96.45
SNB-75	87.95
U251	104.08
**Melanoma**	
LOX IMVI	93.76
MALME-3M	99.48
M14	104.30
MDA-MB-435	101.34
SK-MEL-2	104.16
SK-MEL-28	105.07
SK-MEL-5	101.24
UACC-257	99.45
UACC-62	94.97
**Ovarian Cancer**	
IGROV1	111.55
OVCAR-3	111.03
OVCAR-4	104.62
OVCAR-5	102.80
OVCAR-8	103.10
NCI/ADR-RES	99.55
SK-OV-3	120.74
**Renal Cancer**	
786-0	99.11
ACHN	105.43
CAKI-1	96.56
RXF 393	103.72
SN12C	100.86
TK-10	106.37
UO-31	85.06
**Prostate Cancer**	
PC-3	105.68
DU-145	106.96
**Breast Cancer**	
MCF7	95.28
MDA-MB-231/ATCC	94.96
HS 578T	104.57
BT-549	106.33
T-47D	112.91
MDA-MB-468	103.92
Mean	102.34
Delta	17.28
Range	35.68

Mean Growth Percent - Growth Percent

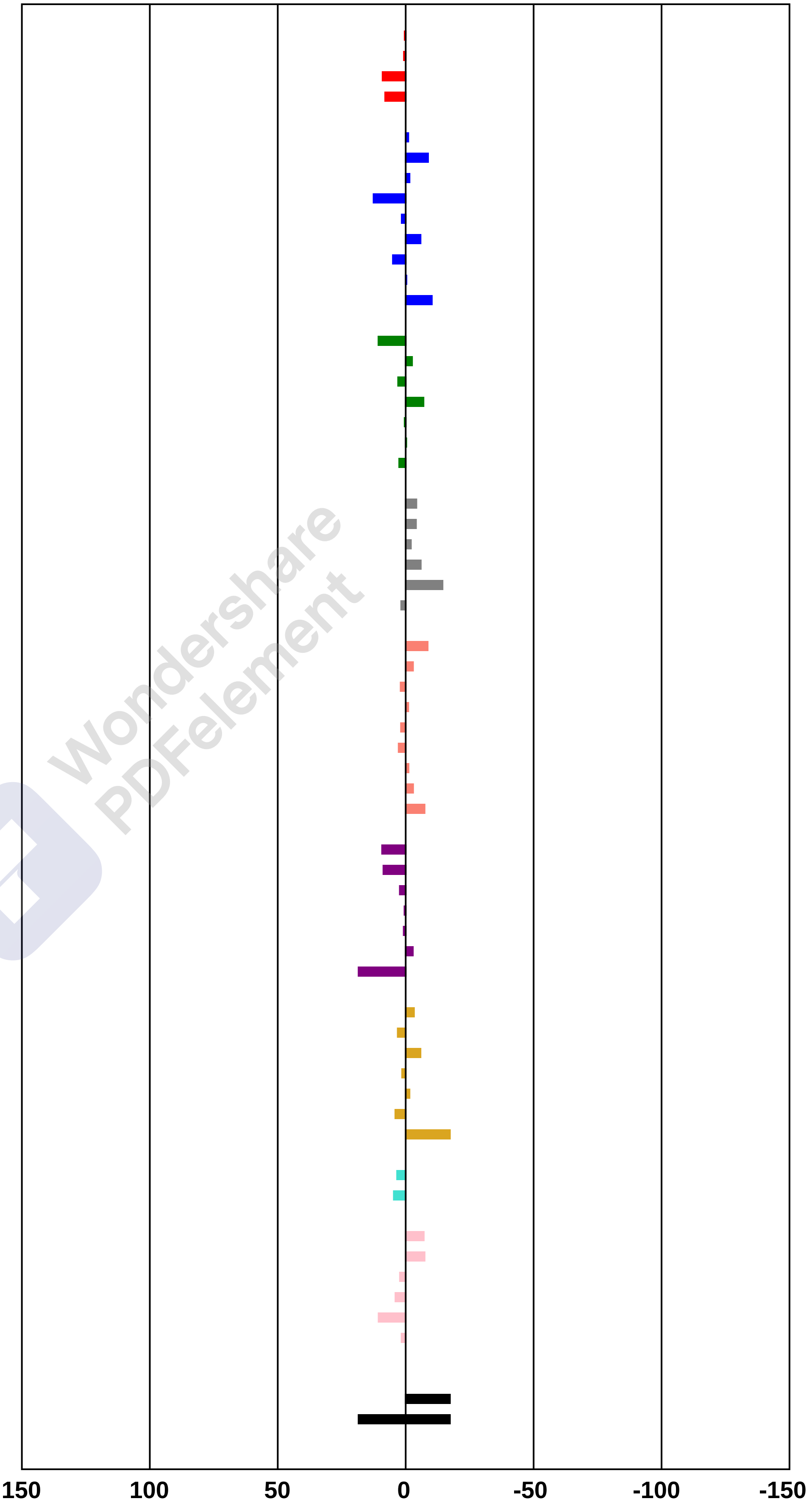

Wondershare PDFelement
Remove Watermark

Developmental Therapeutics Program

One Dose Mean Graph

**NSC:** D-820166 / 1	**Conc**[illegible]
**Experiment ID:** 1910OS87	Compound 20b
**Report Date:** [illegible]	

Panel/Cell Line	Growth Percent
Leukemia	
CCRF-CEM	102.73
HL-60(TB)	104.16
K-562	104.96
MOLT-4	109.45
RPMI-8226	102.20
SR	99.22
Non-Small Cell Lung Cancer	
A549/ATCC	98.87
EKVX	89.65
HOP-62	100.81
HOP-92	110.53
NCI-H226	97.52
NCI-H23	99.63
NCI-H322M	107.33
NCI-H460	99.46
NCI-H522	89.77
Colon Cancer	
COLO 205	106.57
HCC-2998	104.78
HCT-116	104.93
HCT-15	100.23
HT29	102.36
KM12	101.41
SW-620	102.07
CNS Cancer	
SF-268	97.16
SF-295	90.64
SF-539	96.91
SNB-19	97.59
SNB-75	95.60
U251	98.23
Melanoma	
LOX IMVI	96.54
MALME-3M	111.93
M14	112.35
MDA-MB-435	103.32
SK-MEL-2	102.90
SK-MEL-28	115.55
SK-MEL-5	97.62
UACC-257	103.93
UACC-62	96.85
Ovarian Cancer	
IGROV1	107.35
OVCAR-3	109.49
OVCAR-4	105.47
OVCAR-5	100.23
OVCAR-8	98.70
NCI/ADR-RES	100.67
SK-OV-3	125.12
Renal Cancer	
786-0	113.55
ACHN	103.87
CAKI-1	96.93
RXF 393	98.66
SN12C	100.34
TK-10	111.26
UO-31	88.29
Prostate Cancer	
PC-3	108.00
DU-145	111.21
Breast Cancer	
MCF7	92.13
MDA-MB-231/ATCC	110.91
HS 578T	102.24
BT-549	128.88
T-47D	104.47
MDA-MB-468	98.03
Mean	102.91
Delta	14.62
Range	40.59

Mean Growth Percent - Growth Percent

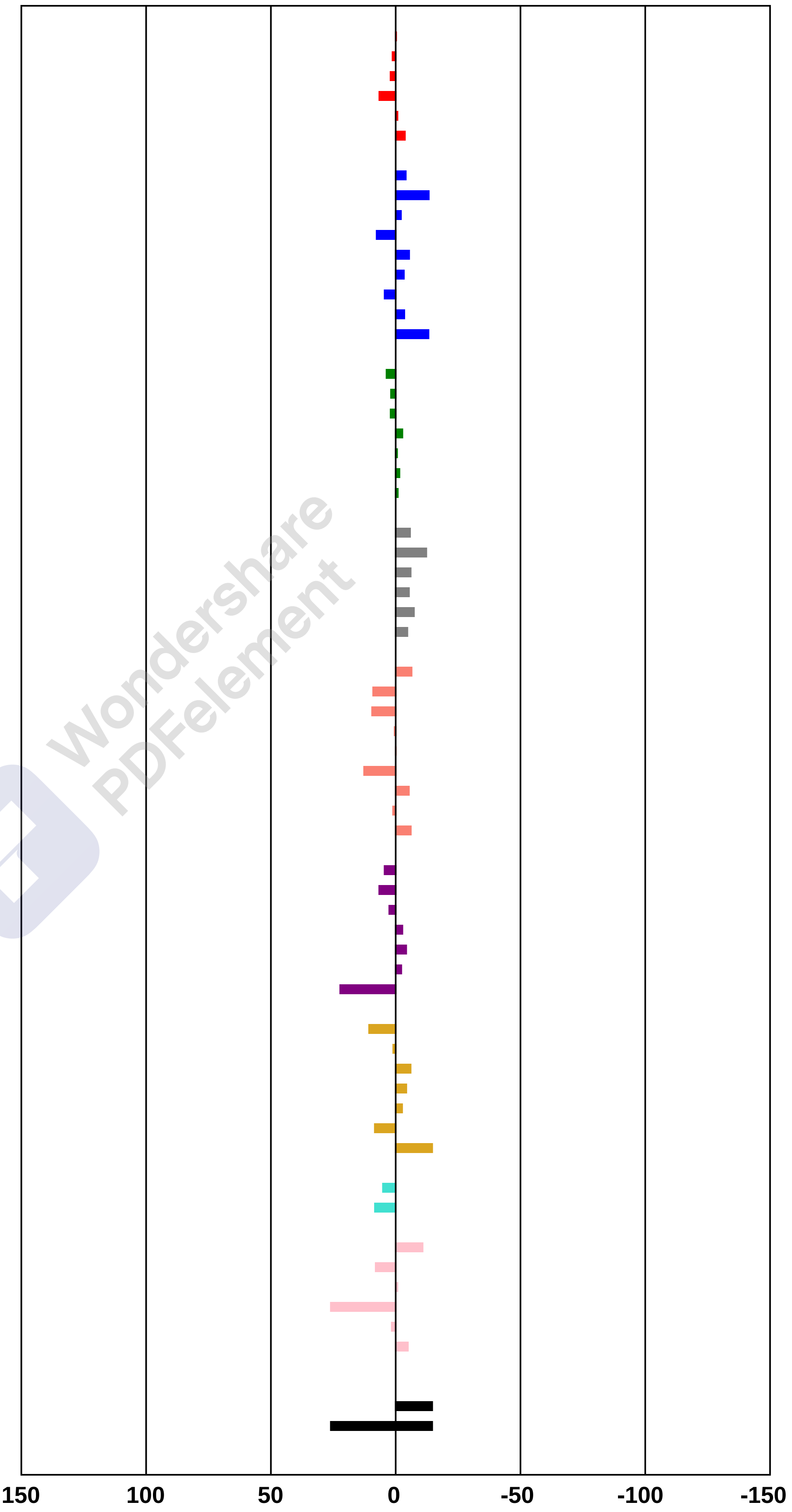

Developmental Therapeutics Program	NSC: D-820174 / 1	Conc[illegible]	
One Dose Mean Graph	Experiment ID: 1910OS87	Compound 19b	Report Date: Nov 25, 2019

Remove Watermark Wondershare PDFelement

Panel/Cell Line	Growth Percent
**Leukemia**	
CCRF-CEM	101.13
HL-60(TB)	103.70
K-562	101.92
MOLT-4	107.47
RPMI-8226	103.33
SR	104.58
**Non-Small Cell Lung Cancer**	
A549/ATCC	98.30
EKVX	87.34
HOP-62	100.46
HOP-92	102.50
NCI-H226	89.44
NCI-H23	95.71
NCI-H322M	99.06
NCI-H460	100.82
NCI-H522	90.58
**Colon Cancer**	
COLO 205	110.74
HCC-2998	97.70
HCT-116	105.69
HCT-15	95.07
HT29	104.96
KM12	101.98
SW-620	99.64
**CNS Cancer**	
SF-268	97.00
SF-295	91.88
SF-539	93.63
SNB-19	99.82
SNB-75	81.46
U251	101.54
**Melanoma**	
LOX IMVI	93.70
MALME-3M	102.54
M14	106.19
MDA-MB-435	102.98
SK-MEL-2	108.65
SK-MEL-28	107.91
SK-MEL-5	94.42
UACC-257	105.04
UACC-62	95.69
**Ovarian Cancer**	
IGROV1	101.82
OVCAR-3	115.34
OVCAR-4	104.94
OVCAR-5	102.09
OVCAR-8	99.45
NCI/ADR-RES	97.26
SK-OV-3	122.03
**Renal Cancer**	
786-0	102.58
ACHN	106.37
CAKI-1	96.21
RXF 393	94.17
SN12C	98.51
TK-10	108.29
UO-31	86.41
**Prostate Cancer**	
PC-3	105.18
DU-145	108.08
**Breast Cancer**	
MCF7	92.31
MDA-MB-231/ATCC	101.73
HS 578T	103.85
BT-549	109.16
T-47D	101.18
MDA-MB-468	92.23
Mean	100.57
Delta	19.11
Range	40.57

Mean Growth Percent - Growth Percent

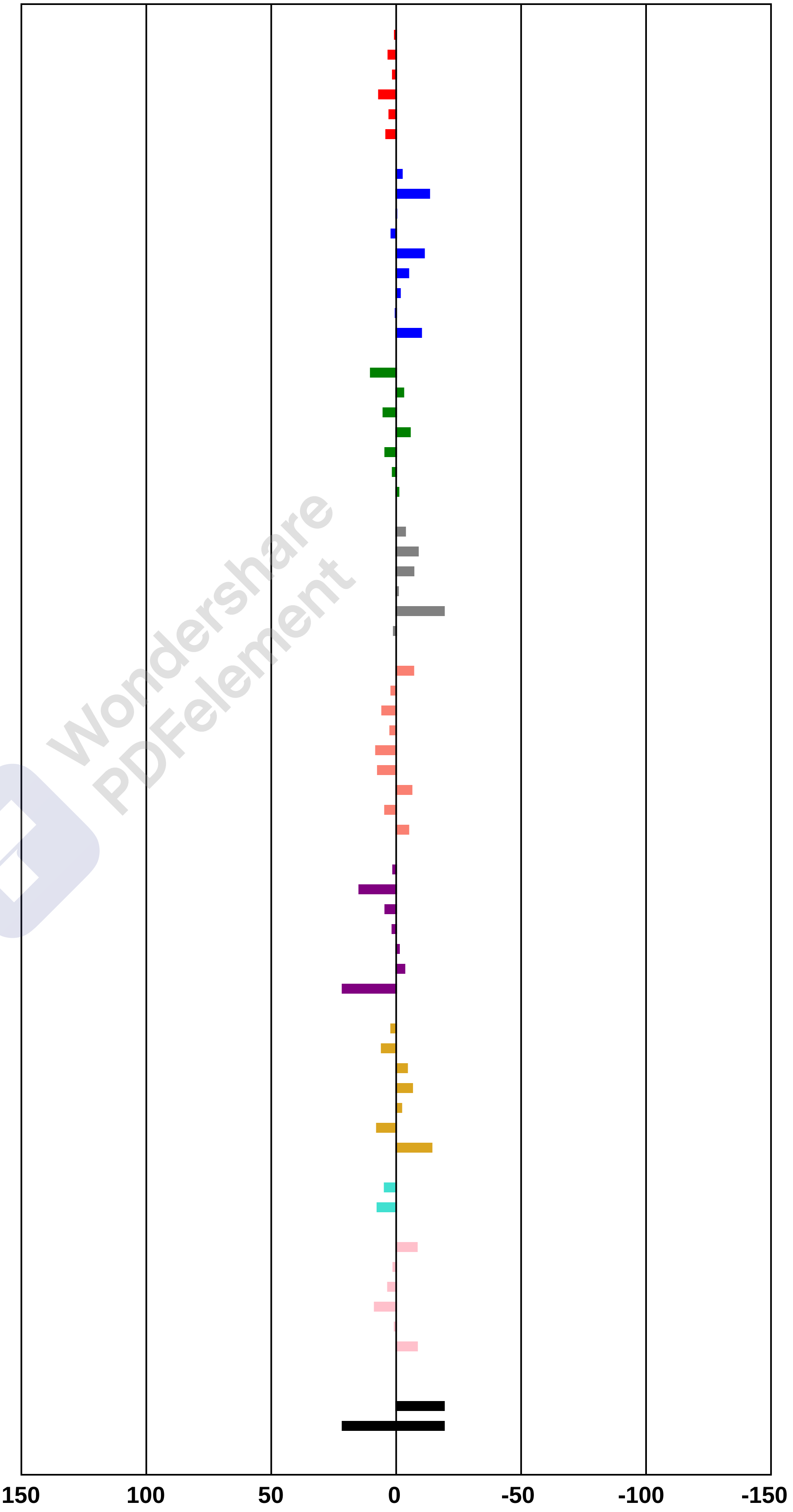

Wondershare PDFelement

Developmental Therapeutics Program One Dose Mean Graph	NSC: D-820176 / 1	Conc [illegible]
	Experiment ID: 1910OS87 Compound [illegible]	Report Date: Nov [illegible], 2019

Remove Watermark Wondershare PDFelement

Panel/Cell Line	Growth Percent
**Leukemia**	
CCRF-CEM	98.78
HL-60(TB)	103.03
K-562	94.99
MOLT-4	103.50
RPMI-8226	106.04
SR	78.20
**Non-Small Cell Lung Cancer**	
A549/ATCC	98.37
EKVX	92.76
HOP-62	103.61
HOP-92	99.83
NCI-H226	89.32
NCI-H23	90.51
NCI-H322M	101.81
NCI-H460	101.66
NCI-H522	86.62
**Colon Cancer**	
COLO 205	115.62
HCC-2998	99.98
HCT-116	102.90
HCT-15	91.34
HT29	100.52
KM12	102.03
SW-620	89.53
**CNS Cancer**	
SF-268	91.83
SF-295	91.96
SF-539	92.86
SNB-19	93.06
SNB-75	74.29
U251	90.06
**Melanoma**	
LOX IMVI	96.03
MALME-3M	101.18
M14	100.48
MDA-MB-435	98.58
SK-MEL-2	109.33
SK-MEL-28	100.91
SK-MEL-5	99.02
UACC-257	107.03
UACC-62	92.58
**Ovarian Cancer**	
IGROV1	87.70
OVCAR-3	96.92
OVCAR-4	94.72
OVCAR-5	98.41
OVCAR-8	104.46
NCI/ADR-RES	96.17
SK-OV-3	125.83
**Renal Cancer**	
786-0	98.65
ACHN	95.07
CAKI-1	77.61
RXF 393	97.22
SN12C	92.82
TK-10	123.28
UO-31	72.78
**Prostate Cancer**	
PC-3	100.15
DU-145	100.13
**Breast Cancer**	
MCF7	87.27
MDA-MB-231/ATCC	100.34
HS 578T	104.75
BT-549	112.44
T-47D	90.83
MDA-MB-468	80.80
Mean	97.09
Delta	24.31
Range	53.05

Mean Growth Percent - Growth Percent

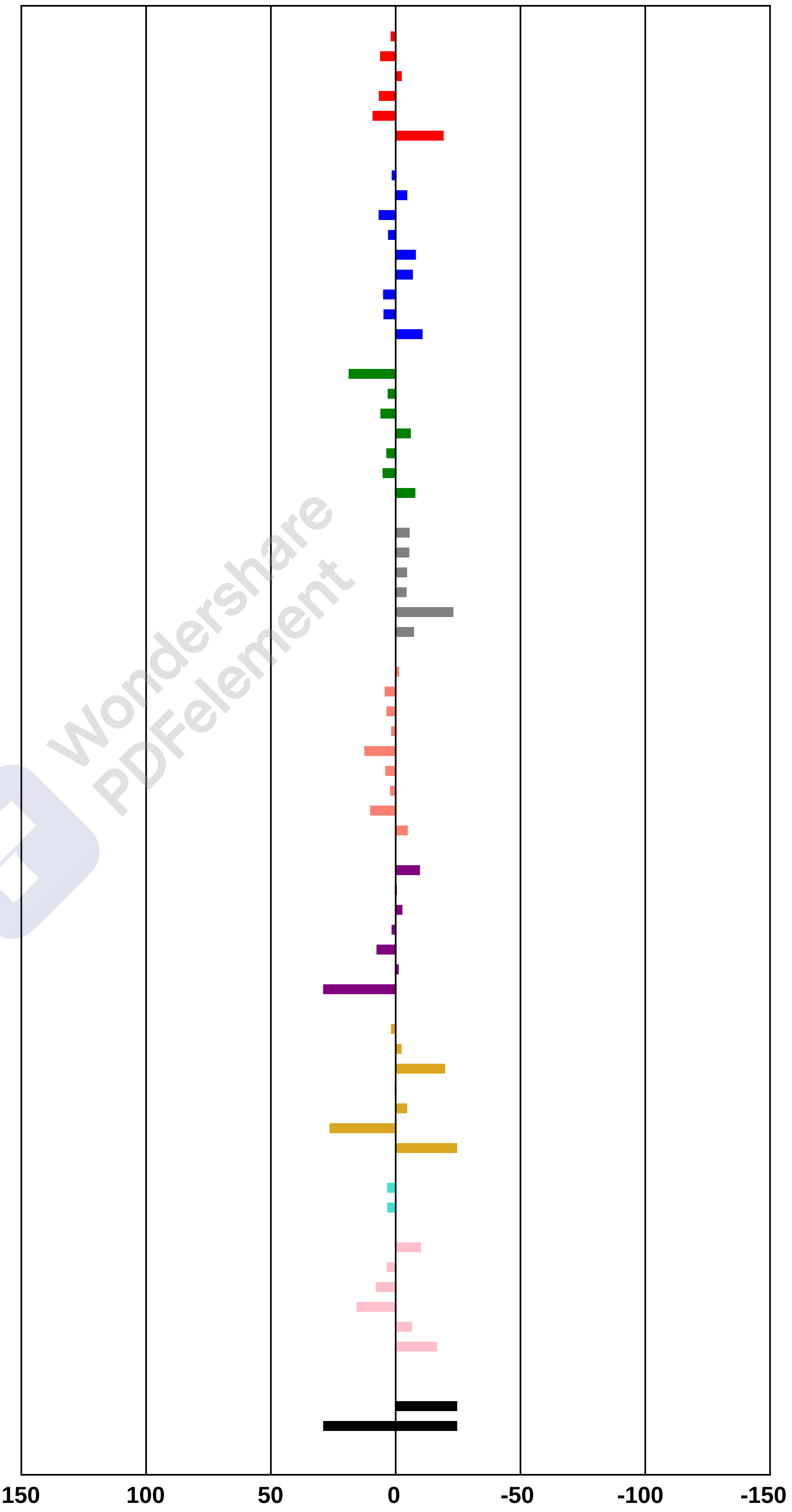

Wondershare PDFelement
